# The role of allyl ammonium salts in palladium-catalyzed cascade reactions towards the synthesis of spiro-fused heterocycles

**DOI:** 10.1038/s41467-020-19110-3

**Published:** 2020-10-23

**Authors:** Fei Ye, Yao Ge, Anke Spannenberg, Helfried Neumann, Matthias Beller

**Affiliations:** 1 Key Laboratory of Organosilicon Chemistry and Material Technology of Ministry of Education, and Key Laboratory of Organosilicon Material Technology of Zhejiang Province, Hangzhou Normal University, No. 2318, Yuhangtang Road, 311121 Hangzhou, PR China; 2 Leibniz-Institute for Catalysis, Albert-Einstein-Str. 29a, 18059 Rostock, Germany

**Keywords:** Catalytic mechanisms, Homogeneous catalysis, Synthetic chemistry methodology

## Abstract

There is a continuous need for designing new and improved synthetic methods aiming at minimizing reaction steps while increasing molecular complexity. In this respect, catalytic, one-pot cascade methodologies constitute an ideal tool for the construction of complex molecules with high chemo-, regio-, and stereoselectivity. Herein, we describe two general and efficient cascade procedures for the synthesis of spiro-fused heterocylces. This transformation combines selective nucleophilic substitution (S_N_2′), palladium-catalyzed Heck and C–H activation reactions in a cascade manner. The use of allylic ammonium salts and specific Pd catalysts are key to the success of the transformations. The synthetic utility of these methodologies is showcased by the preparation of 48 spiro-fused dihydrobenzofuranes and indolines including a variety of fluorinated derivatives.

## Abstract (web summary)

Synthetic methods aiming at minimizing reaction steps while increasing molecular complexity are highly sought after by organic chemists. Here, the authors report two cascade procedures combining nucleophilic substitution, palladium-catalyzed Heck and C–H activation reactions for the synthesis of spiro-fused heterocycles.

## Introduction

The development of novel chemical transformations increasing molecular complexity enables significant innovation potential in life and material sciences. In this respect, catalytic cascade or domino processes offer strong impetus for new methodology developments^1–3^. Compared to traditional consecutive procedures, they permit several practical advantages: In addition to improved step-economy, waste generation from multiple iterations of reaction, workup, and purification procedures are minimized. Consequently, diverse and complex organic molecules can be assembled not only in a faster, but also more sustainable way.

**Fig. 1:**
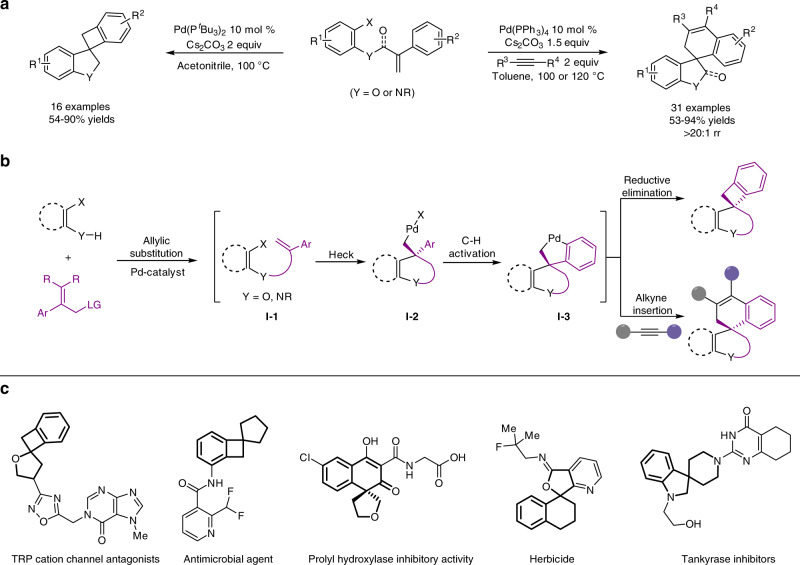
Concepts. **a** Previous work: palladium-catalyzed intramolecular cyclization for the synthesis of spiroheterocycles. **b** This work: palladium-catalyzed allylic substitution/Heck/C–H activation(/alkyne) cascade processes. **c** Selected examples of related bio-active spiro compounds.

**Fig. 2:**
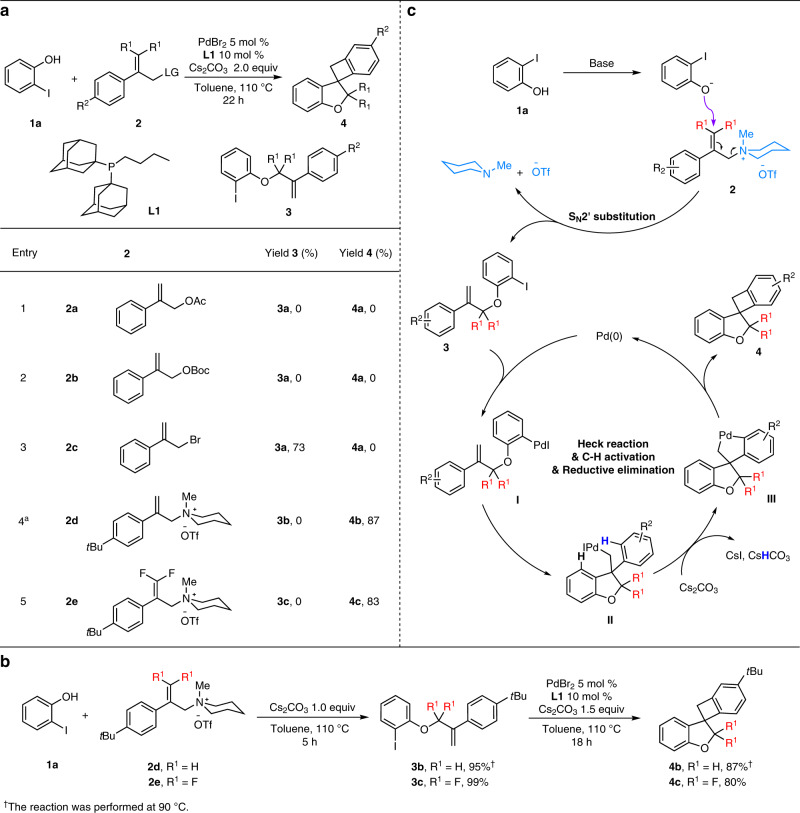
Constructing molecular complexity: spiro-fused heterocycles from simple starting materials. **a** Preliminary results show the importance of ammonium salts as the partners of the nucleophile. **b** Control experiments shown that the allylic aryl ether **3** was formed in situ as important intermediate during the cascade procedure. **c** A S_N_2’ substitution process was proposed for the first reaction step, followed by sequence palladium-catalyzed Heck reaction/C–H activation/Pd reductive elimination to provide the target product **4**.

Since their discovery in the 1970s and 80s^4–8^, palladium-catalyzed C–C bond forming reactions have become the most popular homogeneous catalytic processes in organic chemistry and industrial fine chemical synthesis^9–12^. Their ability to form (stereo)selectively carbon-carbon bonds under mild conditions made them “a true power tool for organic synthesis”^13^. Specifically, the intramolecular Heck reaction provides an entry to useful palladium complexes with quaternary carbon centers as intermediates (Fig. 1b, **I**–**2**), which can be further valorized to a multitude of valuable building blocks^14–18^. Notably, the combination of this reaction with C–H activation processes has also been studied, giving access to structurally unique spiro compounds^19–31^, which possess interesting biological activities (Fig. 1c)^32–38^. However, the necessity of pre-synthesized starting materials in the existing methods limits the full exploitation of this elegant concept and is often tedious. In our quest for the development of new cascade methodologies, we had the idea to provide a more facile entry to this class of compounds by combining three (or four) palladium-catalyzed coupling processes, namely Tsuji–Trost and Heck reactions followed by selective C–H activation (and alkyne insertion) to assemble complex organic molecules from easily available substrates (Fig. 1).

Herein, we describe our recent efforts to establish a palladium-catalyzed allylic substitution/Heck/C–H activation(/alkyne) cascade processes for the synthesis of spiro-fused heterocycles. Key challenges of such processes are obviously the compatibility of the well-matched reactant partners^39^, the required conditions of the individual reactions, the development of a general catalyst system able to promote all three (or four) transformations efficiently, and to achieve the needed high chemo-selectivity, regio-selectivity, and stereoselectivity throughout all elementary steps.

## Results

### Reaction development

Recently, Lautens, Schoenebeck and co-workers reported the synthesis of spiro-fused heterocycles through an intramolecular Heck/C–H activation sequence using specific alkene-tethered aryl iodides (Fig. 1a)^40–42^. Regarding the starting materials, those substrates might be preferably prepared through an initial palladium-catalyzed Tsuji-Trost allylation of 2-halophenols, which would provide a more efficient and step-economic way^43–45^. Following this initial idea, we investigated the coupling of 2-iodophenol (**1a**) with 2-phenylallyl acetate (**2a**) in the presence of PdBr_2_/**L1** (Fig. 2a) and various other palladium catalysts (for details see Supplementary Information, Supplementary Table 1). Unfortunately, in no case the desired spiro-fused product **4a** was observed and no conversion took place. Similar results were obtained when the *tert*-butyl (2-phenylallyl) carbonate (**2b**) was used instead of **2a**. When more reactive 2-phenylallyl bromide **2c** was introduced, only the alkene-tethered aryl iodide **3a** was isolated in 73% yield instead of product **4a**. To improve the reactivity of the starting material further on, other allylic leaving groups were considered. In this respect, allylic ammonium salts, which have been largely neglected in intermolecular palladium-catalyzed allylic substitutions, attracted our attention^39,46,47^. This class of compounds are in general highly stable and can be conveniently prepared from a variety of amines. Surprisingly, testing **2d** in the presence of the PdBr_2_/**L1** catalyst, the desired cascade process took place and product **4b** was obtained in 87% isolated yield! This means that each individual step proceeds with an efficiency of at least 95%. At this point, it should be mentioned that allylic ammonium salts are also known to undergo direct S_N_2-substitution or S_N_2’-substitution reactions under basic conditions^48–50^. To understand whether the first reaction step is really palladium-catalyzed, **2d** was treated with **1a** in the presence of 1 equiv. of base. Interestingly, the allyl aryl ether **3b** was obtained in high yield (95%). Subsequent reaction in the presence of our regular palladium catalyst led to full consumption of **3b**, providing the desired spiro-benzocyclobutane **4b** in 87% yield (Fig. 2b). Obviously, applying substrate **2d** does not allow to distinguish between S_N_2-mechanism and S_N_2’-mechanism in the first reaction step due to its symmetry. Based on the actual interest in fluorinated building blocks^51–53^, the *gem*-difluorinated allylic ammonium salt **2e**^39^ was reacted with 2-iodophenol, which gave product **3c** in 99% yield and excellent regioselectivity. Again, the following palladium-catalyzed steps took place smoothly and provided **4c** in high yield (80%). Similarly, the direct conversion of **2e** proceeded efficiently to give **4c** in 83% isolated yield (Fig. 2a, entry 5). It should be noted that related fluorinated heterocycles in general cannot be easily prepared^54^ and that to the best of our knowledge no example of such spiro compounds has been reported yet. Apart from **2e**, other related ammonium salts **2f**–**2i** underwent similar coupling processes to provide the desired product **4c** in slight lower yield (62–75%) (for details see Supplementary Information, Supplementary Table 2).

To obtain optimal results, an extensive evaluation of the reaction conditions of the model systems was performed (for details see Supplementary Information, Supplementary Tables 1–9) and revealed three significant points: (1) The catalyst system is crucial in this cascade process and only in the presence of sterically hindered and electron-rich diadamantyl phosphines such as **L1**, the desired product was obtained in high yield. (2) Cs_2_CO_3_ and toluene were independently identified as the most effective base and solvent, which nearly doubled the product yield compared to other common bases and solvents. (3) In addition, the concentrations of substrates are decisive. Using an equimolar amount of both substrates led to the best result while an excess of either ammonium salt or aryl halide considerably decreased the yield of **4**. Based on all these observations, a plausible mechanism for the formation of benzocyclobutane derivative **4** is proposed in Fig. 2c: Initially, 2-iodophenol **1** and ammonium salt **2** underwent a base mediated S_N_2’ allylic substitution in a highly regioselective manner. To further confirm the S_N_2’ route, deuterium substituted ammonium salt was tested, details see Supplementary Information, Supplementary Fig. 1. Next, intramolecular palladium-catalyzed Heck reaction of the in situ generated compound **3** followed by site-selective C–H activation forms the spiropalladacycle **III**. Final reductive elimination regenerates the palladium species and produces the desired product^40^. Noteworthily, this novel cascade reaction is a rare example of a domino process involving S_N_2´ substitution with subsequent metal-catalyzed transformations^55,56^.

### Scope for the formation of spiro-fused benzocyclobutene derivatives

**Fig. 3:**
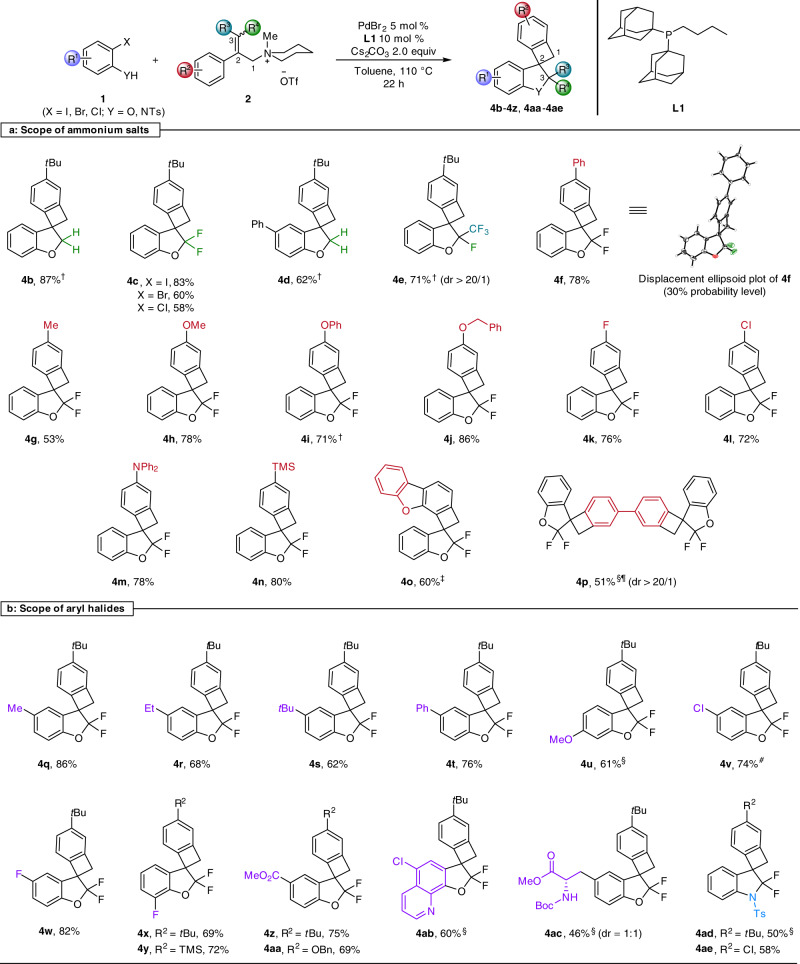
Palladium-catalyzed cascade reaction of ammonium salts with aryl halides. Standard reaction conditions: **1** (0.2 mmol), **2** (0.2 mmol), Cs_2_CO_3_ (0.4 mmol), PdBr_2_ (0.01 mmol), **L1** (0.02 mmol), toluene (2.5 mL), the reaction mixture was performed at 110 °C under argon atmosphere for 22 h, isolated yield, the diastereoselectivity of **4e**, **4p**, and **4ac** was determined by crude ^19^F NMR analyses. ^†^The reaction mixture was performed at 90 °C. ^‡^1 mol% PdBr_2_ and 2 mol% **L1** were used. ^§^10 mol% PdBr_2_ and 20 mol% **L1** were used. ^¶^**1** (0.2 mmol), **2** (0.1 mmol) were used. ^#^2 mol% PdBr_2_ and 4 mol% **L1** were used.

With the optimized reaction conditions in hand, the general feasibility of this approach was examined. As shown in Fig. 3, allylic ammonium salts with different substituents in 3-position including H, F, CF_3_, directly afforded **4b**–**4e** in all cases in good to high isolated yields. For disubstituted substrate **2j** with –F and –CF_3_ substituents in 3-position, high diastereoselectivity for two adjacent quaternary carbon centers was obtained (**4e**, 71%). Next, the reaction of *gem*-difluorinated allylic ammonium salts **2k**–**2u** with aryl halides **1a**–**1o** (for details see Supplementary Information, Supplementary Fig. 2) was investigated. Most of the ammonium salts were conveniently obtained from commercially available phenylboronic acid and vinyl bromides via Suzuki reaction, base-mediated amination, and final *N*-methylation^39^. With regard to the cascade reaction, both electron-donating groups including alkyl, aryl, alkoxy and aryloxy and electron-withdrawing groups including fluoro and chloro were perfectly compatible with the conditions, and the corresponding products **4f**–**4l** were obtained in 53 − 86% yield. The molecular structure of these highly strained 5,4-spirocycles was unambiguously confirmed by X-ray crystal structure analysis of **4f**. Both diphenylamino-substituted and trimethylsilyl-substituted spiro compounds **4m** and **4n** were successfully formed in high yield. Furthermore, dibenzofuran-derived ammonium salt underwent the cascade process, leading to the construction of the heterocycle-embedded tetracyclic framework **4o** in 60% yield. Gratifyingly, the more complex derivative **4p** containing two spiro-fused benzocyclobutanes was smoothly generated in 51% yield via a consecutive two-fold cascade process using the corresponding bis-ammonium salt as the reagent.

Next, we explored the scope of our methodology with respect to the aryl halide coupling partner. In addition to 2-iodophenols **1a**-**1l**, 2-bromophenol **1m**, 2-chlorophenol **1n**, and 2-iodoaniline **1o** were also investigated. The latter case highlights the possibility to construct 3-spiro-indolines, specifically 2-fluorinated indolines (**4ad** and **4ae**), which are of interest as natural products and pharmaceutical molecules^57,58^. As depicted in Fig. 3, several different aryl halides gave the expected tetracyclic products under the standard conditions. Interestingly, considering the three-step cascade, these transformations proceeded in good to excellent yields with either electron-rich or electron-deficient substituents. Notably, substrates containing heteroarenes, such as the quinoline derivative **1k**, provided the *N*,*O*-fused heterocycle **4ab** in 60% yield. Moreover, the *L*-tyrosine derived product **4ac** was obtained by a concise cascade transformation (dr = 1:1).

### Three-component spirocyclization reaction

Considering the versatility of the in-situ-generated palladacycles **III**^16,26,27^, subsequent functionalization including carbene and alkyne insertion should allow for the efficient construction of other classes of novel spiro compounds^29,30,41,42^. To demonstrate this synthetic potential, we performed the reaction of **1a** and **2e** with two equiv. of an additional unsymmetrical alkyne **5a** (ethyl 3-phenyl-propynoate). Indeed, the envisioned cascade process combining S_N_2’ substitution, palladium-catalyzed Heck/C–H activation and final alkyne insertion provided in a straightforward manner only one regioisomer of the respective 6,5-spirocycles **6** (regioselectivity: >20:1). Under standard conditions, the desired product **6a** was obtained in 73% yield; however, in this case the highly reactive palladacycle **III** also underwent minor reductive elimination and the 5,4-spirocycle **4c** was detected in 14% yield. Pleasantly, increasing substrate concentration in the presence of the extended ligand **L3** provided exclusively **6a** in high yield (85% isolated yield; for a brief evaluation of reaction conditions see Supplementary Information, Supplementary Table 10).

**Fig. 4:**
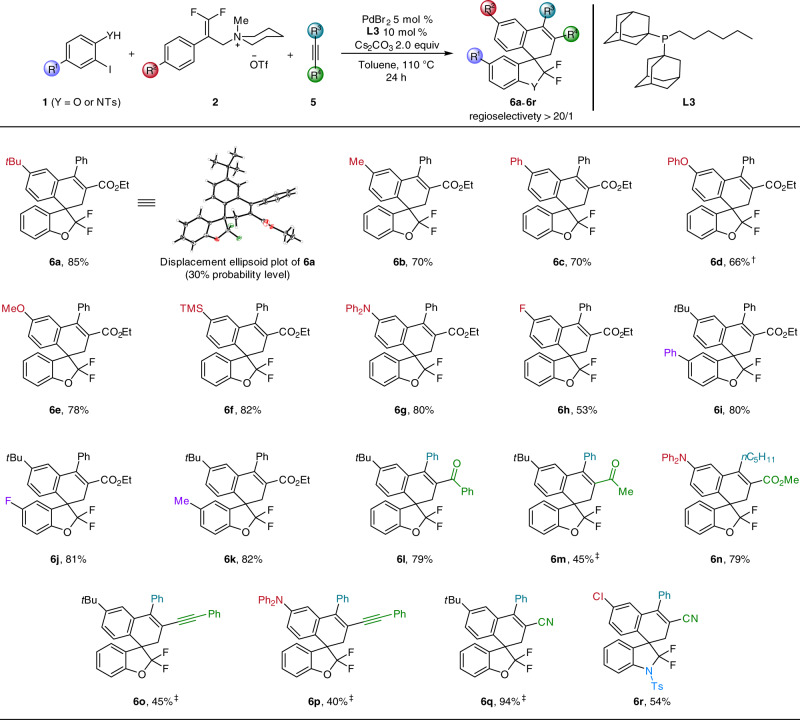
Palladium-catalyzed three-component cascade spriocyclizations. Standard reaction conditions: **1** (0.2 mmol), **2** (0.2 mmol), **5** (0.4 mmol), Cs_2_CO_3_ (0.4 mmol), PdBr_2_ (0.01 mmol), **L3** (0.02 mmol), toluene (1.0 mL), the reaction mixture was stirred at 110 °C under argon atmosphere for 24 h, isolated yield, the regioselectivity of **6** was determined by crude ^19^F NMR analyses. ^†^2 mol% PdBr_2_ and 4 mol% **L3** were used. ^‡^10 mol% PdBr_2_ and 20 mol% **L3** were used.

The generality of this second three-component cascade procedure is shown by variation of five aryl iodides, nine ammonium salts and six alkynes (Fig. 4). In all cases, the domino reaction proceeded smoothly with valuable substituents and functional groups, including alkoxy, aryloxy, halide, silyl, and amino, giving the corresponding products **6a**–**6h** in good to high yields with excellent regioselectivities. The molecular structure of **6a** was confirmed by X-ray crystallography. Substituents on the phenyl ring of aryl iodide displayed only a minor influence on the reactivity and provided **6i**–**6k** in high yields. Notably, various unsymmetrical alkynes with different substituents on the triple bond afforded **6l**–**6q** with excellent degrees of both chemoselectivities and regioselectivities. For example, internal alkynes bearing −COPh, −COCH_3_, and −CO_2_Me substituents gave the corresponding products **6l**–**6n** in 79%, 45 and 79% isolated yield, respectively. It is worthy to note that 3-phenyl-2-propynenitrile and 1,3-diynes were compatible in this transformation, affording **6o**–**6q** in 40–94% isolated yield. Finally, the construction of 3-spiro-indoline **6r** was also achieved in 54% yield.

## Discussion

In summary, we have developed two efficient cascade processes involving allylic substitution (via S_N_2’-mechanism), palladium-catalyzed Heck, remote C–H activation, and reductive elimination or alkyne insertion for the straightforward synthesis of 5,4-spiroheterocycles and 6,5-spiroheterocycles in good to high yields with excellent selectivities. Crucial for the success of these transformations is the use of specifically activated allylic substrates (ammonium salts) in combination with special PdBr_2_/AlkylPAd_2_ catalytic systems. Under optimal conditions diverse (fluorinated) spiro-dihydrobenzofurans and spiro-indolines are achieved in an unprecedented fast and step-economic way, without need for purification of intermediates. We believe these methodologies demonstrate the potential of catalytic cascade processes for a straightforward increase of molecular complexity of simple and easily available aryl halides.

## Methods

### General procedure for the preparation of spiro-fused benzocyclobutanes 4

To a 25 ml oven-dried pressure tube equipped with a magnetic stir bar were added 2-halophenol or aniline **1** (0.2 mmol), ammonium salt **2** (0.2 mmol), Cs_2_CO_3_ (130 mg, 0.4 mmol), PdBr_2_ (2.7 mg, 0.01 mmol), **L1** (7.2 mg, 0.02 mmol), and then degassed toluene (2.5 mL) was introduced under argon atmosphere. The sealed pressure tube was heated and stirred at 110 °C for 22 h. The reaction mixture was allowed cooling to room temperature, diluted with ethyl acetate (10 ml), and filtered through a short pad of celite eluting with ethyl acetate (3 × 10 ml). After evaporation, the residue was purified by chromatography on basic aluminum oxide (It is worthy to note that the 2-fluorinated product can only be separated without decomposition using basic aluminum oxide) to afford the desired product **4**.

### General procedure for the preparation of spiro-fused dihydronaphthalenes 6

To a 25 ml oven-dried pressure tube equipped with a magnetic stir bar were added 2-halophenol or aniline **1** (0.2 mmol), ammonium salt **2** (0.2 mmol), alkyne **5** (0.4 mmol), Cs_2_CO_3_ (130 mg, 0.4 mmol), PdBr_2_ (2.7 mg, 0.01 mmol), **L3** (7.7 mg, 0.02 mmol), and then degassed toluene (1 ml) was introduced under argon atmosphere. The sealed pressure tube was heated and stirred at 110 °C for 24 h. The reaction mixture was allowed cooling to room temperature, diluted with ethyl acetate (10 ml), and filtered through a short pad of celite eluting with ethyl acetate (3 × 10 ml). After evaporation, the residue was purified by chromatography on basic aluminum oxide (It is worthy to note that the 2-fluorinated product can only be separated without decomposition using basic aluminum oxide) to afford the desired product **6**.

## Supplementary information

Supplementary Information

## Data Availability

The authors declare that all the data supporting this study, including the experimental details, data analysis, and spectra for all unknow compounds, see Supplementary Files. All data underlying the findings of this work are available from the corresponding author upon reasonable request. The X-ray crystallographic coordinates for structures reported in this study have been deposited at the Cambridge Crystallographic Data Centre (CCDC), under deposition numbers 2006609 (**4f**) and 2006610 (**6a**). These data are provided free of charge by the joint Cambridge Crystallographic Data Centre and Fachinformationszentrum Karlsruhe Access Structures service www.ccdc.cam.ac.uk/structures.
