# Supplementary material for: The role of allyl ammonium salts in palladium-catalyzed cascade reactions towards the synthesis of spiro-fused heterocycles
Source: Nat Commun. 2020 Oct 23;11:5383. doi: 10.1038/s41467-020-19110-3 (PMC7584656; doi:10.1038/s41467-020-19110-3)
Supplement: Supplementary file 1 — Supplementary Information [file 41467_2020_19110_MOESM1_ESM.pdf]

Supplementary Information

The Role of Allyl Ammonium Salts in Palladium-catalyzed Cascade Reactions towards the Synthesis of Spiro-fused Heterocycles

Fei Ye†, Yao, Ge†, Anke Spannenberg, Helfried Neumann, and Matthias Beller*

[1] Leibniz-Institut für Katalyse e.V., Albert-Einstein-Str. 29a, 18059 Rostock, Germany

[2] Key Laboratory of Organosilicon Chemistry and Material Technology of Ministry of Education, and Key Laboratory of Organosilicon Material Technology of Zhejiang Province, Hangzhou Normal University, No. 2318, Yuhangtang Road, 311121 Hangzhou, PR China

† These authors contributed equally to this work.

* Correspondence to: matthias.beller@catalysis.de

Table of contents

Table of contents 1
1. General information 2
2. Experimental section 3
2.1 Chemical list 3
2.2 General procedure for the synthesis of ammonium salts 2: 5
2.3 General procedure for the preparation of spiro-fused benzocyclobutanes 4 and dihydronaphthalenes 6: 5
2.4 Optimization of reaction conditions 7
3. Characterization of new compounds 17
4. X-ray crystal structure analysis 61
5. NMR spectra for new compounds 63
6. Supplementary Reference 169

1. General information

All reactions were carried out under an atmosphere of dry argon using standard Schlenk technique or Ace pressure tube. Chemicals were purchased from *Sigma-Aldrich*, *TCI*, *Alfa Aesar*, *Fluorochem* or *ABCR*. Toluene, acetonitrile, heptane, 1,4-dioxane, DMF, NMP, DCE, THF were dried and degassed and stored in ©Aldrich Sure/Store flasks under argon. Other chemicals were used as received. Multiplets of NMR were assigned as s (singlet), d (doublet), t (triplet), dd (doublet of doublet), dt (doublet of triplet), td (triplet of doublet), m (multiplet), and br. s (broad singlet). NMR data were recorded on Bruker Avance 300 or Bruker ARX 400 spectrometers at room temperature. ^{1}H and ^{13}C NMR spectra were referenced to signals of deuterated solvents and residual protiated solvents, respectively. High resolution mass spectra (HRMS) were recorded on Agilent 6210. The data are given as mass units per charge (m/z).

2. Experimental section

2.1 Chemical list

Compound 1:

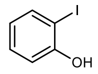
1a

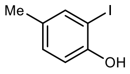
1b

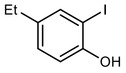
1c

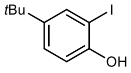
1d

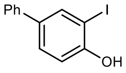
1e

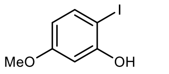
1f

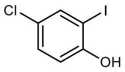
1g

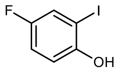
1h

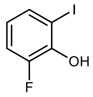
1i

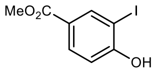
1j

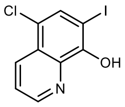
1k

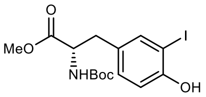
1l

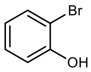
1m

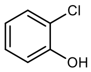
1n

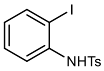
1o

Compound S1:

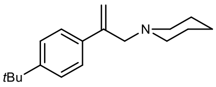
S1d

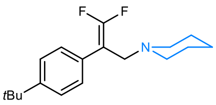
S1e

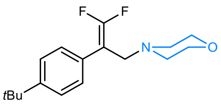
S1f

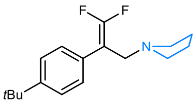
S1g

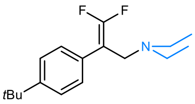
S1h

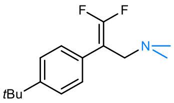
S1i

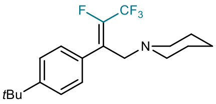
S1j

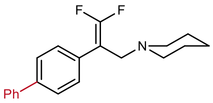
S1k

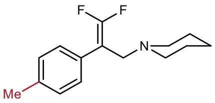
S1l

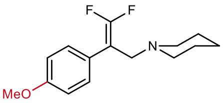
S1m

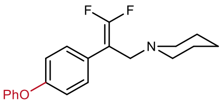
S1n

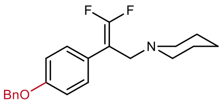
S1o

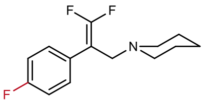
S1p

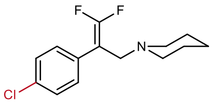
S1q

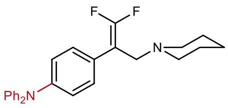
S1r

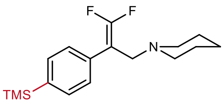
S1s

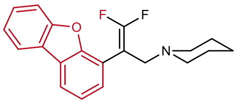
S1t

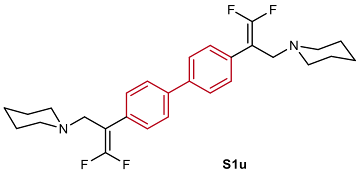
S1u

Compound 2:

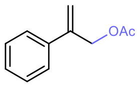

2a

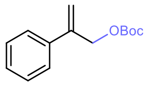

2b

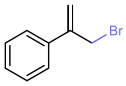

2c

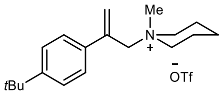

2d

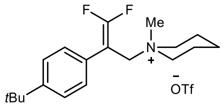

2e

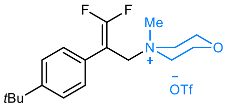

2f

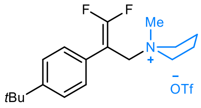

2g

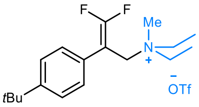

2h

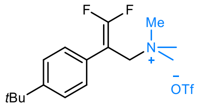

2i

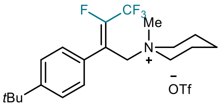

2j

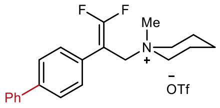

2k

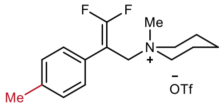

2l

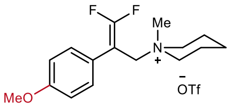

2m

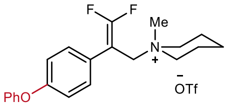

2n

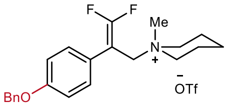

2o

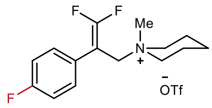

2p

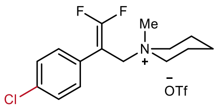

2q

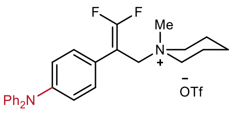

2r

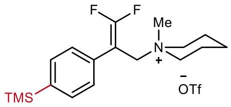

2s

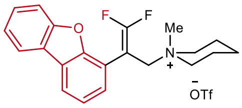

2t

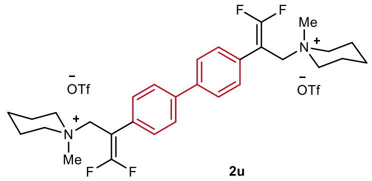

2u

Compound 5

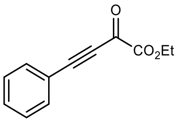

5a

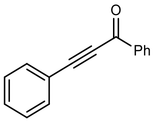

5b

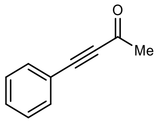

5c

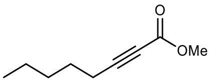

5d

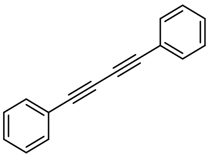

5e

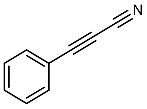

5f

2.2 General procedure for the synthesis of ammonium salts ***2:***

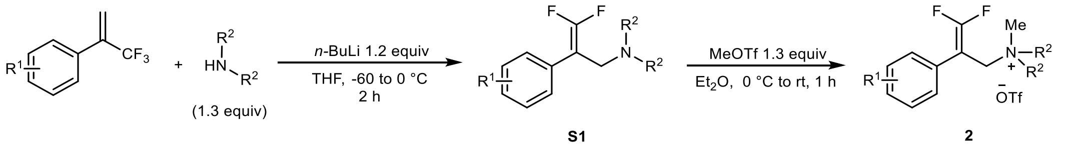

The allylic amine **S1** and ammonium salt **2** were prepared by methods described in the literature with some minor modifications.[1]

Procedure A: Under protection of argon, a 25 mL oven-dried flask equipped with a stirring bar was charged with piperidine (1.3 equiv) and anhydrous THF (0.2 M in the olefin). The flask was cooled to -60 °C using a dry ice/ethanol bath and after cooling for 10 min, a 1.6 M solution of *n*-BuLi (1.2 equiv) in hexanes was added dropwise to the flask. The solution was stirred at -60 °C for 1 h. A solution of fluoroalkylated olefine (1 equiv) was added to the flask dropwise over 5 min. The resulting mixture was stirred at -60 °C for 1 h, then warmed to 0 °C in an ice-water bath and stirred for 1 h before quenching with aqueous NH_4Cl (20 mL). The organic layer was extracted with ethyl acetate (×3), washed with brine, dried over Na_2SO_4, filtered, and concentrated under reduced pressure. The residue was purified by flash chromatography to afford the desired compounds **S1**.

Procedure B: To a Schlenk tube equipped with a magnetic stir bar were added amine **S1** (1 equiv) and dry Et_2O (0.2 M). The flask was cooled to 0 °C using an ice-water bath, MeOTf (1.3 equiv) was added dropwise to the flask over 5 min. The solution was stirred at 0 °C for 30-60 min and gradually became cloudy and white. The solution was filtrated and washed with dry Et_2O (3 × 10 mL). Drying under vacuum for 5 h to give the pure products **2**.

2.3 General procedure for the preparation of spiro-fused benzocyclobutanes ***4*** *and dihydronaphthalenes* ***6:***

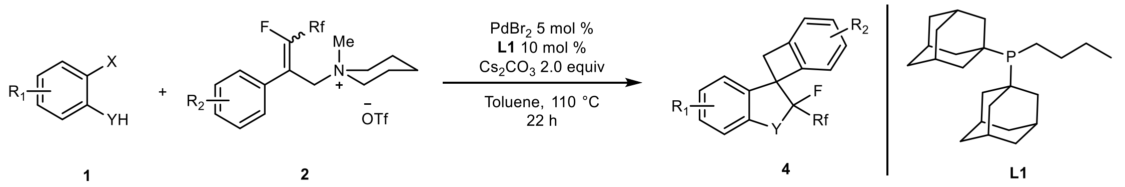

Procedure C: To a 25 mL oven-dried pressure tube equipped with a magnetic stir bar were added 2-halophenol or aniline **1** (0.2 mmol), ammonium salt **2** (0.2 mmol), Cs_2CO_3 (130 mg, 0.4 mmol), $PdBr_2$ (2.7 mg, 0.01 mmol), **L1** (7.2 mg, 0.02 mmol), and then degassed toluene (2.5

mL) was introduced under argon atmosphere. The sealed pressure tube was heated and stirred at 110 °C for 22 h. The reaction mixture was allowed to cool to room temperature, diluted with ethyl acetate (10 mL), and filtered through a short pad of celite eluting with ethyl acetate (3 × 10 mL). After evaporation, the residue was purified by chromatography on basic aluminum oxide (It is worthy to note that the 2-fluorinated product can only be separated without decomposition using basic aluminum oxide) to afford the desired product **4**.

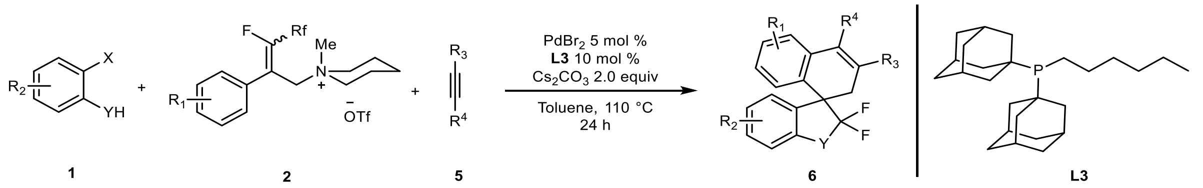

Procedure D: To a 25 mL oven-dried pressure tube equipped with a magnetic stir bar were added 2-halophenol or aniline **1** (0.2 mmol), ammonium salt **2** (0.2 mmol), alkyne **5** (0.4 mmol), Cs_2CO_3 (130 mg, 0.4 mmol), $PdBr_2$ (2.7 mg, 0.01 mmol), **L3** (7.7 mg, 0.02 mmol), and then degassed toluene (1 mL) was introduced under argon atmosphere. The sealed pressure tube was heated and stirred at 110 °C for 24 h. The reaction mixture was allowed to cool to room temperature, diluted with ethyl acetate (10 mL), and filtered through a short pad of celite eluting with ethyl acetate (3 × 10 mL). After evaporation, the residue was purified by chromatography on basic aluminum oxide (It is worthy to note that the 2-fluorinated product can only be separated without decomposition using basic aluminum oxide) to afford the desired product **6**.

2.4 Optimization of reaction conditions

Supplementary Table 1. Initiate results: Palladium-catalyzed cascade reaction of 1a and 2a

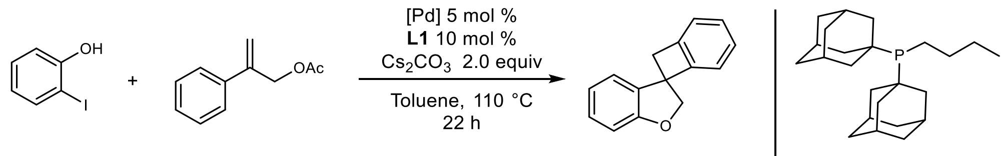

1a **2a** **4a** **L1**

Entry	Pd pre-catalyst	Ligand	4a (%)[a]
1	$Pd(OAc)_2$	**L1**	0
2	$Pd(P^tBu_3)_2$	/	0
3	$Pd(PPh_3)_4$	/	0
4	$Pd(dba)_2$	**L1**	0
5	$PdCl_2(cod)$	**L1**	0
6[b]	$PdBr_2$	**L1**	0

Reaction conditions: **1a** (0.2 mmol), **2a** (0.2 mmol), Cs_2CO_3 (0.4 mmol), [Pd] (0.01 mmol), **L1** (0.02 mmol), Toluene (1.5 mL), the reaction mixture was stirred at 110 °C under Argon atmosphere for 22 h. [a] the yield was determined by GC. [b] KO^tBu (0.2 mmol) was used.

Supplementary Table 2. Screening various living groups of 2

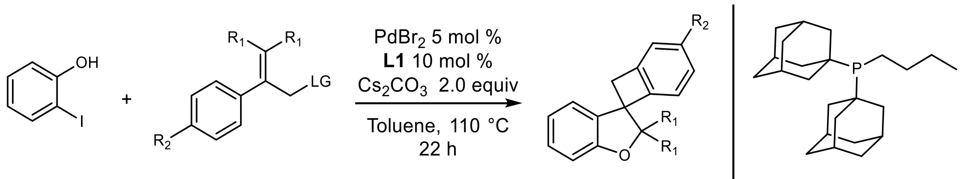

1a **2** **4** **L1**

Entry	R_1	R_2	LG	Yield 4
1	H	H	OAc	0[a]
2	H	H	OBoc	0[a]
3	H	*t*Bu	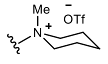	87[b]
4	F	*t*Bu	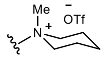	84[c]
5	F	*t*Bu	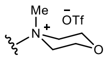	62[c]
6	F	*t*Bu	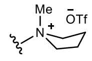	69[c]
7	F	*t*Bu	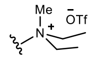	74[c]
8	F	*t*Bu	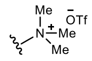	75[c]

Reaction conditions: **1a** (0.2 mmol), **2** (0.2 mmol), Cs_2CO_3 (0.4 mmol), $PdBr_2$ (0.01 mmol), **L1** (0.02 mmol), Toluene (2.5 mL), the reaction mixture was stirred at 110 °C under Argon atmosphere for 22 h. [a] The yield was determined by GC. [b] Isolated yield of **4** was given. [c] The NMR yield of **4c** was determined by ^{19}F-NMR using 1,4-difluorobenzene as internal standard.

Supplementary Table 3. The effect of the palladium source

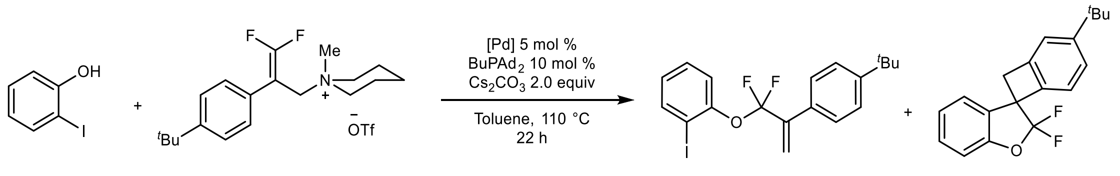

Entry	[Pd]	3c (%)	4c (%)
1	$Pd(OAc)_2$	trace	56 (49) [b]
2[a]	$Pd(P^tBu_3)_2$	55	21
3	$Pd(TFA)_2$	trace	73
4	$Pd(dba)_2$	53	20
5	$Pd(PPh_3)_4$	trace	71
6	$PdCl_2$	0	76
7	$PdBr_2$	0	77
8	PdI_2	0	73
9	$Pd(acac)_2$	91	trace
10	$Pd(cod)Cl_2$	0	75
11	$[PdCl(C_3H_5)]_2$	0	73

Reaction conditions: **1a** (0.315 mmol), **2e** (0.3 mmol), [Pd] (0.015 mmol), $BuPAd_2$ (0.003 mmol), Cs_2CO_3 (0.6 mmol) and Toluene (2 mL) were stirred under argon atmosphere at 110 °C for 22 h, the yield was determined by ^{19}F-NMR using 1,4-difluorobenzene as internal standard.
[a] $BuPAd_2$ was not used as ligand. [b] Isolated yield is given in the parentheses.

Supplementary Table 4. The effect of phosphine ligands

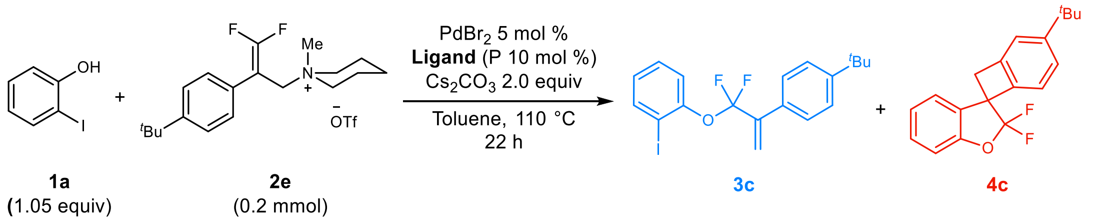

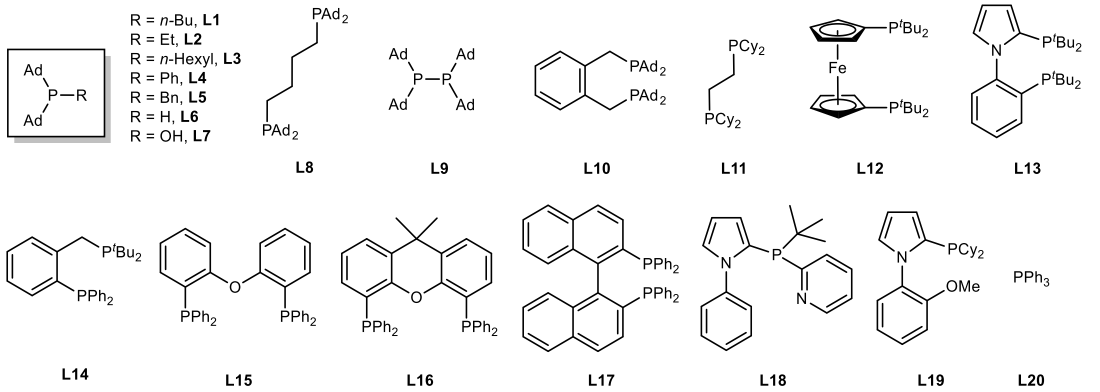

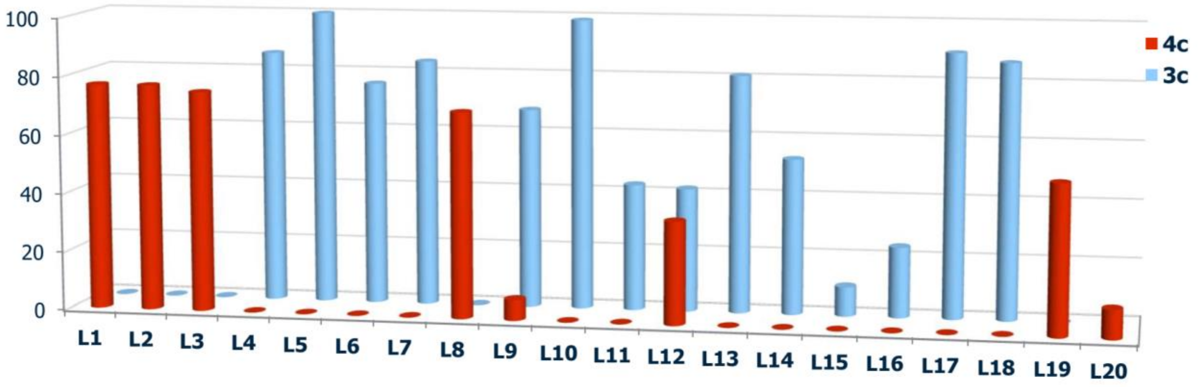

Reaction conditions: **1a** (0.21 mmol), **2e** (0.2 mmol), $PdBr_2$ (0.01 mmol), Ligand (bidentate ligand 0.01 mmol or monodentate ligand 0.02 mmol), Base (0.4 mmol) and Toluene (1.5 mL) were stirred under argon atmosphere at 110 °C for 22 h, the yield was determined by ^{19}F-NMR using 1,4-difluorobenzene as internal standard.

Supplementary Table 5. The effect of base

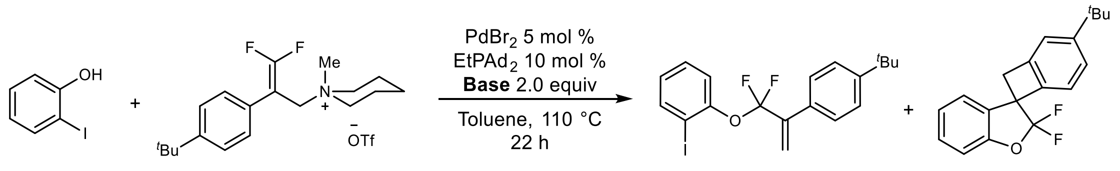

Entry	Base	3c (%)	4c (%)
1	Cs_2CO_3	0	77
2	K_2CO_3	0	33
3	K_3PO_4	17	48
4	KOH	42	15
5	KO^{t}Bu	45	3
6	CsOAc	50	0
7	CsTFA	31	33
8	CsBr	32	0
9	CsCl	40	0
10	NaOEt	67	0
11	NEt_3	42	0
12	DABCO	76	0

Reaction conditions: **1a** (0.21 mmol), **2e** (0.2 mmol), $PdBr_2$ (0.01 mmol), $EtPAd_2$ (0.02 mmol), Base (0.4 mmol) and Toluene (1.5 mL) were stirred under argon atmosphere at 110 °C for 22 h, the yield was determined by ^{19}F-NMR using 1,4-difluorobenzene as internal standard.

Supplementary Table 6. The effect of solvent

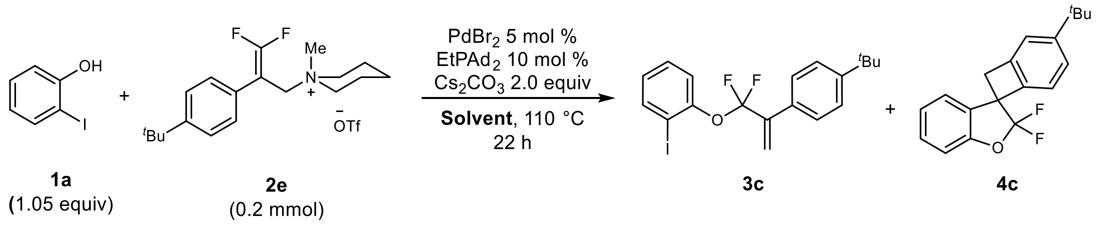

Entry	Solvent	3c (%)	4c (%)
1	Toluene	0	77
2	*o*-Xylene	0	34
3	Mesitylene	0	60
4	Benzene	0	74
5	1,4-Dioxane	0	40
6	CH_3CN	45	12
7	DMF	0	25
8	NMP	0	40
9	DCE	40	18

Reaction conditions: **1a** (0.21 mmol), **2e** (0.2 mmol), $PdBr_2$ (0.01 mmol), $EtPAd_2$ (0.02 mmol), Cs_2CO_3 (0.4 mmol) and Solvent (1.5 mL) were stirred under argon atmosphere at 110 °C for 22 h, the yield was determined by ^{19}F-NMR using 1,4-difluorobenzene as internal standard.

Supplementary Table 7. The effect of [Pd]/Ligand ratio

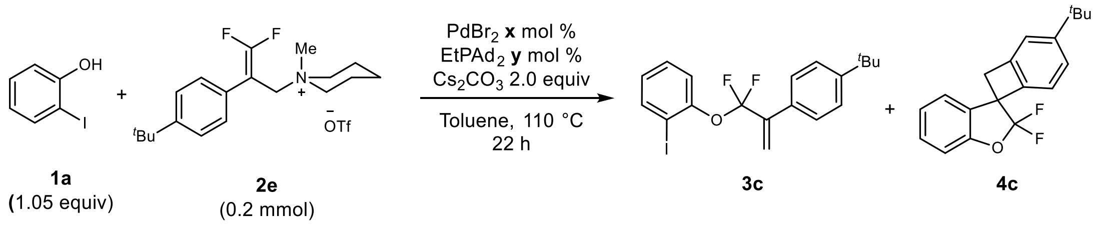

Entry	$PdBr_2$	$EtPAd_2$ (mol %)	3c (%)	4c (%)
1	5	10	0	77
2	5	15	0	34
3	5	20	0	60
4	10	20	0	77

Reaction conditions: **1a** (0.21 mmol), **2e** (0.2 mmol), $PdBr_2$ (*x* mol %), $EtPAd_2$ (*y* mol %), Cs_2CO_3 (0.4 mmol) and Toluene (1.5 mL) were stirred under argon atmosphere at 110 °C for 22 h, the yield was determined by ^{19}F-NMR using 1,4-difluorobenzene as internal standard.

Supplementary Table 8. The effect of starting material's ratio

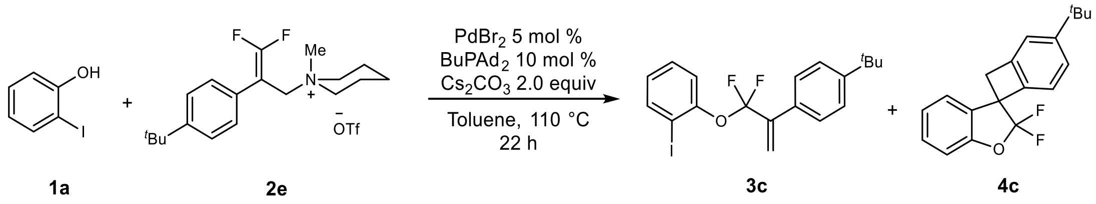

Entry	Ratio of 1a/2e	3c (%)	4c (%)
1	1.05/1	0	77
2	1.2/1	0	54
3	1.5/1	90	0
4	1/1	0	78
5	1/1.1	0	78
6	1/1.3	0	37
7	1/1.5	0	32

Reaction conditions: **1a** (x mmol), **2e** (y mmol), $PdBr_2$ (0.01 mmol), $BuPAd_2$ (0.02 mmol), Cs_2CO_3 (0.4 mmol) and Toluene (1.5 mL) were stirred under argon atmosphere at 110 °C for 22 h, the yield was determined by ^{19}F-NMR using 1,4-difluorobenzene as internal standard.

Supplementary Table 9. The effect of catalyst loading and concentration

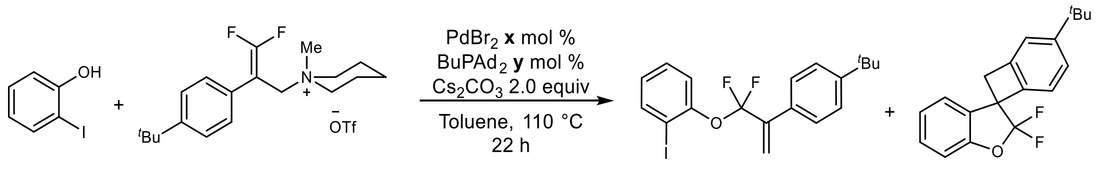

Entry	x (mol %)	y (mol %)	Conc.	3c (%)	4c (%)
1	5	10	0.133 M	0	78
2	2	4	0.133 M	0	79
3	1	2	0.133 M	0	76
4	5	10	0.2 M	0	78
5	5	10	0.08 M	0	84(83)[b]
6	2	4	0.08 M	0	79
7[a]	1	2	0.08 M	0	79
8[a]	0.5	1	0.08 M	75	12

Reaction conditions: **1a** (0.2 mmol), **2e** (0.2 mmol), PdBr$_2$ (*x* mol %), BuPAd$_2$ (*y* mol %), Cs$_2$CO$_3$ (0.4 mmol) and Toluene (*z* mL) were stirred under argon atmosphere at 110 °C for 22 h, the yield was determined by ^{19}F-NMR using 1,4-difluorobenzene as internal standard. [a] 0.4 mmol scale. [b] Isolated yield.

Supplementary Table 10. Evaluation of the optimized conditions: Palladium-catalyzed cascade reaction of 1a, 2e and 5a.

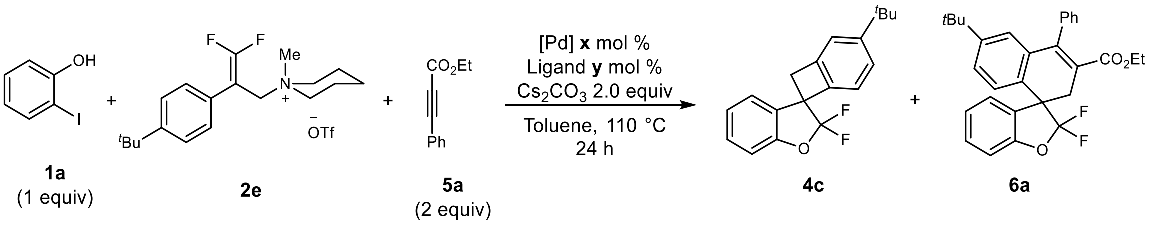

Entry	[Pd] (x mol %)	Ligand (y mol %)	Conc.	4c (%)	6a (%)
1[a]	$PdBr_2$ (5)	**L1** (10)	0.08 M	14	73
2[a]	$PdBr_2$ (10)	**L1** (20)	0.08 M	18	74
3	$PdBr_2$ (5)	**L1** (10)	0.08 M	10	82
4[b]	$PdBr_2$ (5)	**L1** (10)	0.08M	20	73
5[c]	$PdBr_2$ (5)	**L1** (10)	0.08 M	11	80
6	$PdBr_2$ (2)	**L1** (4)	0.08 M	11	81
7	$PdBr_2$ (5)	**L1** (10)	0.2 M	6	87
8	$PdCl_2$ (5)	**L1** (10)	0.2 M	6	88
9	$PdBr_2$ (5)	**L2** (10)	0.2 M	4	89
10	$PdBr_2$ (5)	**L3** (10)	0.2 M	0	92
11	$PdBr_2$ (5)	**L8** (5)	0.2 M	6	85

Reaction conditions: **1a** (0.2 mmol), **2e** (0.2 mmol), **5a** (0.4 mmol), [Pd] (x mol%), Ligand (y mol%), Cs_2CO_3 (0.4 mmol) and Toluene were stirred under argon atmosphere at 110 °C for 24 h, the yield was determined by ^{19}F-NMR using 1,4-difluorobenzene as internal standard. [a] 1.5 equivalent of **5a** was used. [b] The reaction mixture was heated at 120 °C for 24 h. [c] 3.0 equivalent of Cs_2CO_3 was used.

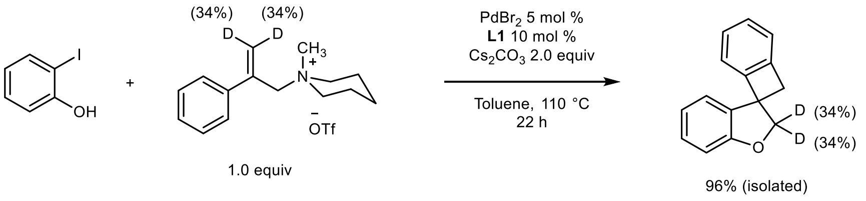

Supplementary Fig. 1. Control experiment. The allyl ammonium salt substituted with two deuterium at 3,3'-position was examined in the reaction.

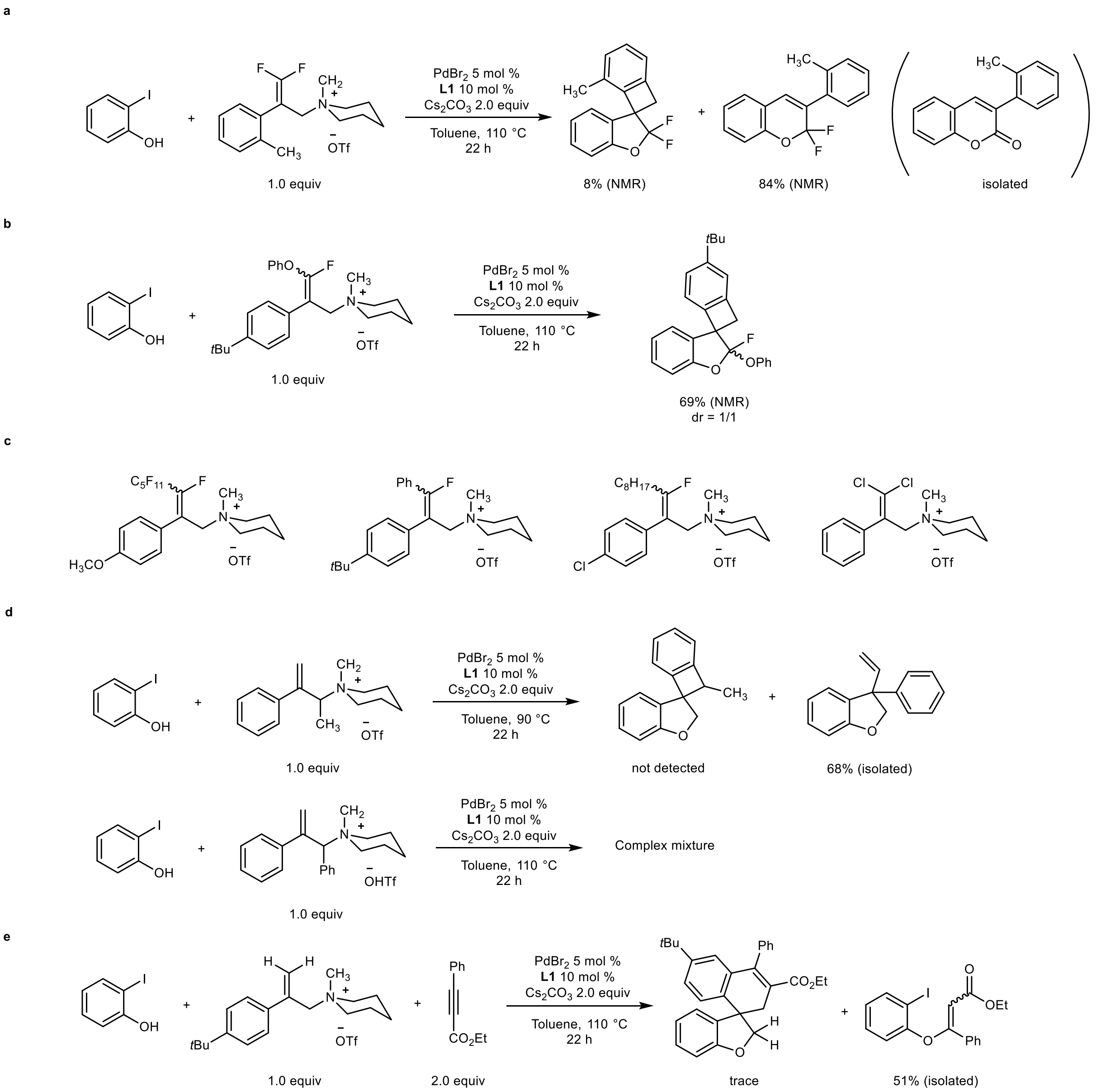

Supplementary Fig. 2. Unsuccessful examples. **a**, *Ortho* methyl-substituted ammonium salt provided low yield of the desired 4-membered spiro-heterocycle, *3-(o-tolyl)-2H-chromen-2-one* was isolated as the major product after chromatography. **b**, The 3,3'-F,-OPh-substituted ammonium salt converted to the corresponding 5,4-spirocyles in 69% yield (NMR) with 1/1 *dr*; however, the purification failed using either neutral silica gel or basic aluminum oxide. **c**, The ammonium salts containing -C_5F_{11}, -Ph, -C_8H_{17}, -Cl at 3,3'-position provide a complex mixture. **d**, the ammonium salt containing a methyl (providing beta-H) at C1 position did not afford the desired spiro-heterocycle, the beta-H elimination took place giving the dihydrobenzofuran with vinyl and aryl substitutes. The phenyl-substituted ammonium salt (not providing beta-H) at C1 position in substrate **2** gave a product mixture under standard conditions. **e**, The allyl ammonium salt **2d** (with two protons) in the three-component cascade reaction gave only small amounts of the 6,5-spiroheterocycle, the conjugate addition product of 2-iodophenol and alkyne was isolated as the major product in 51% yield.

3. Characterization of new compounds

Compound S1

S1d

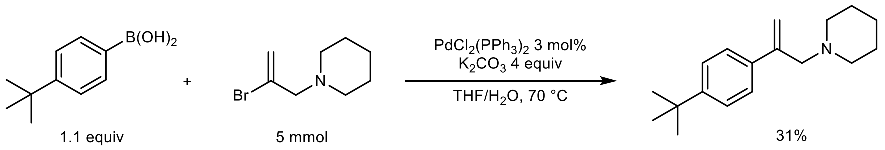

To a Schlenk tube equipped with a magnetic stir bar were added 1-(2-bromoallyl)piperidine (1.1 g, 5.0 mmol), 4-*tert*-butylphenylboronic acid (0.98 g, 5.5 mmol, 1.1 equiv), $PdCl_2(PPh_3)_2$ (105 mg, 0.15 mmol, 3 mol%), K_2CO_3 (2.76 mg, 20 mmol, 4.0 equiv), and THF/H_2O (20 mL). The resulting solution was stirred at 60°C for 24 h. After the reaction mixture was cooled to room temperature, the reaction mixture was quenched with saturated aqueous NH_4Cl, and extracted with ethyl acetate (3 × 20 mL). The combined organic layers were dried with Na_2SO_4 and the solvent was removed under reduced pressure. The resultant crude product material was purified by flash chromatography on silica gel (Pentane/Ethyl acetate gradient from 98/2 to 90/10), affording compound **S1d** (0.4 g, 31%) as a colorless oil.

^{1}H NMR (300 MHz, $CDCl_3$) δ 7.54 – 7.46 (m, 2H), 7.38 – 7.31 (m, 2H), 5.46 (d, *J* = 2.0 Hz, 1H), 5.24 – 5.18 (m, 1H), 3.28 (s, 2H), 2.51 – 2.28 (m, 4H), 1.63 – 1.51 (m, 4H), 1.48 – 1.38 (m, 2H), 1.33 (s, 9H).

^{13}C NMR (75 MHz, $CDCl_3$) δ 150.4, 144.1, 137.1, 126.1, 125.2, 114.3, 63.9, 54.8, 34.6, 31.5, 26.2, 24.7.

S1f

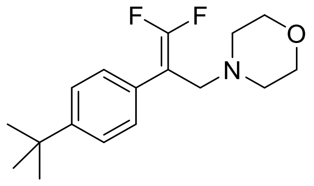

Chemical Formula: $C_{17}H_{23}F_2NO$
Exact Mass: 295.1748

This compound was obtained following the general procedure A. Starting from 1-(*tert*-butyl)-4-(3,3,3-trifluoroprop-1-en-2-yl)benzene (0.68 g, 3.0 mmol), morpholine (0.34 g, 3.9 mmol, 1.3 equiv), *n*-butyl lithium (1.6 M in hexane, 3.6 mmol, 1.2 equiv) and THF (20 mL). Purification on silica gel (Pentane/Ethyl acetate gradient from 98/2 to 90/10) afforded **S1f** (0.36 g, 41%) as a white solid.

^{1}H NMR (300 MHz, $CDCl_3$) δ 7.49 – 7.41 (m, 2H), 7.41 – 7.33 (m, 2H), 3.73 – 3.62 (m, 4H), 3.28 (dd, *J* = 3.2, 1.7 Hz, 2H), 2.55 – 2.40 (m, 4H), 1.33 (s, 9H).

^{13}C NMR (75 MHz, $CDCl_3$) δ 155.5, 150.4, 131.0 – 130.7 (m), 128.1 – 127.8 (m), 125.4, 88.9 (dd, *J* = 18.8, 12.0 Hz), 67.1, 56.0 (d, *J* = 4.3 Hz), 53.2, 34.7, 31.4.

^{19}F NMR (282 MHz, $CDCl_3$) δ -87.9 (d, *J* = 35.8 Hz, 1F), -88.4 (d, *J* = 35.8 Hz, 1F).

S1g

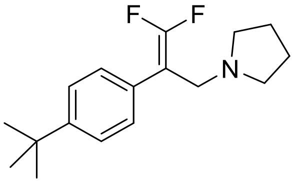

Chemical Formula: $C_{17}H_{23}F_2N$
Exact Mass: 279.1799

This compound was obtained following the general procedure A. Starting from 1-(*tert*-butyl)-4-(3,3,3-trifluoroprop-1-en-2-yl)benzene (0.68 g, 3.0 mmol), pyrrolidine (0.25 g, 3.6 mmol, 1.2 equiv), *n*-butyl lithium (1.6 M in hexane, 3.6 mmol, 1.2 equiv) and THF (20 mL). Purification on silica gel (Pentane/Ethyl acetate gradient from 98/2 to 90/10) afforded **S1g** (0.54 g, 65%) as a colorless oil.

^{1}H NMR (300 MHz, $CDCl_3$) δ 7.47 – 7.32 (m, 4H), 3.42 (dd, *J* = 3.2, 1.8 Hz, 2H), 2.61 – 2.36 (m, 4H), 1.83 – 1.67 (m, 4H), 1.32 (s, 9H).

^{13}C NMR (75 MHz, $CDCl_3$) δ 150.3, 130.9 – 130.7 (m), 127.9 (t, *J* = 3.4 Hz), 125.5, 53.9, 53.1 – 52.7 (m), 34.7, 31.4, 23.7.

^{19}F NMR (282 MHz, $CDCl_3$) δ -87.51 – -90.7 (m, 2F).

S1h

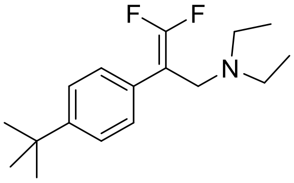

Chemical Formula: $C_{17}H_{25}F_2N$
Exact Mass: 281.1955

This compound was obtained following the general procedure A. Starting from 1-(tert-butyl)-4-(3,3,3-trifluoroprop-1-en-2-yl)benzene (0.68 g, 3.0 mmol), diethylamine (0.285 g, 3.9 mmol, 1.3 equiv), *n*-butyl lithium (1.6 M in hexane, 3.6 mmol, 1.2 equiv) and THF (20 mL). Purification on silica gel (Pentane/Ethyl acetate gradient from 98/2 to 90/10) afforded **S1h** (0.42 g, 50%) as a colorless oil.

^{1}H NMR (300 MHz, $CDCl_3$) δ 7.47 – 7.37 (m, 3H), 7.41 – 7.31 (m, 3H), 3.38 (dd, *J* = 3.0, 1.7 Hz, 2H), 2.54 (q, *J* = 7.1 Hz, 4H), 1.33 (s, 9H), 1.00 (t, *J* = 7.1 Hz, 6H).

^{13}C NMR (75 MHz, $CDCl_3$) δ 155.1 (dd, *J* = 291.0, 286.5 Hz), 150.1, 130.8 (t, *J* = 3.8 Hz), 128.1 (t, *J* = 3.4 Hz), 125.3, 90.5 (dd, *J* = 18.0, 11.3 Hz), 50.1 (d, *J* = 4.1 Hz), 46.5, 34.6, 31.4, 11.5.

^{19}F NMR (282 MHz, $CDCl_3$) δ -88.9 (d, *J* = 39.2 Hz, 1F), -90.0 (d, *J* = 39.0 Hz, 1F).

S1j

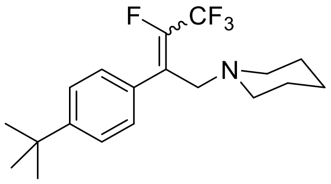

Chemical Formula: $C_{19}H_{25}F_4N$
Exact Mass: 343.1923

This compound was obtained following the general procedure A. Starting from 1-(*tert*-butyl)-4-(3,3,4,4,4-pentafluorobut-1-en-2-yl)benzene (1.0 g, 3.6mmol), piperidine (0.4 g, 4.7 mmol, 1.3 equiv), *n*-butyl lithium (1.6 M in hexane, 4.32 mmol, 1.2 equiv) and THF (20 mL). Purification on silica gel (Pentane/Ethyl acetate gradient from 98/2 to 90/10) afforded **S1j** (0.93 g, 75%, dr = 3.5/1) as a colorless oil.

^{1}H NMR (300 MHz, $CDCl_3$) δ 7.41 – 7.15 (m, 4H), 3.39 – 3.34 (m, 2H, minor), 3.34 – 3.26 (m, 2H, major), 2.50 – 2.27 (m, 4H), 1.59 – 1.46 (m, 4H), 1.46 – 1.36 (m, 2H), 1.34 (s, 9H, minor), 1.33 (s, 9H, major).

^{13}C NMR (75 MHz, $CDCl_3$) δ 151.5 (minor), 151.3 (major), 131.7 (minor), 131.3 (d, *J* = 5.0 Hz), 128.5 – 128.3 (m, minor), 128.3 – 128.1 (m, major), 125.2 (minor), 124.9 (major), 58.4 (d, *J* = 3.5 Hz), 54.4 (major), 54.2 (minor), 34.7, 31.5 (major), 31.4 (minor), 26.1, 24.4 (minor), 24.3 (major).

^{19}F NMR (282 MHz, $CDCl_3$) δ -63.6 (d, *J* = 8.0 Hz, 3F, minor), -64.7 (d, *J* = 9.6 Hz, 3F, major), -126.2 (s, 1F, minor), -128.9 (s, 1F, major).

S1o

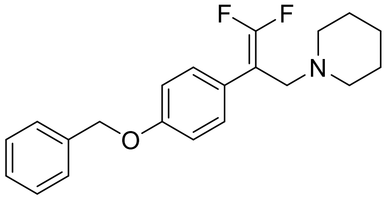

Chemical Formula: $C_{21}H_{23}F_2NO$
Exact Mass: 343.1748

This compound was obtained following the general procedure A. Starting from 1-(benzyloxy)-

4-(3,3,3-trifluoroprop-1-en-2-yl)benzene (0.62 g, 2.2 mmol), piperidine (0.25 g, 2.9 mmol, 1.3 equiv), *n*-butyl lithium (1.6 M in hexane, 2.6 mmol, 1.2 equiv) and THF (15 mL). Purification on silica gel (Pentane/Ethyl acetate gradient from 98/2 to 90/10) afforded **S1o** (0.45 g, 60%) as a colorless oil.

^{1}H NMR (300 MHz, $CDCl_3$) δ 7.52 – 7.30 (m, 7H), 7.01 – 6.92 (m, 2H), 5.08 (s, 2H), 3.24 (dd, *J* = 3.1, 1.8 Hz, 2H), 2.40 (t, *J* = 5.2 Hz, 4H), 1.62 – 1.48 (m, 4H), 1.47 – 1.35 (m, 2H).

^{13}C NMR (75 MHz, $CDCl_3$) δ 158.0, 155.1 (dd, *J* = 290.3, 286.5 Hz), 137.1, 129.7 (t, *J* = 3.4 Hz), 128.7, 128.1, 127.6, 126.8 (t, *J* = 3.4 Hz), 114.7, 89.3 (dd, *J* = 18.7, 12.0 Hz), 70.1, 56.4 (d, *J* = 3.8 Hz), 54.1, 26.1, 24.5.

^{19}F NMR (282 MHz, $CDCl_3$) δ -88.5 (d, *J* = 38.9 Hz, 1F), -89.9 (d, *J* = 38.9 Hz, 1F).

S1s

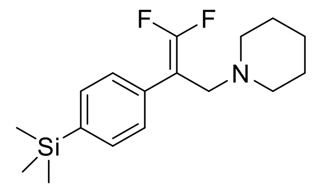

Chemical Formula: $C_{17}H_{25}F_2NSi$
Exact Mass: 309.1724

This compound was obtained following the general procedure A. Starting from trimethyl(4-(3,3,3-trifluoroprop-1-en-2-yl)phenyl)silane (0.65 g, 2.66 mmol), piperidine (0.35 mL, 3.5 mmol, 1.3 equiv), *n*-butyl lithium (1.6 M in hexane, 3.2 mmol, 1.2 equiv) and THF (15 mL). Purification on silica gel (Pentane/Ethyl acetate gradient from 98/2 to 90/10) afforded **S1s** (0.5 g, 61%) as a colorless oil.

^{1}H NMR (300 MHz, $CDCl_3$) δ 7.54 – 7.46 (m, 4H), 3.27 (t, *J* = 2.4 Hz, 2H), 2.40 (t, *J* = 5.2 Hz, 4H), 1.64 – 1.48 (m, 4H), 1.48 – 1.33 (m, 2H), 0.28 (s, 9H).

^{13}C NMR (75 MHz, $CDCl_3$) δ 159.4 (t, *J* = 290.0 Hz), 139.4, 134.7, 133.4, 127.7 (t, *J* = 3.4 Hz), 89.7 (dd, *J* = 16.3, 13.7 Hz), 56.2, 54.1, 26.1, 24.5, -1.0.

^{19}F NMR (282 MHz, $CDCl_3$) δ -88.3 (s, 2F).

S1t

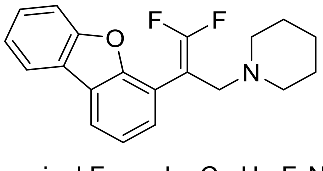

Chemical Formula: $C_{20}H_{19}F_2NO$
Exact Mass: 327.1435

This compound was obtained following the general procedure A. Starting from 4-(3,3,3-

trifluoroprop-1-en-2-yl)dibenzo[*b*,*d*]furan (0.67 g, 2.5 mmol), piperidine (0.25 mL, 2.55 mmol, 1.05 equiv), *n*-butyl lithium (1.6 M in hexane, 2.5 mmol, 1.0 equiv) and THF (20 mL). Purification on silica gel (Pentane/Ethyl acetate gradient from 98/2 to 90/10) afforded **S1t** (0.8 g, 98%) as a colorless oil.

^{1}H NMR (300 MHz, $CDCl_3$) δ 7.96 (ddd, *J* = 7.6, 1.4, 0.7 Hz, 1H), 7.91 (dd, *J* = 7.7, 1.3 Hz, 1H), 7.67 – 7.55 (m, 1H), 7.55 – 7.41 (m, 2H), 7.41 – 7.29 (m, 2H), 3.55 (dd, *J* = 2.9, 1.7 Hz, 2H), 2.43 (t, *J* = 5.3 Hz, 4H), 1.58 – 1.42 (m, 4H), 1.42 – 1.29 (m, 2H).

^{13}C NMR (75 MHz, $CDCl_3$) δ 158.9 (t, *J* = 278.3 Hz), 156.2, 154.5 (d, *J* = 94.4 Hz), 128.1 – 127.9 (m), 127.4, 124.6, 124.4, 122.9, 122.8, 120.8, 120.1, 118.7 (dd, *J* = 5.3, 2.3 Hz), 111.9, 56.0 (d, *J* = 3.8 Hz), 53.9, 26.0, 24.3.

^{19}F NMR (282 MHz, $CDCl_3$) δ -84.7 (d, *J* = 31.6 Hz, 1F), -88.8 (d, *J* = 31.6 Hz, 1F).

S1u

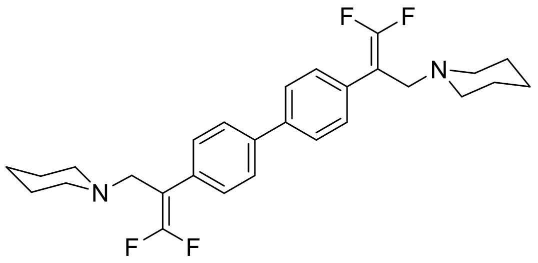

Chemical Formula: $C_{28}H_{32}F_4N_2$
Exact Mass: 472.2502

This compound was obtained following the general procedure A. Starting from 4,4'-bis(3,3,3-trifluoroprop-1-en-2-yl)-1,1'-biphenyl (0.38 g, 1.1 mmol), piperidine (0.29 mL, 2.8 mmol, 2.5 equiv), *n*-butyl lithium (1.6 M in hexane, 2.6 mmol, 2.4 equiv) and THF (15 mL). Purification on silica gel (Pentane/Ethyl acetate gradient from 98/2 to 90/10) afforded **S1u** (0.265 g, 51%) as a white solid.

^{1}H NMR (300 MHz, $CDCl_3$) δ 7.63 – 7.55 (m, 4H), 3.37 – 3.25 (m, 2H), 2.43 (t, *J* = 5.3 Hz, 4H), 1.63 – 1.50 (m, 4H), 1.48 – 1.36 (m, 2H).

^{13}C NMR (75 MHz, $CDCl_3$) δ 155.4 (dd, *J* = 291.0, 288.8 Hz), 139.5, 133.3, 128.9 (t, *J* = 3.5 Hz), 126.9, 89.4 (dd, *J* = 16.6, 14.1 Hz), 56.2, 54.1, 26.0, 24.4.

^{19}F NMR (282 MHz, $CDCl_3$) δ -88.0 (s, 2F).

Compound 1

1d

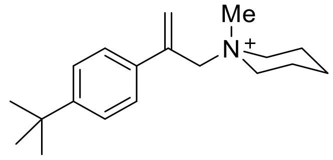

$^{-}$OTf

Chemical Formula: $CF_3O_3S^-$
Exact Mass: 148.9526

Chemical Formula: $C_{19}H_{30}N^+$
Exact Mass: 272.2373

This compound was obtained following the general procedure B. Starting from amine **S1d** (0.35 g, 1.36 mmol), methyl trifluoromethanesulfonate (0.29 g, 1.77 mmol, 1.3 equiv), and diethyl ether (10 mL). Filtration to afford **1d** (0.57 g, 99%) as a white solid.

^{1}H NMR (300 MHz, Acetone-d_6) δ 7.62 – 7.54 (m, 2H), 7.51 – 7.43 (m, 2H), 5.94 – 5.84 (m, 2H), 4.73 (s, 2H), 3.61 – 3.39 (m, 4H), 3.07 (s, 3H), 1.99 – 1.82 (m, 4H), 1.78 – 1.56 (m, 2H), 1.31 (s, 9H).

^{13}C NMR (75 MHz, Acetone) δ 152.5, 138.5, 137.9, 128.8, 127.2, 126.7, 67.7, 62.2, 48.2, 35.1, 31.5, 21.6, 20.7.

^{19}F NMR (282 MHz, Acetone-d_6) δ -78.9 (s, 3F).

HRMS (ESI): calculated for $C_{19}H_{30}N$ [M-OTf]$^+$: 272.2383, found: 272.2376.

1f

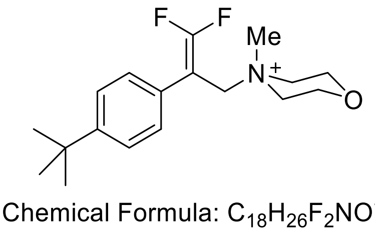

$^{-}$OTf

Chemical Formula: $CF_3O_3S^-$
Exact Mass: 148.9526

Chemical Formula: $C_{18}H_{26}F_2NO^+$
Exact Mass: 310.1977

This compound was obtained following the general procedure B. Starting from amine **S1f** (0.34 g, 1.15 mmol), methyl trifluoromethanesulfonate (0.25 g, 1.5 mmol, 1.3 equiv), and diethyl ether (10 mL). Filtration to afford **1f** (0.5 g, 95%) as a white solid.

^{1}H NMR (300 MHz, Acetone-d_6) δ 7.64 – 7.56 (m, 2H), 7.56 – 7.49 (m, 2H), 4.91 – 4.79 (m, 2H), 4.13 – 3.92 (m, 4H), 3.73 – 3.52 (m, 4H), 3.30 (s, 3H), 1.32 (s, 9H).

^{13}C NMR (75 MHz, Acetone-d_6) δ 160.39 (t, J = 297.4 Hz), 152.6, 129.4 (t, J = 3.0 Hz), 129.2 (t, J = 2.3 Hz), 126.9, 122.1 (d, J = 321.3 Hz), 85.7 – 85.0 (m), 64.0, 61.1, 60.6, 47.4, 35.2, 31.4.

^{19}F NMR (282 MHz, Acetone-d_6) δ -78.0 (s, 3F), -80.0 (d, J = 15.7 Hz, 1F), -81.8 (d, J = 15.9 Hz, 1F).

HRMS (ESI): calculated for $C_{18}H_{26}F_2NO$ [M-OTf]$^+$: 310.1987, found: 310.1981.

1g

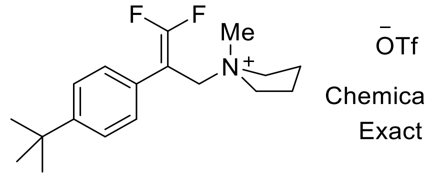

Chemical Formula: $CF_3O_3S^-$
Exact Mass: 148.9526

Chemical Formula: $C_{18}H_{26}F_2N^+$
Exact Mass: 294.2028

This compound was obtained following the general procedure B. Starting from amine **S1g** (0.50 g, 1.8 mmol), methyl trifluoromethanesulfonate (0.38 g, 2.34 mmol, 1.3 equiv), and diethyl ether (10 mL). Filtration to afford **1g** (0.75 g, 94%) as a white solid.

^{1}H NMR (300 MHz, Acetone-d_6) δ 7.68 – 7.45 (m, 4H), 4.79 – 4.68 (m, 2H), 3.77 – 3.52 (m, 4H), 3.25 – 3.10 (m, 3H), 2.34 – 2.11 (m, 4H), 1.33 (s, 9H).

^{13}C NMR (75 MHz, Acetone-d_6) 152.8 – 152.6 (m), 129.3, 129.2 – 129.1 (m), 126.9, 86.9 (dd, *J* = 20.6, 17.6 Hz), 65.0, 61.9 – 61.3 (m), 48.9, 35.2, 31.5, 21.9.

^{19}F NMR (282 MHz, Acetone-d_6) δ -78.9 (s, 3F), -82.1 (d, *J* = 17.4 Hz, 1F), -83.5 (d, *J* = 19.0 Hz, 1F).

HRMS (ESI): calculated for $C_{18}H_{26}F_2N$ [M-OTf]$^+$: 294.2038, found: 294.2035.

1h

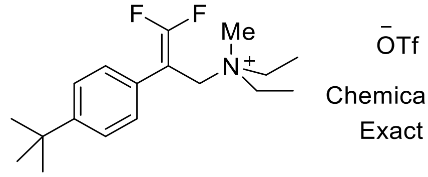

Chemical Formula: $CF_3O_3S^-$
Exact Mass: 148.9526

Chemical Formula: $C_{18}H_{28}F_2N^+$
Exact Mass: 296.2184

This compound was obtained following the general procedure B. Starting from amine **S1h** (0.40 g, 1.42 mmol), methyl trifluoromethanesulfonate (0.30 g, 1.85 mmol, 1.3 equiv), and diethyl ether (10 mL). Filtration to afford **1h** (0.57 g, 90%) as a white solid.

^{1}H NMR (300 MHz, Acetone-d_6) δ 7.64 – 7.56 (m, 2H), 7.56 – 7.44 (m, 2H), 4.70 – 4.50 (m, 2H), 3.58 – 3.40 (m, 4H), 3.06 (s, 3H), 1.32 (s, 9H), 1.31 – 1.22 (m, 6H).

^{13}C NMR (75 MHz, Acetone-d_6) δ 158.3 (t, *J* = 293.3 Hz), 152.6, 129.4, 126.8, 86.9 – 84.8 (m), 60.2, 57.1, 48.0, 35.2, 31.5, 8.1.

^{19}F NMR (282 MHz, Acetone-d_6) δ -78.9 (s, 3F), -80.9 (d, *J* = 17.7 Hz, 1F), -82.1 (d, *J* = 17.4 Hz, 1F).

HRMS (ESI): calculated for $C_{18}H_{28}F_2N$ [M-OTf]$^+$: 296.2195, found: 296.2189.

1i

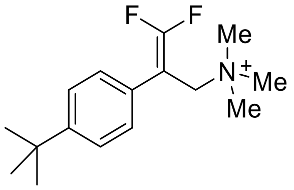

$^{-}$OTf

Chemical Formula: $CF_3O_3S^-$
Exact Mass: 148.9526

Chemical Formula: $C_{16}H_{24}F_2N$
Exact Mass: 268.1877

This compound was obtained following the general procedure B. Starting from amine **S1i** (0.35 g, 1.38 mmol), methyl trifluoromethanesulfonate (0.3 g, 1.8 mmol, 1.3 equiv), and diethyl ether (5 mL). Filtration to afford **1i** (0.53 g, 92%) as a white solid.

^{1}H NMR (300 MHz, Acetone-*d*$_6$) δ 7.65 – 7.56 (m, 2H), 7.55 – 7.46 (m, 2H), 4.78 – 4.62 (m, 2H), 3.26 (s, 9H), 1.31 (s, 9H).

^{13}C NMR (75 MHz, Acetone-*d*$_6$) δ 158.3 (t, *J* = 296.3 Hz), 152.4, 129.3 (t, *J* = 3.0 Hz), 129.2 (t, *J* = 2.6 Hz), 126.8, 122.2 (d, *J* = 321.5 Hz), 86.3 (dd, *J* = 20.3, 17.7 Hz), 64.1, 53.7, 35.2, 31.4.

^{19}F NMR (282 MHz, Acetone-*d*$_6$) δ -78.9 (s, 3F), -81.0 (d, *J* = 16.7 Hz, 1F), -82.7 (d, *J* = 16.4 Hz, 1F).

HRMS (ESI): calculated for $C_{16}H_{24}F_2N$ [M-OTf]$^+$: 268.1882, found: 268.1877.

1j

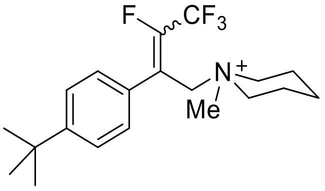

$^{-}$OTf

Chemical Formula: $CF_3O_3S^-$
Exact Mass: 148.9526

Chemical Formula: $C_{20}H_{28}F_4N^+$
Exact Mass: 358.2152

This compound was obtained following the general procedure B. Starting from amine **S1j** (0.9 g, 2.6 mmol), methyl trifluoromethanesulfonate (0.56 g, 3.4 mmol, 1.3 equiv), and diethyl ether (10 mL). Filtration to afford **1j** (1.3 g, 99%, dr = 4.9/1) as a white solid.

^{1}H NMR (300 MHz, Acetone-*d*$_6$) δ 7.75 – 7.52 (m, 4H), 4.93 – 4.86 (m, 2H, minor), 4.85 – 4.72 (m, 2H, major), 3.66 – 3.40 (m, 4H), 3.20 (s, 3H), 2.00 – 1.79 (m, 4H), 1.78 – 1.54 (m, 2H), 1.34 (s, 9H, minor), 1.33 (s, 9H, major).

^{13}C NMR (75 MHz, Acetone-*d*$_6$) δ 154.2, 129.8, 129.7 (major), 129.4 (minor), 127.0 (minor), 126.8 (major), 63.9 – 63.6 (m, major), 63.5 – 63.3 (m, minor), 63.0, 48.8, 35.4, 31.4, 21.3 (major), 21.2 (minor), 20.7 (minor), 20.6 (major).

^{19}F NMR (282 MHz, Acetone-*d*$_6$) δ -65.1 (d, *J* = 8.8 Hz, 3F, minor), -65.4 (d, *J* = 8.1 Hz, 3F, major), -78.9 (s, 3F), -113.8 – -114.2 (m, 1F, major), -114.6 – -114.9 (m, 1F, minor).

HRMS (ESI): calculated for $C_{20}H_{28}F_4N$ [M-OTf]$^+$: 358.2163, found: 358.2158.

1n

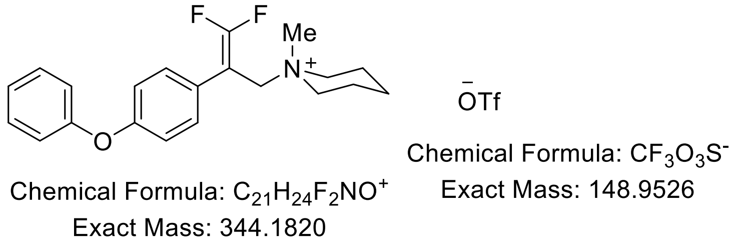

This compound was obtained following the general procedure B. Starting from amine **S1n** (0.58 g, 1.76 mmol), methyl trifluoromethanesulfonate (0.38 g, 2.3 mmol, 1.3 equiv), and diethyl ether (10 mL). Filtration to afford **1n** (0.8 g, 92%) as a white solid.

^{1}H NMR (300 MHz, Acetone-d_6) δ 7.72 – 7.64 (m, 2H), 7.46 – 7.36 (m, 2H), 7.22 – 7.15 (m, 1H), 7.11 – 7.02 (m, 4H), 4.71 (t, J = 2.0 Hz, 2H), 3.56 (t, J = 5.8 Hz, 4H), 3.18 (s, 3H), 2.03 – 1.80 (m, 4H), 1.79 – 1.55 (m, 2H).

^{13}C NMR (75 MHz, Acetone-d_6) δ 158.6, 158.4, 157.3, 131.4 (t, J = 2.6 Hz), 130.9, 127.3 – 127.1 (m), 124.9, 120.2, 119.6, 85.4 (dd, J = 20.3, 18.0 Hz), 63.0, 61.9, 21.5, 20.6.

^{19}F NMR (282 MHz, Acetone-d_6) δ -78.9 (s, 3F), -80.6 (d, J = 16.9 Hz, 1F), -82.2 (d, J = 16.9 Hz, 1F).

HRMS (ESI): calculated for $C_{21}H_{24}F_2NO$ [M-OTf]$^+$: 344.1831, found: 344.1826.

1o

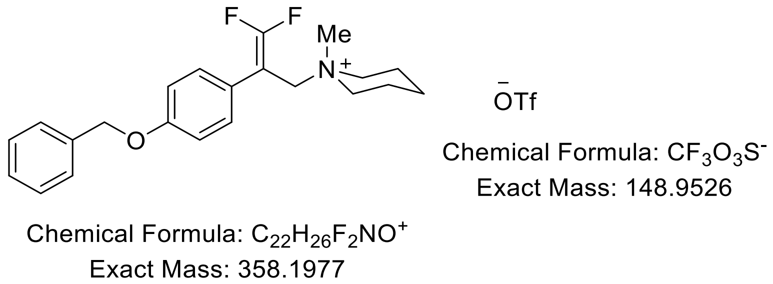

This compound was obtained following the general procedure B. Starting from amine **S1o** (0.46 g, 1.24 mmol), methyl trifluoromethanesulfonate (0.26 g, 1.6 mmol, 1.2 equiv), and diethyl ether (5 mL). Filtration to afford **1o** (0.53 g, 84%) as a white solid.

^{1}H NMR (300 MHz, $CDCl_3$) δ 7.45 – 7.29 (m, 7H), 7.07 – 6.99 (m, 2H), 5.04 (s, 2H), 4.51 (s, 2H), 3.36 (t, J = 5.8 Hz, 4H), 2.96 (s, 3H), 1.86 – 1.54 (m, 6H).

^{13}C NMR (75 MHz, $CDCl_3$) δ 159.3, 136.5, 129.8, 128.8, 128.3, 127.7, 123.0, 122.9 – 122.7 (m), 116.0, 70.3, 61.4, 47.7, 20.6, 20.2.

^{19}F NMR (282 MHz, $CDCl_3$) δ -78.4 (s, 3F), -79.2 (d, J = 14.0 Hz, 1F), -80.3 (d, J = 13.9 Hz, 1F).

HRMS (ESI): calculated for $C_{22}H_{26}F_2NO$ [M-OTf]$^+$: 358.1987, found: 358.1981.

1p

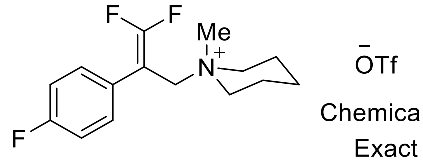

Chemical Formula: $CF_3O_3S^-$
Exact Mass: 148.9526

Chemical Formula: $C_{15}H_{19}F_3N^+$
Exact Mass: 270.1464

This compound was obtained following the general procedure B. Starting from amine **S1p** (400 mg, 1.56 mmol), methyl trifluoromethanesulfonate (340 mg, 2.0 mmol, 1.2 equiv), and diethyl ether (5 mL). Filtration to afford **1p** (0.5 g, 77%) as a white solid.

^{1}H NMR (300 MHz, $CDCl_3$) δ 7.57 – 7.45 (m, 2H), 7.17 – 7.05 (m, 2H), 4.51 (s, 2H), 3.36 (t, *J* = 5.6 Hz, 4H), 2.95 (s, 3H), 1.90 – 1.73 (m, 4H), 1.73 – 1.50 (m, 2H).

^{13}C NMR (75 MHz, $CDCl_3$) δ 164.5, 159.4 (d, *J* = 269.6 Hz), 130.6 (d, *J* = 8.1 Hz), 120.84 (d, *J* = 320.3 Hz), 116.7 (d, *J* = 21.9 Hz), 116.58, 84.5 – 83.9 (m), 62.83 – 62.02 (m), 61.4, 47.4, 20.6, 20.1.

^{19}F NMR (282 MHz, $CDCl_3$) δ -78.2 (d, *J* = 11.1 Hz, 1F), -78.6 (s, 3F), -79.3 (d, *J* = 11.3 Hz, 1F), -111.6 – -111.8 (m, 1F).

HRMS (ESI): calculated for $C_{15}H_{19}F_3N$ [M-OTf]$^+$: 270.1475, found: 270.1468.

1s

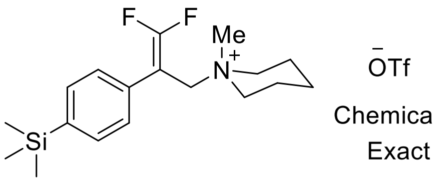

Chemical Formula: $CF_3O_3S^-$
Exact Mass: 148.9526

Chemical Formula: $C_{18}H_{28}F_2NSi^+$
Exact Mass: 324.1954

This compound was obtained following the general procedure B. Starting from amine **S1s** (530 mg, 1.7 mmol), methyl trifluoromethanesulfonate (340 mg, 2.0 mmol, 1.2 equiv), and diethyl ether (10 mL). Filtration to afford **1s** (0.7 g, 87%) as a white solid.

^{1}H NMR (300 MHz, $CDCl_3$) δ 7.64 – 7.52 (m, 2H), 7.48 – 7.37 (m, 2H), 4.57 (s, 2H), 3.39 (t, *J* = 5.4 Hz, 4H), 2.97 (s, 3H), 1.92 – 1.76 (m, 4H), 1.77 – 1.55 (m, 3H), 0.33 – 0.19 (m, 9H).

^{13}C NMR (75 MHz, $CDCl_3$) δ 143.1 – 141.2 (m), 134.6, 127.6, 127.5, 61.4, 47.7, 20.6, 20.2, -1.2.

^{19}F NMR (282 MHz, $CDCl_3$) δ -77.8 – -78.0 (m, 1F), -78.5 (s, 3F), -79.2 (d, *J* = 12.7 Hz, 1F).

HRMS (ESI): calculated for $C_{18}H_{28}F_2NSi^+$ [M-OTf]$^+$: 324.1964, found: 324.1963.

1t

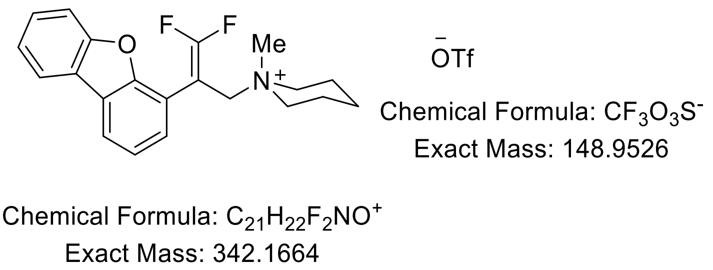

This compound was obtained following the general procedure B. Starting from amine **S1t** (0.4 g, 1.22 mmol), methyl trifluoromethanesulfonate (0.26 g, 1.58 mmol, 1.3 equiv), and diethyl ether (5 mL). Filtration to afford **1t** (0.54 g, 90%) as a white solid.

^{1}H NMR (300 MHz, Acetone-*d*$_6$) δ 8.28 – 8.11 (m, 2H), 7.89 – 7.81 (m, 1H), 7.75 – 7.66 (m, 1H), 7.63 – 7.39 (m, 3H), 4.93 (s, 2H), 3.59 (t, *J* = 5.9 Hz, 4H), 3.21 (s, 3H), 2.00 – 1.76 (m, 4H), 1.76 – 1.45 (m, 2H).

^{13}C NMR (75 MHz, Acetone-*d*$_6$) δ 158.4 (t, *J* = 296.3 Hz), 156.9, 153.8, 128.9, 128.8, 125.9, 124.6, 124.6, 124.4, 122.8, 122.1, 120.1, 116.6 (t, *J* = 3.1 Hz), 112.8, 81.8 – 81.0 (m), 62.4, 61.9, 47.7, 21.4, 20.6.

^{19}F NMR (282 MHz, Acetone-*d*$_6$) δ -76.9 (s, 1F), -78.6 (s, 1F), -78.8 (s, 3F).

HRMS (ESI): calculated for $C_{21}H_{22}F_2NO$ [M-OTf]$^+$: 342.1674, found: 342.1666.

1u

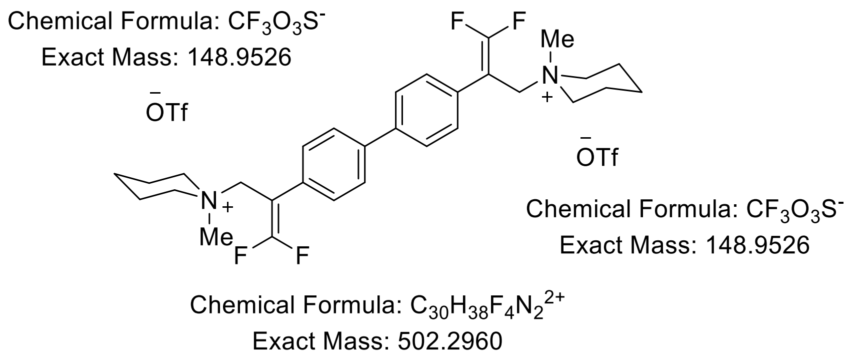

This compound was obtained following the general procedure B. Starting from amine **S1u** (235 mg, 0.5 mmol), methyl trifluoromethanesulfonate (262 mg, 1.3 mmol, 1.2 equiv), and diethyl ether (5 mL). Filtration to afford **1u** (0.28 g, 70%) as a white solid.

^{1}H NMR (300 MHz, Acetone-*d*$_6$) δ 7.89 – 7.71 (m, 8H), 4.78 (s, 4H), 3.59 (t, *J* = 5.8 Hz, 8H), 3.28 – 3.12 (m, 6H), 2.85 (s, 8H), 2.03 – 1.83 (m, 8H), 1.74 – 1.59 (m, 4H).

^{13}C NMR (75 MHz, Acetone-*d*$_6$) δ 140.8, 132.2, 130.43 – 130.12 (m), 128.3, 62.0, 54.5, 47.8, 21.5, 20.6.

^{19}F NMR (282 MHz, Acetone-*d*$_6$) δ -78.9 (s, 3F), -79.7 (d, *J* = 15.1 Hz, 1F), -81.5 (d, *J* = 14.8 Hz, 1F).

HRMS (ESI): (m/z, z = 2) calculated for $C_{30}H_{38}F_4N_2$ $[M-2OTf]^{2+}$: 251.1491, found: 251.1482.

Compound 3

3a

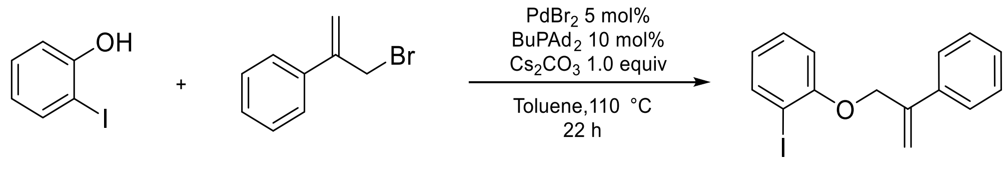

1a
(1.0 equiv)

2c

3a, 73%

This compound was obtained following the general procedure C. Starting from 2-iodophenol **1a** (44 mg, 0.2 mmol), 2-phenylallyl bromide **2c** (39.2 mg, 0.2 mmol), $PdBr_2$ (2.7 mg, 0.01 mmol), **L1** (7.2 mg, 0.02 mmol), Cs_2CO_3 (130 mg, 0.4 mmol, 2.0 equiv), and toluene (2.5 mL). Purification on basic aluminum oxide (Pentane/Ethyl acetate gradient from 99/1 to 95/5) afforded **4a** (48 mg, 73%) as a colorless oil.

^{1}H NMR (300 MHz, $CDCl_3$) δ 7.80 (dd, *J* = 7.8, 1.6 Hz, 1H), 7.54 – 7.45 (m, 2H), 7.42 – 7.26 (m, 4H), 6.88 (dd, *J* = 8.3, 1.4 Hz, 1H), 6.78 – 6.69 (m, 1H), 5.68 – 5.59 (m, 2H), 4.95 (t, *J* = 1.4 Hz, 2H).

^{13}C NMR (75 MHz, $CDCl_3$) δ 157.2, 142.5, 139.7, 138.5, 129.5, 128.6, 128.2, 126.3, 123.0, 114.9, 112.7, 86.8, 70.61.

The NMR data obtained agreed with the literature.[2]

3b

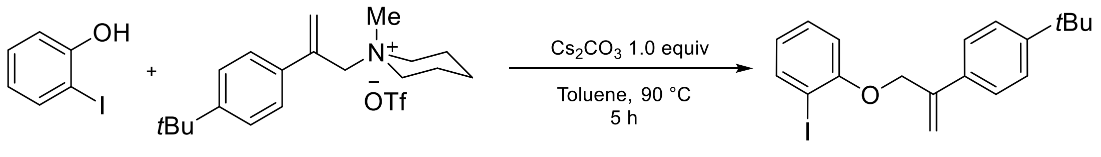

1a
(1.0 equiv)

2d

3b, 95%

To a Schlenk tube equipped with a magnetic stir bar were added 2-iodophenol **1a** (88 mg, 0.4 mmol, 1.0 equiv), ammonium salt **2d** (168.4 mg, 0.4 mmol), Cs_2CO_3 (130 mg, 0.4 mmol, 1.0 equiv), and toluene (2 mL). The resulting solution was stirred at 90°C for 5 h. After the reaction mixture was cooled to room temperature, the reaction mixture was extracted with ethyl acetate (3 × 20 mL). The combined organic layers were dried with Na_2SO_4 and the solvent was removed under reduced pressure. The resultant crude product material was purified by flash chromatography on silica gel (Pentane/Ethyl acetate gradient from 99/1 to 95/5), affording compound **3b** (149 mg, 95%) as a colorless oil.

^{1}H NMR (300 MHz, $CDCl_3$) δ 7.84 – 7.76 (m, 1H), 7.48 – 7.37 (m, 4H), 7.33 – 7.26 (m, 1H), 6.91 – 6.83 (m, 1H), 6.77 – 6.68 (m, 1H), 5.65 – 5.57 (m, 2H), 4.95 (s, 2H), 1.35 (s, 9H).

^{13}C NMR (75 MHz, $CDCl_3$) δ 157.3, 151.3, 142.1, 139.7, 135.6, 129.5, 125.9, 125.6, 122.9, 114.0, 112.7, 86.8, 70.6, 34.7, 31.4.

HRMS (EI): calculated for $C_{19}H_{21}OI$ $[M]^+$: 392.0632, found: 392.0633.

3c

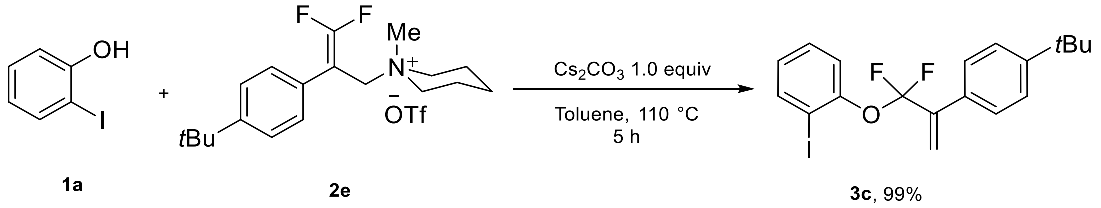

To a Schlenk tube equipped with a magnetic stir bar were added 2-iodophenol **1a** (88 mg, 0.4 mmol, 1.0 equiv), ammonium salt **2e** (182.8 mg, 0.4 mmol), Cs_2CO_3 (130 mg, 0.4 mmol, 1.0 equiv), and toluene (2 mL). The resulting solution was stirred at 110°C for 5 h. After the reaction mixture was cooled to room temperature, the reaction mixture was extracted with ethyl acetate (3 × 20 mL). The combined organic layers were dried with Na_2SO_4 and the solvent was removed under reduced pressure. The resultant crude product material was purified by flash chromatography on silica gel (Pentane/Ethyl acetate gradient from 99/1 to 98/8), affording compound **3c** (170 mg, 99%) as a colorless oil.

^{1}H NMR (300 MHz, $CDCl_3$) δ 7.82 (dd, J = 7.9, 1.6 Hz, 1H), 7.55 – 7.47 (m, 2H), 7.44 – 7.37 (m, 3H), 7.37 – 7.29 (m, 1H), 6.98 – 6.88 (m, 1H), 6.18 – 6.11 (m, 1H), 5.78 – 5.69 (m, 1H), 1.35 (s, 9H).

^{13}C NMR (75 MHz, $CDCl_3$) δ 151.7, 150.8, 141.3 (t, J = 29.4 Hz), 139.9, 132.3, 129.4, 127.7, 127.0, 125.4, 122.0, 121.8 (t, J = 2.3 Hz), 119.9 (t, J = 6.0 Hz), 90.2, 34.7, 31.4.

^{19}F NMR (282 MHz, $CDCl_3$) δ -66.8 (s, 2F).

HRMS (EI): calculated for $C_{19}H_{19}F_2IO$ $[M]^+$: 428.0443, found: 428.0443.

Compound 4

4b

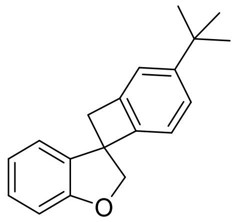

Chemical Formula: $C_{19}H_{20}O$
Exact Mass: 264.1514

This compound was obtained following the general procedure C. Starting from 2-iodophenol **1a** (44 mg, 0.2 mmol), ammonium salt **2d** (84 mg, 0.2 mmol), $PdBr_2$ (2.7 mg, 0.01 mmol), **L1** (7.2 mg, 0.02 mmol), Cs_2CO_3 (130 mg, 0.4 mmol, 2.0 equiv), and toluene (2.5 mL). Purification on basic aluminum oxide (Pentane/Ethyl acetate gradient from 99/1 to 95/5) afforded **4b** (46 mg, 87%) as a white solid.

^{1}H NMR (300 MHz, $CDCl_3$) δ 7.36 – 7.30 (m, 1H), 7.27 – 7.23 (m, 1H), 7.22 – 7.14 (m, 1H), 7.07 – 6.96 (m, 2H), 6.92 – 6.80 (m, 2H), 4.84 (d, *J* = 9.4 Hz, 1H), 4.78 (d, *J* = 9.5 Hz, 1H), 3.53 (d, *J* = 13.9 Hz, 1H), 3.46 (d, *J* = 14.2 Hz, 1H), 1.36 (s, 9H).

^{13}C NMR (75 MHz, $CDCl_3$) δ 160.2, 152.0, 145.3, 142.9, 132.2, 128.8, 125.1, 123.3, 121.0, 120.9, 120.4, 109.8, 80.4, 55.8, 48.0, 35.4, 31.8.

The NMR data obtained agreed with the literature.[3]

4c

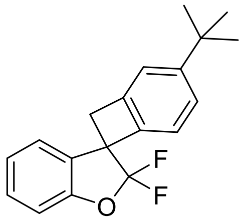

Chemical Formula: $C_{19}H_{18}F_2O$
Exact Mass: 300.1326

This compound was obtained following the general procedure C. Starting from 2-iodophenol **1a** (44 mg, 0.2 mmol), ammonium salt **2e** (91.4 mg, 0.2 mmol), $PdBr_2$ (2.7 mg, 0.01 mmol), **L1** (7.2 mg, 0.02 mmol), Cs_2CO_3 (130 mg, 0.4 mmol, 2.0 equiv), and toluene (2.5 mL). Purification on basic aluminum oxide (Pentane/Ethyl acetate gradient from 99/1 to 95/5) afforded **4c** (51 mg, 83%) as a colorless oil. This compound can also be synthesized using 2-bromophenol **1m** (34.4 mg, 0.2 mmol) or 2-chlorophenol **1n** (25.7 mg, 0.2 mmol) instead of **1a**, the corresponding product **4c** was obtained in 60% and 58% yield, respectively.

^{1}H NMR (300 MHz, $CDCl_3$) δ 7.38 – 7.33 (m, 1H), 7.31 – 7.23 (m, 2H), 7.13 – 6.96 (m, 4H), 4.02 (d, J = 14.0 Hz, 1H), 3.39 – 3.26 (m, 1H), 1.36 (s, 9H).

^{13}C NMR (75 MHz, $CDCl_3$) δ 154.6 (d, *J* = 3.0 Hz), 153.1, 142.5, 139.2 (dd, *J* = 7.5, 1.5 Hz), 132.4 (t, *J* = 264.8 Hz), 129.7 (d, *J* = 2.3 Hz), 129.6, 125.5, 123.6, 123.5, 122.6, 120.1, 110.3, 58.5(t, *J* = 30.0 Hz), 41.6 (dd, *J* = 9.0, 4.5 Hz), 35.5, 31.7.

^{19}F NMR (282 MHz, $CDCl_3$) δ -70.3 (dd, *J* = 148.7, 3.0 Hz, 1F), -79.5 (d, *J* = 148.6 Hz, 1F).

HRMS (EI): calculated for $C_{19}H_{18}F_2O$ $[M]^+$: 300.1320, found: 300.1319.

4d

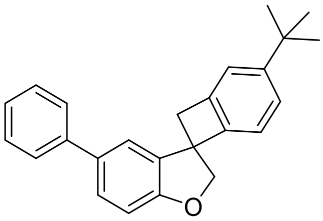

Chemical Formula: $C_{25}H_{24}O$
Exact Mass: 340.1827

This compound was obtained following the general procedure C. Starting from 2-iodophenol **1e** (59.2 mg, 0.2 mmol), ammonium salt **2d** (84.2 mg, 0.2 mmol), $PdBr_2$ (2.7 mg, 0.01 mmol), **L1** (7.2 mg, 0.02 mmol), Cs_2CO_3 (130 mg, 0.4 mmol, 2.0 equiv), and toluene (2.5 mL). Purification on basic aluminum oxide (Pentane/Ethyl acetate gradient from 98/2 to 95/5) afforded **4d** (42 mg, 62%) as a white solid.

^{1}H NMR (300 MHz, $CDCl_3$) δ 7.53 – 7.31 (m, 6H), 7.30 – 7.20 (m, 3H), 7.04 (dd, *J* = 7.8, 1.0 Hz, 1H), 6.95 (d, *J* = 8.3 Hz, 1H), 4.86 (q, *J* = 18.0, 9.0 Hz, 2H), 3.63 – 3.47 (m, 2H), 1.37 (s, 9H).

^{13}C NMR (75 MHz, $CDCl_3$) δ 159.9, 152.1, 145.1, 142.8, 141.4, 134.7, 132.9, 128.7, 128.0, 127.0, 126.7, 125.2, 122.1, 121.0, 120.4, 110.0, 80.9, 55.8, 48.1, 35.4, 31.8.

HRMS (EI): calculated for $C_{25}H_{14}O$ $[M]^+$: 340.1822, found: 340.1818.

4e

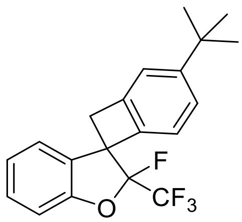

Chemical Formula: $C_{20}H_{18}F_4O$
Exact Mass: 350.1294

This compound was obtained following the general procedure C. Starting from 2-iodophenol **1a** (44 mg, 0.2 mmol), ammonium salt **2j** (101.4 mg, 0.2 mmol), $PdBr_2$ (2.7 mg, 0.01 mmol), **L1** (7.2 mg, 0.02 mmol), Cs_2CO_3 (130 mg, 0.4 mmol, 2.0 equiv), and toluene (2.5 mL). Purification on basic aluminum oxide (Pentane/Ethyl acetate gradient from 99/1 to 95/5) afforded **4e** (50

mg, 71%, dr >20/1) as a slight yellow solid.

^{1}H NMR (400 MHz, $CDCl_3$) δ 7.31 – 7.23 (m, 3H), 7.10 – 6.98 (m, 3H), 6.88 – 6.81 (m, 1H), 4.12 – 4.02 (m, 1H), 3.49 (d, *J* = 14.5 Hz, 1H), 1.34 (s, 9H).

^{13}C NMR (75 MHz, $CDCl_3$) δ 155.8 (d, *J* = 2.8 Hz), 152.8, 141.8, 141.3 (t, *J* = 9.0 Hz), 132.6 (dd, *J* = 9.2, 4.9 Hz), 129.7, 129.6, 125.3, 123.9, 123.3, 122.33 (dd, *J* = 3.9, 1.9 Hz), 119.9, 110.8, 59.5 (d, *J* = 26.0 Hz), 39.7 (d, *J* = 15.1 Hz), 35.4, 31.7.

^{19}F NMR (282 MHz, $CDCl_3$) δ -80.8 (d, *J* = 3.7 Hz), -117.1 (q, *J* = 4.1 Hz).

HRMS (EI): calculated for $C_{20}H_{18}F_4O$ $[M]^+$: 350.1288, found: 350.1282.

4f

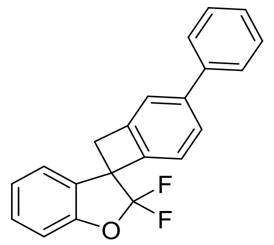

Chemical Formula: $C_{21}H_{14}F_2O$
Exact Mass: 320.1013

This compound was obtained following the general procedure C. Starting from ammonium salt **1j** (95.4 mg, 0.2 mmol), 2-iodophenol **2a** (44 mg, 0.2 mmol), $PdBr_2$ (2.7 mg, 0.01 mmol), **L1** (7.2 mg, 0.02 mmol), Cs_2CO_3 (130 mg, 0.4 mmol, 2.0 equiv), and toluene (2.5 mL). Purification on basic aluminum oxide (Pentane/Ethyl acetate gradient from 99/1 to 95/5) afforded **4e** (50 mg, 78%) as a white solid.

^{1}H NMR (300 MHz, $CDCl_3$) δ 7.63 – 7.55 (m, 2H), 7.55 – 7.42 (m, 4H), 7.42 – 7.27 (m, 2H), 7.21 – 7.01 (m, 4H), 4.10 (d, *J* = 14.2 Hz, 1H), 3.47 – 3.33 (m, 1H).

^{13}C NMR (75 MHz, $CDCl_3$) δ 154.6 (d, *J* = 2.1 Hz), 143.4, 143.3, 141.8, 141.31 (dd, *J* = 7.3, 1.8 Hz), 132.3 (t, *J* = 264.8 Hz), 129.8, 129.4 (d, *J* = 2.3 Hz), 129.0, 127.8, 127.6, 127.5, 123.7, 123.5, 123.4, 122.1, 110.4, 58.7 (t, *J* = 29.3 Hz), 41.5 (dd, *J* = 9.3, 4.4 Hz).

^{19}F NMR (282 MHz, $CDCl_3$) δ -70.3 (dd, *J* = 148.7, 3.0 Hz), -79.3 (d, *J* = 148.8 Hz).

HRMS (EI): calculated for $C_{21}H_{14}F_2O$ $[M]^+$: 320.1007, found: 320.1007.

4g

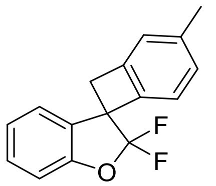

Chemical Formula: $C_{16}H_{12}F_2O$
Exact Mass: 258.0856

This compound was obtained following the general procedure C. Starting from 2-iodophenol **1a** (44 mg, 0.2 mmol), ammonium salt **2l** (83 mg, 0.2 mmol), $PdBr_2$ (2.7 mg, 0.01 mmol), **L1** (7.2 mg, 0.02 mmol), Cs_2CO_3 (130 mg, 0.4 mmol, 2.0 equiv), and toluene (2.5 mL). Purification on basic aluminum oxide (Pentane/Ethyl acetate gradient from 99/1 to 95/5) afforded **4g** (27.5 mg, 53%) as a colorless oil.

^{1}H NMR (300 MHz, $CDCl_3$) δ 7.32 – 7.25 (m, 1H), 7.15 – 7.09 (m, 1H), 7.09 – 6.95 (m, 5H), 4.01 (d, *J* = 14.1 Hz, 1H), 3.36 – 3.27 (m, 1H), 2.41 (s, 3H).

^{13}C NMR (75 MHz, $CDCl_3$) δ 154.4(d, *J* = 2.1 Hz), 151.7, 142.8, 139.6, 139. 0 (dd, *J* = 7.6, 2.1 Hz), 132.2 (t, *J* = 264.8 Hz), 129.5, 129.0, 123.6, 123.5, 123.3, 122.8, 110.2, 58.4 (t, *J* = 30.1 Hz), 41.3 (dd, *J* = 9.4, 4.5 Hz), 22.3.

^{19}F NMR (282 MHz, $CDCl_3$) δ -70.6 (dd, *J* = 148.7, 3.0 Hz), -79.6 (d, *J* = 148.7 Hz).

HRMS (EI): calculated for $C_{16}H_{12}F_2O$ $[M]^+$: 258.0851, found: 258.0849.

4h

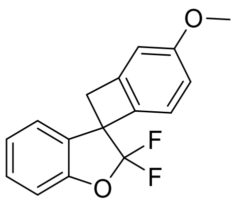

Chemical Formula: $C_{16}H_{12}F_2O_2$
Exact Mass: 274.0805

This compound was obtained following the general procedure C. Starting from 2-iodophenol **1a** (44 mg, 0.2 mmol), ammonium salt **2m** (86 mg, 0.2 mmol), $PdBr_2$ (2.7 mg, 0.01 mmol), **L1** (7.2 mg, 0.02 mmol), Cs_2CO_3 (130 mg, 0.4 mmol, 2.0 equiv), and toluene (2.5 mL). Purification on basic aluminum oxide (Pentane/Ethyl acetate gradient from 99/1 to 90/10) afforded **4h** (43 mg, 78%) as a colorless oil.

^{1}H NMR (300 MHz, $CDCl_3$) δ 7.32 – 7.23 (m, 1H), 7.09 – 6.95 (m, 4H), 6.89 – 6.78 (m, 2H), 3.98 (d, *J* = 14.2 Hz, 1H), 3.83 (s, 3H), 3. 34 – 3.24 (m, 1H).

^{13}C NMR (75 MHz, $CDCl_3$) δ 161.2, 154.4 (d, *J* = 4.3 Hz), 143.7, 133.6 (dd, *J* = 7.8, 1.7 Hz), 132.2 (t, *J* = 264.8 Hz), 129.6, 129.5, 124.4, 123.5, 123.3, 115.1, 110.2, 108.6, 57.96 (t, *J* = 29.3 Hz),

55.5, 41.1 (dd, J = 9.5, 4.7 Hz).

^{19}F NMR (282 MHz, $CDCl_3$) δ -70.6 (dd, J = 148.8, 3.1 Hz), -80.1 (d, J = 148.9 Hz).

HRMS (EI): calculated for $C_{16}H_{12}F_2O_2$ $[M]^+$: 274.0800, found: 274.0805.

4i

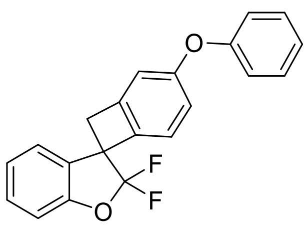

Chemical Formula: $C_{21}H_{14}F_2O_2$
Exact Mass: 336.0962

This compound was obtained following the general procedure C. Starting from 2-iodophenol **1a** (88 mg, 0.4 mmol), ammonium salt **2n** (197 mg, 0.4 mmol), $PdBr_2$ (1.06 mg, 0.004 mmol), **L1** (2.9 mg, 0.008 mmol), Cs_2CO_3 (260 mg, 0.8 mmol, 2.0 equiv), and toluene (5.0 mL). Purification on basic aluminum oxide (Pentane/Ethyl acetate gradient from 99/1 to 90/10) afforded **4i** (95 mg, 70%) as a white solid.

^{1}H NMR (300 MHz, $CDCl_3$) δ 7.42 – 7.23 (m, 4H), 7.19 – 6.91 (m, 8H), 6.92 – 6.85 (m, 1H), 3.99 (d, J = 14.3 Hz, 1H), 3.30 (dd, J = 14.3, 2.2 Hz, 1H).

^{13}C NMR (75 MHz, $CDCl_3$) δ 159.0, 157.2, 154.7 – 154.5 (m), 144.0, 136.5 (d, J = 9.1 Hz), 132.2, 130.0, 129.8, 129.4 – 129.3 (m), 124.8, 123.7, 123.7, 123.4, 119.6, 119.3, 114.0, 110.4, 58.7 – 57.7 (m), 41.3 (dd, J = 9.5, 4.6 Hz).

^{19}F NMR (282 MHz, $CDCl_3$) δ -70.5 (dd, J = 148.7, 3.1 Hz, 1F), -79.9 (d, J = 148.6 Hz, 1F).

HRMS (EI): calculated for $C_{21}H_{14}F_2O_2$ $[M]^+$: 336.0956, found: 336.0950.

4j

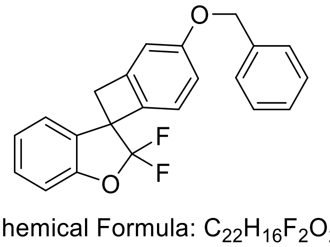

Chemical Formula: $C_{22}H_{16}F_2O_2$
Exact Mass: 350.1118

This compound was obtained following the general procedure C. Starting from 2-iodophenol **1a** (44 mg, 0.2 mmol), ammonium salt **2o** (114 mg, 0.2 mmol), $PdBr_2$ (2.7 mg, 0.01 mmol), **L1** (7.2 mg, 0.02 mmol), Cs_2CO_3 (130 mg, 0.4 mmol, 2.0 equiv), and toluene (2.5 mL). Purification on basic aluminum oxide (Pentane/Ethyl acetate gradient from 99/1 to 95/5) afforded **4j** (60

mg, 86%) as a white solid.

^{1}H NMR (300 MHz, $CDCl_3$) δ 7.50 – 7.32 (m, 5H), 7.32 – 7.24 (m, 1H), 7.09 – 6.98 (m, 4H), 6.98 – 6.87 (m, 2H), 5.09 (s, 2H), 3.99 (d, *J* = 14.2 Hz, 1H), 3.35 – 3.24 (m, 1H).

^{13}C NMR (75 MHz, $CDCl_3$) δ 160.6, 154.5 (d, *J* = 2.2 Hz), 143.8, 137.0, 134.1 (dd, *J* = 7.6, 1.7 Hz), 132.3 (t, *J* = 264.8 Hz), 129.7, 129.6, 128.8, 128.2, 127.6, 124.6, 123.6, 123.4, 116.1, 110.3, 109.7, 70.5, 58.1 (t, *J* = 29.3 Hz), 41.3 (dd, *J* = 9.5, 4.6 Hz).

^{19}F NMR (282 MHz, $CDCl_3$) δ -70.5 (dd, *J* = 148.7, 3.0 Hz), -80.1 (d, *J* = 148.7 Hz).

HRMS (EI): calculated for $C_{22}H_{16}F_2O_2$ $[M]^+$: 350.1113, found: 350.1112.

4k

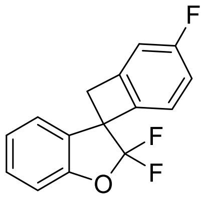

Chemical Formula: $C_{15}H_9F_3O$
Exact Mass: 262.0605

This compound was obtained following the general procedure C. Starting from 2-iodophenol **1a** (44 mg, 0.2 mmol), ammonium salt **2p** (83.8 mg, 0.2 mmol), $PdBr_2$ (2.7 mg, 0.01 mmol), **L1** (7.2 mg, 0.02 mmol), Cs_2CO_3 (130 mg, 0.4 mmol, 2.0 equiv), and toluene (2.5 mL). Purification on basic aluminum oxide (Pentane/Ethyl acetate gradient from 98/2 to 95/5) afforded **4k** (40 mg, 76%) as a colorless oil.

^{1}H NMR (300 MHz, $CDCl_3$) δ 7.33 – 7.26 (m, 1H), 7.12 – 6.92 (m, 6H), 4.01 (d, *J* = 14.4 Hz, 1H), 3.37 – 3.25 (m, 1H).

^{13}C NMR (75 MHz, $CDCl_3$) δ 164.1 (d, *J* = 247.3 Hz), 154.6 (d, *J* = 2.4 Hz), 144.2 (d, *J* = 8.3 Hz), 137.6 – 137.3 (m), 132.0 (t, *J* = 264.8 Hz), 130.0, 129.0 (d, *J* = 1.6 Hz), 125.2 (d, *J* = 9.2 Hz), 123.8, 123.4, 116.1 (d, *J* = 24.1 Hz), 111.2 (d, *J* = 23.0 Hz), 110.5, 58.2 (t, *J* = 29.3 Hz), 41.3 – 40.8 (m).

^{19}F NMR (282 MHz, $CDCl_3$) δ -70.6 (dd, *J* = 148.7, 3.0 Hz, 1F), -79.9 (d, *J* = 149.0 Hz, 1F), -109.4 – -109.7 (m, 1F).

HRMS (EI): calculated for $C_{15}H_9F_3O$ $[M]^+$: 262.0600, found: 262.0596.

4l

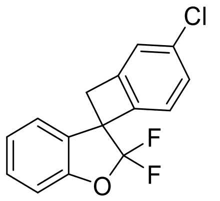

Chemical Formula: $C_{15}H_9ClF_2O$
Exact Mass: 278.0310

This compound was obtained following the general procedure C. Starting from 2-iodophenol **1a** (44 mg, 0.2 mmol), ammonium salt **2q** (87 mg, 0.2 mmol), $PdBr_2$ (2.7 mg, 0.01 mmol), **L1** (7.2 mg, 0.02 mmol), Cs_2CO_3 (130 mg, 0.4 mmol, 2.0 equiv), and toluene (2.5 mL). Purification on basic aluminum oxide (Pentane/Ethyl acetate gradient from 99/1 to 95/5) afforded **4l** (40 mg, 72%) as a colorless oil.

^{1}H NMR (300 MHz, $CDCl_3$) δ 7.36 – 7.26 (m, 3H), 7.12 – 7.01 (m, 4H), 4.05 (d, *J* = 14.5 Hz, 1H), 3.42 – 3.30 (m, 1H).

^{13}C NMR (75 MHz, $CDCl_3$) δ 154.7 – 154.6 (m), 144.2, 140.47 (dd, *J* = 9.5, 2.3 Hz), 135.6, 132.0 (t, *J* = 265.5 Hz), 130.0, 129.0, 128.8 (d, *J* = 2.2 Hz), 124.8, 123.9, 123.8, 123.4, 110.5, 58.5 (t, *J* = 30.1 Hz), 41.4 (dd, *J* = 9.5, 4.5 Hz).

^{19}F NMR (282 MHz, $CDCl_3$) δ -70.5 (dd, *J* = 148.7, 3.0 Hz, 1F), -79.5 (d, *J* = 148.7 Hz, 1F).

HRMS (EI): calculated for $C_{15}H_9ClF_2O$ $[M]^+$: 278.0305, found: 278.0301.

4m

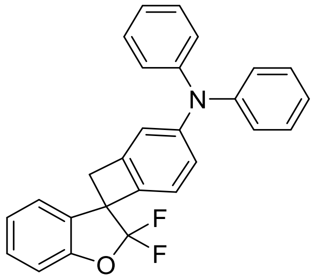

Chemical Formula: $C_{27}H_{19}F_2NO$
Exact Mass: 411.1435

This compound was obtained following the general procedure C. Starting from 2-iodophenol **1a** (44 mg, 0.2 mmol), ammonium salt **2r** (114 mg, 0.2 mmol), $PdBr_2$ (2.7 mg, 0.01 mmol), **L1** (7.2 mg, 0.02 mmol), Cs_2CO_3 (130 mg, 0.4 mmol, 2.0 equiv), and toluene (2.5 mL). Purification on basic aluminum oxide (Pentane/Ethyl acetate gradient from 98/2 to 95/5) afforded **4m** (66 mg, 80%) as a slight yellow solid.

^{1}H NMR (300 MHz, $CDCl_3$) δ 7.32 – 7.24 (m, 5H), 7.17 – 7.10 (m, 5H), 7.09 – 6.99 (m, 5H), 6.98 – 6.93 (m, 2H), 3.95 (d, *J* = 14.2 Hz, 1H), 3.32 – 3.20 (m, 1H).

^{13}C NMR (75 MHz, $CDCl_3$) δ 154.6 (d, *J* = 2.3 Hz), 149.7, 148.0, 143.5, 135.9 (dd, *J* = 7.4, 1.9 Hz), 132.3 (t, *J* = 264.8 Hz), 129.7, 129.59 (d, *J* = 2.2 Hz), 129.4, 124.7, 124.6, 124.1, 123.6, 123.5, 123.1, 118.8, 110.3, 58.4 (t, *J* = 30.1 Hz), 41.4 (dd, *J* = 9.4, 4.6 Hz).

^{19}F NMR (282 MHz, $CDCl_3$) δ -70.3 (dd, *J* = 148.6, 3.1 Hz, 1F), -79.8 (d, *J* = 148.5 Hz, 1F).

HRMS (EI): calculated for $C_{27}H_{19}F_2NO$ $[M]^+$: 411.1429, found: 411.1424.

4n

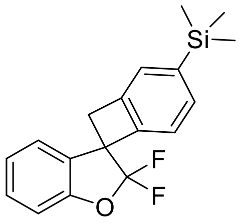

Chemical Formula: $C_{18}H_{18}F_2OSi$
Exact Mass: 316.1095

This compound was obtained following the general procedure C. Starting from 2-iodophenol **1a** (44 mg, 0.2 mmol), ammonium salt **2s** (94.6 mg, 0.2 mmol), $PdBr_2$ (2.7 mg, 0.01 mmol), **L1** (7.2 mg, 0.02 mmol), Cs_2CO_3 (130 mg, 0.4 mmol, 2.0 equiv), and toluene (2.5 mL). Purification on basic aluminum oxide (Pentane/Ethyl acetate gradient from 98/2 to 95/5) afforded **4n** (49 mg, 78%) as a colorless oil.

^{1}H NMR (300 MHz, $CDCl_3$) δ 7.51 – 7.44 (m, 1H), 7.44 – 7.37 (m, 1H), 7.32 – 7.23 (m, 1H), 7.14 – 6.97 (m, 4H), 4.06 (d, *J* = 14.2 Hz, 1H), 3.43 – 3.31 (m, 1H), 0.31 (s, 9H).

^{13}C NMR (75 MHz, $CDCl_3$) δ 154.6 (d, *J* = 2.1 Hz), 143.1 (dd, *J* = 7.4, 1.7 Hz), 142.6, 142.5, 133.0, 132.3 (t, *J* = 264.8 Hz), 129.7, 129.5 (d, *J* = 2.3 Hz), 127.7, 123.6, 123.5, 122.2, 110.3, 59.0 (t, *J* = 30.1 Hz), 41.8 (d, *J* = 5.0 Hz), -0.9.

^{19}F NMR (282 MHz, $CDCl_3$) δ -70.3 (dd, *J* = 148.5, 2.9 Hz, 1F), -79.2 (d, *J* = 148.7 Hz, 1F).

HRMS (EI): calculated for $C_{18}H_{18}F_2OSi$ $[M]^+$: 316.1090, found: 316.1095.

4o

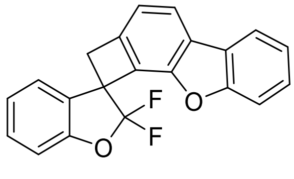

Chemical Formula: $C_{21}H_{12}F_2O_2$
Exact Mass: 334.0805

This compound was obtained following the general procedure C. Starting from 2-iodophenol **1a** (44 mg, 0.2 mmol), ammonium salt **2t** (98.2 mg, 0.2 mmol), $PdBr_2$ (5.4 mg, 0.02 mmol), **L1** (14.4 mg, 0.04 mmol), Cs_2CO_3 (130 mg, 0.4 mmol, 2.0 equiv), and toluene (2.5 mL). Purification

on basic aluminum oxide (Pentane/Ethyl acetate gradient from 98/2 to 95/5) afforded **4o** (40 mg, 60%) as a white solid.

^{1}H NMR (300 MHz, $CDCl_3$) δ 8.03 (d, *J* = 7.7 Hz, 1H), 7.98 – 7.89 (m, 1H), 7.55 – 7.30 (m, 4H), 7.27 – 7.21 (m, 1H), 7.16 – 6.97 (m, 3H), 4.22 (d, *J* = 14.1 Hz, 1H), 3.60 – 3.45 (m, 1H).

^{13}C NMR (75 MHz, $CDCl_3$) δ 156.2, 154.7 (d, *J* = 1.9 Hz), 148.8, 142.5, 132.3 (t, *J* = 264.8 Hz), 130.1, 128.1 (d, *J* = 2.2 Hz), 127.4, 127.3, 125.2, 124.4, 123.8, 123.5, 123.1, 122.7, 120.2, 117.5, 112.2, 110.7, 58.2 (t, *J* = 30.1 Hz), 42.7 (dd, *J* = 9.4, 4.4 Hz).

^{19}F NMR (282 MHz, $CDCl_3$) δ -68.7 (dd, *J* = 147.7, 3.0 Hz, 1F), -79.3 (d, *J* = 147.7 Hz, 1F).

HRMS (EI): calculated for $C_{21}H_{12}F_2O_2$ $[M]^+$: 334.0800, found: 334.0794.

4p

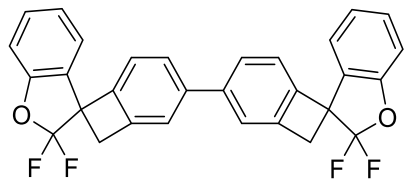

Chemical Formula: $C_{30}H_{18}F_4O_2$
Exact Mass: 486.1243

This compound was obtained following the general procedure C. Starting from 2-iodophenol **1a** (44 mg, 0.2 mmol), ammonium salt **2u** (80 mg, 0.1 mmol), $PdBr_2$ (2.7 mg, 0.01 mmol), **L1** (7.2 mg, 0.02 mmol), Cs_2CO_3 (130 mg, 0.4 mmol, 2.0 equiv), and toluene (2.5 mL). Purification on basic aluminum oxide (Pentane/Ethyl acetate gradient from 98/2 to 95/5) afforded **4p** (25 mg, 51%) as a white solid.

^{1}H NMR (300 MHz, $CDCl_3$) δ 7.54 – 7.46 (m, 2H), 7.44 – 7.39 (m, 2H), 7.35 – 7.27 (m, 2H), 7.22 – 7.15 (m, 2H), 7.15 – 6.99 (m, 6H), 4.11 (d, *J* = 14.2 Hz, 2H), 3.42 (dd, *J* = 14.3, 2.0 Hz, 2H).

^{13}C NMR (75 MHz, $CDCl_3$) δ 154.6 (d, *J* = 2.1 Hz), 143.7, 143.5, 141.5 (dd, *J* = 7.8, 2.0 Hz), 132.3 (t, *J* = 264.8 Hz), 129.8, 129.3 (d, *J* = 2.1 Hz), 128.0, 123.7, 123.5, 122.3, 110.4, 58.71 (t, *J* = 29.7 Hz), 41.5 (dd, *J* = 9.4, 4.5 Hz).

^{19}F NMR (282 MHz, $CDCl_3$) δ -70.3 (dd, *J* = 148.5, 3.0 Hz, 1F), -79.3 (d, *J* = 148.6 Hz, 1F).

HRMS (EI): calculated for $C_{30}H_{18}F_4O_2$ $[M]^+$: 486.1237, found: 486.1235.

4q

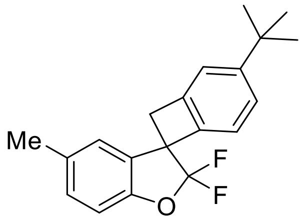

Chemical Formula: $C_{20}H_{20}F_2O$
Exact Mass: 314.1482

This compound was obtained following the general procedure C. Starting from 4-methyl-2-iodophenol **1b** (46.8 mg, 0.2 mmol), ammonium salt **2e** (91.4 mg, 0.2 mmol), $PdBr_2$ (2.7 mg, 0.01 mmol), **L1** (7.2 mg, 0.02 mmol), Cs_2CO_3 (130 mg, 0.4 mmol, 2.0 equiv), and toluene (2.5 mL). Purification on basic aluminum oxide (Pentane/Ethyl acetate gradient from 98/2 to 95/5) afforded **4q** (54 mg, 86%) as a colorless oil.

^{1}H NMR (300 MHz, $CDCl_3$) δ 7.41 – 7.32 (m, 1H), 7.30 – 7.26 (m, 1H), 7.11 – 6.97 (m, 2H), 6.95 – 6.80 (m, 2H), 4.00 (d, *J* = 14.0 Hz, 1H), 3.31 (dd, *J* = 14.1, 2.3 Hz, 1H), 2.27 (s, 3H), 1.37 (s, 9H).

^{13}C NMR (75 MHz, $CDCl_3$) δ 153.0, 152.53 (d, *J* = 3.5 Hz), 142.5, 139.28 (d, *J* = 7.3 Hz), 133.2, 132.5 (t, *J* = 264.8 Hz), 130.0, 129.54 (d, *J* = 2.3 Hz), 125.4, 123.9, 122.6, 120.1, 109.9, 58.6 (t, *J* = 29.3 Hz), 41.5 (dd, *J* = 9.4, 4.6 Hz), 35.5, 31.8, 21.0.

^{19}F NMR (282 MHz, $CDCl_3$) δ -70.3 (dd, *J* = 148.6, 3.0 Hz, 1F), -79.6 (d, *J* = 148.6 Hz, 1F).

HRMS (EI): calculated for $C_{20}H_{20}F_2O$ $[M]^+$: 314.1478, found: 314.1473.

4r

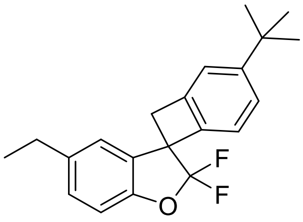

Chemical Formula: $C_{21}H_{22}F_2O$
Exact Mass: 328.1639

This compound was obtained following the general procedure C. Starting from 4-ethyl-2-iodophenol **1c** (50 mg, 0.2 mmol), ammonium salt **2e** (91.4 mg, 0.2 mmol), $PdBr_2$ (2.7 mg, 0.01 mmol), **L1** (7.2 mg, 0.02 mmol), Cs_2CO_3 (130 mg, 0.4 mmol, 2.0 equiv), and toluene (2.5 mL). Purification on basic aluminum oxide (Pentane/Ethyl acetate gradient from 98/2 to 95/5) afforded **4r** (45 mg, 68%) as a colorless oil.

^{1}H NMR (300 MHz, $CDCl_3$) δ 7.41 – 7.32 (m, 1H), 7.33 – 7.25 (m, 1H), 7.15 – 7.00 (m, 2H), 6.97 – 6.85 (m, 2H), 4.01 (d, *J* = 14.3 Hz, 1H), 3.33 (dd, *J* = 14.2, 2.0 Hz, 1H), 2.57 (q, *J* = 7.6 Hz, 2H), 1.37 (s, 9H), 1.18 (t, *J* = 7.6 Hz, 3H).

^{13}C NMR (75 MHz, $CDCl_3$) δ 153.0, 152.7 (d, *J* = 2.3 Hz), 142.6, 139.9, 139.3 (dd, *J* = 7.6, 1.7 Hz), 132.6 (t, *J* = 264.8 Hz), 129.5 (d, *J* = 2.2 Hz), 128.9, 125.4, 122.8, 122.7, 120.1, 110.0, 58.7 (t, *J* = 29.3 Hz), 41.6 (dd, *J* = 9.4, 4.6 Hz), 35.5, 31.8, 28.6, 16.1.

^{19}F NMR (282 MHz, $CDCl_3$) δ -70.1 (dd, *J* = 148.6, 3.0 Hz, 1F), -79.7 (d, *J* = 148.5 Hz, 1F).

HRMS (EI): calculated for $C_{21}H_{22}F_2O$ $[M]^+$: 328.1633, found: 328.1635.

4s

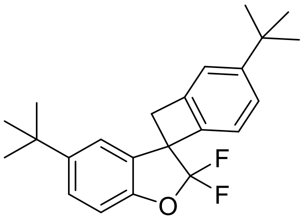

Chemical Formula: $C_{23}H_{26}F_2O$
Exact Mass: 356.1952

This compound was obtained following the general procedure C. Starting from 4-*tert*-butyl-2-iodophenol **1d** (55.2 mg, 0.2 mmol), ammonium salt **2e** (91.4 mg, 0.2 mmol), $PdBr_2$ (2.7 mg, 0.01 mmol), **L1** (7.2 mg, 0.02 mmol), Cs_2CO_3 (130 mg, 0.4 mmol, 2.0 equiv), and toluene (2.5 mL). Purification on basic aluminum oxide (Pentane/Ethyl acetate gradient from 98/2 to 95/5) afforded **4s** (44 mg, 62%) as a colorless oil.

^{1}H NMR (300 MHz, $CDCl_3$) δ 7.41 – 7.25 (m, 3H), 7.10 – 7.01 (m, 2H), 6.97 – 6.88 (m, 1H), 3.99 (d, *J* = 14.0 Hz, 1H), 3.33 (dd, *J* = 14.9, 3.3 Hz, 1H), 1.37 (s, 10H), 1.26 (s, 10H).

^{13}C NMR (75 MHz, $CDCl_3$) δ 152.9, 152.5 (d, *J* = 3.0 Hz), 147.0, 142.6, 139.2 (dd, *J* = 7.9, 1.9 Hz), 132.7 (t, *J* = 264.8 Hz), 129.0 (d, *J* = 2.3 Hz), 126.6, 125.5, 122.8, 120.2, 120.1, 109.6, 58.9 (t, *J* = 29.3 Hz), 41.7 (dd, *J* = 9.5, 4.7 Hz), 35.5, 34.8, 31.8 (2C).

^{19}F NMR (282 MHz, $CDCl_3$) δ -69.9 (dd, *J* = 148.2, 3.1 Hz, 1F), -80.0 (d, *J* = 148.6 Hz, 1F).

HRMS (EI): calculated for $C_{23}H_{26}F_2O$ $[M]^+$: 356.1946, found: 356.1949.

4t

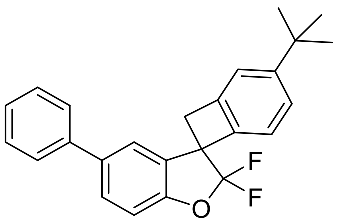

Chemical Formula: $C_{25}H_{22}F_2O$
Exact Mass: 376.1639

This compound was obtained following the general procedure C. Starting from 3-iodo-[1,1'-biphenyl]-4-ol **1e** (59.2 mg, 0.2 mmol), ammonium salt **2e** (91.4 mg, 0.2 mmol), $PdBr_2$ (2.7 mg,

0.01 mmol), **L1** (7.2 mg, 0.02 mmol), Cs_2CO_3 (130 mg, 0.4 mmol, 2.0 equiv), and toluene (2.5 mL). Purification on basic aluminum oxide (Pentane/Ethyl acetate gradient from 98/2 to 95/5) afforded **4t** (58 mg, 76%) as a white solid.

^{1}H NMR (300 MHz, $CDCl_3$) δ 7.52 – 7.43 (m, 3H), 7.41 – 7.23 (m, 6H), 7.13 – 7.01 (m, 2H), 4.04 (d, *J* = 14.3 Hz, 1H), 3.38 (dd, *J* = 14.2, 2.2 Hz, 1H), 1.36 (s, 9H).

^{13}C NMR (75 MHz, $CDCl_3$) δ 154.1 (d, *J* = 2.8 Hz), 153.1, 142.5, 140.7, 139.0 (d, *J* = 5.8 Hz), 137.4, 132.6 (t, *J* = 264.8 Hz), 130.3 (d, *J* = 2.5 Hz), 128.9, 128.6, 127.3, 127.1, 125.6, 122.7, 122.3, 120.2, 110.5, 58.6 (t, *J* = 29.3 Hz), 41.7 (dd, *J* = 9.4, 4.6 Hz), 35.5, 31.7.

^{19}F NMR (282 MHz, $CDCl_3$) δ -70.0 (dd, *J* = 148.4, 3.0 Hz, 1F), -79.4 (d, *J* = 148.2 Hz, 1F).

HRMS (EI): calculated for $C_{25}H_{22}F_2O$ $[M]^+$: 376.1633, found: 376.1631.

4u

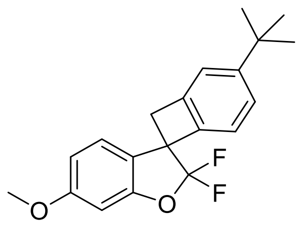

Chemical Formula: $C_{20}H_{20}F_2O_2$
Exact Mass: 330.1431

This compound was obtained following the general procedure C. Starting from 2-iodo-5-methoxyphenol **1f** (50.0 mg, 0.2 mmol), ammonium salt **2e** (91.4 mg, 0.2 mmol), $PdBr_2$ (2.7 mg, 0.01 mmol), **L1** (7.2 mg, 0.02 mmol), Cs_2CO_3 (130 mg, 0.4 mmol, 2.0 equiv), and toluene (2.5 mL). Purification on basic aluminum oxide (Pentane/Ethyl acetate gradient from 98/2 to 95/5) afforded **4u** (40 mg, 61%) as a colorless oil.

^{1}H NMR (300 MHz, $CDCl_3$) δ 7.38 – 7.29 (m, 1H), 7.28 – 7.22 (m, 1H), 7.01 (d, *J* = 7.7 Hz, 1H), 6.95 (d, *J* = 8.3 Hz, 1H), 6.63 – 6.51 (m, 2H), 3.98 (d, *J* = 14.0 Hz, 1H), 3.80 (s, 3H), 3.34 – 3.22 (m, 1H), 1.35 (s, 9H).

^{13}C NMR (75 MHz, $CDCl_3$) δ 161.3, 155.6 (d, *J* = 3.2 Hz), 153.0, 142.5, 132.7 (t, *J* = 264.8 Hz), 125.4, 123.9, 122.6, 121.4, 120.1, 109.4, 96.9, 58.6 (t, *J* = 29.3 Hz), 55.8, 41.7 (dd, *J* = 9.2, 4.5 Hz), 35.4, 31.7.

^{19}F NMR (282 MHz, $CDCl_3$) δ -70.1 (dd, *J* = 148.1, 3.1 Hz, 1F), -78.9 (d, *J* = 148.1 Hz, 1F).

HRMS (EI): calculated for $C_{20}H_{20}F_2O_2$ $[M]^+$: 330.1426, found: 330.1420.

4v

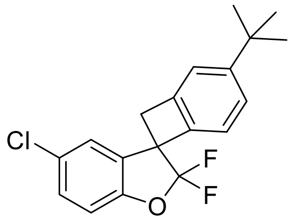

Chemical Formula: $C_{19}H_{17}ClF_2O$
Exact Mass: 334.0936

This compound was obtained following the general procedure C. Starting from 4-chloro-2-iodophenol **1g** (51 mg, 0.2 mmol), ammonium salt **2e** (91.4 mg, 0.2 mmol), $PdBr_2$ (1.0 mg, 0.004 mmol), **L1** (2.9 mg, 0.008 mmol), Cs_2CO_3 (130 mg, 0.4 mmol, 2.0 equiv), and toluene (2.5 mL). Purification on basic aluminum oxide (Pentane/Ethyl acetate gradient from 98/2 to 95/5) afforded **4v** (48 mg, 74%) as a white solid.

^{1}H NMR (300 MHz, $CDCl_3$) δ 7.43 – 7.32 (m, 1H), 7.32 – 7.24 (m, 2H), 7.29 – 7.18 (m, 2H), 7.10 – 6.98 (m, 2H), 6.99 – 6.89 (m, 1H), 4.02 (d, *J* = 14.1 Hz, 1H), 3.32 (dd, *J* = 14.5, 2.7 Hz, 1H), 1.36 (s, 10H).

^{13}C NMR (75 MHz, $CDCl_3$) δ 153.4, 153.0 (d, *J* = 3.2 Hz), 142.3, 138.3 (dd, *J* = 7.3, 1.7 Hz), 132.4 (t, *J* = 264.8 Hz), 131.5 (d, *J* = 2.5 Hz), 129.6, 128.8, 125.7, 123.7, 122.7, 120.2, 111.5, 58.5 (t, *J* = 29.3 Hz), 41.5 (dd, *J* = 9.2, 4.4 Hz), 35.5, 31.7.

^{19}F NMR (282 MHz, $CDCl_3$) δ -70.0 (dd, *J* = 147.8, 2.9 Hz, 1F), -79.2 (d, *J* = 147.6 Hz, 1F).

HRMS (EI): calculated for $C_{19}H_{17}ClF_2O$ $[M]^+$: 334.0931, found: 334.0925.

4w

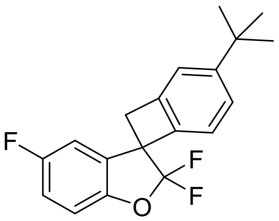

Chemical Formula: $C_{19}H_{17}F_3O$
Exact Mass: 318.1231

This compound was obtained following the general procedure C. Starting from 4-fluoro-2-iodophenol **1h** (47.6 mg, 0.2 mmol), ammonium salt **2e** (91.4 mg, 0.2 mmol), $PdBr_2$ (2.7 mg, 0.01 mmol), **L1** (7.2 mg, 0.02 mmol), Cs_2CO_3 (130 mg, 0.4 mmol, 2.0 equiv), and toluene (2.5 mL). Purification on basic aluminum oxide (Pentane/Ethyl acetate gradient from 98/2 to 95/5) afforded **4w** (52 mg, 82%) as a colorless oil.

^{1}H NMR (300 MHz, $CDCl_3$) δ 7.41 – 7.31 (m, 1H), 7.30 – 7.26 (m, 1H), 7.08 – 6.98 (m, 1H), 7.00 – 6.90 (m, 2H), 6.82 – 6.72 (m, 1H), 4.02 (d, *J* = 13.8 Hz, 1H), 3.37 – 3.25 (m, 1H), 1.35 (s, 9H).

^{13}C NMR (75 MHz, $CDCl_3$) δ 159.3 (d, J = 241.5 Hz), 153.4, 150.7 – 150.1 (m), 142.3, 138.5 (dd, J = 7.3, 1.6 Hz), 132.5 (t, J = 264.8 Hz), 131.3 (d, J = 2.2 Hz), 125.7, 122.6, 120.2, 116.1 (d, J = 24.6 Hz), 111.1 (d, J = 8.4 Hz), 110.8 (d, J = 25.3 Hz), 58.8 (t, J = 29.3 Hz), 41.5 (dd, J = 9.2, 4.5 Hz), 35.5, 31.7.

^{19}F NMR (282 MHz, $CDCl_3$) δ -70.0 (dd, J = 147.9, 3.0 Hz, 1F), -79.0 (d, J = 148.1 Hz, 1F), -119.2 – -119.6 (m, 1F).

HRMS (EI): calculated for $C_{19}H_{17}F_3O$ $[M]^+$: 318.1226, found: 318.1220.

4x

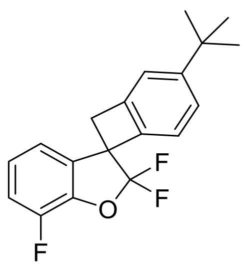

Chemical Formula: $C_{19}H_{17}F_3O$
Exact Mass: 318.1231

This compound was obtained following the general procedure C. Starting from 2-fluoro-6-iodophenol **1i** (47.6 mg, 0.2 mmol), ammonium salt **2e** (91.4 mg, 0.2 mmol), $PdBr_2$ (2.7 mg, 0.01 mmol), **L1** (7.2 mg, 0.02 mmol), Cs_2CO_3 (130 mg, 0.4 mmol, 2.0 equiv), and toluene (2.5 mL). Purification on basic aluminum oxide (Pentane/Ethyl acetate gradient from 98/2 to 95/5) afforded **4x** (44 mg, 69%) as a colorless oil.

^{1}H NMR (300 MHz, $CDCl_3$) δ 7.39 – 7.32 (m, 1H), 7.30 – 7.27 (m, 1H), 7.11 – 6.92 (m, 3H), 6.84 (dd, J = 7.4, 1.3 Hz, 1H), 4.03 (d, J = 14.0 Hz, 1H), 3.38 – 3.28 (m, 1H), 1.35 (s, 9H).

^{13}C NMR (75 MHz, $CDCl_3$) δ 153.4, 148.4, 145.1, 142.3, 138.6 – 138.4 (m), 133.0 – 132.8 (m), 132.6 (t, J = 267 Hz), 125.7, 124.29 (d, J = 5.7 Hz), 122.6, 120.2, 118.8 (d, J = 3.7 Hz), 117.0 (d, J = 16.8 Hz), 58.6 (t, J = 44.3 Hz), 41.6 (dd, J = 9.1, 4.4 Hz), 35.5, 31.7.

^{19}F NMR (282 MHz, $CDCl_3$) δ -69.8 (dd, J = 146.8, 3.0 Hz, 1F), -79.0 (d, J = 146.6 Hz, 1F), -136.9 (dd, J = 10.0, 4.3 Hz, 1F).

HRMS (EI): calculated for $C_{19}H_{17}F_3O$ $[M]^+$: 318.1226, found: 318.1232.

4y

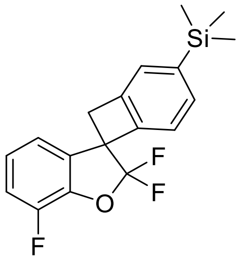

Chemical Formula: $C_{18}H_{17}F_3OSi$
Exact Mass: 334.1001

This compound was obtained following the general procedure C. Starting from 2-fluoro-6-iodophenol **1i** (47.6 mg, 0.2 mmol), ammonium salt **2s** (94.6 mg, 0.2 mmol), $PdBr_2$ (2.7 mg, 0.01 mmol), **L1** (7.2 mg, 0.02 mmol), Cs_2CO_3 (130 mg, 0.4 mmol, 2.0 equiv), and toluene (2.5 mL). Purification on basic aluminum oxide (Pentane/Ethyl acetate gradient from 98/2 to 95/5) afforded **4y** (48 mg, 72%) as a colorless oil.

^{1}H NMR (300 MHz, $CDCl_3$) δ 7.51 – 7.45 (m, 1H), 7.42 – 7.37 (m, 1H), 7.12 – 7.02 (m, 2H), 7.01 – 6.93 (m, 1H), 6.87 – 6.80 (m, 1H), 4.13 – 4.00 (m, 1H), 3.43 – 3.32 (m, 1H), 0.30 (s, 9H).

^{13}C NMR (75 MHz, $CDCl_3$) δ 148.4, 145.1, 142.9, 142.5 – 142.3 (m, 2C), 136.1, 133.1, 132.7 – 132.4 (m), 127.8, 124.4 (d, *J* = 5.6 Hz), 122.2, 118.8 (d, *J* = 3.7 Hz), 117.1 (d, *J* = 16.8 Hz), 59.2 – 58.5 (m), 41.83 (dd, *J* = 9.0, 4.3 Hz), -0.9.

^{19}F NMR (282 MHz, $CDCl_3$) δ -69.9 (dd, J = 146.8, 3.0 Hz, 1F), -78.6 (d, J = 146.7 Hz, 1F), -136.7 (dd, J = 10.0, 4.4 Hz, 1F).

HRMS (EI): calculated for $C_{18}H_{17}F_3OSi$ $[M]^+$: 334.0995, found: 334.0992.

4z

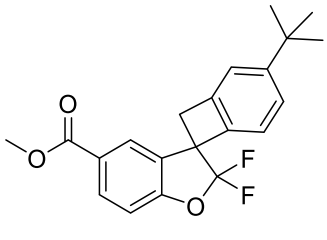

Chemical Formula: $C_{21}H_{20}F_2O_3$
Exact Mass: 358.1381

This compound was obtained following the general procedure C. Starting from methyl 4-hydroxy-3-iodobenzoate **1j** (55.6 mg, 0.2 mmol), ammonium salt **2e** (91.4 mg, 0.2 mmol), $PdBr_2$ (2.7 mg, 0.01 mmol), **L1** (7.2 mg, 0.02 mmol), Cs_2CO_3 (130 mg, 0.4 mmol, 2.0 equiv), and toluene (2.5 mL). Purification on basic aluminum oxide (Pentane/Ethyl acetate gradient from 98/2 to 95/5) afforded **4z** (54 mg, 75%) as a colorless oil.

^{1}H NMR (300 MHz, $CDCl_3$) δ 8.03 (dd, *J* = 8.5, 1.9 Hz, 1H), 7.76 (d, *J* = 1.8 Hz, 1H), 7.39 – 7.32 (m, 1H), 7.31 – 7.27 (m, 1H), 7.09 – 6.96 (m, 2H), 4.03 (d, *J* = 14.1 Hz, 1H), 3.86 (s, 3H), 3.42 –

3.29 (m, 1H), 1.36 (s, 9H).

^{13}C NMR (75 MHz, $CDCl_3$) δ 166.4, 158.0, 153.4, 142.32, 138.4 (d, *J* = 7.4 Hz), 132.6 (d, *J* = 267.0 Hz), 132.2, 130.2 (d, *J* = 2.4 Hz), 125.7, 125.4, 122.6, 120.2, 110.2, 58.0 (d, *J* = 30.0 Hz), 52.3, 41.6 (dd, J = 9.3, 4.3 Hz), 35.5, 31.7.

^{19}F NMR (282 MHz, $CDCl_3$) δ -69.9 (dd, J = 147.8, 2.9 Hz, 1F), -78.7 (d, J = 147.8 Hz, 1F).

HRMS (EI): calculated for $C_{21}H_{20}F_2O_3$ $[M]^+$: 358.1375, found: 358.1373.

4aa

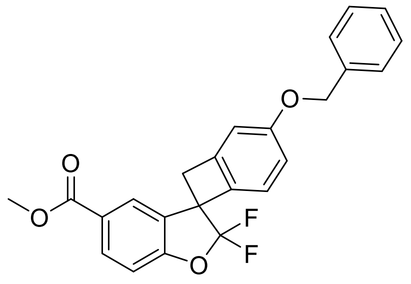

Chemical Formula: $C_{24}H_{18}F_2O_4$
Exact Mass: 408.1173

This compound was obtained following the general procedure C. Starting from methyl 4-hydroxy-3-iodobenzoate **1j** (55.6 mg, 0.2 mmol), ammonium salt **2o** (101.4 mg, 0.2 mmol), $PdBr_2$ (2.7 mg, 0.01 mmol), **L1** (7.2 mg, 0.02 mmol), Cs_2CO_3 (130 mg, 0.4 mmol, 2.0 equiv), and toluene (2.5 mL). Purification on basic aluminum oxide (Pentane/Ethyl acetate gradient from 98/2 to 95/5) afforded **4aa** (56 mg, 69%) as a colorless oil.

^{1}H NMR (300 MHz, $CDCl_3$) δ 8.03 (dd, *J* = 8.5, 1.8 Hz, 1H), 7.75 (dd, *J* = 1.8, 0.5 Hz, 1H), 7.50 – 7.31 (m, 5H), 7.09 – 6.85 (m, 4H), 5.10 (s, 2H), 4.00 (d, *J* = 14.2 Hz, 1H), 3.86 (s, 3H), 3.38 – 3.23 (m, 1H).

^{13}C NMR (75 MHz, $CDCl_3$) δ 166.3, 160.8, 157.9, 143.6, 137.0, 133.4 – 133.2 (m), 132.4 (d, *J* = 267.0 Hz), 132.2, 130.3 – 129.9 (m), 128.8, 128.2, 127.6, 126.1, 125.3, 124.6, 116.4, 110.2, 109.8, 70.5, 57.6 (t, *J* = 28.5 Hz), 52.3, 41.3 (dd, J = 9.8, 4.5 Hz).

^{19}F NMR (282 MHz, $CDCl_3$) δ -70.1 (dd, *J* = 148.1, 3.0 Hz, 1F), -79.3 (d, *J* = 147.9 Hz, 1F).

HRMS (EI): calculated for $C_{24}H_{18}F_2O_4$ $[M]^+$: 408.1168, found: 408.1162.

4ab

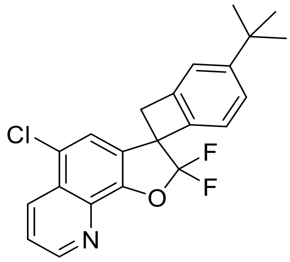

Chemical Formula: $C_{22}H_{18}ClF_2NO$
Exact Mass: 385.1045

This compound was obtained following the general procedure C. Starting from 5-chloro-7-iodoquinolin-8-ol **1k** (61.0 mg, 0.2 mmol), ammonium salt **2e** (91.4 mg, 0.2 mmol), $PdBr_2$ (5.4 mg, 0.02 mmol), **L1** (14.4 mg, 0.04 mmol), Cs_2CO_3 (130 mg, 0.4 mmol, 2.0 equiv), and toluene (2.5 mL). Purification on basic aluminum oxide (Pentane/Ethyl acetate gradient from 98/2 to 90/10) afforded **4ab** (46 mg, 60%) as a slight yellow solid.

^{1}H NMR (300 MHz, $CDCl_3$) δ 9.01 (dd, *J* = 4.2, 1.6 Hz, 1H), 8.54 (dd, *J* = 8.7, 1.6 Hz, 1H), 7.56 (dd, *J* = 8.7, 4.2 Hz, 1H), 7.40 – 7.34 (m, 1H), 7.33 – 7.27 (m, 2H), 7.04 (d, *J* = 8.0 Hz, 1H), 4.11 (d, *J* = 14.2 Hz, 1H), 3.42 (dd, *J* = 14.1, 2.0 Hz, 1H), 1.36 (s, 9H).

^{13}C NMR (75 MHz, $CDCl_3$) δ 153.6, 151.6, 148.9 (dd, *J* = 2.8, 1.2 Hz), 142.2, 138.2 (dd, *J* = 7.8, 2.1 Hz), 135.5, 133.6, 132.5, 127.8 (d, *J* = 2.4 Hz), 127.3, 126.1, 125.8, 122.8, 122.7, 121.1, 120.3, 59.4 (t, *J* = 30.1 Hz), 40.89 (dd, *J* = 9.6, 4.4 Hz), 35.5, 31.7.

^{19}F NMR (282 MHz, $CDCl_3$) δ -69.5 (dd, *J* = 147.6, 2.9 Hz, 1F), -77.9 (d, *J* = 147.9 Hz, 1F).

HRMS (ESI): calculated for $C_{22}H_{19}ClF_2NO$ $[M+H]^+$: 386.1123, found: 386.1119.

4ac

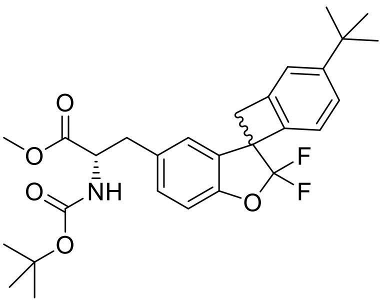

Chemical Formula: $C_{28}H_{33}F_2NO_5$
Exact Mass: 501.2327

This compound was obtained following the general procedure C. Starting from methyl (*S*)-2-((*tert*-butoxycarbonyl)amino)-3-(4-hydroxy-3-iodophenyl)propanoate **1l** (84.1 mg, 0.2 mmol), ammonium salt **2e** (91.4 mg, 0.2 mmol), $PdBr_2$ (5.4 mg, 0.02 mmol), **L1** (14.4 mg, 0.04 mmol), Cs_2CO_3 (130 mg, 0.4 mmol, 2.0 equiv), and toluene (2.5 mL). Purification on basic aluminum oxide (Pentane/Ethyl acetate gradient from 98/2 to 90/10) afforded **4ac** (46 mg, 46% yield, dr

= 1.1/1) as a white solid.

^{1}H NMR (300 MHz, $CDCl_3$) δ 7.37 – 7.31 (m, 1H), 7.28 – 7.24 (m, 1H), 7.06 – 6.96 (m, 2H), 6.95 – 6.87 (m, 1H), 6.80 – 6.70 (m, 1H), 4.95 (d, *J* = 8.2 Hz, 1H), 4.58 – 4.38 (m, 1H), 4.02 (d, *J* = 5.6 Hz, 1H, minor), 3.97 (d, *J* = 5.6 Hz, 1H, major), 3.65 (s, 3H, major), 3.55 (s, 3H, minor), 3.34 – 3.20 (m, 1H), 3.10 – 2.91 (m, 2H), 1.39 (s, 9H, minor), 1.35 (s, 9H), 1.34 (s, 9H, major).

^{13}C NMR (75 MHz, $CDCl_3$) δ 172.3, 172.1, 155.0, 153.6, 153.1, 142.4, 139.0, 131.5, 130.5, 130.0, 125.5, 124.3, 122.6, 120.1, 110.2, 80.1, 58.4 (t, *J* = 30.1 Hz), 54.6, 52.3, 41.8 – 41.4 (m), 37.9, 35.4, 31.7, 28.3.

^{19}F NMR (282 MHz, $CDCl_3$) δ -69.6 (s, 1F, minor), -70.2 (s, 1F, major), -79.1 (d, *J* = 127.6 Hz, 1F, major), -79.6 (d, *J* = 127.3 Hz, 1F, minor).

HRMS (ESI): calculated for $C_{28}H_{33}F_2NO_5Na$ $[M+Na]^+$: 524.2224, found: 524.2225.

4ad

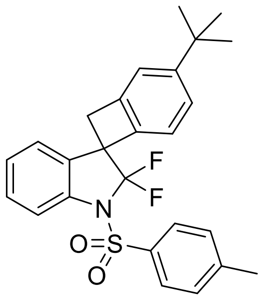

Chemical Formula: $C_{26}H_{25}F_2NO_2S$
Exact Mass: 453.1574

This compound was obtained following the general procedure C. Starting from *N*-(2-iodophenyl)-4-methylbenzenesulfonamide **1o** (74.6 mg, 0.2 mmol), ammonium salt **2e** (91.4 mg, 0.2 mmol), $PdBr_2$ (5.4 mg, 0.02 mmol), **L1** (14.4 mg, 0.04 mmol), Cs_2CO_3 (130 mg, 0.4 mmol, 2.0 equiv), and toluene (2.5 mL). Purification on basic aluminum oxide (Pentane/Ethyl acetate gradient from 98/2 to 90/10) afforded **4ad** (45 mg, 50%) as a white solid.

^{1}H NMR (300 MHz, $CDCl_3$) δ 7.96 (d, *J* = 8.3 Hz, 2H), 7.62 – 7.53 (m, 1H), 7.36 – 7.21 (m, 5H), 7.06 – 6.91 (m, 2H), 6.84 (d, *J* = 8.1 Hz, 1H), 3.96 (d, *J* = 14.0 Hz, 1H), 3.20 – 3.09 (m, 1H), 2.42 (s, 3H), 1.33 (s, 9H).

^{13}C NMR (75 MHz, $CDCl_3$) δ 153.1, 145.0, 142.4, 138.8 (d, *J* = 10.3 Hz), 138.6, 135.9, 130.2 (d, *J* = 2.7 Hz), 129.9, 129.3, 128.3, 126.6, 125.5, 124.6, 123.3, 122.6, 120.1, 113.0, 58.5 (t, *J* = 29.3 Hz), 41.9 (dd, *J* = 10.5, 3.8 Hz), 35.5, 31.7, 21.8.

^{19}F NMR (282 MHz, $CDCl_3$) δ -74.9 (dd, *J* = 187.5, 2.7 Hz, 1F), -83.9 (d, *J* = 187.2 Hz, 1F).

HRMS (EI): calculated for $C_{26}H_{25}F_2NO_2S$ $[M]^+$: 453.1569, found: 453.1568.

4ae

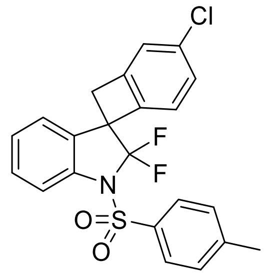

Chemical Formula: $C_{22}H_{16}ClF_2NO_2S$
Exact Mass: 431.0558

This compound was obtained following the general procedure C. Starting from *N*-(2-iodophenyl)-4-methylbenzenesulfonamide **1o** (74.6 mg, 0.2 mmol), ammonium salt **2e** (87 mg, 0.2 mmol), $PdBr_2$ (2.7 mg, 0.01 mmol), **L1** (7.2 mg, 0.02 mmol), Cs_2CO_3 (130 mg, 0.4 mmol, 2.0 equiv), and toluene (2.5 mL). Purification on basic aluminum oxide (Pentane/Ethyl acetate gradient from 98/2 to 90/10) afforded **4ae** (50 mg, 58%) as a white solid.

^{1}H NMR (300 MHz, $CDCl_3$) δ 7.95 (d, *J* = 8.2 Hz, 2H), 7.63 – 7.52 (m, 1H), 7.37 – 7.17 (m, 5H), 7.07 – 6.97 (m, 1H), 6.89 – 6.81 (m, 1H), 7.07 – 6.97 (m, 1H), 3.97 (d, *J* = 14.7 Hz, 1H), 3.25 – 3.08 (m, 1H), 2.42 (s, 3H).

^{13}C NMR (75 MHz, $CDCl_3$) δ 145.2, 144.1, 140.3 – 140.1 (m), 138.7, 135.8, 135.7, 129.9, 129.7, 129.3 – 129.1 (m), 129.0, 128.3, 128.2, 124.8, 123.9, 123.1, 113.1, 59.8 (t, *J* = 27.0 Hz), 41.7 (dd, *J* = 11.3, 4.5 Hz), 21.8.

^{19}F NMR (282 MHz, $CDCl_3$) δ -75.2 (dd, *J* = 187.6, 2.7 Hz, 1F), -83.7 (d, *J* = 187.1 Hz, 1F).

HRMS (ESI): calculated for $C_{22}H_{16}ClF_2NO_2SNa$ $[M+Na]^+$: 454.0455, found: 454.0452.

Compound 6

6a

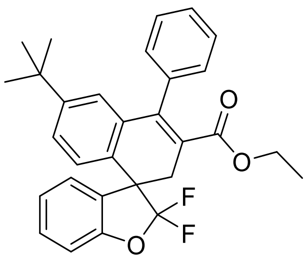

Chemical Formula: $C_{30}H_{28}F_2O_3$
Exact Mass: 474.2007

This compound was obtained following the general procedure D. Starting from 2-iodophenol **1a** (44.0 mg, 0.2 mmol, 1 equiv), ammonium salt **2e** (91.4 mg, 0.2 mmol), alkyne **5a** (66 µL, 0.4

mmol, 2 equiv), $PdBr_2$ (2.7 mg, 0.01 mmol), **L3** (7.7 mg, 0.02 mmol), Cs_2CO_3 (130 mg, 0.4 mmol, 2.0 equiv), and toluene (1.0 mL). Purification on basic aluminum oxide (Pentane/Ethyl acetate gradient from 99/1 to 95/5) afforded **6a** (81 mg, 85%) as a slight yellow solid.

^{1}H NMR (300 MHz, $CDCl_3$) δ 7.48 – 7.32 (m, 4H), 7.30 – 7.12 (m, 5H), 7.09 – 7.01 (m, 1H), 6.90 (d, *J* = 2.1 Hz, 1H), 6.64 (d, *J* = 8.1 Hz, 1H), 4.05 – 3.85 (m, 2H), 3.37 – 3.11 (m, 2H), 1.12 (s, 9H), 0.89 (t, *J* = 7.1 Hz, 3H).

^{13}C NMR (75 MHz, $CDCl_3$) δ 168.1, 151.5, 145.9, 139.0, 136.1, 130.8 (d, *J* = 1.6 Hz), 130.5, 130.0, 128.9, 128.1, 127.5, 127.0, 126.3, 126.2, 124.8, 124.3, 123.9, 110.5, 60.5, 53.5 (t, *J* = 24.5 Hz), 34.7, 33.1 (d, *J* = 7.7 Hz), 31.1, 13.7.

^{19}F NMR (282 MHz, $CDCl_3$) δ -72.9 (dd, *J* = 142.1, 2.7 Hz, 1F), -73.9 (d, *J* = 142.1 Hz, 1F).

HRMS (EI): calculated for $C_{30}H_{28}F_2O_3$ $[M]^+$: 474.2001, found: 474.2000.

6b

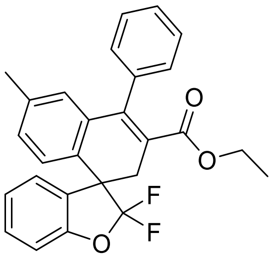

Chemical Formula: $C_{27}H_{22}F_2O_3$
Exact Mass: 432.1537

This compound was obtained following the general procedure D. Starting from 2-iodophenol **1a** (44.0 mg, 0.2 mmol, 1 equiv), ammonium salt **2l** (83 mg, 0.2 mmol), alkyne **5a** (66 µL, 0.4 mmol, 2 equiv), $PdBr_2$ (2.7 mg, 0.01 mmol), **L3** (7.7 mg, 0.02 mmol), Cs_2CO_3 (130 mg, 0.4 mmol, 2.0 equiv), and toluene (1.0 mL). Purification on basic aluminum oxide (Pentane/Ethyl acetate gradient from 99/1 to 95/5) afforded **6b** (61 mg, 70%) as a colorless oil.

^{1}H NMR (300 MHz, $CDCl_3$) δ 7.48 – 7.34 (m, 4H), 7.29 – 7.16 (m, 4H), 7.10 – 6.96 (m, 2H), 6.74 – 6.68 (m, 1H), 6.63 (d, *J* = 7.8 Hz, 1H), 4.04 – 3.85 (m, 2H), 3.38 – 3.12 (m, 2H), 2.20 (s, 3H), 0.89 (t, *J* = 7.1 Hz, 3H).

^{13}C NMR (75 MHz, $CDCl_3$) δ 168.0, 155.0 – 154.9 (m), 145.4, 138.9, 138.5, 136.3, 131.1 (dd, *J* = 275.3, 266.3 Hz), 130.9 – 130.7 (m), 130.4, 130.1, 130.0, 129.6, 128.9, 128.2, 127.5, 127.2, 124.8, 124.8, 123.9, 110.5, 60.5, 53.5 (t, *J* = 24.7 Hz), 33.20 (d, *J* = 7.9 Hz), 21.3, 13.7.

^{19}F NMR (282 MHz, $CDCl_3$) δ -72.6 (dd, *J* = 142.5, 2.6 Hz, 1F), -73.7 (d, *J* = 142.5 Hz, 1F).

HRMS (EI): calculated for $C_{27}H_{22}F_2O_3$ $[M]^+$: 432.1532, found: 432.1523.

6c

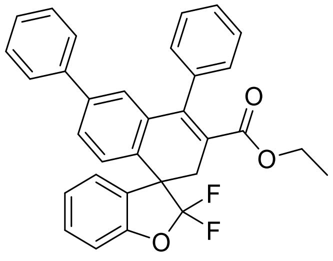

Chemical Formula: $C_{32}H_{24}F_2O_3$
Exact Mass: 494.1694

This compound was obtained following the general procedure D. Starting from 2-iodophenol **1a** (44.0 mg, 0.2 mmol, 1 equiv), ammonium salt **2k** (95.4 mg, 0.2 mmol), alkyne **5a** (66 µL, 0.4 mmol, 2 equiv), $PdBr_2$ (2.7 mg, 0.01 mmol), **L3** (7.7 mg, 0.02 mmol), Cs_2CO_3 (130 mg, 0.4 mmol, 2.0 equiv), and toluene (1.0 mL). Purification on basic aluminum oxide (Pentane/Ethyl acetate gradient from 99/1 to 95/5) afforded **6c** (69 mg, 70%) as a colorless oil.

^{1}H NMR (300 MHz, $CDCl_3$) δ 7.47 – 7.39 (m, 5H), 7.37 – 7.27 (m, 7H), 7.26 – 7.18 (m, 2H), 7.15 – 7.07 (m, 2H), 6.84 – 6.78 (m, 1H), 4.03 – 3.90 (m, 2H), 3.31 (qd, *J* = 17.0, 1.7 Hz, 2H), 0.90 (t, *J* = 7.1 Hz, 3H).

^{13}C NMR (75 MHz, $CDCl_3$) δ 168.0, 155.0 – 154.9 (m), 145.4, 138.9, 138.5, 136.3, 131.1 (dd, *J* = 275.3, 266.3 Hz), 130.9 – 130.7 (m), 130.4, 130.1, 130.0, 129.6, 128.9, 128.2, 127.5, 127.2, 124.8, 124.8, 123.9, 110.5, 60.5, 53.49 (t, *J* = 24.7 Hz), 33.20 (d, *J* = 7.9 Hz), 21.3, 13.7.

^{13}C NMR (75 MHz, $CDCl_3$) δ 167.9, 155.2 – 154.8 (m), 145.3, 141.6, 140.3, 138.7, 137.0, 132.8 – 132.5 (m), 130.2, 130.2, 129.0, 128.9, 128.3, 128.0, 127.8, 127.7, 127.7, 127.1, 125.2, 124.8, 124.0, 110.6, 60.6, 53.6 (t, *J* = 24.7 Hz), 33.2 (d, *J* = 8.3 Hz), 13.7.

^{19}F NMR (282 MHz, $CDCl_3$) δ -72.3 (dd, *J* = 142.5, 2.6 Hz, 1F), -73.5 (d, *J* = 142.5 Hz), 1F).

HRMS (EI): calculated for $C_{32}H_{24}F_2O_3$ $[M]^+$: 494.1688, found: 494.1687.

6d

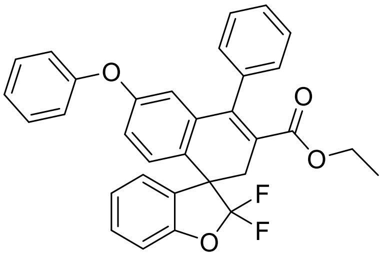

Chemical Formula: $C_{32}H_{24}F_2O_4$
Exact Mass: 510.1643

This compound was obtained following the general procedure D. Starting from 2-iodophenol **1a** (44.0 mg, 0.2 mmol, 1 equiv), ammonium salt **2n** (98.6 mg, 0.2 mmol), alkyne **5a** (66 µL, 0.4 mmol, 2 equiv), $PdBr_2$ (1.0 mg, 0.004 mmol), **L3** (3.1 mg, 0.008 mmol), Cs_2CO_3 (130 mg, 0.4

mmol, 2.0 equiv), and toluene (1.0 mL). Purification on basic aluminum oxide (Pentane/Ethyl acetate gradient from 99/1 to 95/5) afforded **6d** (68 mg, 67%) as a white solid.

^{1}H NMR (300 MHz, $CDCl_3$) δ 7.42 – 7.34 (m, 2H), 7.32 – 7.24 (m, 2H), 7.23 – 7.15 (m, 1H), 7.09 – 7.01 (m, 1H), 6.98 – 6.85 (m, 1H), 6.82 – 6.58 (m, 1H), 4.03 – 3.86 (m, 2H), 3.27 (qd, *J* = 17.1, 1.9 Hz, 1H), 0.88 (t, *J* = 7.1 Hz, 1H).

^{13}C NMR (75 MHz, $CDCl_3$) δ 167.8, 157.7, 156.3, 154.9, 144.8, 144.6 – 144.3 (m), 138.4, 138.3, 130.2, 130.1, 129.8, 128.8, 128.6, 128.3, 127.7, 125.6, 124.7, 124.0, 123.8, 119.4, 119.2, 118.2, 110.6, 60.6, 53.4 (t, *J* = 24.7 Hz), 33.2 (d, *J* = 8.2 Hz), 13.7.

^{19}F NMR (282 MHz, $CDCl_3$) δ -72.7 (dd, *J* = 142.3, 2.7 Hz, 1F), -73.9 (d, *J* = 142.2 Hz, 1F).

HRMS (EI): calculated for $C_{32}H_{24}F_2O_4$ $[M]^+$: 494.510.1637, found: 510.1643.

6e

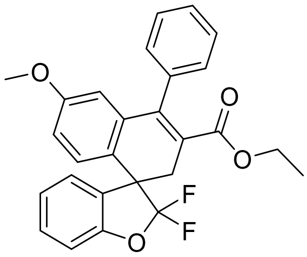

Chemical Formula: $C_{27}H_{22}F_2O_4$
Exact Mass: 448.1486

This compound was obtained following the general procedure D. Starting from 2-iodophenol **1a** (44.0 mg, 0.2 mmol, 1 equiv), ammonium salt **2m** (86.2 mg, 0.2 mmol), alkyne **5a** (66 µL, 0.4 mmol, 2 equiv), $PdBr_2$ (2.7 mg, 0.01 mmol), **L3** (7.7 mg, 0.02 mmol), Cs_2CO_3 (130 mg, 0.4 mmol, 2.0 equiv), and toluene (1.0 mL). Purification on basic aluminum oxide (Pentane/Ethyl acetate gradient from 99/1 to 95/5) afforded **6e** (70 mg, 78%) as a colorless oil.

^{1}H NMR (300 MHz, $CDCl_3$) δ 7.47 – 7.32 (m, 4H), 7.29 – 7.14 (m, 4H), 7.09 – 7.02 (m, 1H), 6.76 – 6.62 (m, 2H), 6.46 (d, *J* = 2.5 Hz, 1H), 4.04 – 3.85 (m, 2H), 3.63 (s, 3H), 3.36 – 3.13 (m, 2H), 0.88 (t, *J* = 7.1 Hz, 3H).

^{13}C NMR (75 MHz, $CDCl_3$) δ 167.9, 159.6, 155.0 – 154.8 (m), 145.1, 138.7, 137.8, 133.1 (dd, *J* = 275.3, 266.3 Hz), 130.4, 130.0, 128.9, 128.5, 128.2, 127.6, 125.9 – 125.6 (m), 125.4, 124.7, 123.9, 115.5, 113.6, 110.5, 60.5, 55.3, 53.3 (t, *J* = 24.7 Hz), 33.3 (d, *J* = 8.0 Hz), 13.6.

^{19}F NMR (282 MHz, $CDCl_3$) δ -72.7 (dd, *J* = 142.3, 2.9 Hz, 1F), -74.1 (d, *J* = 142.4 Hz, 1F).

HRMS (ESI): calculated for $C_{27}H_{22}F_2O_4Na$ $[M+Na]^+$: 471.1383, found: 471.1383.

6f

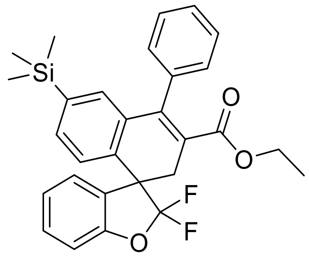

Chemical Formula: $C_{29}H_{28}F_2O_3Si$
Exact Mass: 490.1776

This compound was obtained following the general procedure D. Starting from 2-iodophenol **1a** (44.0 mg, 0.2 mmol, 1 equiv), ammonium salt **2s** (94.6 mg, 0.2 mmol), alkyne **5a** (66 µL, 0.4 mmol, 2 equiv), $PdBr_2$ (2.7 mg, 0.01 mmol), **L3** (7.7 mg, 0.02 mmol), Cs_2CO_3 (130 mg, 0.4 mmol, 2.0 equiv), and toluene (1.0 mL). Purification on basic aluminum oxide (Pentane/Ethyl acetate gradient from 99/1 to 95/5) afforded **6f** (80 mg, 82%) as a white solid.

^{1}H NMR (300 MHz, $CDCl_3$) δ 7.47 – 7.30 (m, 5H), 7.29 – 7.15 (m, 4H), 7.10 – 7.03 (m, 1H), 7.02 – 6.98 (m, 1H), 6.70 (dd, *J* = 7.5, 0.6 Hz, 1H), 4.04 – 3.85 (m, 2H), 3.42 – 3.13 (m, 2H), 0.89 (t, *J* = 7.1 Hz, 3H), 0.09 (s, 9H).

^{13}C NMR (75 MHz, $CDCl_3$) δ 168.0, 154.0, 145.7, 141.2, 138.8, 135.6, 134.5, 134.2 – 134.1 (m), 133.8, 130.3, 130.1, 129.0, 128.9, 128.1, 127.6, 126.5, 124.8, 124.4, 124.0, 110.6, 60.5, 53.7 (t, *J* = 24.7 Hz), 33.0 (d, *J* = 7.7 Hz), 13.7, -1.3.

^{19}F NMR (282 MHz, $CDCl_3$) δ -72.5 (dd, *J* = 142.5, 2.8 Hz, 1F), -73.5 (d, *J* = 142.1 Hz, 1F).

HRMS (ESI): calculated for $C_{29}H_{28}F_2O_3SiNa$ $[M+Na]^+$: 513.1673, found: 513.1673.

6g

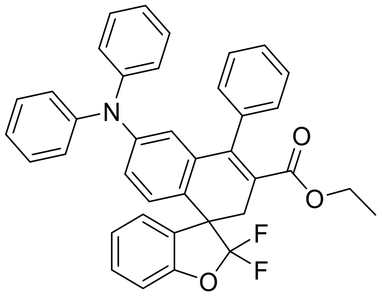

Chemical Formula: $C_{38}H_{29}F_2NO_3$
Exact Mass: 585.2116

This compound was obtained following the general procedure D. Starting from 2-iodophenol **1a** (44.0 mg, 0.2 mmol, 1 equiv), ammonium salt **2r** (114 mg, 0.2 mmol), alkyne **5a** (66 µL, 0.4 mmol, 2 equiv), $PdBr_2$ (2.7 mg, 0.01 mmol), **L3** (7.7 mg, 0.02 mmol), Cs_2CO_3 (130 mg, 0.4 mmol, 2.0 equiv), and toluene (1.0 mL). Purification on basic aluminum oxide (Pentane/Ethyl acetate gradient from 99/1 to 95/5) afforded **6g** (94 mg, 80%) as a slight yellow oil.

^{1}H NMR (300 MHz, $CDCl_3$) δ 7.40 – 7.33 (m, 1H), 7.31 – 7.14 (m, 9H), 7.11 – 6.92 (m, 9H), 6.85 – 6.77 (m, 1H), 6.61 – 6.49 (m, 2H), 4.03 – 3.85 (m, 2H), 3.37 – 3.12 (m, 2H), 0.87 (t, *J* = 7.1 Hz, 3H).

^{13}C NMR (75 MHz, $CDCl_3$) δ 168.0, 155.0 – 154.8 (m), 148.0, 146.9, 145.3, 138.5, 137.2, 130.4, 130.0, 129.3, 128.6, 128.0, 127.9 127.3, 126.6 – 126.4 (m), 124.8, 124.8, 124.7, 123.9, 123.5, 122.8, 122.3, 110.5, 60.5, 53.3 (t, *J* = 24.8 Hz), 33.2 (d, *J* = 7.6 Hz), 13.7.

^{19}F NMR (282 MHz, $CDCl_3$) δ -73.0 (dd, *J* = 142.0, 2.6 Hz, 1F), -74.1 (d, *J* = 142.1 Hz, 1F).

HRMS (ESI): calculated for $C_{38}H_{29}F_2NO_3Na$ $[M+Na]^+$: 608.2012, found: 608.2012.

6h

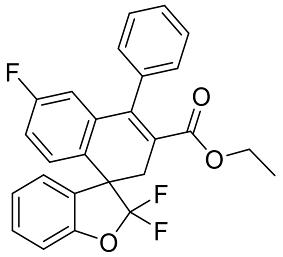

Chemical Formula: $C_{26}H_{19}F_3O_3$
Exact Mass: 436.1286

This compound was obtained following the general procedure D. Starting from 2-iodophenol **1a** (44.0 mg, 0.2 mmol, 1 equiv), ammonium salt **2p** (83.8 mg, 0.2 mmol), alkyne **5a** (66 µL, 0.4 mmol, 2 equiv), $PdBr_2$ (2.7 mg, 0.01 mmol), **L3** (7.7 mg, 0.02 mmol), Cs_2CO_3 (130 mg, 0.4 mmol, 2.0 equiv), and toluene (1.0 mL). Purification on basic aluminum oxide (Pentane/Ethyl acetate gradient from 99/1 to 95/5) afforded **6h** (46 mg, 53%) as a colorless oil.

^{1}H NMR (300 MHz, $CDCl_3$) δ 7.48 – 7.34 (m, 4H), 7.29 – 7.16 (m, 4H), 7.11 – 7.02 (m, 1H), 6.92 – 6.82 (m, 1H), 6.70 (dd, *J* = 8.6, 5.5 Hz, 1H), 6.61 (dd, *J* = 10.0, 2.7 Hz, 1H), 4.03 – 3.87 (m, 2H), 3.37 – 3.13 (m, 2H), 0.88 (t, *J* = 7.1 Hz, 3H).

^{13}C NMR (75 MHz, CDCl3) δ 167.6, 162.8 (d, *J* = 247.3 Hz), 155.1 – 154.5 (m), 144.2, 138.8 (d, *J* = 8.0 Hz), 138.2, 130.3, 129.9, 129.4 – 129.2 (m), 129.1 (d, *J* = 8.4 Hz), 128.9, 128.4, 127.9, 126.1, 124.7, 124.1, 116.0 (d, *J* = 3.5 Hz), 115.7, 110.7, 60.7, 53.3 (t, *J* = 24.8 Hz), 33.2 (d, *J* = 8.1 Hz), 13.65.

^{19}F NMR (282 MHz, $CDCl_3$) δ -72.5 (dd, *J* = 142.3, 2.8 Hz, 1F), -73.8 (d, *J* = 142.5 Hz, 1F), -112.5 – -112.8 (m).

HRMS (ESI): calculated for $C_{26}H_{19}F_3O_3Na$ $[M+Na]^+$: 459.1183, found: 459.1180.

6i

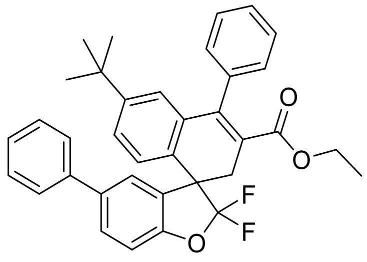

Chemical Formula: $C_{36}H_{32}F_2O_3$
Exact Mass: 550.2320

This compound was obtained following the general procedure D. Starting from 3-iodo-[1,1'-biphenyl]-4-ol **1e** (59.2 mg, 0.2 mmol, 1 equiv), ammonium salt **2e** (91.4 mg, 0.2 mmol), alkyne **5a** (66 µL, 0.4 mmol, 2 equiv), $PdBr_2$ (2.7 mg, 0.01 mmol), **L3** (7.7 mg, 0.02 mmol), Cs_2CO_3 (130 mg, 0.4 mmol, 2.0 equiv), and toluene (1.0 mL). Purification on basic aluminum oxide (Pentane/Ethyl acetate gradient from 99/1 to 95/5) afforded **6i** (88 mg, 80%) as a white solid.

^{1}H NMR (300 MHz, $CDCl_3$) δ 7.66 – 7.53 (m, 3H), 7.53 – 7.30 (m, 7H), 7.30 – 7.18 (m, 3H), 7.14 (dd, *J* = 8.4, 0.5 Hz, 1H), 6.93 (d, *J* = 2.1 Hz, 1H), 6.75 (d, *J* = 8.1 Hz, 1H), 4.06 – 3.87 (m, 2H), 3.33 (qd, *J* = 16.8, 2.0 Hz, 2H), 1.14 (s, 9H), 0.91 (t, *J* = 7.1 Hz, 3H).

^{13}C NMR (75 MHz, $CDCl_3$) δ 168.0, 154.5 (d, *J* = 2.8 Hz), 151.6, 146.0, 140.5, 139.0, 137.6, 136.1, 133.3 (dd, *J* = 275.3, 267.0 Hz), 131.2, 130.76 – 130.46 (m), 129.0, 128.9, 128.9, 128.1, 127.6, 127.5, 127.1, 127.1, 126.4, 126.3, 124.3, 123.5, 110.7, 60.5, 53.6 (t, *J* = 24.8 Hz), 34.7, 33.3 (d, *J* = 7.9 Hz), 31.1, 13.7.

^{19}F NMR (282 MHz, $CDCl_3$) δ -72.3 (dd, *J* = 141.9, 2.9 Hz, 1F), -73.8 (d, *J* = 141.6 Hz, 1F).

HRMS (EI): calculated for $C_{36}H_{32}F_2O_3$ $[M]^+$: 550.2314, found: 550.2311.

6j

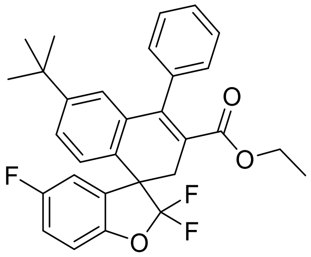

Chemical Formula: $C_{30}H_{27}F_3O_3$
Exact Mass: 492.1912

This compound was obtained following the general procedure D. Starting from 4-fluoro-2-iodophenol **1h** (47.6 mg, 0.2 mmol, 1 equiv), ammonium salt **2e** (91.4 mg, 0.2 mmol), alkyne **5a** (66 µL, 0.4 mmol, 2 equiv), $PdBr_2$ (2.7 mg, 0.01 mmol), **L3** (7.7 mg, 0.02 mmol), Cs_2CO_3 (130 mg, 0.4 mmol, 2.0 equiv), and toluene (1.0 mL). Purification on basic aluminum oxide (Pentane/Ethyl acetate gradient from 99/1 to 95/5) afforded **6j** (80 mg, 81%) as a colorless oil.

^{1}H NMR (300 MHz, $CDCl_3$) δ 7.53 – 7.32 (m, 3H), 7.30 – 7.16 (m, 3H), 7.14 – 6.96 (m, 3H), 6.91 (d, *J* = 2.1 Hz, 1H), 6.66 (d, *J* = 8.1 Hz, 1H), 4.08 – 3.78 (m, 2H), 3.41 – 3.01 (m, 2H), 1.13 (s, 9H), 0.89 (t, *J* = 7.1 Hz, 3H).

^{13}C NMR (75 MHz, $CDCl_3$) δ 167.9, 160.9, 151.8, 146.0, 138.9, 136.0, 130.1, 128.9, 128.1, 127.6, 126.8, 126.5, 126.5, 124.0, 116.6 (d, *J* = 24.5 Hz), 112.1 (d, *J* = 25.7 Hz), 111.4 (d, *J* = 8.7 Hz), 60.5, 54.0 (t, *J* = 24.8 Hz), 34.7, 33.0 (d, *J* = 7.9 Hz), 31.1, 13.7.

^{19}F NMR (282 MHz, $CDCl_3$) δ -72.3 (dd, *J* = 141.4, 3.0 Hz, 1F), - 73.7 (d, *J* = 141.4 Hz, 1F), -118.4 (td, *J* = 8.2, 4.2 Hz).

HRMS (EI): calculated for $C_{30}H_{27}F_3O_3$ $[M]^+$: 492.1907, found: 492.1904.

6k

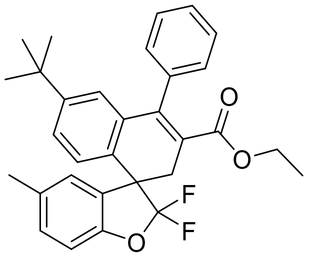

Chemical Formula: $C_{31}H_{30}F_2O_3$
Exact Mass: 488.2163

This compound was obtained following the general procedure D. Starting from 4-methyl-2-iodophenol **1b** (46.8 mg, 0.2 mmol, 1 equiv), ammonium salt **2e** (91.4 mg, 0.2 mmol), alkyne **5a** (66 µL, 0.4 mmol, 2 equiv), $PdBr_2$ (2.7 mg, 0.01 mmol), **L3** (7.7 mg, 0.02 mmol), Cs_2CO_3 (130 mg, 0.4 mmol, 2.0 equiv), and toluene (1.0 mL). Purification on basic aluminum oxide (Pentane/Ethyl acetate gradient from 99/1 to 95/5) afforded **6k** (80 mg, 82%) as a colorless oil.

^{1}H NMR (300 MHz, $CDCl_3$) δ 7.47 – 7.35 (m, 3H), 7.26 – 7.13 (m, 4H), 7.10 – 7.03 (m, 1H), 6.98 – 6.88 (m, 2H), 6.68 (d, *J* = 8.1 Hz, 1H), 4.07 – 3.86 (m, 2H), 3.36 – 3.11 (m, 2H), 2.38 (s, 3H), 1.14 (s, 9H), 0.90 (t, *J* = 7.2 Hz, 3H).

^{13}C NMR (75 MHz, $CDCl_3$) δ 168.0, 153.0 – 152.8 (m), 151.4, 145.9, 139.1 136.1, 133.5, 133.3 (dd, J = 274.5, 267.0 Hz), 133.23, 131.1 – 130.8 (m), 130.4, 130.3, 128.9, 128.1, 127.5, 127.0, 126.3, 126.2, 125.1, 124.4, 110.1, 60.4, 53.5 (t, *J* = 24.8 Hz), 34.7, 33.1 (d, *J* = 7.6 Hz), 31.1, 21.2, 13.7.

^{19}F NMR (282 MHz, $CDCl_3$) δ -72.9 (dd, *J* = 142.3, 2.7 Hz, 1F), -73.7 (d, *J* = 142.1 Hz, 1F).

HRMS (EI): calculated for $C_{31}H_{30}F_2O_3$ $[M]^+$: 488.2158, found: 488.2155.

6l

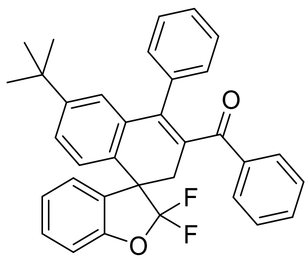

Chemical Formula: $C_{34}H_{28}F_2O_2$
Exact Mass: 506.2057

This compound was obtained following the general procedure D. Starting from 2-iodophenol **1a** (44.0 mg, 0.2 mmol, 1 equiv), ammonium salt **2e** (91.4 mg, 0.2 mmol), alkyne **5b** (82.4 g, 0.4 mmol, 2 equiv), $PdBr_2$ (2.7 mg, 0.01 mmol), **L3** (7.7 mg, 0.02 mmol), Cs_2CO_3 (130 mg, 0.4 mmol, 2.0 equiv), and toluene (1.0 mL). Purification on basic aluminum oxide (Pentane/Ethyl acetate gradient from 99/1 to 95/5) afforded **6l** (80 mg, 79%) as a colorless oil.

^{1}H NMR (300 MHz, $CDCl_3$) δ 7.72 – 7.61 (m, 2H), 7.43 – 7.34 (m, 1H), 7.33 – 7.16 (m, 5H), 7.16 – 7.03 (m, 7H), 7.00 (d, *J* = 2.0 Hz, 1H), 6.64 (d, *J* = 8.1 Hz, 1H), 3.56 (dd, *J* = 16.8, 2.0 Hz, 1H), 3.09 (dd, *J* = 16.8, 1.5 Hz, 1H), 1.15 (s, 9H).

^{13}C NMR (75 MHz, $CDCl_3$) δ 199.9, 155.1 – 154.8 (m), 151.6, 142.3, 137.3, 137.2,135.4, 133.4 (dd, J = 274.5, 267.0 Hz), 132.3, 132.1, 131.2 – 131.0 (m), 130.5, 130.0, 129.4, 129.3, 128.2, 128.0, 127.9, 127.1, 126.1, 125.8, 124.9, 124.1, 110.5, 53.4 (t, *J* = 24.8 Hz), 35.0 (d, *J* = 8.0 Hz), 34.7, 31.2.

^{19}F NMR (282 MHz, $CDCl_3$) δ -71.3 (d, *J* = 140.7 Hz, 1F), -73.9 (dd, *J* = 141.0, 2.9 Hz, 1F).

HRMS (ESI): calculated for $C_{34}H_{28}F_2O_2Na$ $[M+Na]^+$: 529.1954, found: 529.1962.

6m

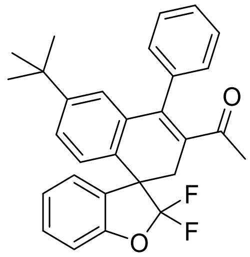

Chemical Formula: $C_{29}H_{26}F_2O_2$
Exact Mass: 444.1901

This compound was obtained following the general procedure D. Starting from iodophenol **1a** (44.0 mg, 0.2 mmol, 1 equiv), ammonium salt **2e** (91.4 mg, 0.2 mmol), alkyne **5c** (58 µl, 0.4 mmol, 2 equiv), $PdBr_2$ (5.4 mg, 0.02 mmol), **L3** (15.4 mg, 0.04 mmol), Cs_2CO_3 (130 mg, 0.4 mmol, 2.0 equiv), and toluene (1.0 mL). Purification on basic aluminum oxide (Pentane/Ethyl acetate gradient from 99/1 to 95/5) afforded **6m** (40 mg, 45%) as a slight yellow oil.

^{1}H NMR (300 MHz, $CDCl_3$) δ 7.53 – 7.45 (m, 3H), 7.41 – 7.33 (m, 1H), 7.31 – 7.14 (m, 5H), 7.10 – 7.00 (m, 1H), 6.91 (d, *J* = 2.1 Hz, 1H), 6.64 (d, *J* = 8.1 Hz, 1H), 3.31 – 3.18 (m, 1H), 3.10 (dd, *J* = 16.5, 1.4 Hz, 1H), 1.74 (s, 3H), 1.14 (s, 9H).

^{13}C NMR (75 MHz, $CDCl_3$) δ 204.7, 155.0 – 154.8 (m), 151.6, 143.3, 138.2, 135.9, 134.0, 131.3 – 131.1 (m), 130.3, 130.0, 128.9, 128.8, 127.0, 126.4, 126.2, 124.8, 124.0, 110.5, 53.6 (t, *J* = 24.8 Hz), 34.7, 34.2 (d, *J* = 7.5 Hz), 31.1, 30.4.

^{19}F NMR (282 MHz, $CDCl_3$) δ -72.4 (dd, *J* = 142.7, 2.8 Hz, 1F), -73.3 (d, *J* = 142.6 Hz, 1F).

HRMS (ESI): calculated for $C_{29}H_{26}F_2O_2Na$ $[M+Na]^+$: 467.1798, found: 467.1794.

6n

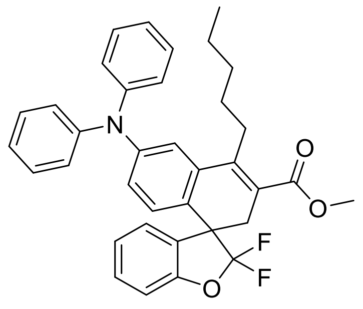

Chemical Formula: $C_{36}H_{33}F_2NO_3$
Exact Mass: 565.2429

This compound was obtained following the general procedure D. Starting from 2-iodophenol **1a** (44.0 mg, 0.2 mmol, 1 equiv), ammonium salt **2r** (113.6 mg, 0.2 mmol), alkyne **5d** (67 µl, 0.4 mmol, 2 equiv), $PdBr_2$ (2.7 mg, 0.01 mmol), **L3** (7.7 mg, 0.02 mmol), Cs_2CO_3 (130 mg, 0.4 mmol, 2.0 equiv), and toluene (1.0 mL). Purification on basic aluminum oxide (Pentane/Ethyl acetate gradient from 99/1 to 95/5) afforded **6n** (92 mg, 79%) as a slight yellow oil.

^{1}H NMR (300 MHz, $CDCl_3$) δ 7.29 – 7.11 (m, 7H), 7.09 – 7.01 (m, 5H), 7.00 – 6.89 (m, 3H), 6.79 (dd, *J* = 8.4, 2.3 Hz, 1H), 6.43 (d, *J* = 8.4 Hz, 1H), 3.69 (s, 3H), 3.21 – 3.03 (m, 1H), 2.95 – 2.70 (m, 2H), 2.65 – 2.41 (m, 1H), 1.37 – 1.10 (m, 6H), 0.79 – 0.70 (m, 3H).

^{13}C NMR (75 MHz, $CDCl_3$) δ 168.2, 155.0 – 154.7 (m), 154.2, 148.4, 147.4, 146.8, 136.2, 130.6, 129.9, 129.5, 128.3, 127.5 – 127.2 (m), 125.0, 124.7, 123.8, 123.6, 122.7, 122.6, 120.1, 110.4, 53.1 (t, J = 24.0 Hz), 51.8, 33.1 (d, *J* = 8.2 Hz), 32.01, 29.2 (d, *J* = 6.8 Hz), 22.5, 14.2.

^{19}F NMR (282 MHz, $CDCl_3$) δ -72.4 (dd, *J* = 142.3, 3.1 Hz, 1F), -74.3 (d, *J* = 142.1 Hz, 1F).

HRMS (ESI): calculated for $C_{36}H_{34}F_2NO_3Na$ $[M+H]^+$: 566.2507, found: 566.2504.

6o

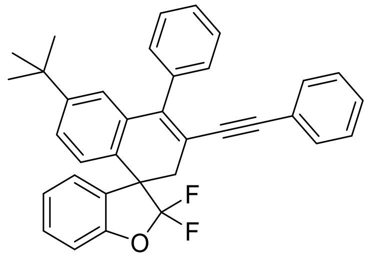

Chemical Formula: $C_{35}H_{28}F_2O$
Exact Mass: 502.2108

This compound was obtained following the general procedure D. Starting from iodophenol **1a** (44 mg, 0.2 mmol, 1 equiv), ammonium salt **2e** (91.4 mg, 0.2 mmol), alkyne **5e** (80.8 g, 0.4 mmol, 2 equiv), $PdBr_2$ (5.4 mg, 0.02 mmol), **L3** (15.4 mg, 0.04 mmol), Cs_2CO_3 (130 mg, 0.4 mmol, 2.0 equiv), and toluene (1.0 mL). Purification on basic aluminum oxide (Pentane/Ethyl acetate gradient from 99/1 to 95/5) afforded **6o** (45 mg, 45%) as a slight yellow oil.

^{1}H NMR (300 MHz, $CDCl_3$) δ 7.56 – 7.44 (m, 5H), 7.41 – 7.31 (m, 3H), 7.25 – 7.14 (m, 7H), 7.10 – 7.02 (m, 2H), 6.65 (d, *J* = 8.2 Hz, 1H), 3.31 – 3.20 (m, 1H), 3.10 (dd, *J* = 16.5, 1.2 Hz, 1H), 1.18 (s, 9H).

^{13}C NMR (75 MHz, $CDCl_3$) δ 155.0 – 154.9 (m), 151.5, 144.4, 138.5, 136.2, 132.7, 131.5, 130.4, 129.9, 129.4, 128.6, 128.3, 128.2, 128.0, 127.9, 127.0, 125.1, 124.8, 124.7, 123.9, 123.6, 115.6, 110.5, 95.5, 90.4, 53.5 (t, J = 24.8 Hz), 36.5 (d, J = 6.8 Hz), 34.7, 31.2.

^{19}F NMR (282 MHz, $CDCl_3$) δ -73.4 (dd, *J* = 142.0, 2.8 Hz, 1F), -74.5 (d, *J* = 142.0 Hz, 1F).

HRMS (EI): calculated for $C_{35}H_{28}F_2O$ $[M]^+$: 502.2103, found: 502.2105.

6p

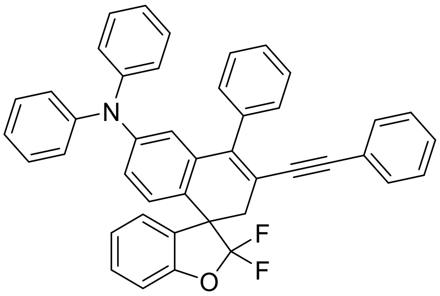

Chemical Formula: $C_{43}H_{29}F_2NO$
Exact Mass: 613.2217

This compound was obtained following the general procedure D. Starting from iodophenol **1a** (44 mg, 0.2 mmol, 1 equiv), ammonium salt **2r** (113.6 mg, 0.2 mmol), alkyne **5e** (80.8 g, 0.4 mmol, 2 equiv), $PdBr_2$ (5.4 mg, 0.02 mmol), **L3** (15.4 mg, 0.04 mmol), Cs_2CO_3 (130 mg, 0.4 mmol, 2.0 equiv), and toluene (1.0 mL). Purification on basic aluminum oxide (Pentane/Ethyl acetate gradient from 99/1 to 95/5) afforded **6p** (49 mg, 40%) as a slight yellow oil.

^{1}H NMR (300 MHz, $CDCl_3$) δ 7.42 – 7.26 (m, 7H), 7.25 – 7.13 (m, 10H), 7.08 – 6.92 (m, 7H), 6.80 – 6.71 (m, 2H), 6.57 – 6.50 (m, 1H), 3.26 (dd, *J* = 16.4, 2.6 Hz, 1H), 3.09 (dd, *J* = 16.5, 1.2 Hz, 1H).

^{13}C NMR (75 MHz, $CDCl_3$) δ 155.1 – 154.7 (m), 148.0, 147.2, 143.8, 138.0, 137.3, 132.7, 131.6, 130.5, 130.2, 129.9, 129.3, 128.3, 128.2, 128.2, 127.9, 127.8, 124.8, 124.6, 123.9, 123.6, 123.3, 122.1, 122.0, 115.9, 110.5, 95.7, 90.3, 53.40 (t, *J* = 25.5 Hz), 36.7 (d, *J* = 7.5 Hz).

^{19}F NMR (282 MHz, $CDCl_3$) δ -73.6 (dd, *J* = 141.9, 2.9 Hz, 1F), -74.7 (d, *J* = 141.5 Hz, 1F).

HRMS (ESI): calculated for $C_{43}H_{30}F_2NO$ $[M+H]^+$: 614.2296, found: 614.2294.

6q

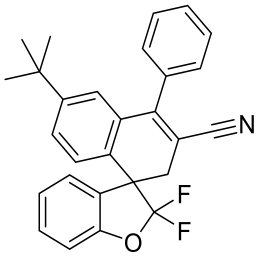

Chemical Formula: $C_{28}H_{23}F_2NO$
Exact Mass: 427.1748

This compound was obtained following the general procedure D. Starting from 2-iodophenol **1a** (44.0 mg, 0.2 mmol, 1 equiv), ammonium salt **2e** (91.4 mg, 0.2 mmol), alkyne **5f** (51 g, 0.4 mmol, 2 equiv), $PdBr_2$ (5.4 mg, 0.02 mmol), **L3** (15.4 mg, 0.04 mmol), Cs_2CO_3 (130 mg, 0.4 mmol, 2.0 equiv), and toluene (1.0 mL). Purification on basic aluminum oxide (Pentane/Ethyl acetate gradient from 99/1 to 95/5) afforded **6q** (80 mg, 94%) as a slight yellow solid.

^{1}H NMR (300 MHz, $CDCl_3$) δ 7.56 – 7.48 (m, 3H), 7.48 – 7.36 (m, 3H), 7.28 (dd, *J* = 8.1, 2.1 Hz, 1H), 7.25 – 7.16 (m, 2H), 7.08 (dt, *J* = 8.1, 0.8 Hz, 1H), 7.03 (d, *J* = 2.1 Hz, 1H), 6.66 (d, *J* = 8.1 Hz, 1H), 3.27 – 3.01 (m, 2H), 1.16 (s, 9H).

^{13}C NMR (75 MHz, $CDCl_3$) δ 155.2 – 154.7 (m), 153.7, 152.1, 136.1, 134.1, 132.8 – 132.6 (m), 130.9 – 130.7 (m), 130.5, 129.6, 129.4, 129.1, 128.7, 127.6, 127.4, 126.3, 124.7, 124.2, 118.7, 110.8, 104.6, 53.1 (t, *J* = 25.1 Hz), 34.8, 33.4 (d, *J* = 6.2 Hz), 31.1.

^{19}F NMR (282 MHz, $CDCl_3$) δ -73.0 (dd, *J* = 142.5, 2.5 Hz, 1F), -73.8 (d, *J* = 142.6 Hz), 1F).

HRMS (EI): calculated for $C_{28}H_{23}F_2NO$ $[M]^+$: 427.1742, found: 427.1737.

6r

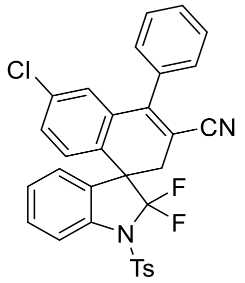

Chemical Formula: $C_{31}H_{21}ClF_2N_2O_2S$
Exact Mass: 558.0980

This compound was obtained following the general procedure D. Starting from iodophenol **1o** (74.6 mg, 0.2 mmol, 1 equiv), ammonium salt **2q** (87 mg, 0.2 mmol), alkyne **5f** (51 g, 0.4 mmol, 2 equiv), $PdBr_2$ (2.7 mg, 0.01 mmol), **L3** (7.7 mg, 0.02 mmol), Cs_2CO_3 (130 mg, 0.4 mmol, 2.0 equiv), and toluene (1.0 mL). Purification on basic aluminum oxide (Pentane/Ethyl acetate gradient from 99/1 to 95/5) afforded **6r** (60 mg, 54%) as a white solid.

^{1}H NMR (300 MHz, $CDCl_3$) δ 7.85 (d, *J* = 8.2 Hz, 2H), 7.72 (d, *J* = 8.2 Hz, 1H), 7.63 – 7.48 (m, 4H), 7.47 – 7.39 (m, 2H), 7.31 – 7.26 (m, 2H), 7.20 (td, *J* = 7.5, 1.0 Hz, 1H), 7.16 – 7.10 (m, 1H), 7.07 (dd, *J* = 8.3, 2.2 Hz, 1H), 6.96 (d, *J* = 2.2 Hz, 1H), 6.37 (d, *J* = 8.3 Hz, 1H), 3.22 – 3.02 (m, 1H), 2.86 (dd, *J* = 16.9, 1.6 Hz, 1H), 2.41 (s, 3H).

^{13}C NMR (75 MHz, $CDCl_3$) δ 152.0, 145.5, 139.6, 136.4, 135.4, 135.3, 135.1, 131.9 – 131.7 (m), 130.6, 130.3, 130.0, 129.9, 129.3, 129.2, 129.1, 129.0, 128.6, 128.5, 128.0, 125.3, 124.5, 117.9, 113.7, 106.4, 53.9 (t, *J* = 23.2 Hz), 33.2 (d, *J* = 10.1 Hz), 21.8.

^{19}F NMR (282 MHz, $CDCl_3$) δ -76.0 (d, *J* = 183.6 Hz, 1F), -77.5 (d, *J* = 183.6 Hz, 1F).

HRMS (ESI): calculated for $C_{31}H_{21}ClF_2N_2O_2SNa$ $[M+Na]^+$: 581.0877, found: 581.0876.

4. X-ray crystal structure analysis

Data were collected on a Bruker Kappa APEX II Duo and a a STOE-IPDS II diffractometer, respectively. The structures were solved by direct methods (SHELXS-97: Sheldrick, G. M. *Acta Cryst.* **2008**, *A64*, 112.) and refined by full-matrix least-squares procedures on F^2 (SHELXL-2018: Sheldrick, G. M. *Acta Cryst.* **2015**, *C71*, 3.). XP (Bruker AXS) was used for graphical representations.

Crystal data of compound **4f**: $C_{21}H_{14}F_2O$, M = 320.32, monoclinic, space group $P2_1/c$, a = 7.3253(6), b = 5.9659(5), c = 35.379(3) Å, β = 91.742(3)°, V = 1545.4(2) Å^3, T = 150(2) K, Z = 4, 17845 reflections measured, 2714 independent reflections (R_{int} = 0.0367), final R values ($I > 2\sigma(I)$): R_1 = 0.0511, wR_2 = 0.1279, final R values (all data): R_1 = 0.0541, wR_2 = 0.1300, 217 parameters.

CCDC 2006609 (compound **4f**) contains the supplementary crystallographic data for this paper. These data are provided free of charge by the joint Cambridge Crystallographic Data Centre and Fachinformationszentrum Karlsruhe Access Structures service www.ccdc.cam.ac.uk/structures.

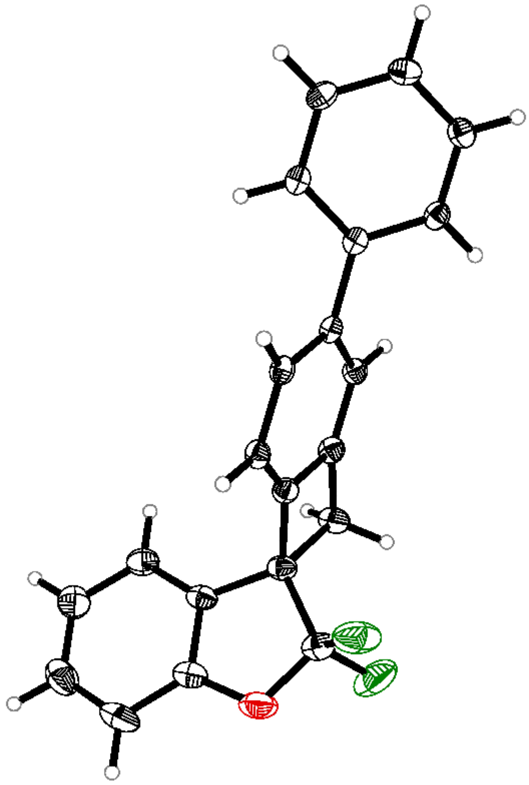

Molecular structure of **4f** in the solid state. Displacement ellipsoids correspond to 30% probability.

Crystal data of compound **6a**: $C_{30}H_{28}F_2O_3$, M = 474.52, triclinic, space group $P\bar{1}$, a = 11.2613(6), b = 11.3205(7), c = 11.4530(7) Å, α = 102.213(5), β = 114.059(4), γ = 103.168(5)°, V = 1219.71(13) Å^3, T = 150(2) K, Z = 2, 21537 reflections measured, 5880 independent reflections (R_{int} = 0.0282), final R values ($I > 2\sigma(I)$): R_1 = 0.0371, wR_2 = 0.1000, final R values (all data): R_1 = 0.0488, wR_2 = 0.1037, 320 parameters.

CCDC 2006610 (compound **6a**) contains the supplementary crystallographic data for this paper. These data are provided free of charge by the joint Cambridge Crystallographic Data Centre and Fachinformationszentrum Karlsruhe Access Structures service www.ccdc.cam.ac.uk/structures.

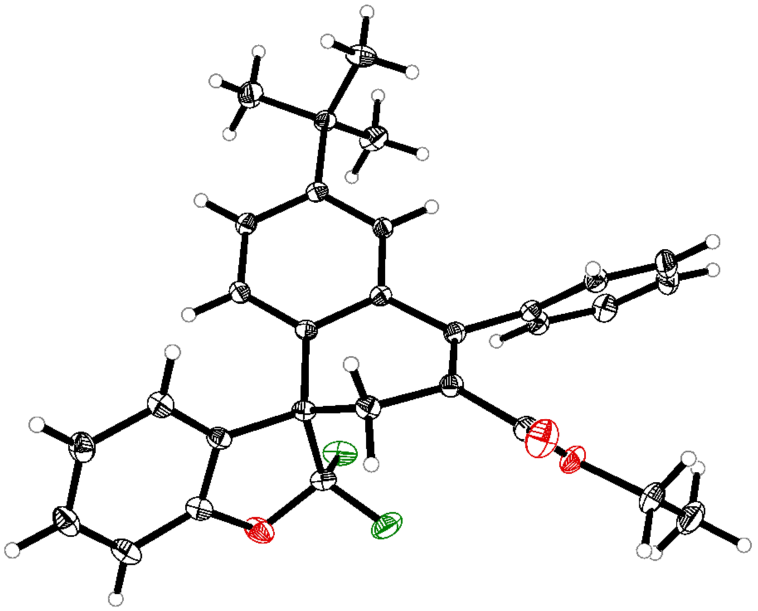

Molecular structure of **6a** in the solid state. Displacement ellipsoids correspond to 30% probability.

5. NMR spectra for new compounds

S1e

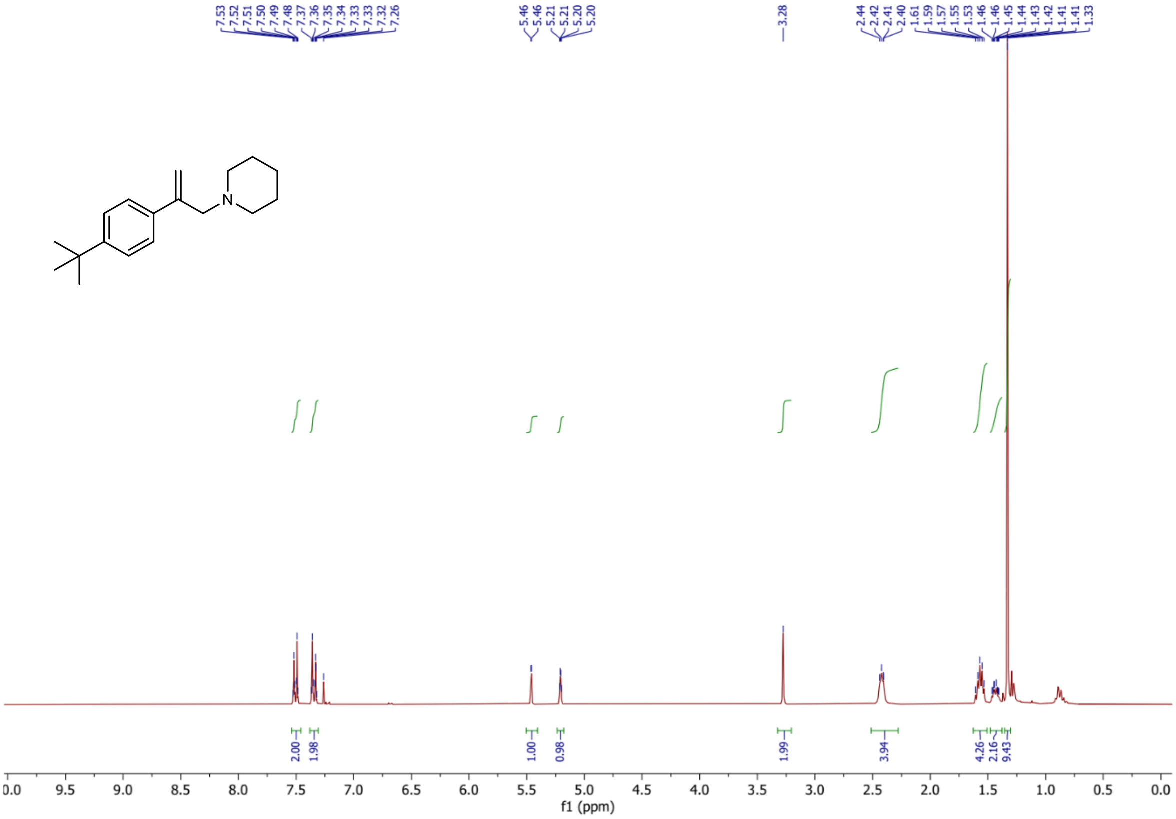

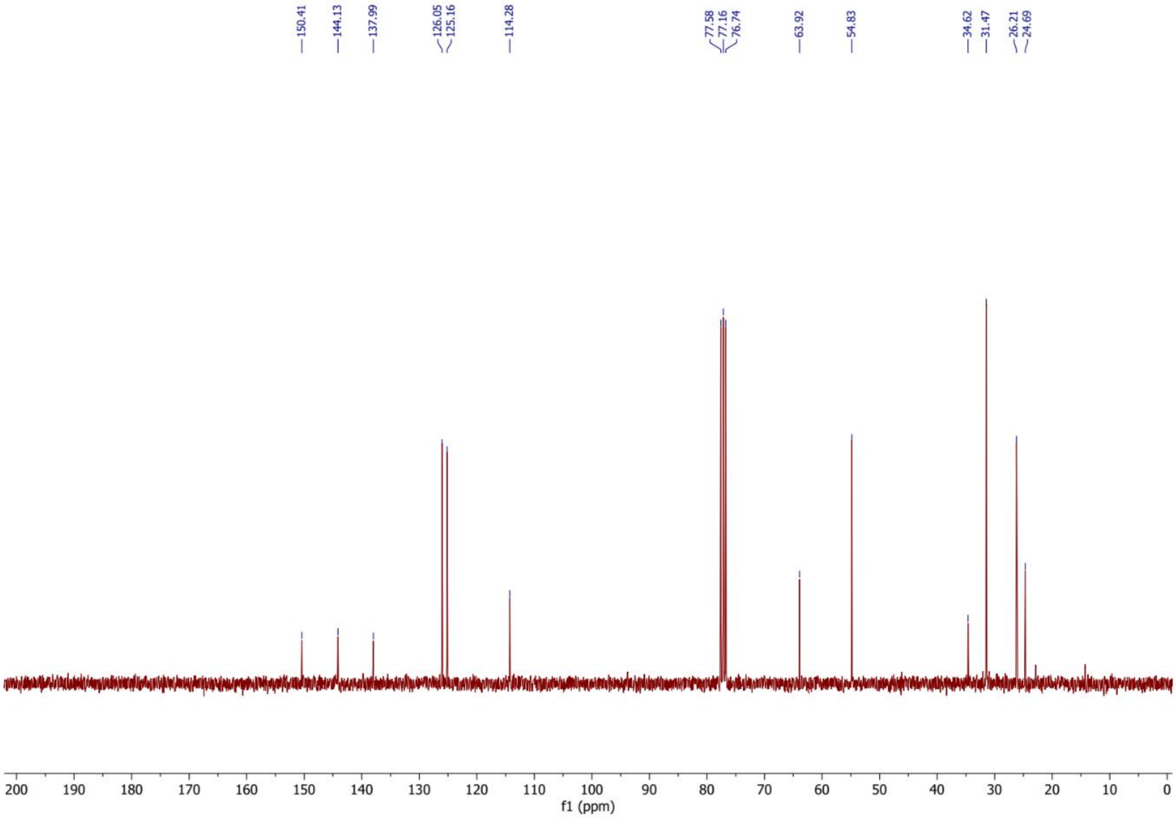

S1f

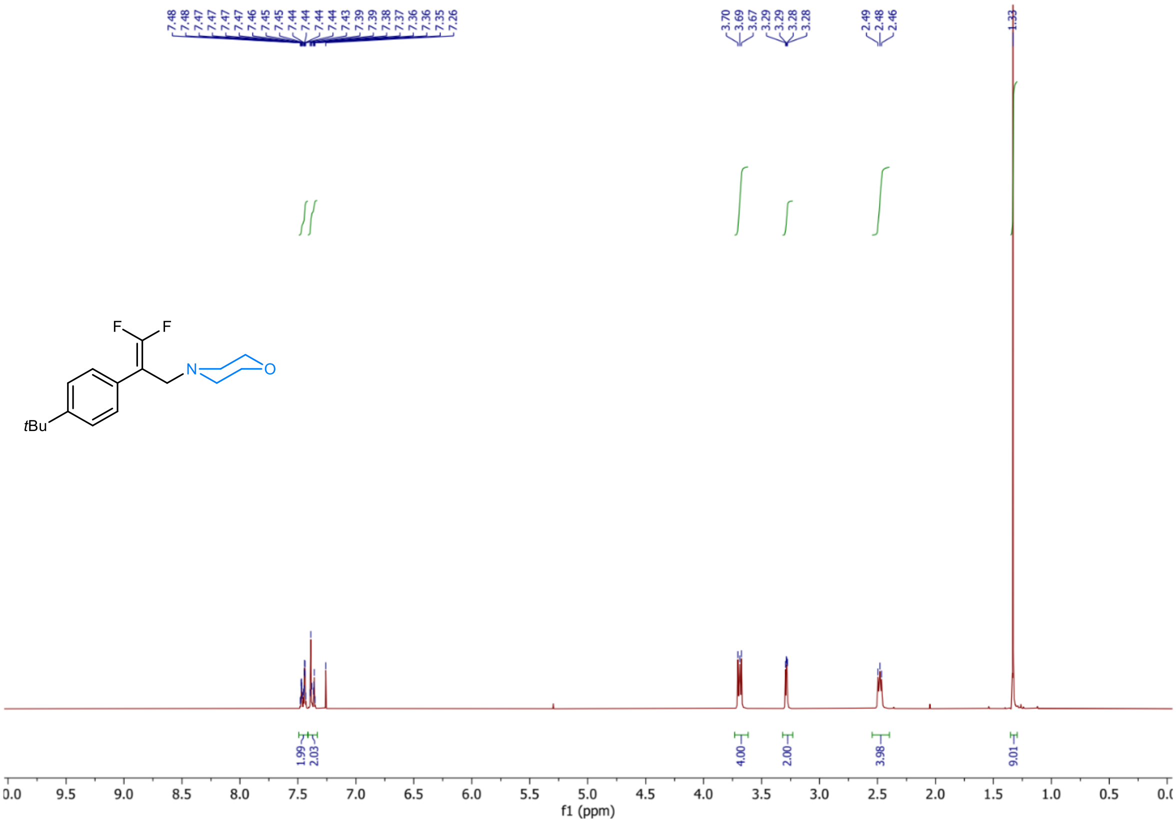

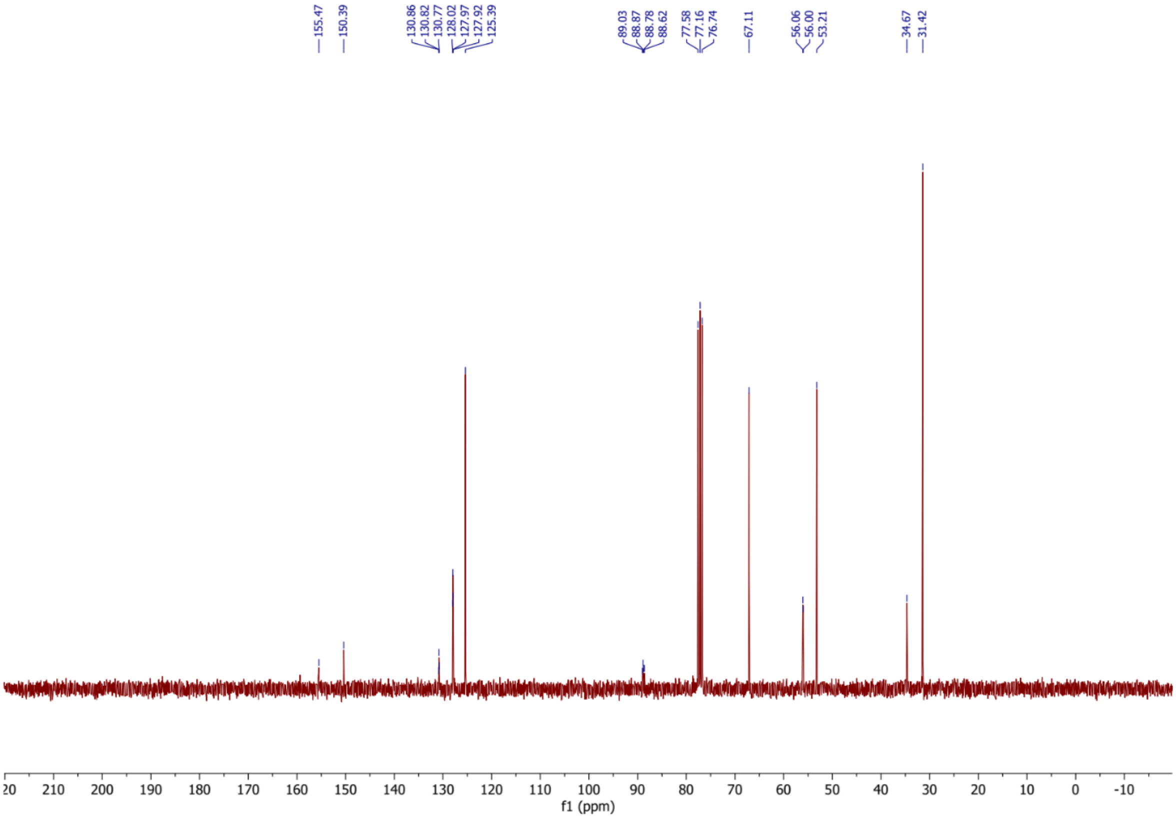

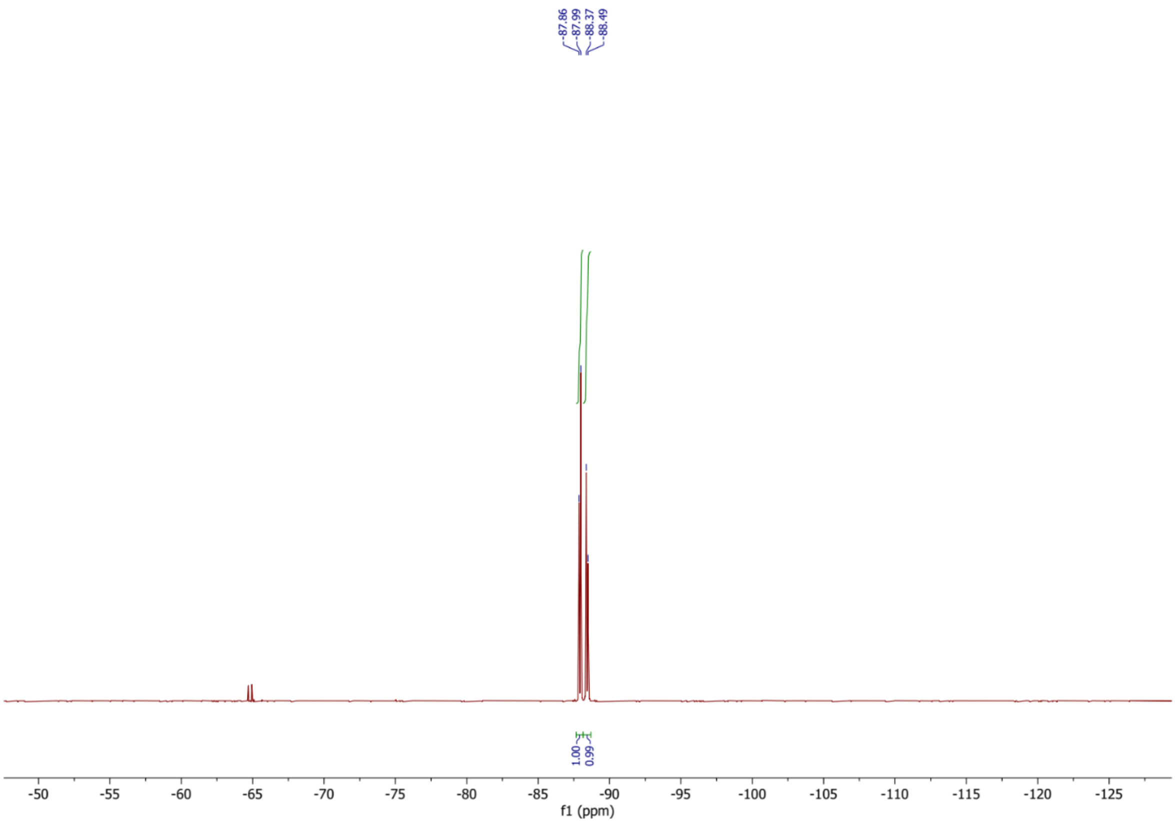

S1g

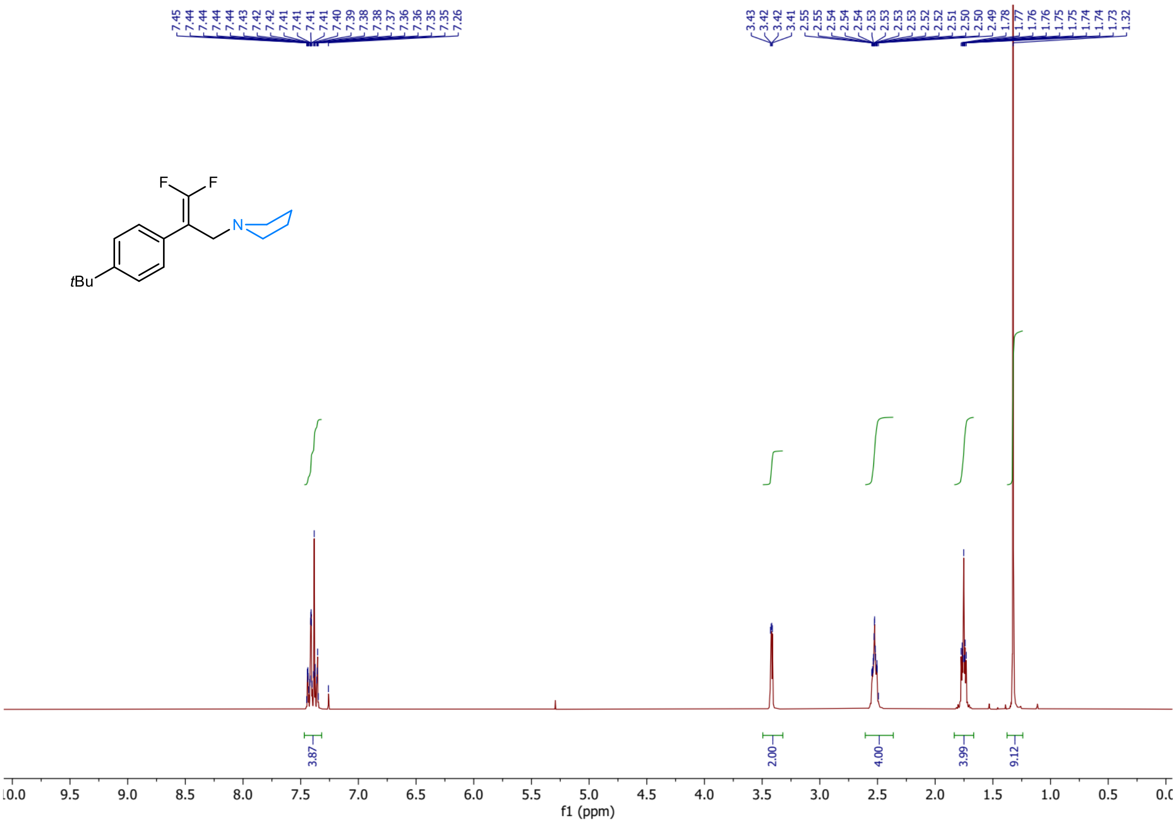

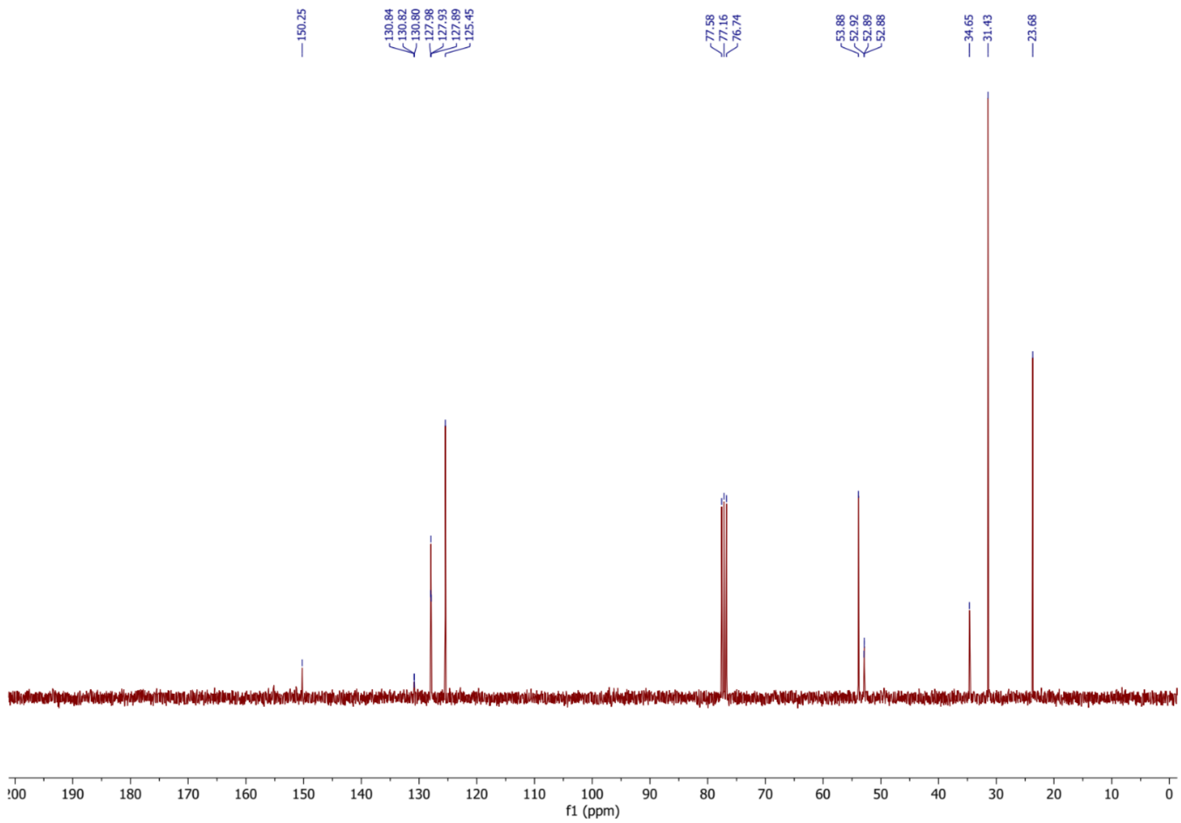

150.25
130.84
130.82
130.80
127.98
127.93
127.89
125.45
77.58
77.16
76.74
53.88
52.92
52.89
52.88
34.65
31.43
23.68
200
190
180
170
160
150
140
130
120
110
100
90
80
70
60
50
40
30
20
10
0
f1 (ppm)

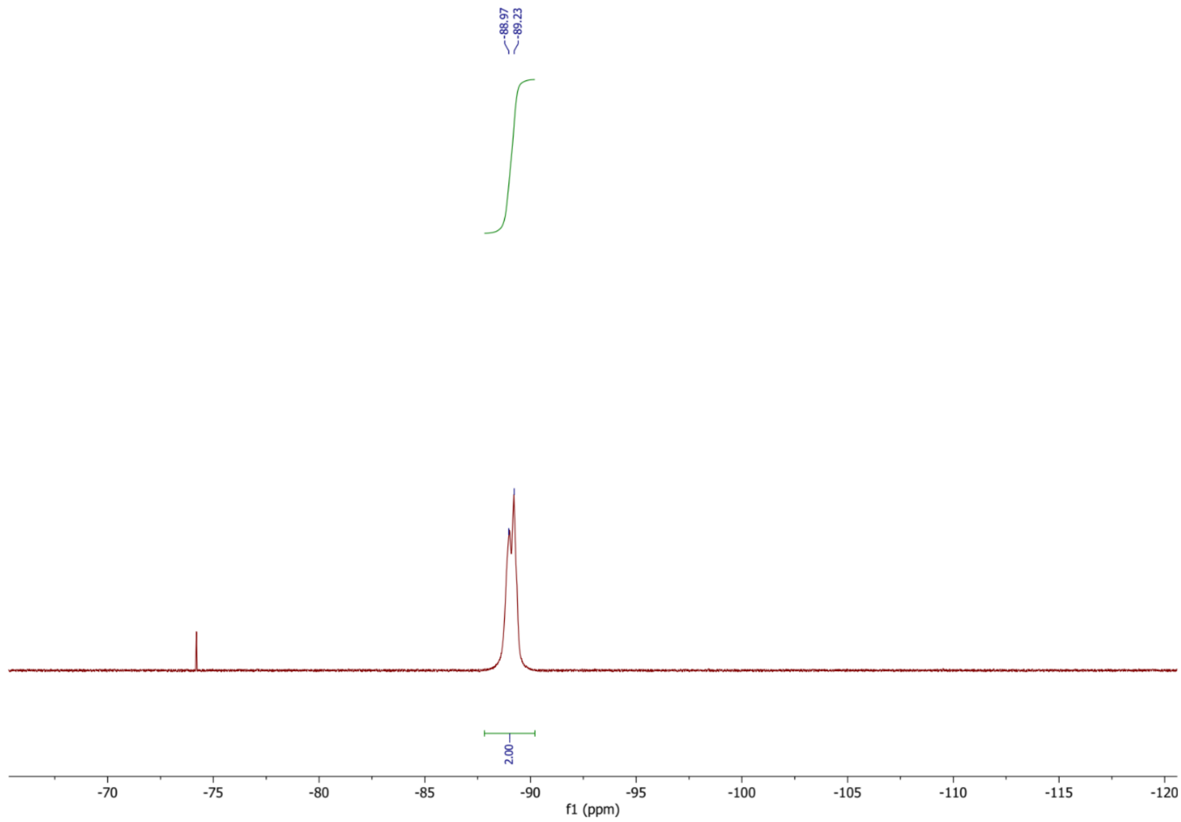

-88.97
-89.23
2.00
-70
-75
-80
-85
-90
-95
-100
-105
-110
-115
-120
f1 (ppm)

S1h

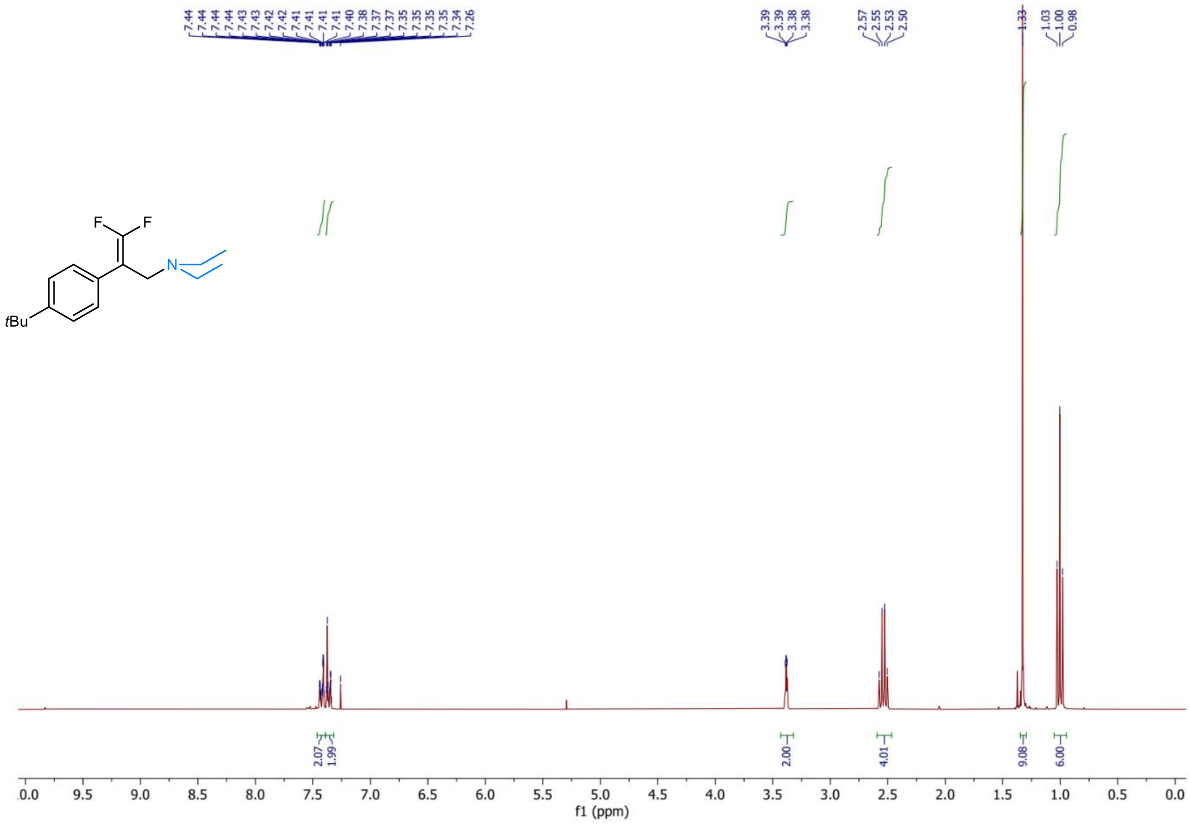
F
F
N
tBu
7.44
7.44
7.44
7.43
7.43
7.42
7.42
7.41
7.41
7.41
7.41
7.40
7.38
7.37
7.37
7.35
7.35
7.35
7.34
7.26
3.39
3.39
3.38
3.38
2.57
2.55
2.53
2.50
1.33
1.03
1.00
0.98
2.07
1.99
2.00
4.01
9.08
6.00
f1 (ppm)

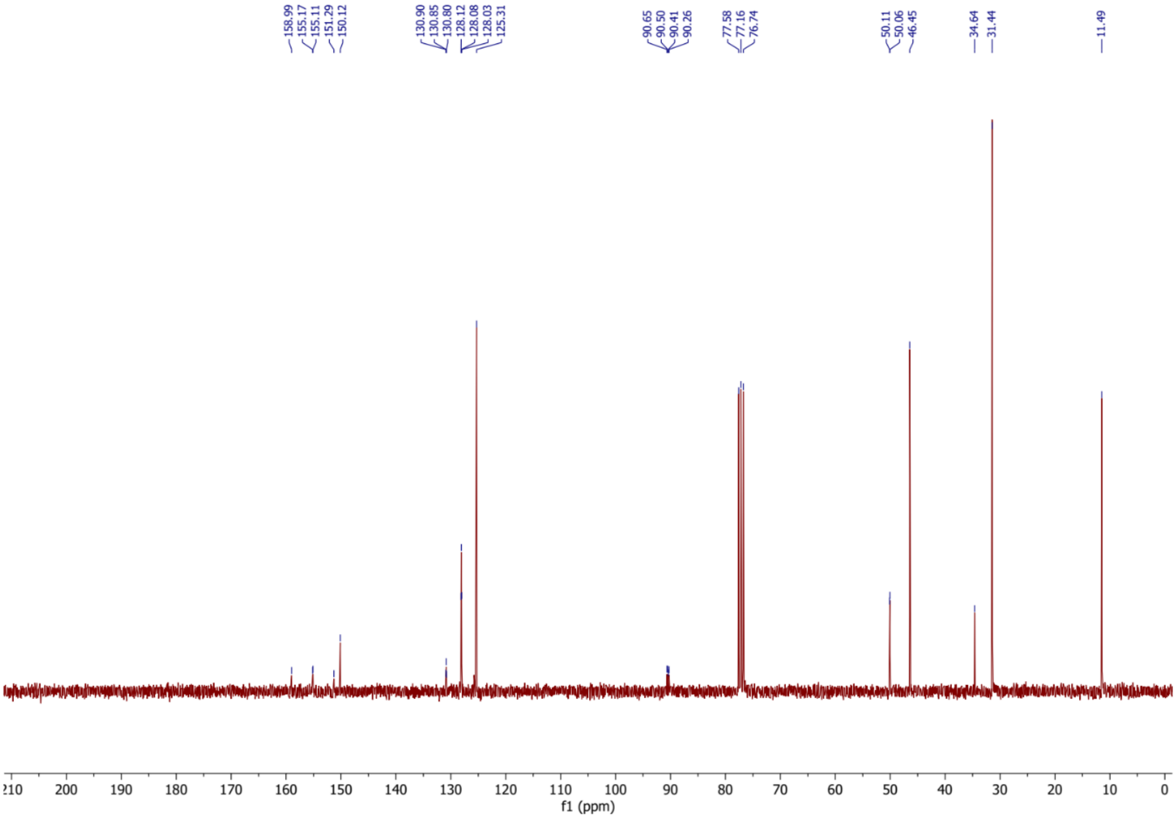
158.99
155.17
155.11
151.29
150.12
130.90
130.85
130.80
128.12
128.08
128.03
125.31
90.65
90.50
90.41
90.26
77.58
77.16
76.74
50.11
50.06
46.45
34.64
31.44
11.49
f1 (ppm)

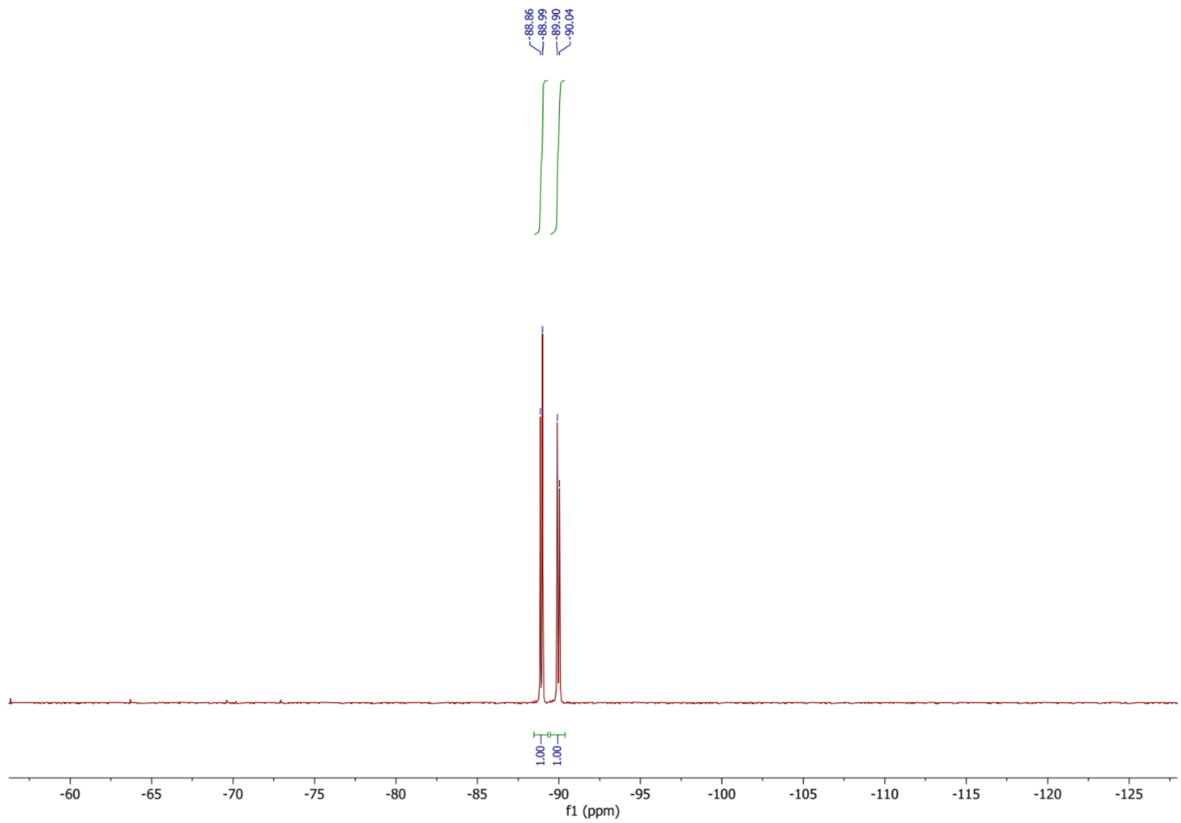
-88.86
-88.99
-89.90
-90.04
1.00
1.00
-60
-65
-70
-75
-80
-85
-90
-95
-100
-105
-110
-115
-120
-125
f1 (ppm)

S1j

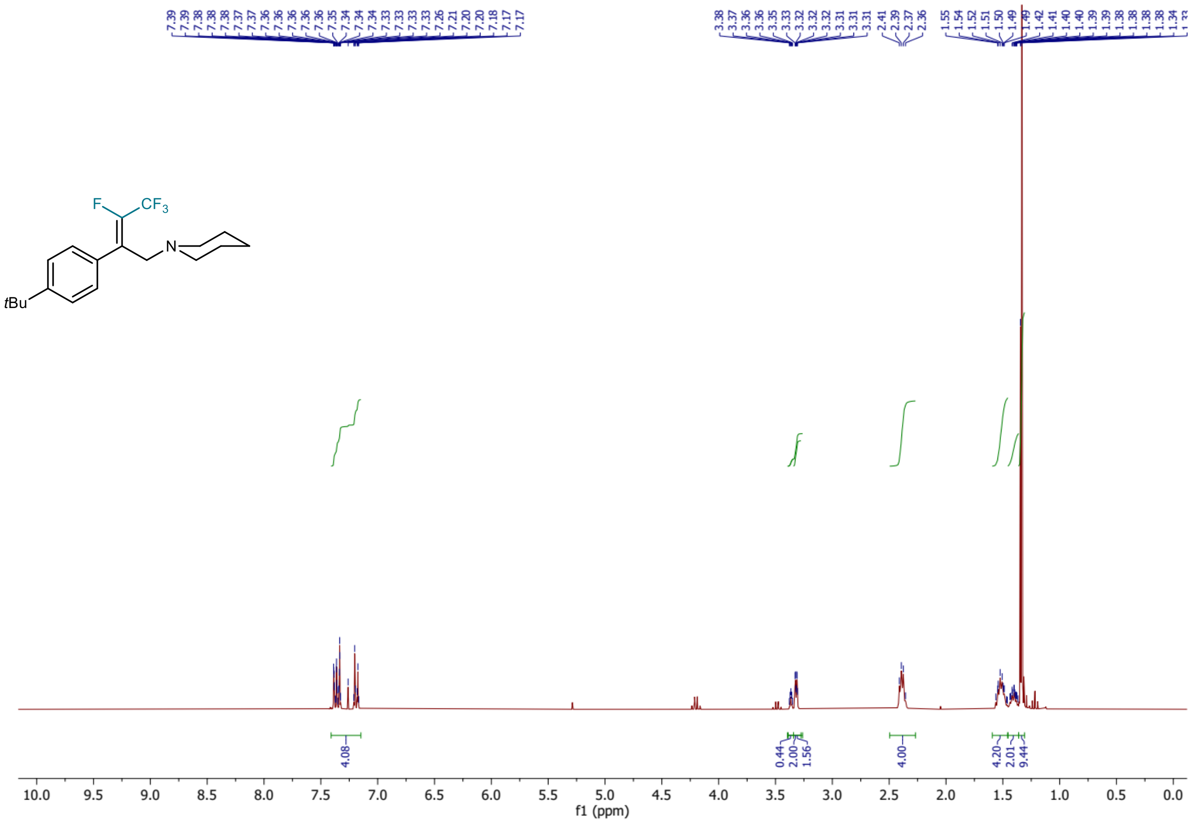
F
CF_3
N
*t*Bu
4.08
0.44
2.00
1.56
4.00
4.20
2.01
9.44
10.0
9.5
9.0
8.5
8.0
7.5
7.0
6.5
6.0
5.5
5.0
4.5
4.0
3.5
3.0
2.5
2.0
1.5
1.0
0.5
0.0
f1 (ppm)

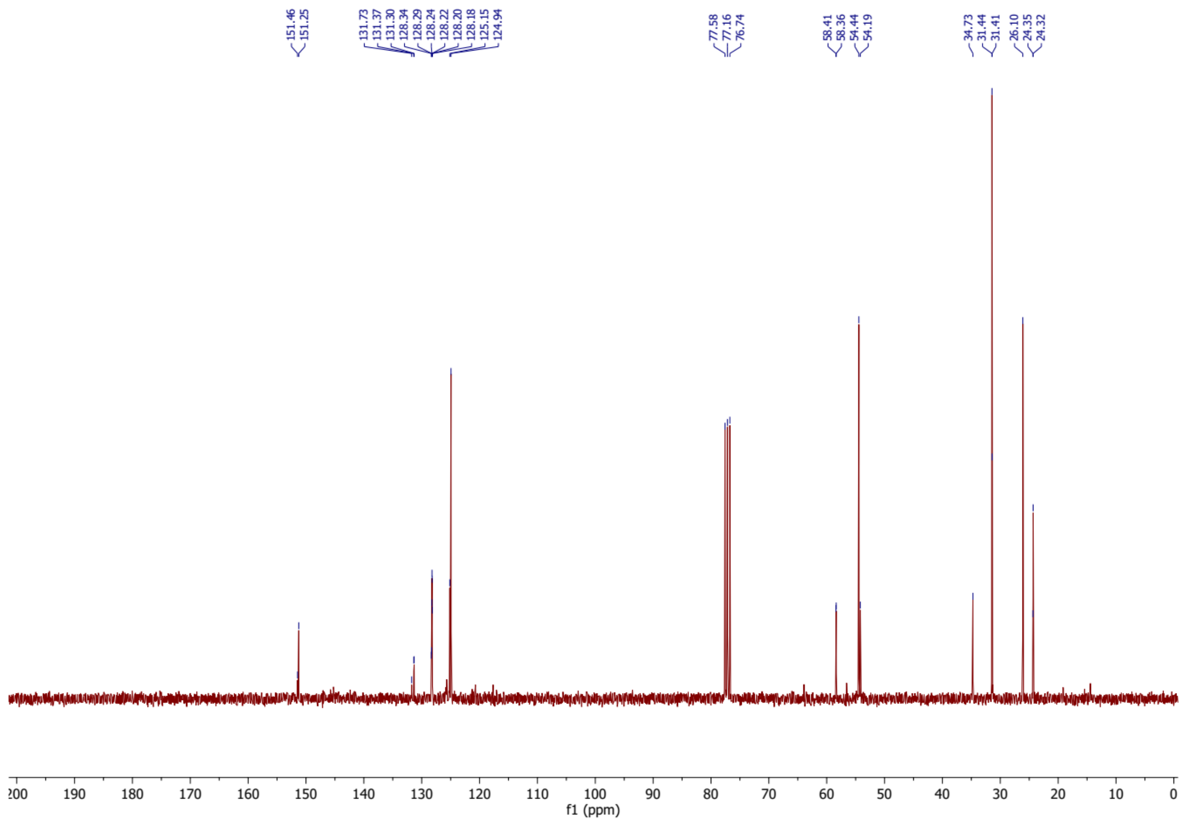
151.46
151.25
131.73
131.37
131.30
128.34
128.29
128.24
128.22
128.20
128.18
125.15
124.94
77.58
77.16
76.74
58.41
58.36
54.44
54.19
34.73
31.44
31.41
26.10
24.35
24.32
f1 (ppm)

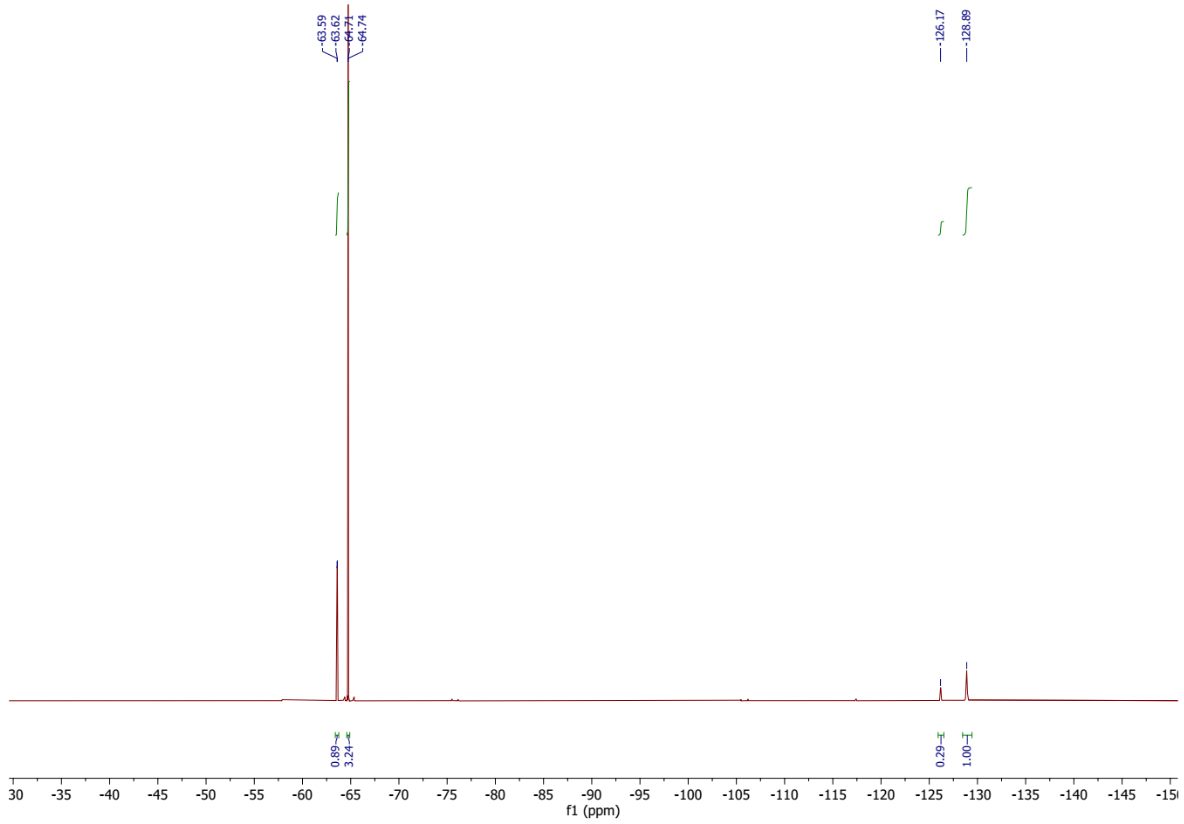
-63.59
-63.62
-64.71
-64.74
-126.17
-128.89
0.89
3.24
0.29
1.00
f1 (ppm)

S1o

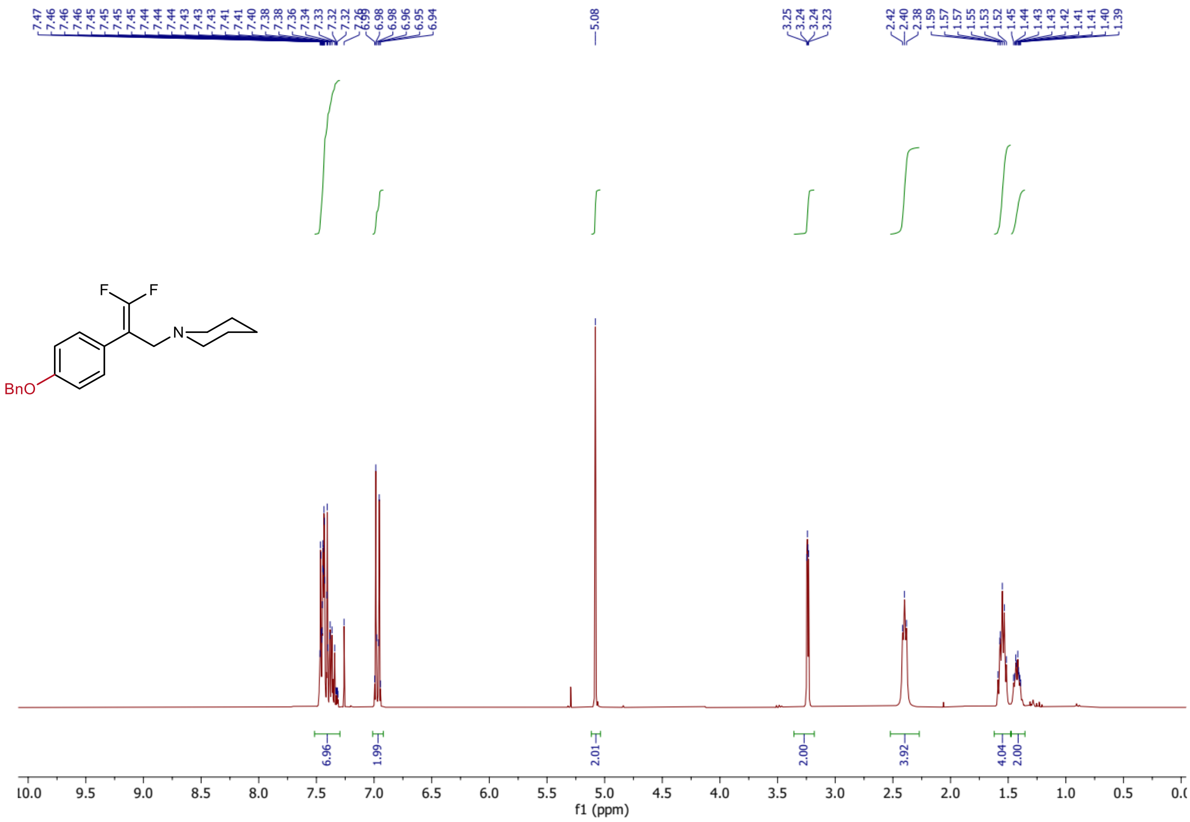

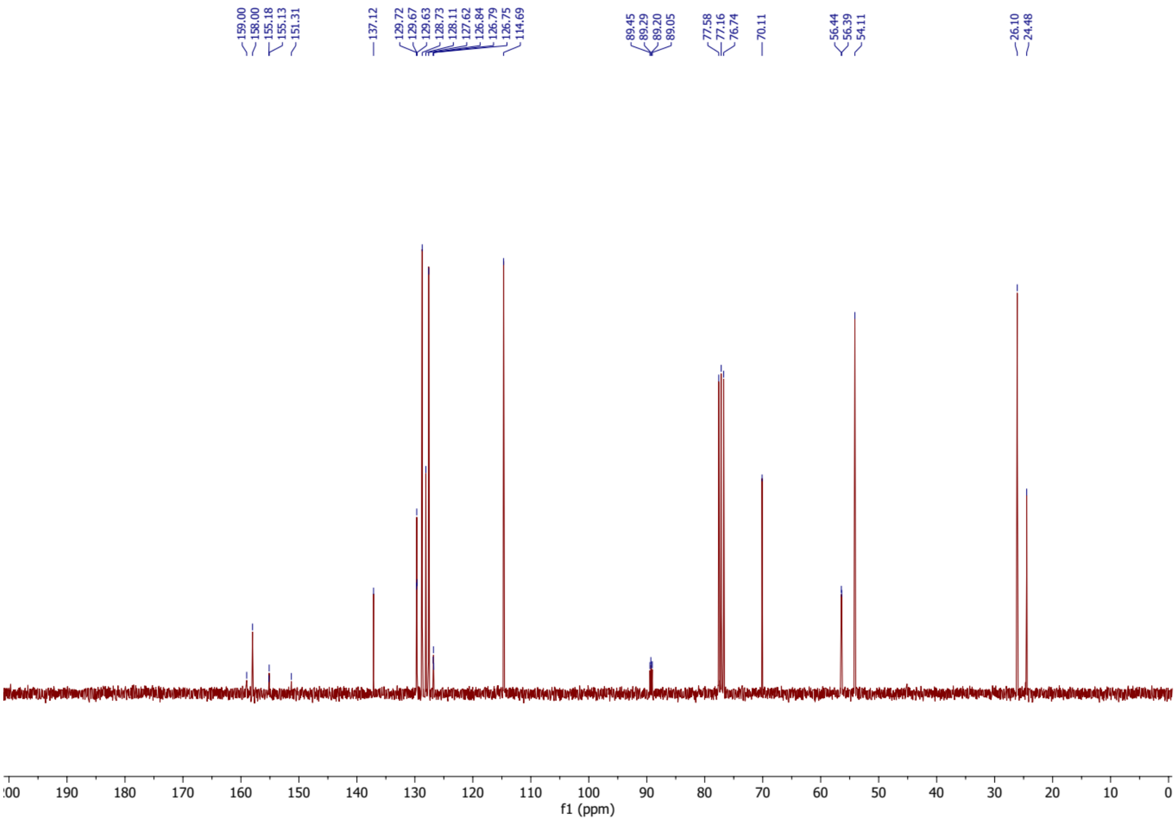

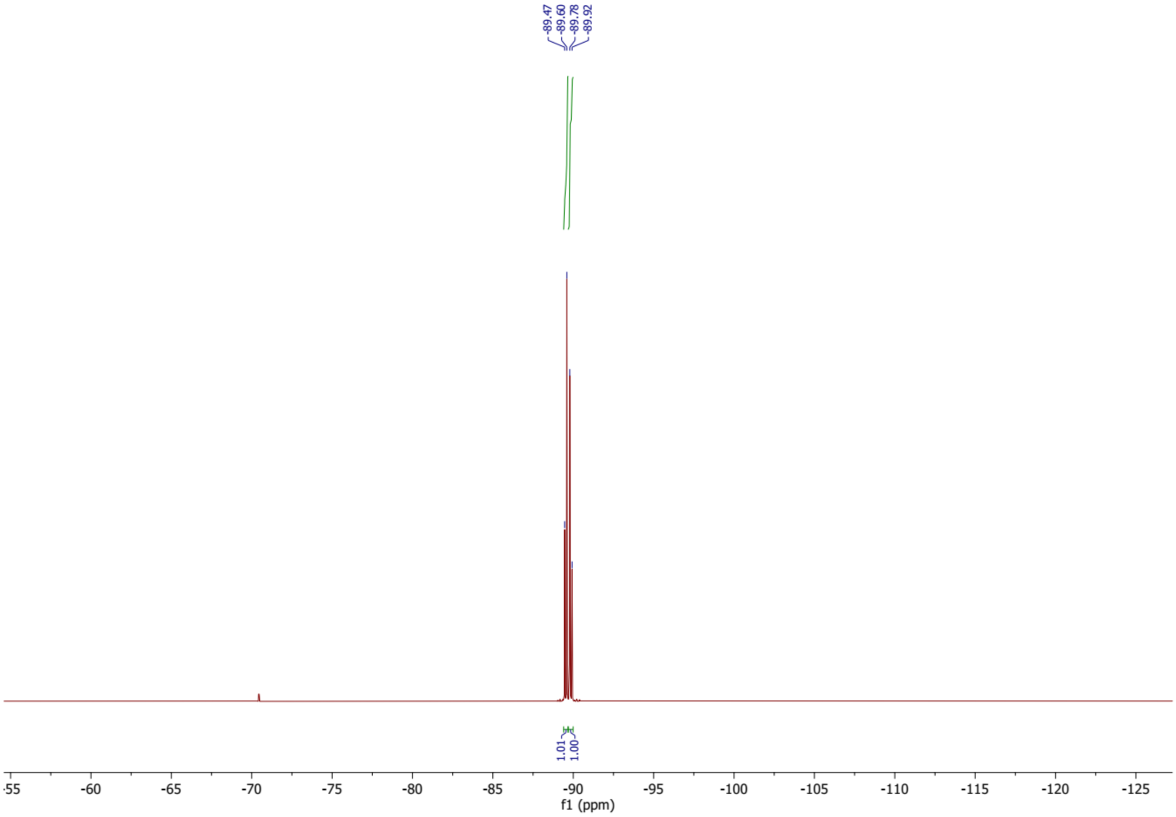
-89.47
-89.60
-89.78
-89.92
1.01
1.00
f1 (ppm)

S1s

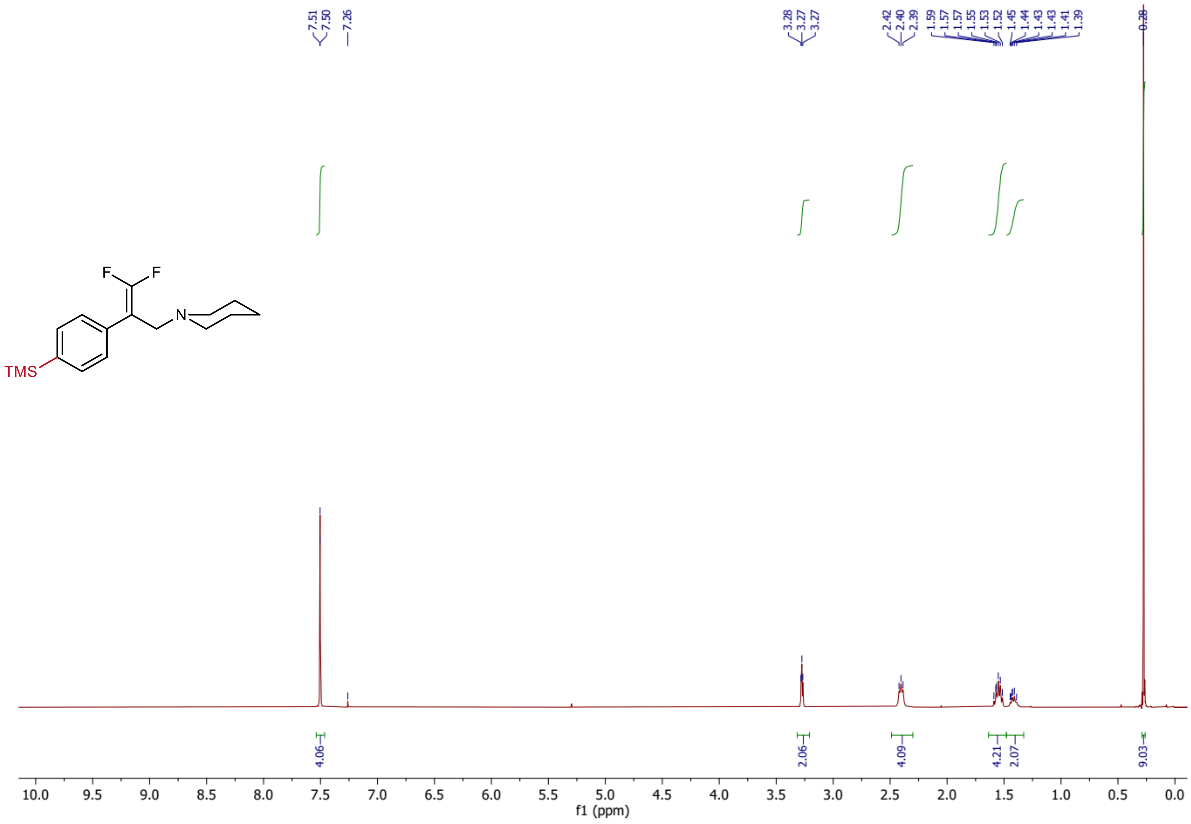
7.51
7.50
7.26
3.28
3.27
3.27
2.42
2.40
2.39
F
F
N
TMS
4.06
2.06
4.09
4.21
2.07
9.03
f1 (ppm)

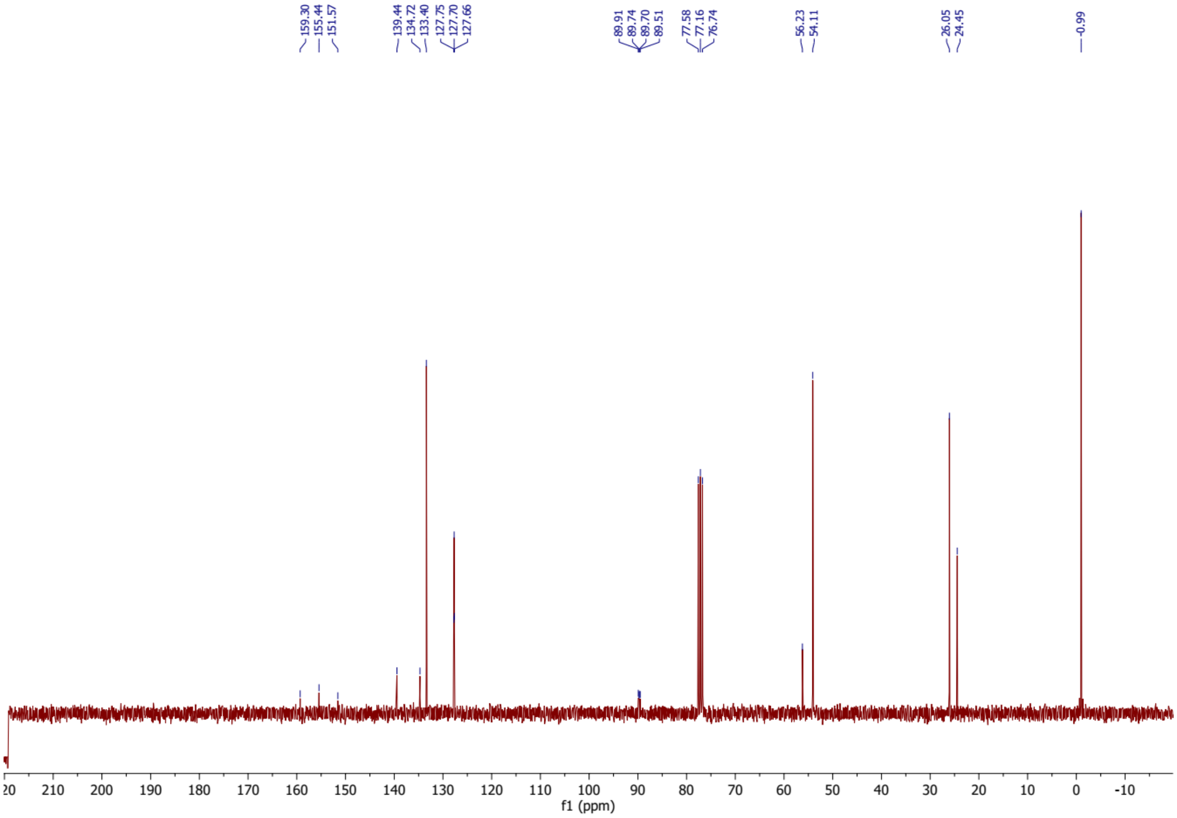
159.30
155.44
151.57
139.44
134.72
133.40
127.75
127.70
127.66
89.91
89.74
89.70
89.51
77.58
77.16
76.74
56.23
54.11
26.05
24.45
-0.99
f1 (ppm)

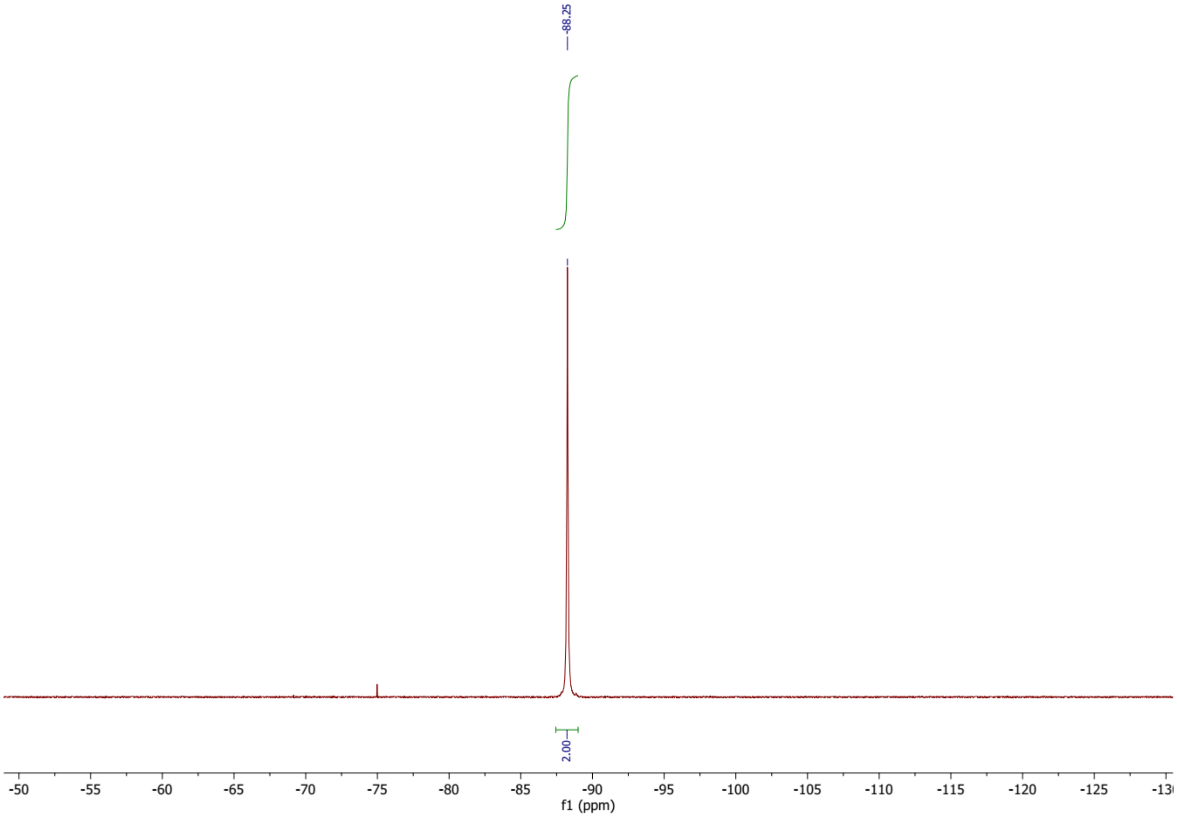
-88.25
2.00
f1 (ppm)

S1t

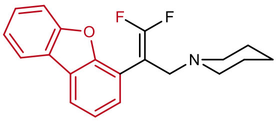
F
F
O
N

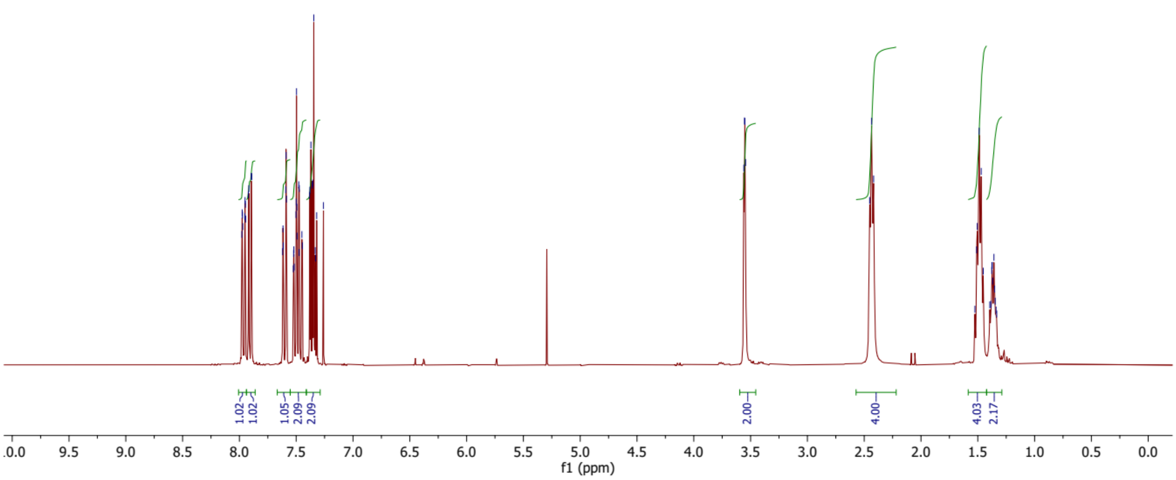
7.98
7.98
7.97
7.95
7.95
7.95
7.95
7.92
7.92
7.90
7.89
7.62
7.62
7.61
7.59
7.59
7.59
7.52
7.52
7.52
7.50
7.50
7.49
7.49
7.48
7.47
7.45
7.45
7.38
7.38
7.37
7.36
7.35
7.35
7.33
7.33
7.32
7.26
3.56
3.55
3.55
3.54
2.45
2.43
2.42
1.53
1.51
1.51
1.49
1.47
1.45
1.39
1.39
1.38
1.38
1.37
1.37
1.36
1.35
1.35
1.34
1.33
1.02
1.02
1.05
2.09
2.09
2.00
4.00
4.03
2.17
f1 (ppm)

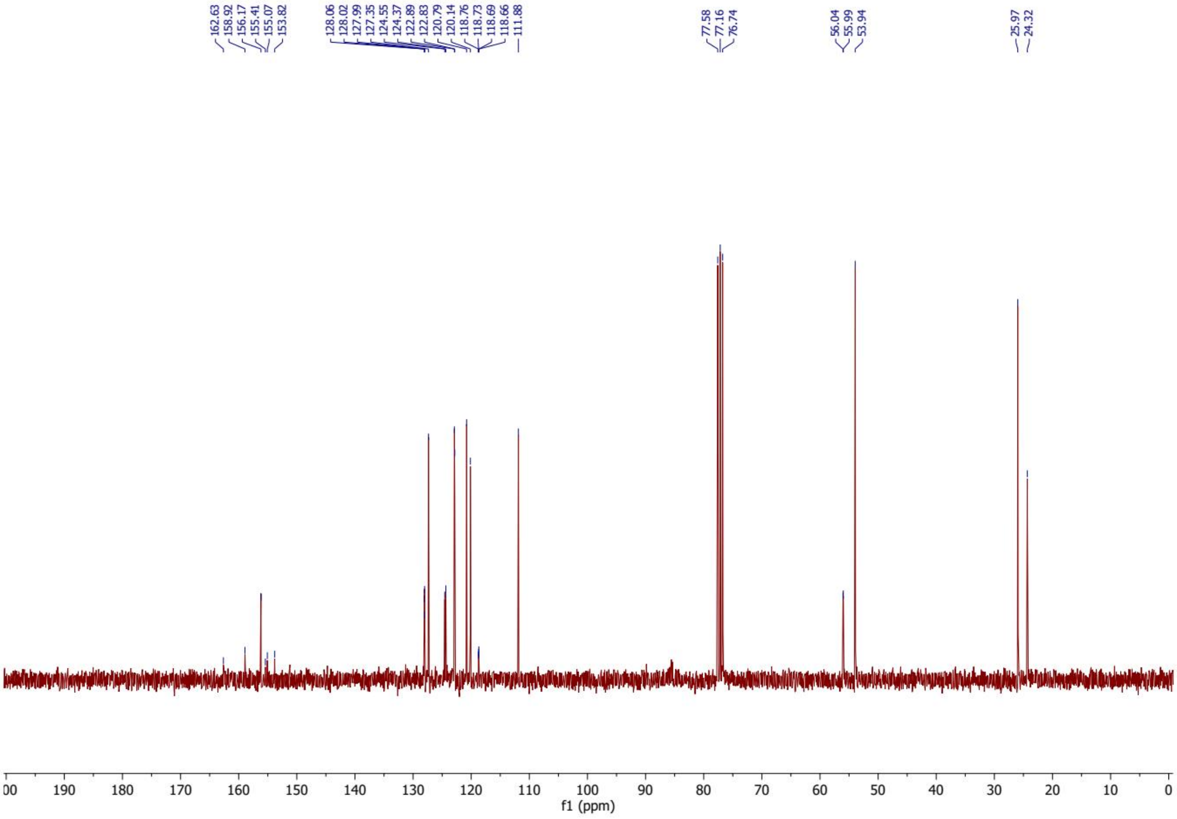
162.63
158.92
156.17
155.41
155.07
153.82
128.06
128.02
127.99
127.35
124.55
124.37
122.89
122.83
120.79
120.14
118.76
118.73
118.69
118.66
111.88
77.58
77.16
76.74
56.04
55.99
53.94
25.97
24.32
f1 (ppm)

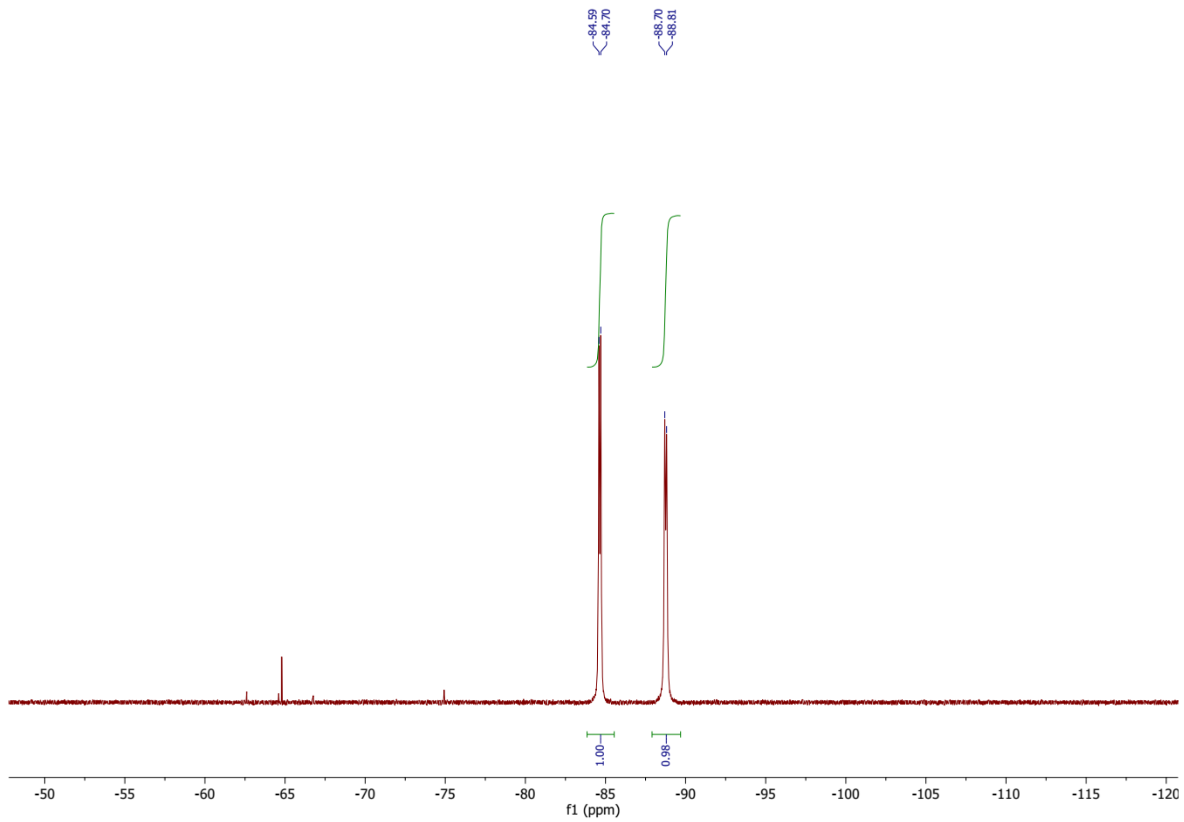
-84.59
-84.70
-88.70
-88.81
1.00
0.98
-50
-55
-60
-65
-70
-75
-80
-85
-90
-95
-100
-105
-110
-115
-120
f1 (ppm)

S1u

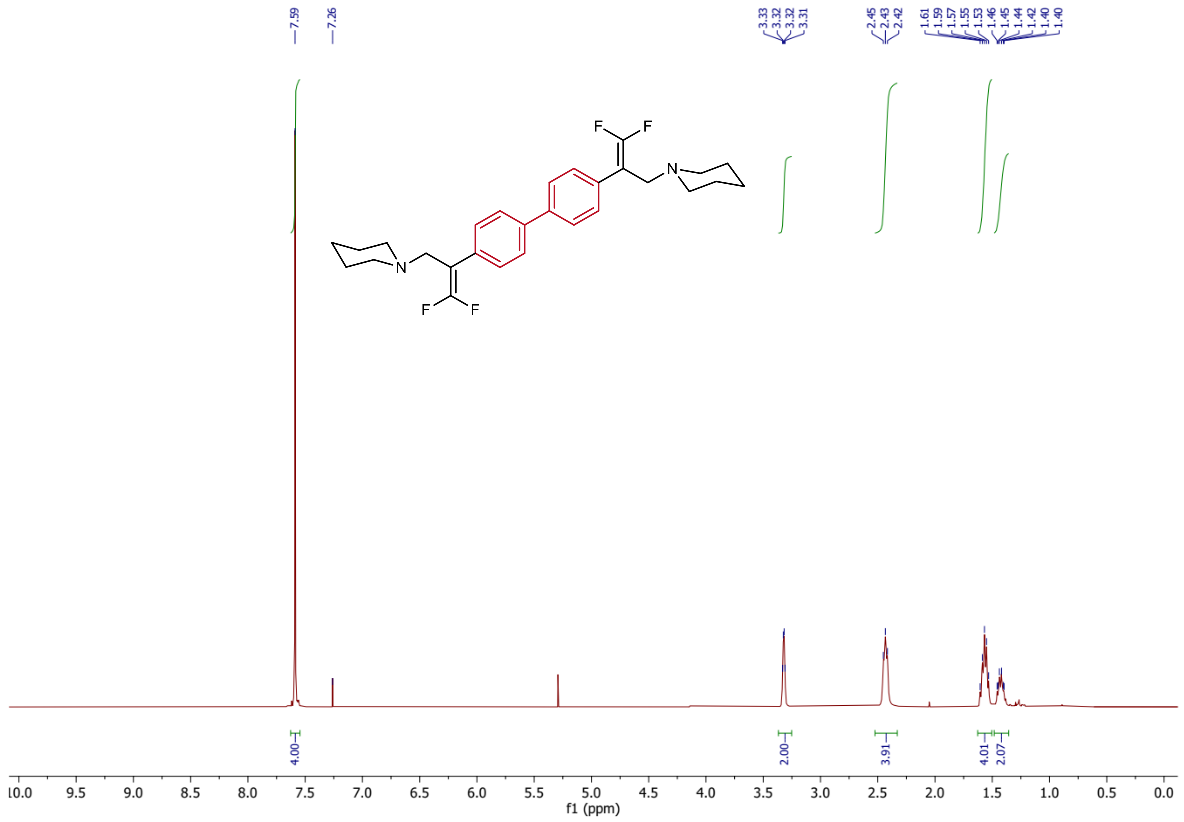
7.59
7.26
3.33
3.32
3.32
3.31
2.45
2.43
2.42
1.61
1.59
1.57
1.55
1.53
1.46
1.45
1.44
1.42
1.40
1.40
F
F
N
4.00
2.00
3.91
4.01
2.07
10.0
9.5
9.0
8.5
8.0
7.5
7.0
6.5
6.0
5.5
5.0
4.5
4.0
3.5
3.0
2.5
2.0
1.5
1.0
0.5
0.0
f1 (ppm)

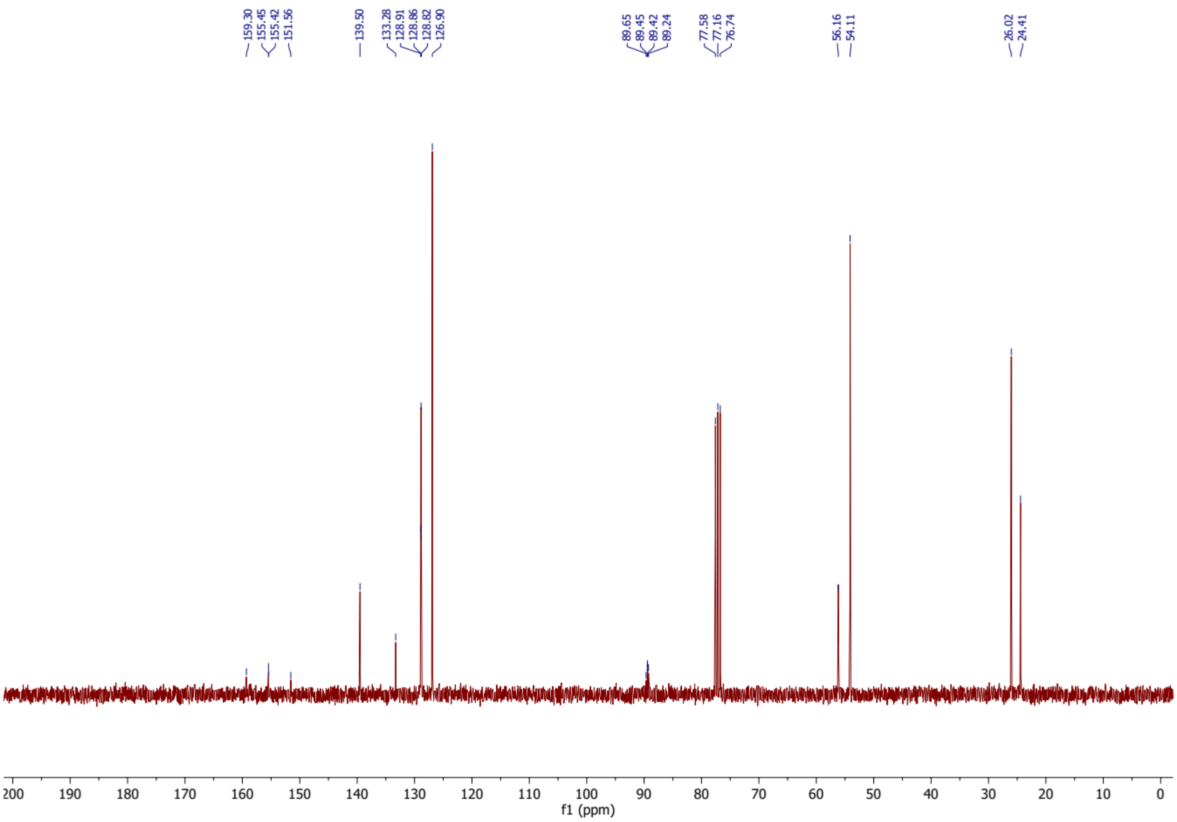
159.30
155.45
155.42
151.56
139.50
133.28
128.91
128.86
128.82
126.90
89.65
89.45
89.42
89.24
77.58
77.16
76.74
56.16
54.11
26.02
24.41
200 190 180 170 160 150 140 130 120 110 100 90 80 70 60 50 40 30 20 10 0
f1 (ppm)

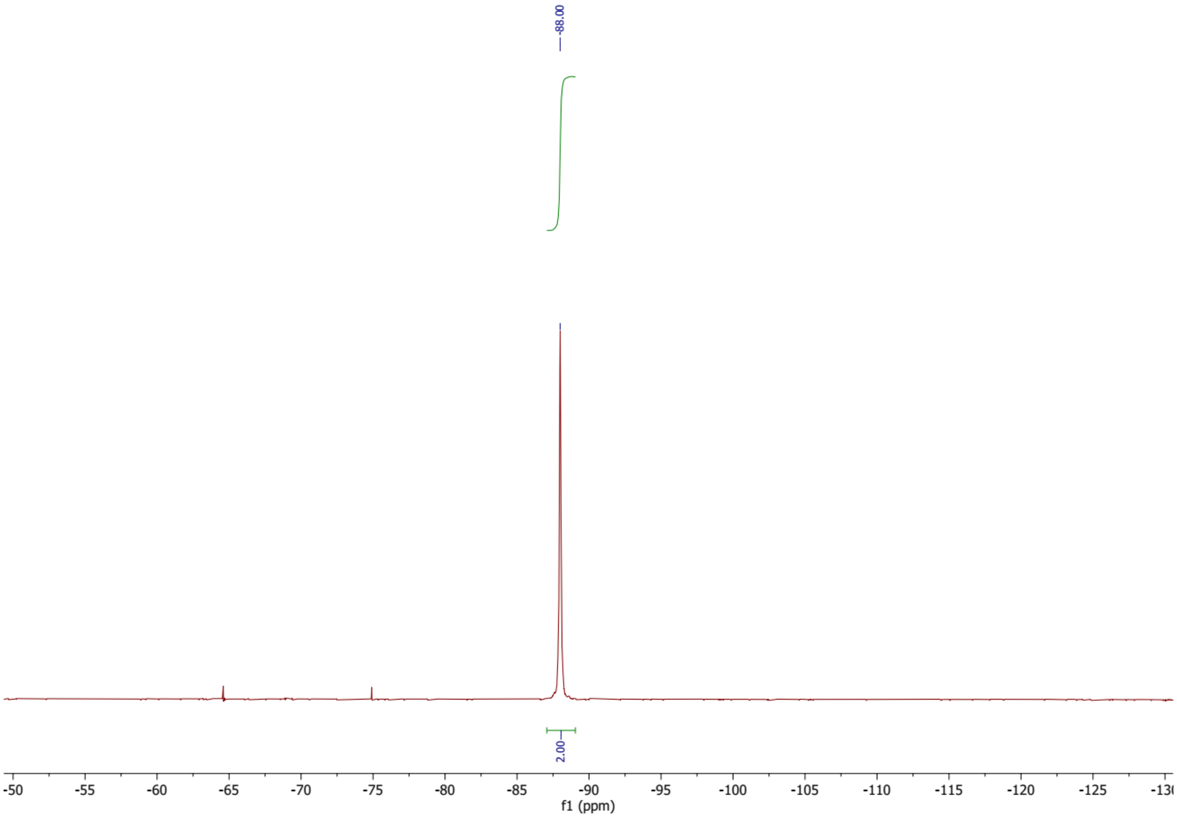
-88.00
2.00
-50 -55 -60 -65 -70 -75 -80 -85 -90 -95 -100 -105 -110 -115 -120 -125
f1 (ppm)

2d

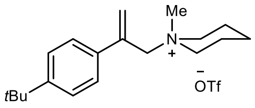

Me
N
+
−
OTf
tBu

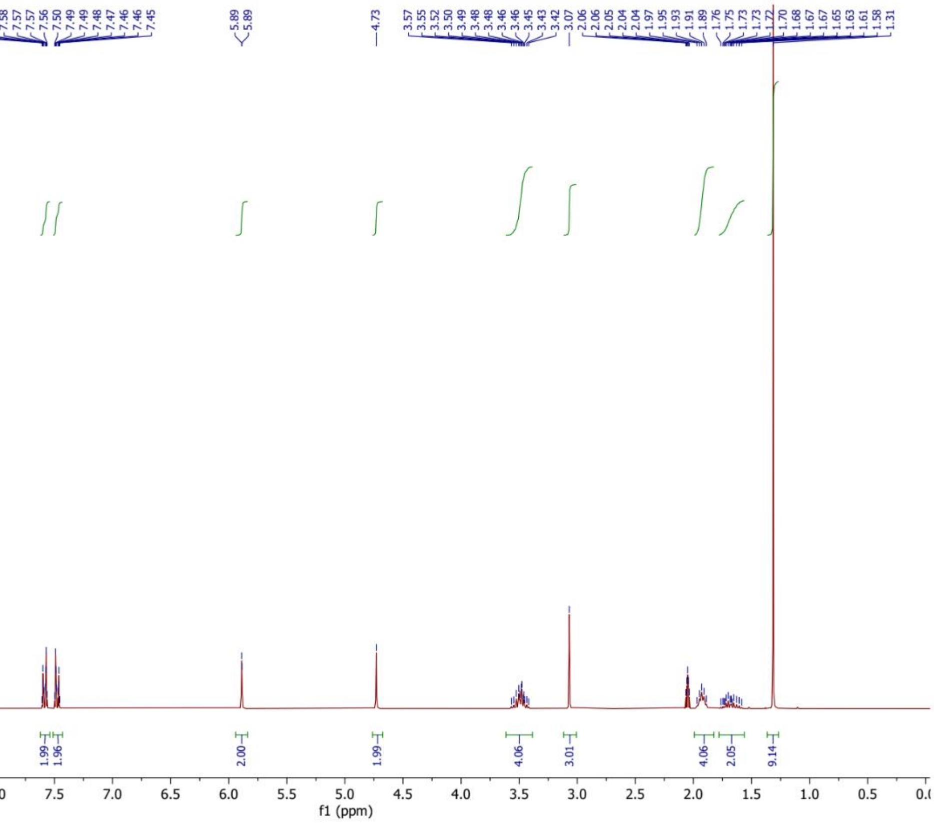

7.61 7.60 7.59 7.58 7.57 7.56 7.50 7.49 7.49 7.48 7.47 7.46 7.46 7.45
5.89 5.89
4.73
3.57 3.55 3.52 3.50 3.49 3.48 3.46 3.45 3.43 3.42
3.07
2.06 2.06 2.05 2.04 2.04 1.97 1.95 1.93 1.91 1.89 1.76 1.75 1.73 1.73 1.72 1.70 1.68 1.67 1.67 1.65 1.63 1.61 1.58 1.31
1.99 1.96 2.00 1.99 4.06 3.01 4.06 2.05 9.14
f1 (ppm)

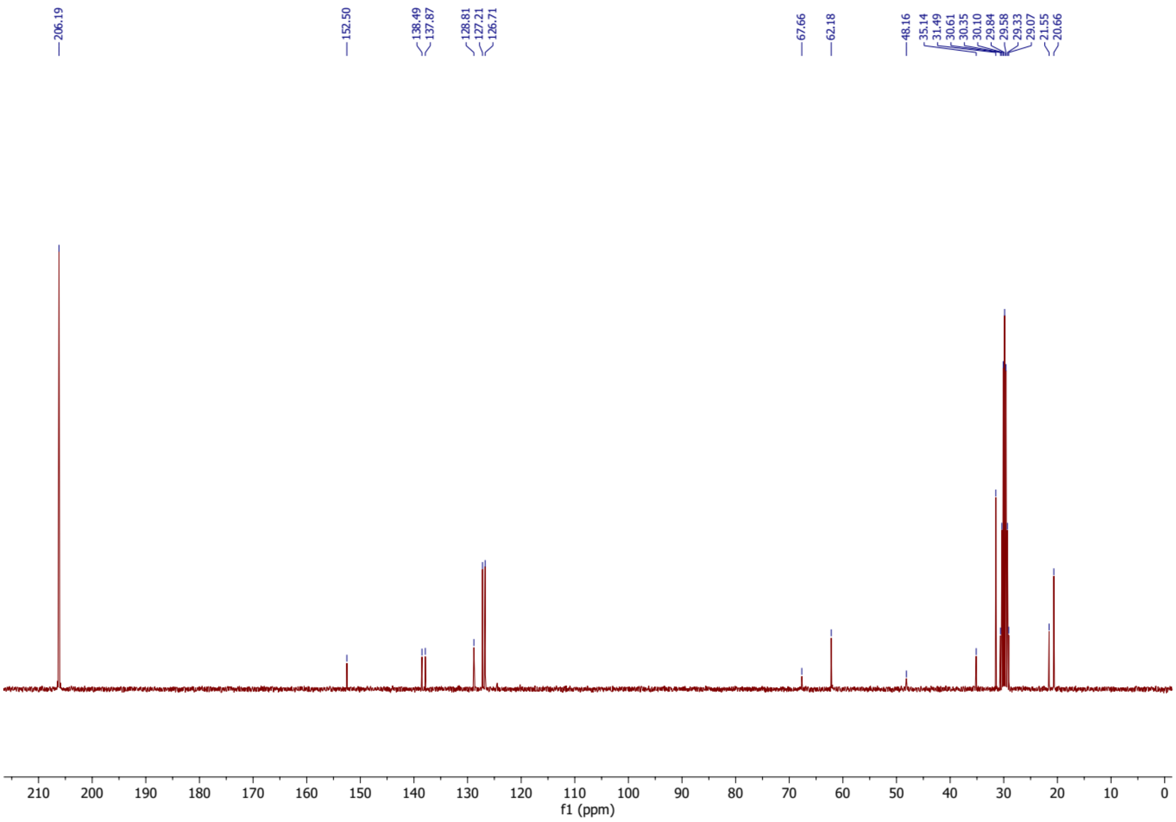

206.19
152.50
138.49 137.87
128.81 127.21 126.71
67.66
62.18
48.16 35.14 31.49 30.61 30.35 30.10 29.84 29.58 29.33 29.07 21.55 20.66
f1 (ppm)

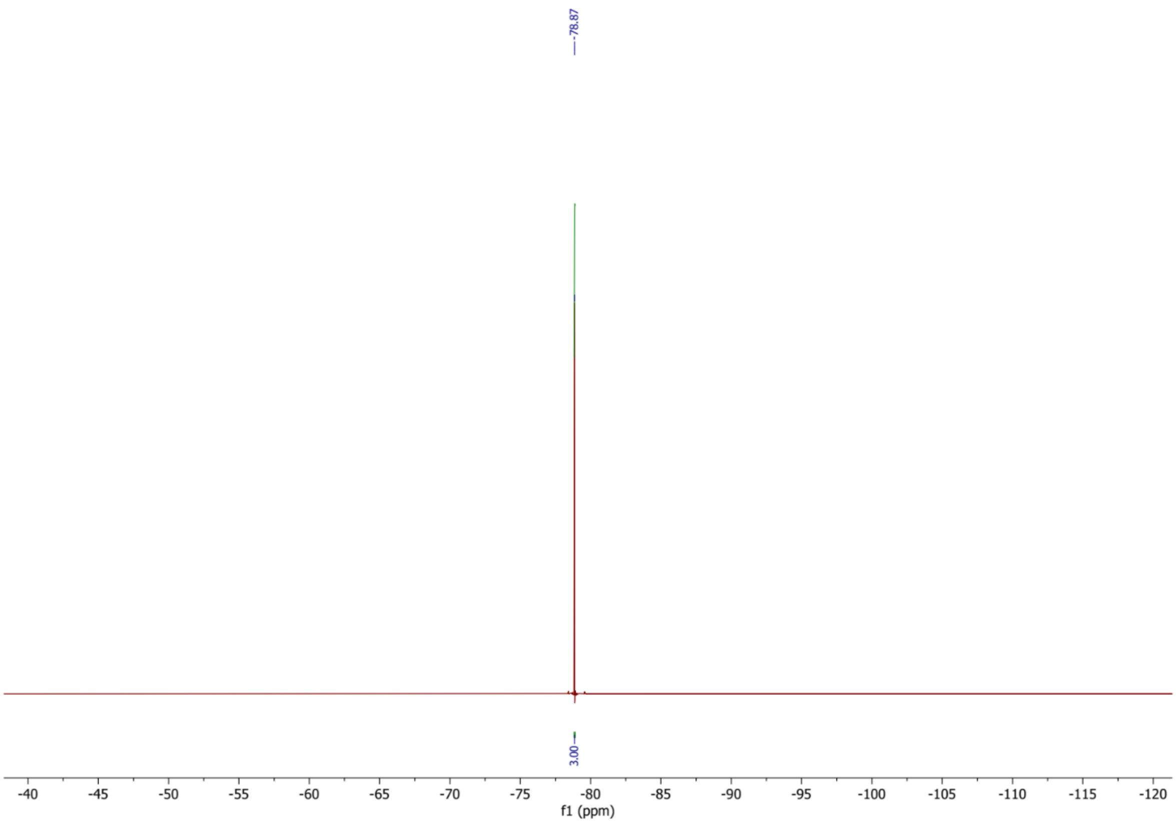
-78.87
3.00
f1 (ppm)

2f

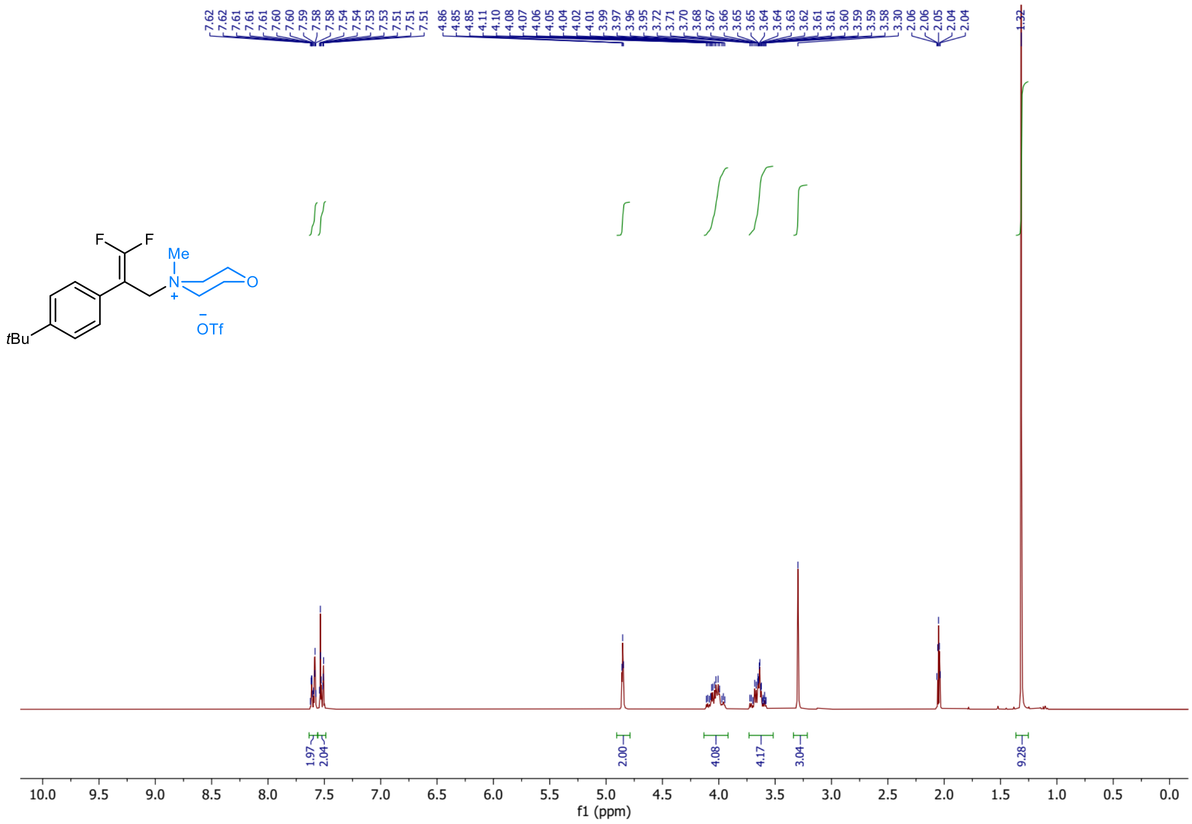
F
F
Me
N
O
OTf
tBu
1.97
2.04
2.00
4.08
4.17
3.04
9.28
f1 (ppm)

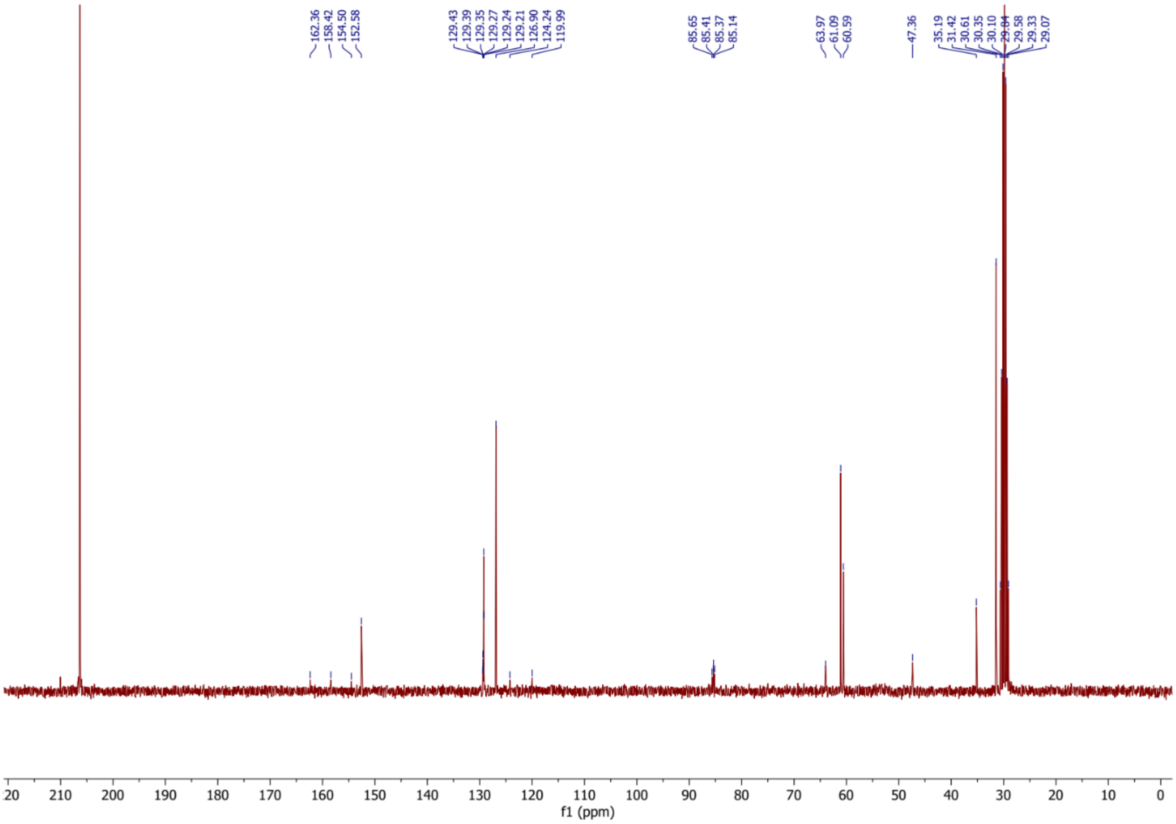
162.36
158.42
154.50
152.58
129.43
129.39
129.35
129.27
129.24
129.21
126.90
124.24
119.99
85.65
85.41
85.37
85.14
63.97
61.09
60.59
47.36
35.19
31.42
30.61
30.35
30.10
29.84
29.58
29.33
29.07
f1 (ppm)

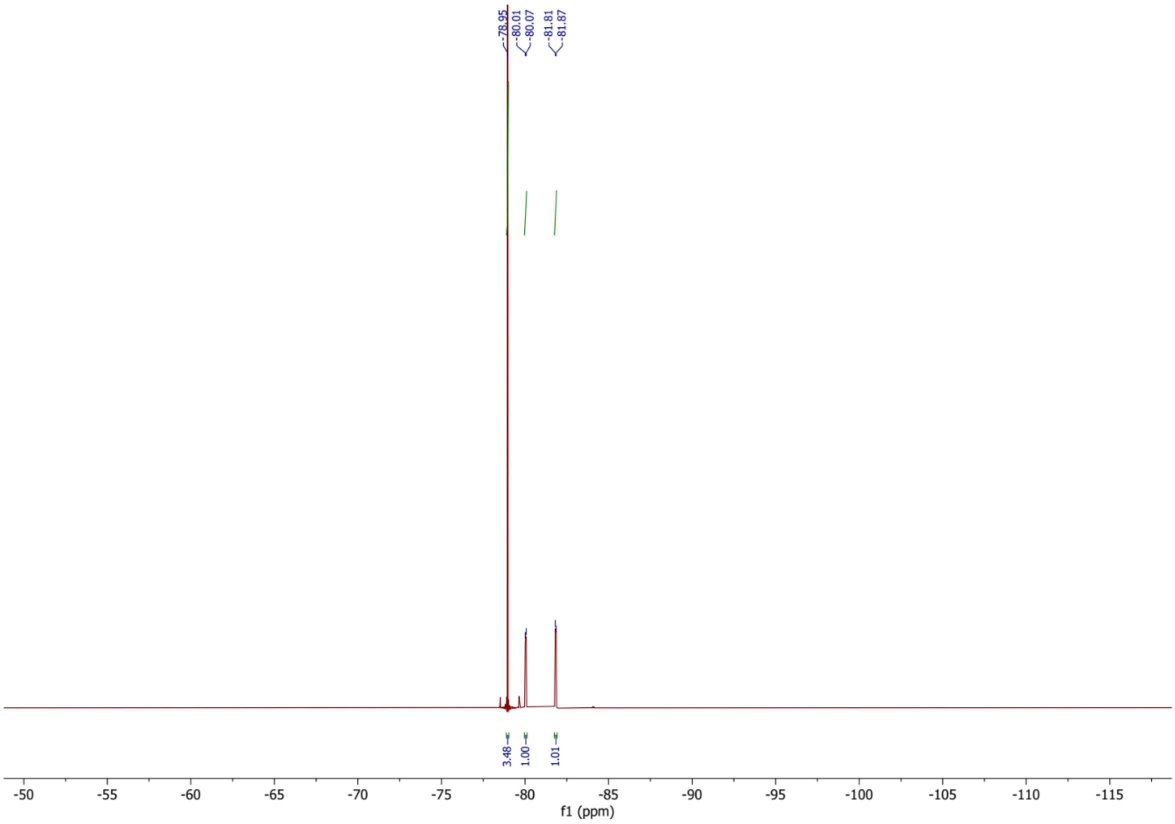
-78.95
-80.01
-80.07
-81.81
-81.87
3.48
1.00
1.01
f1 (ppm)

2g

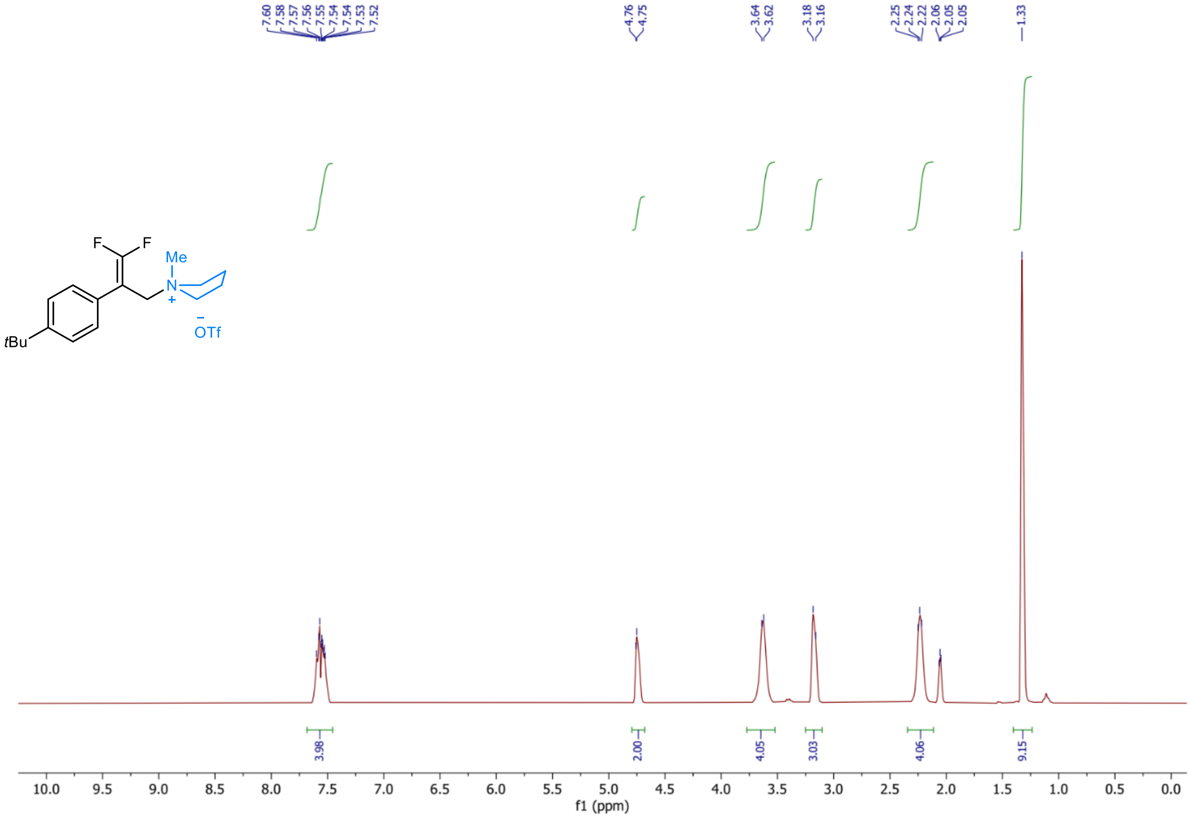
F
F
Me
N
+
–
OTf
tBu
7.60
7.58
7.57
7.56
7.55
7.54
7.54
7.54
7.53
7.52
4.76
4.75
3.64
3.62
3.18
3.16
2.25
2.24
2.22
2.06
2.05
2.05
1.33
3.98
2.00
4.05
3.03
4.06
9.15
f1 (ppm)

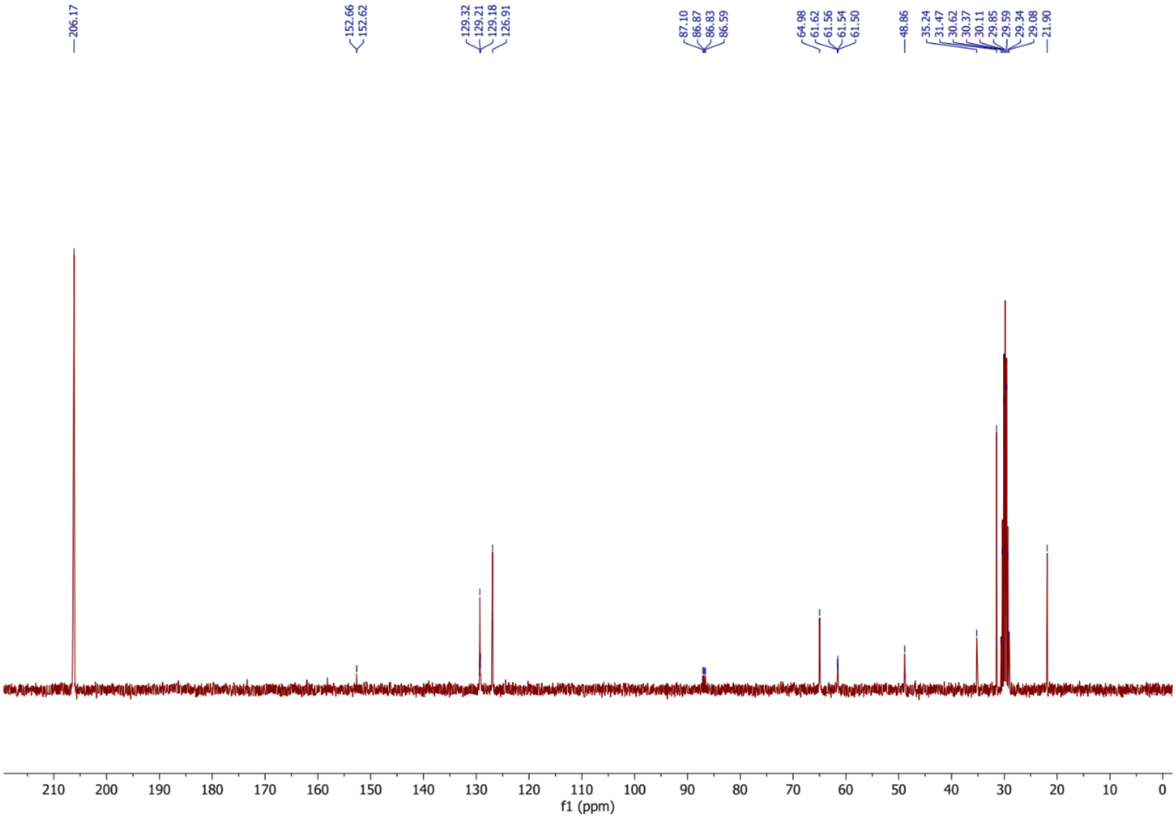
206.17
152.66
152.62
129.32
129.21
129.18
126.91
87.10
86.87
86.83
86.59
64.98
61.62
61.56
61.54
61.50
48.86
35.24
31.47
30.62
30.37
30.11
29.85
29.59
29.34
29.08
21.90
f1 (ppm)

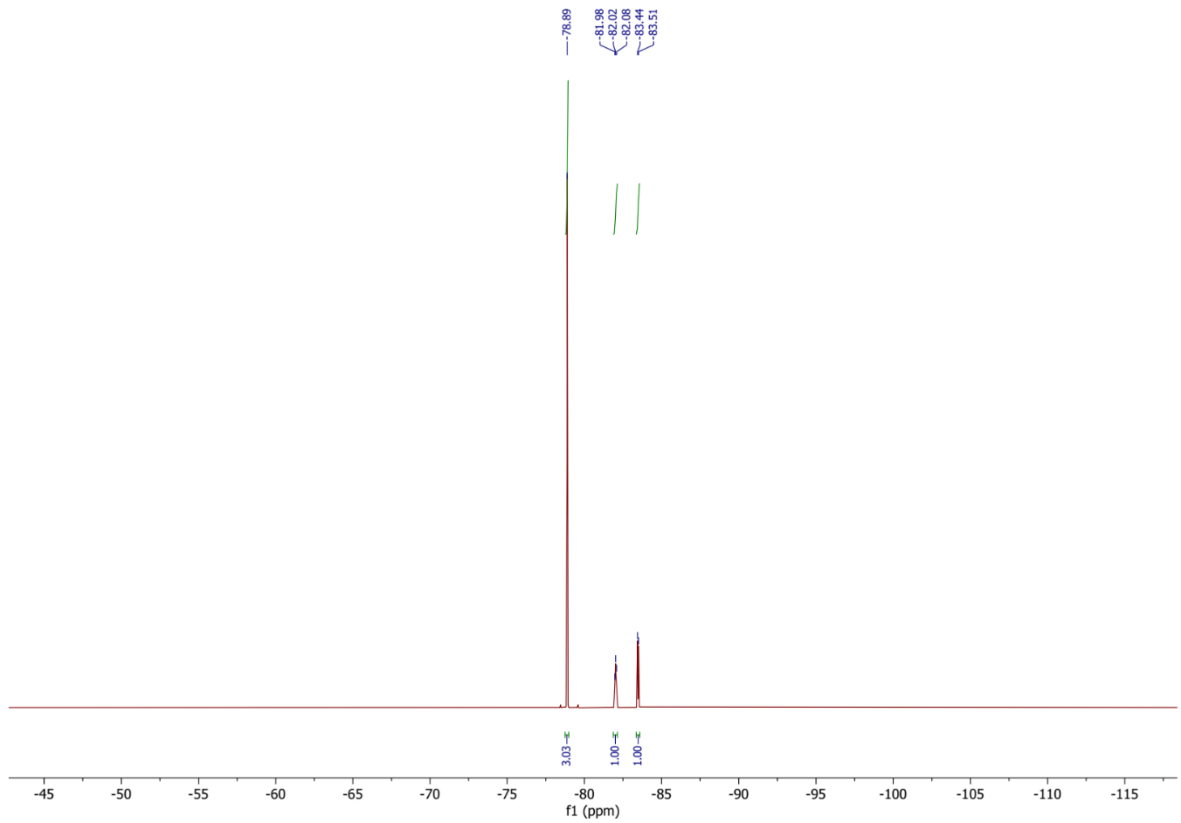

2h

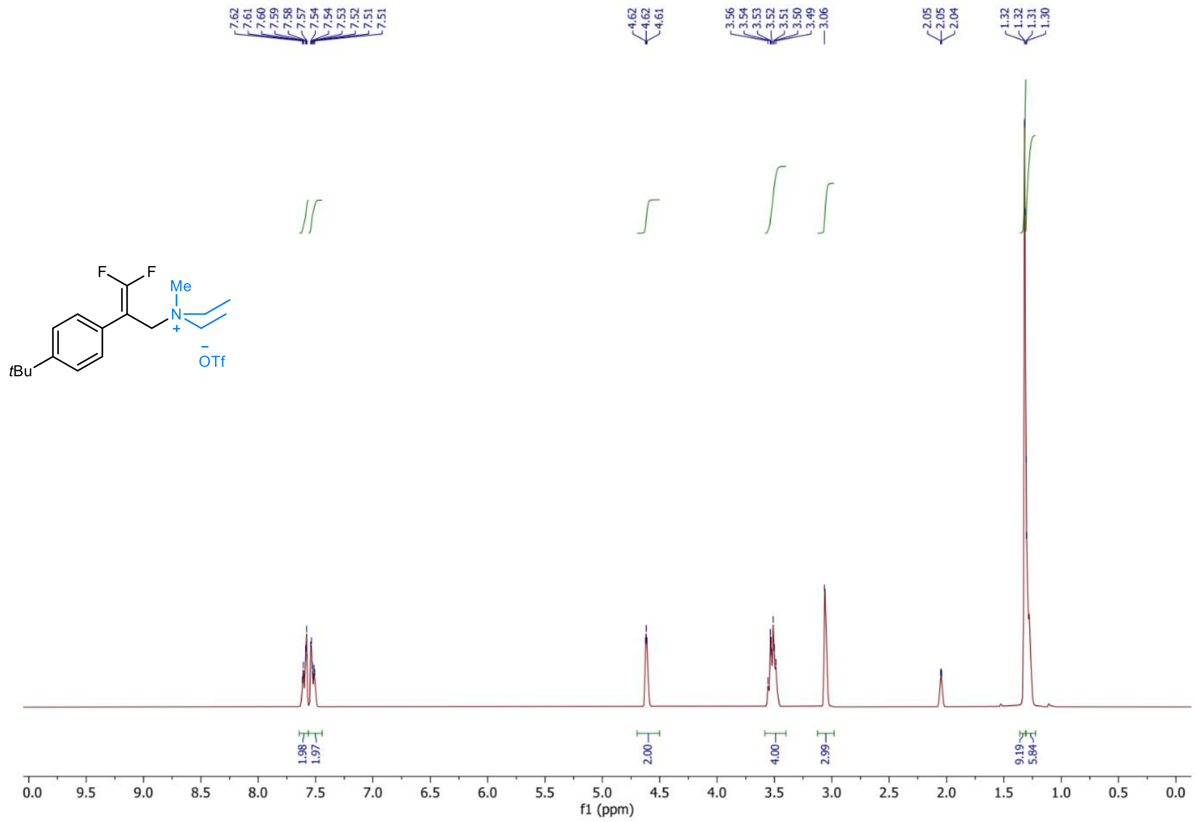

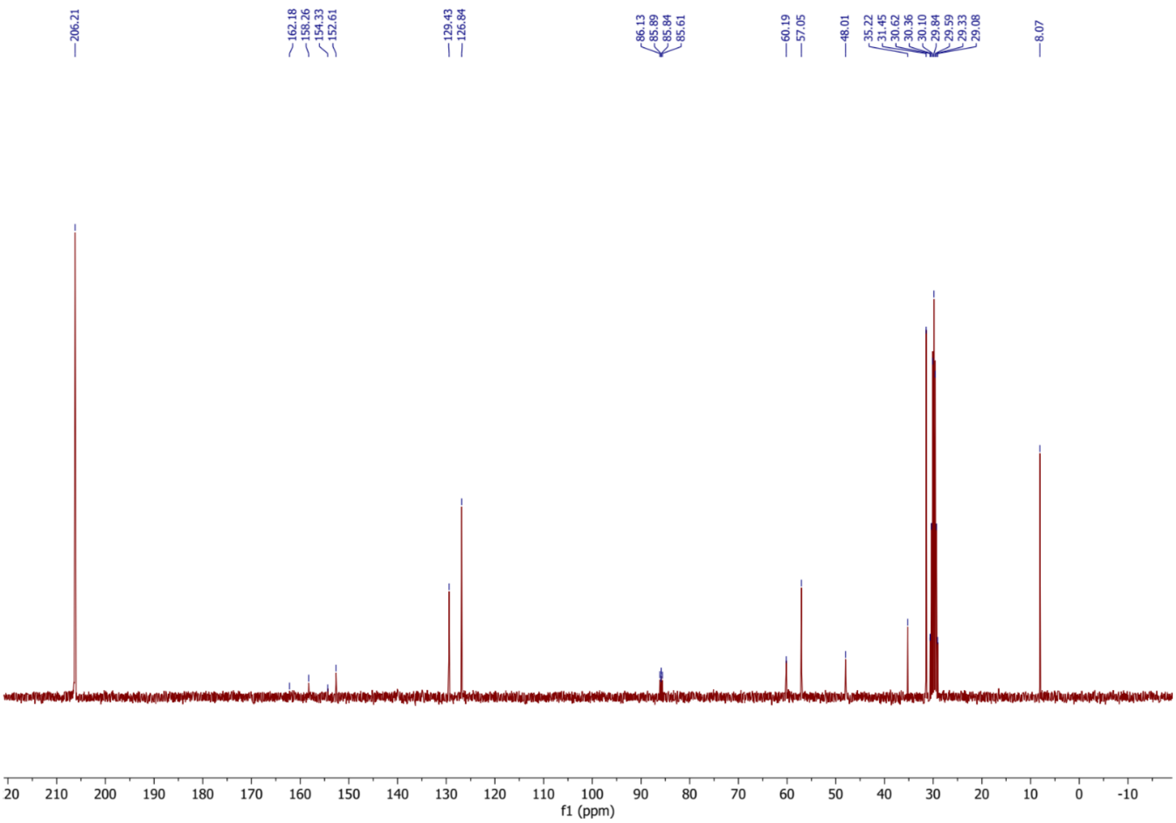
206.21
162.18
158.26
154.33
152.61
129.43
126.84
86.13
85.89
85.84
85.61
60.19
57.05
48.01
35.22
31.45
30.62
30.36
30.10
29.84
29.59
29.33
29.08
8.07
20 210 200 190 180 170 160 150 140 130 120 110 100 90 80 70 60 50 40 30 20 10 0 -10
f1 (ppm)

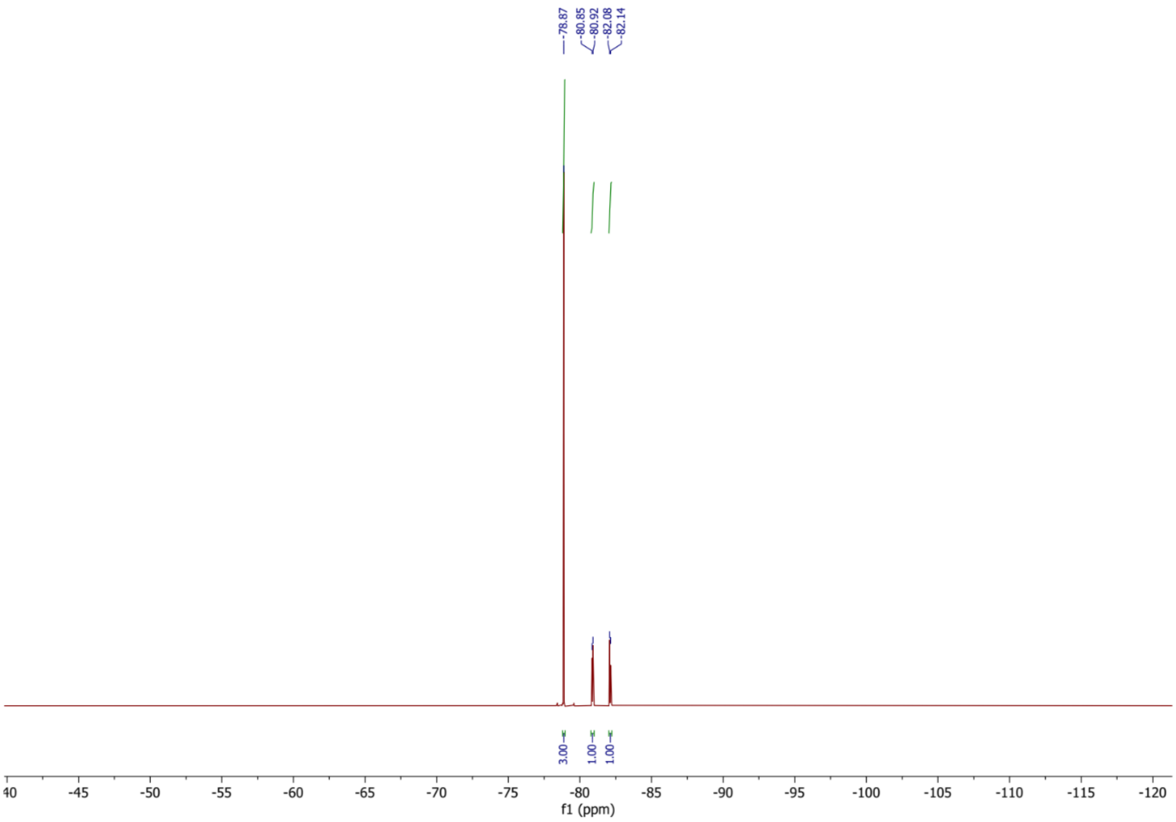
-78.87
-80.85
-80.92
-82.08
-82.14
3.00
1.00
1.00
40 -45 -50 -55 -60 -65 -70 -75 -80 -85 -90 -95 -100 -105 -110 -115 -120
f1 (ppm)

2i

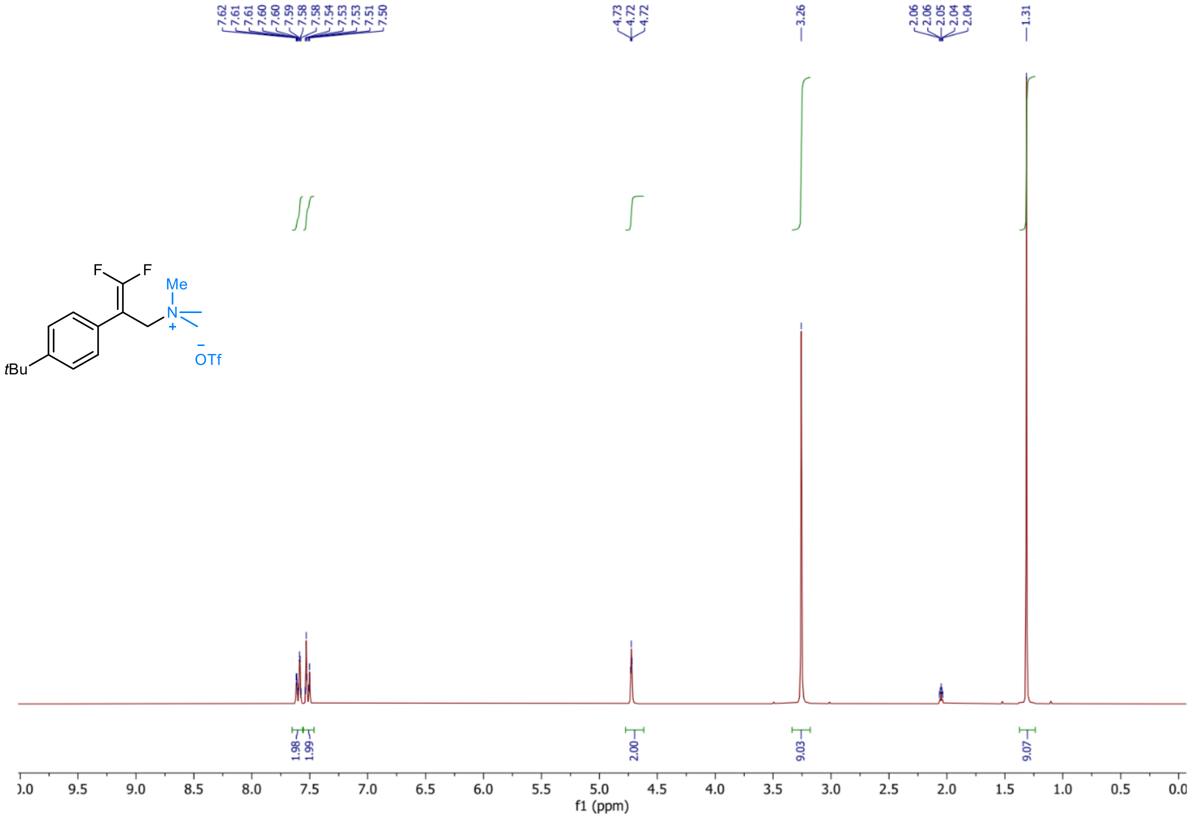

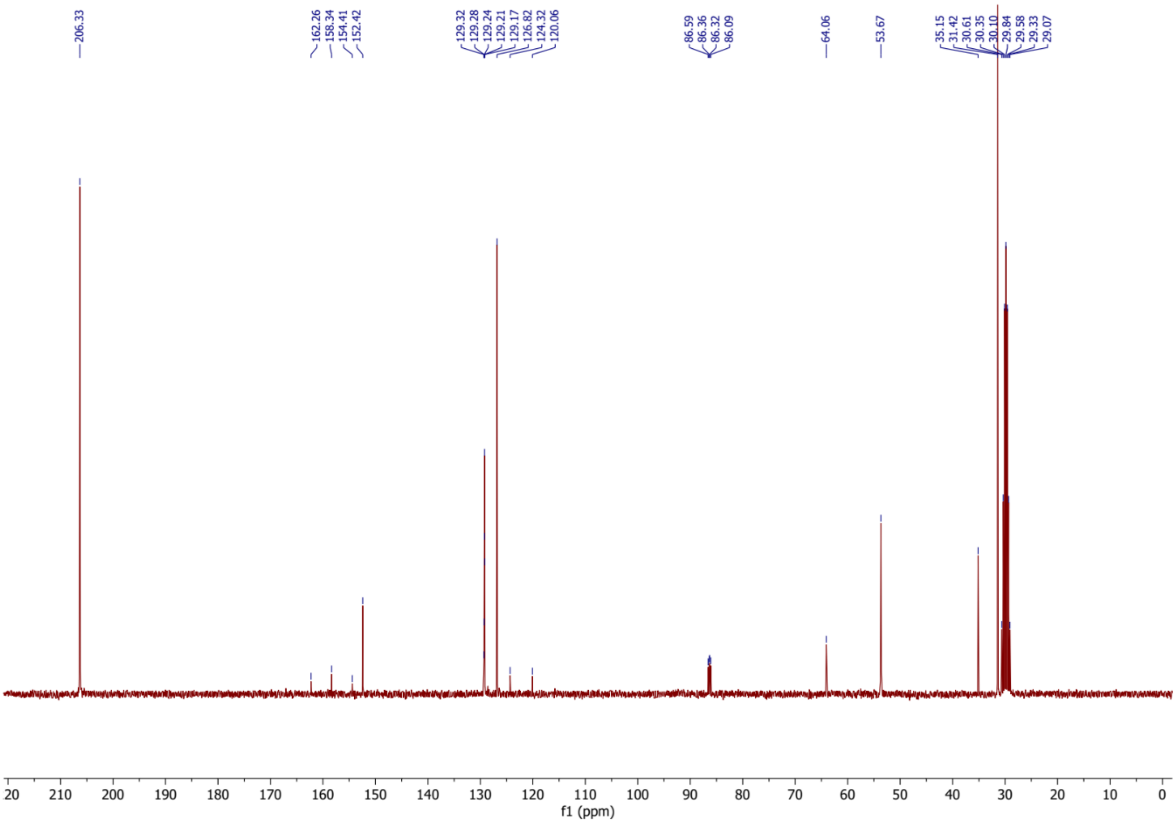

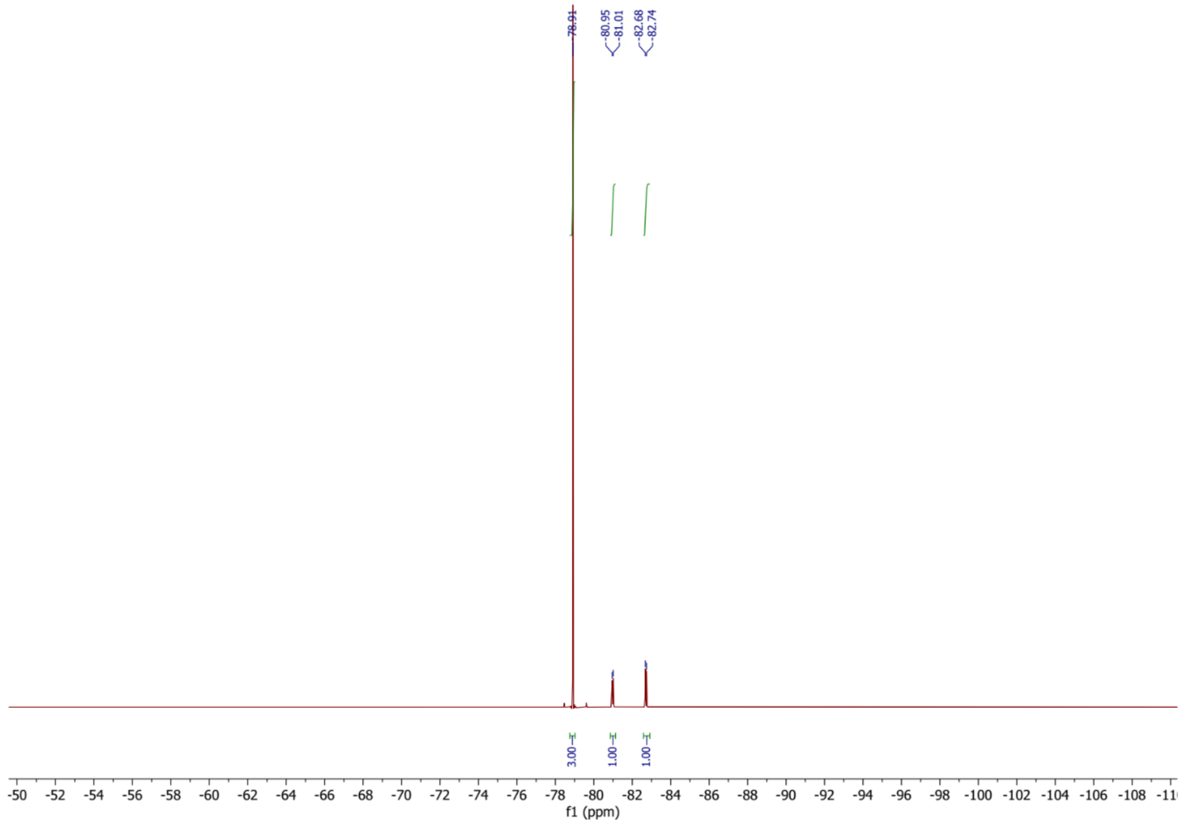

2j

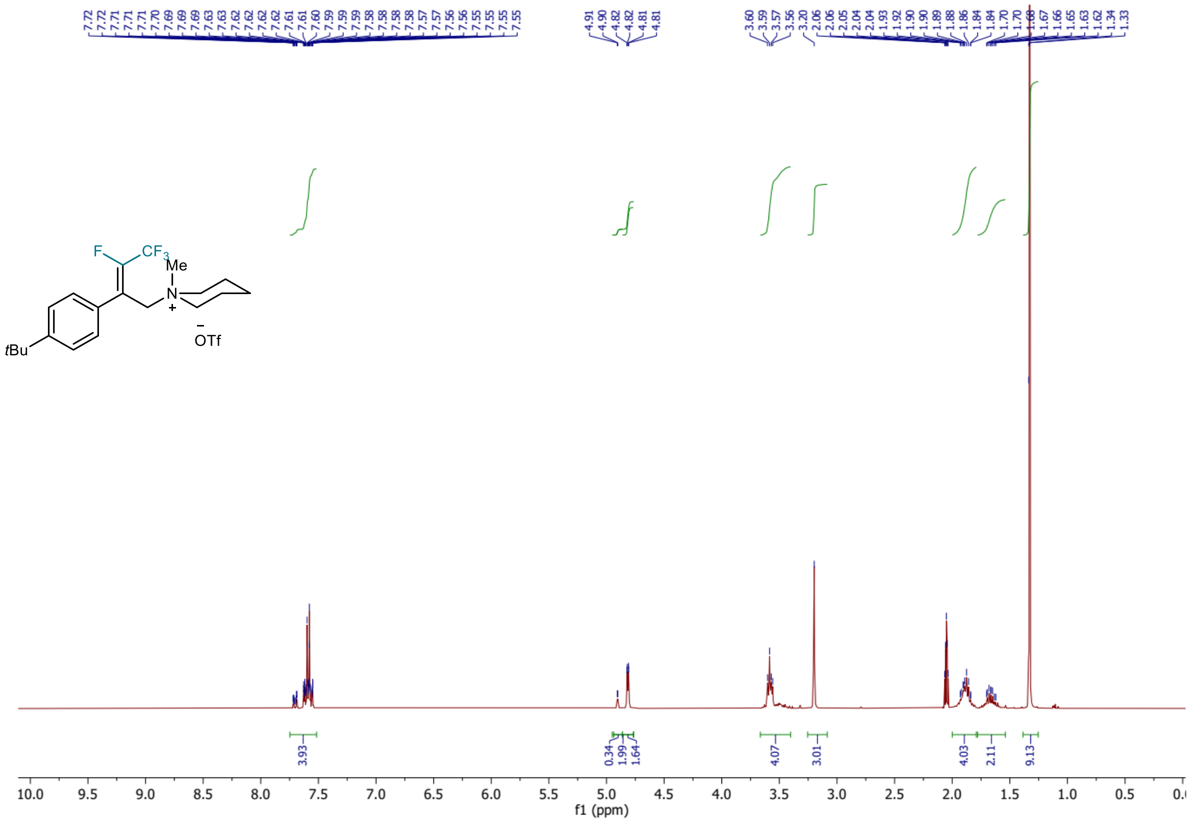

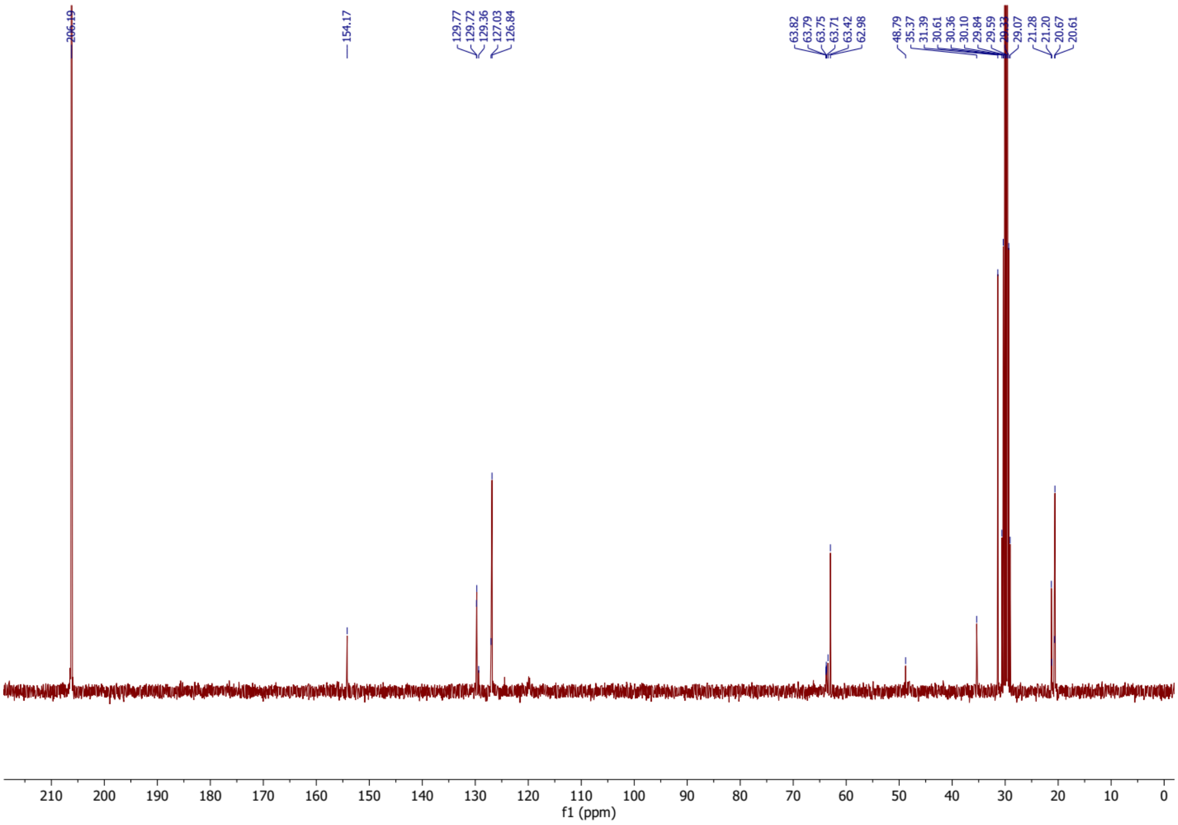
206.19
154.17
129.77
129.72
129.36
127.03
126.84
63.82
63.79
63.75
63.71
63.42
62.98
48.79
35.37
31.39
30.61
30.36
30.10
29.84
29.59
29.33
29.07
21.28
21.20
20.67
20.61
f1 (ppm)

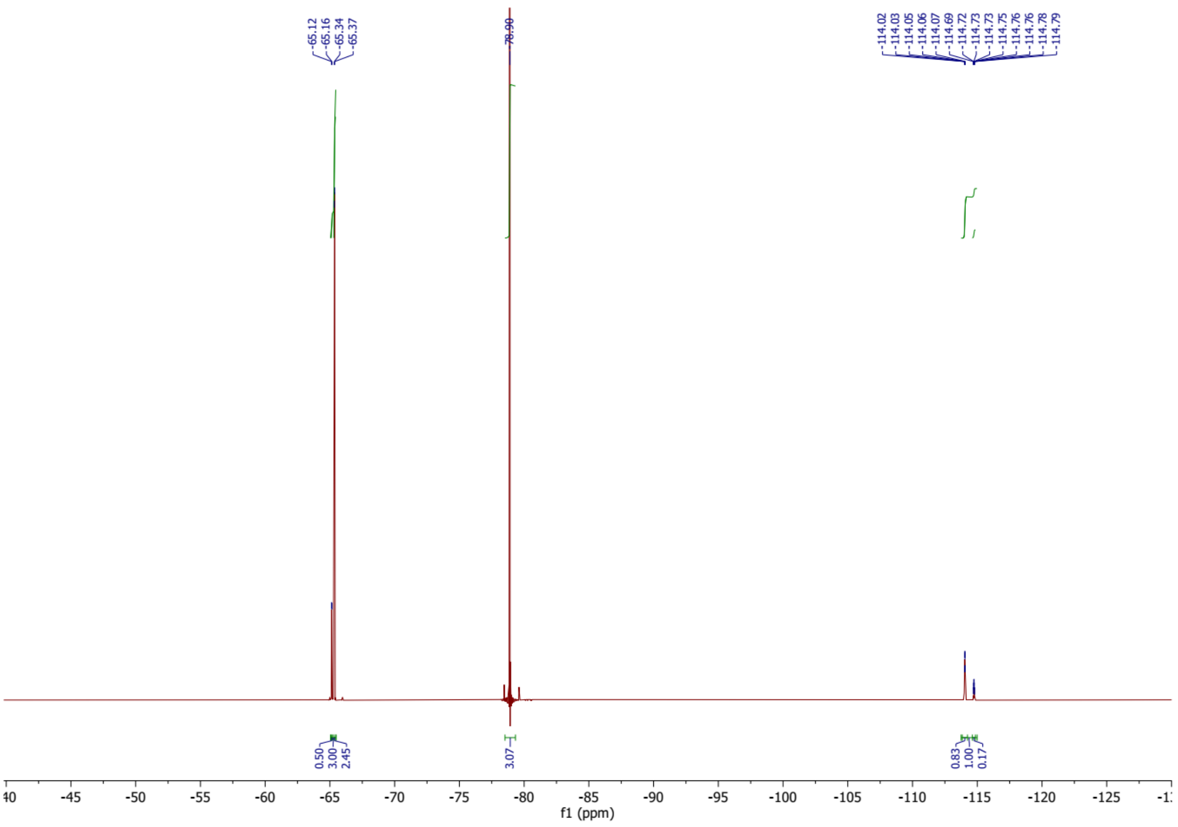
-65.12
-65.16
-65.34
-65.37
-78.90
-114.02
-114.03
-114.05
-114.06
-114.07
-114.69
-114.72
-114.73
-114.73
-114.75
-114.76
-114.76
-114.78
-114.79
0.50
3.00
2.45
3.07
0.83
1.00
0.17
f1 (ppm)

2n

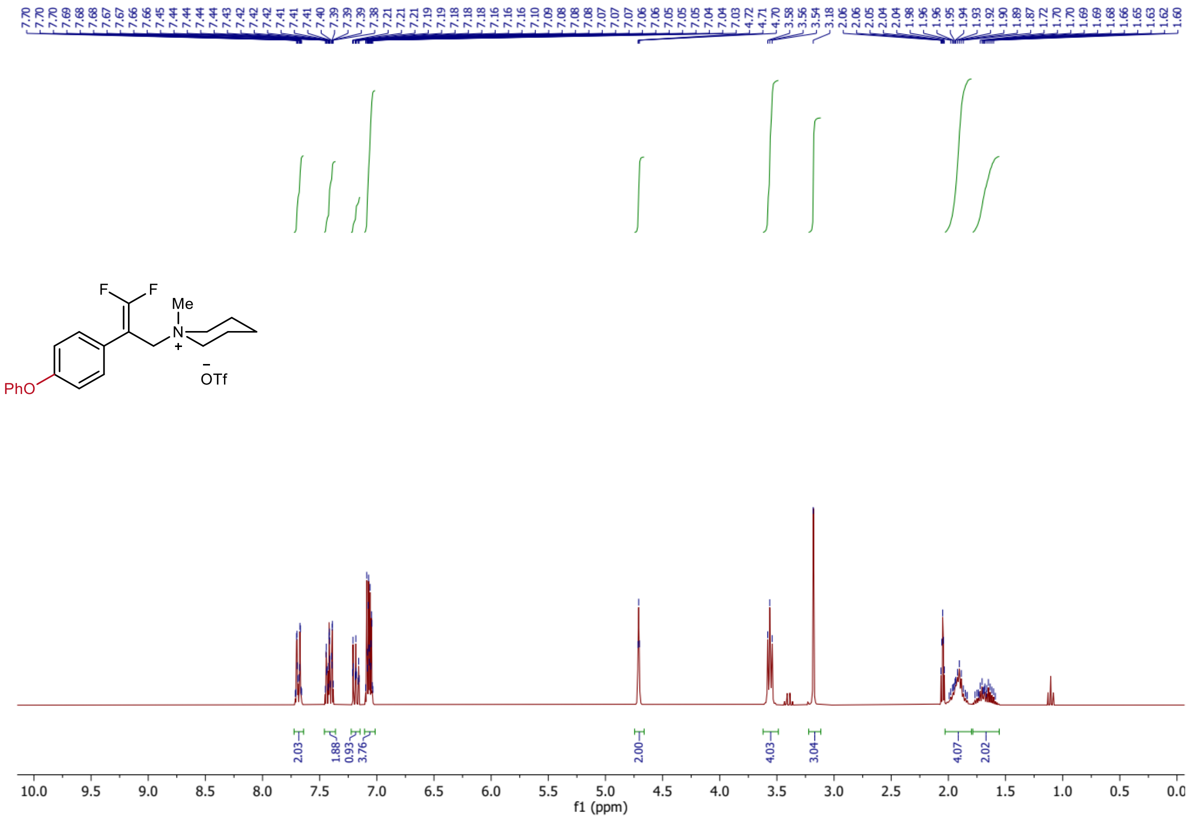

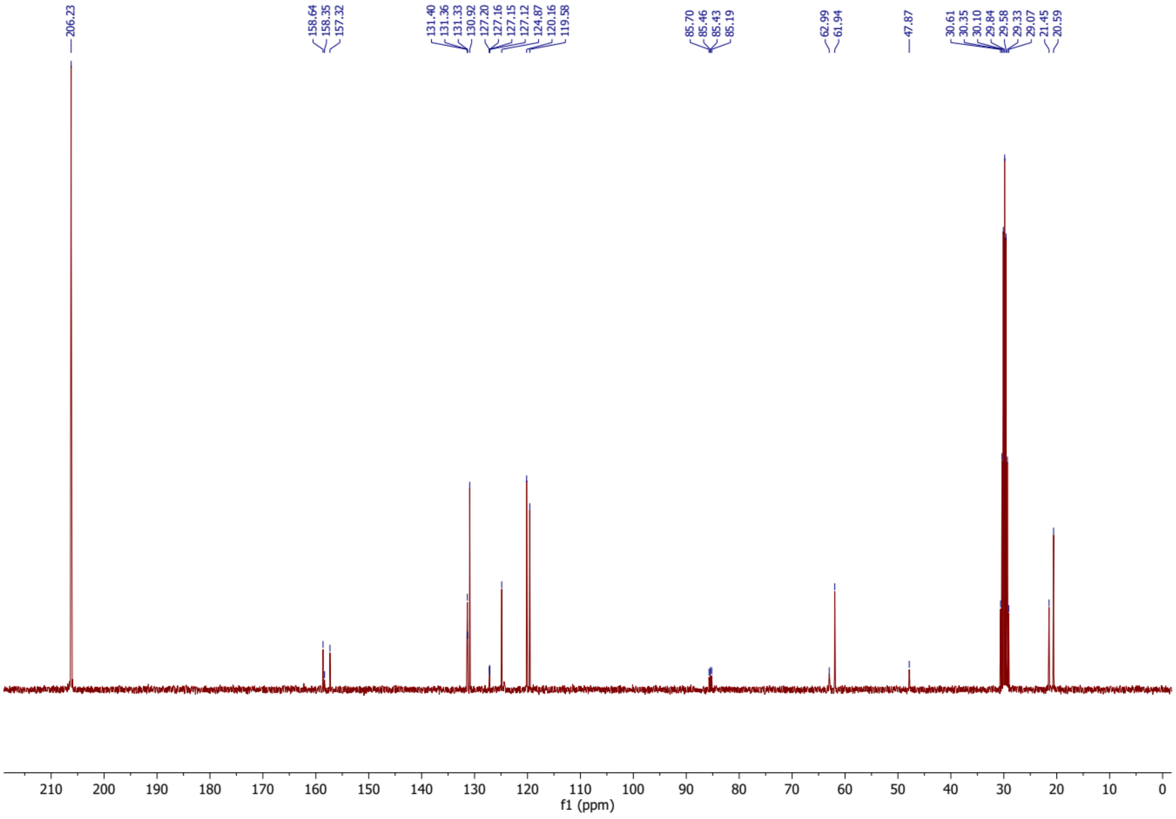

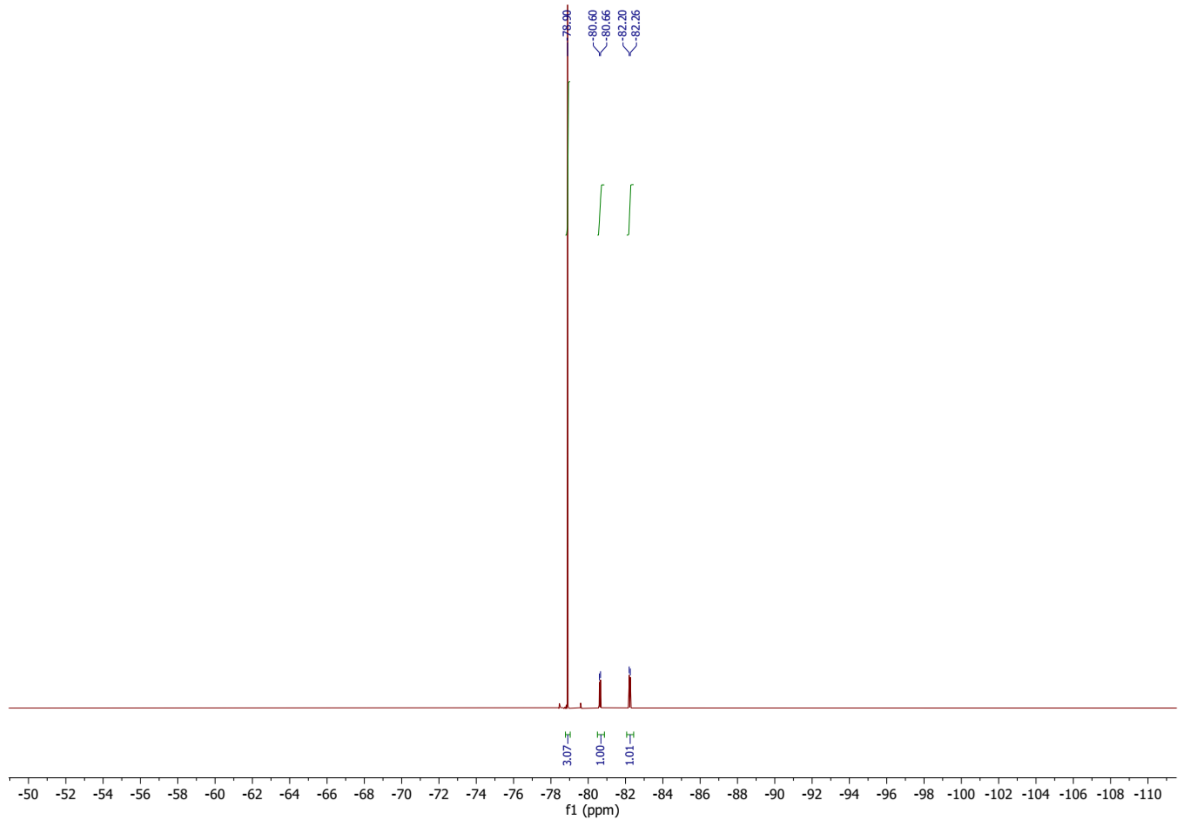

2o

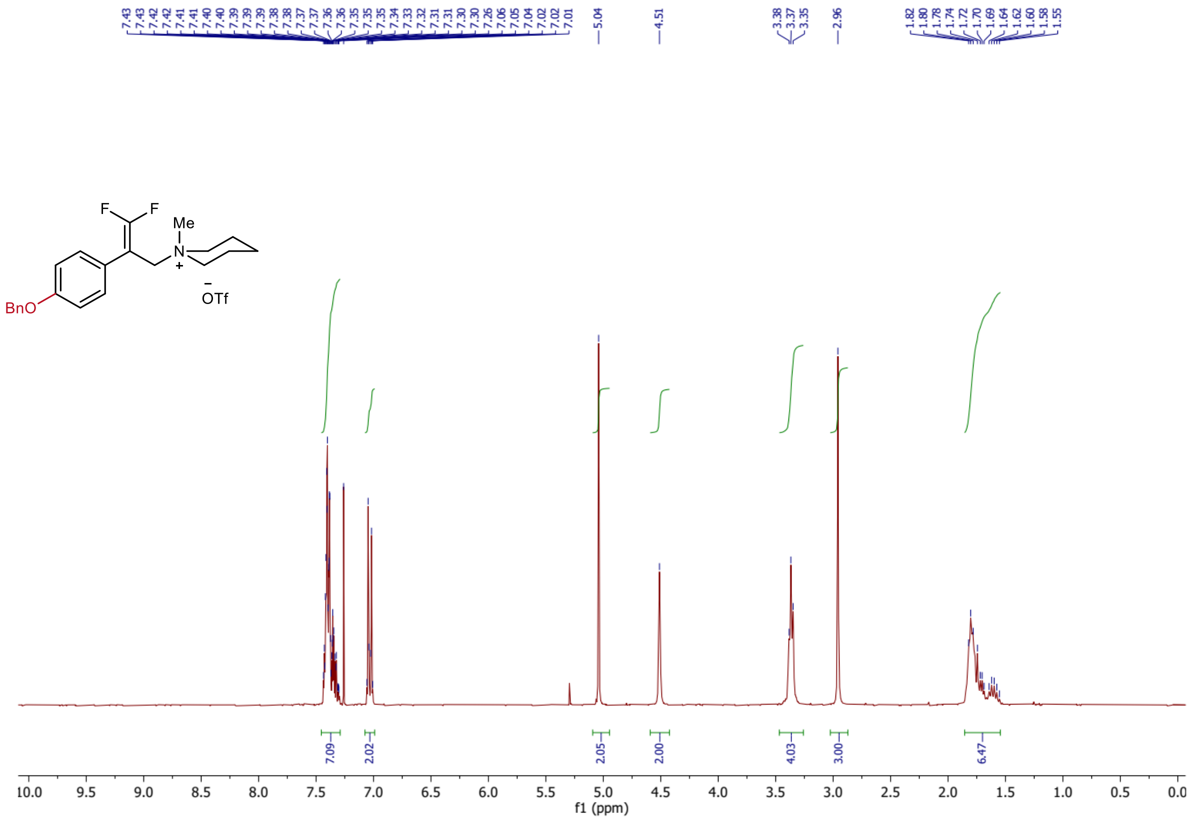

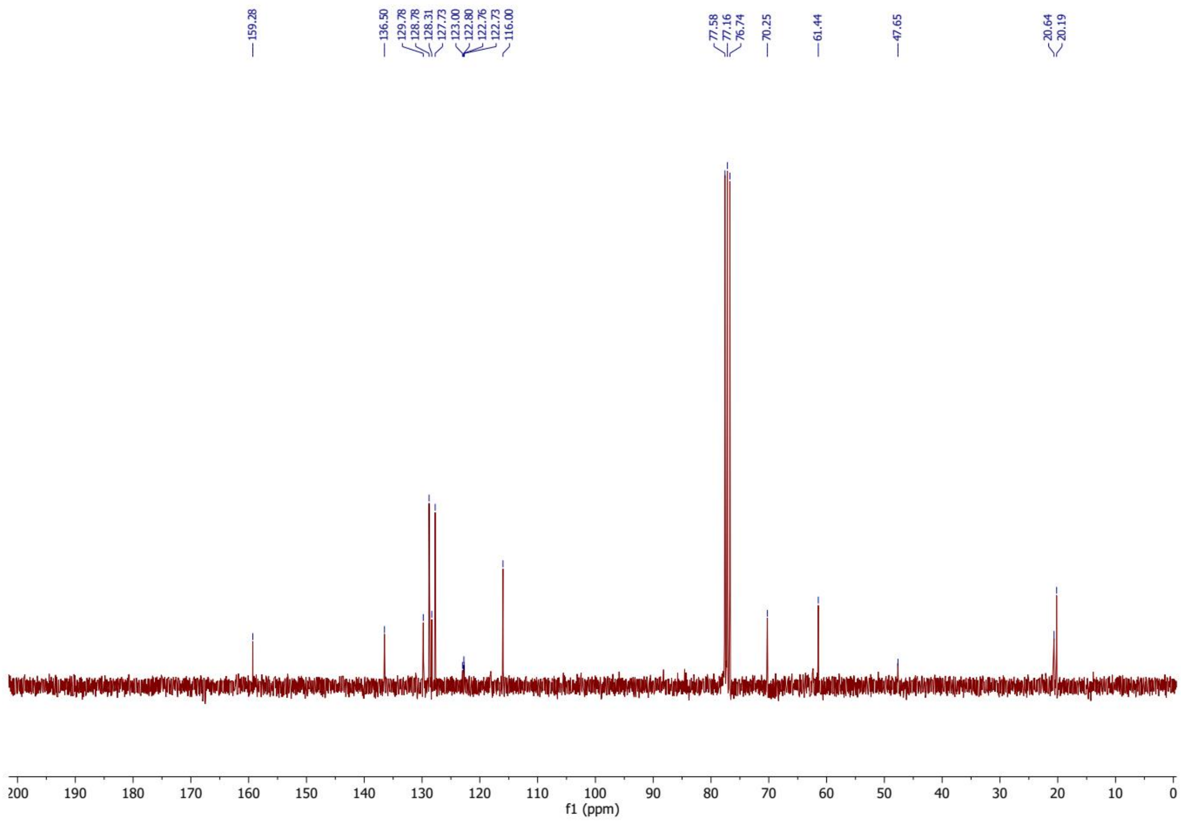
159.28
136.50
129.78
128.78
128.31
127.73
123.00
122.80
122.76
122.73
116.00
77.58
77.16
76.74
70.25
61.44
47.65
20.64
20.19
200
190
180
170
160
150
140
130
120
110
100
90
80
70
60
50
40
30
20
10
0
f1 (ppm)

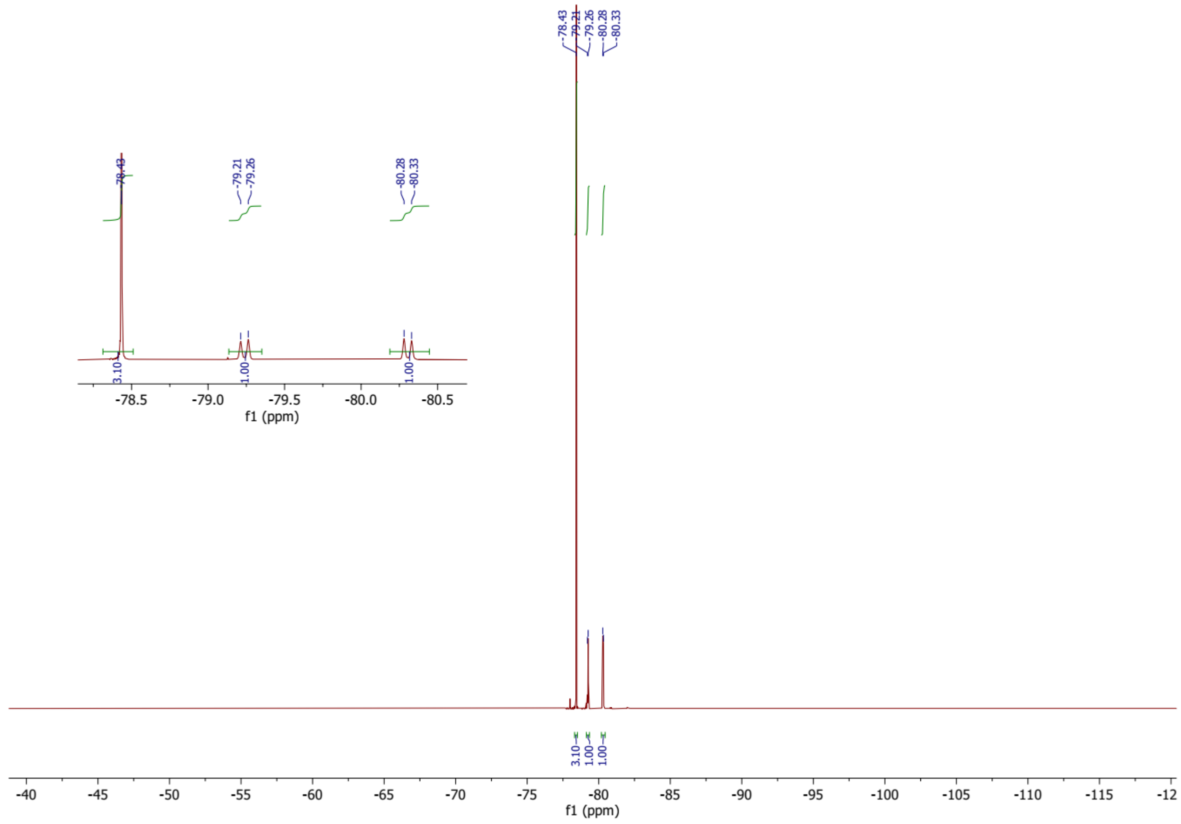
-78.43
-79.21
-79.26
-80.28
-80.33
3.10
1.00
1.00
-78.5
-79.0
-79.5
-80.0
-80.5
f1 (ppm)
-40
-45
-50
-55
-60
-65
-70
-75
-80
-85
-90
-95
-100
-105
-110
-115
-12
f1 (ppm)

2p

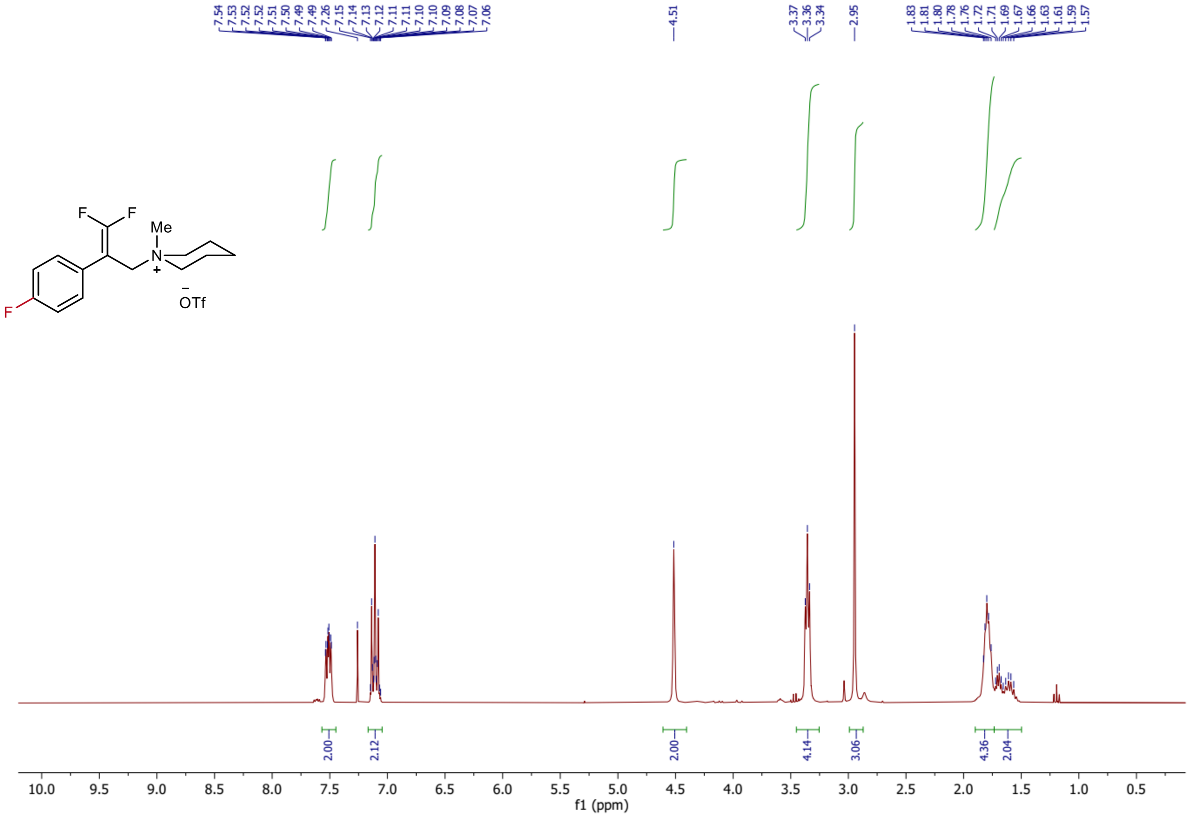

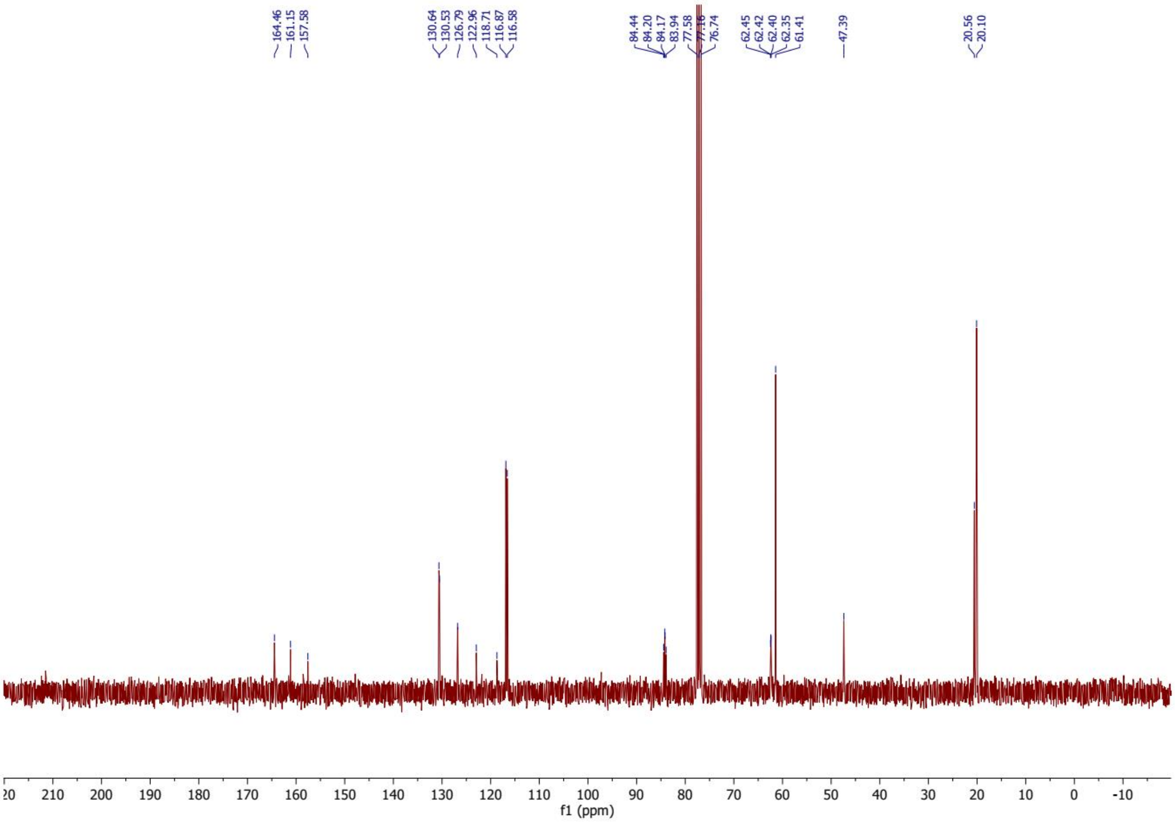

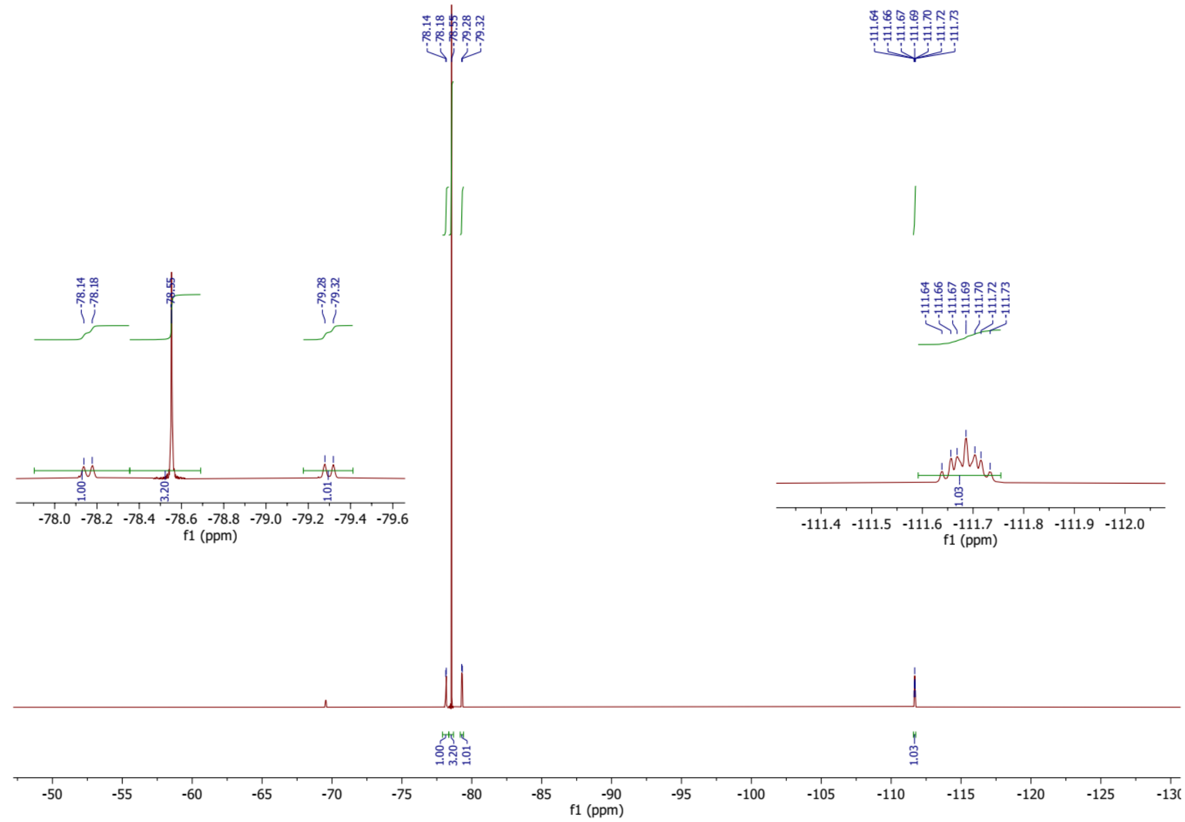

2s

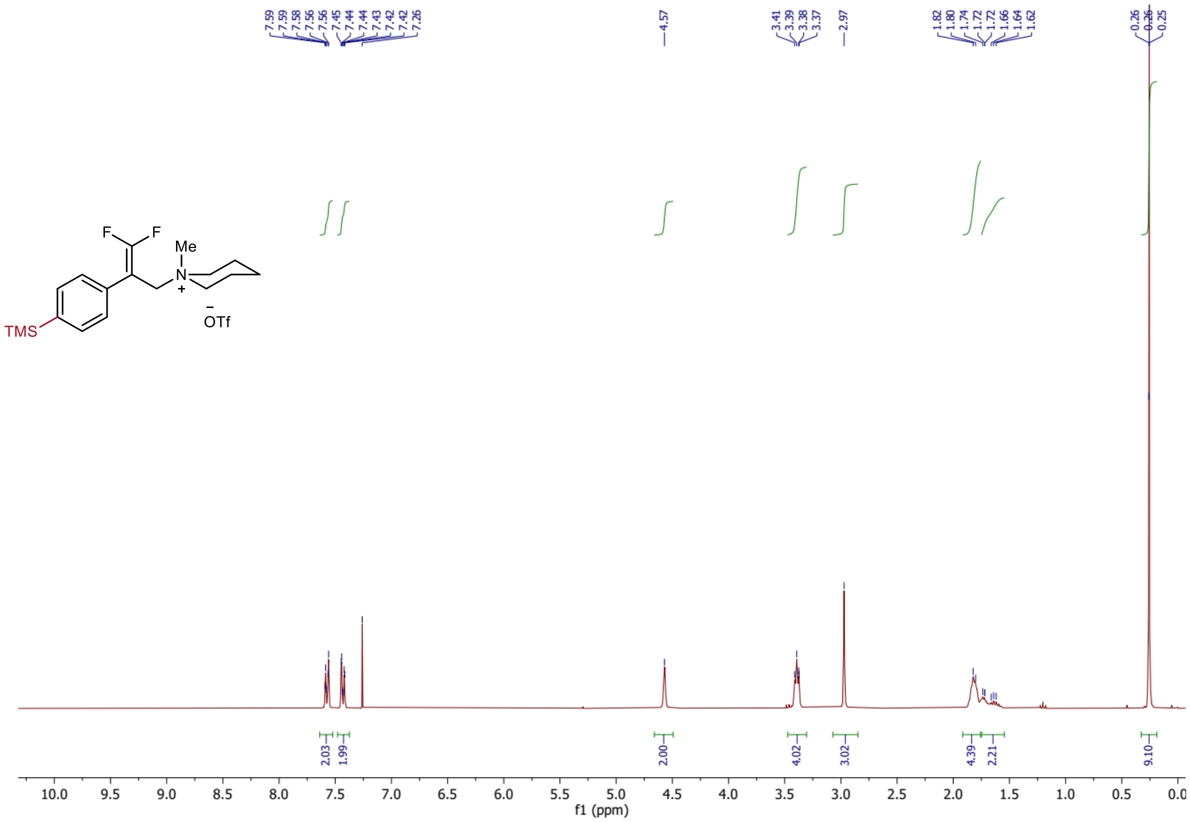

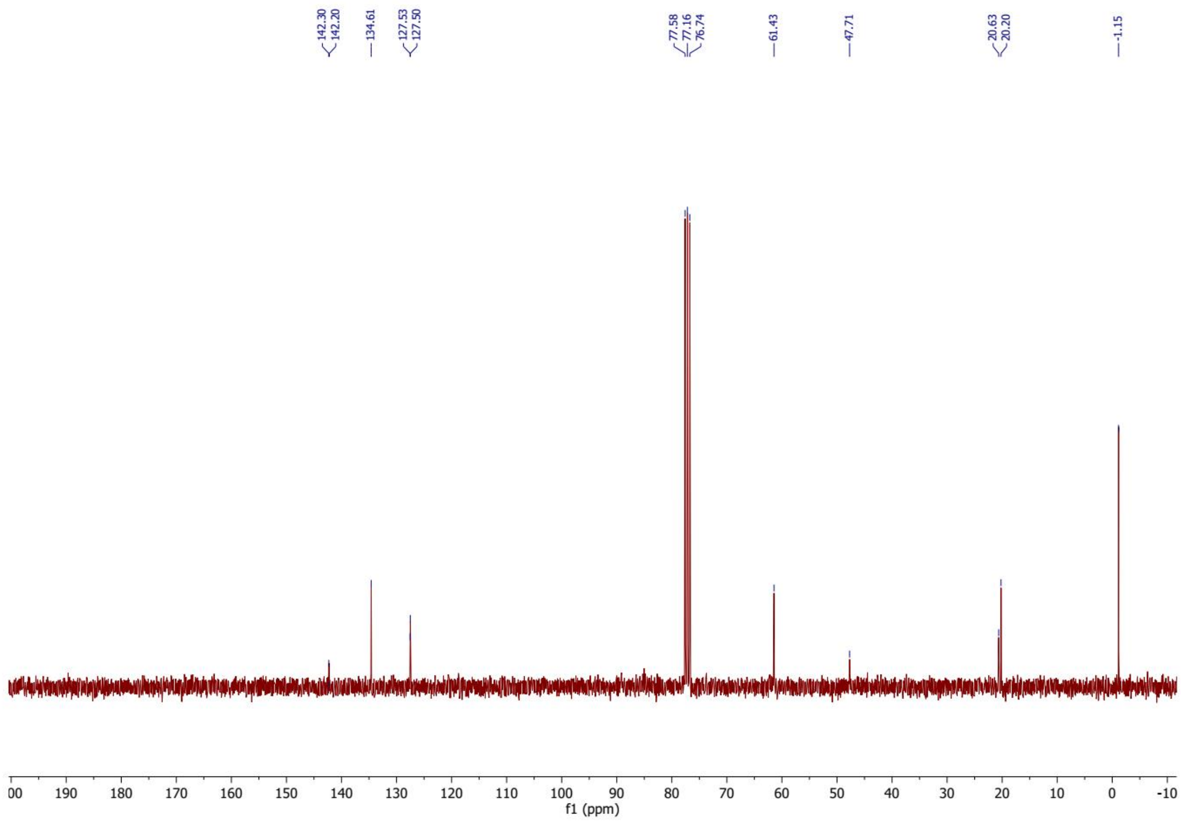
142.30
142.20
134.61
127.53
127.50
77.58
77.16
76.74
61.43
47.71
20.63
20.20
-1.15
f1 (ppm)

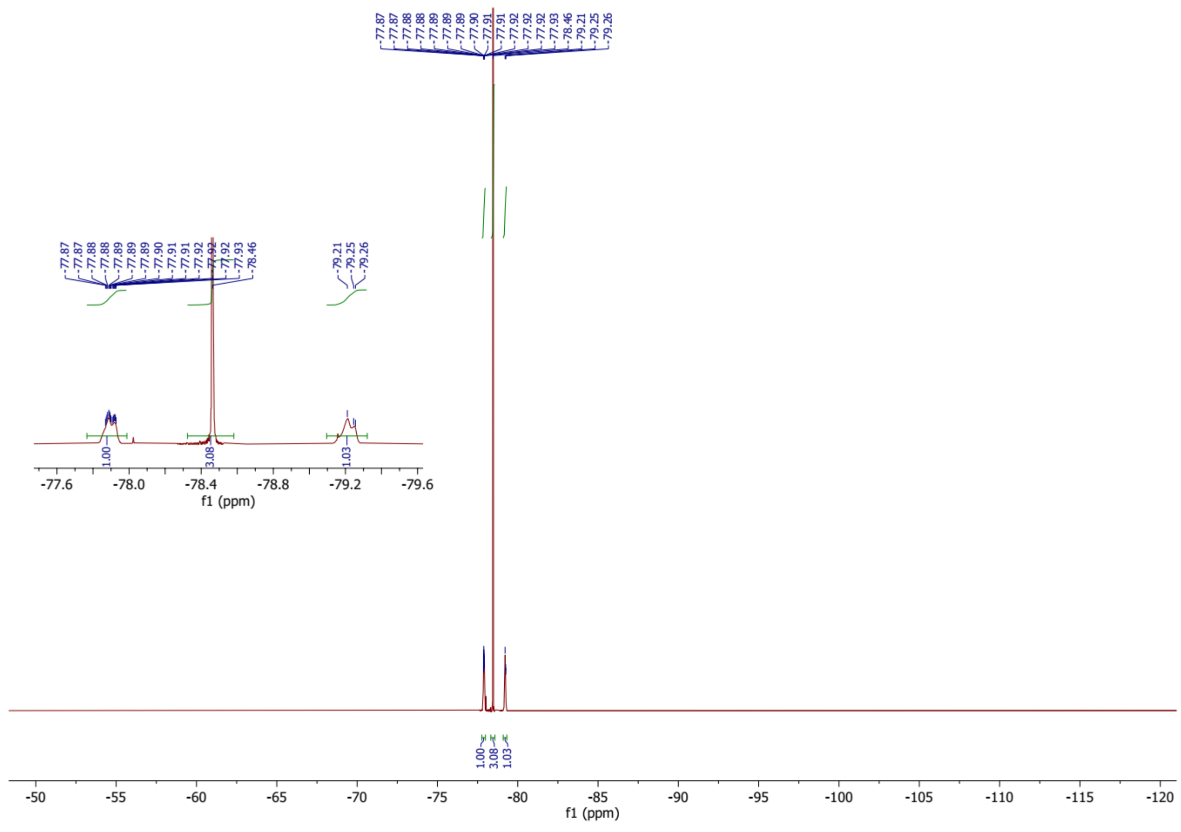
-77.87
-77.87
-77.88
-77.88
-77.89
-77.89
-77.89
-77.90
-77.91
-77.91
-77.92
-77.92
-77.92
-77.93
-78.46
-79.21
-79.25
-79.26
1.00
3.08
1.03
f1 (ppm)

2t

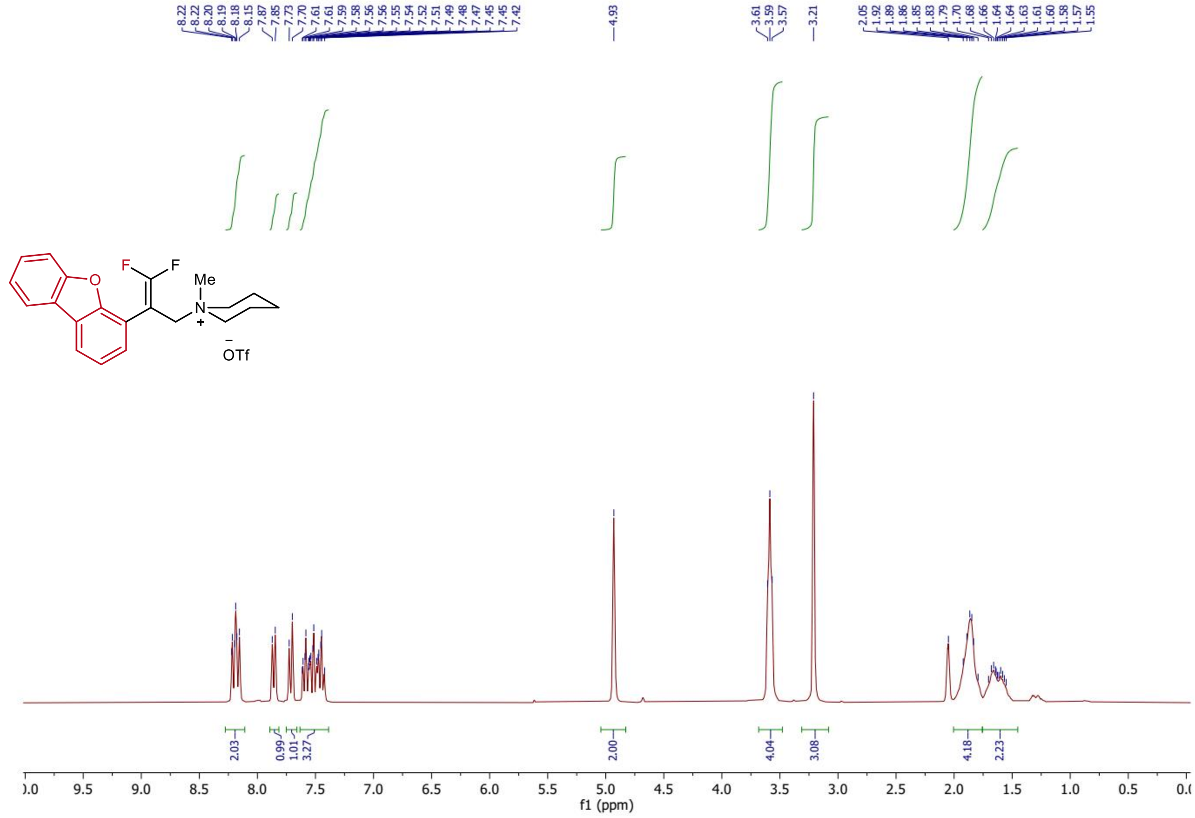
8.22 8.22 8.20 8.19 8.18 8.15 7.87 7.85 7.73 7.70 7.61 7.61 7.59 7.58 7.56 7.56 7.55 7.54 7.52 7.51 7.49 7.48 7.47 7.45 7.45 7.42
4.93
3.61 3.59 3.57
3.21
2.05 1.92 1.89 1.86 1.85 1.83 1.79 1.70 1.68 1.66 1.64 1.64 1.63 1.61 1.60 1.58 1.57 1.55
F
F
Me
N
+
O
OTf
2.03
0.99
1.01
3.27
2.00
4.04
3.08
4.18
2.23
f1 (ppm)

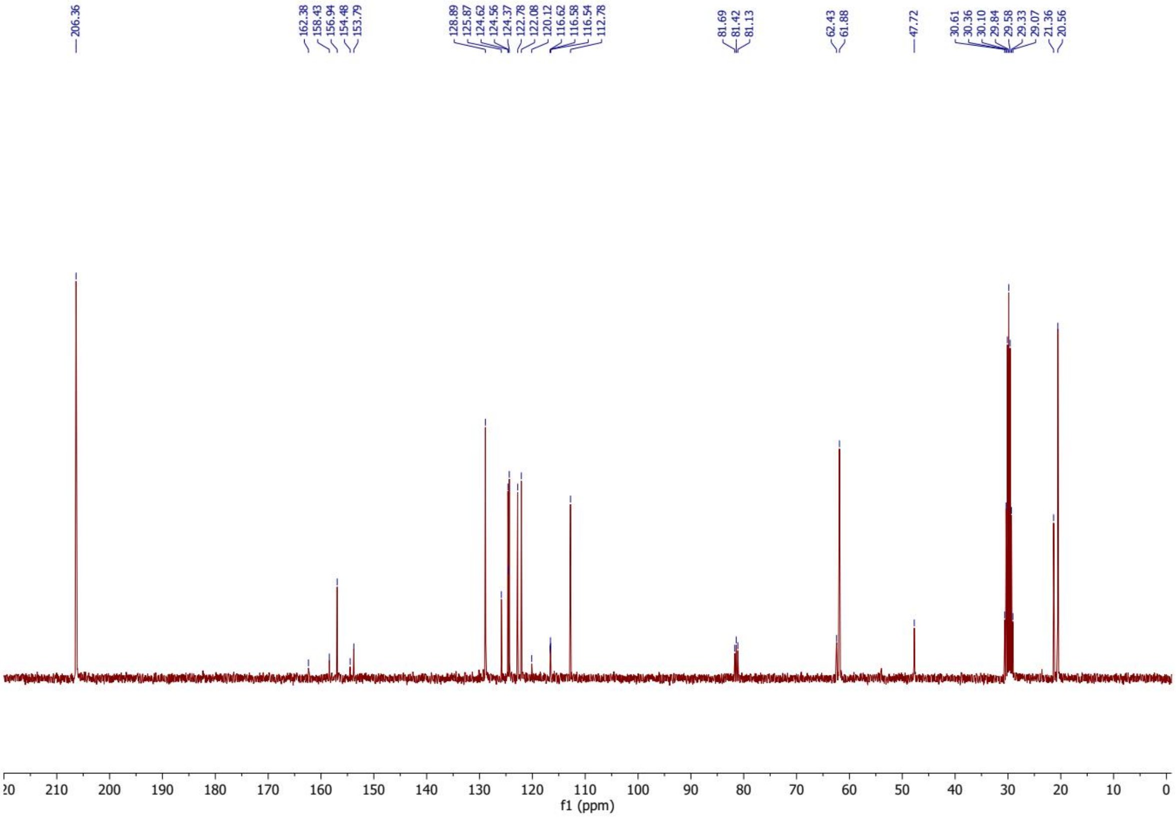
206.36
162.38 158.43 156.94 154.48 153.79
128.89 125.87 124.62 124.56 124.37 122.78 122.08 120.12 116.62 116.58 116.54 112.78
81.69 81.42 81.13
62.43 61.88
47.72
30.61 30.36 30.10 29.84 29.58 29.33 29.07 21.36 20.56
f1 (ppm)

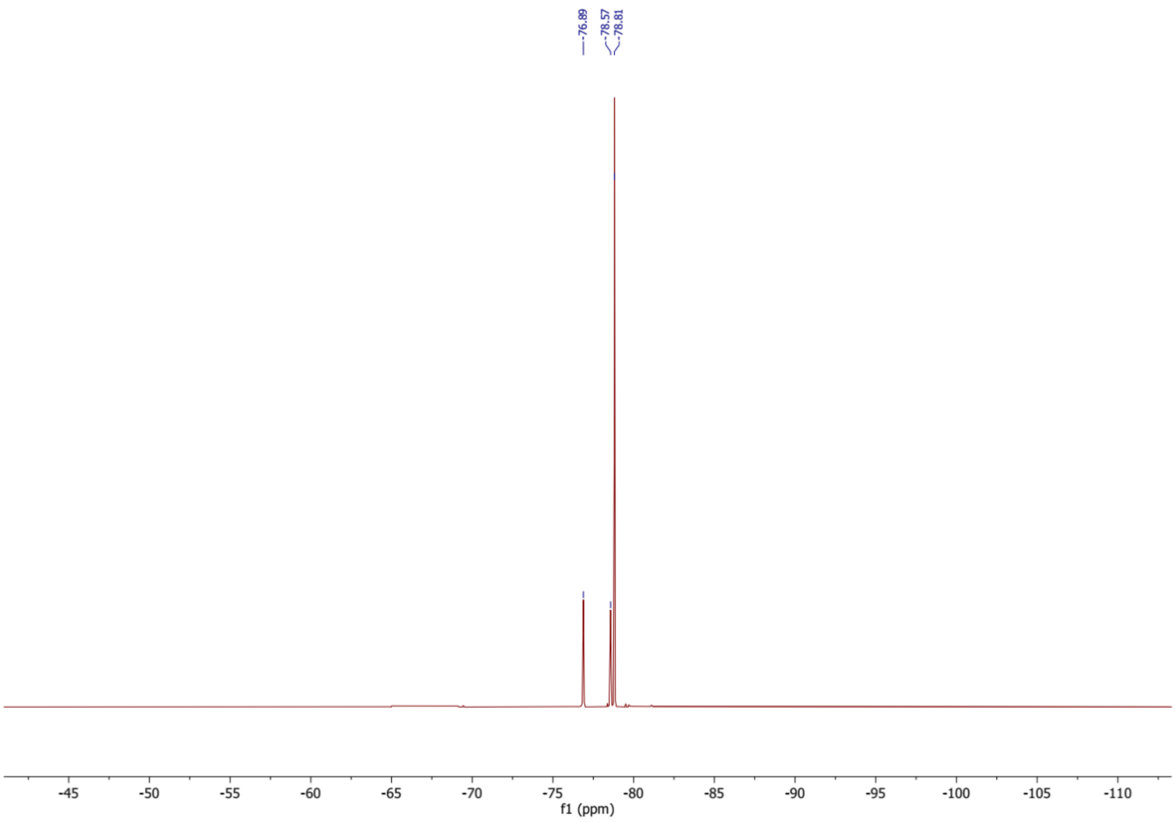

2u

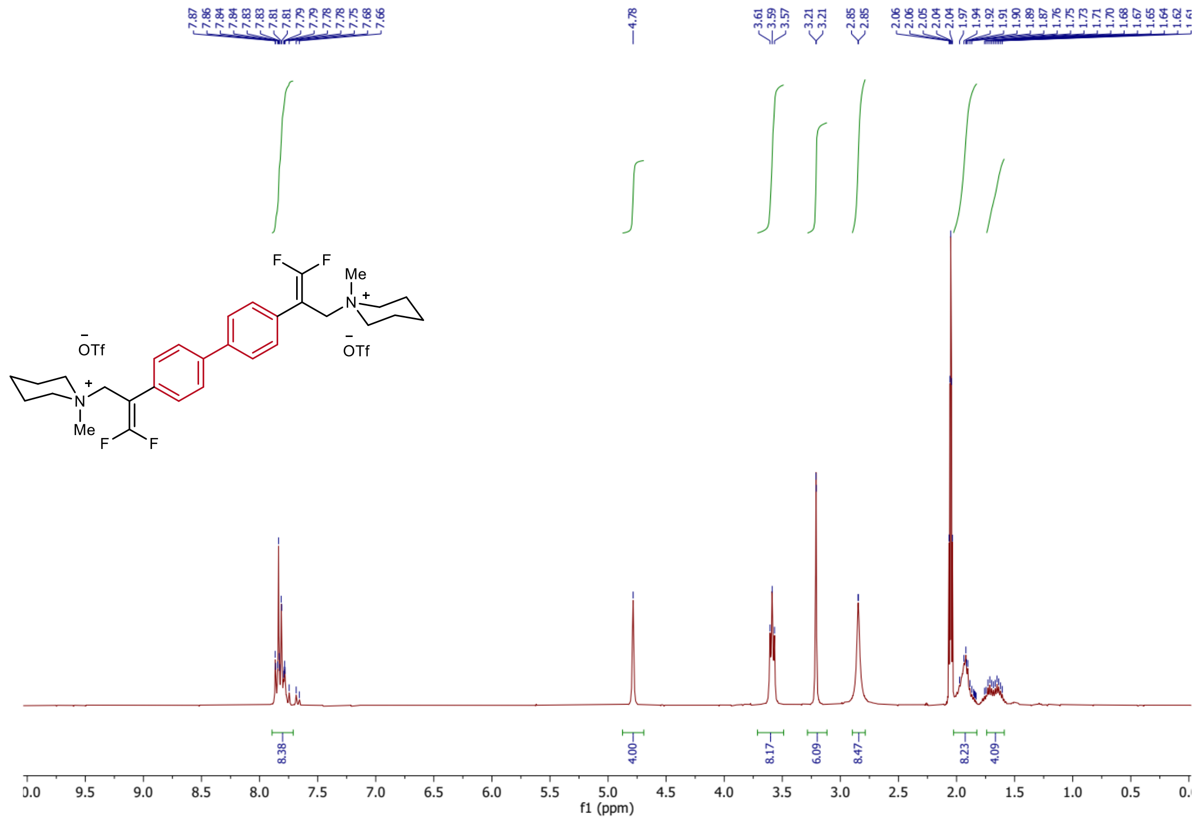

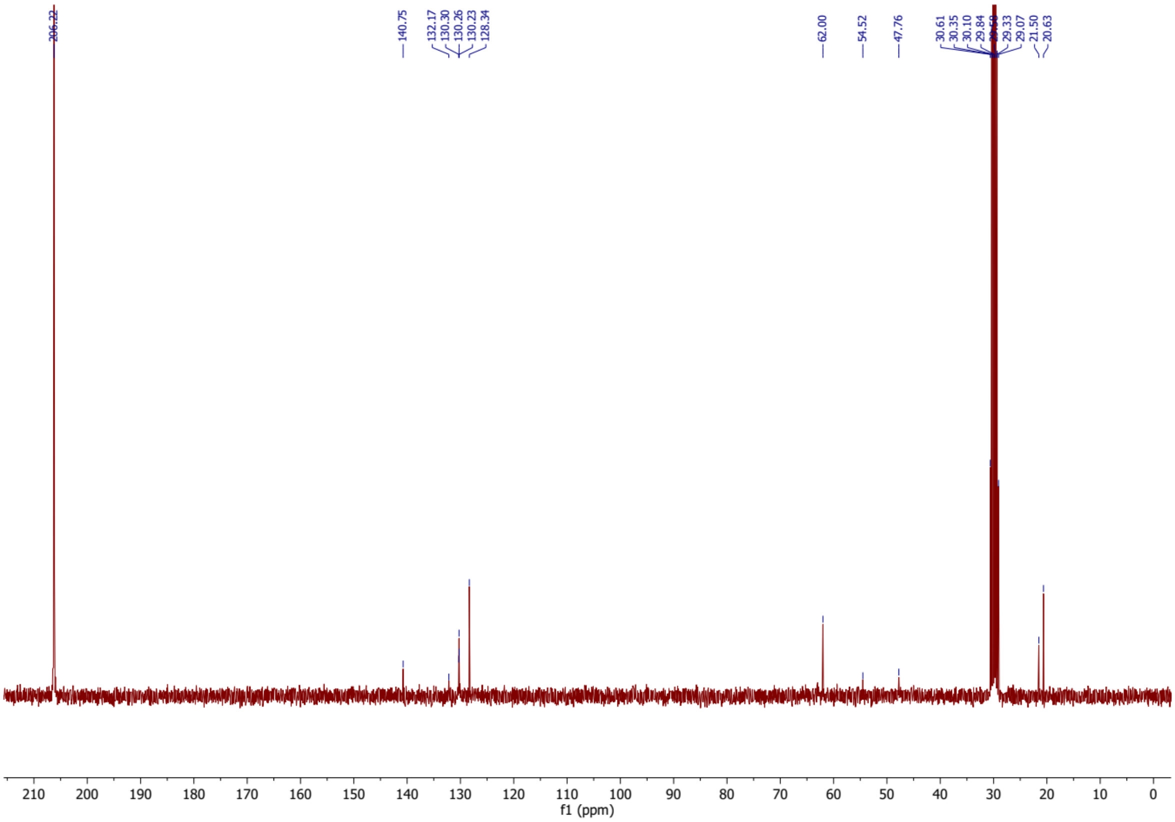
206.22
140.75
132.17
130.30
130.26
130.23
128.34
62.00
54.52
47.76
30.61
30.35
30.10
29.84
29.59
29.33
29.07
21.50
20.63
210 200 190 180 170 160 150 140 130 120 110 100 90 80 70 60 50 40 30 20 10 0
f1 (ppm)

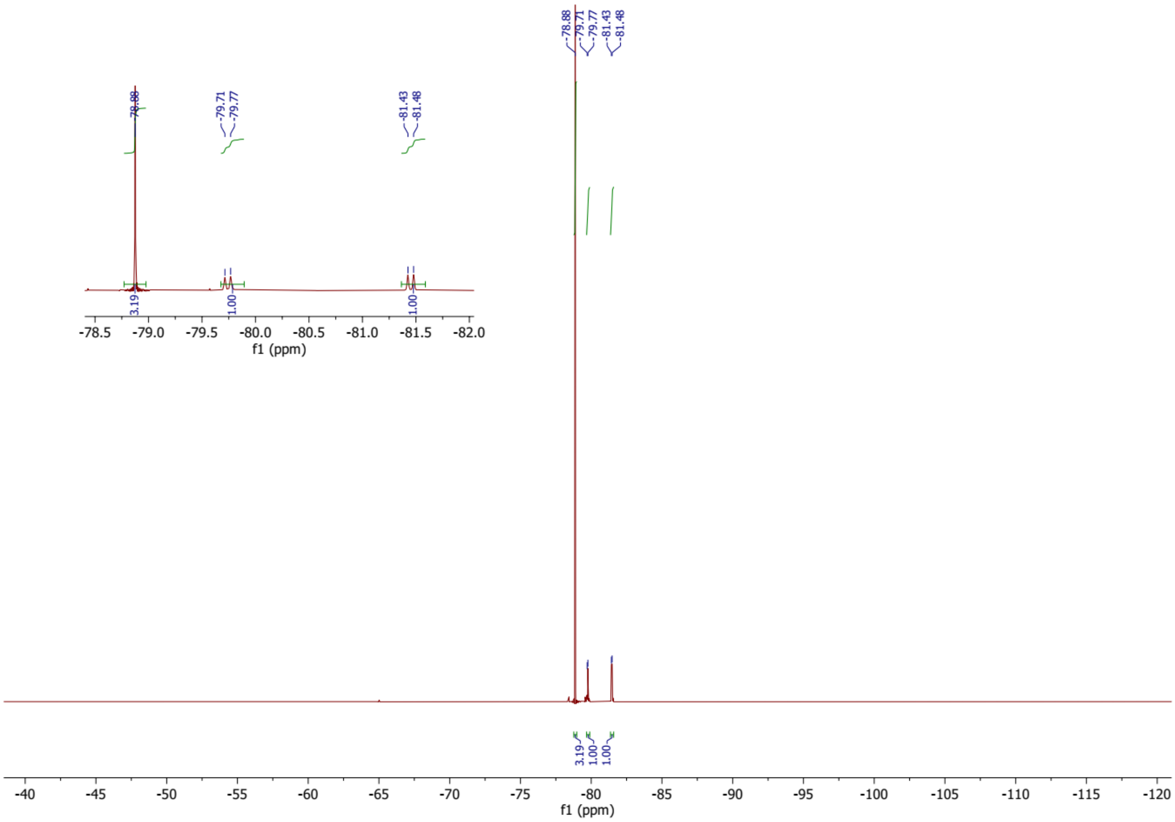
-78.88
-79.71
-79.77
-81.43
-81.48
3.19
1.00
1.00
-78.5 -79.0 -79.5 -80.0 -80.5 -81.0 -81.5 -82.0
f1 (ppm)
-40 -45 -50 -55 -60 -65 -70 -75 -80 -85 -90 -95 -100 -105 -110 -115 -120
f1 (ppm)

3a

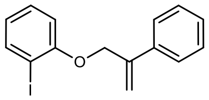
O
I

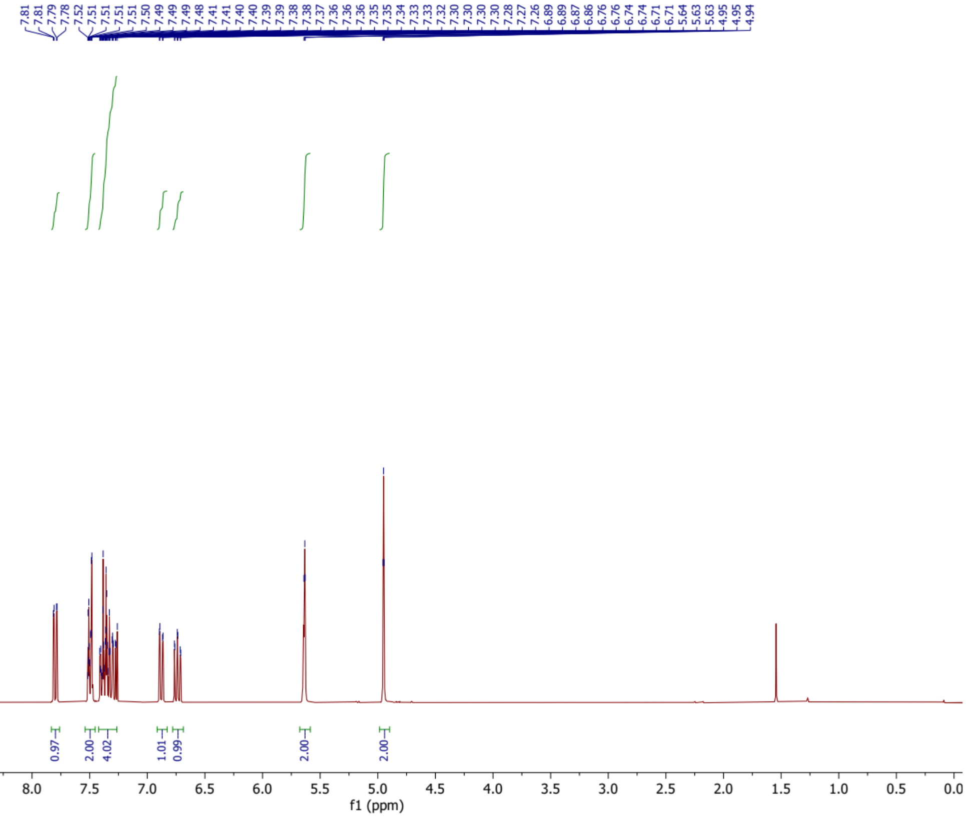
7.81 7.81 7.79 7.78 7.52 7.51 7.51 7.51 7.51 7.50 7.49 7.49 7.49 7.48 7.41 7.41 7.40 7.40 7.39 7.39 7.38 7.38 7.37 7.36 7.36 7.36 7.35 7.35 7.34 7.33 7.33 7.32 7.30 7.30 7.30 7.30 7.28 7.27 7.26 6.89 6.89 6.87 6.86 6.76 6.76 6.74 6.74 6.71 5.64 5.63 5.63 4.95 4.95 4.94
0.97 2.00 4.02 1.01 0.99 2.00 2.00
f1 (ppm)

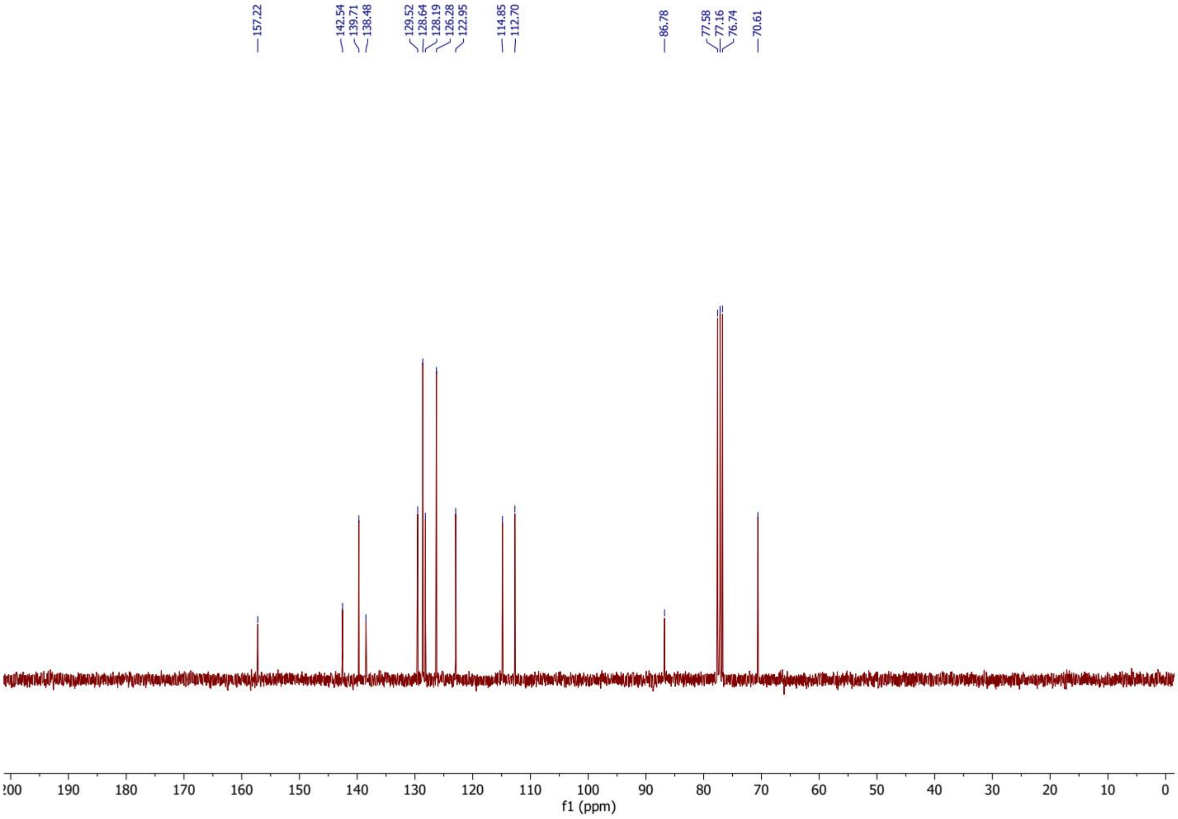
157.22 142.54 139.71 138.48 129.52 128.64 128.19 126.28 122.95 114.85 112.70 86.78 77.58 77.16 76.74 70.61
f1 (ppm)

3b

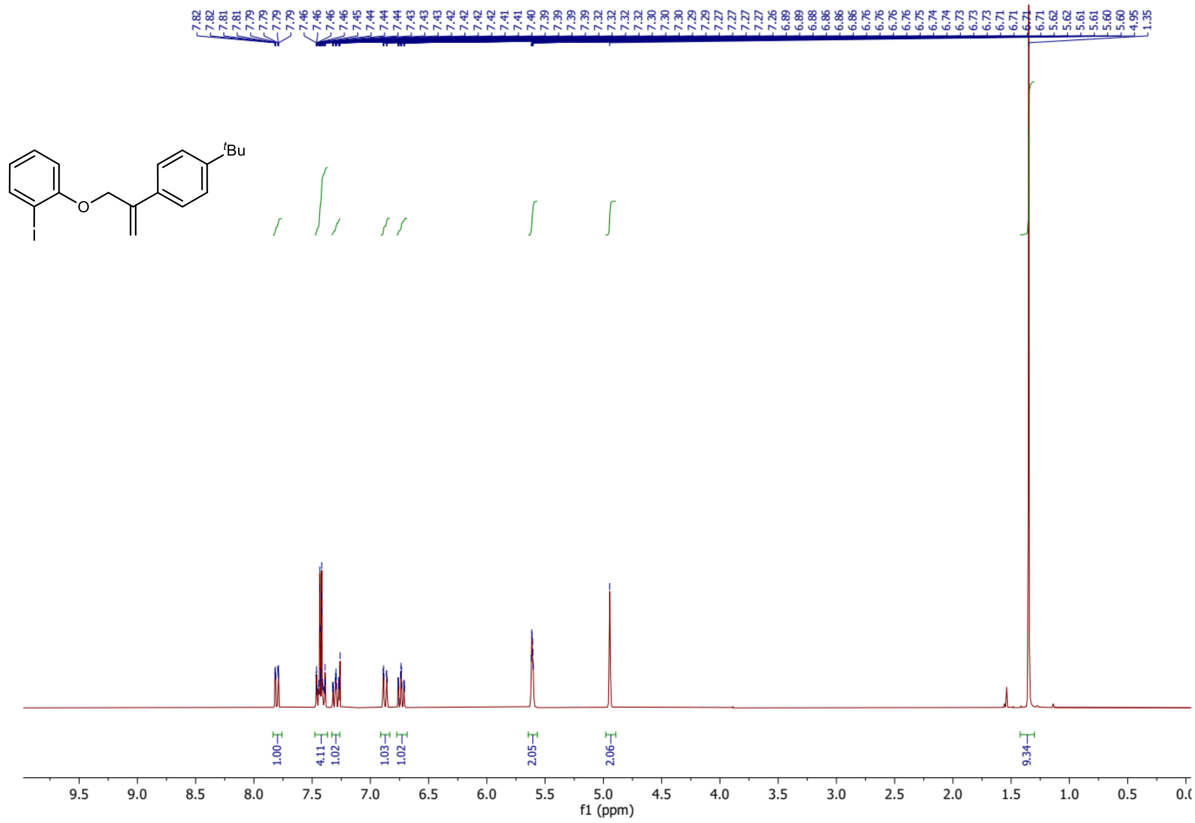

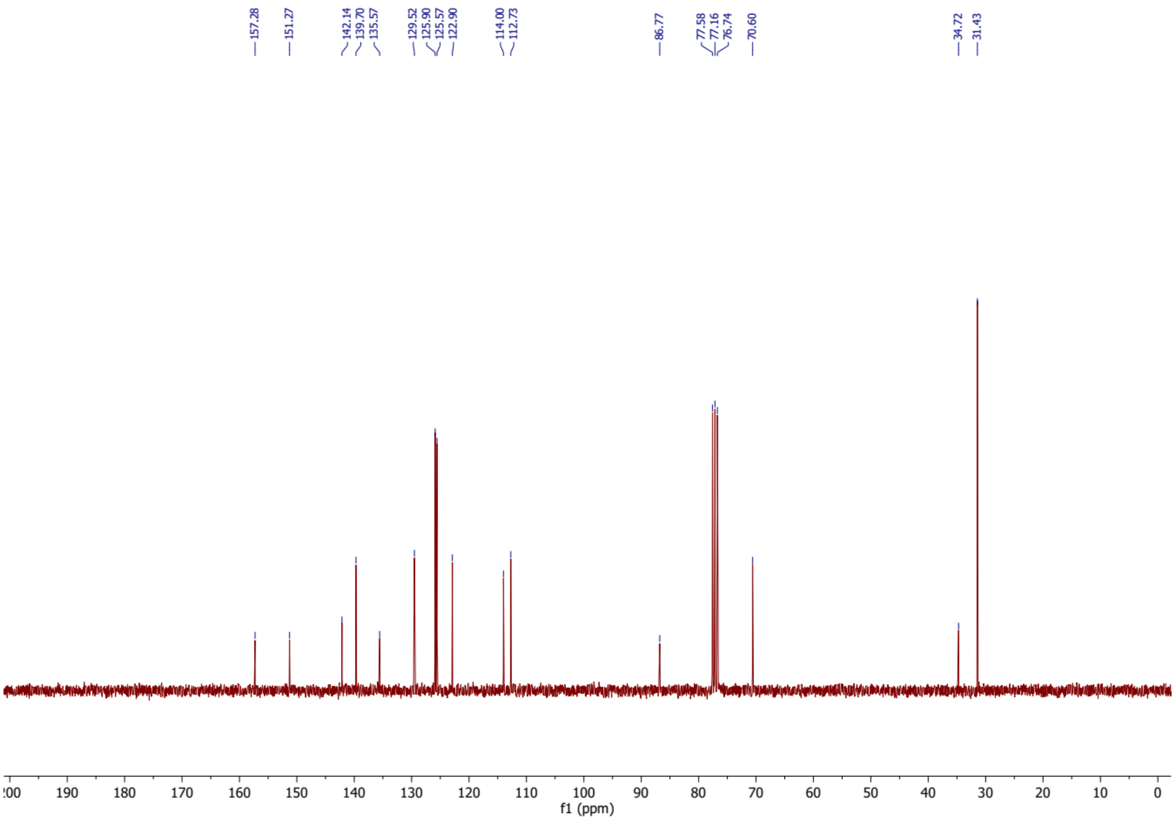

3c

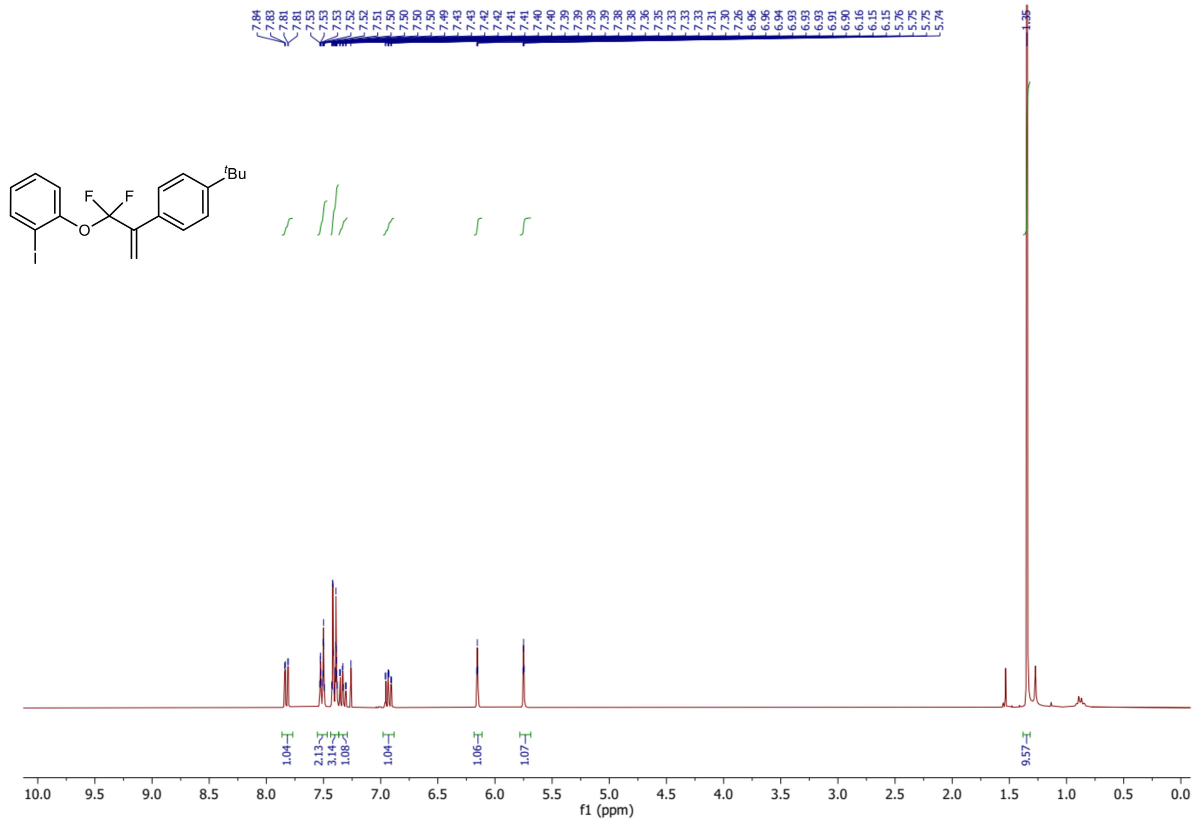

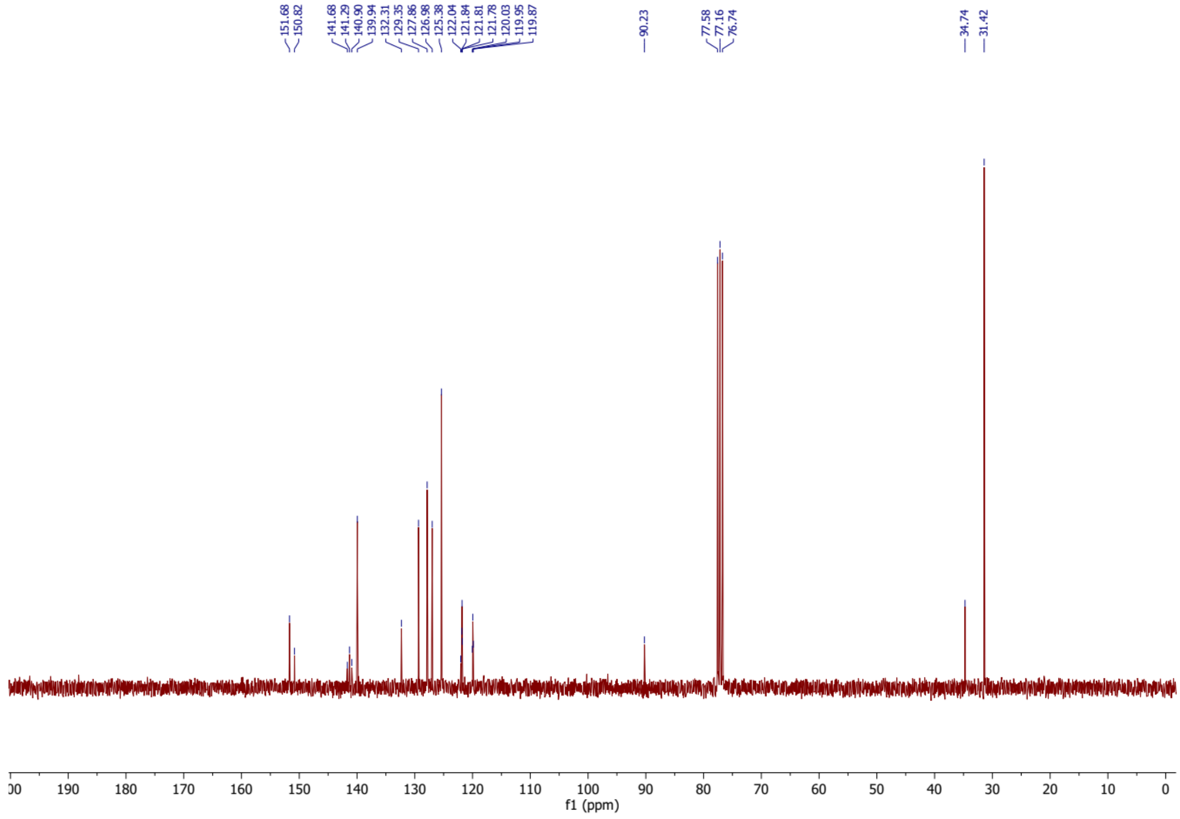

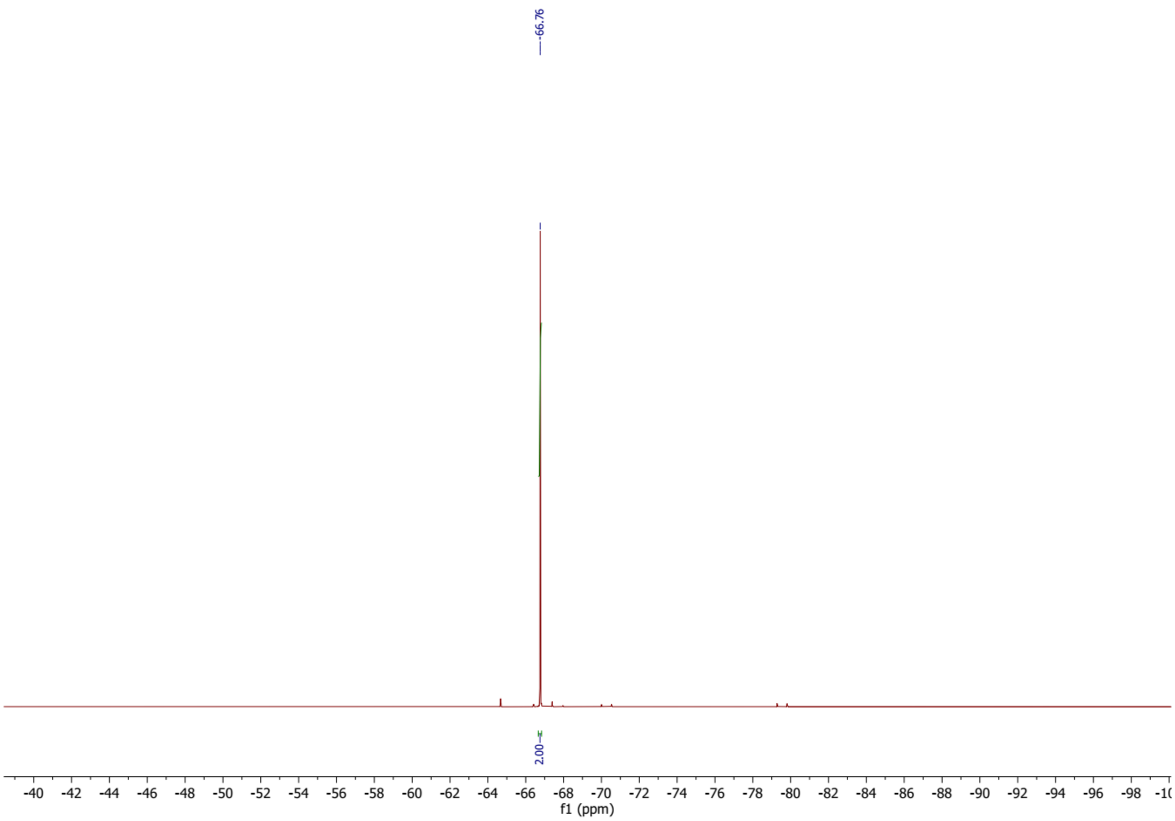

4b

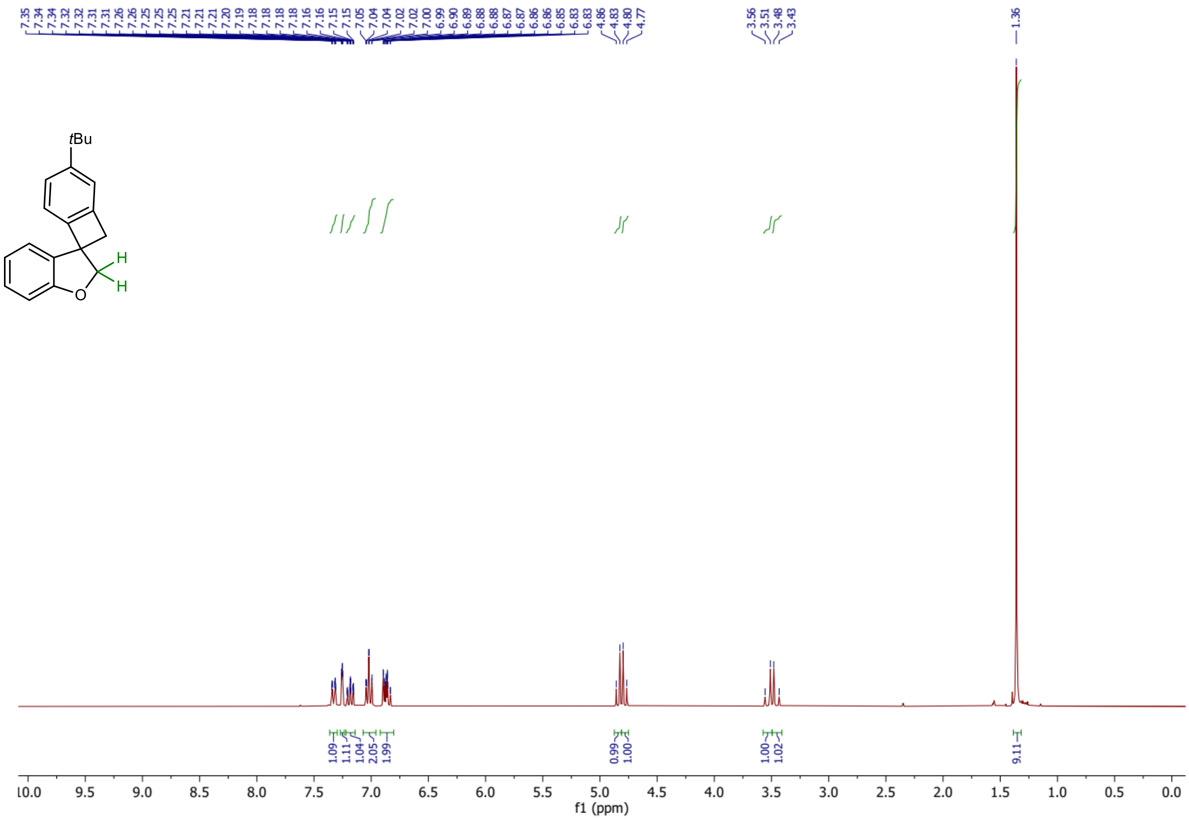

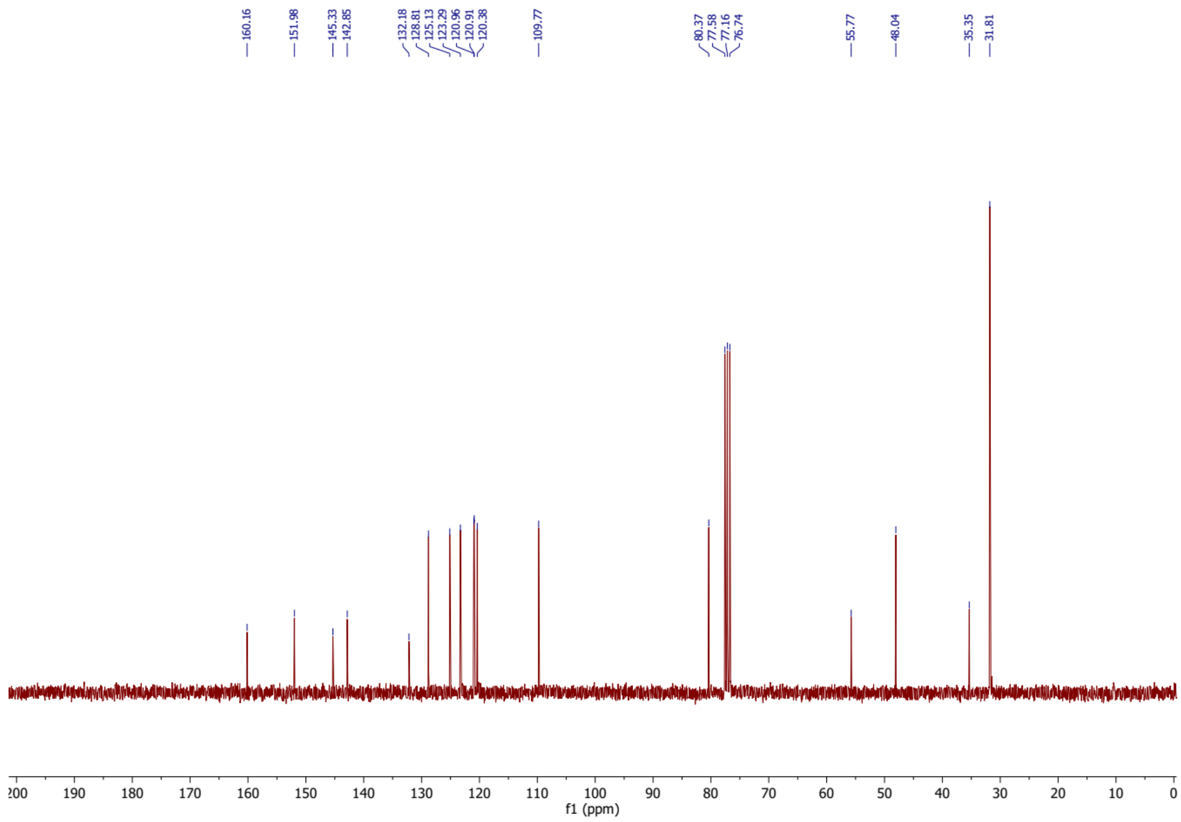
160.16
151.98
145.33
142.85
132.18
128.81
125.13
123.29
120.96
120.91
120.38
109.77
80.37
77.58
77.16
76.74
55.77
48.04
35.35
31.81
f1 (ppm)

4c

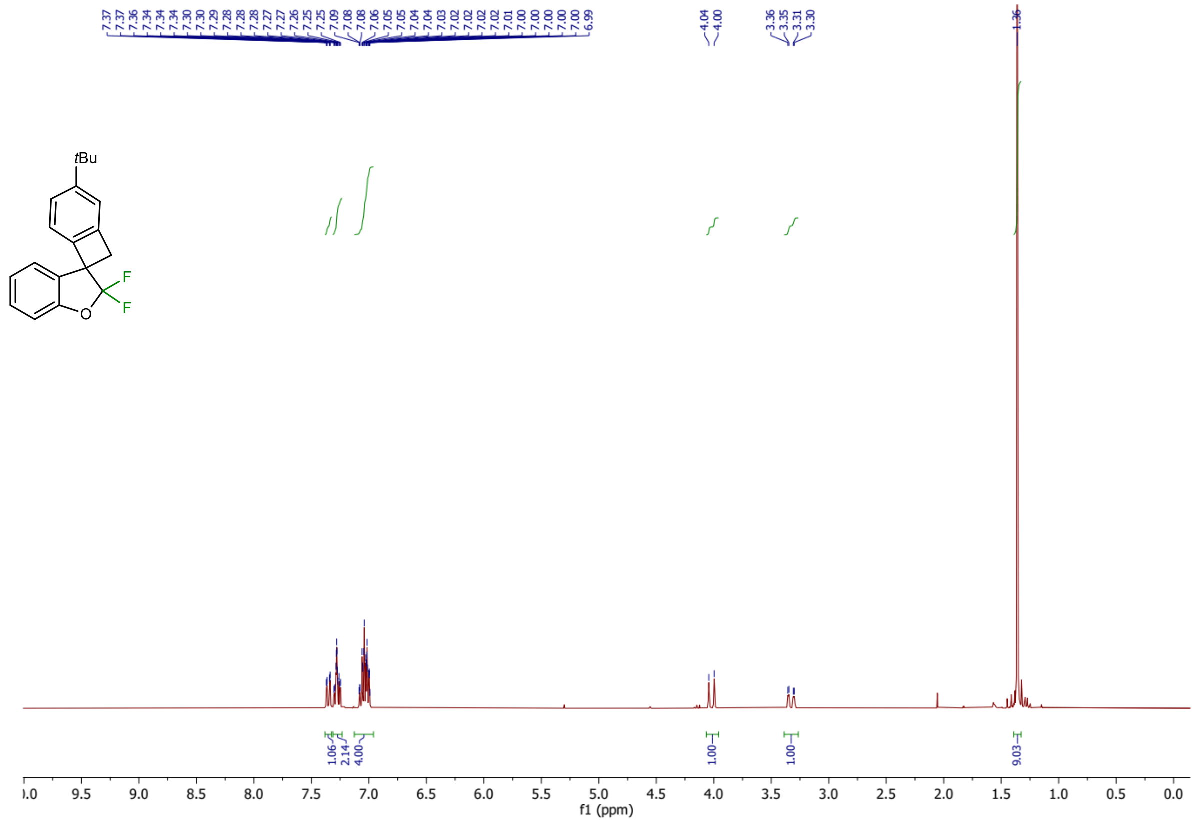
7.37
7.37
7.36
7.34
7.34
7.34
7.30
7.30
7.29
7.28
7.28
7.28
7.27
7.26
7.25
7.25
7.09
7.08
7.06
7.05
7.05
7.04
7.04
7.03
7.02
7.02
7.02
7.02
7.01
7.00
7.00
7.00
7.00
6.99
4.04
4.00
3.36
3.35
3.31
3.30
1.36
tBu
F
F
O
1.06
2.14
4.00
1.00
1.00
9.03
f1 (ppm)

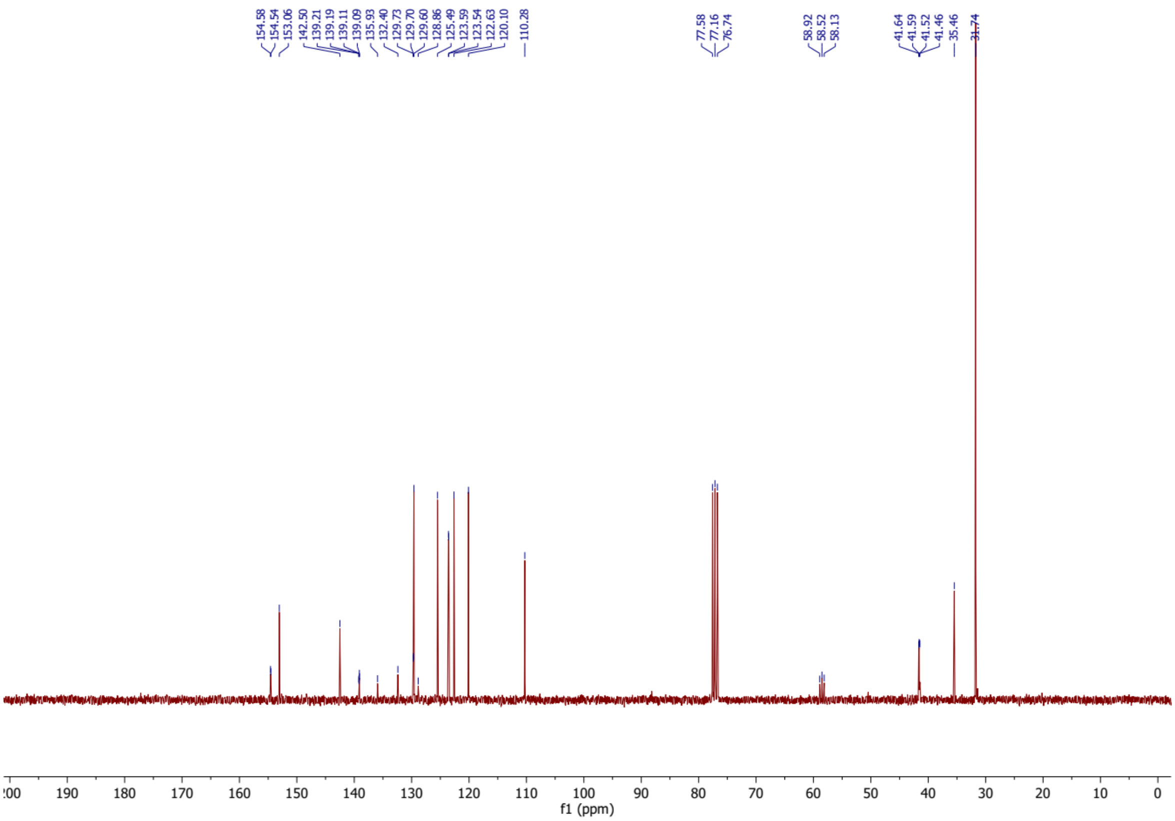
154.58
154.54
153.06
142.50
139.21
139.19
139.11
139.09
135.93
132.40
129.73
129.70
129.60
128.86
125.49
123.59
123.54
122.63
120.10
110.28
77.58
77.16
76.74
58.92
58.52
58.13
41.64
41.59
41.52
41.46
35.46
31.74
f1 (ppm)

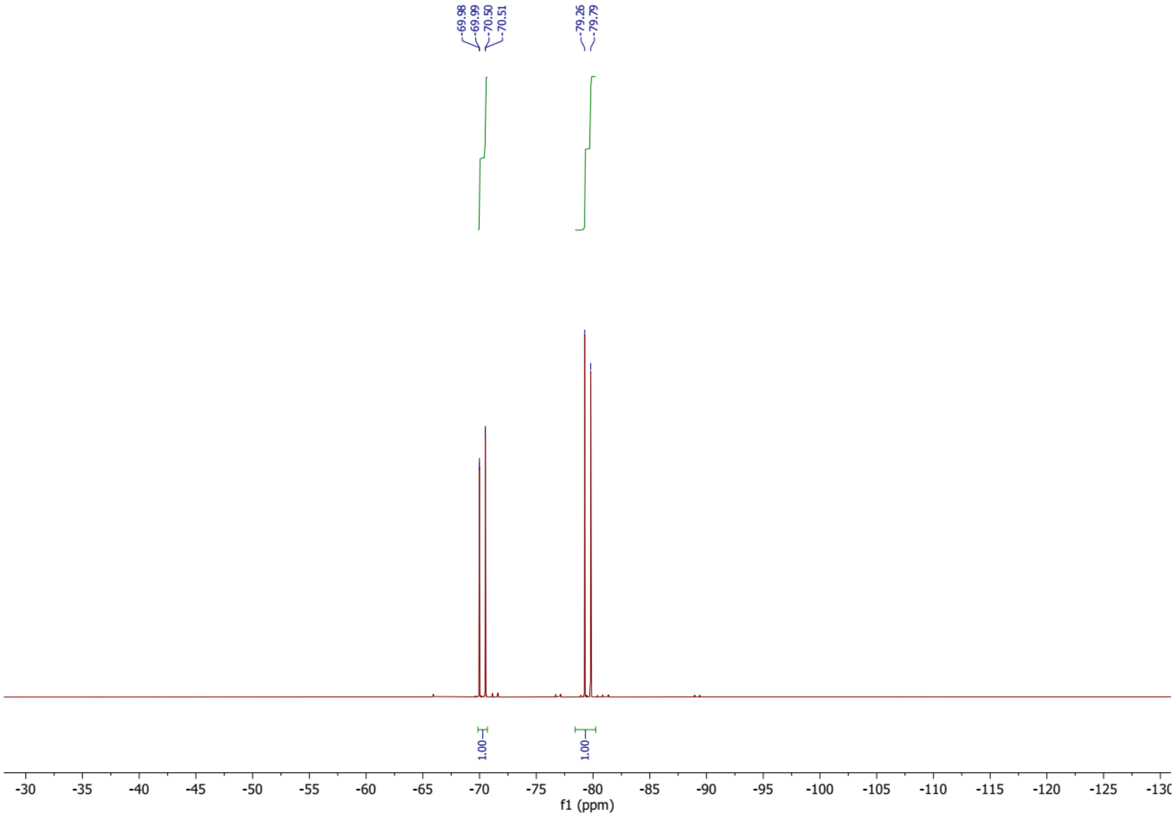
-69.98
-69.99
-70.50
-70.51
-79.26
-79.79
1.00
1.00
f1 (ppm)

4d

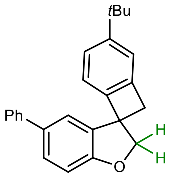

tBu
Ph
H
H
O

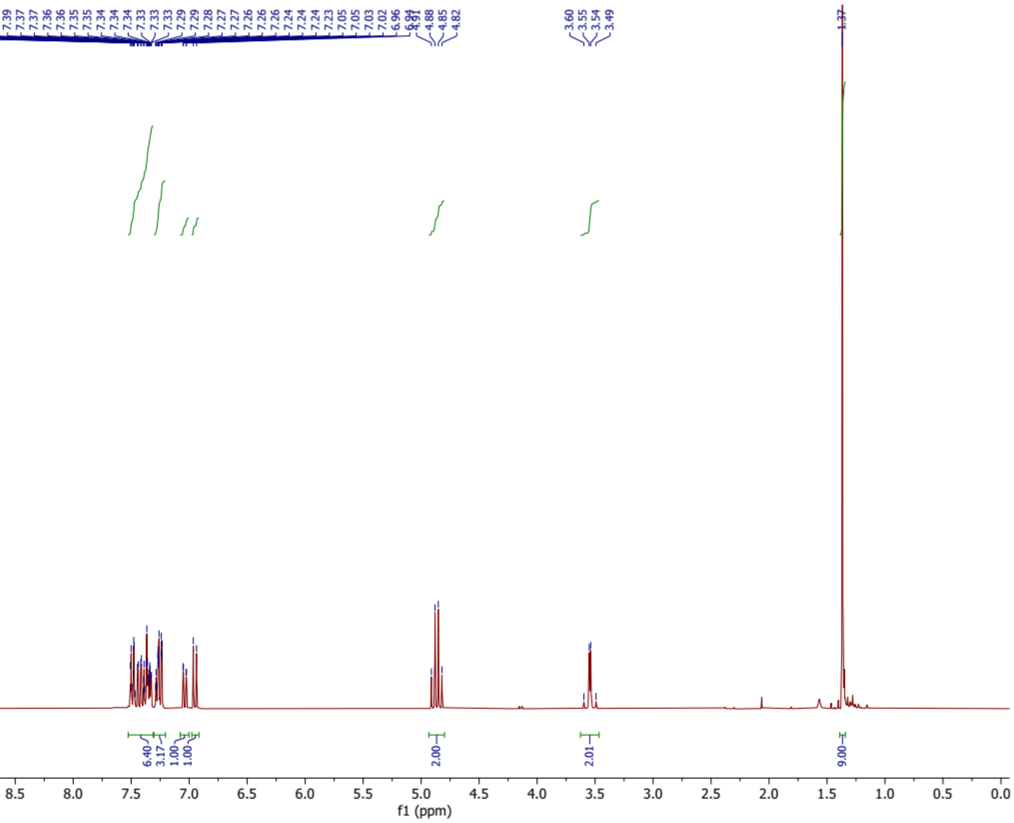

7.51 7.50 7.49 7.48 7.48 7.47 7.45 7.44 7.42 7.41 7.39 7.39 7.37 7.37 7.36 7.36 7.35 7.35 7.34 7.34 7.33 7.33 7.33 7.33 7.29 7.29 7.28 7.27 7.27 7.26 7.26 7.26 7.24 7.24 7.24 7.23 7.05 7.05 7.03 7.02 6.96 6.94 4.91 4.88 4.85 4.82
3.60 3.55 3.54 3.49
1.37
6.40 3.17 1.00 1.00
2.00
2.01
9.00
f1 (ppm)

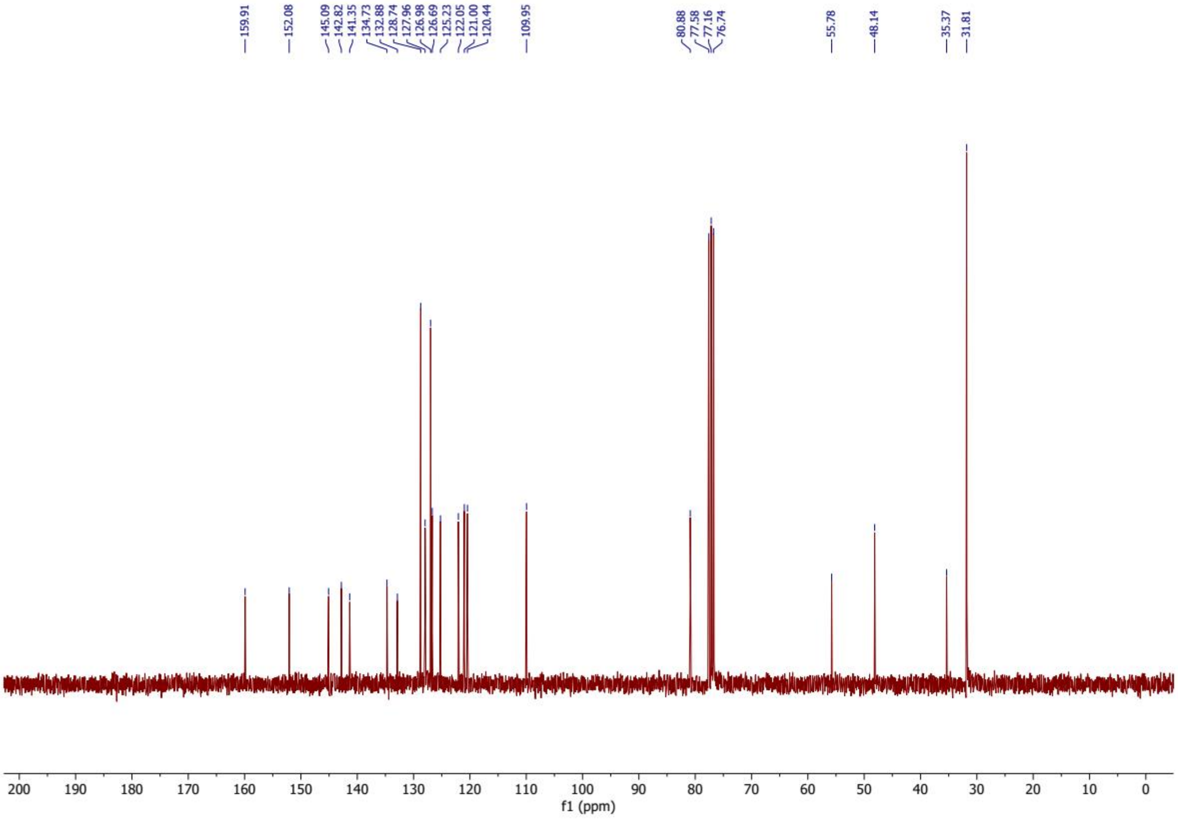

159.91
152.08
145.09 142.82 141.35 134.73 132.88 128.74 127.96 126.98 126.69 125.23 122.05 121.00 120.44
109.95
80.88 77.58 77.16 76.74
55.78
48.14
35.37
31.81
f1 (ppm)

4e

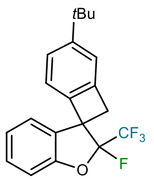

tBu
CF_3
F
O

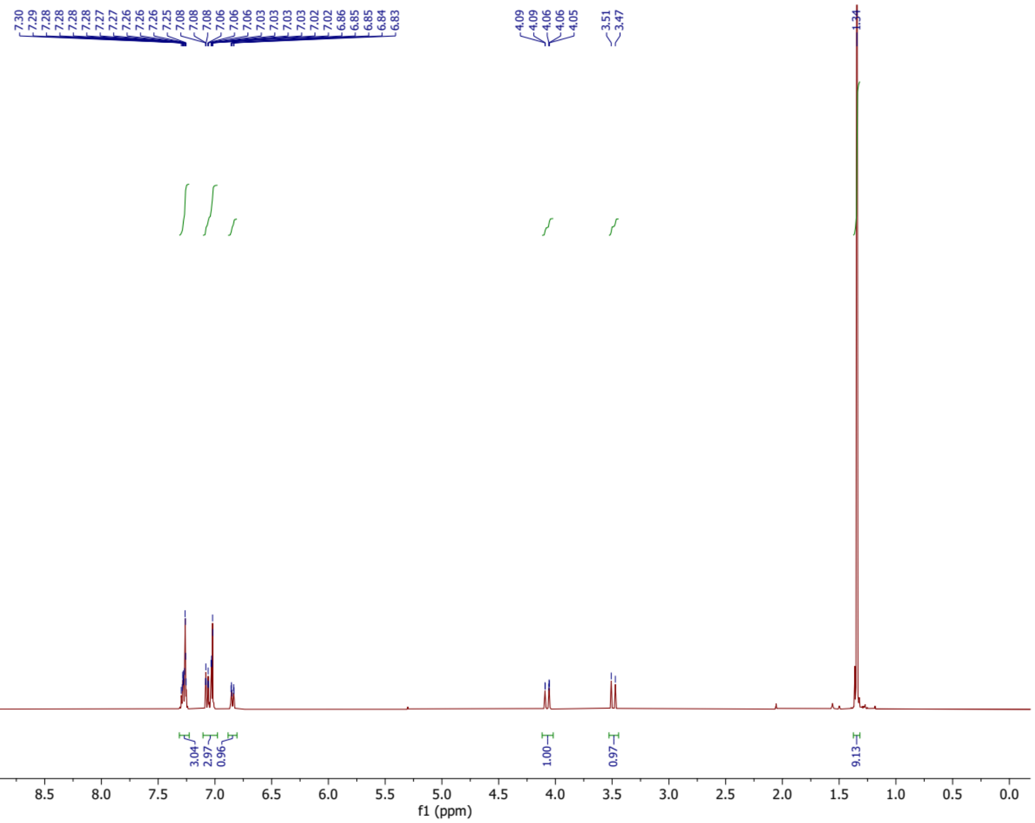

7.30 7.29 7.28 7.28 7.28 7.27 7.27 7.26 7.26 7.26 7.25 7.08 7.08 7.08 7.06 7.06 7.06 7.03 7.03 7.03 7.03 7.02 6.86 6.85 6.85 6.84 6.83
4.09 4.09 4.06 4.06 4.05
3.51 3.47
1.34
3.04 2.97 0.96
1.00
0.97
9.13
10.0 9.5 9.0 8.5 8.0 7.5 7.0 6.5 6.0 5.5 5.0 4.5 4.0 3.5 3.0 2.5 2.0 1.5 1.0 0.5 0.0
f1 (ppm)

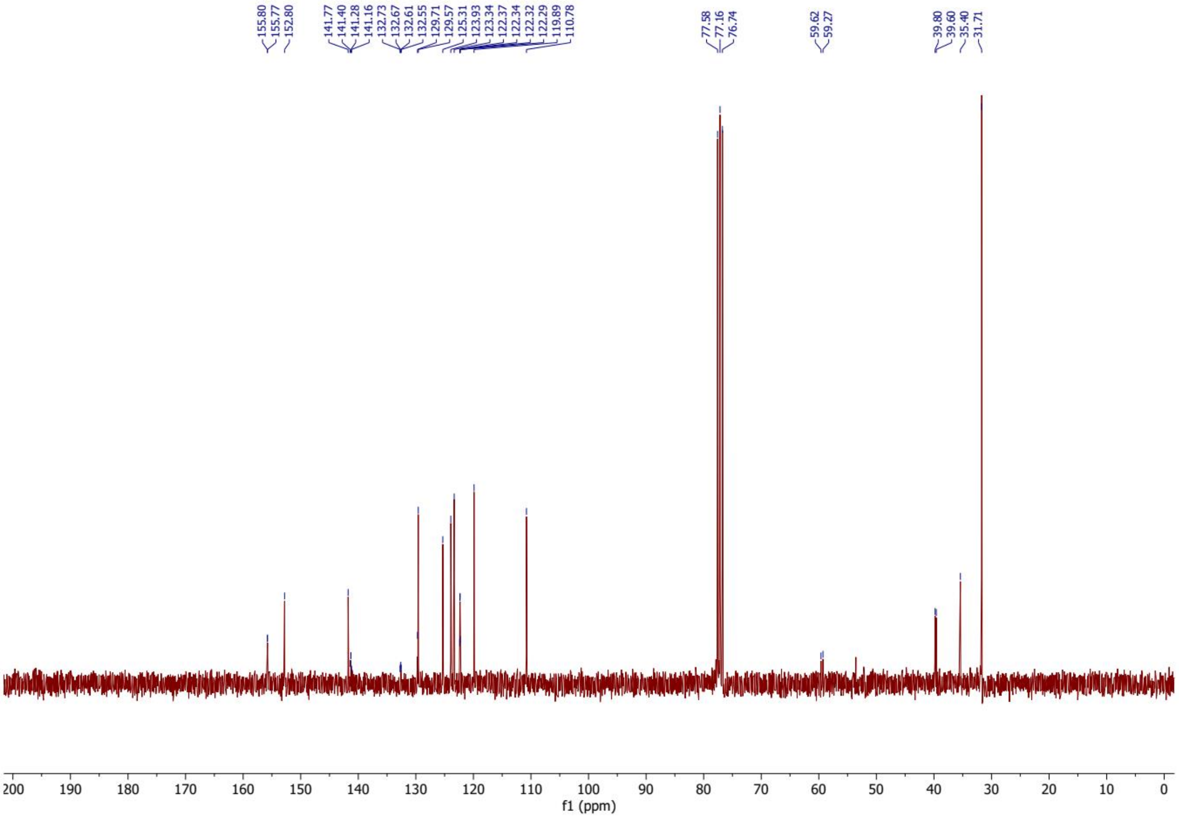

155.80 155.77 152.80
141.77 141.40 141.28 141.16 132.73 132.67 132.61 132.55 129.71 129.57 125.31 123.93 123.34 122.37 122.34 122.32 122.29 119.89 110.78
77.58 77.16 76.74
59.62 59.27
39.80 39.60 35.40 31.71
200 190 180 170 160 150 140 130 120 110 100 90 80 70 60 50 40 30 20 10 0
f1 (ppm)

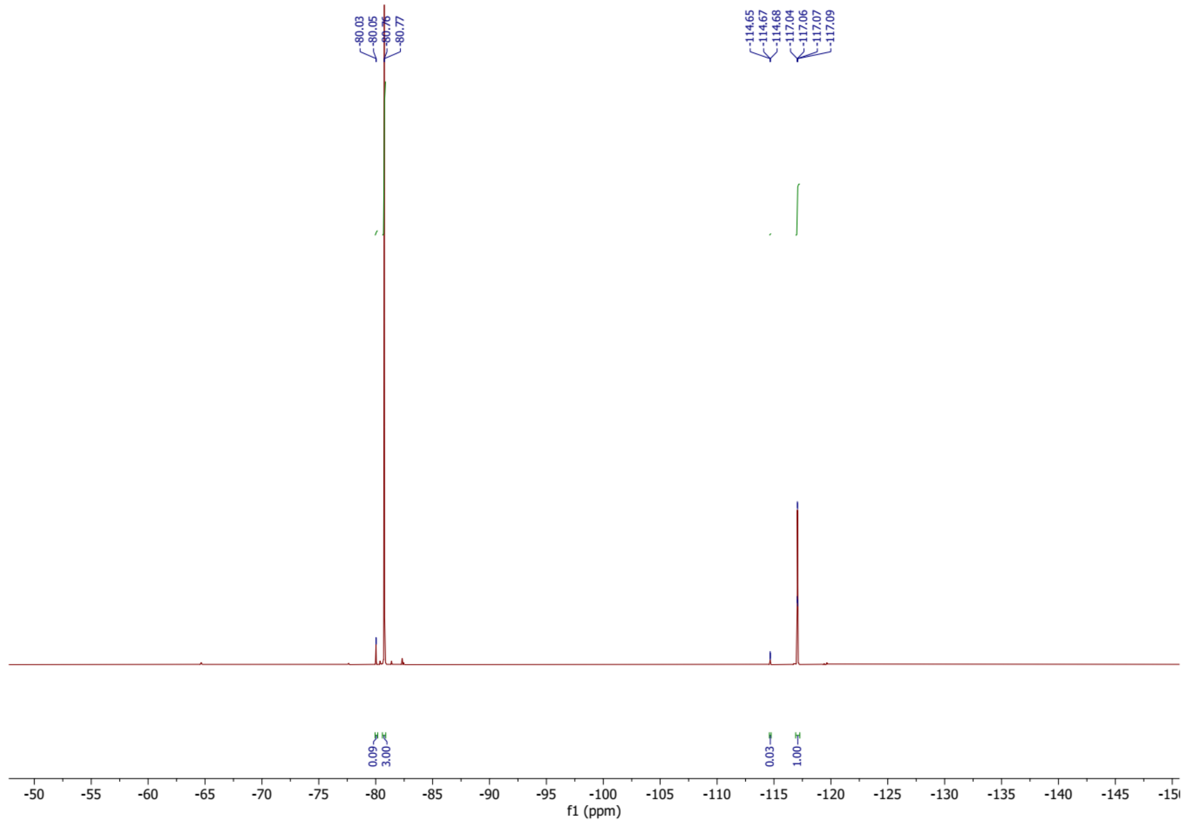
-80.03
-80.05
-80.76
-80.77
-114.65
-114.67
-114.68
-117.04
-117.06
-117.07
-117.09
0.09
3.00
0.03
1.00
f1 (ppm)

4f

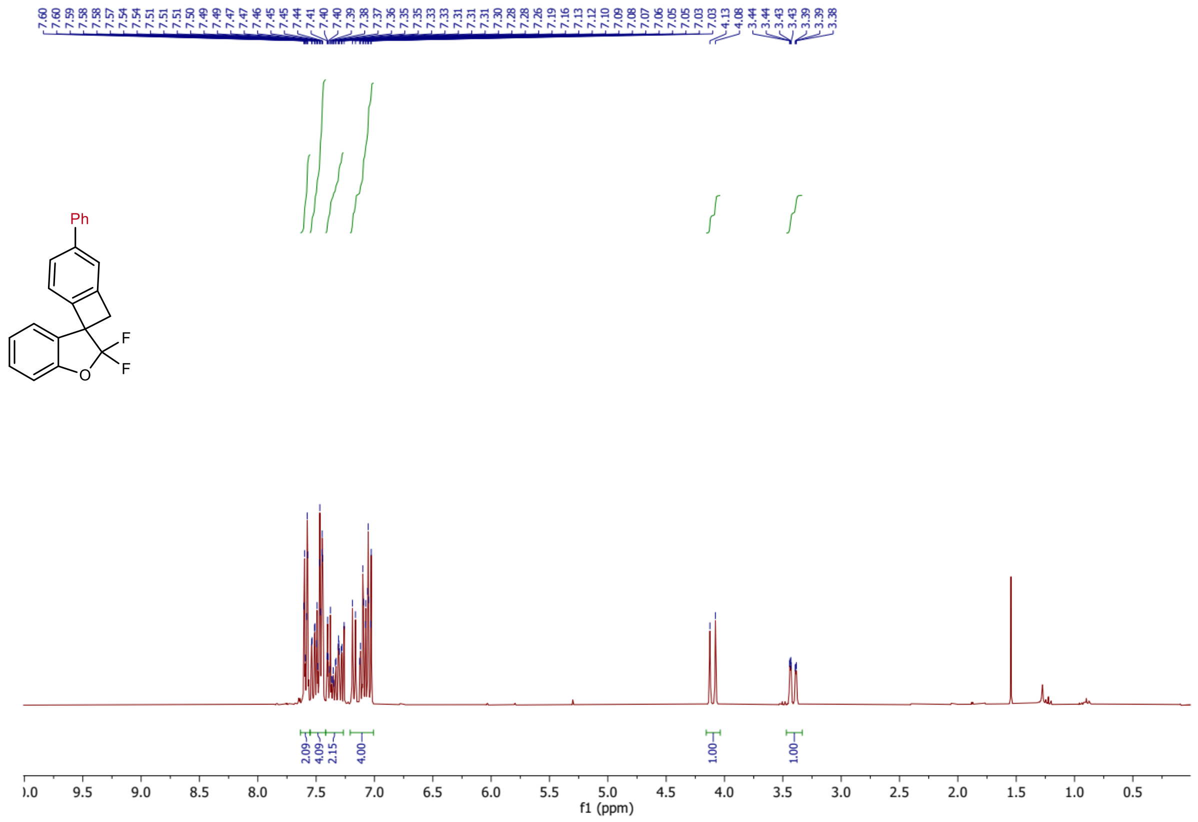
7.60 7.60 7.59 7.58 7.58 7.57 7.54 7.54 7.51 7.51 7.51 7.50 7.49 7.49 7.47 7.47 7.46 7.45 7.45 7.44 7.41 7.40 7.40 7.39 7.38 7.37 7.36 7.35 7.35 7.33 7.33 7.31 7.31 7.30 7.28 7.28 7.26 7.19 7.16 7.13 7.12 7.10 7.09 7.08 7.07 7.06 7.05 7.05 7.03 7.03 4.13 4.08 3.44 3.44 3.43 3.43 3.39 3.39 3.38
Ph
F
F
O
2.09
4.09
2.15
4.00
1.00
1.00
f1 (ppm)

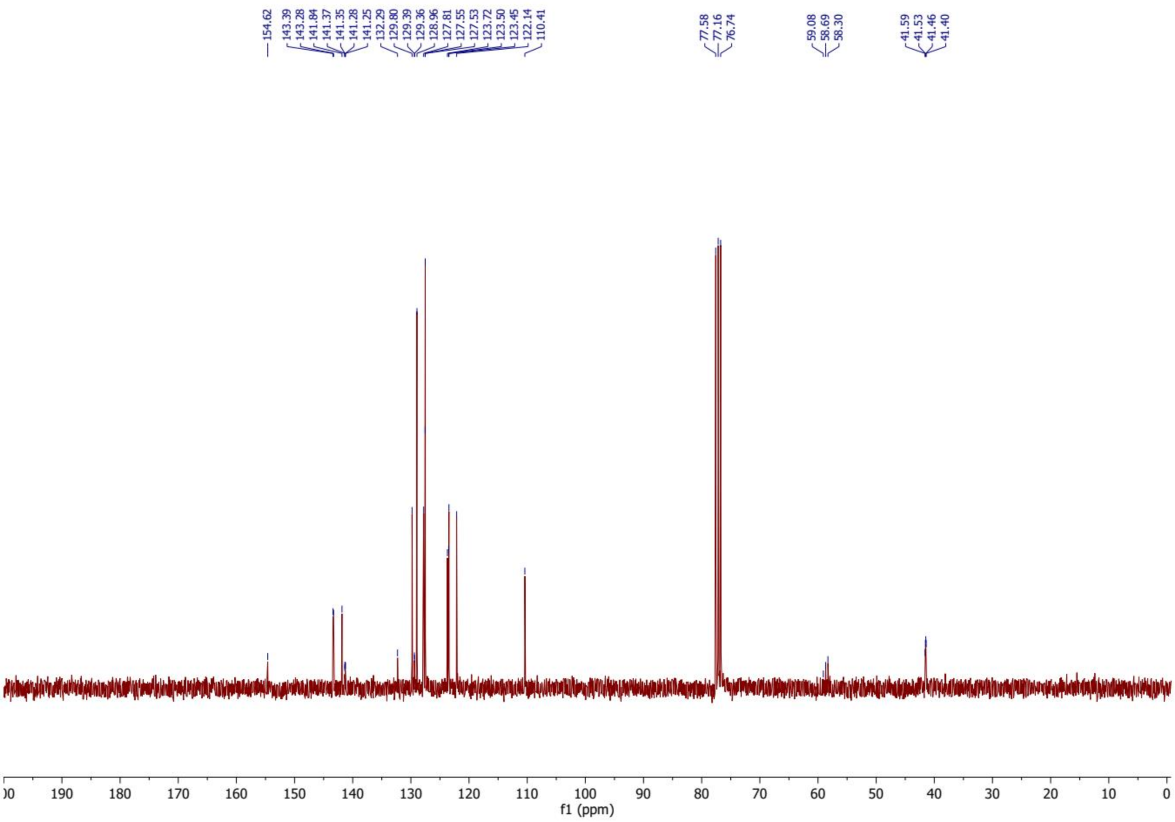
154.62
143.39
143.28
141.84
141.37
141.35
141.28
141.25
132.29
129.80
129.39
129.36
128.96
127.81
127.55
127.53
123.72
123.50
123.45
122.14
110.41
77.58
77.16
76.74
59.08
58.69
58.30
41.59
41.53
41.46
41.40
190 180 170 160 150 140 130 120 110 100 90 80 70 60 50 40 30 20 10 0
f1 (ppm)

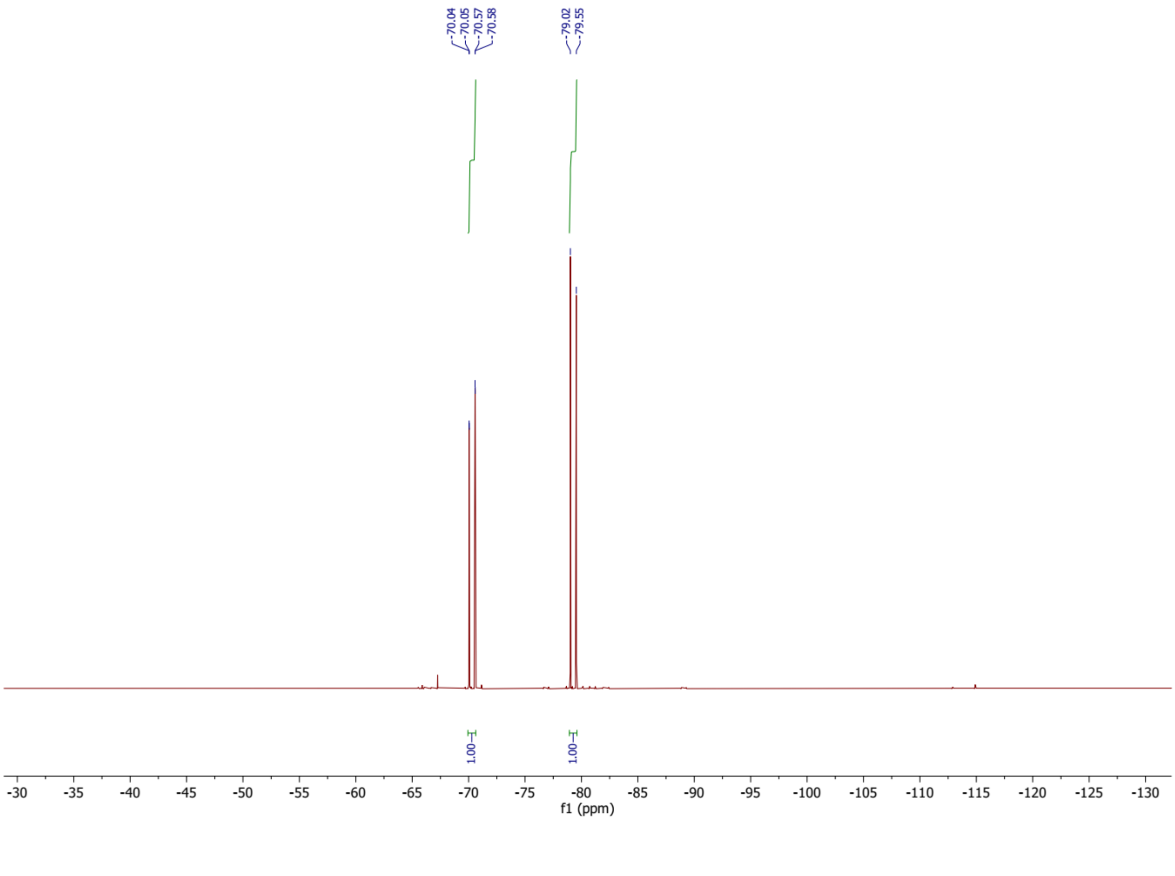
-70.04
-70.05
-70.57
-70.58
-79.02
-79.55
1.00
1.00
-30 -35 -40 -45 -50 -55 -60 -65 -70 -75 -80 -85 -90 -95 -100 -105 -110 -115 -120 -125 -130
f1 (ppm)

4g

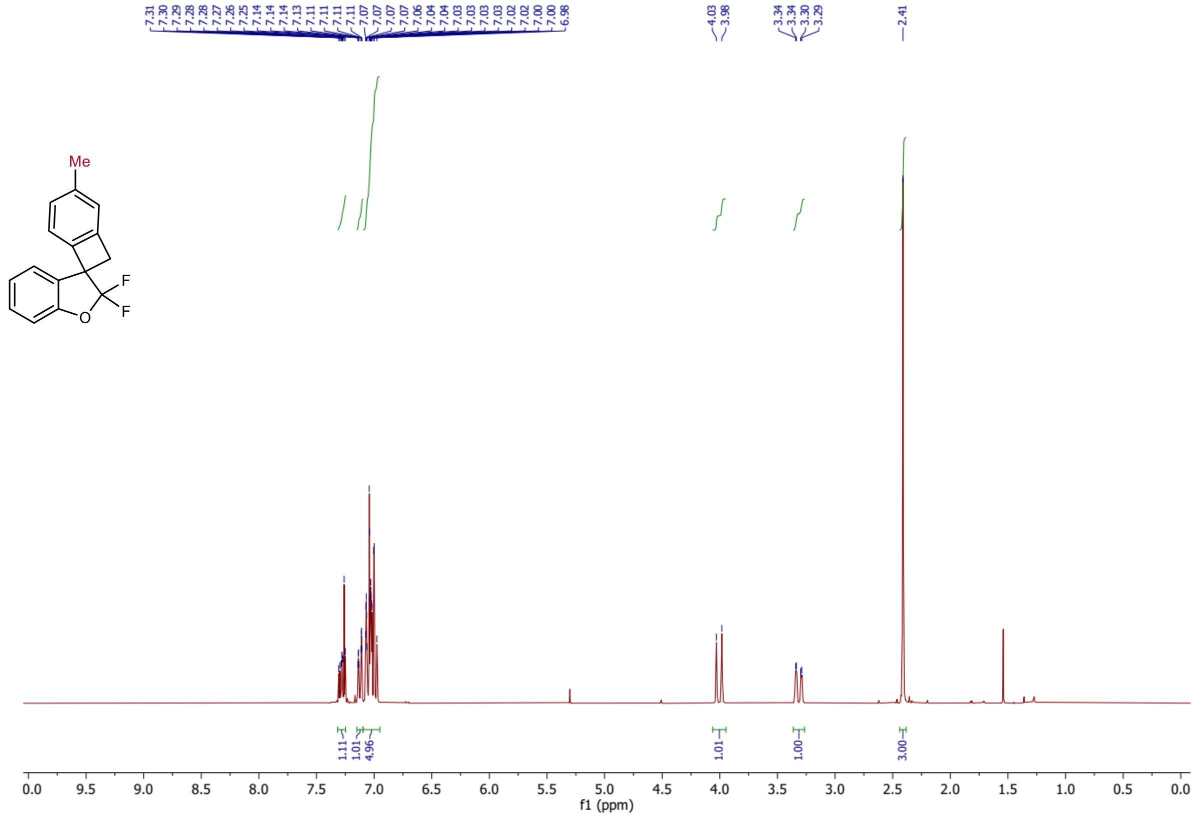

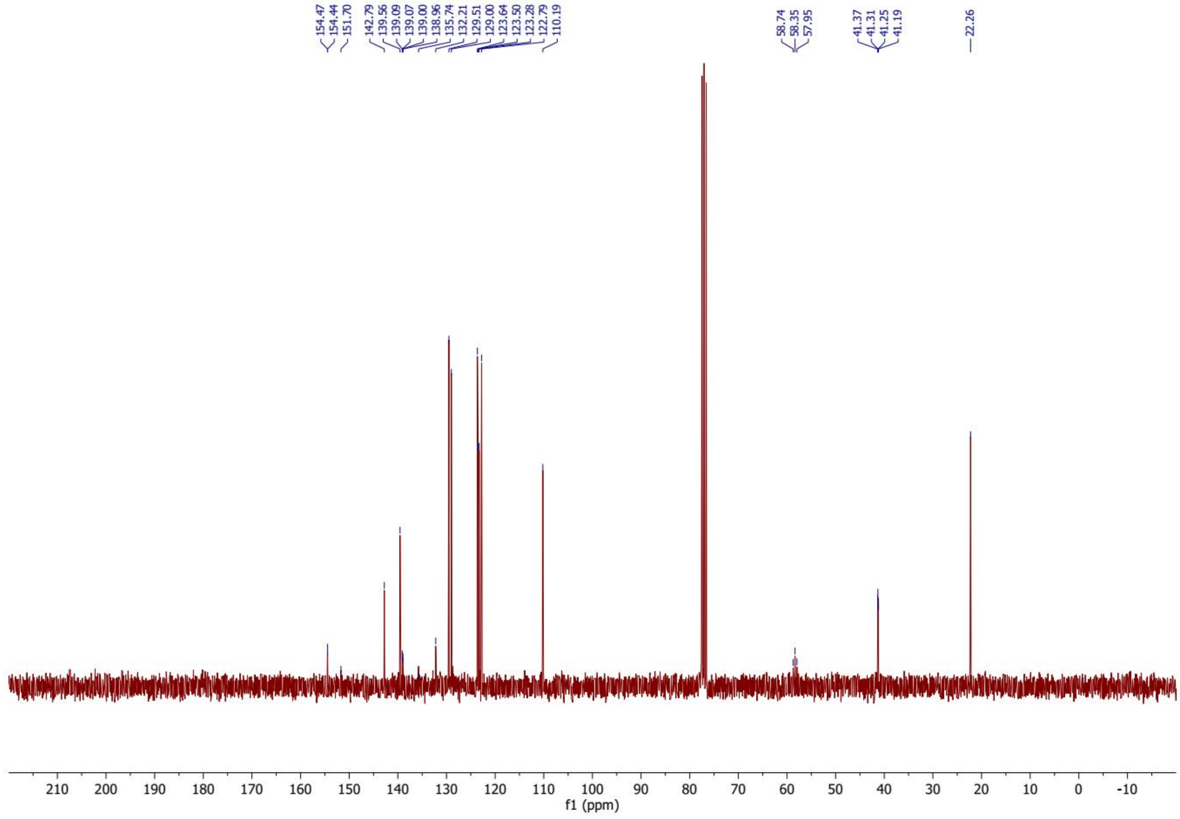

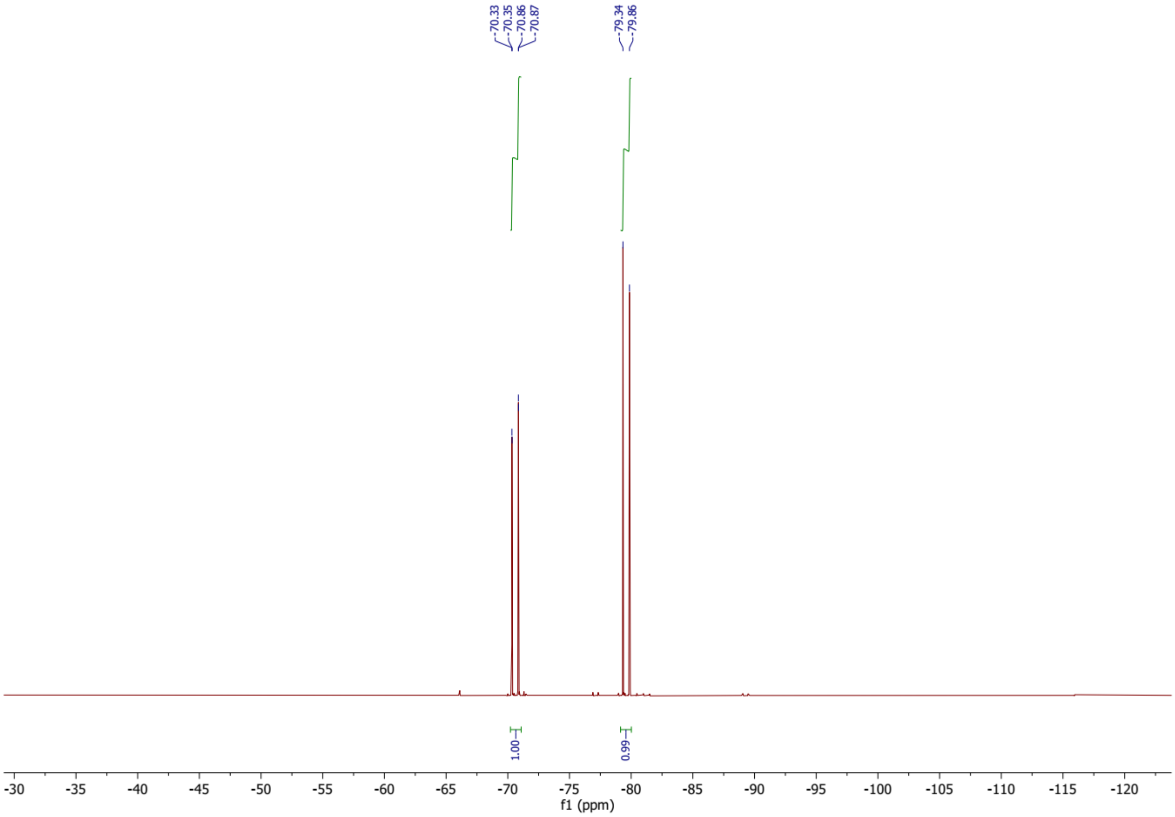
-70.33
-70.35
-70.86
-70.87
-79.34
-79.86
1.00
0.99
f1 (ppm)

4h

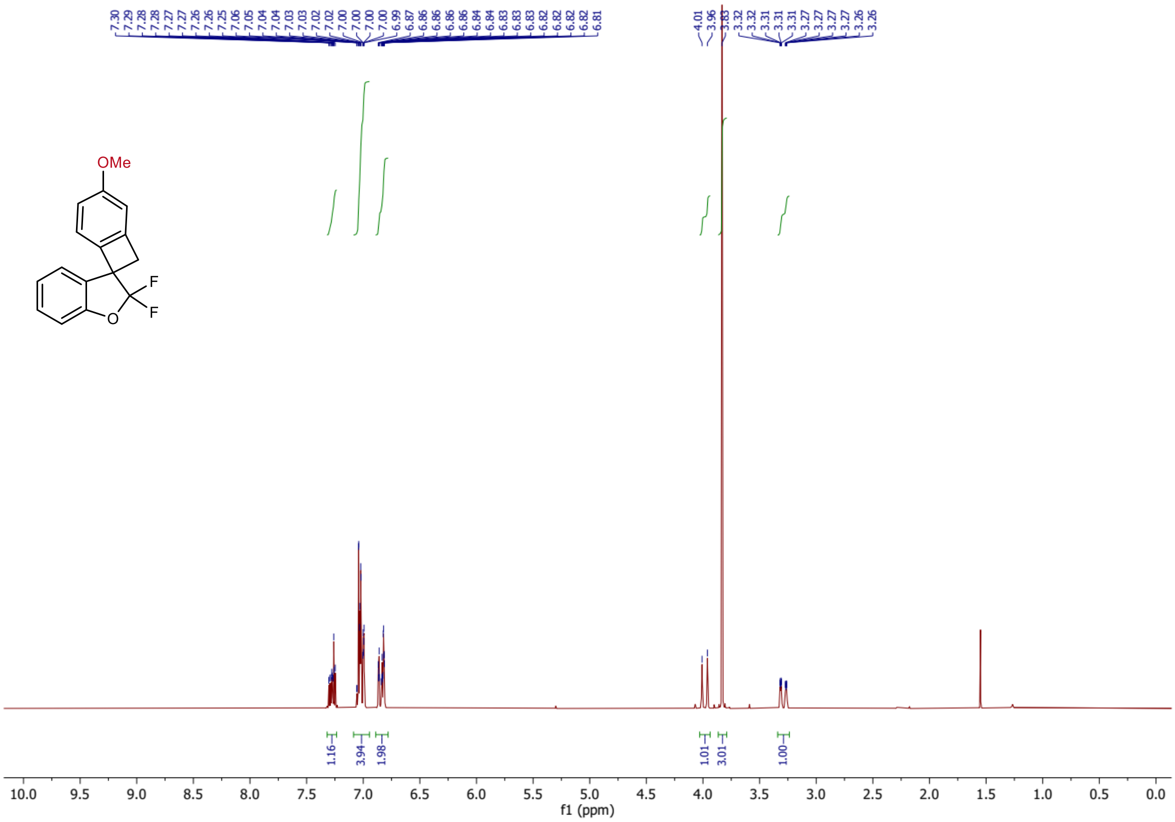
7.30
7.29
7.28
7.28
7.27
7.27
7.26
7.26
7.25
7.06
7.05
7.04
7.04
7.03
7.03
7.02
7.02
7.00
7.00
7.00
7.00
6.99
6.87
6.86
6.86
6.86
6.86
6.84
6.84
6.83
6.83
6.83
6.82
6.82
6.82
6.82
6.81
4.01
3.96
3.83
3.32
3.32
3.31
3.31
3.27
3.27
3.27
3.27
3.26
OMe
F
F
O
1.16
3.94
1.98
1.01
3.01
1.00
f1 (ppm)

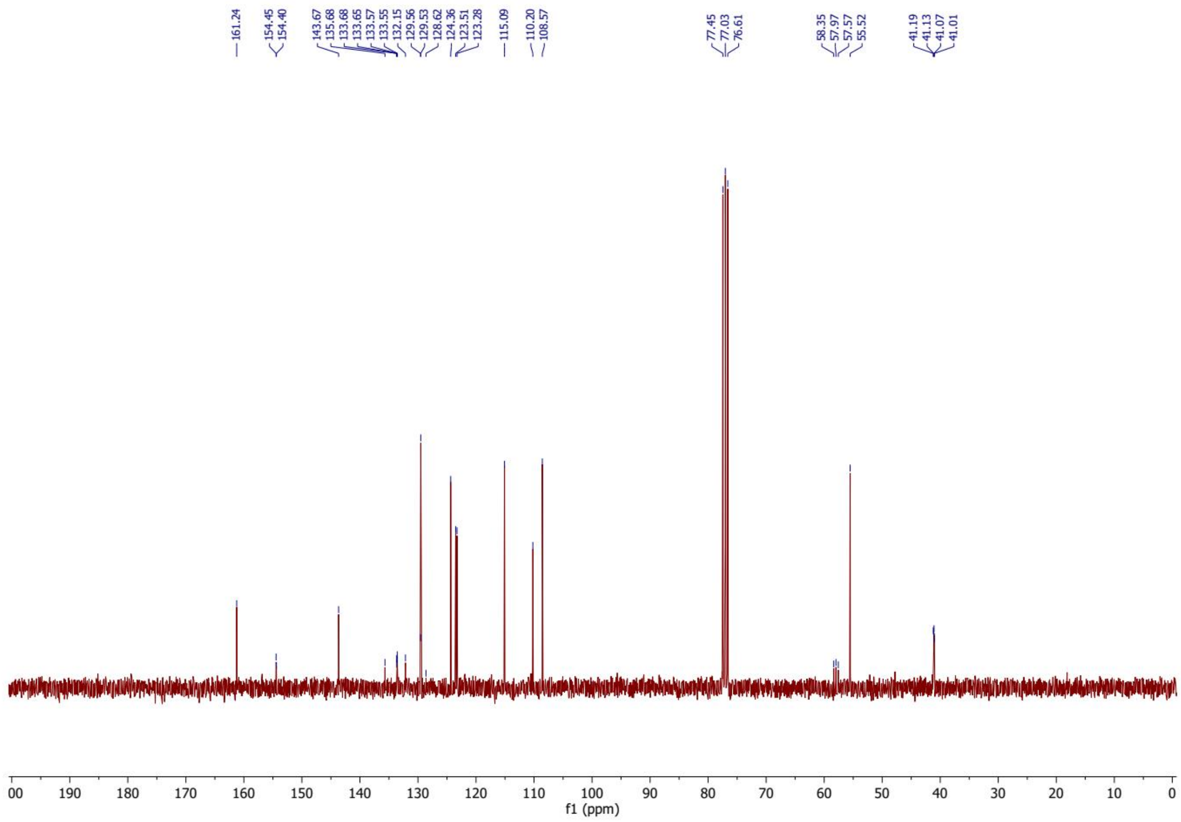
161.24
154.45
154.40
143.67
135.68
133.68
133.65
133.57
133.55
132.15
129.56
129.53
128.62
124.36
123.51
123.28
115.09
110.20
108.57
77.45
77.03
76.61
58.35
57.97
57.57
55.52
41.19
41.13
41.07
41.01
f1 (ppm)

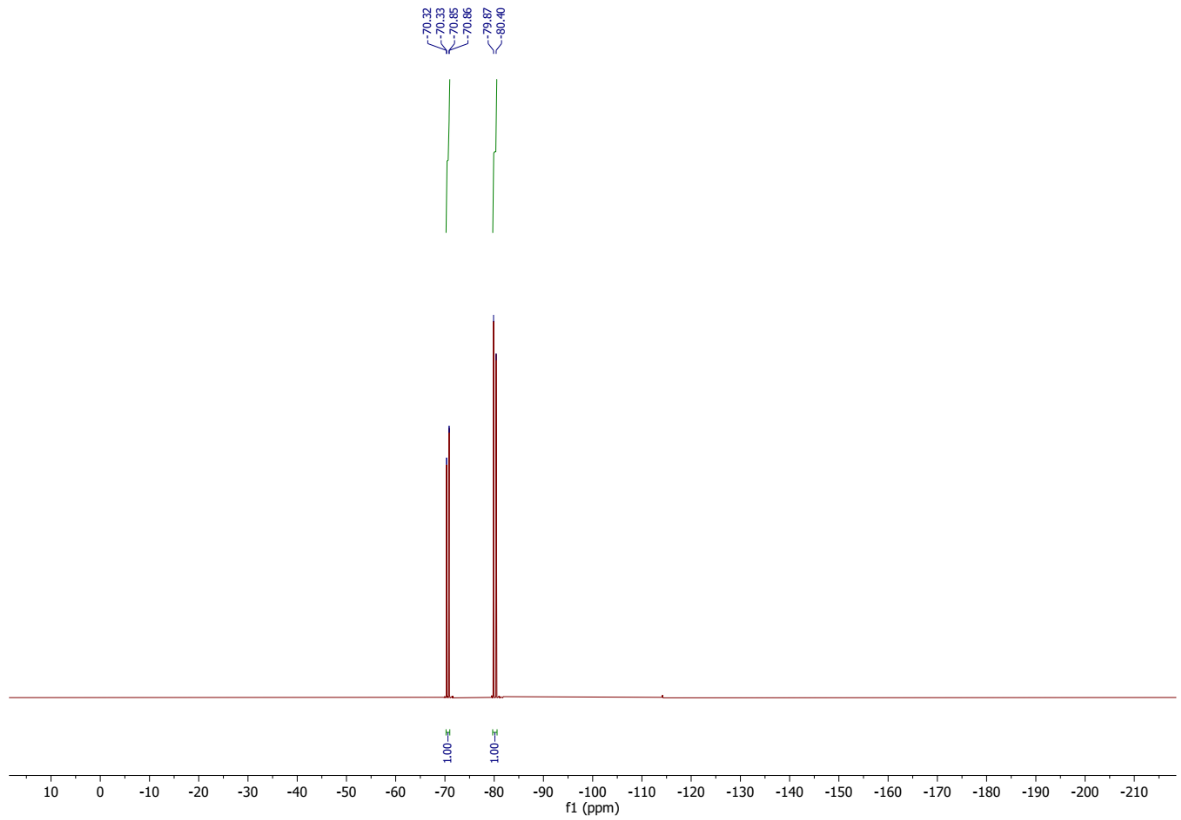
-70.32
-70.33
-70.85
-70.86
-79.87
-80.40
1.00
1.00
f1 (ppm)

4i

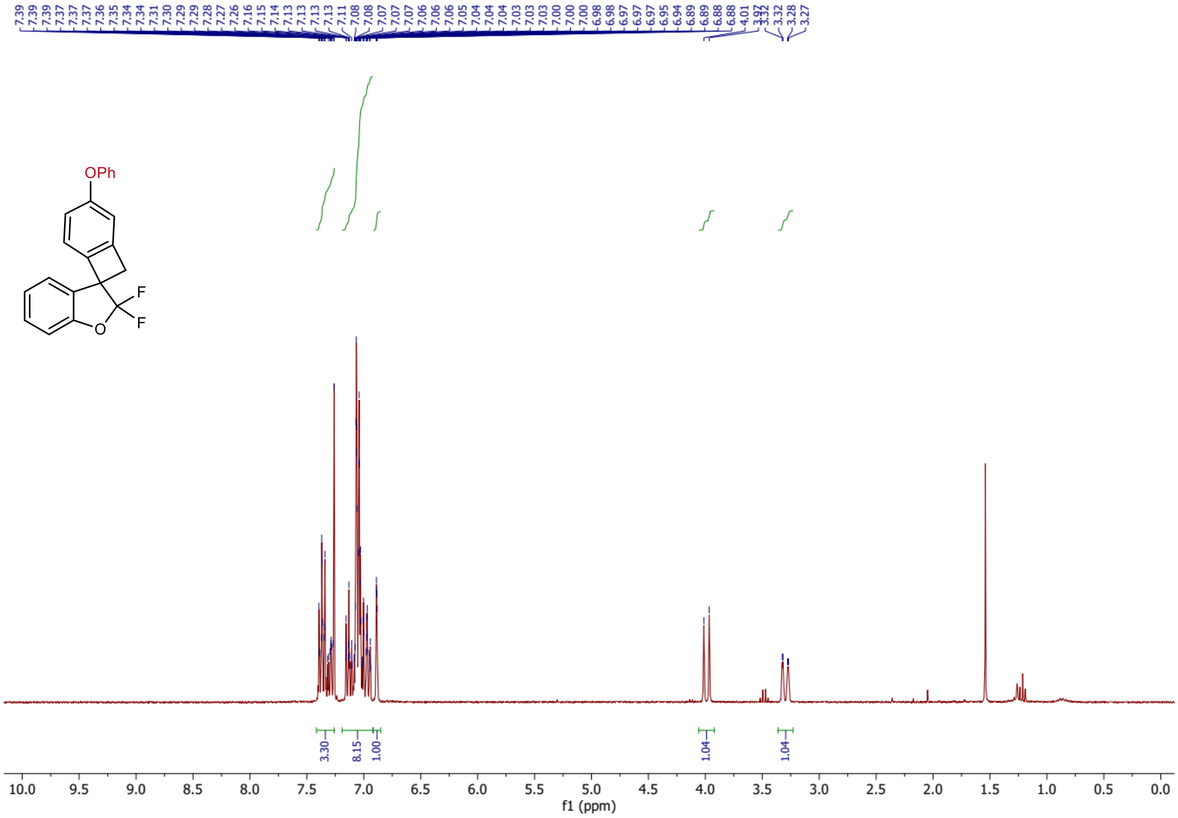

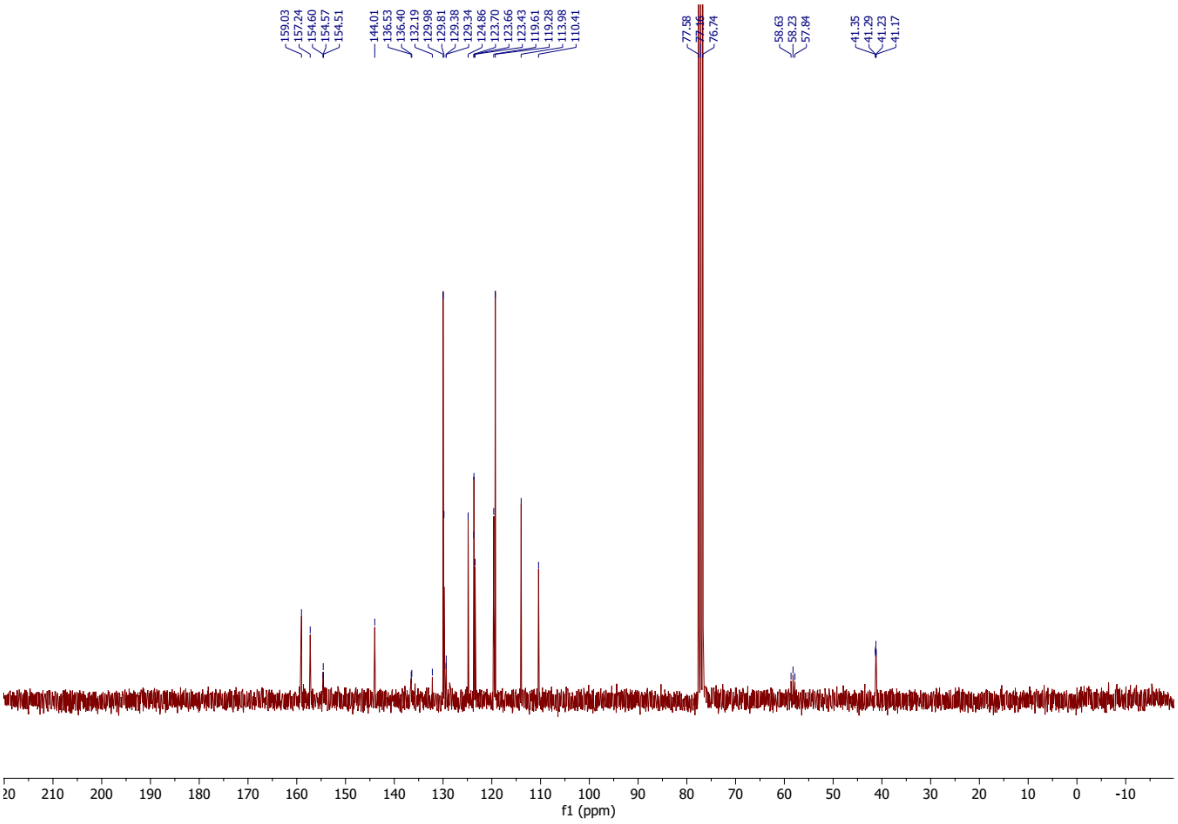

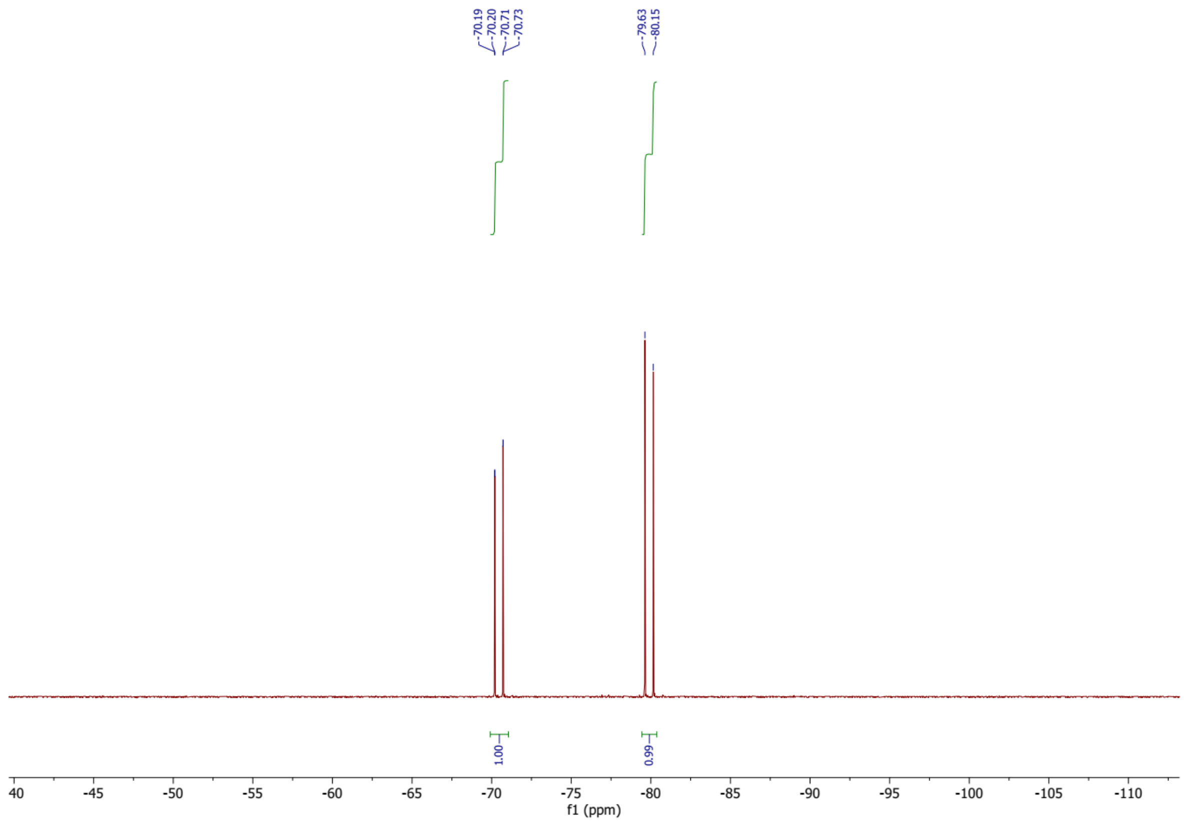
-70.19
-70.20
-70.71
-70.73
-79.63
-80.15
1.00
0.99
f1 (ppm)

4j

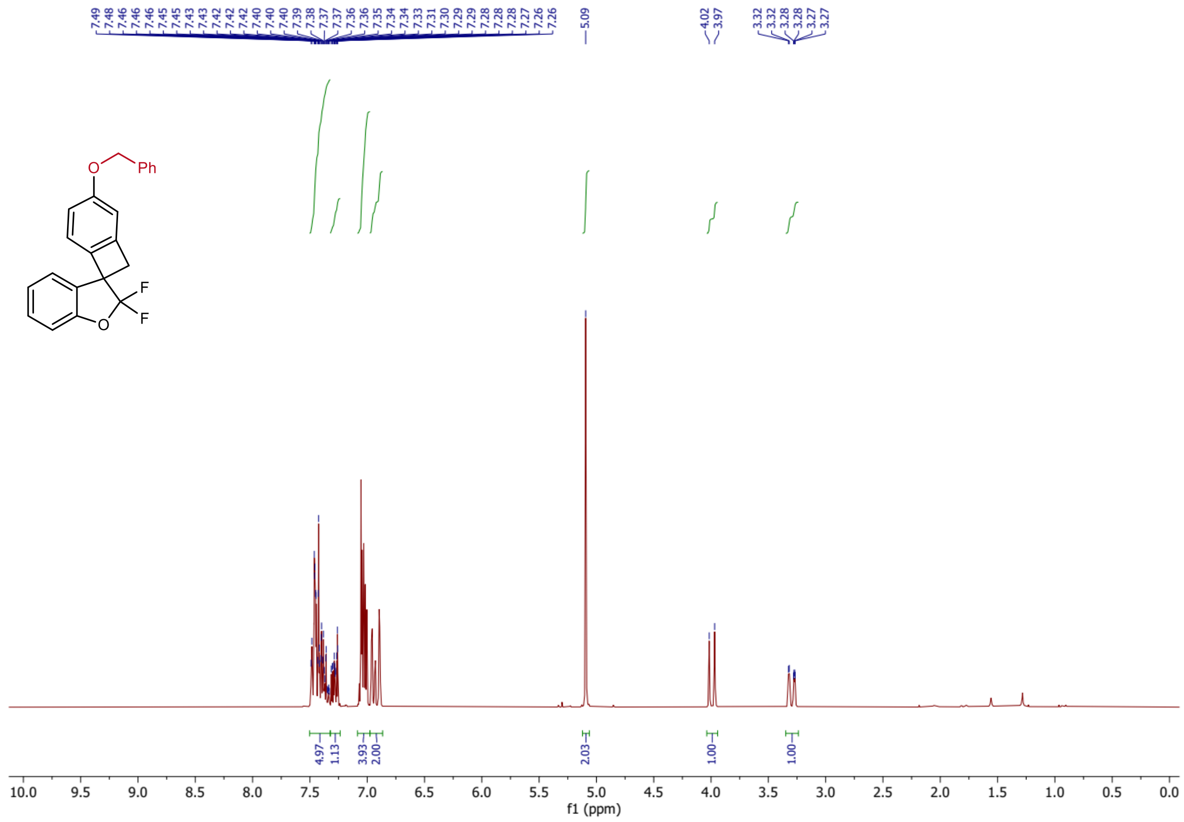
7.49
7.48
7.46
7.46
7.45
7.45
7.43
7.43
7.42
7.42
7.42
7.40
7.40
7.40
7.39
7.38
7.37
7.37
7.36
7.36
7.35
7.34
7.34
7.33
7.31
7.30
7.29
7.29
7.28
7.28
7.28
7.27
7.26
7.26
5.09
4.02
3.97
3.32
3.32
3.28
3.28
3.27
3.27
O
Ph
F
F
O
4.97
1.13
3.93
2.00
2.03
1.00
1.00
f1 (ppm)

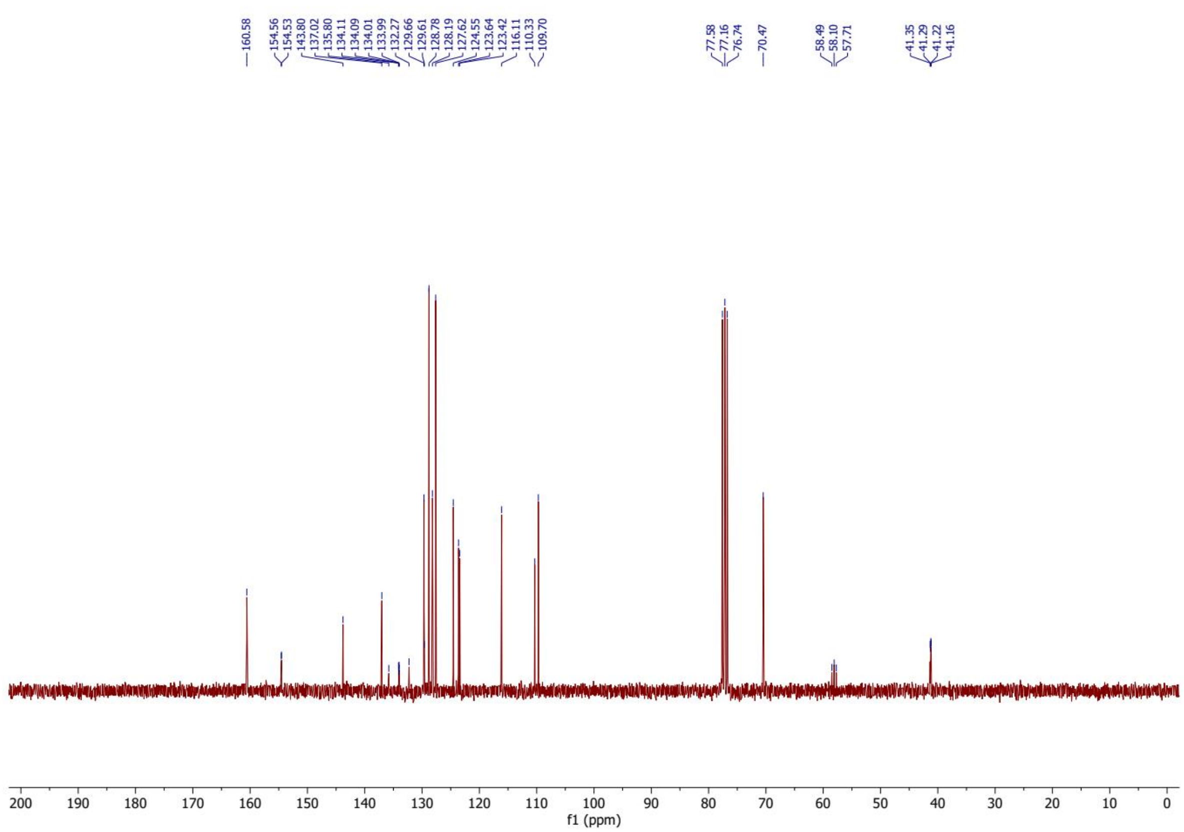
160.58
154.56
154.53
143.80
137.02
135.80
134.11
134.09
134.01
133.99
132.27
129.66
129.61
128.78
128.19
127.62
124.55
123.64
123.42
116.11
110.33
109.70
77.58
77.16
76.74
70.47
58.49
58.10
57.71
41.35
41.29
41.22
41.16
f1 (ppm)

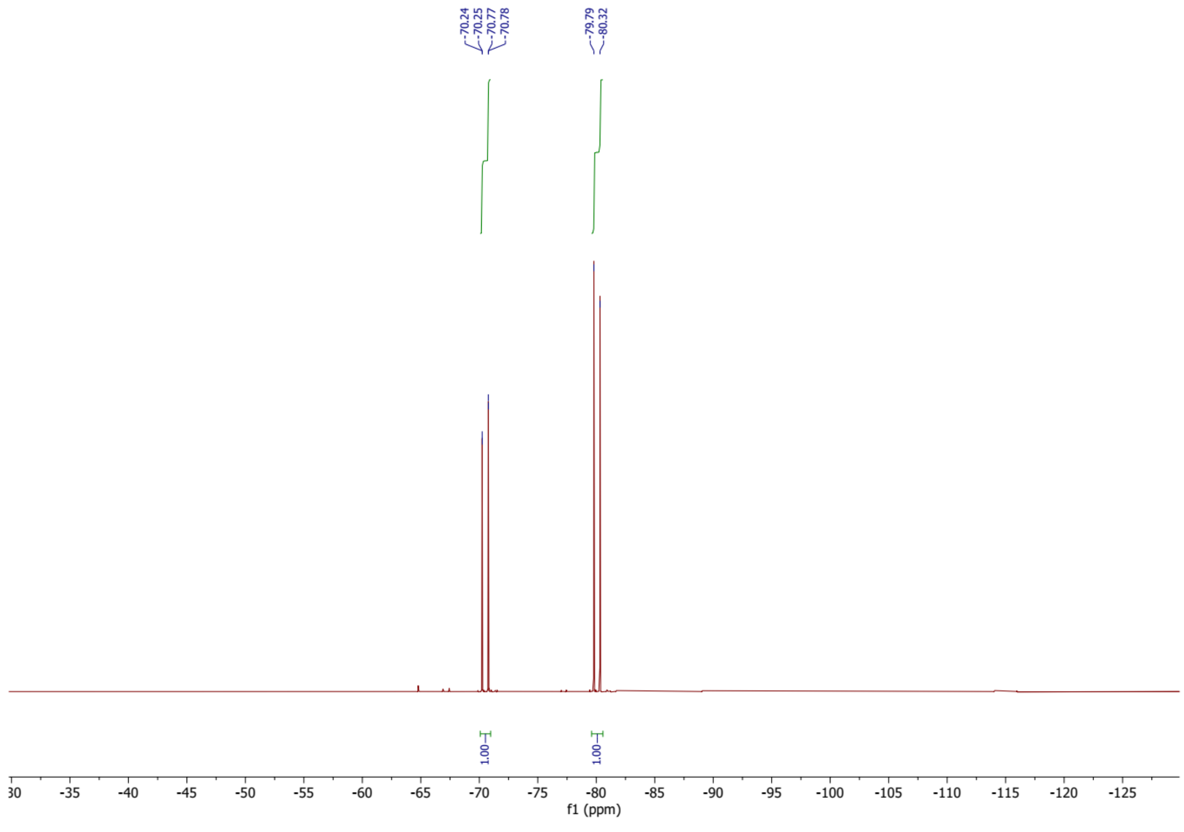
-70.24
-70.25
-70.77
-70.78
-79.79
-80.32
1.00
1.00
f1 (ppm)

4k

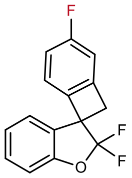

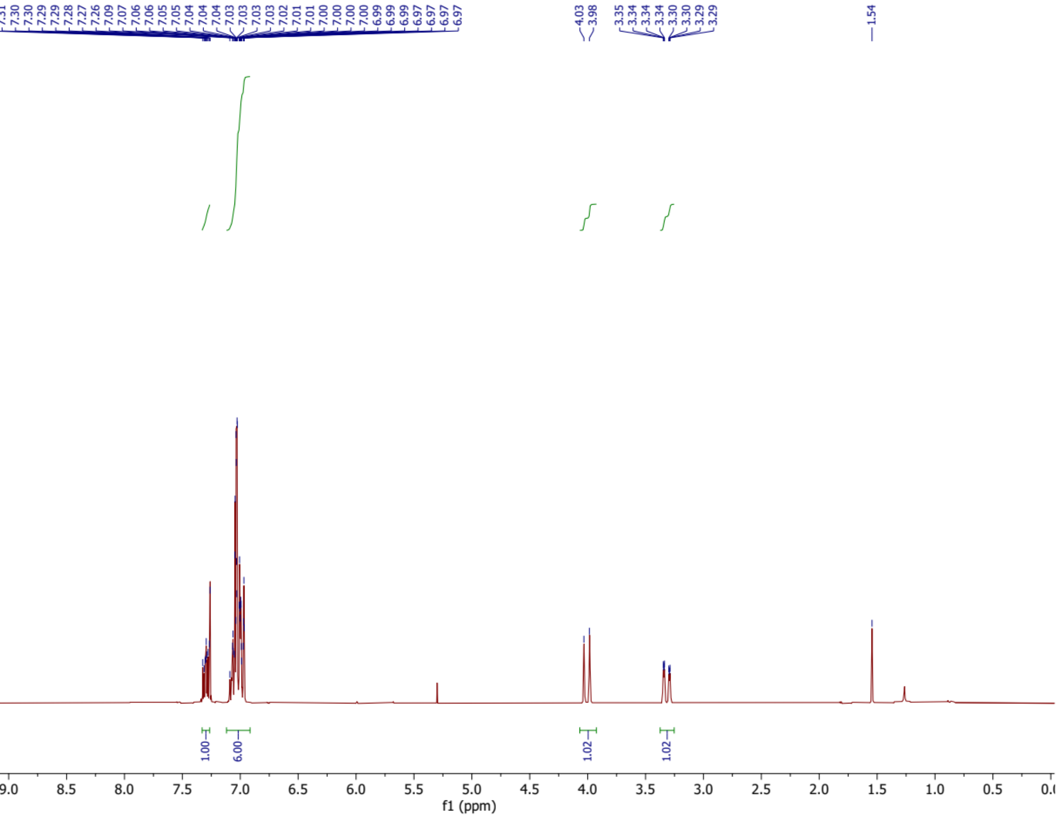
7.32 7.31 7.30 7.29 7.29 7.28 7.27 7.26 7.09 7.07 7.06 7.06 7.05 7.05 7.04 7.04 7.04 7.03 7.03 7.03 7.03 7.02 7.01 7.01 7.00 7.00 7.00 7.00 6.99 6.99 6.99 6.97 6.97 6.97 6.97
4.03 3.98
3.35 3.34 3.34 3.34 3.30 3.30 3.29 3.29
1.54
1.00
6.00
1.02
1.02
f1 (ppm)

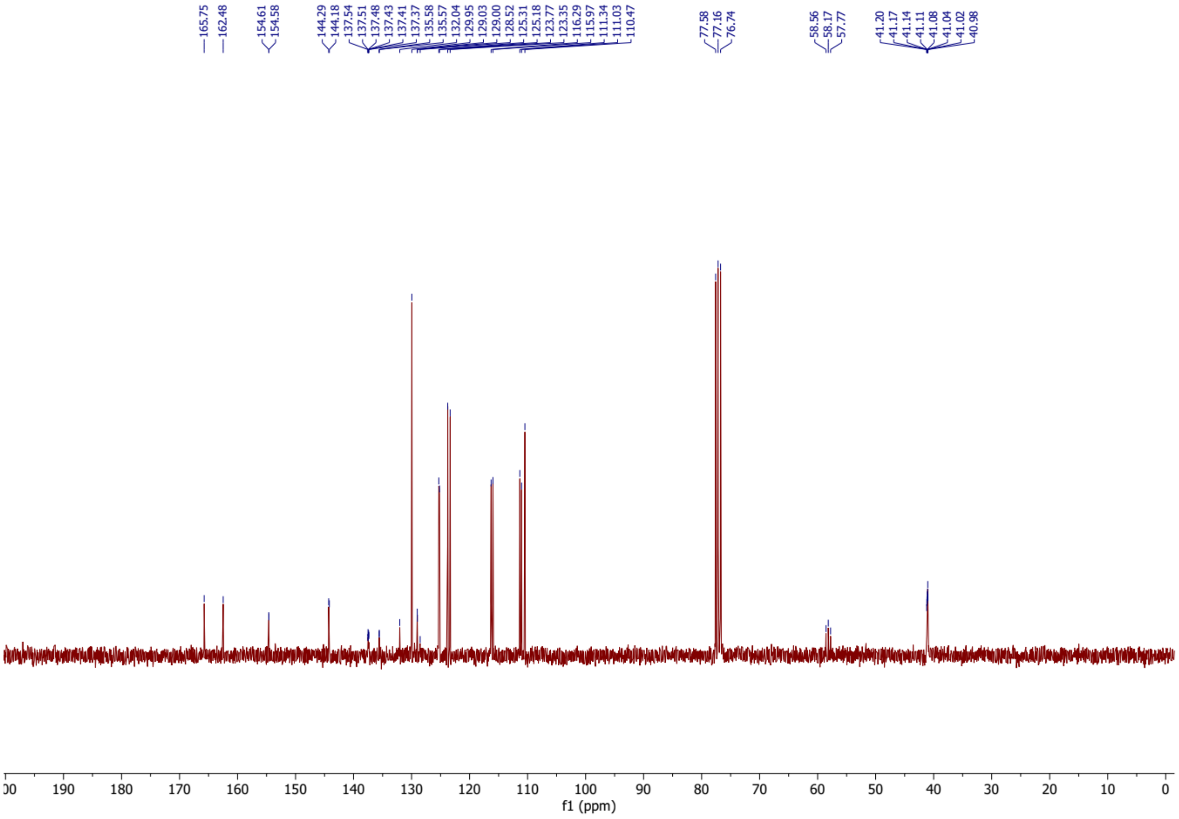
165.75
162.48
154.61 154.58
144.29 144.18 137.54 137.51 137.48 137.43 137.41 137.37 135.58 135.57 132.04 129.95 129.03 129.00 128.52 125.31 125.18 123.77 123.35 116.29 115.97 111.34 111.03 110.47
77.58 77.16 76.74
58.56 58.17 57.77
41.20 41.17 41.14 41.11 41.08 41.04 41.02 40.98
f1 (ppm)

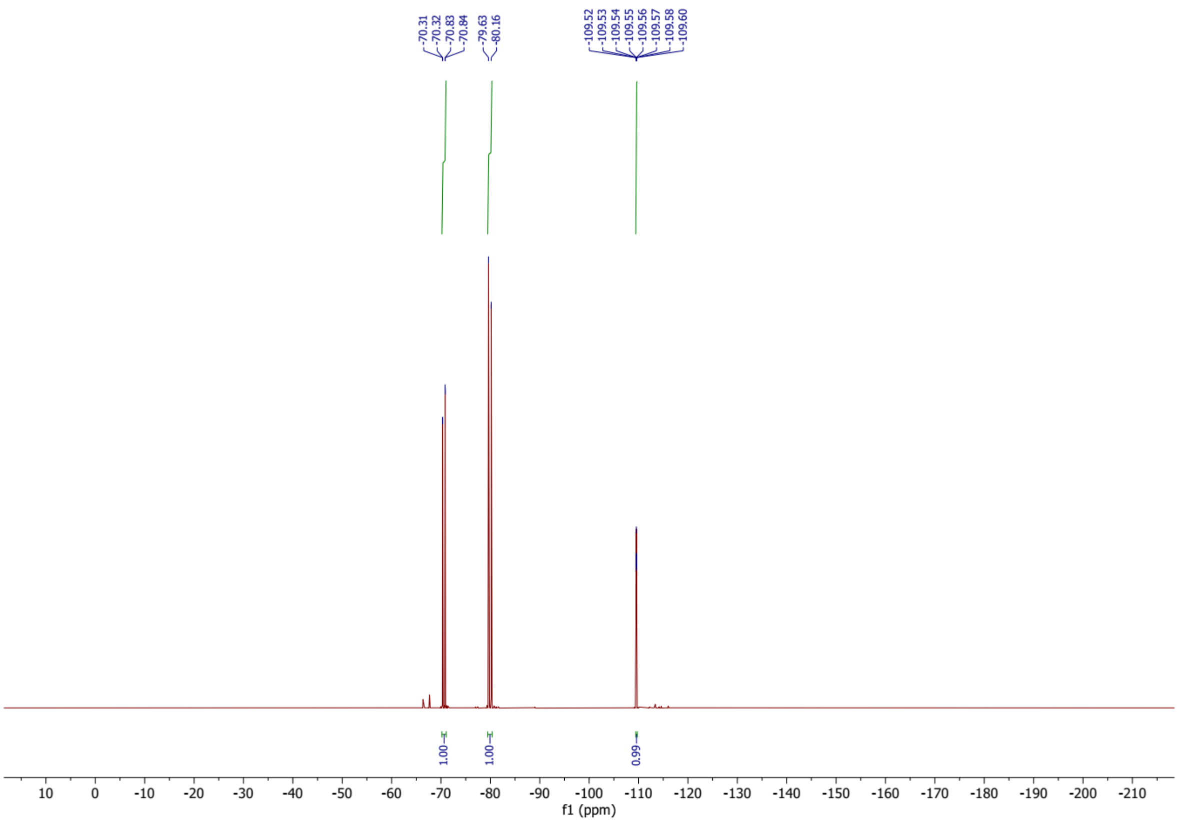

4l

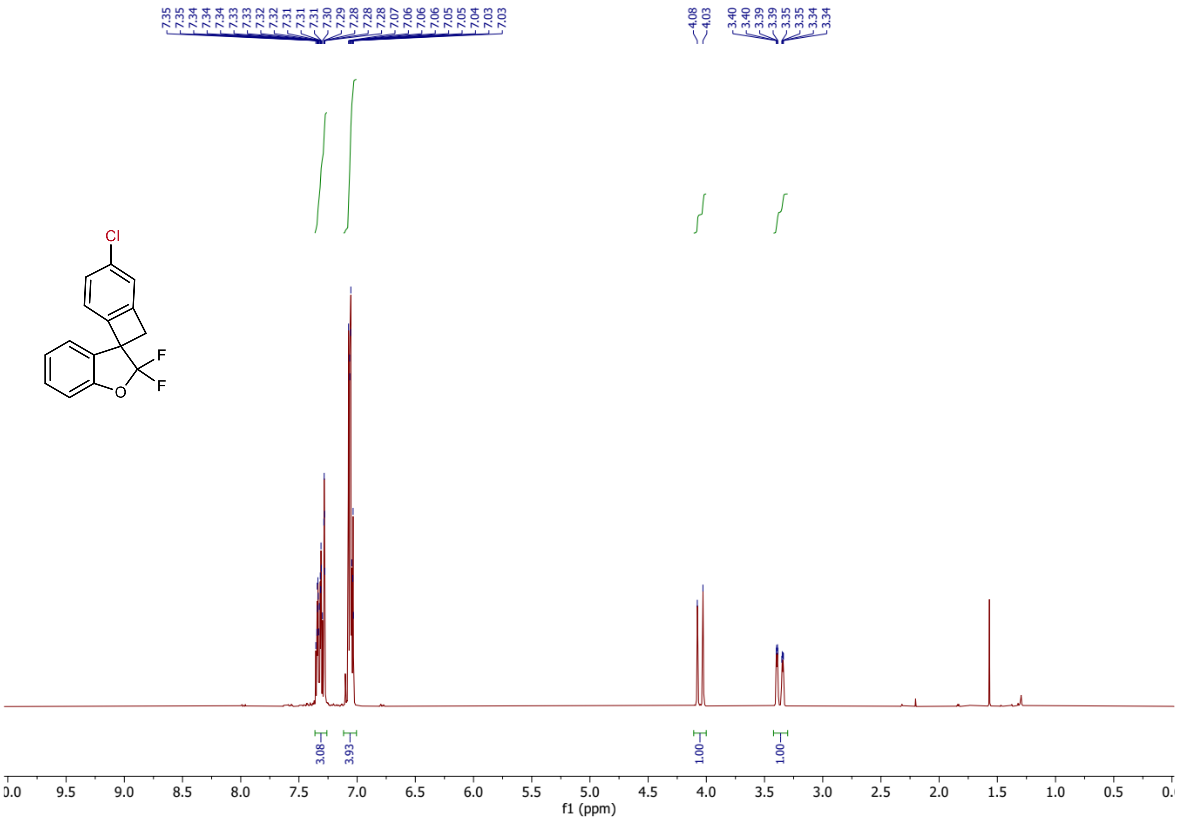

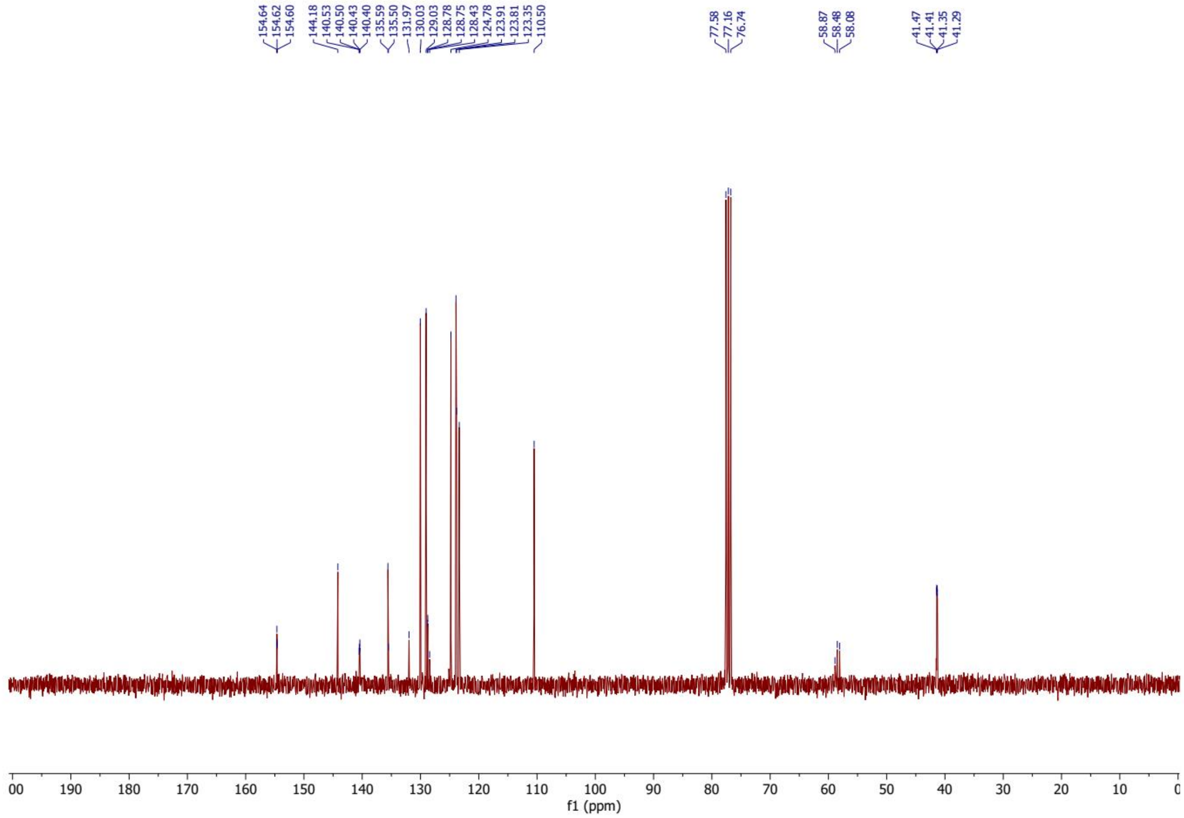
154.64
154.62
154.60
144.18
140.53
140.50
140.43
140.40
135.59
135.50
131.97
130.03
129.03
128.78
128.75
128.43
124.78
123.91
123.81
123.35
110.50
77.58
77.16
76.74
58.87
58.48
58.08
41.47
41.41
41.35
41.29
f1 (ppm)

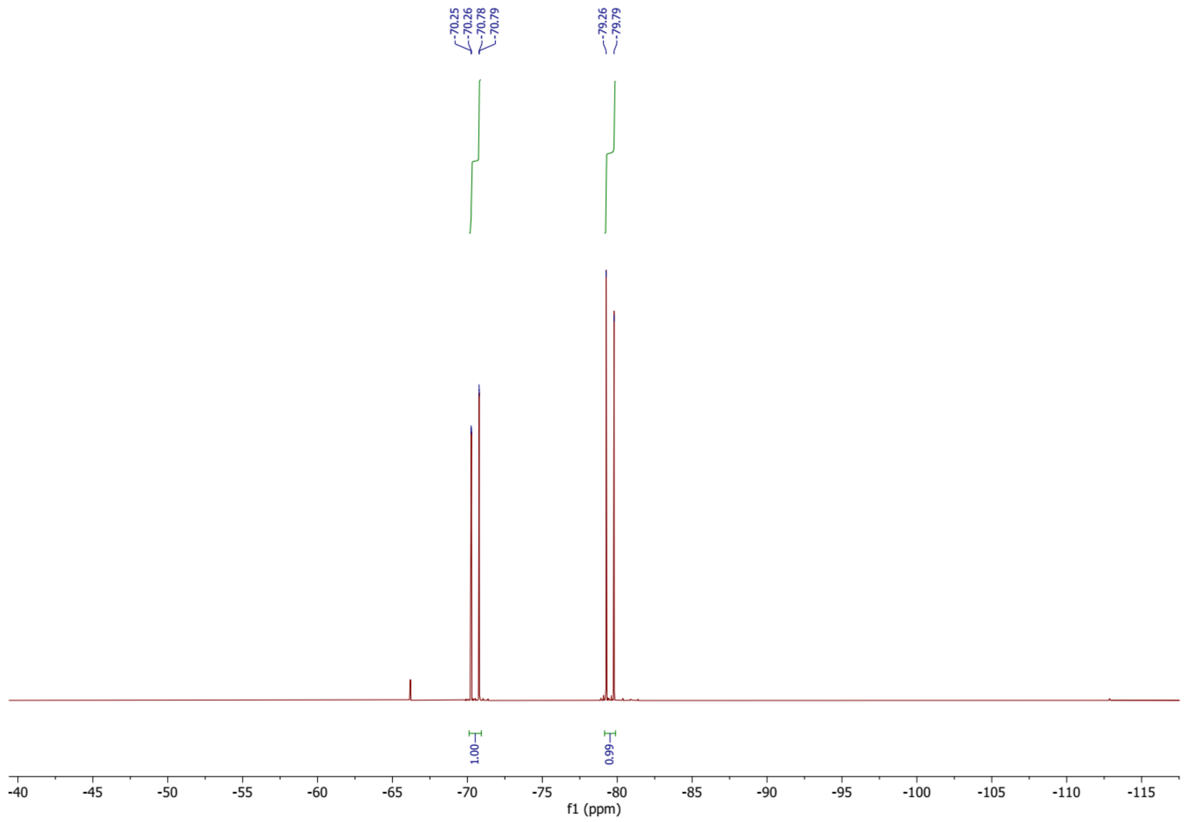
-70.25
-70.26
-70.78
-70.79
-79.26
-79.79
1.00
0.99
f1 (ppm)

4m

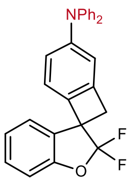
NPh2
F
F
O

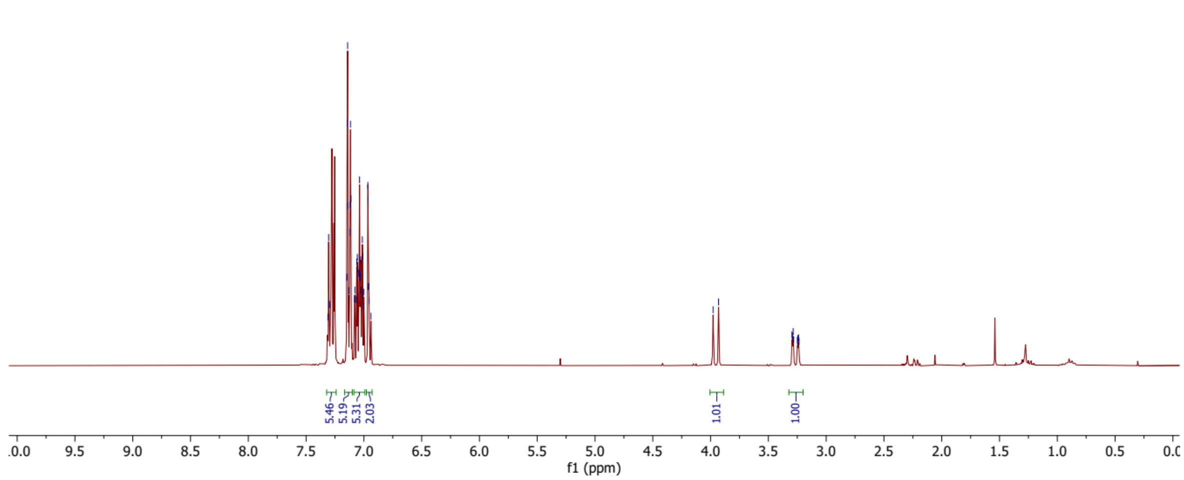
7.31 7.31 7.30 7.30 7.15 7.14 7.14 7.14 7.13 7.12 7.12 7.12 7.11 7.08 7.08 7.07 7.06 7.06 7.05 7.04 7.04 7.04 7.03 7.03 7.03 7.03 7.02 7.01 7.01 7.00 7.00 6.97 6.96 6.96 6.96 6.95 6.94
3.98 3.93 3.30 3.29 3.29 3.28 3.25 3.25 3.24 3.24 3.23
5.46 5.19 5.31 2.03 1.01 1.00
f1 (ppm)

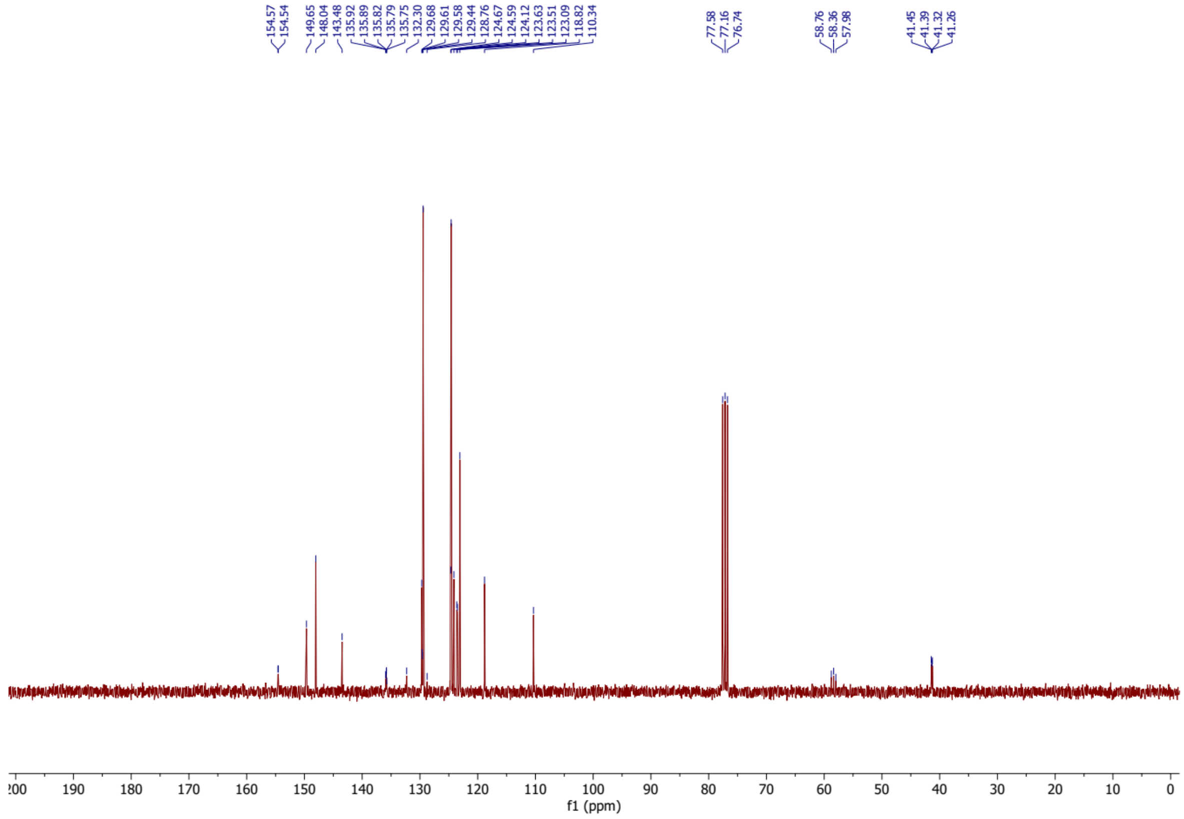
154.57 154.54 149.65 148.04 143.48 135.92 135.89 135.82 135.79 135.75 132.30 129.68 129.61 129.58 129.44 128.76 124.67 124.59 124.12 123.63 123.51 123.09 118.82 110.34
77.58 77.16 76.74
58.76 58.36 57.98
41.45 41.39 41.32 41.26
f1 (ppm)

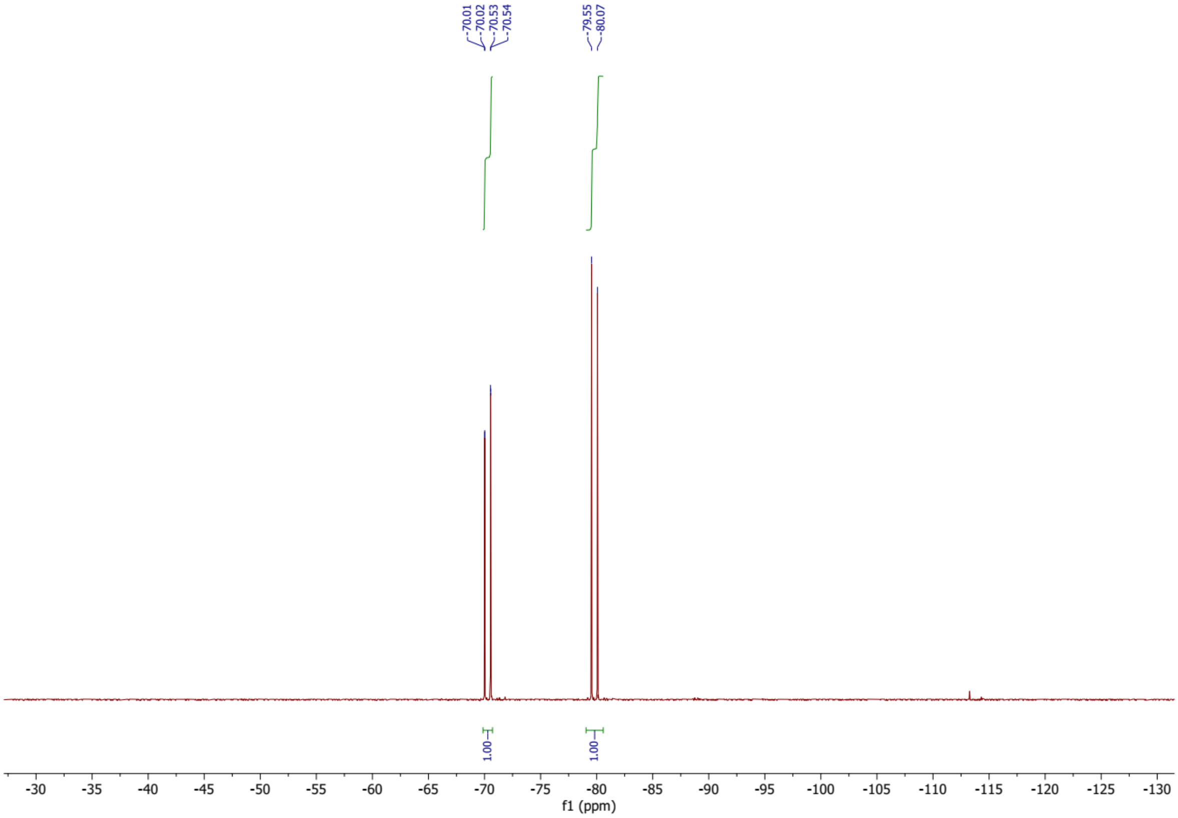

-70.01
-70.02
-70.53
-70.54
-79.55
-80.07
1.00
1.00
f1 (ppm)

4n

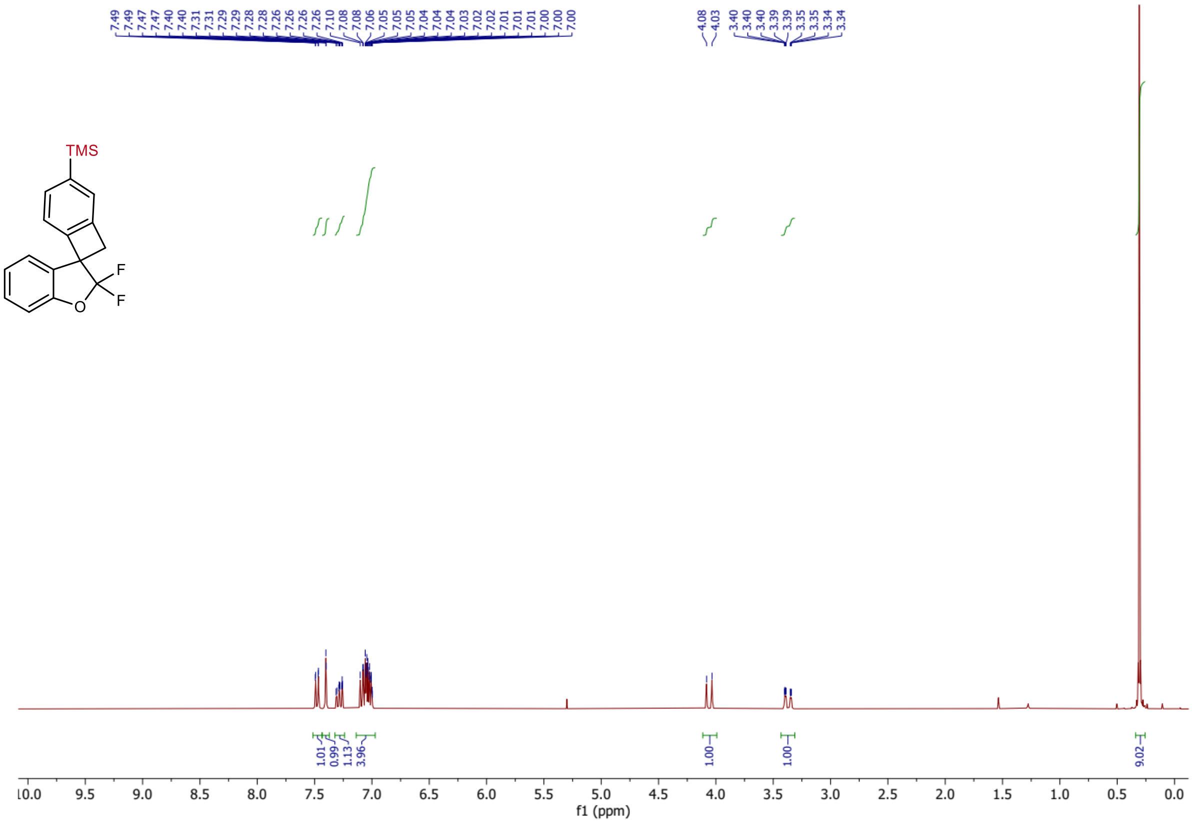

7.49 7.49 7.47 7.47 7.40 7.40 7.31 7.31 7.29 7.29 7.28 7.28 7.26 7.26 7.26 7.10 7.08 7.06 7.05 7.05 7.04 7.04 7.03 7.02 7.02 7.01 7.01 7.00 7.00 7.00
4.08 4.03
3.40 3.40 3.39 3.39 3.35 3.35 3.34 3.34
TMS
F
F
O
1.01
0.99
1.13
3.96
1.00
1.00
9.02
f1 (ppm)

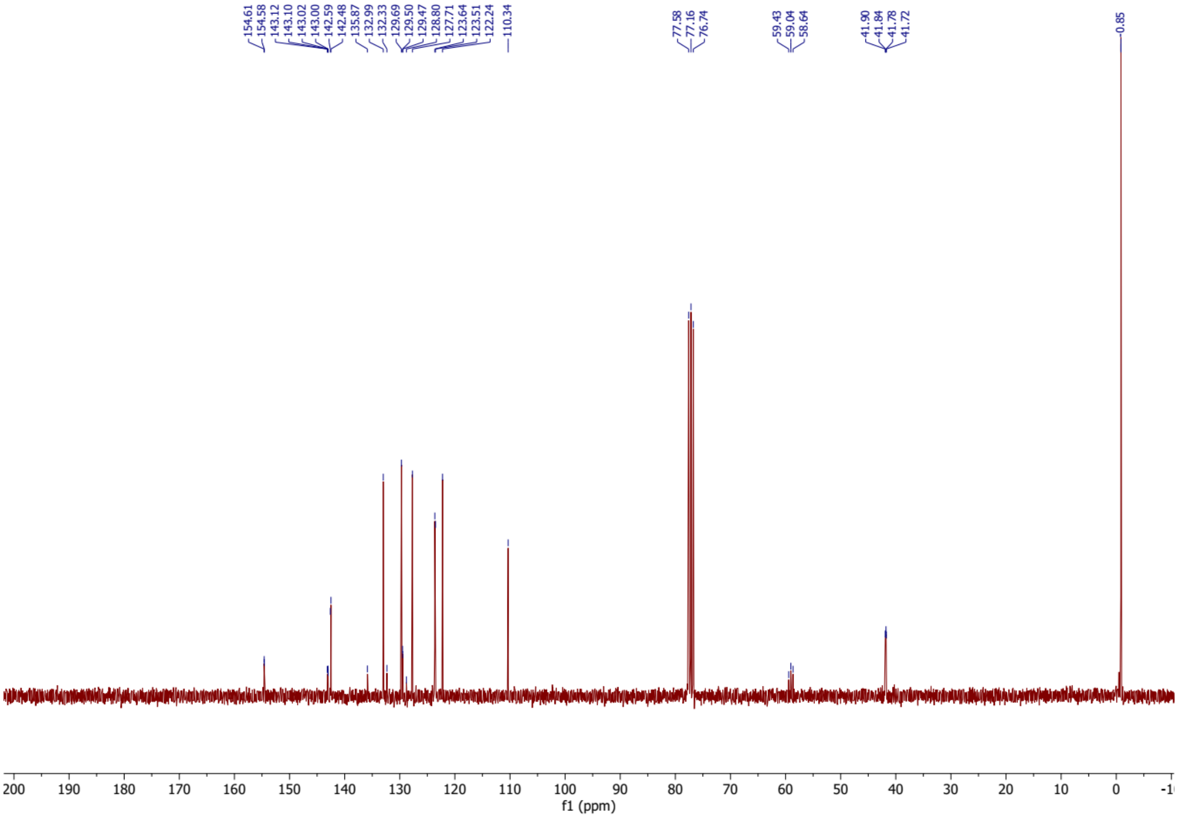
154.61
154.58
143.12
143.10
143.02
143.00
142.59
142.48
135.87
132.99
132.33
129.69
129.50
129.47
128.80
127.71
123.64
123.51
122.24
110.34
77.58
77.16
76.74
59.43
59.04
58.64
41.90
41.84
41.78
41.72
0.85
f1 (ppm)

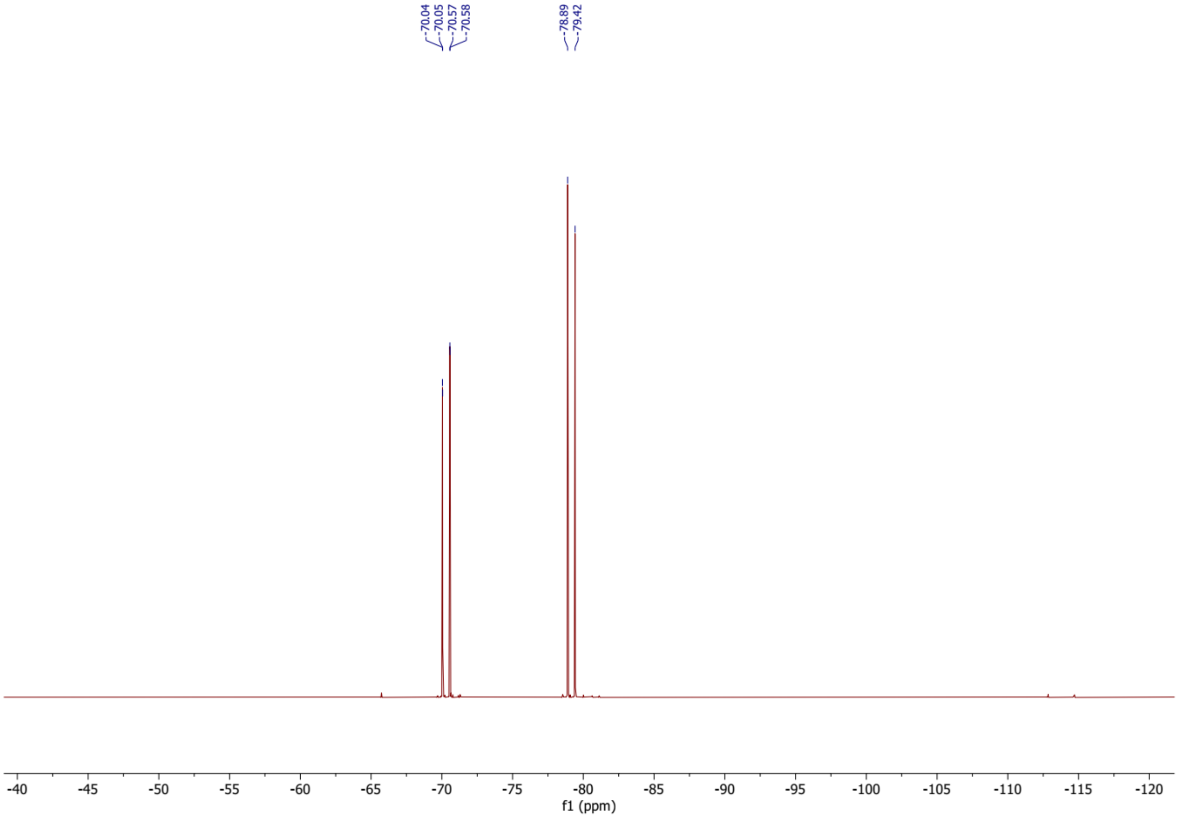
-70.04
-70.05
-70.57
-70.58
-78.89
-79.42
f1 (ppm)

4o

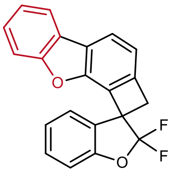
O
O
F
F

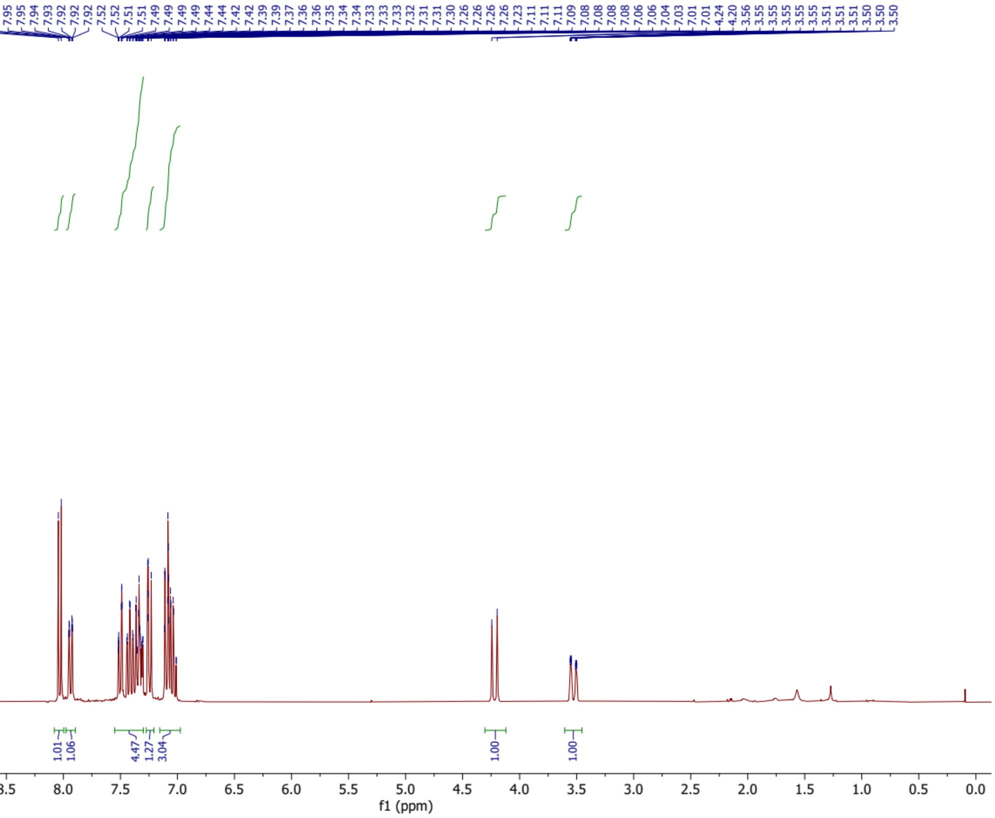
8.04 8.02 7.95 7.95 7.94 7.93 7.92 7.92 7.92 7.52 7.52 7.51 7.51 7.49 7.49 7.49 7.49 7.44 7.44 7.42 7.42 7.39 7.39 7.37 7.36 7.36 7.35 7.34 7.34 7.33 7.33 7.33 7.32 7.31 7.31 7.30 7.26 7.26 7.26 7.23 7.11 7.11 7.11 7.09 7.08 7.08 7.08 7.06 7.06 7.04 7.03 7.01 7.01 4.24 4.20 3.56 3.55 3.55 3.55 3.55 3.51 3.51 3.51 3.50 3.50
1.01 1.06 4.47 1.27 3.04 1.00 1.00
10.0 9.5 9.0 8.5 8.0 7.5 7.0 6.5 6.0 5.5 5.0 4.5 4.0 3.5 3.0 2.5 2.0 1.5 1.0 0.5 0.0
f1 (ppm)

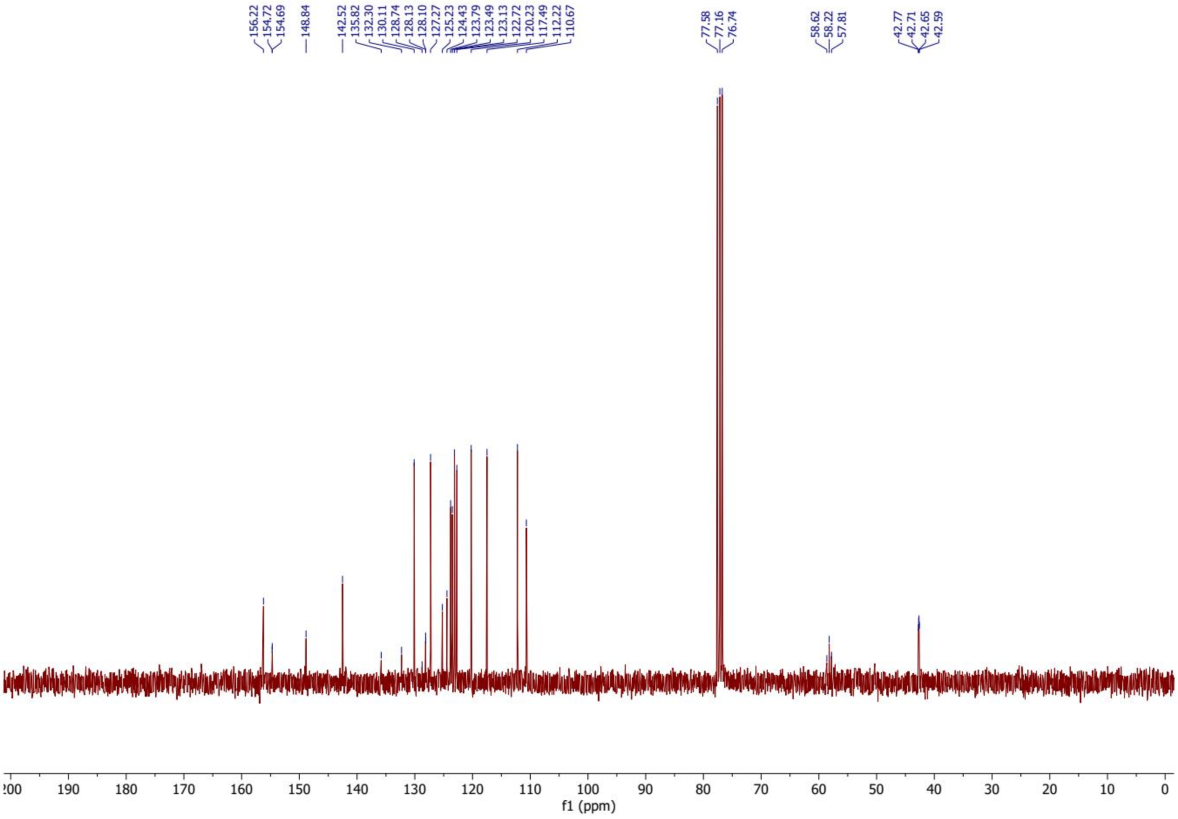
156.22 154.72 154.69 148.84 142.52 135.82 132.30 130.11 128.74 128.13 128.10 127.27 125.23 124.43 123.79 123.49 123.13 122.72 120.23 117.49 112.22 110.67 77.58 77.16 76.74 58.62 58.22 57.81 42.77 42.71 42.65 42.59
200 190 180 170 160 150 140 130 120 110 100 90 80 70 60 50 40 30 20 10 0
f1 (ppm)

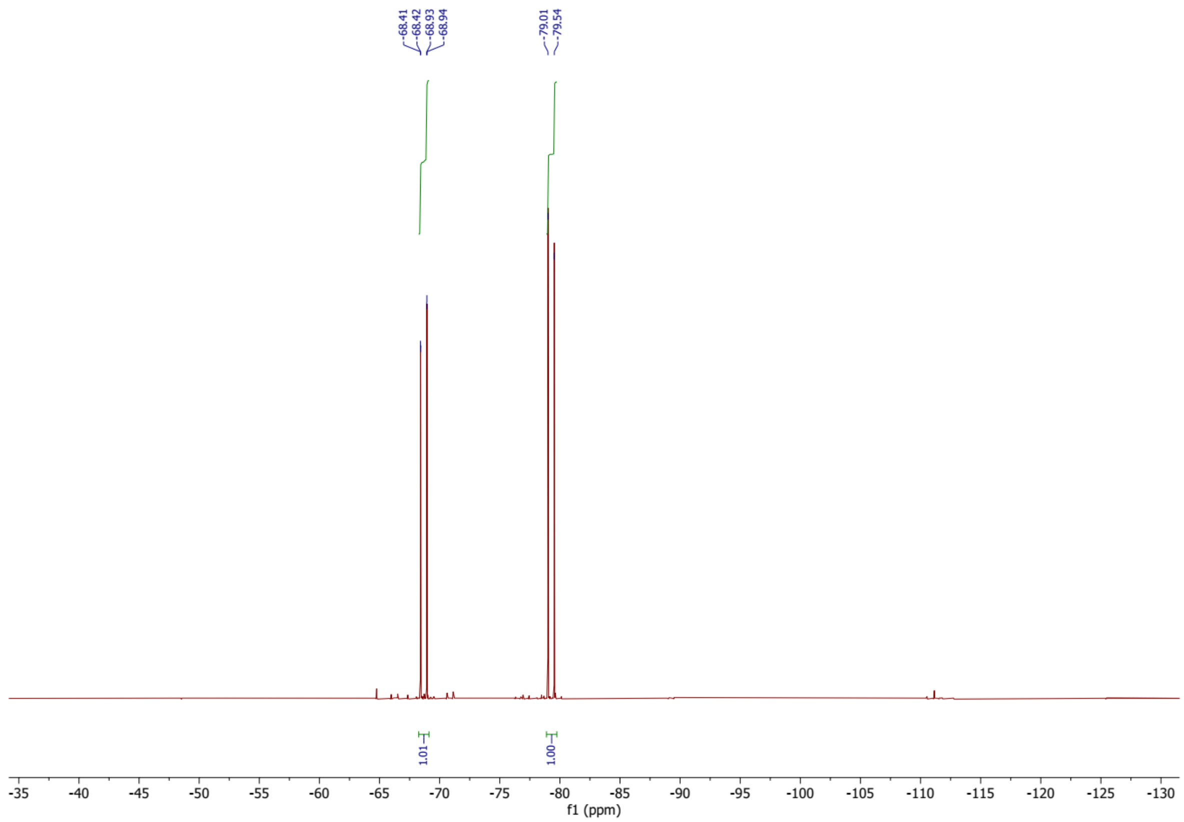

4p

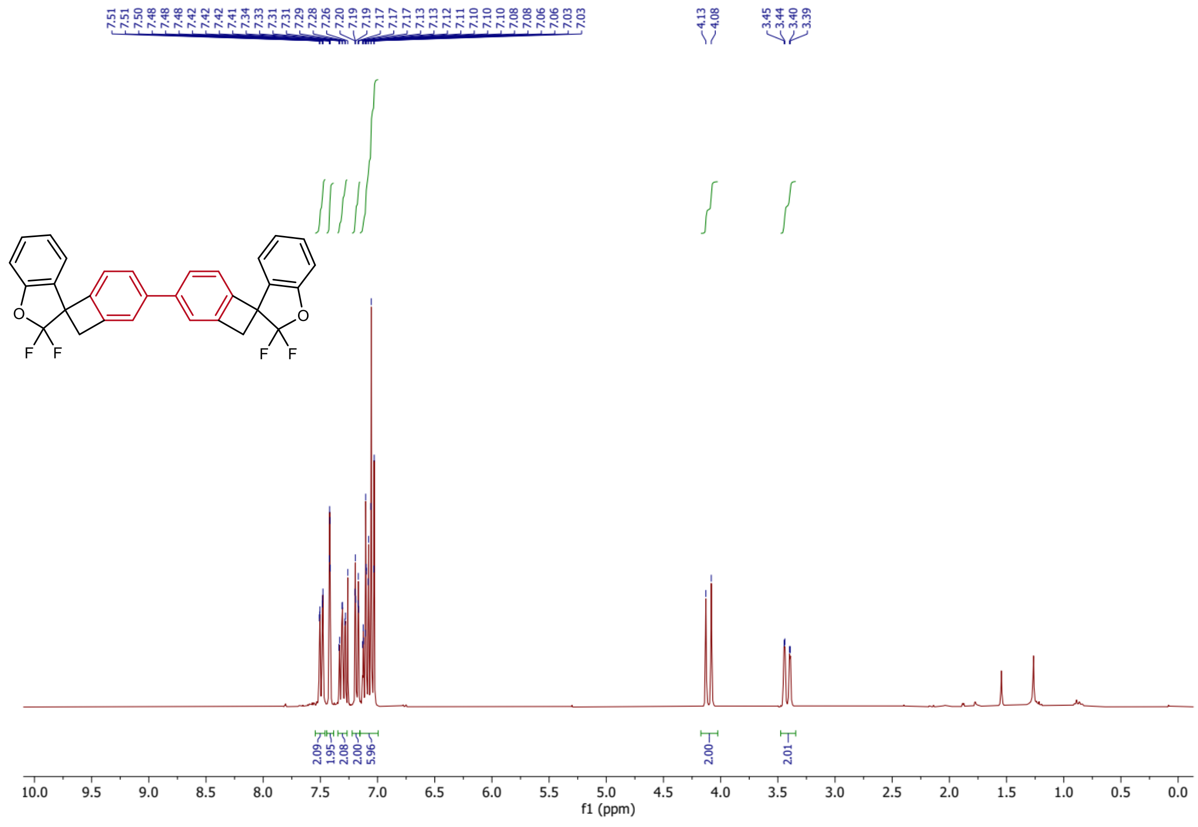

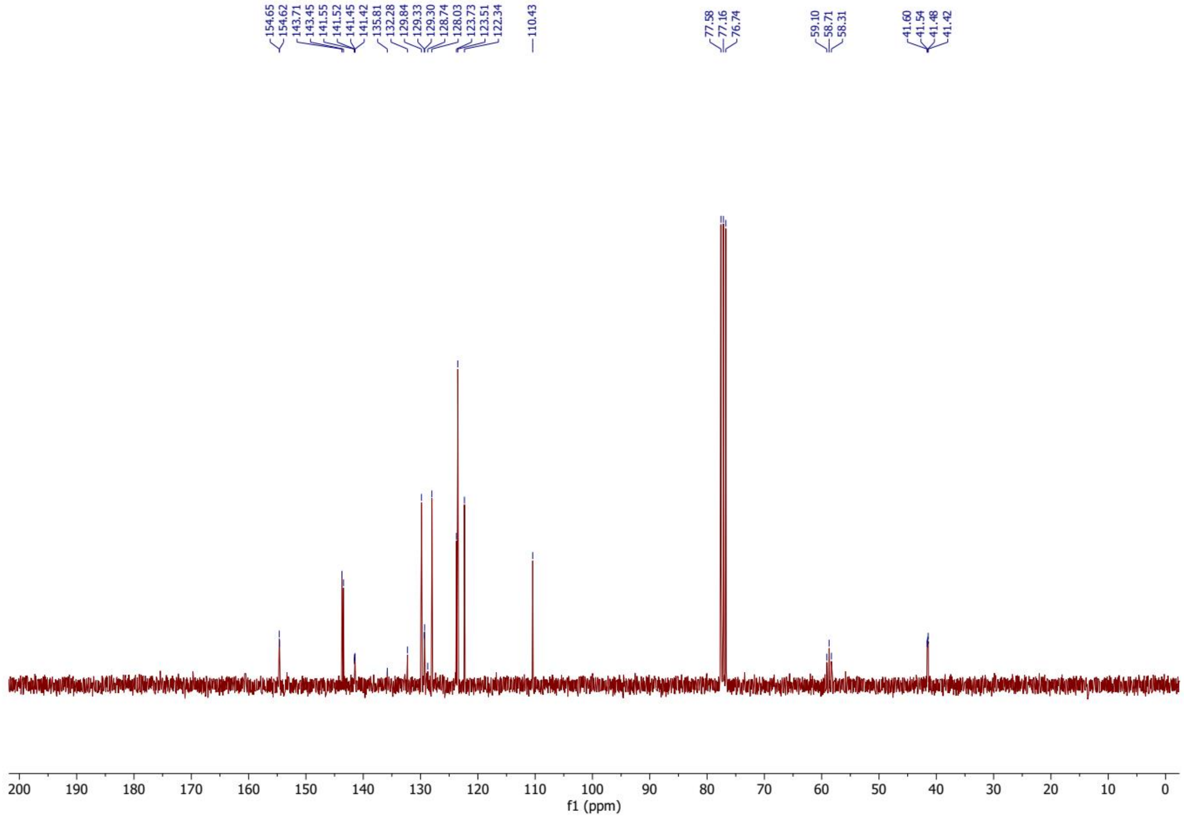
154.65
154.62
143.71
143.45
141.55
141.52
141.45
141.42
135.81
132.28
129.84
129.33
129.30
128.74
128.03
123.73
123.51
122.34
110.43
77.58
77.16
76.74
59.10
58.71
58.31
41.60
41.54
41.48
41.42
f1 (ppm)

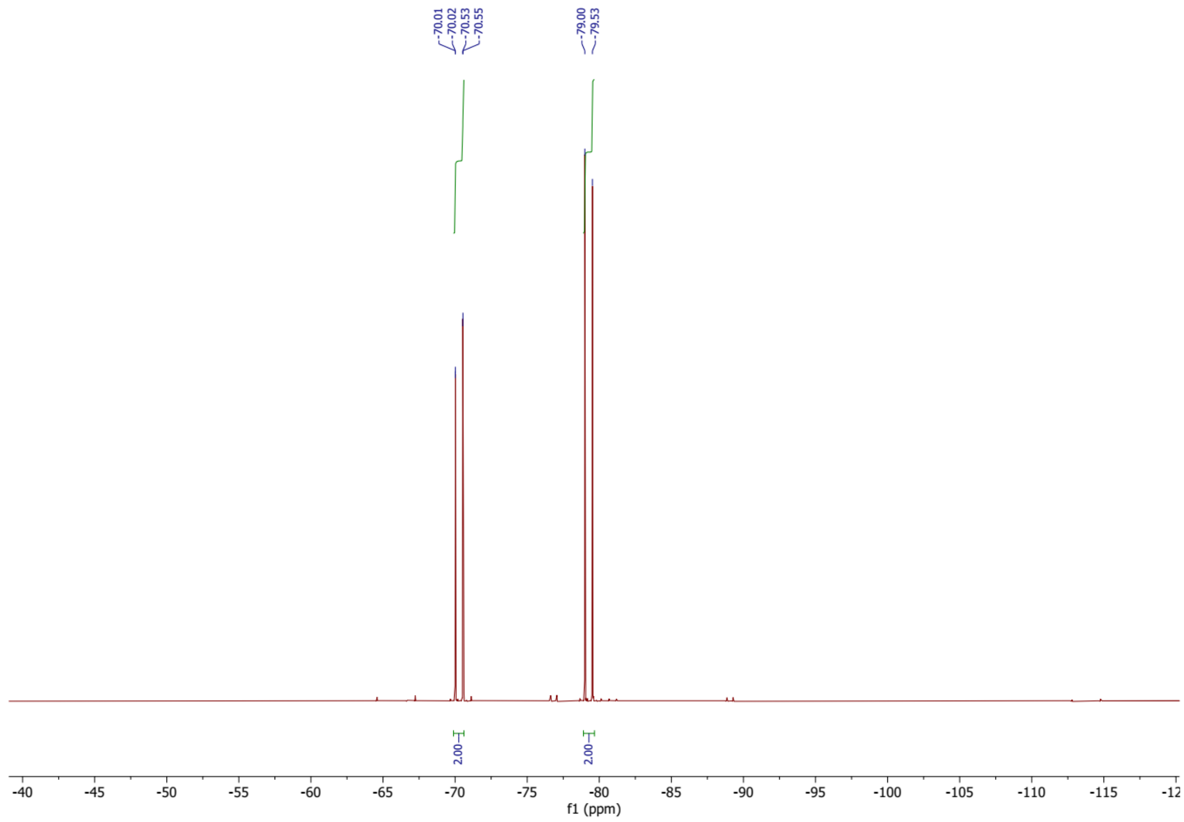
-70.01
-70.02
-70.53
-70.55
-79.00
-79.53
2.00
2.00
f1 (ppm)

4q

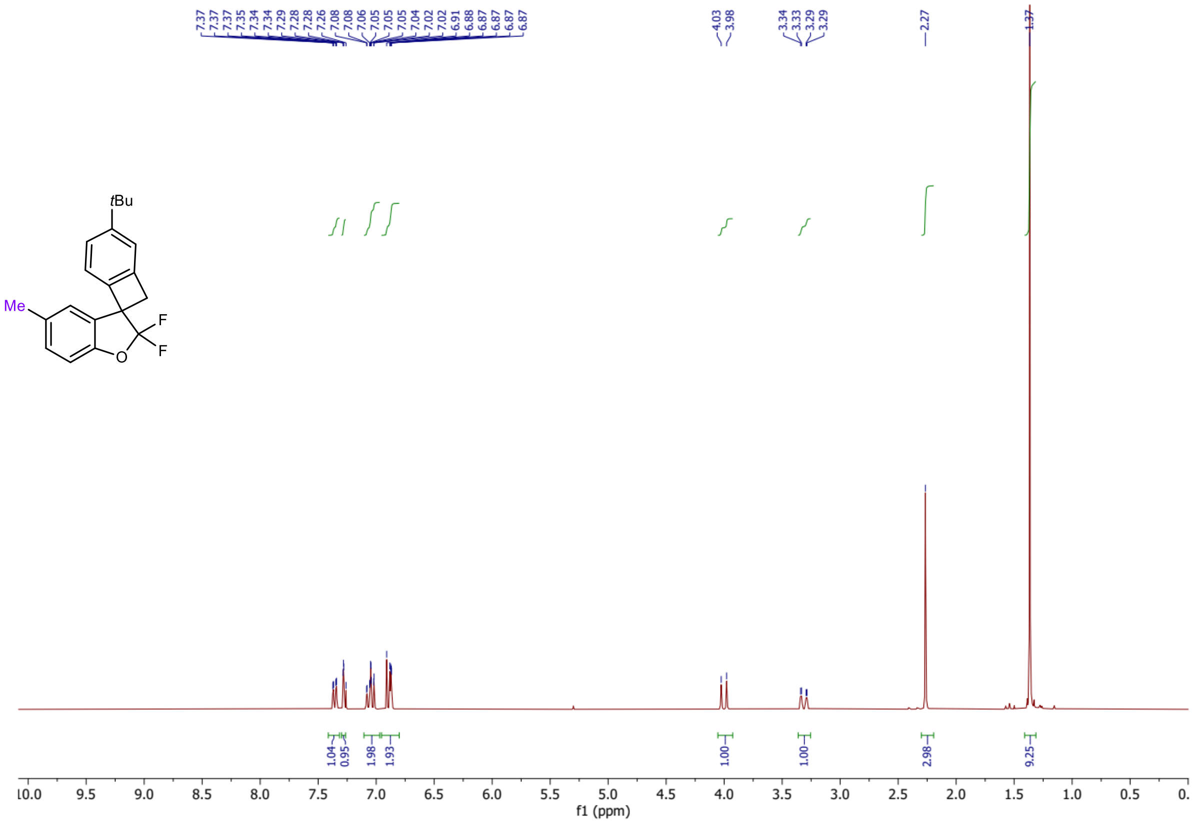
tBu
Me
F
F
O
7.37
7.37
7.35
7.34
7.34
7.29
7.28
7.28
7.26
7.08
7.08
7.06
7.05
7.05
7.05
7.04
7.02
7.02
6.91
6.88
6.87
6.87
6.87
6.87
4.03
3.98
3.34
3.33
3.29
3.29
2.27
1.37
1.04
0.95
1.98
1.93
1.00
1.00
2.98
9.25
f1 (ppm)

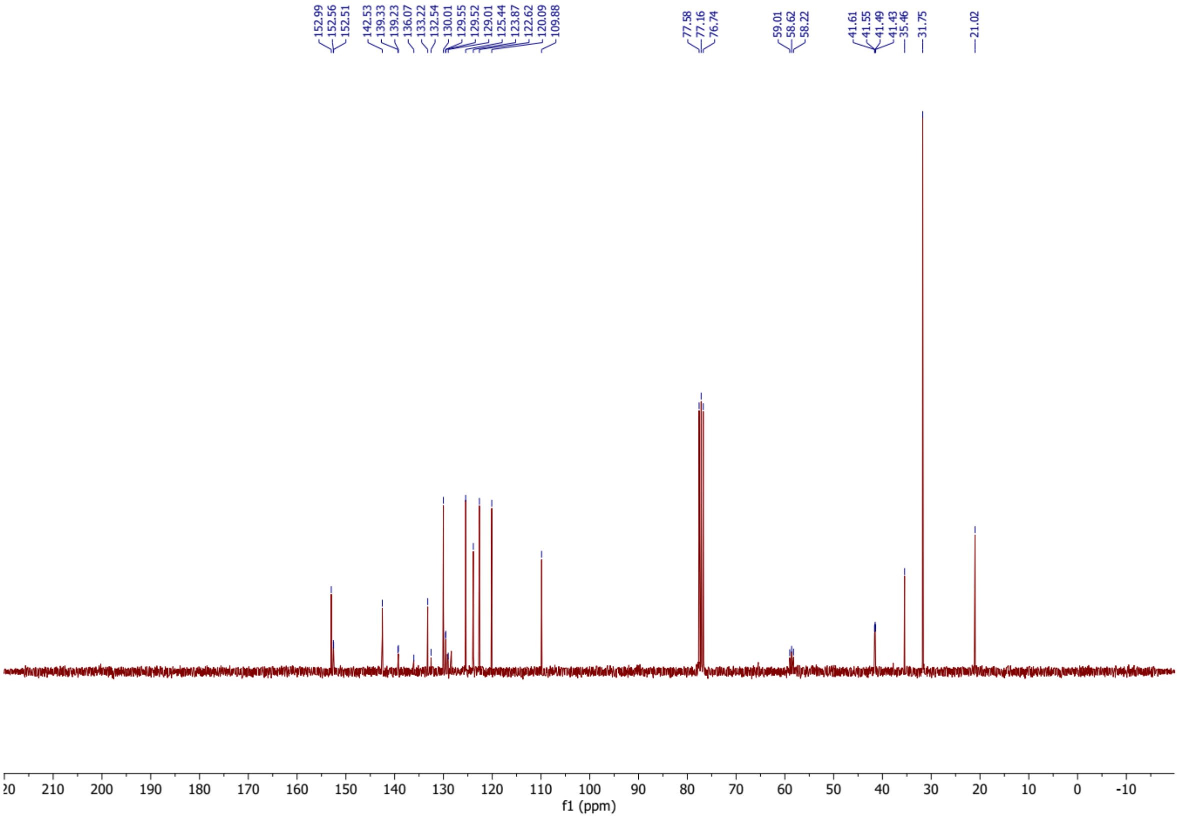
152.99
152.56
152.51
142.53
139.33
139.23
136.07
133.22
132.54
130.01
129.55
129.52
129.01
125.44
123.87
122.62
120.09
109.88
77.58
77.16
76.74
59.01
58.62
58.22
41.61
41.55
41.49
41.43
35.46
31.75
21.02
f1 (ppm)

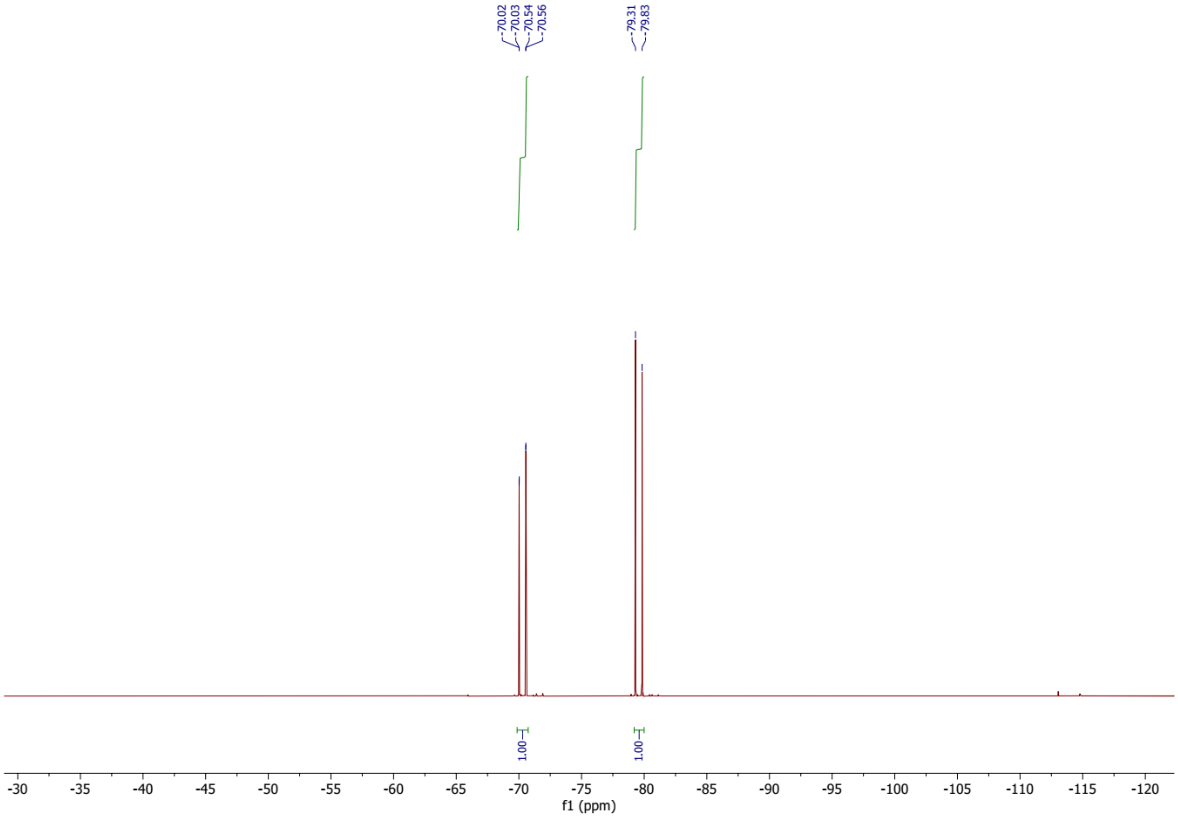

4r

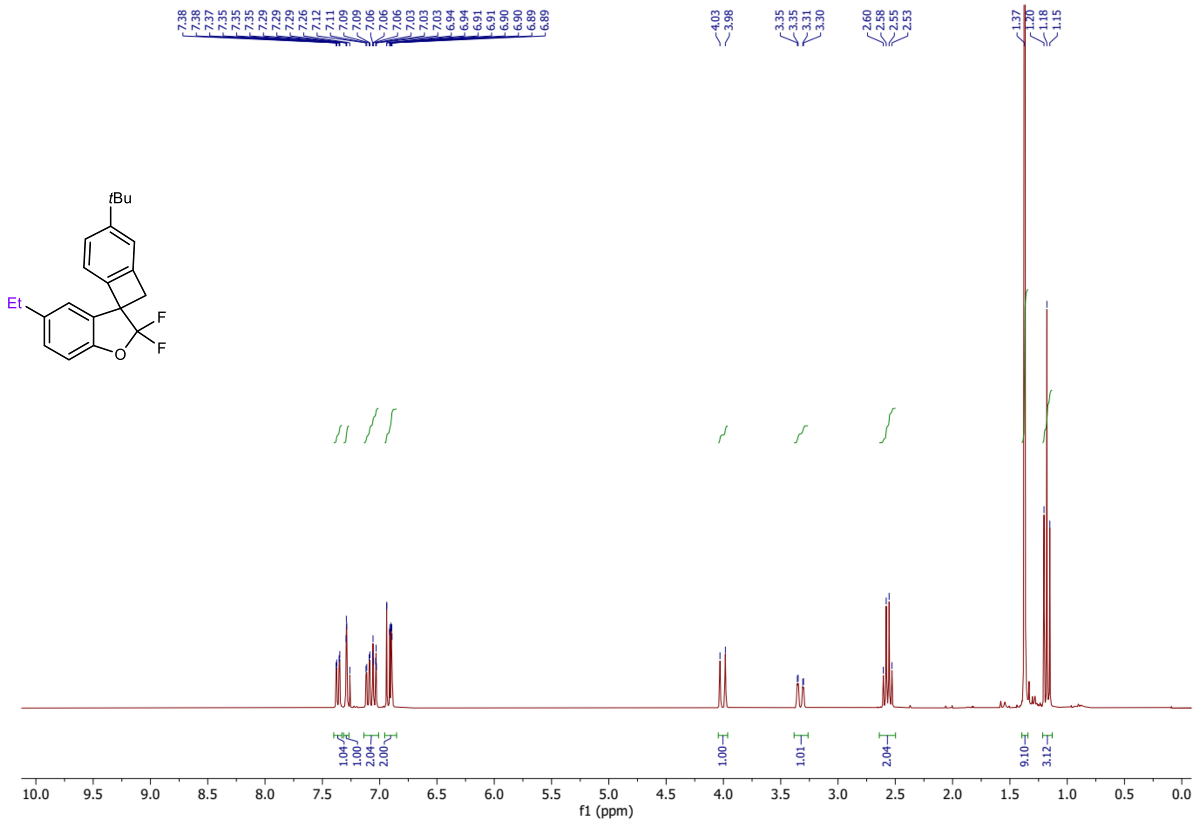

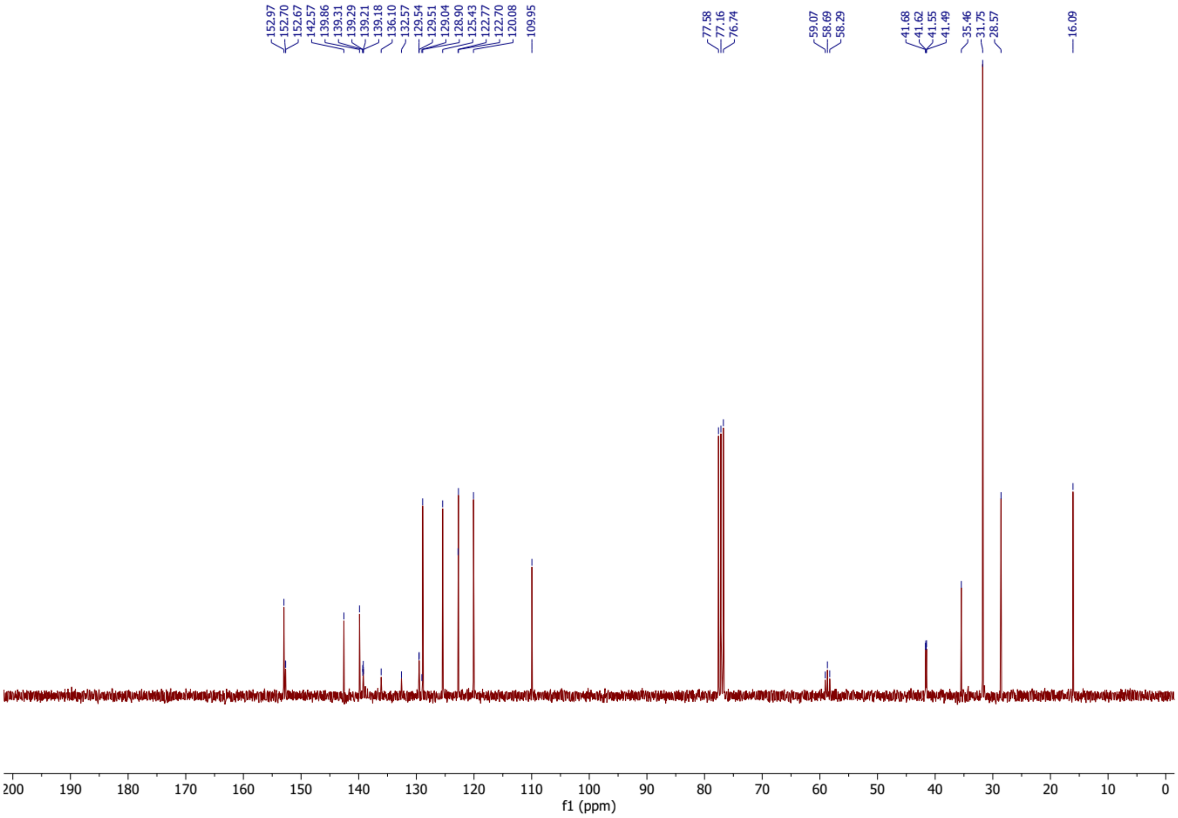
152.97
152.70
152.67
142.57
139.86
139.31
139.29
139.21
139.18
136.10
132.57
129.54
129.51
129.04
128.90
125.43
122.77
122.70
120.08
109.95
77.58
77.16
76.74
59.07
58.69
58.29
41.68
41.62
41.55
41.49
35.46
31.75
28.57
16.09
200 190 180 170 160 150 140 130 120 110 100 90 80 70 60 50 40 30 20 10 0
f1 (ppm)

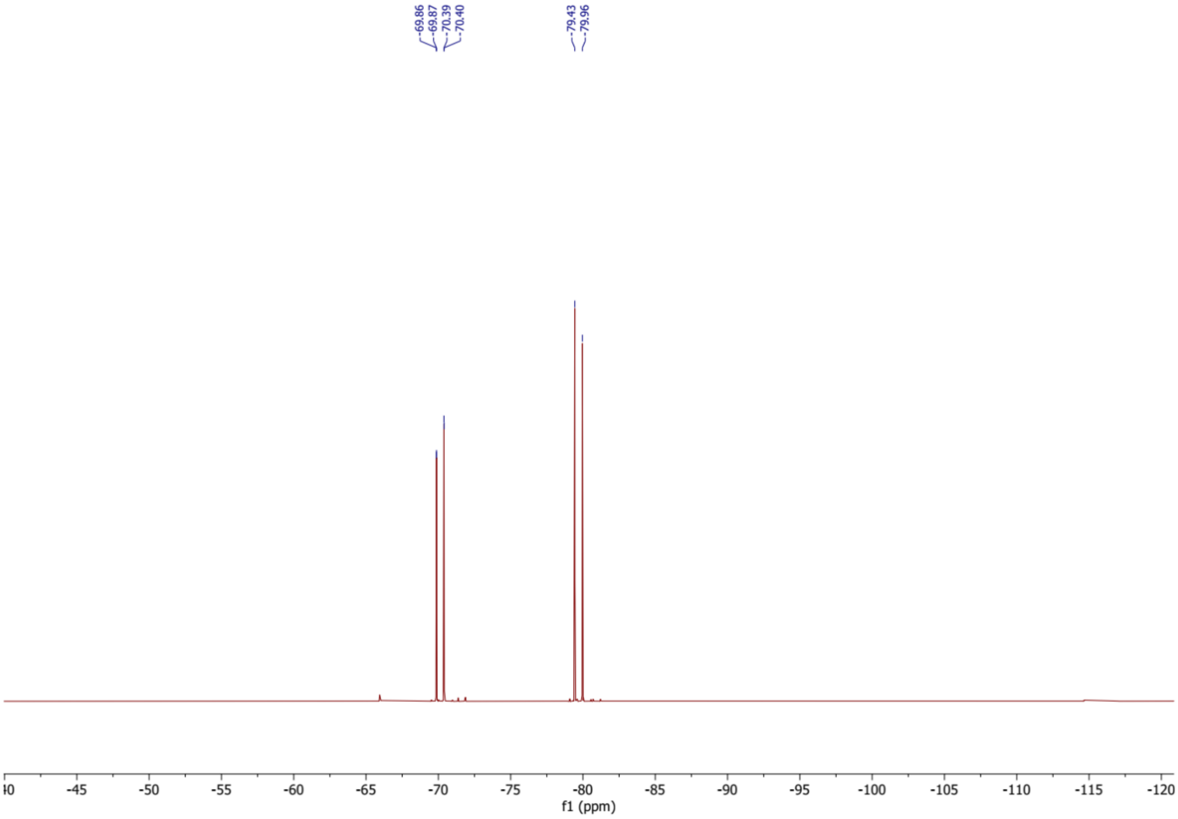
-69.86
-69.87
-70.39
-70.40
-79.43
-79.96
-40 -45 -50 -55 -60 -65 -70 -75 -80 -85 -90 -95 -100 -105 -110 -115 -120
f1 (ppm)

4s

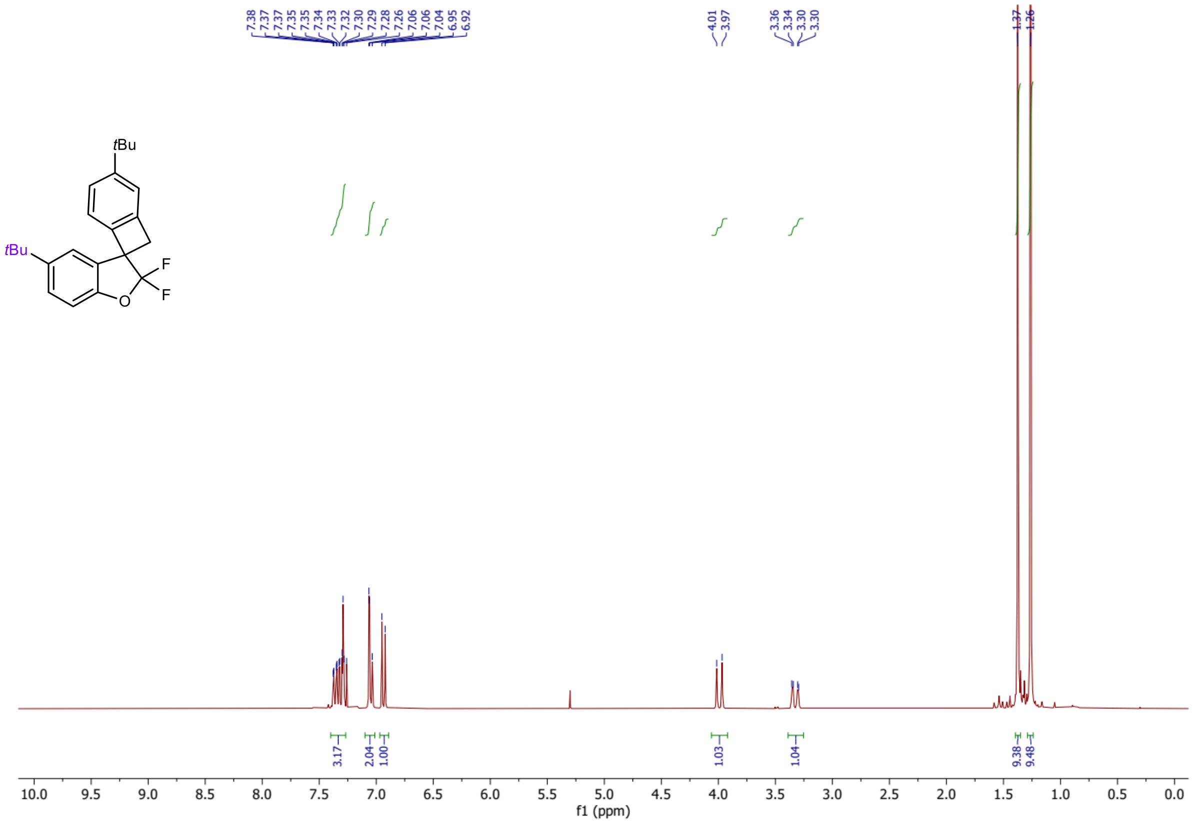
tBu
tBu
F
F
O
7.38
7.37
7.37
7.35
7.35
7.34
7.33
7.32
7.30
7.29
7.28
7.26
7.06
7.06
7.04
6.95
6.92
4.01
3.97
3.36
3.34
3.30
3.30
1.37
1.26
3.17
2.04
1.00
1.03
1.04
9.38
9.48
f1 (ppm)

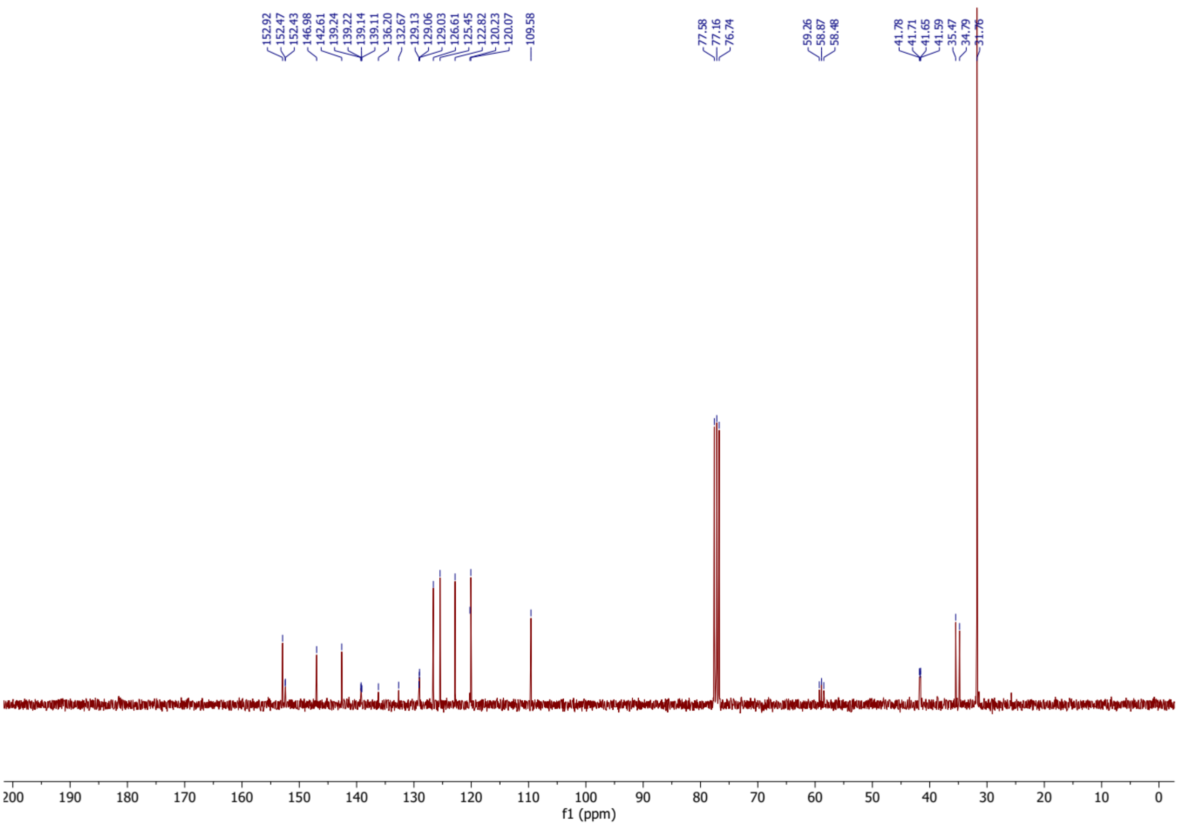
152.92
152.47
152.43
146.98
142.61
139.24
139.22
139.14
139.11
136.20
132.67
129.13
129.06
129.03
126.61
125.45
122.82
120.23
120.07
109.58
77.58
77.16
76.74
59.26
58.87
58.48
41.78
41.71
41.65
41.59
35.47
34.79
31.76
f1 (ppm)

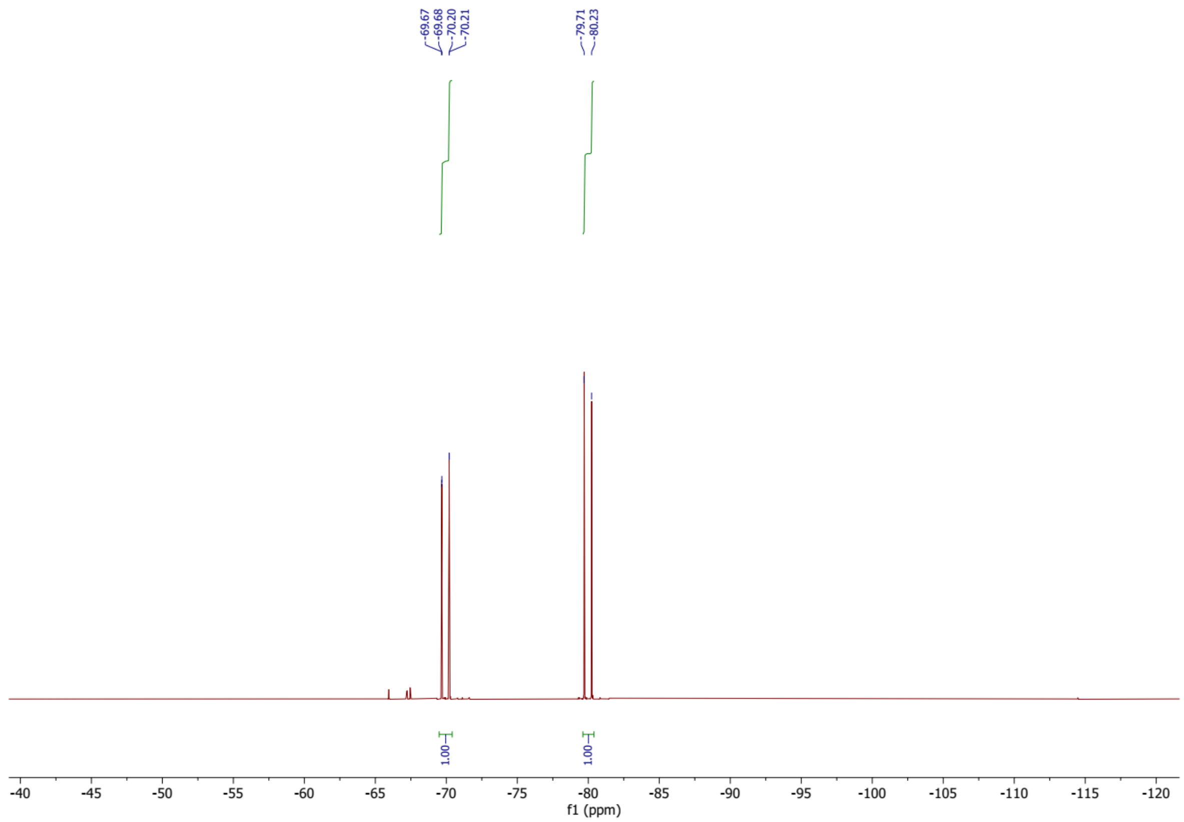

4t

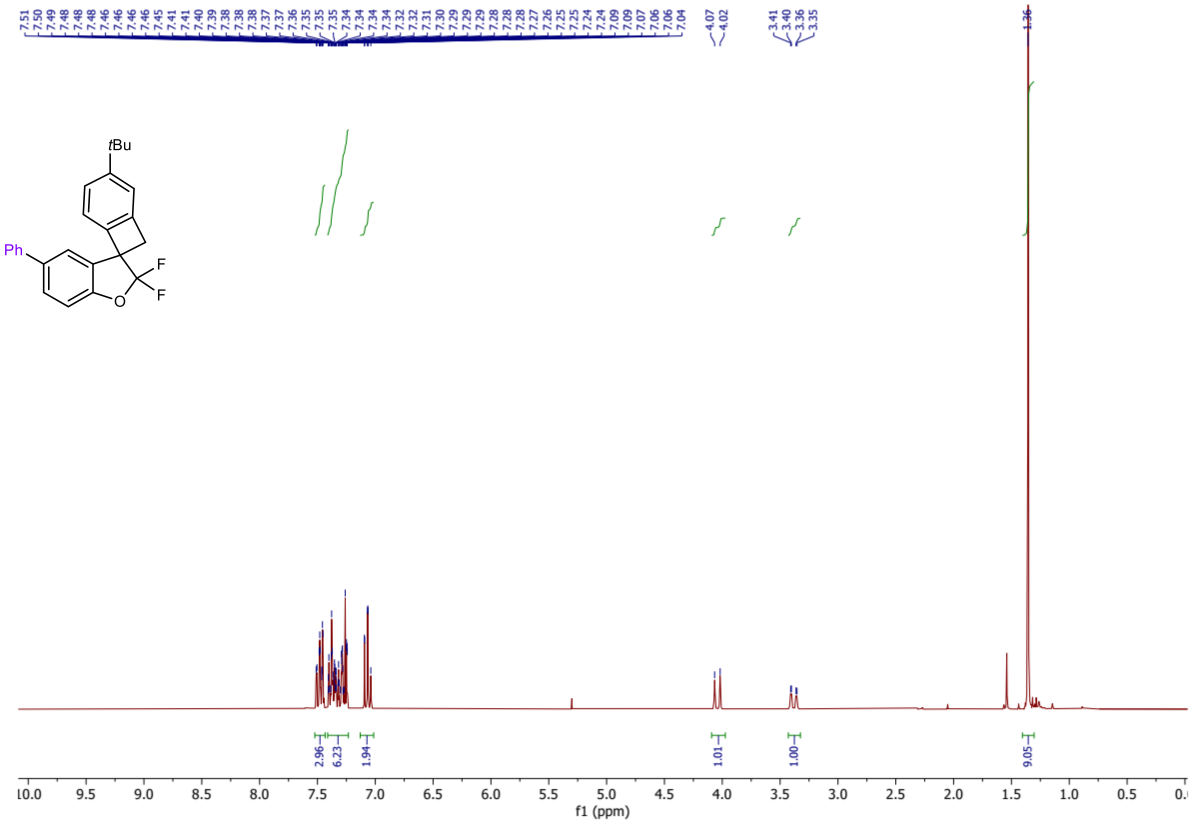

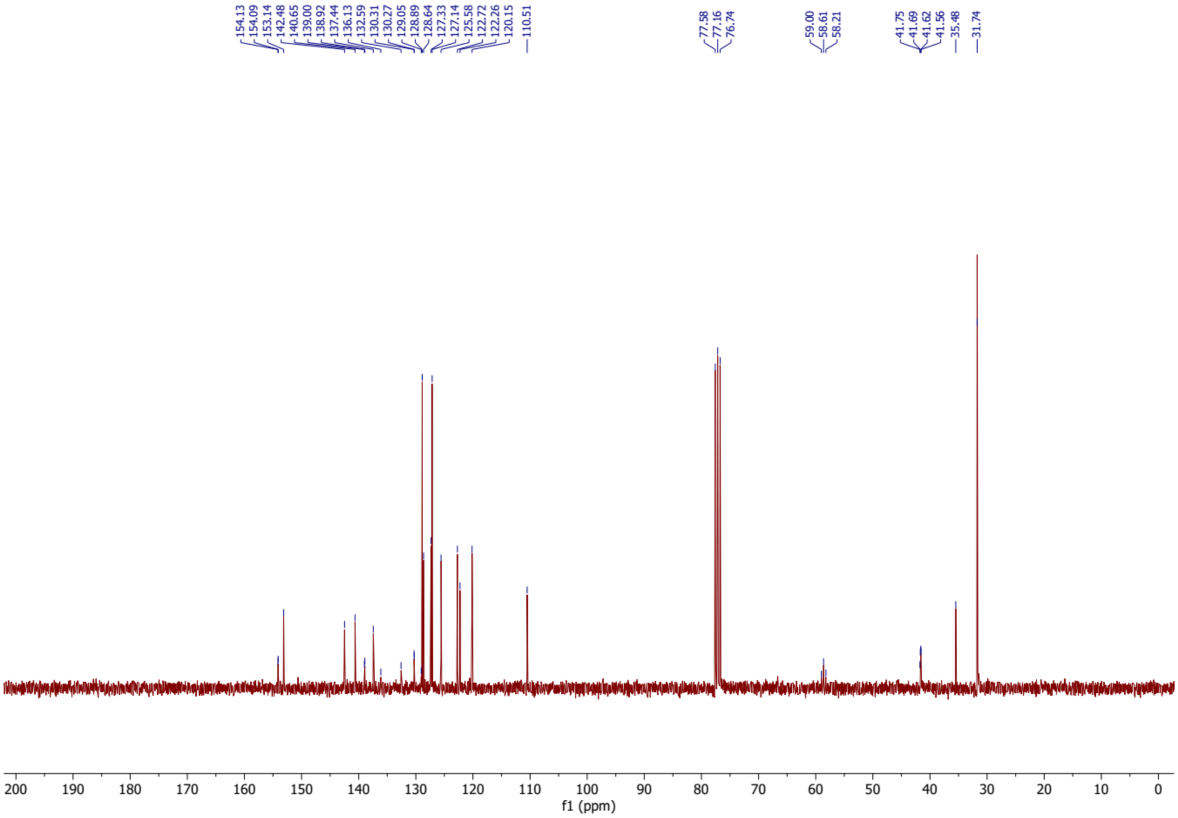
154.13
154.09
153.14
142.48
140.65
139.00
138.92
137.44
136.13
132.59
130.31
130.27
129.05
128.89
128.64
127.33
127.14
125.58
122.72
122.26
120.15
110.51
77.58
77.16
76.74
59.00
58.61
58.21
41.75
41.69
41.62
41.56
35.48
31.74
200 190 180 170 160 150 140 130 120 110 100 90 80 70 60 50 40 30 20 10 0
f1 (ppm)

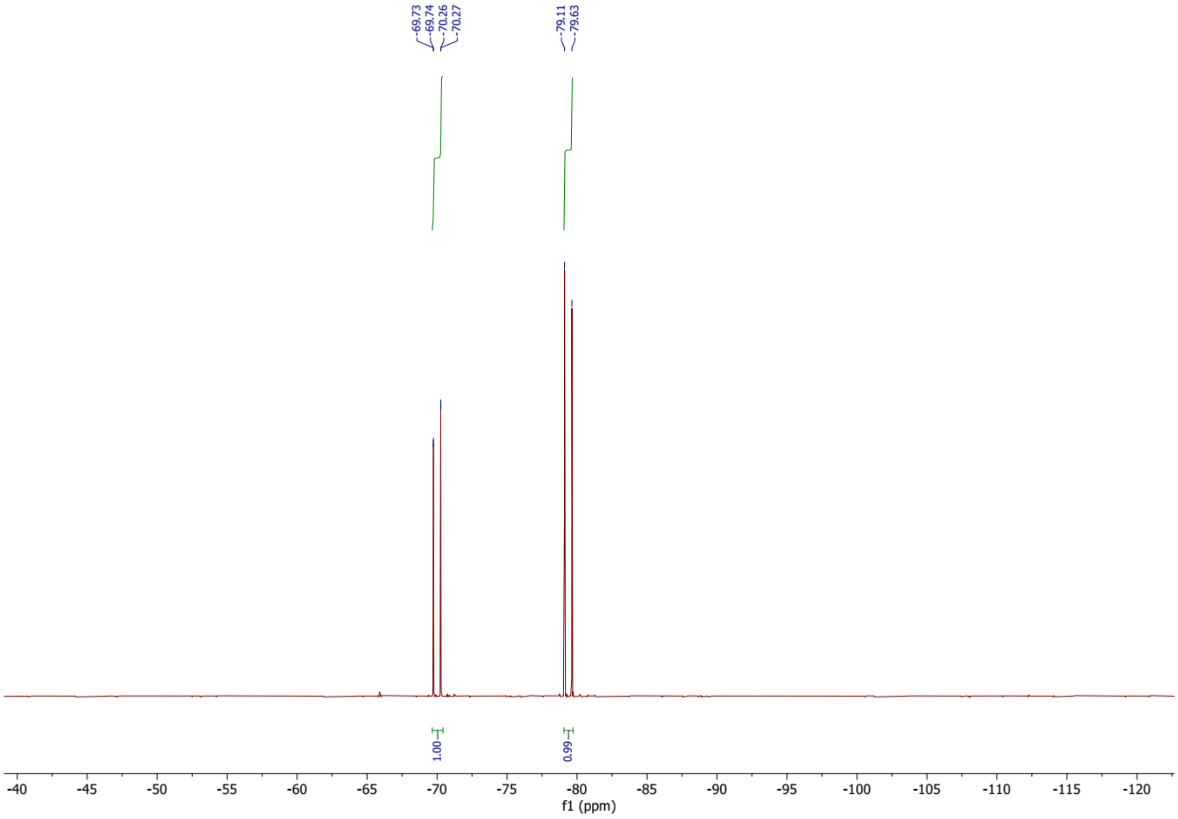
-69.73
-69.74
-70.26
-70.27
-79.11
-79.63
1.00
0.99
-40 -45 -50 -55 -60 -65 -70 -75 -80 -85 -90 -95 -100 -105 -110 -115 -120
f1 (ppm)

4u

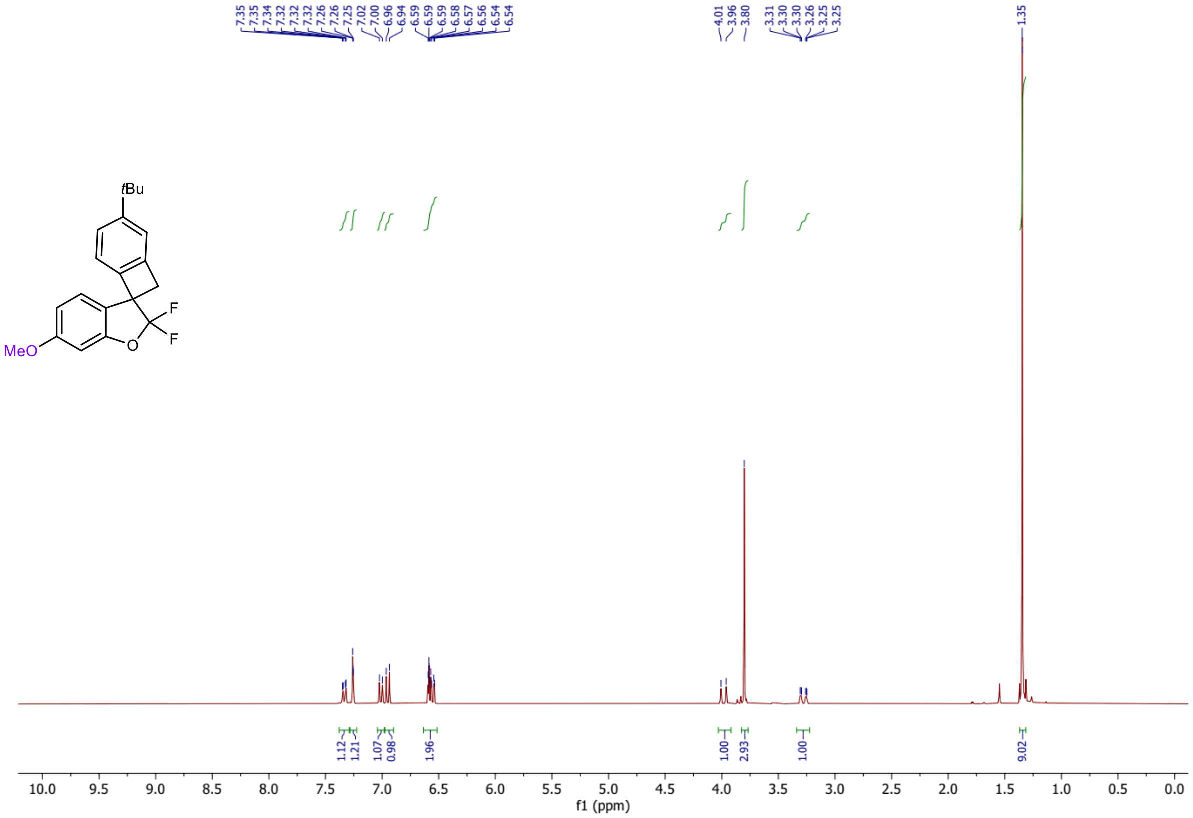
tBu
F
F
O
MeO
7.35
7.35
7.34
7.32
7.32
7.32
7.26
7.25
7.02
7.00
6.96
6.94
6.59
6.59
6.59
6.58
6.57
6.56
6.54
6.54
4.01
3.96
3.80
3.31
3.30
3.30
3.26
3.25
3.25
1.35
1.12
1.21
1.07
0.98
1.96
1.00
2.93
1.00
9.02
10.0
9.5
9.0
8.5
8.0
7.5
7.0
6.5
6.0
5.5
5.0
4.5
4.0
3.5
3.0
2.5
2.0
1.5
1.0
0.5
0.0
f1 (ppm)

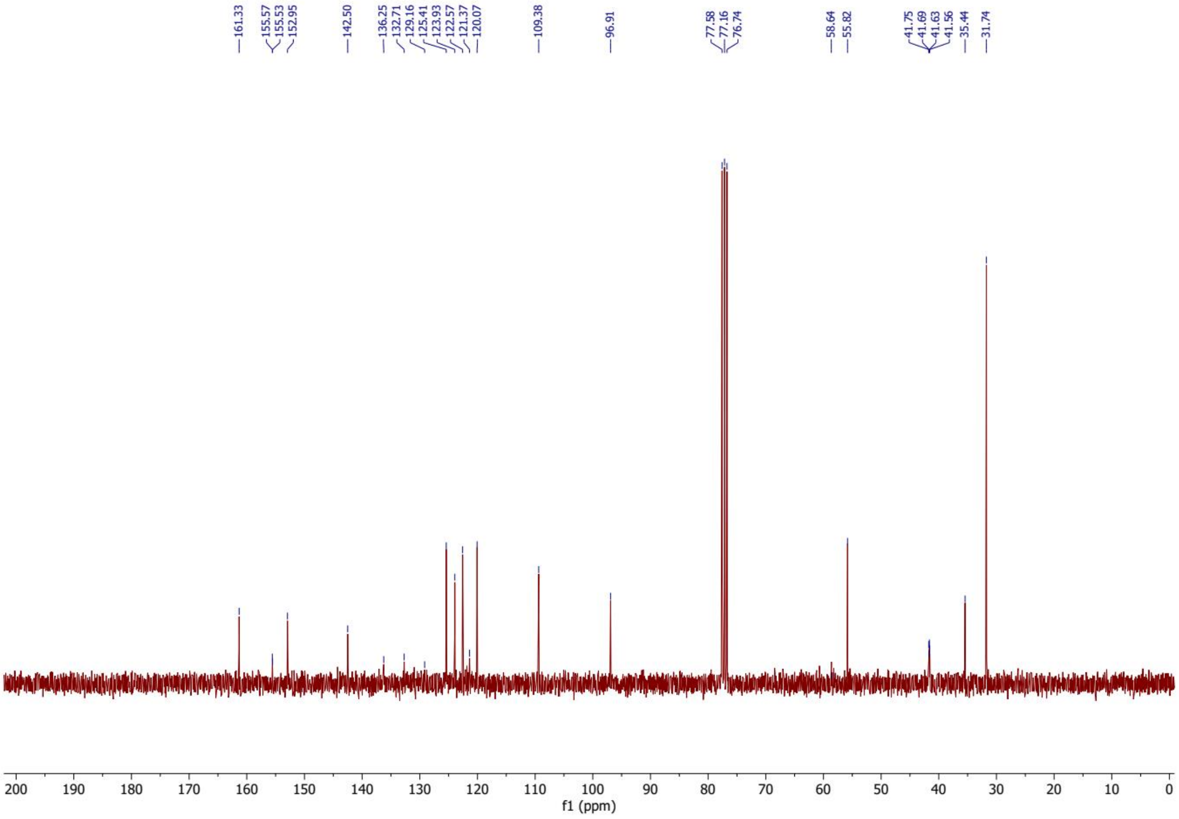
161.33
155.57
155.53
152.95
142.50
136.25
132.71
129.16
125.41
123.93
122.57
121.37
120.07
109.38
96.91
77.58
77.16
76.74
58.64
55.82
41.75
41.69
41.63
41.56
35.44
31.74
200
190
180
170
160
150
140
130
120
110
100
90
80
70
60
50
40
30
20
10
0
f1 (ppm)

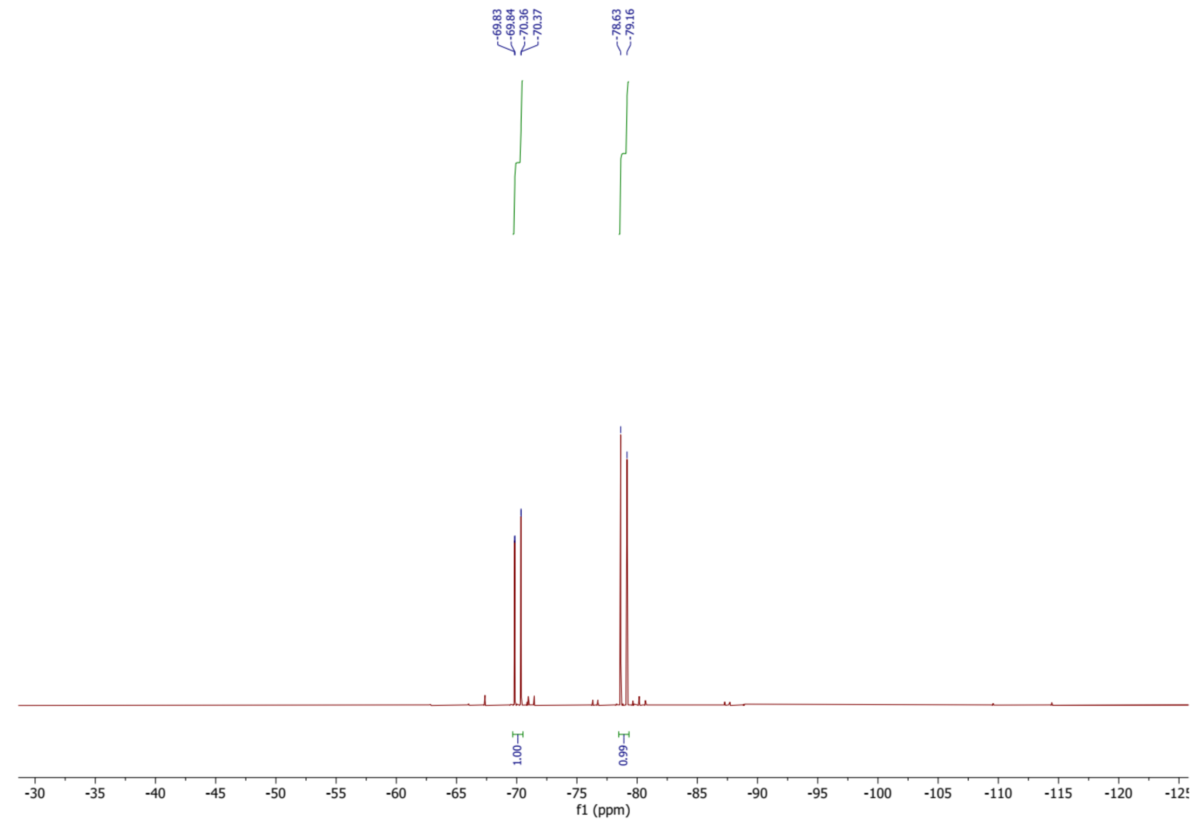
-69.83
-69.84
-70.36
-70.37
-78.63
-79.16
1.00
0.99
-30 -35 -40 -45 -50 -55 -60 -65 -70 -75 -80 -85 -90 -95 -100 -105 -110 -115 -120
f1 (ppm)

4v

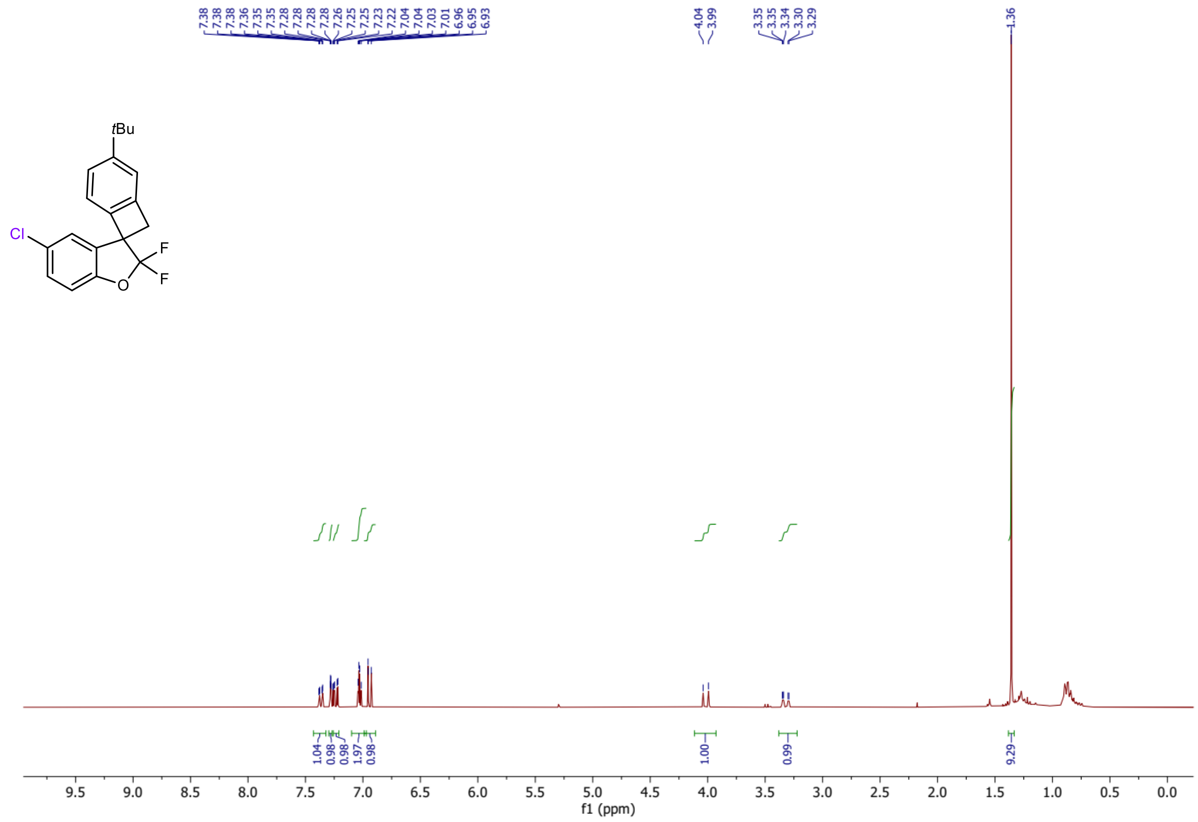
7.38
7.38
7.38
7.36
7.35
7.35
7.28
7.28
7.28
7.26
7.25
7.25
7.23
7.22
7.04
7.04
7.03
7.01
6.96
6.93
4.04
3.99
3.35
3.35
3.34
3.30
3.29
1.36
tBu
Cl
F
F
O
1.04
0.98
0.98
1.97
0.98
1.00
0.99
9.29
9.5 9.0 8.5 8.0 7.5 7.0 6.5 6.0 5.5 5.0 4.5 4.0 3.5 3.0 2.5 2.0 1.5 1.0 0.5 0.0
f1 (ppm)

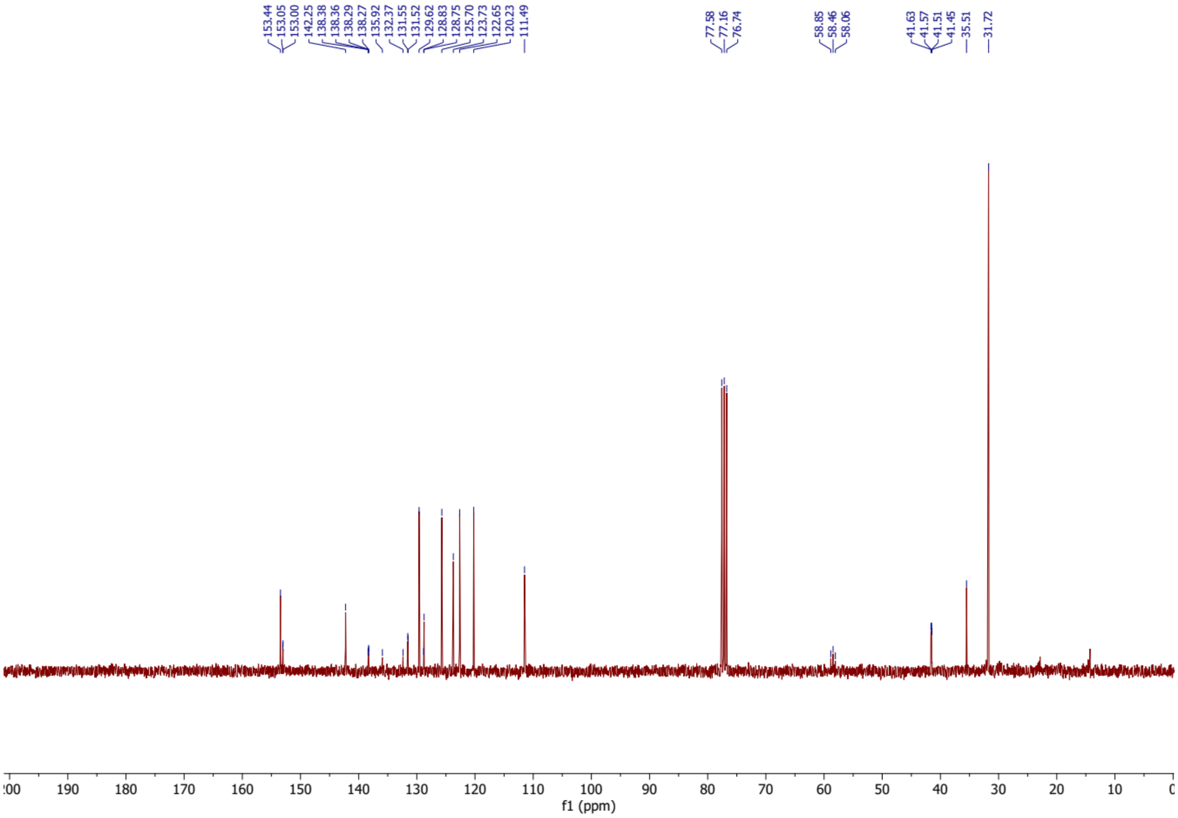
153.44
153.05
153.00
142.25
138.38
138.36
138.29
138.27
135.92
132.37
131.55
131.52
129.62
128.83
128.75
125.70
123.73
122.65
120.23
111.49
77.58
77.16
76.74
58.85
58.46
58.06
41.63
41.57
41.51
41.45
35.51
31.72
f1 (ppm)

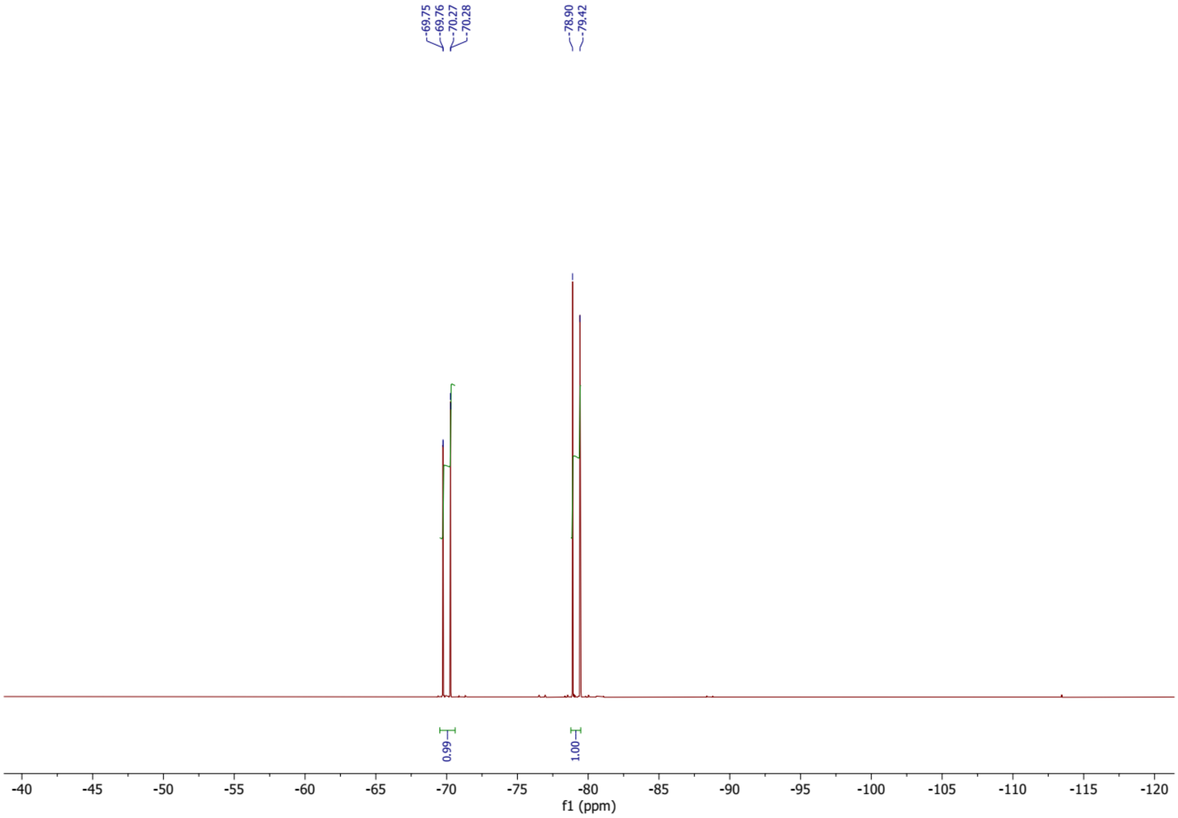
-69.75
-69.76
-70.27
-70.28
-78.90
-79.42
0.99
1.00
f1 (ppm)

4w

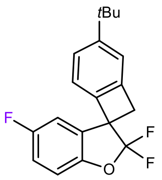

tBu
F
F
F
O

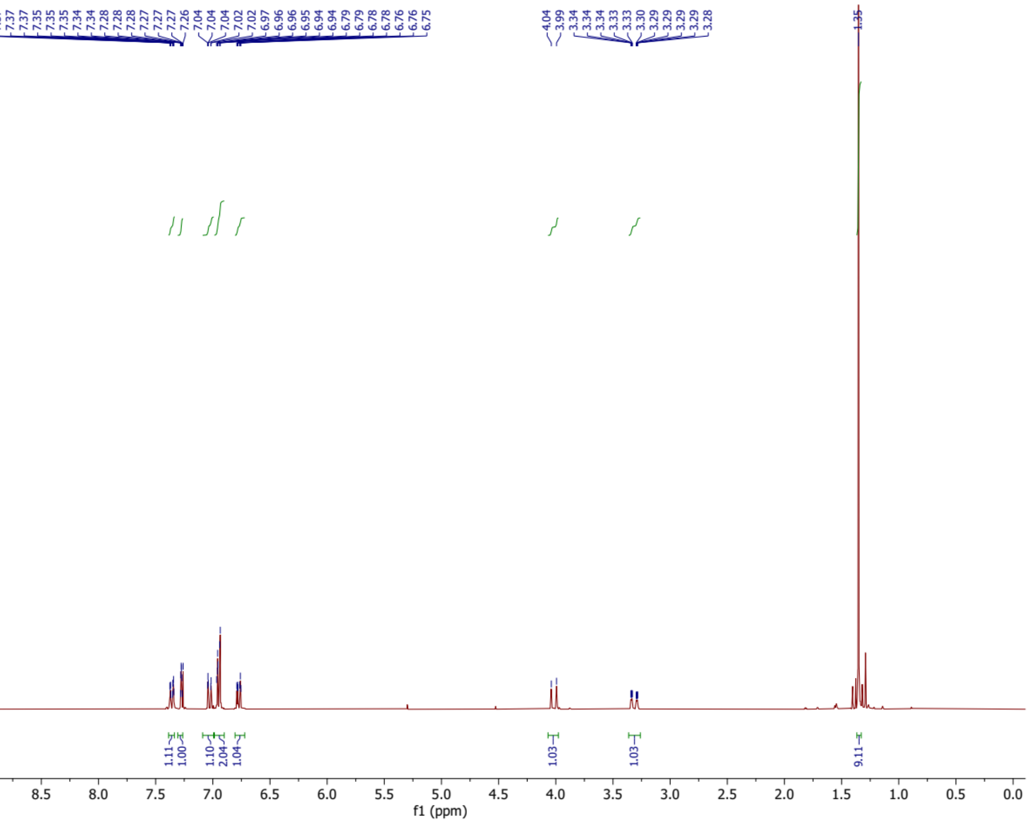

7.38 7.37 7.37 7.35 7.35 7.35 7.34 7.34 7.28 7.28 7.28 7.27 7.27 7.26 7.04 7.04 7.04 7.02 7.02 6.97 6.96 6.96 6.95 6.94 6.94 6.79 6.79 6.78 6.76 6.76 6.75
4.04 3.99 3.34 3.34 3.33 3.33 3.30 3.29 3.29 3.29 3.28
1.35
1.11 1.00 1.10 2.04 1.04 1.03 1.03 9.11
10.0 9.5 9.0 8.5 8.0 7.5 7.0 6.5 6.0 5.5 5.0 4.5 4.0 3.5 3.0 2.5 2.0 1.5 1.0 0.5 0.0
f1 (ppm)

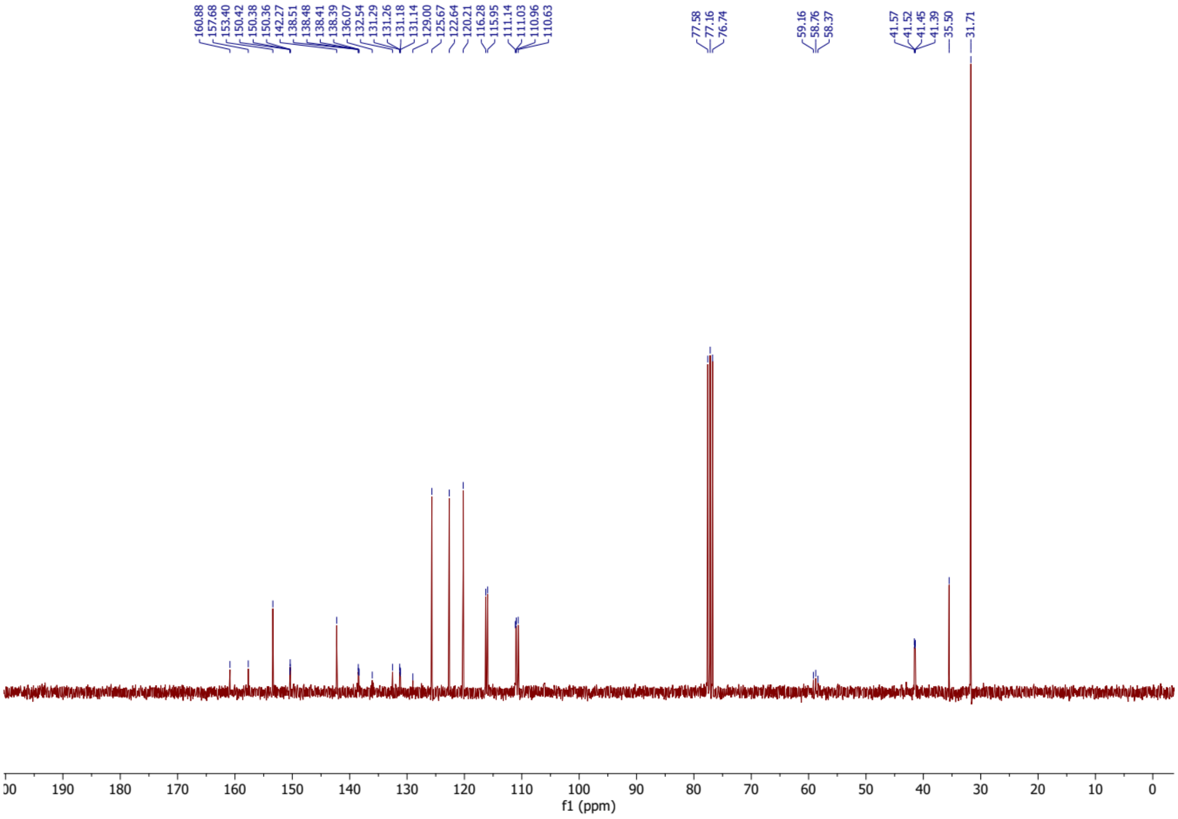

160.88 157.68 153.40 150.42 150.38 150.36 142.27 138.51 138.48 138.41 138.39 136.07 132.54 131.29 131.26 131.18 131.14 129.00 125.67 122.64 120.21 116.28 115.95 111.14 111.03 110.96 110.63
77.58 77.16 76.74
59.16 58.76 58.37
41.57 41.52 41.45 41.39 35.50 31.71
190 180 170 160 150 140 130 120 110 100 90 80 70 60 50 40 30 20 10 0
f1 (ppm)

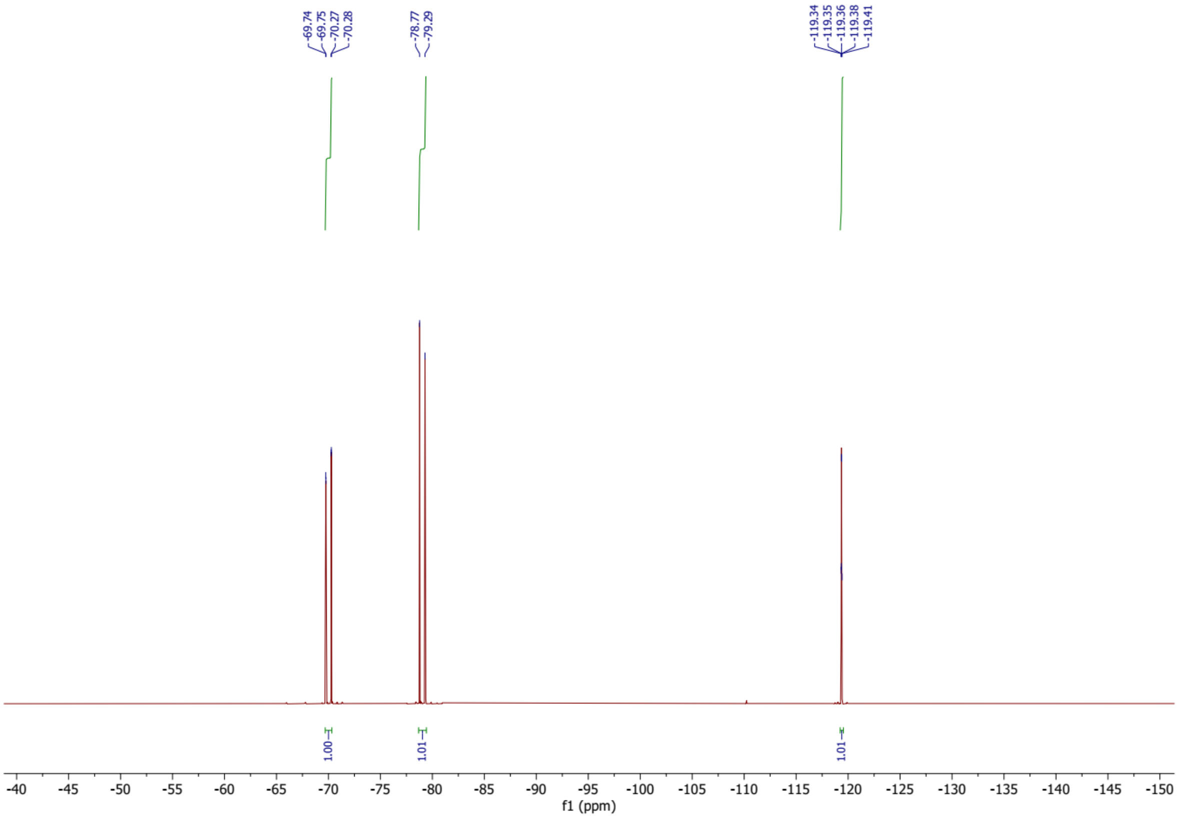
-69.74
-69.75
-70.27
-70.28
-78.77
-79.29
-119.34
-119.35
-119.36
-119.38
-119.41
1.00
1.01
1.01
-40 -45 -50 -55 -60 -65 -70 -75 -80 -85 -90 -95 -100 -105 -110 -115 -120 -125 -130 -135 -140 -145 -150
f1 (ppm)

4x

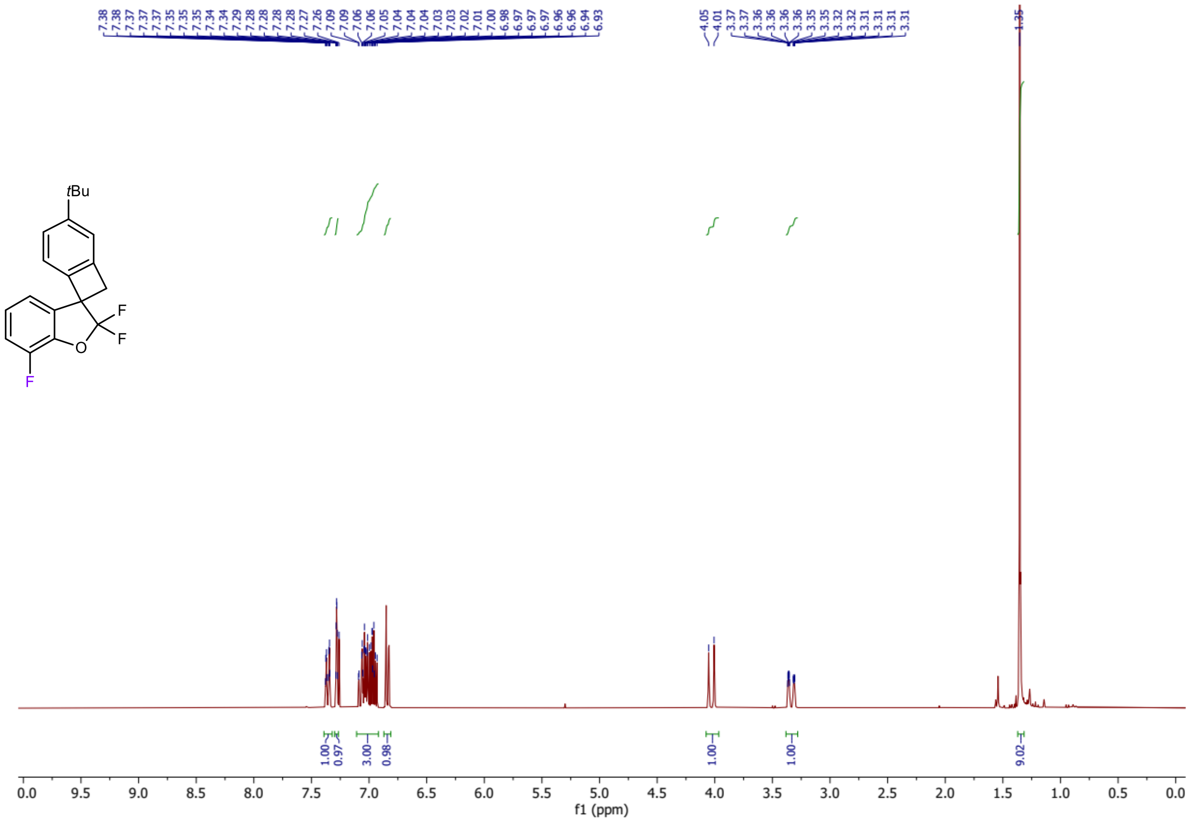
7.38
7.38
7.37
7.37
7.37
7.35
7.35
7.35
7.34
7.34
7.29
7.28
7.28
7.28
7.27
7.26
7.09
7.09
7.06
7.06
7.05
7.04
7.04
7.04
7.03
7.03
7.02
7.01
7.00
6.98
6.97
6.97
6.97
6.96
6.94
6.93
4.05
4.01
3.37
3.36
3.36
3.36
3.36
3.35
3.35
3.32
3.32
3.31
3.31
3.31
3.31
1.35
tBu
F
F
O
F
1.00
0.97
3.00
0.98
1.00
1.00
9.02
0.0 9.5 9.0 8.5 8.0 7.5 7.0 6.5 6.0 5.5 5.0 4.5 4.0 3.5 3.0 2.5 2.0 1.5 1.0 0.5 0.0
f1 (ppm)

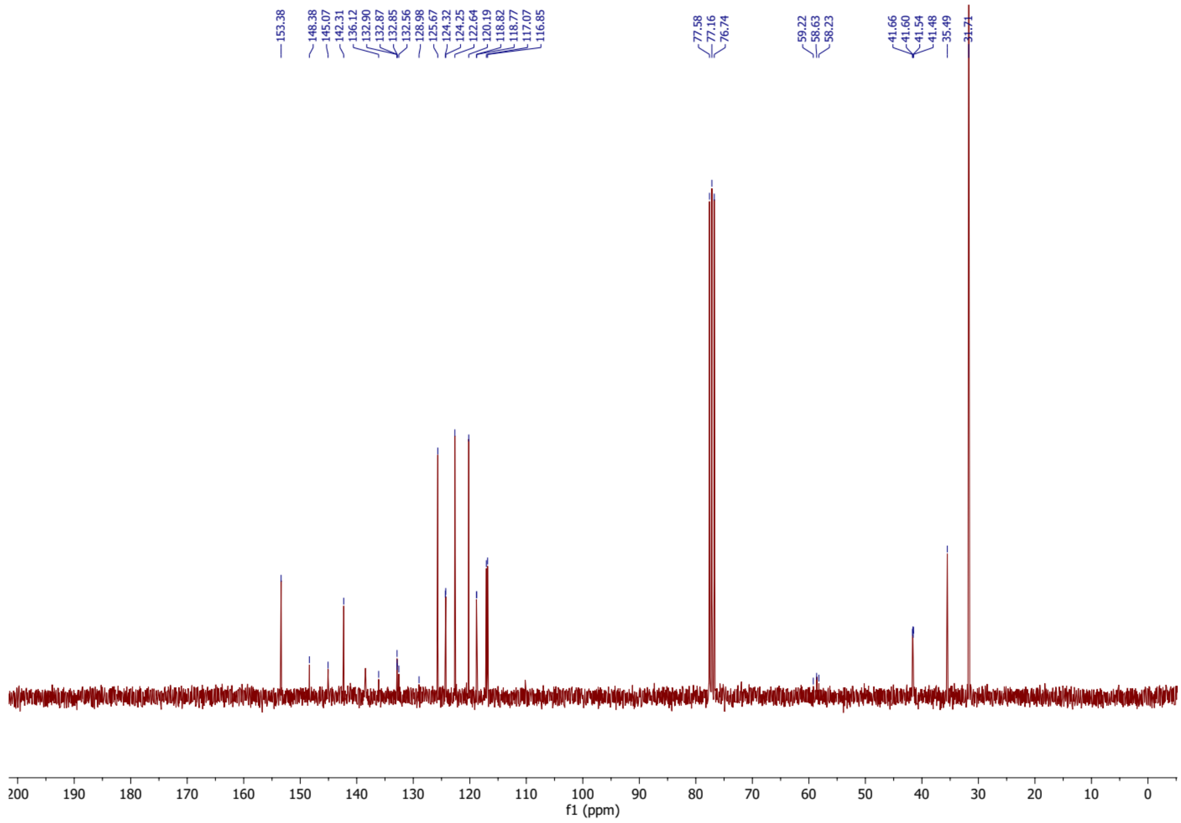
153.38
148.38
145.07
142.31
136.12
132.90
132.87
132.85
132.56
128.98
125.67
124.32
124.25
122.64
120.19
118.82
118.77
117.07
116.85
77.58
77.16
76.74
59.22
58.63
58.23
41.66
41.60
41.54
41.48
35.49
31.71
f1 (ppm)

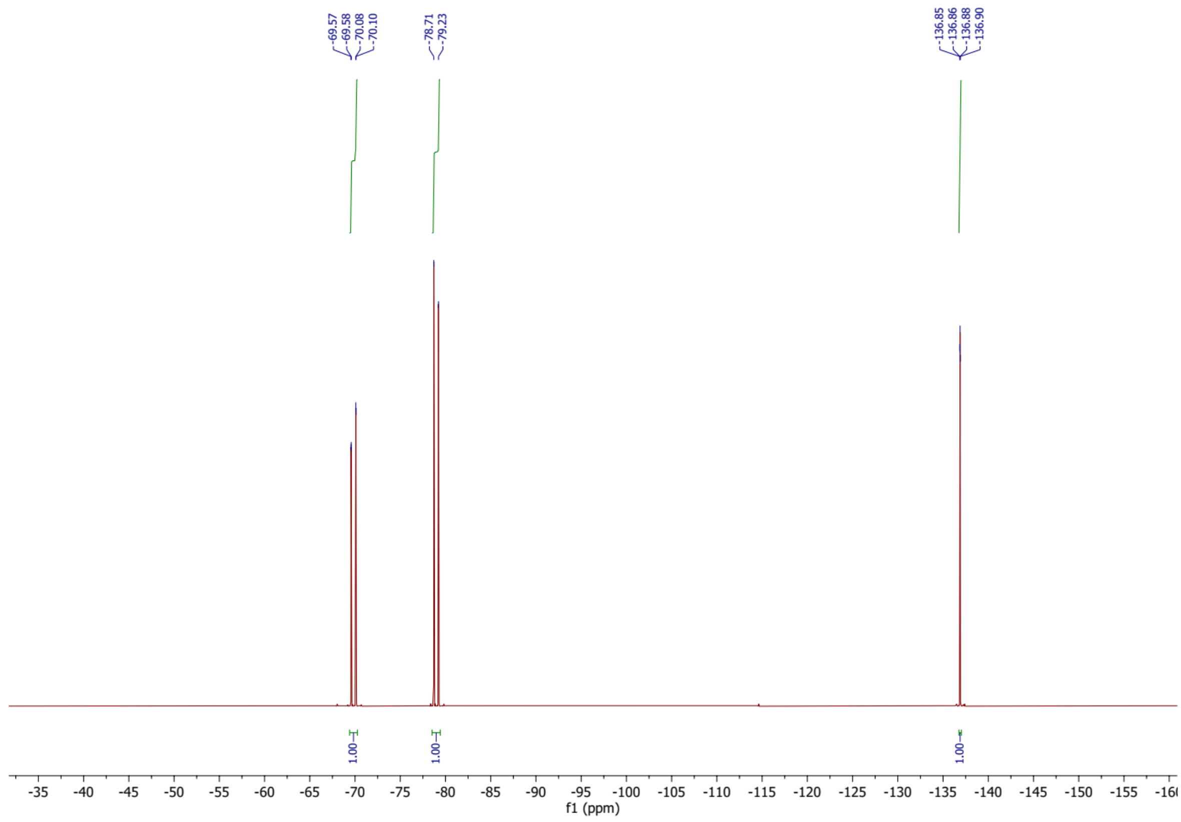
-69.57
-69.58
-70.08
-70.10
-78.71
-79.23
-136.85
-136.86
-136.88
-136.90
1.00
1.00
1.00
f1 (ppm)

4y

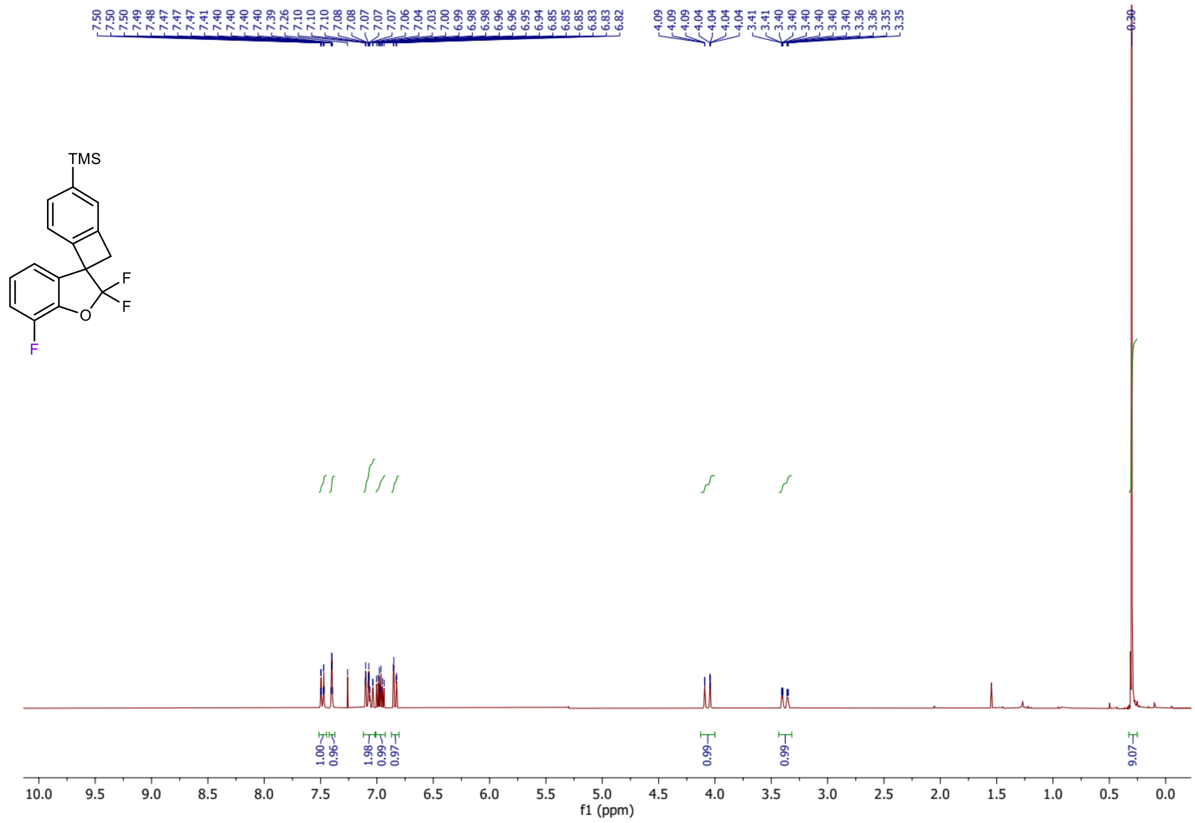

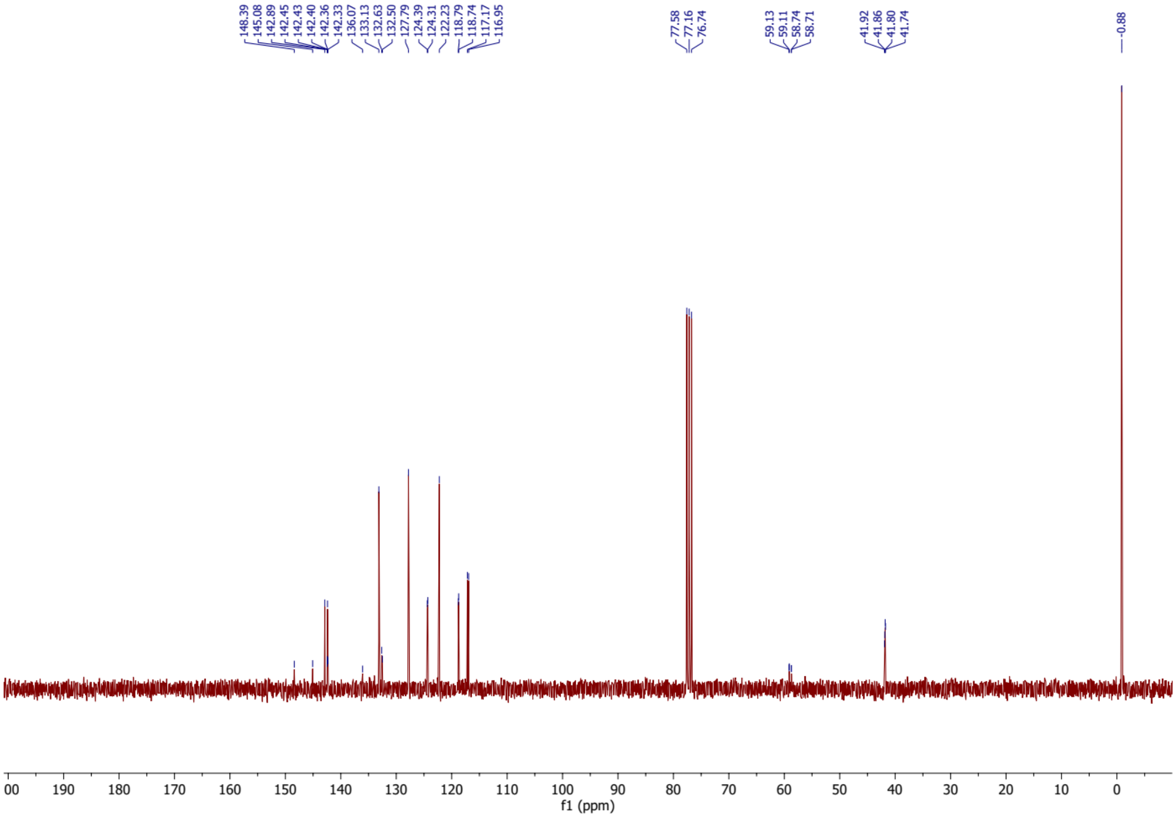

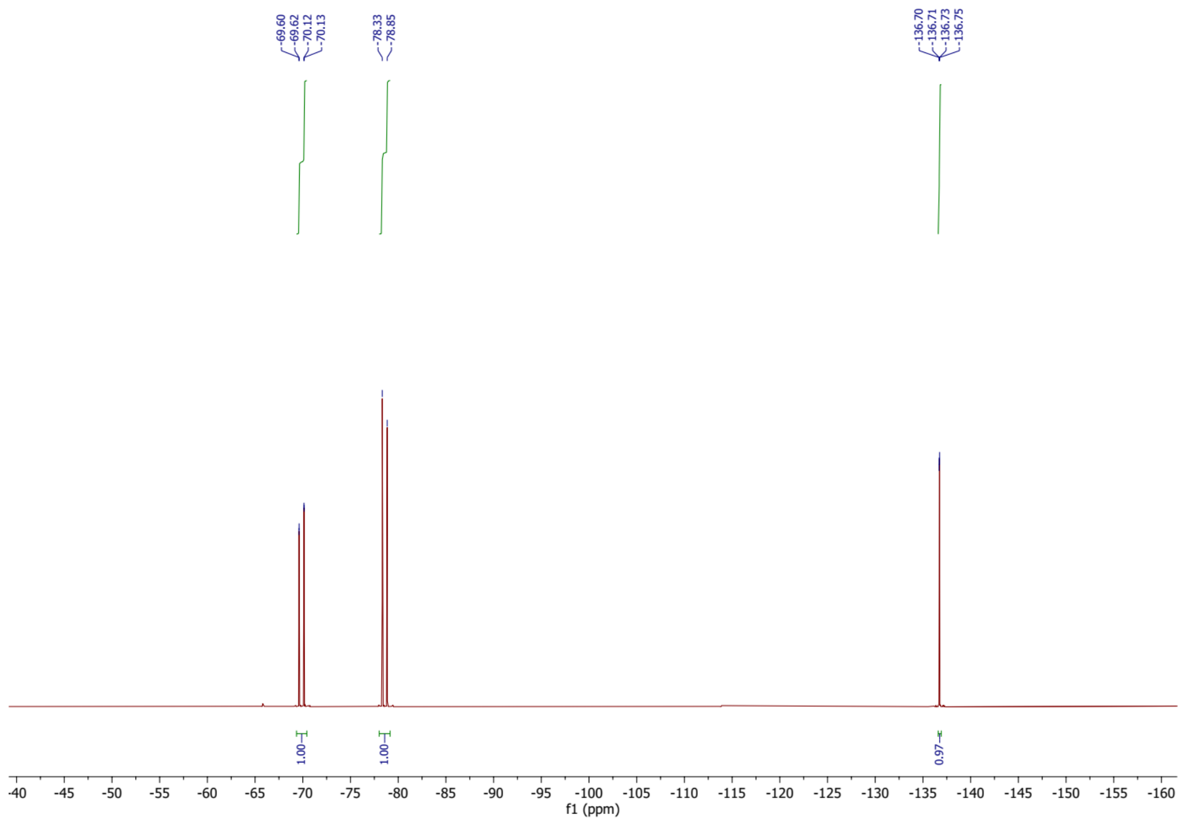

4z

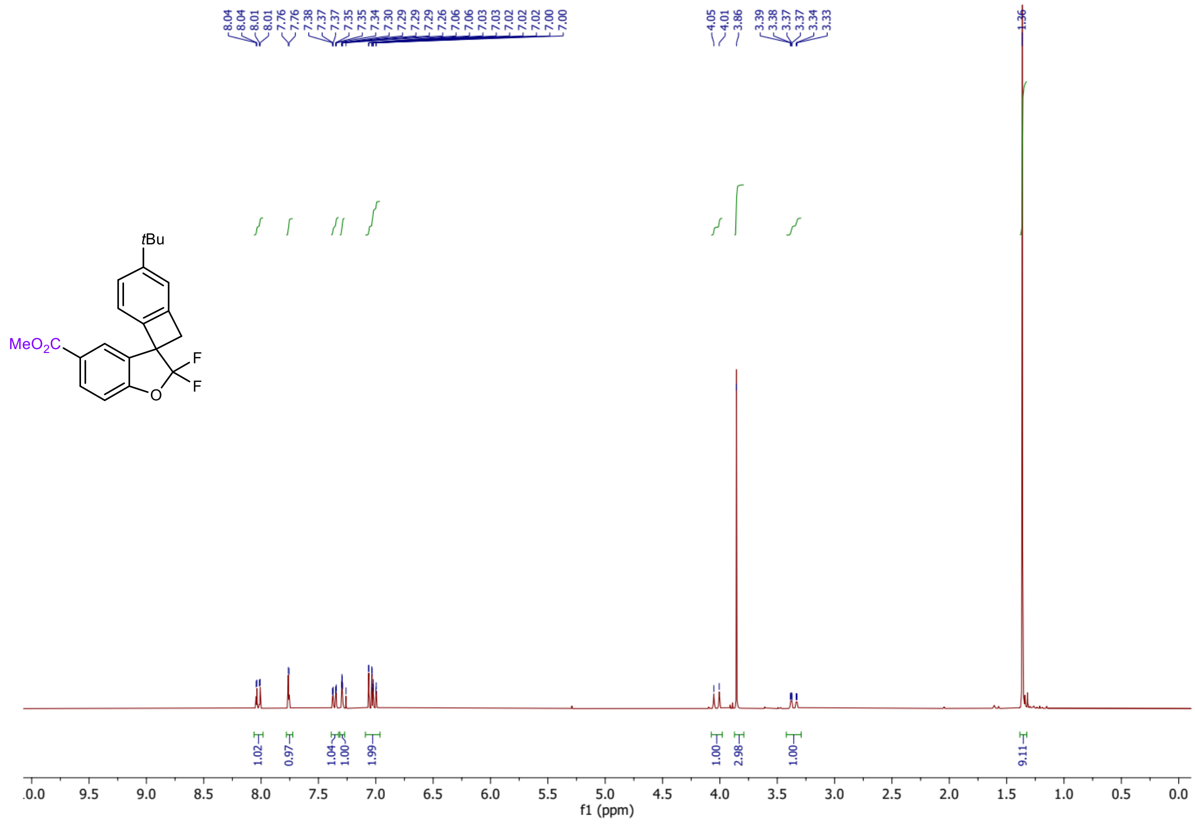

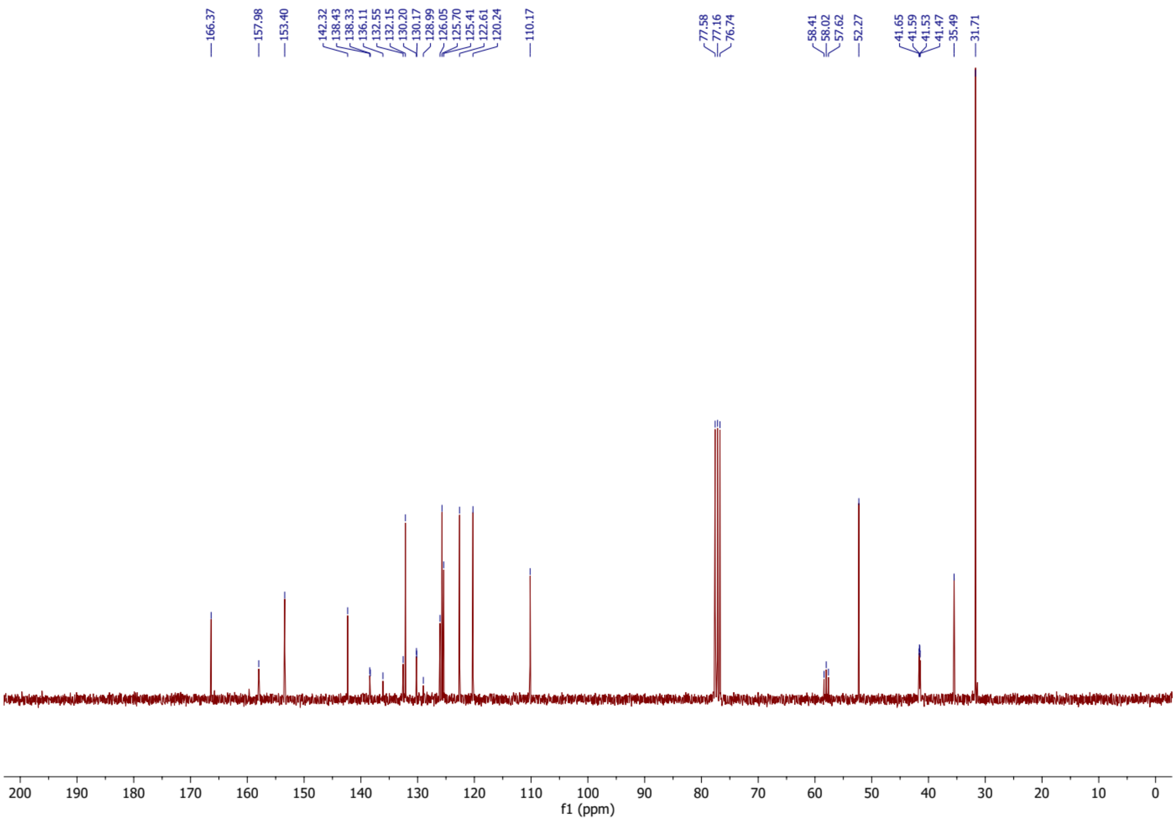
166.37
157.98
153.40
142.32
138.43
138.33
136.11
132.55
132.15
130.20
130.17
128.99
126.05
125.70
125.41
122.61
120.24
110.17
77.58
77.16
76.74
58.41
58.02
57.62
52.27
41.65
41.59
41.53
41.47
35.49
31.71
200
190
180
170
160
150
140
130
120
110
100
90
80
70
60
50
40
30
20
10
0
f1 (ppm)

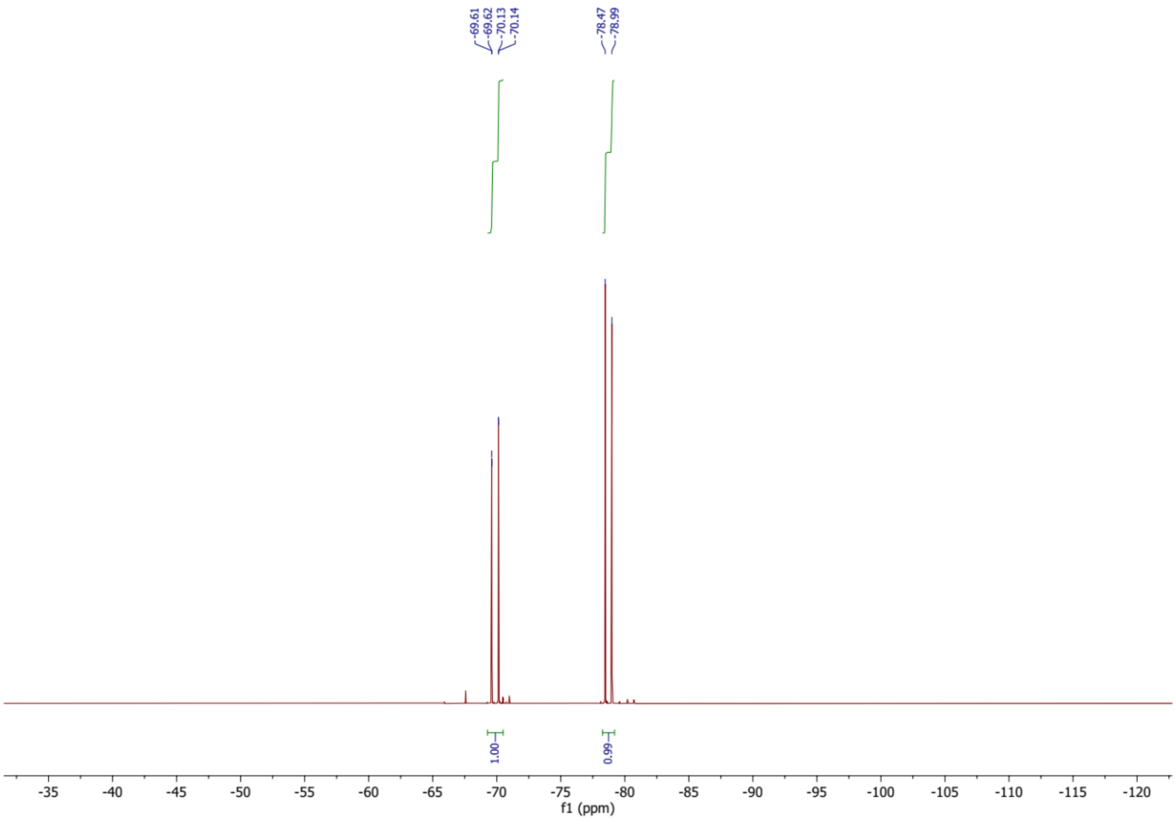
-69.61
-69.62
-70.13
-70.14
-78.47
-78.99
1.00
0.99
-35
-40
-45
-50
-55
-60
-65
-70
-75
-80
-85
-90
-95
-100
-105
-110
-115
-120
f1 (ppm)

4aa

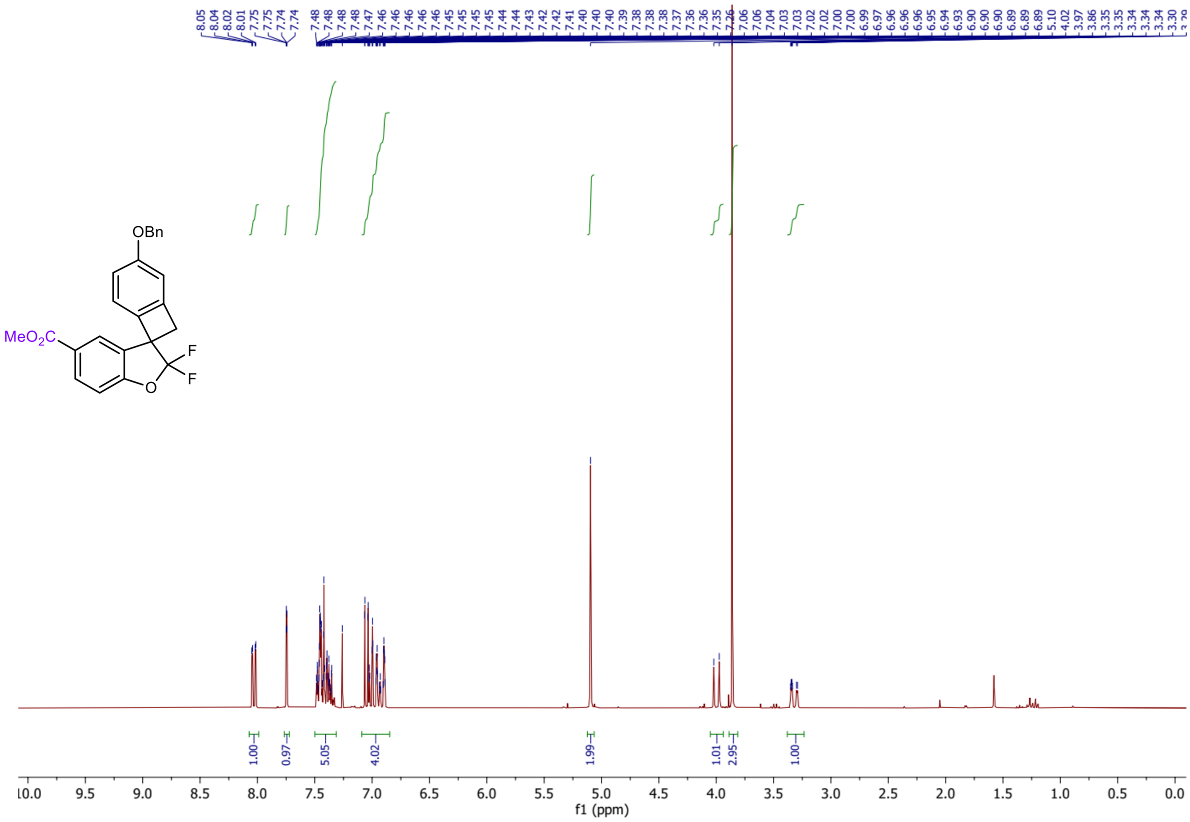

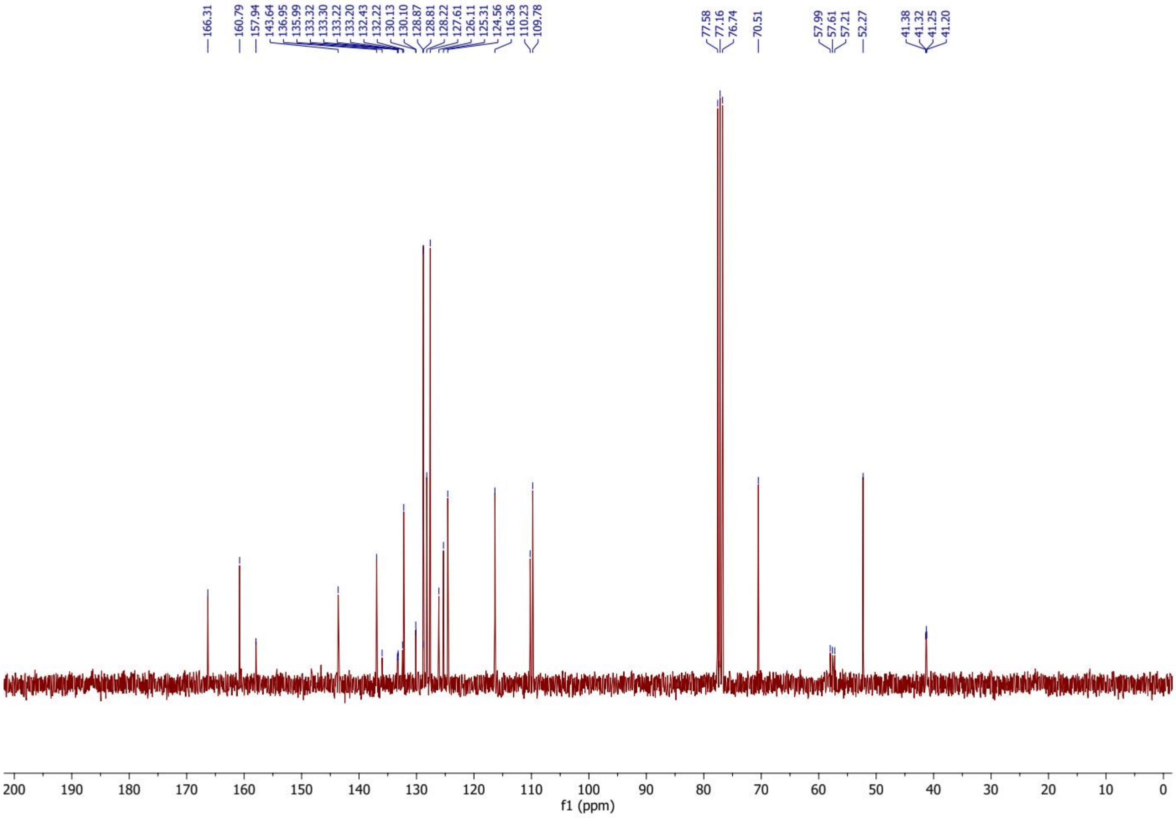

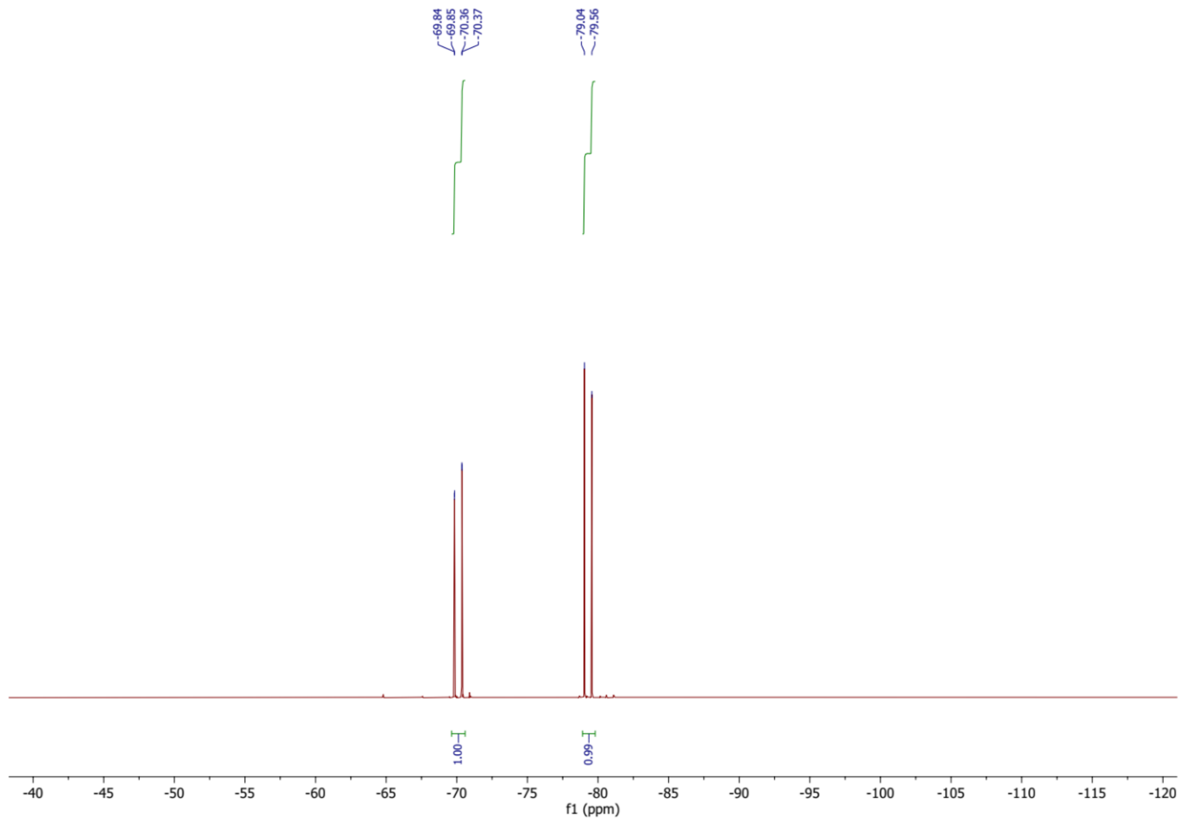
-69.84
-69.85
-70.36
-70.37
-79.04
-79.56
1.00
0.99
f1 (ppm)

4ab

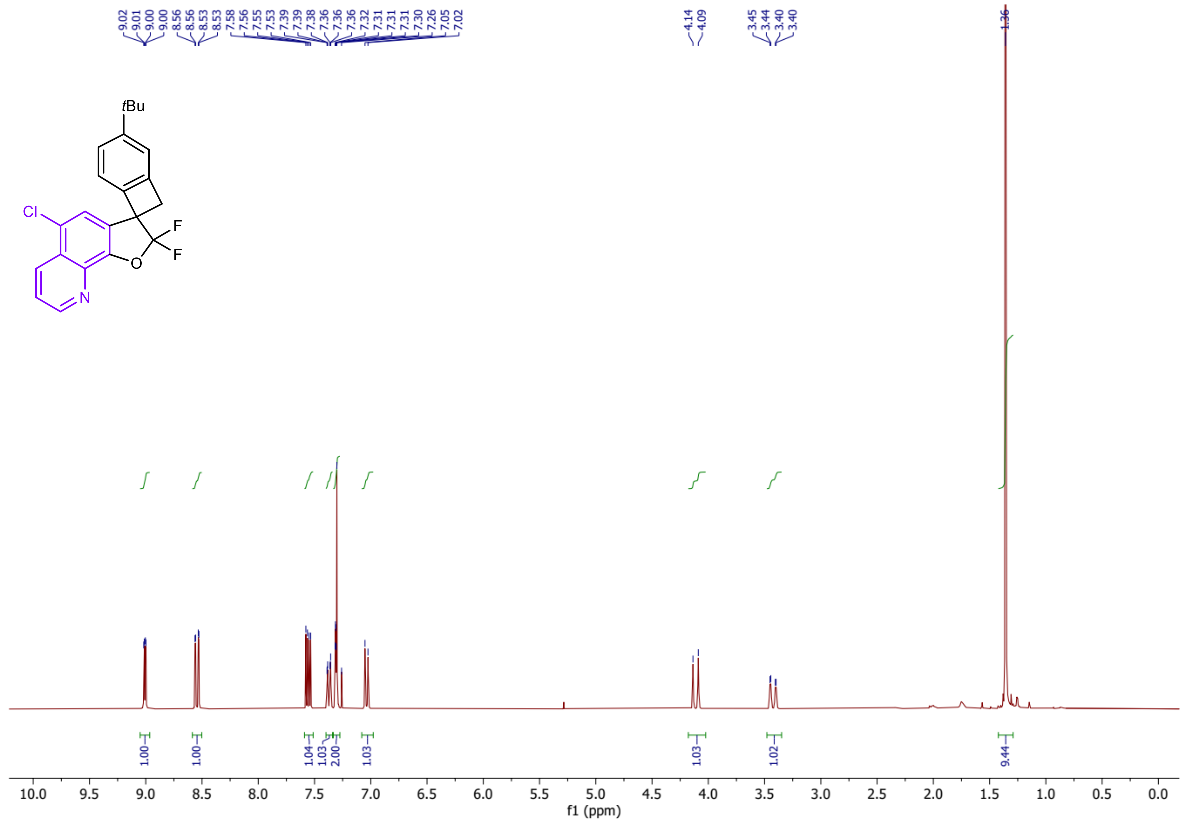
tBu
Cl
F
F
O
N
4.14
4.09
3.45
3.44
3.40
3.40
1.36
1.00
1.00
1.04
1.03
2.00
1.03
1.03
1.02
9.44
f1 (ppm)

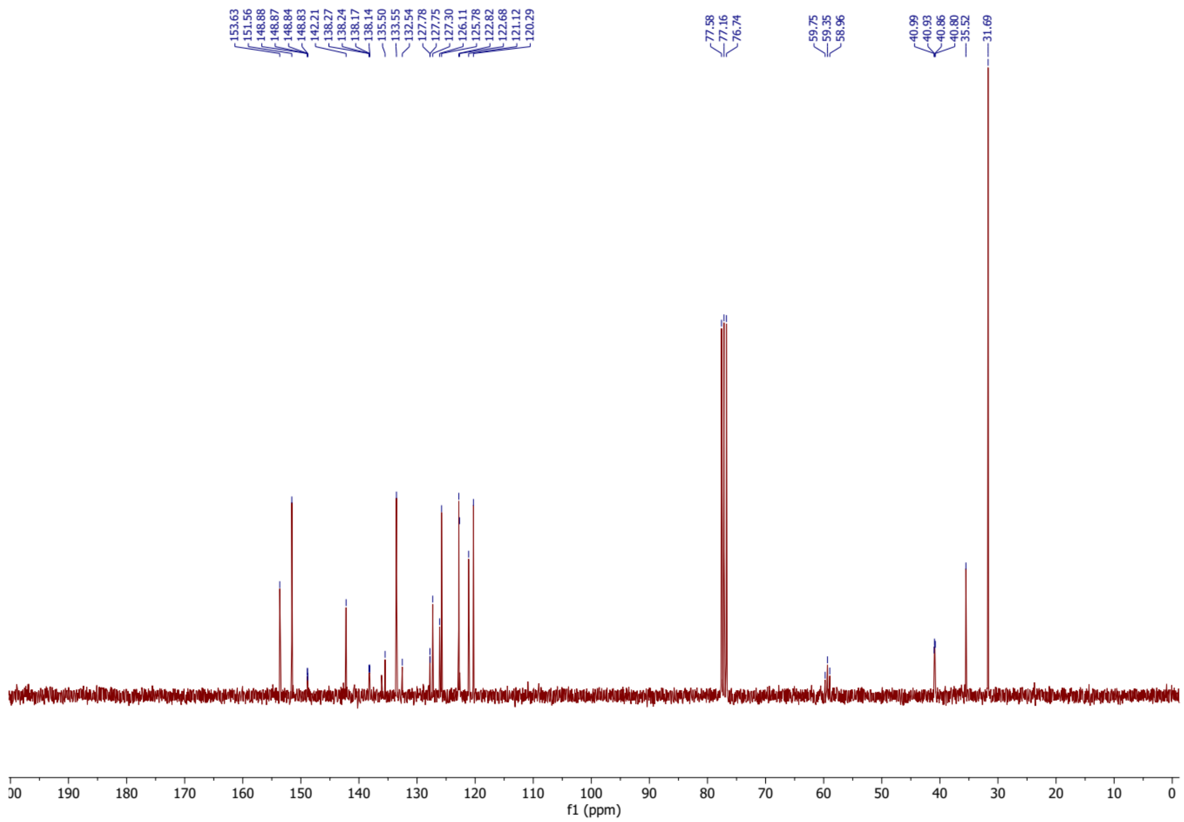
153.63
151.56
148.88
148.87
148.84
148.83
142.21
138.27
138.24
138.17
138.14
135.50
133.55
132.54
127.78
127.75
127.30
126.11
125.78
122.82
122.68
121.12
120.29
77.58
77.16
76.74
59.75
59.35
58.96
40.99
40.93
40.86
40.80
35.52
31.69
f1 (ppm)

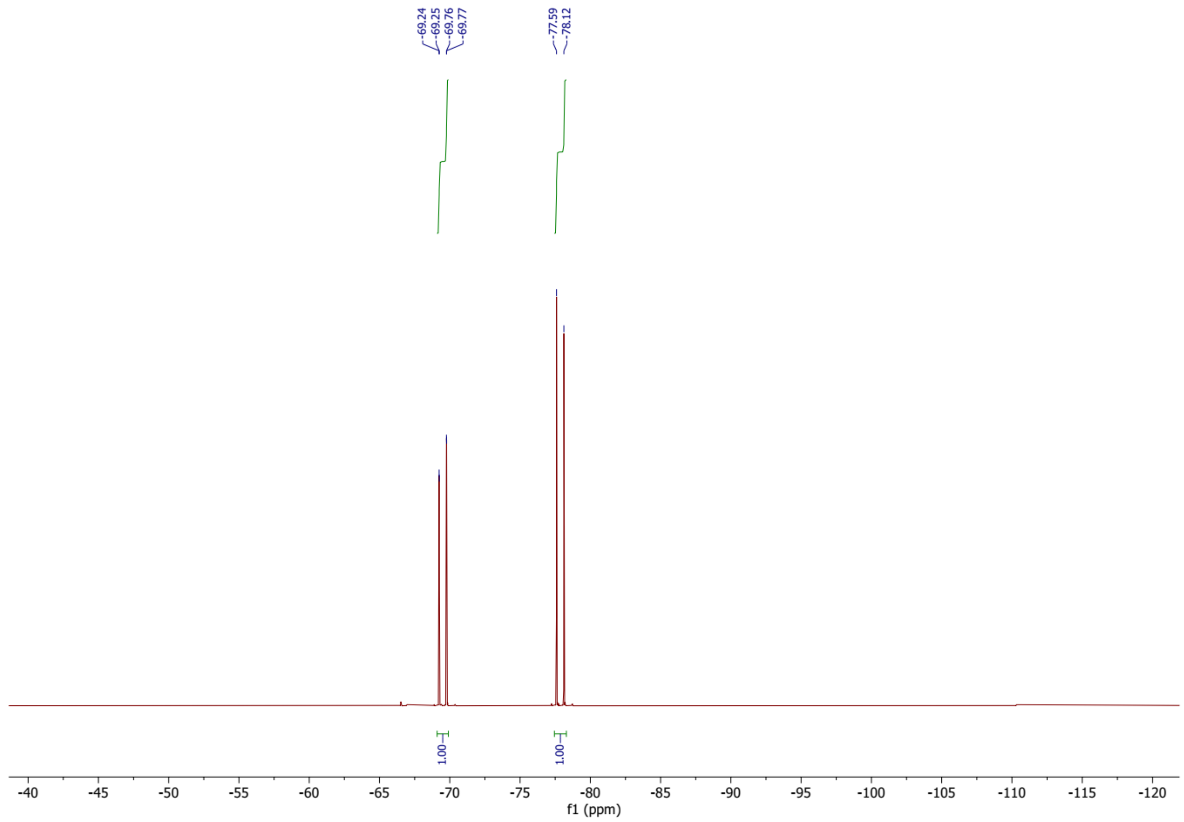
-69.24
-69.25
-69.76
-69.77
-77.59
-78.12
1.00
1.00
f1 (ppm)

4ac

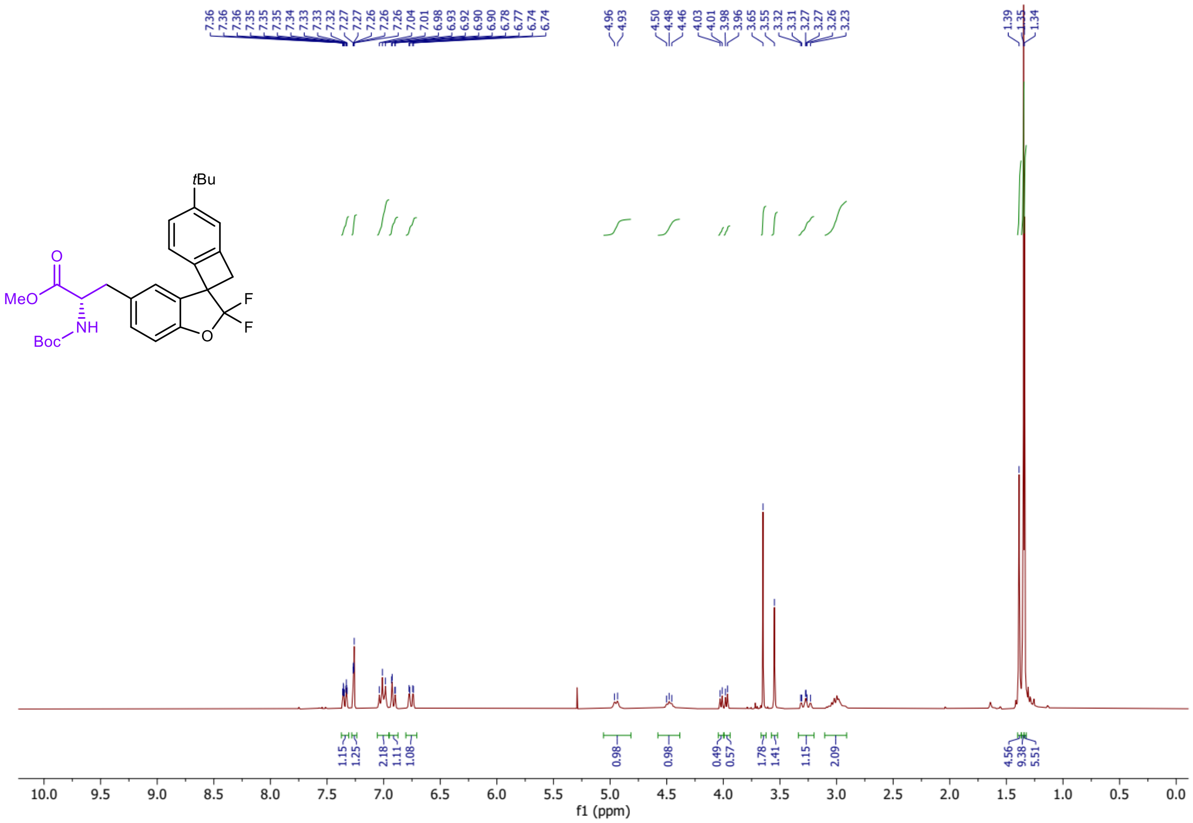
tBu
O
MeO
NH
Boc
F
F
O
7.36 7.36 7.36 7.35 7.35 7.34 7.33 7.33 7.32 7.27 7.27 7.26 7.26 7.04 7.01 6.98 6.93 6.92 6.90 6.78 6.77 6.74
4.96 4.93
4.50 4.48 4.46 4.03 4.01 3.98 3.96 3.65 3.55 3.32 3.31 3.27 3.26 3.23
1.39 1.35 1.34
1.15 1.25 2.18 1.11 1.08 0.98 0.98 0.49 0.57 1.78 1.41 1.15 2.09 4.56 9.38 5.51
f1 (ppm)

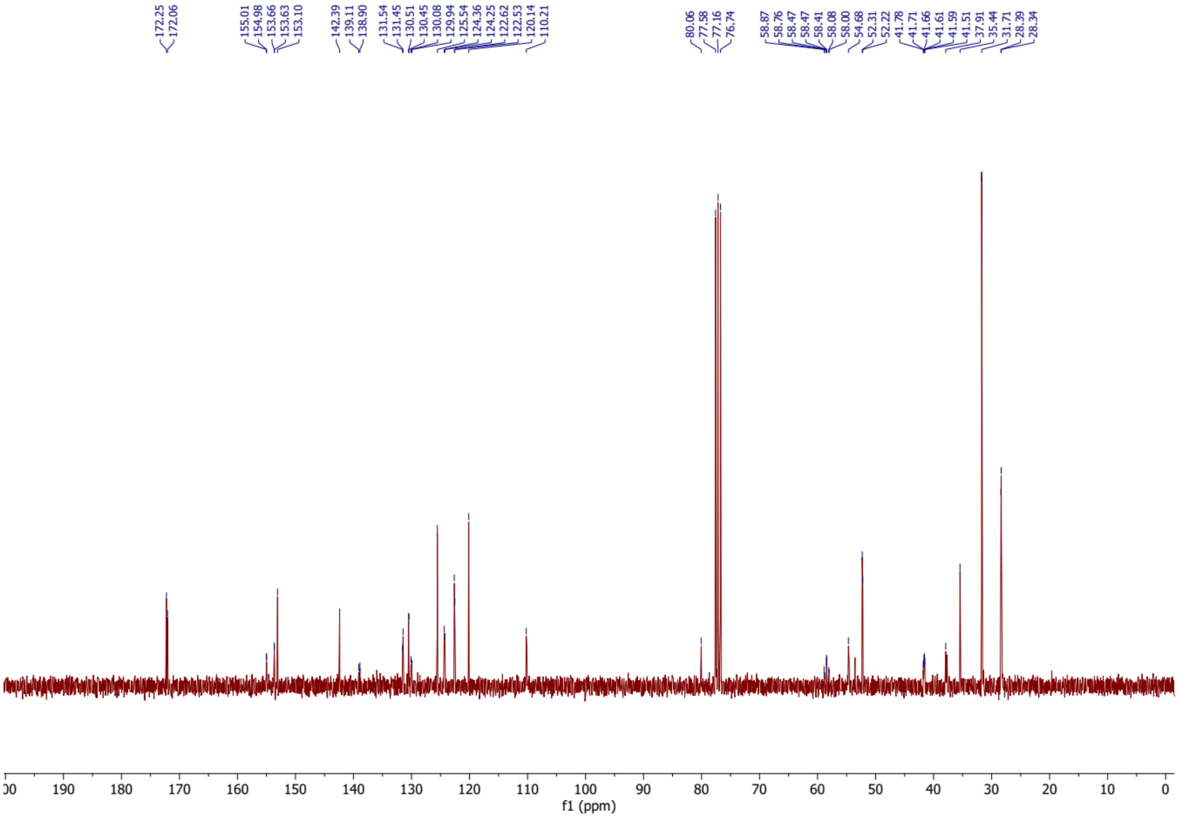
172.25 172.06
155.01 154.98 153.66 153.63 153.10
142.39 139.11 138.90 131.54 131.45 130.51 130.45 130.08 129.94 125.54 124.36 124.25 122.62 122.53 120.14 110.21
80.06 77.58 77.16 76.74
58.87 58.76 58.47 58.47 58.41 58.08 58.00 54.68 52.31 52.22 41.78 41.71 41.66 41.61 41.59 41.51 37.91 35.44 31.71 28.39 28.34
f1 (ppm)

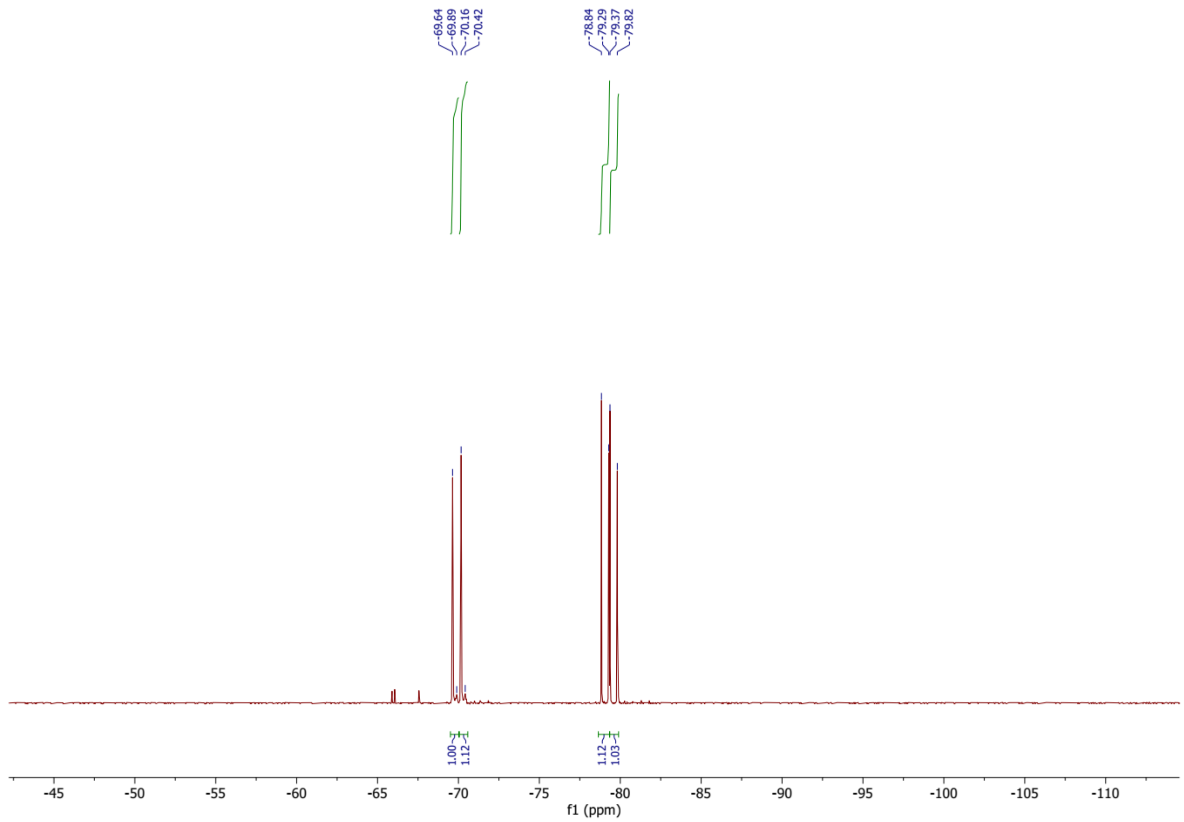

4ad

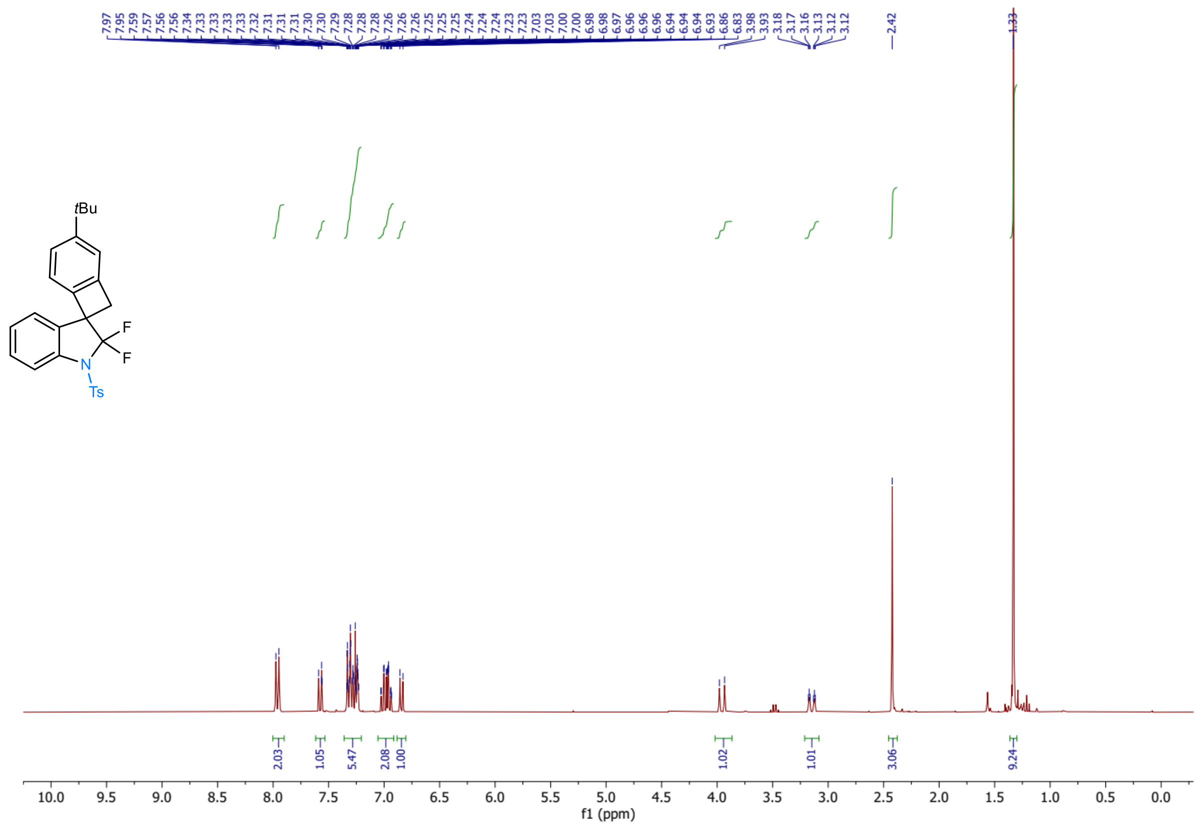

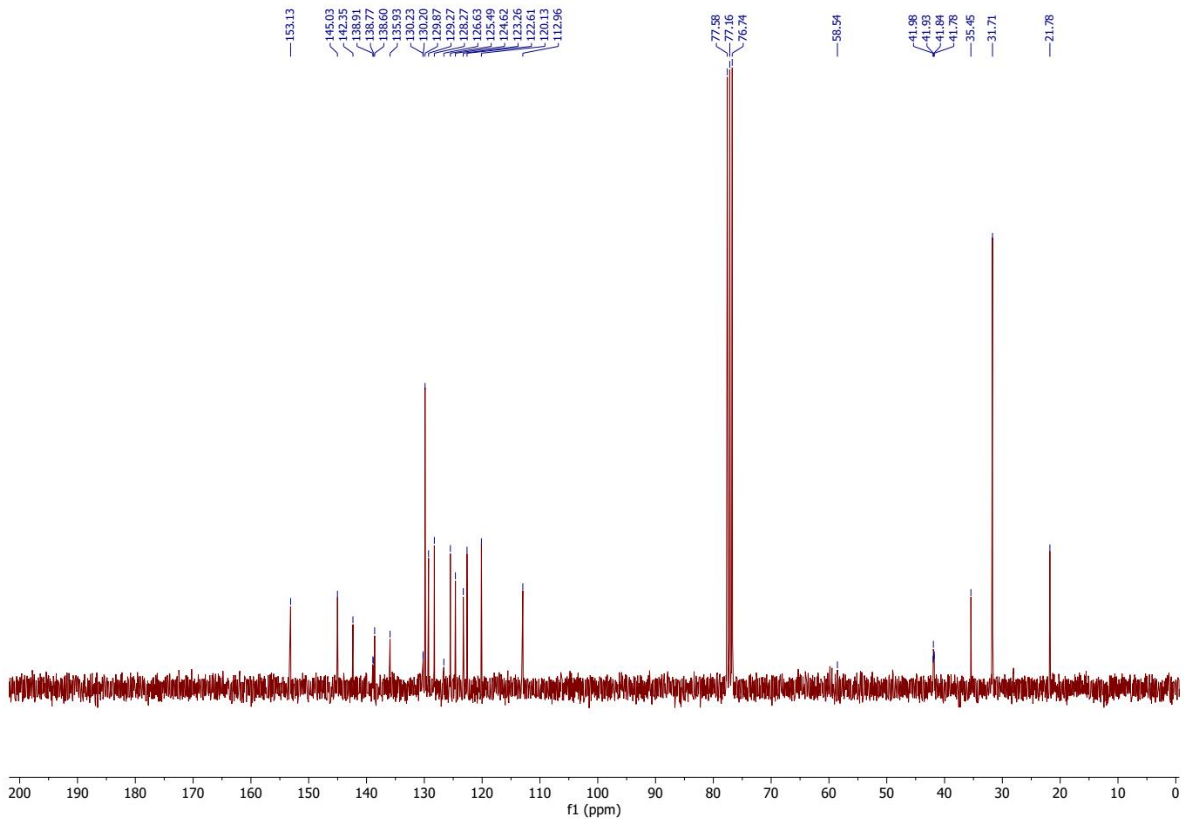
153.13
145.03
142.35
138.91
138.77
138.60
135.93
130.23
130.20
129.87
129.27
128.27
126.63
125.49
124.62
123.26
122.61
120.13
112.96
77.58
77.16
76.74
58.54
41.98
41.93
41.84
41.78
35.45
31.71
21.78
200 190 180 170 160 150 140 130 120 110 100 90 80 70 60 50 40 30 20 10 0
f1 (ppm)

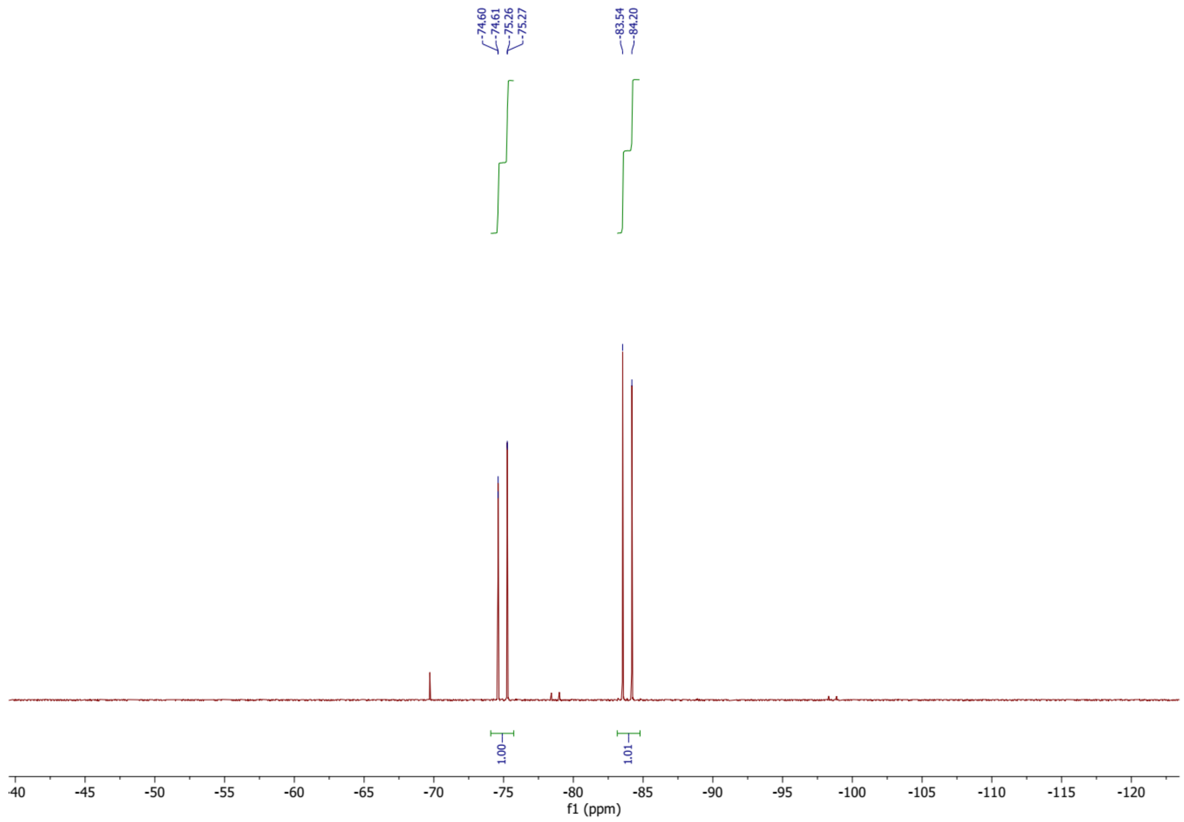
-74.60
-74.61
-75.26
-75.27
-83.54
-84.20
1.00
1.01
-40 -45 -50 -55 -60 -65 -70 -75 -80 -85 -90 -95 -100 -105 -110 -115 -120
f1 (ppm)

4ae

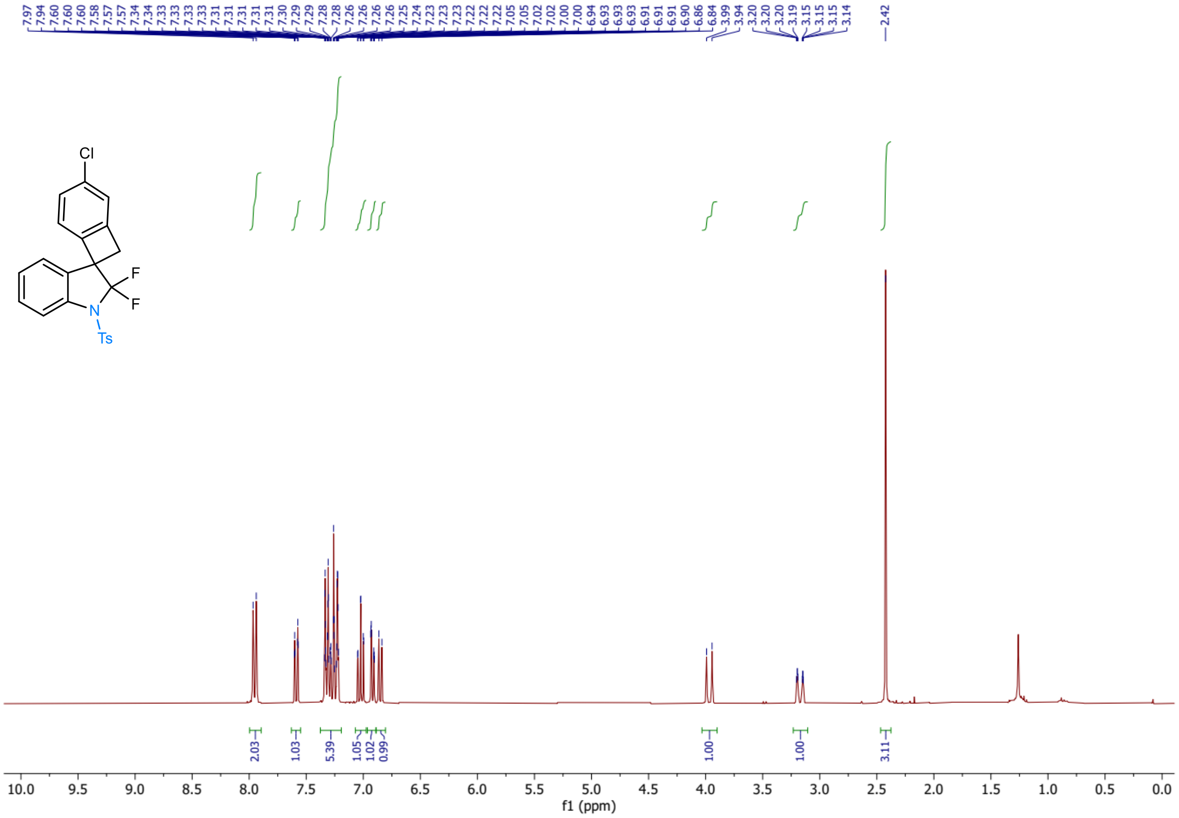
Cl
F
F
N
Ts
7.97
7.94
7.60
7.60
7.58
7.57
7.57
7.34
7.34
7.33
7.33
7.33
7.33
7.31
7.31
7.31
7.31
7.30
7.29
7.29
7.28
7.28
7.26
7.26
7.26
7.26
7.25
7.24
7.23
7.23
7.23
7.22
7.22
7.22
7.05
7.05
7.02
7.02
7.00
7.00
6.94
6.93
6.93
6.91
6.91
6.91
6.90
6.86
6.84
3.99
3.94
3.20
3.20
3.19
3.15
3.15
3.15
3.14
2.42
2.03
1.03
5.39
1.05
1.02
0.99
1.00
1.00
3.11
f1 (ppm)

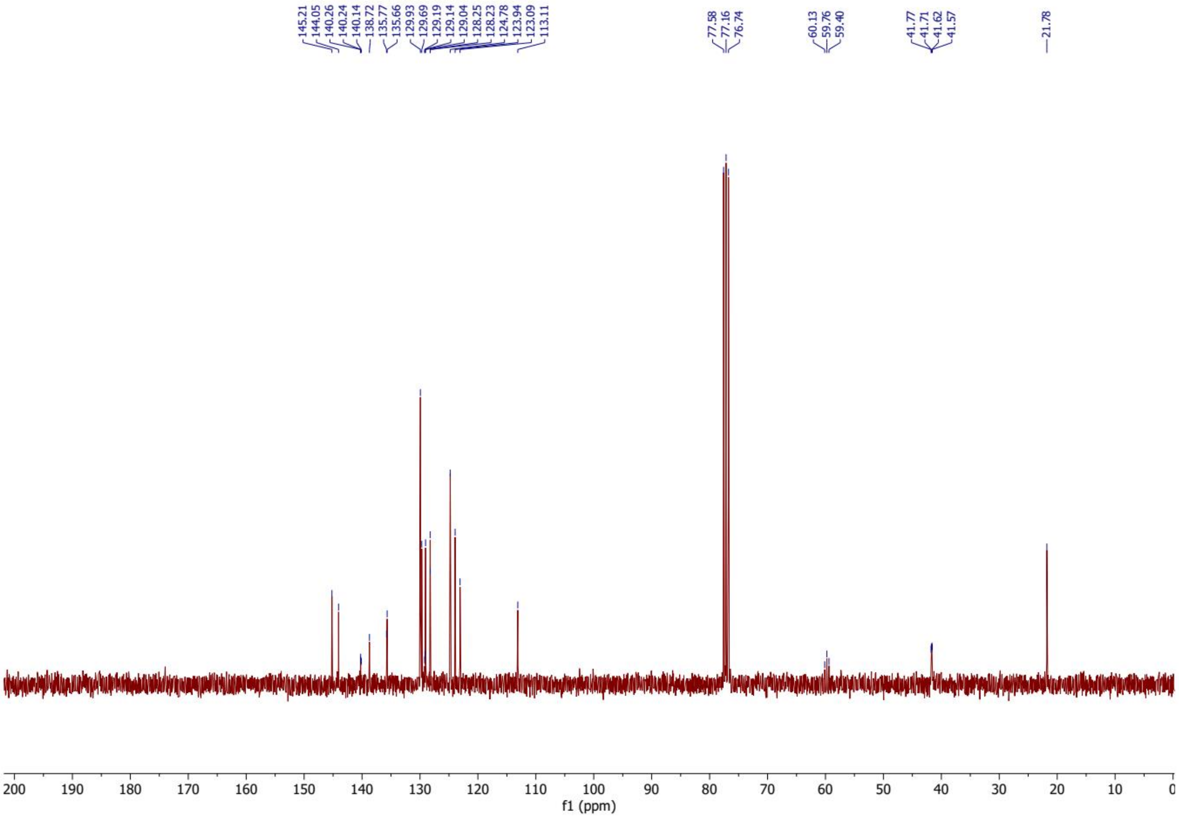
145.21
144.05
140.26
140.24
140.14
138.72
135.77
135.66
129.93
129.69
129.19
129.14
129.04
128.25
128.23
124.78
123.94
123.09
113.11
77.58
77.16
76.74
60.13
59.76
59.40
41.77
41.71
41.62
41.57
21.78
f1 (ppm)

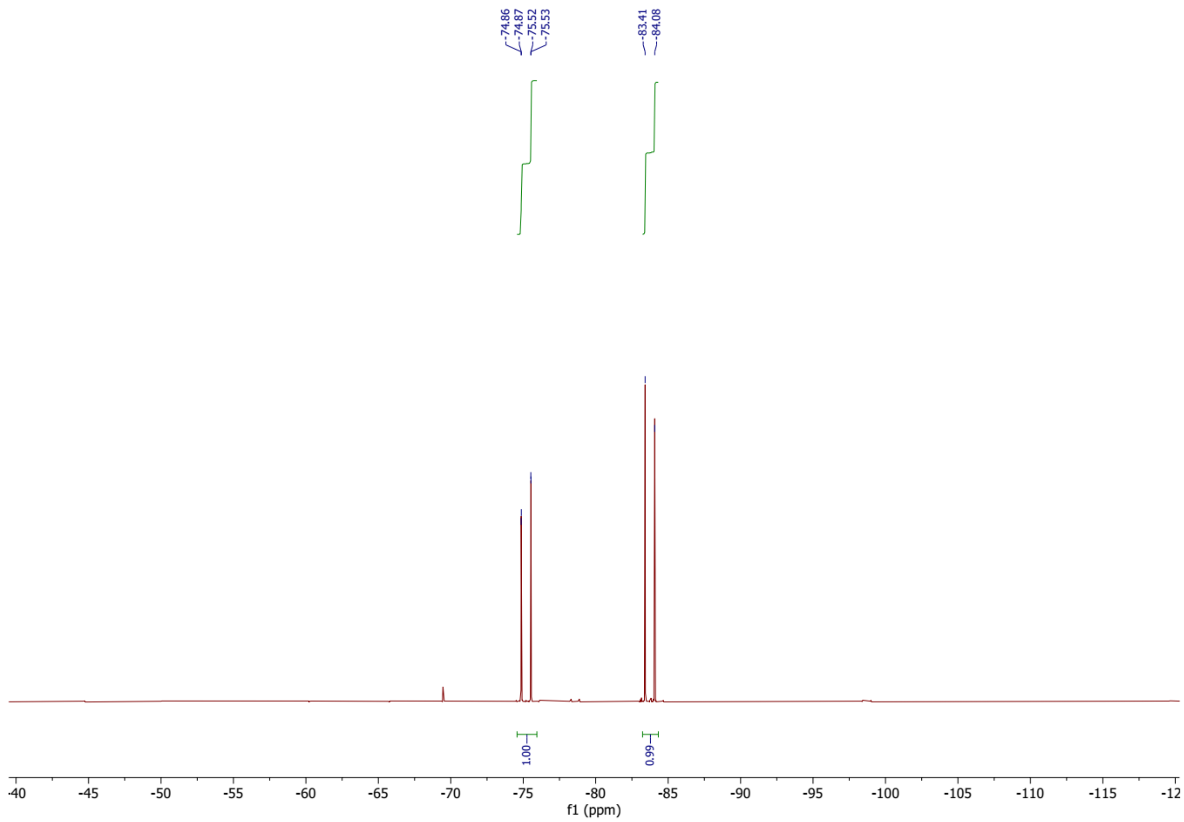

6a

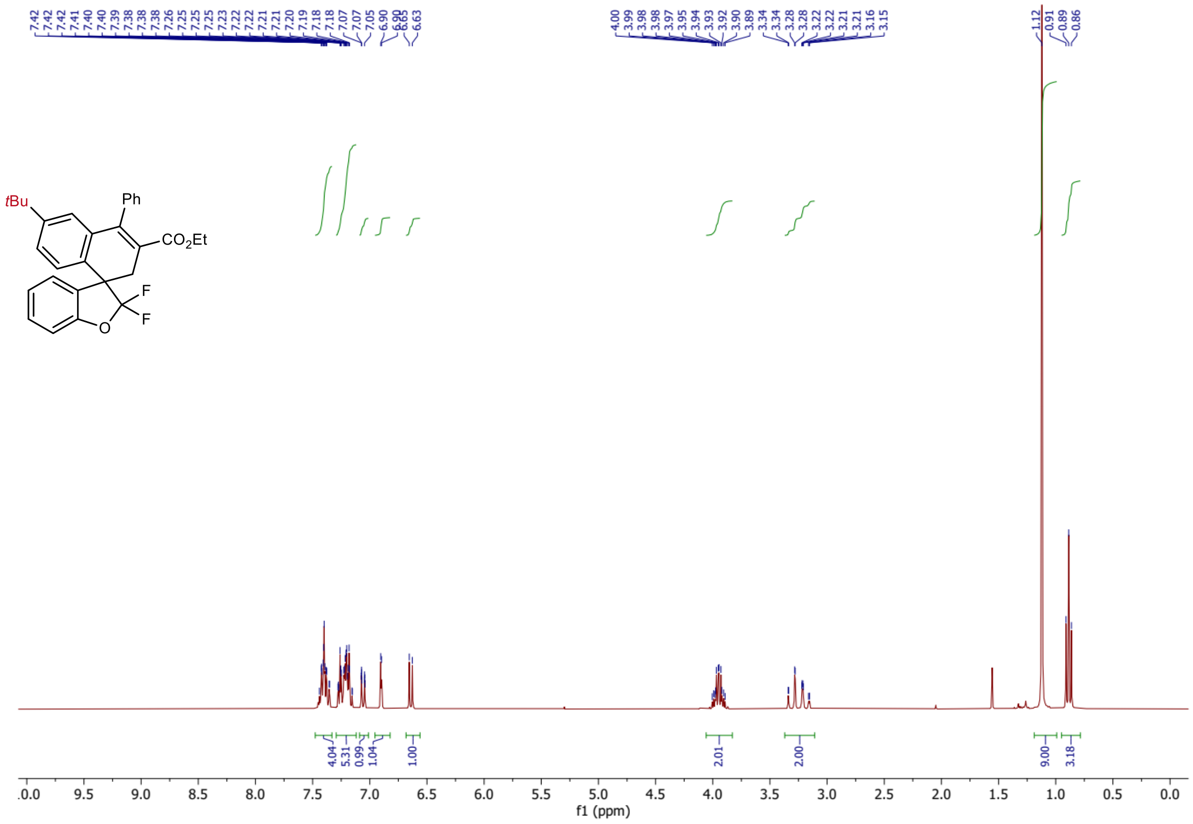

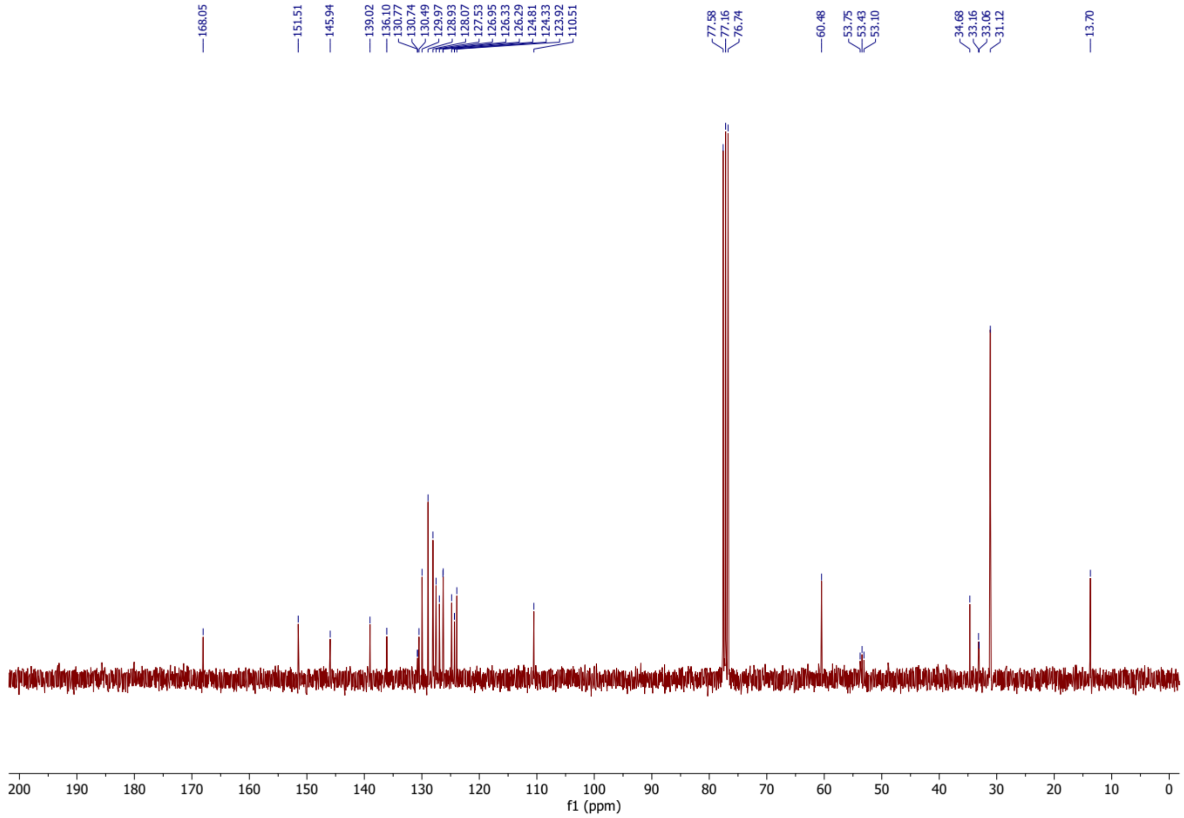
168.05
151.51
145.94
139.02
136.10
130.77
130.74
130.49
129.97
128.93
128.07
127.53
126.95
126.33
126.29
124.81
124.33
123.92
110.51
77.58
77.16
76.74
60.48
53.75
53.43
53.10
34.68
33.16
33.06
31.12
13.70
200 190 180 170 160 150 140 130 120 110 100 90 80 70 60 50 40 30 20 10 0
f1 (ppm)

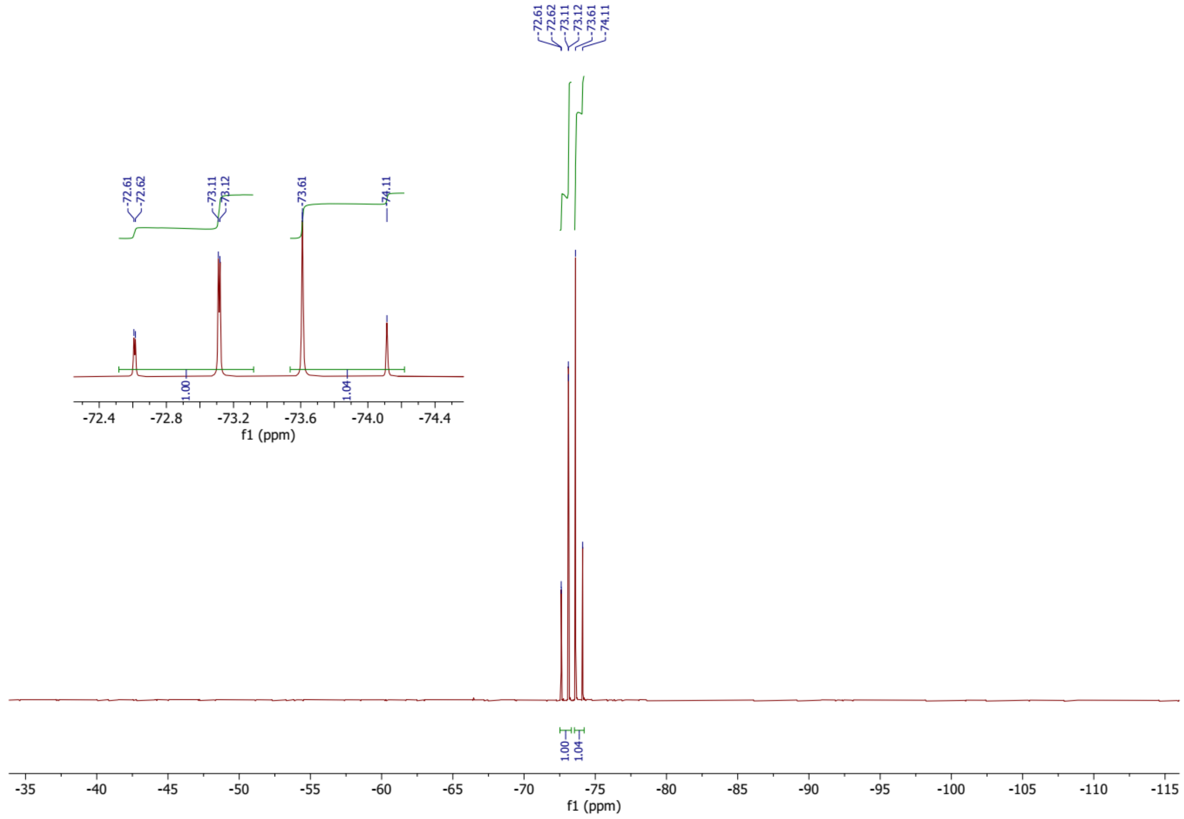
-72.61
-72.62
-73.11
-73.12
-73.61
-74.11
1.00
1.04
-72.4 -72.8 -73.2 -73.6 -74.0 -74.4
f1 (ppm)
-35 -40 -45 -50 -55 -60 -65 -70 -75 -80 -85 -90 -95 -100 -105 -110 -115
f1 (ppm)

6b

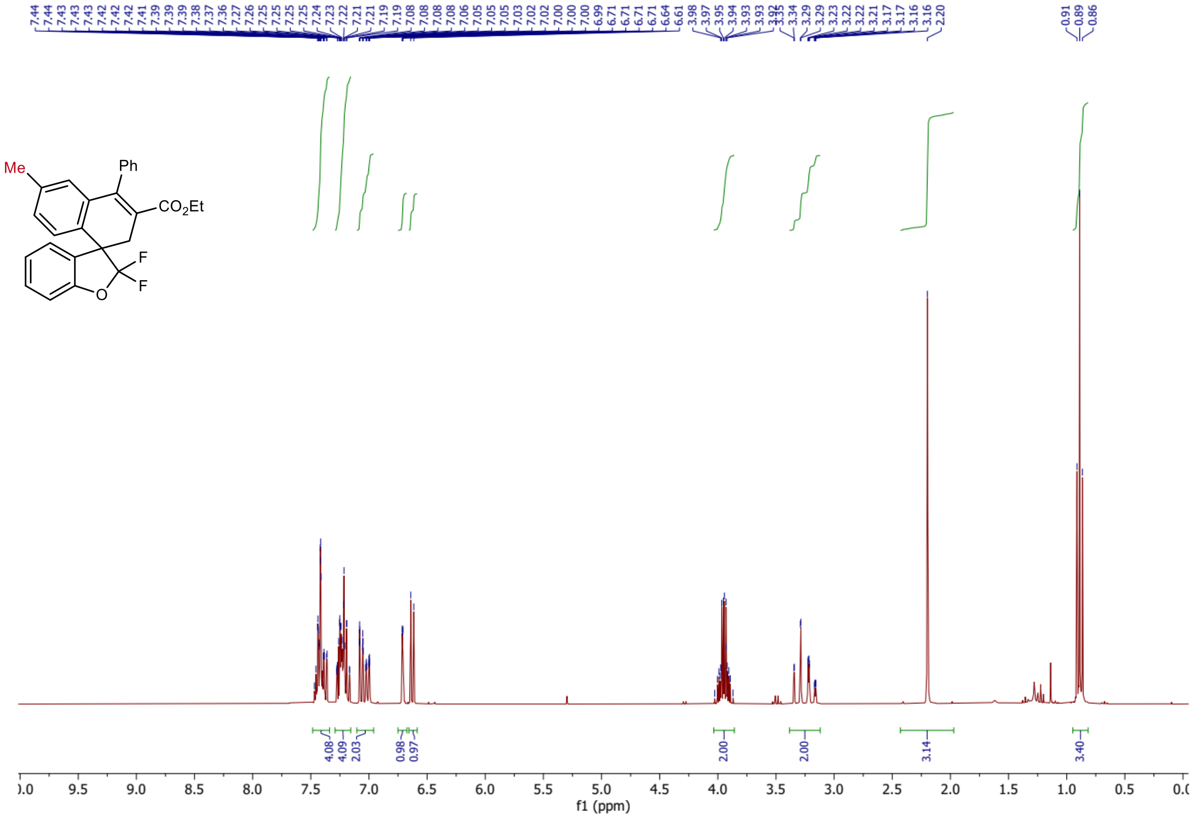

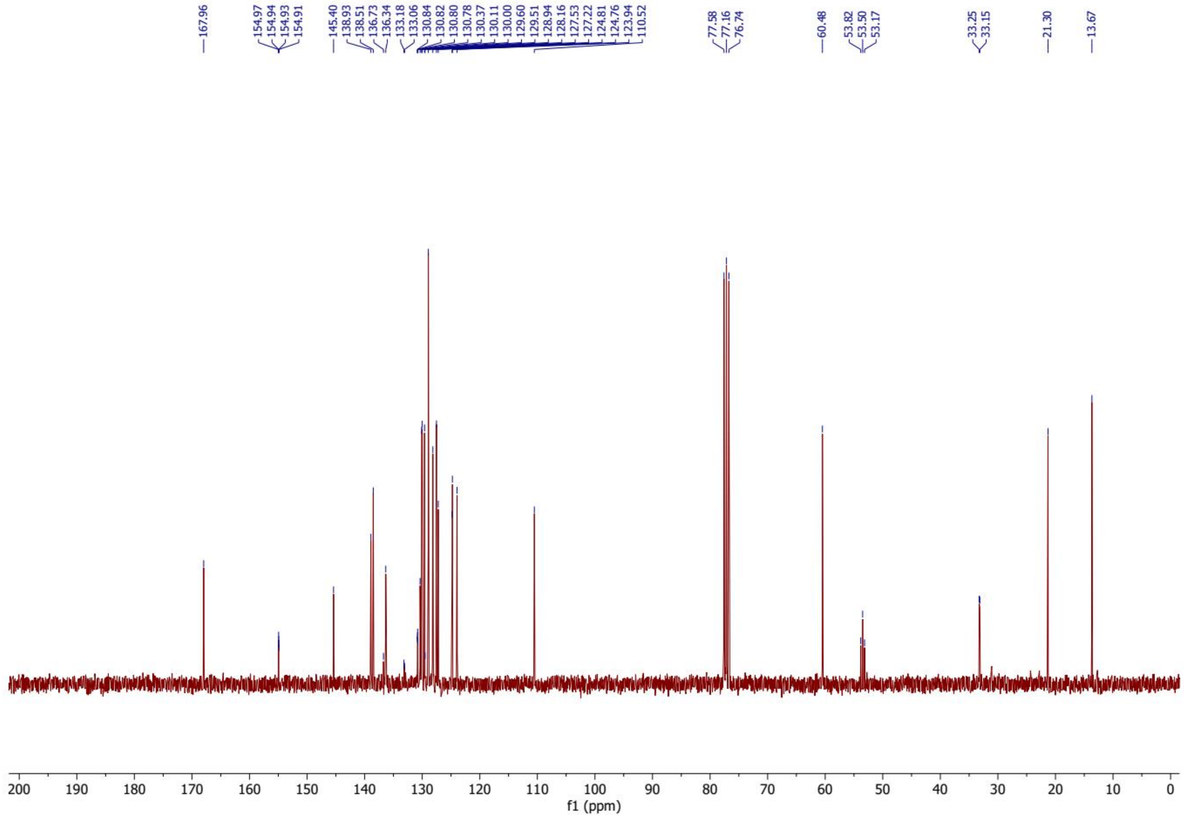

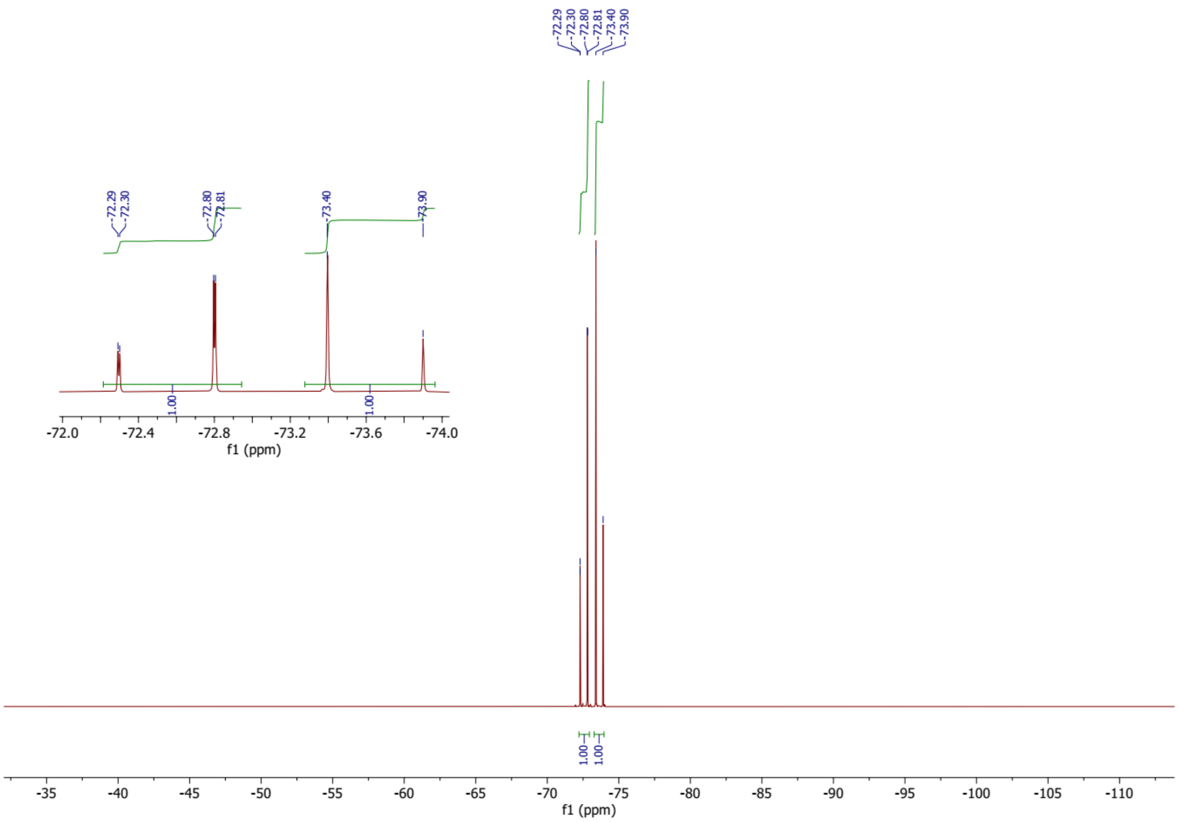

6c

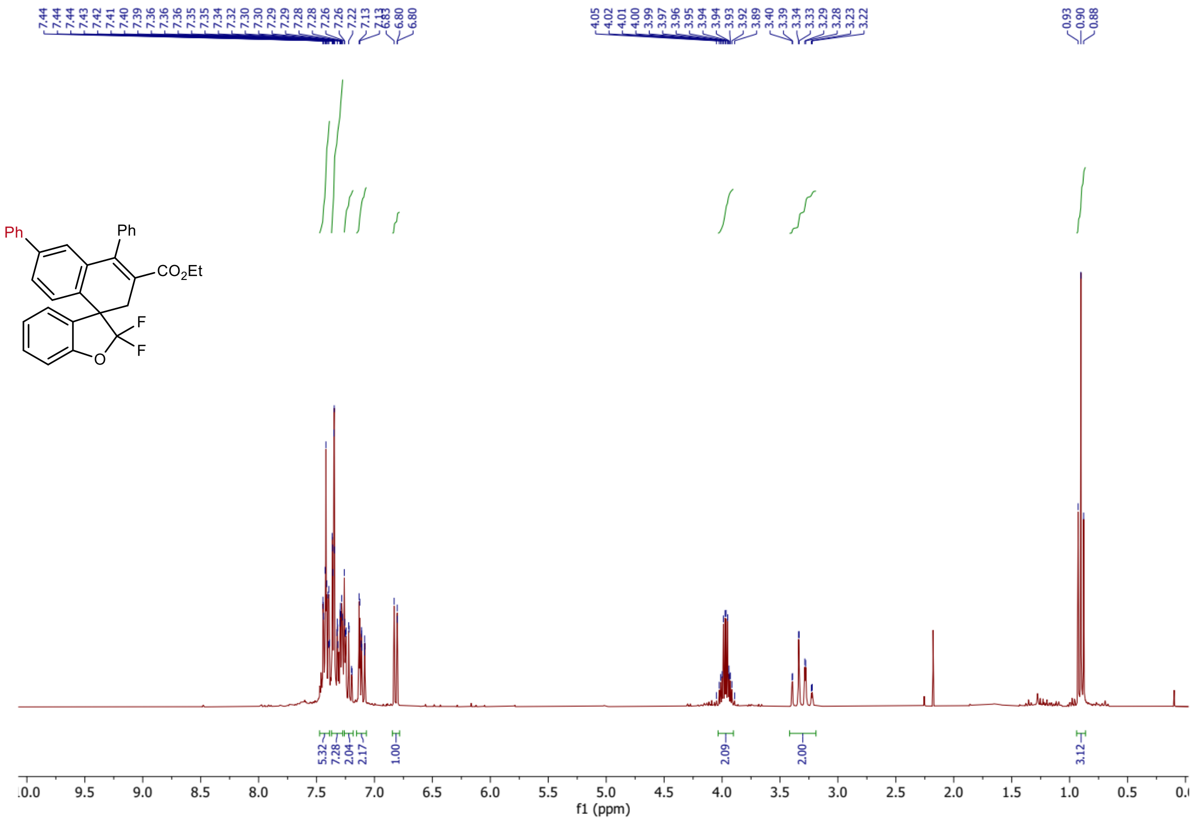

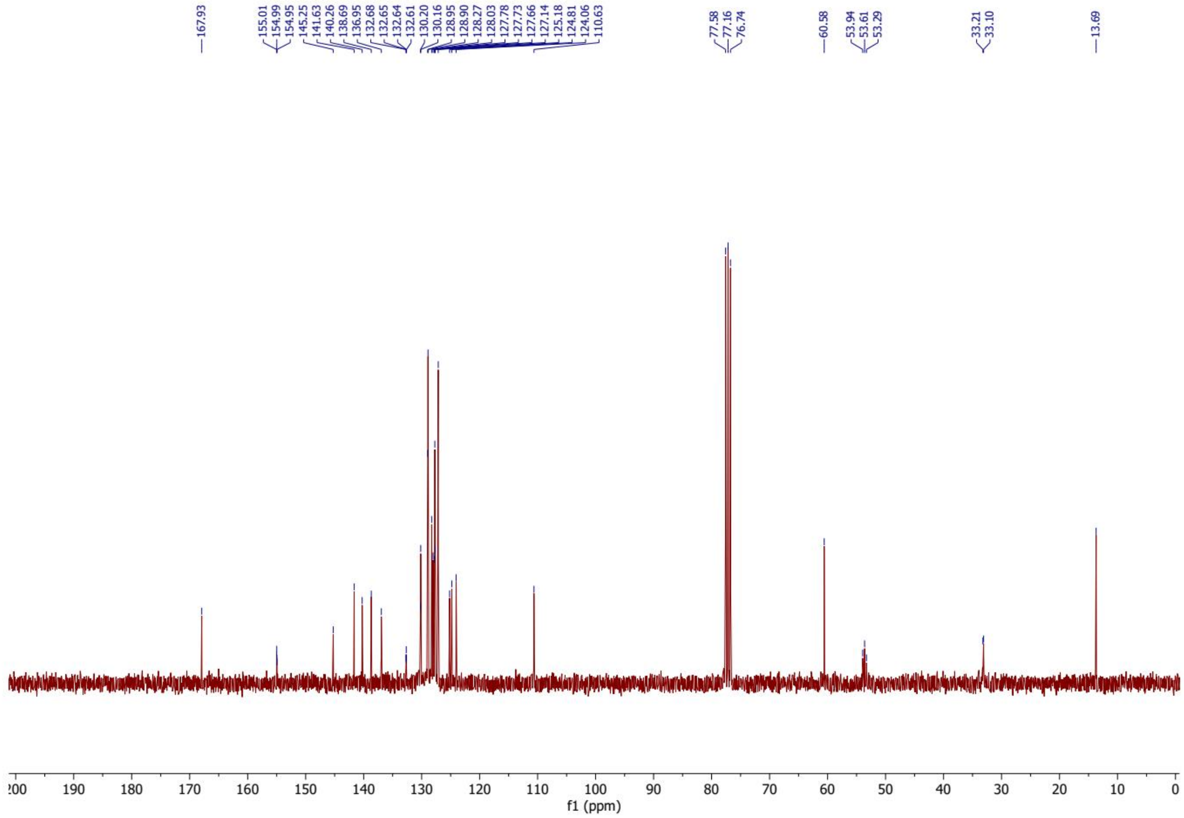
167.93
155.01
154.99
154.95
145.25
141.63
140.26
138.69
136.95
132.68
132.65
132.64
132.61
130.20
130.16
128.95
128.90
128.27
128.03
127.78
127.73
127.66
127.14
125.18
124.81
124.06
110.63
77.58
77.16
76.74
60.58
53.94
53.61
53.29
33.21
33.10
13.69
f1 (ppm)

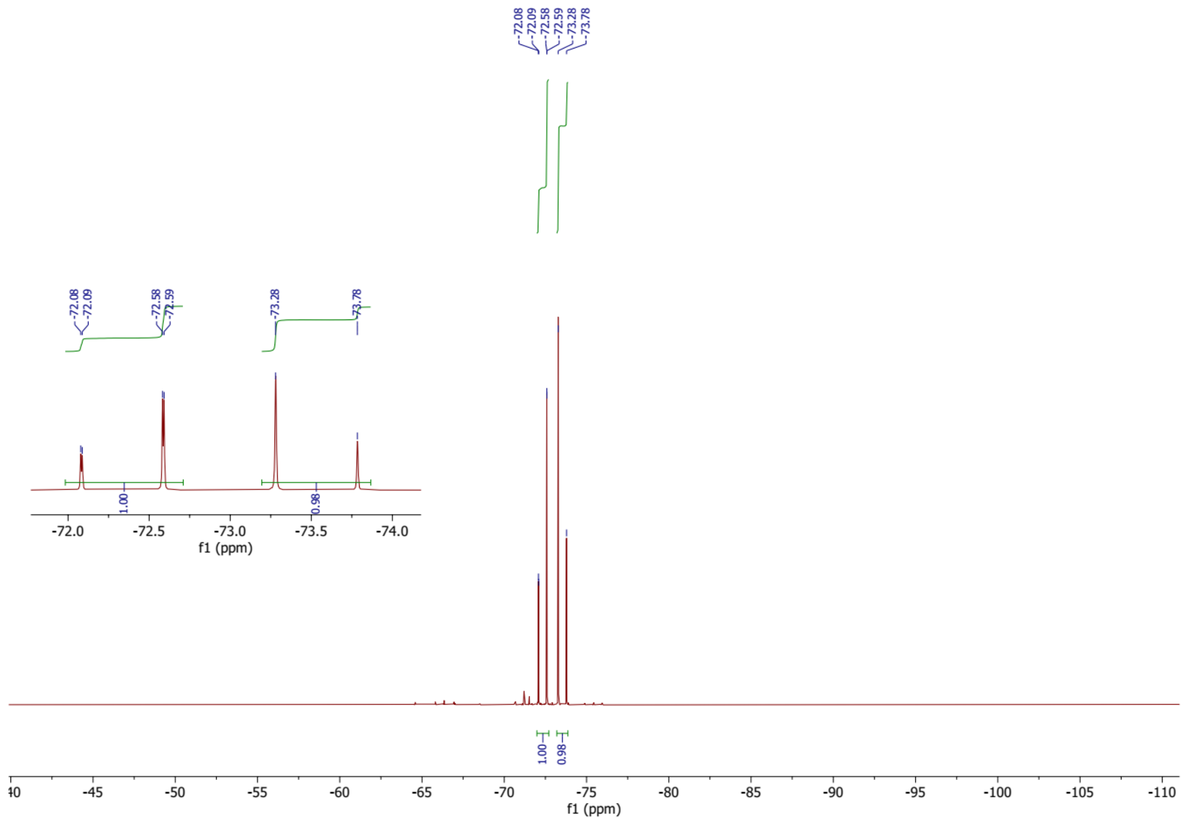
-72.08
-72.09
-72.58
-72.59
-73.28
-73.78
1.00
0.98
f1 (ppm)

6d

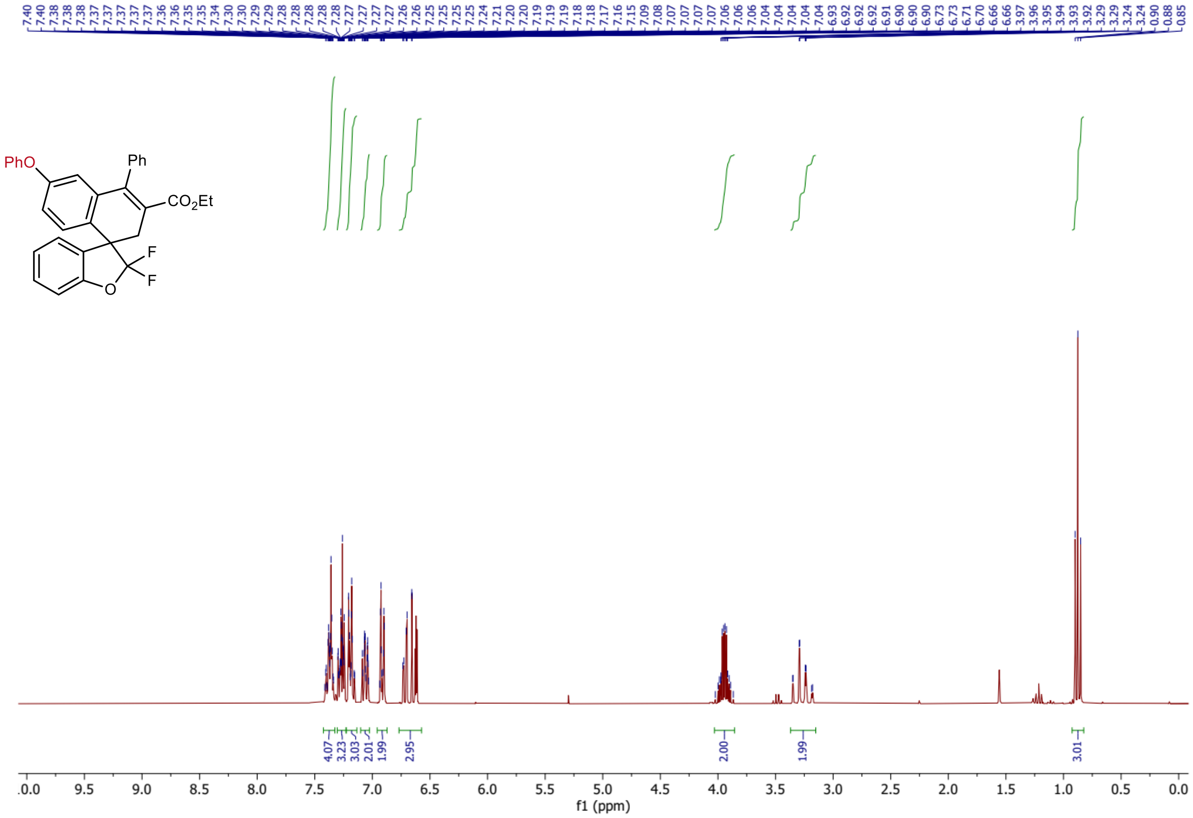
7.40 7.40 7.38 7.38 7.37 7.37 7.37 7.37 7.36 7.36 7.35 7.35 7.34 7.30 7.30 7.29 7.29 7.28 7.28 7.28 7.28 7.27 7.27 7.27 7.27 7.26 7.26 7.25 7.25 7.25 7.25 7.24 7.21 7.20 7.20 7.19 7.19 7.19 7.18 7.18 7.17 7.16 7.15 7.09 7.08 7.07 7.07 7.07 7.07 7.06 7.06 7.06 7.04 7.04 7.04 7.04 6.93 6.92 6.92 6.92 6.91 6.90 6.90 6.90 6.73 6.71 6.70 6.66 6.66 3.97 3.96 3.95 3.94 3.93 3.92 3.29 3.29 3.24 3.24 0.90 0.88 0.85
PhO
Ph
CO_2Et
F
F
O
4.07 3.23 3.03 2.01 1.99 2.95 2.00 1.99 3.01
f1 (ppm)

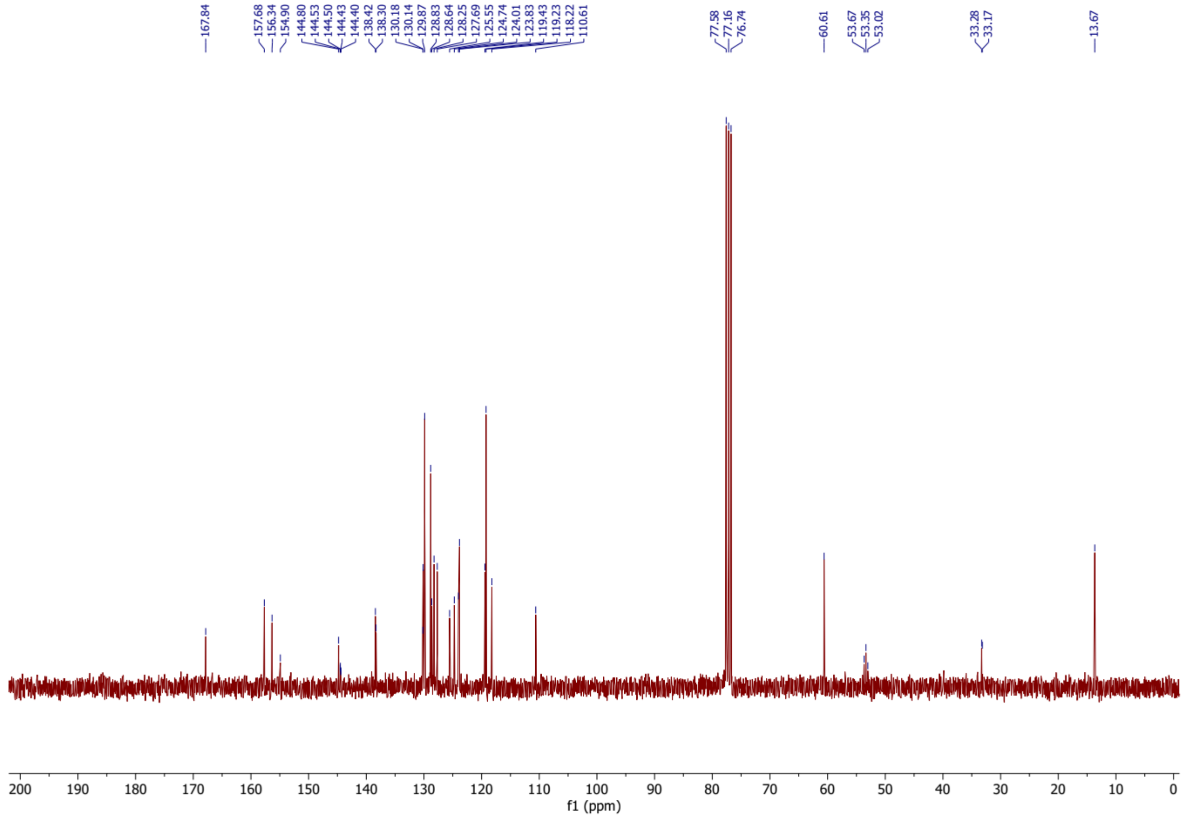
167.84
157.68 156.34 154.90 144.80 144.53 144.50 144.43 144.40 138.42 138.30 130.18 130.14 129.87 128.83 128.64 128.25 127.69 125.55 124.74 124.01 123.83 119.43 119.23 118.22 110.61
77.58 77.16 76.74
60.61
53.67 53.35 53.02
33.28 33.17
13.67
f1 (ppm)

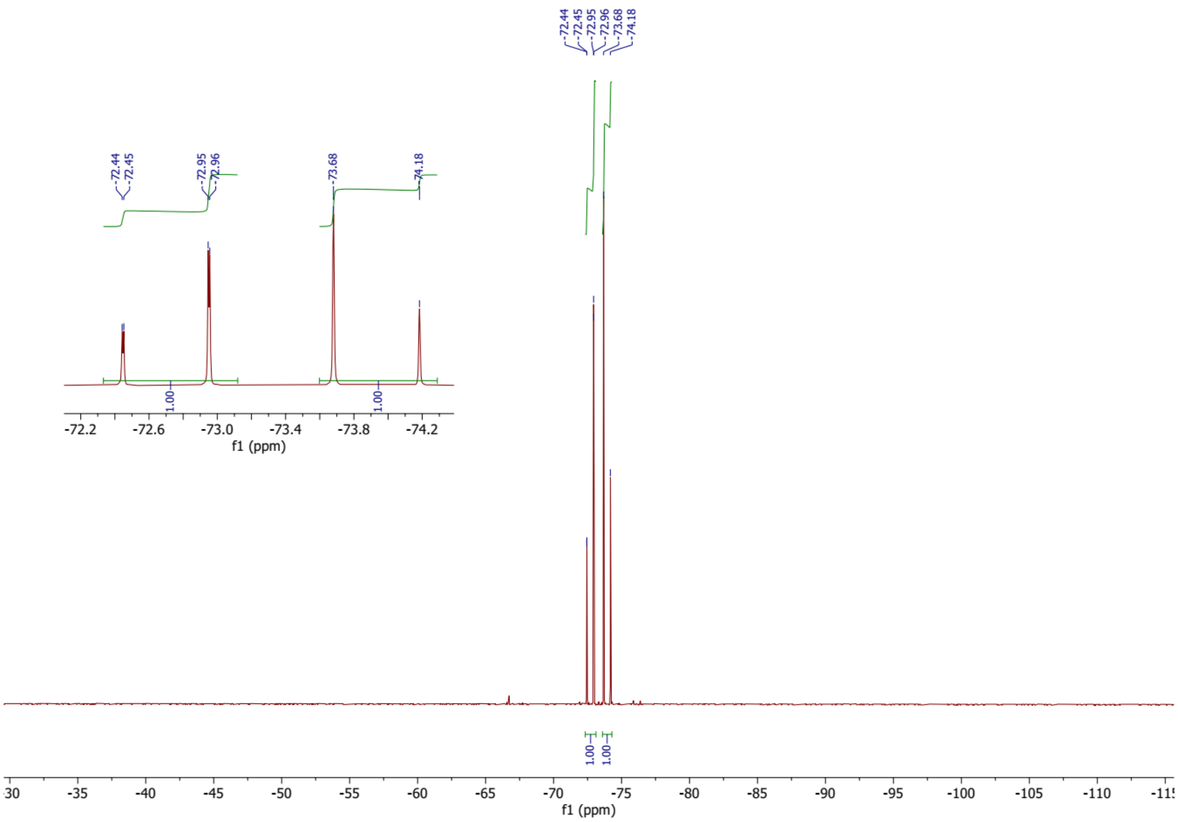

4e

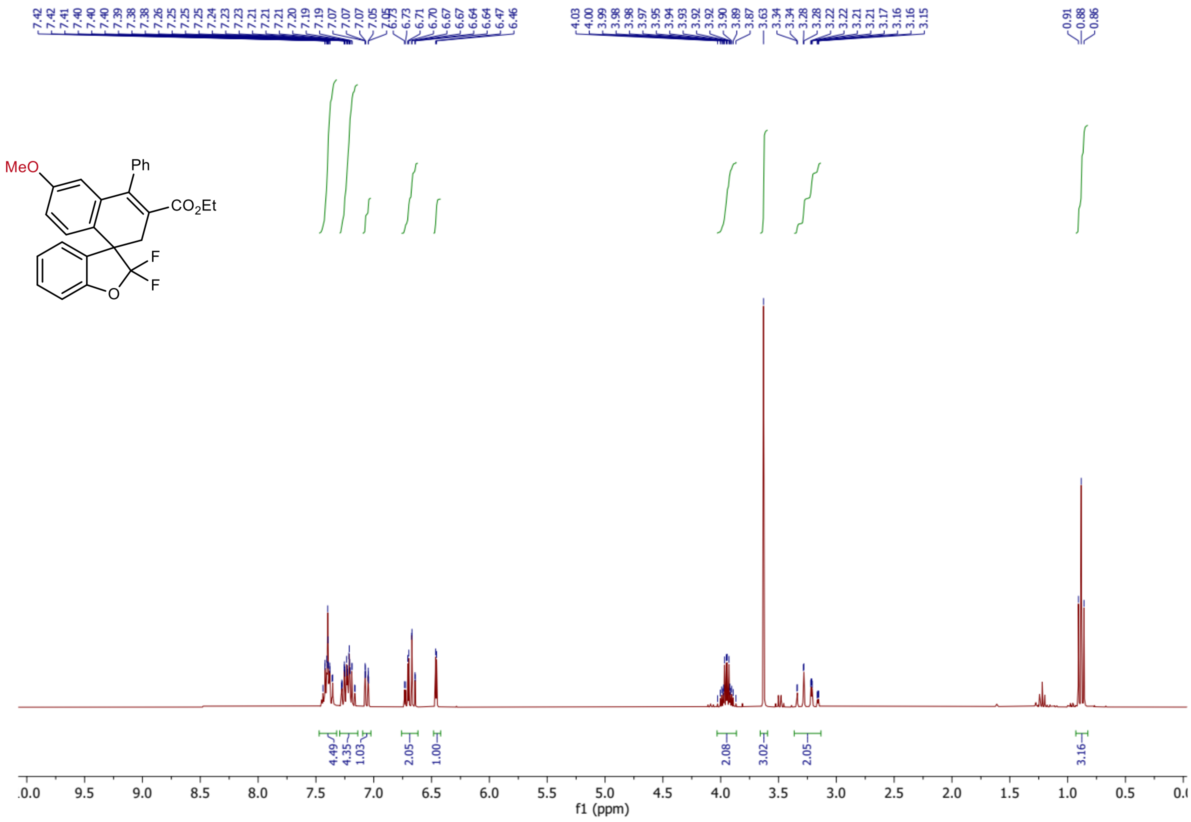

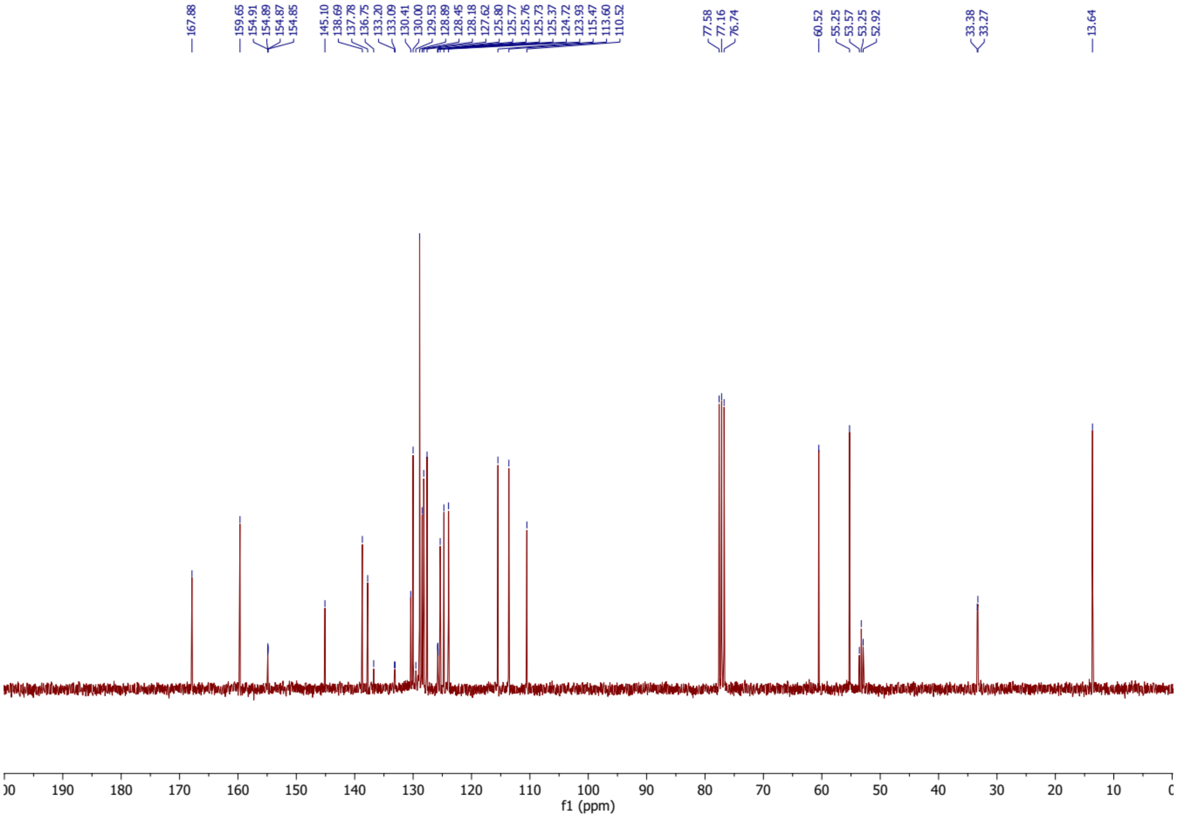

167.88
159.65
154.91
154.89
154.87
154.85
145.10
138.69
137.78
136.75
133.20
133.09
130.41
130.00
129.53
128.89
128.45
128.18
127.62
125.80
125.77
125.76
125.73
125.37
124.72
123.93
115.47
113.60
110.52
77.58
77.16
76.74
60.52
55.25
53.57
53.25
52.92
33.38
33.27
13.64
f1 (ppm)

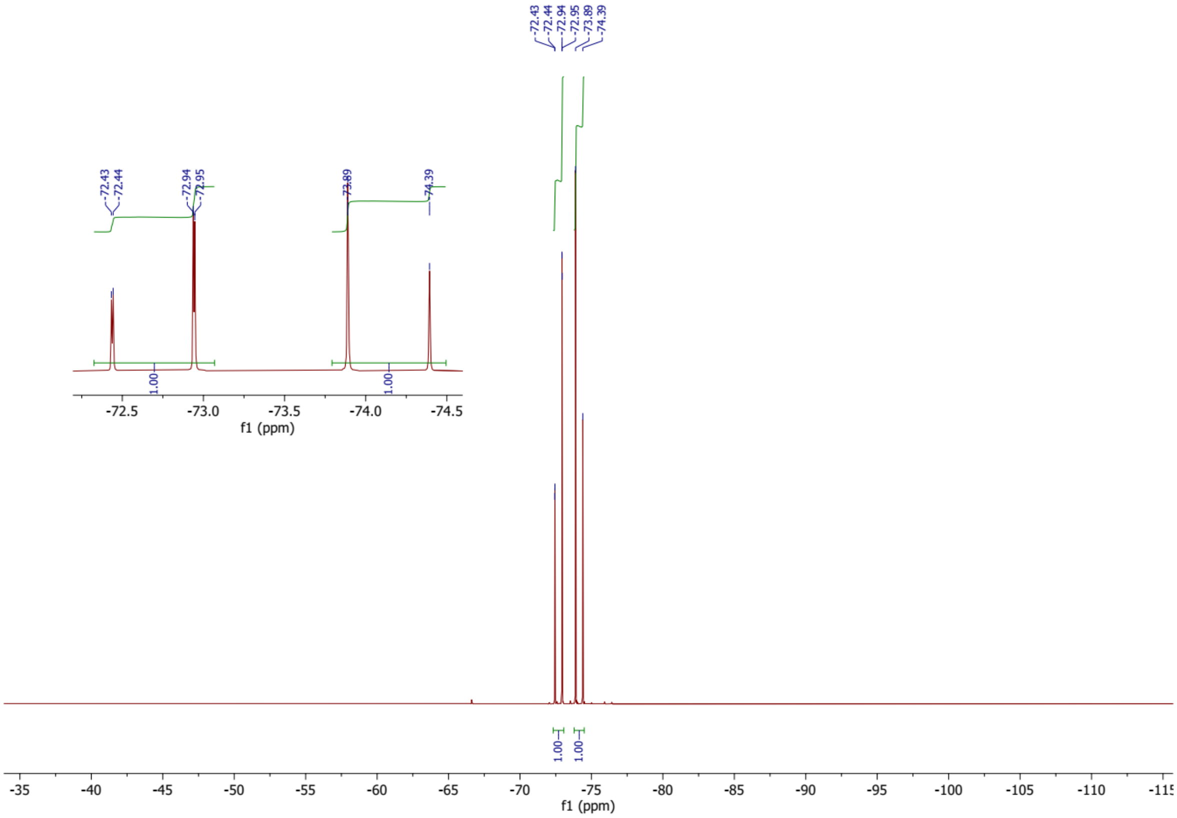

-72.43
-72.44
-72.94
-72.95
-73.89
-74.39
1.00
1.00
f1 (ppm)

6f

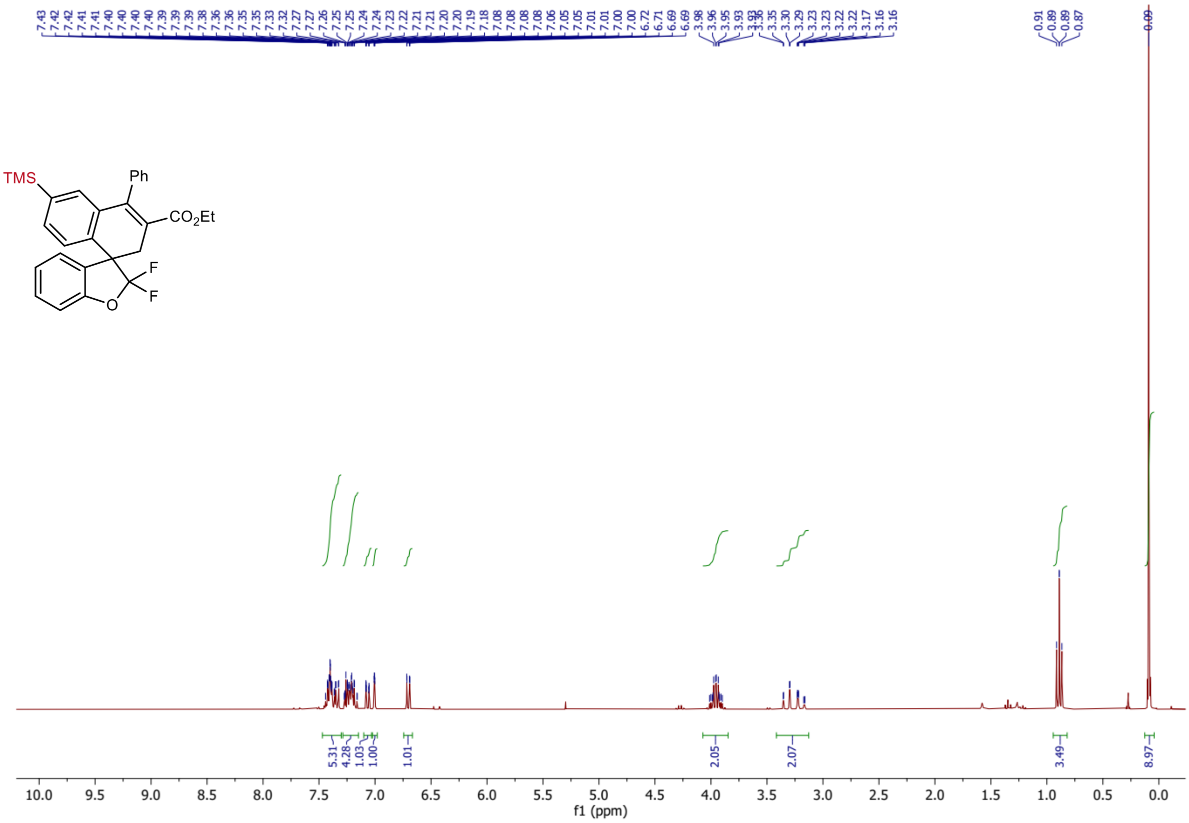

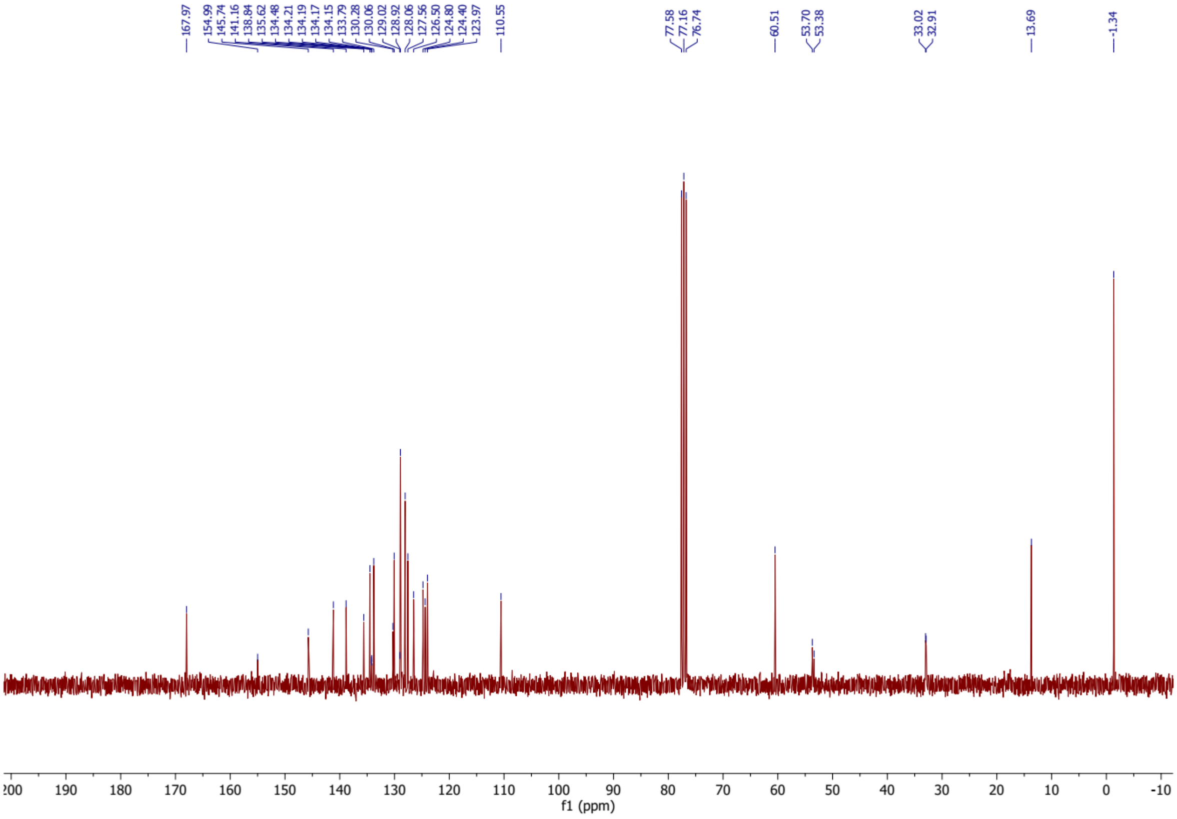

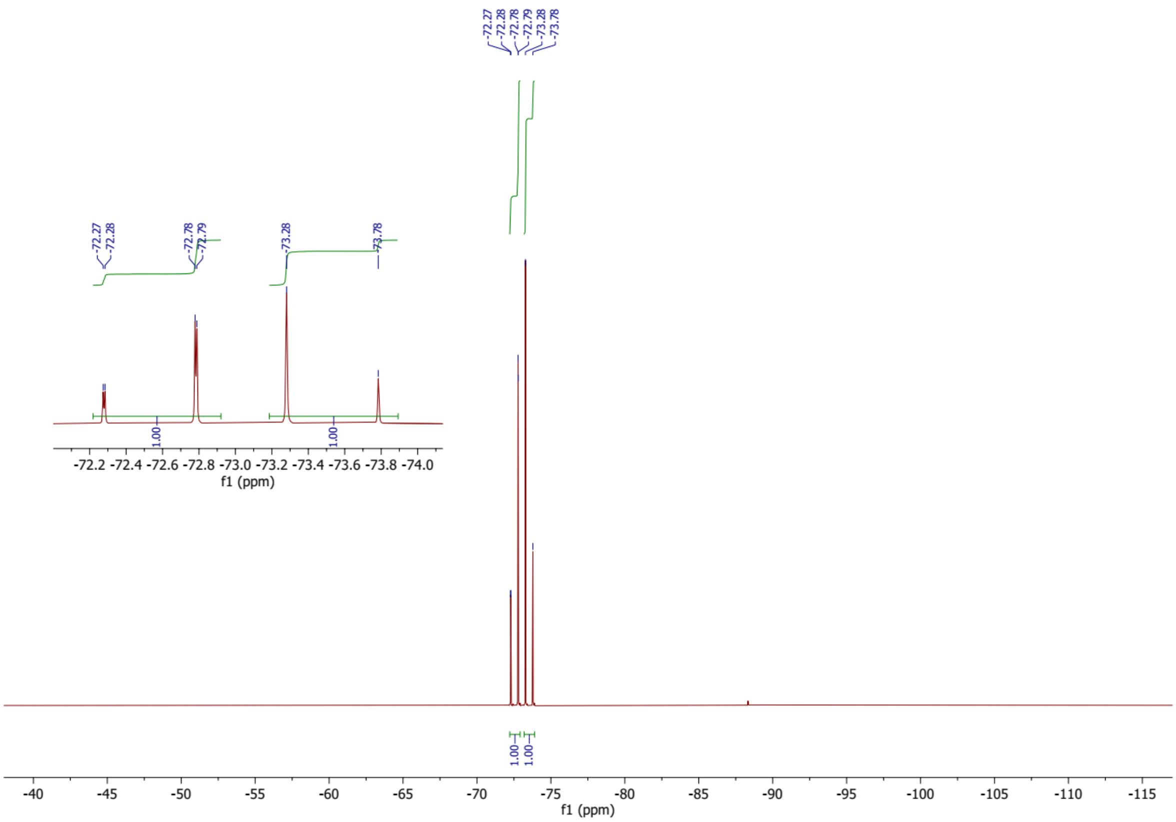

6g

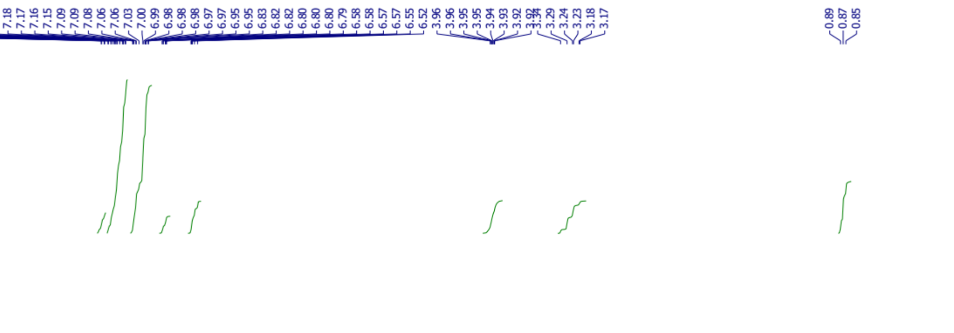

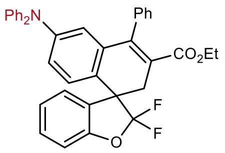

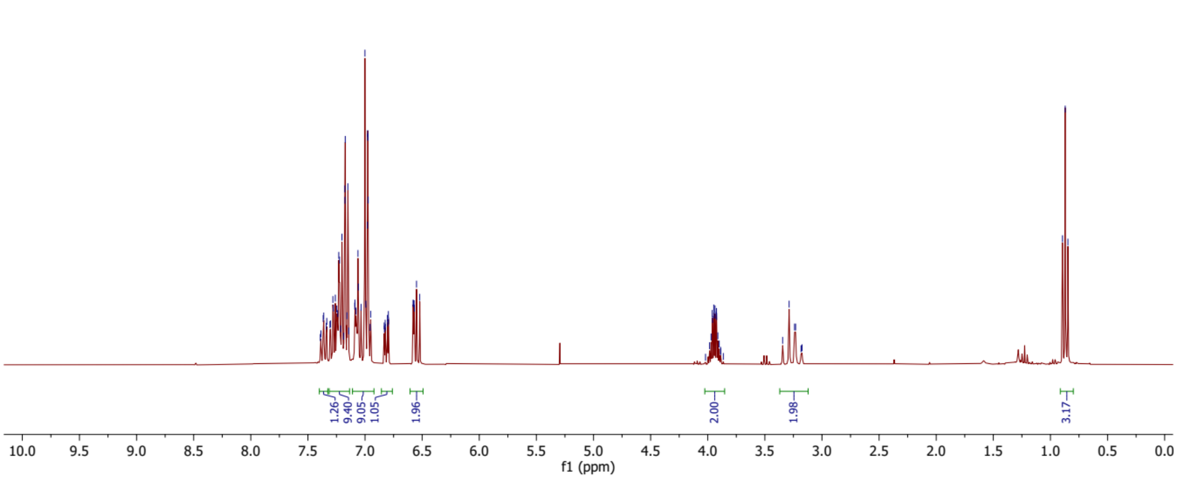

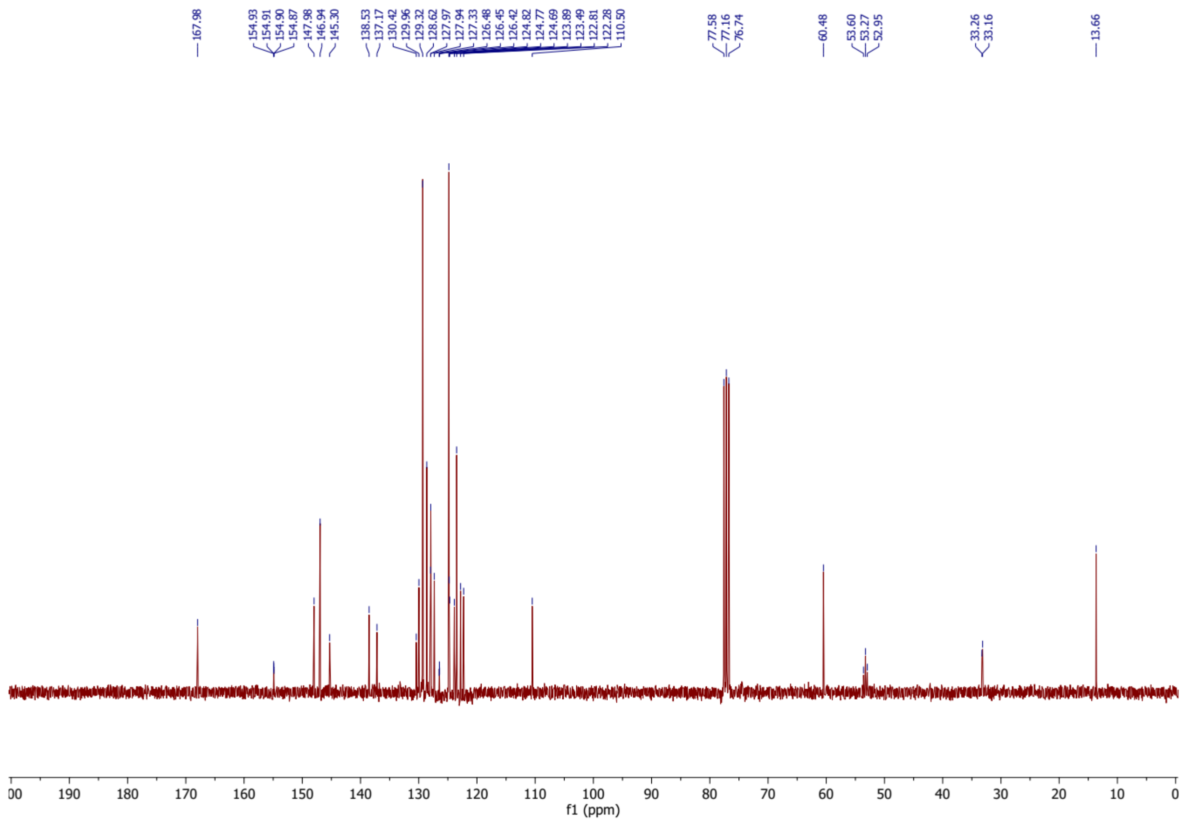
167.98
154.93
154.91
154.90
154.87
147.98
146.94
145.30
138.53
137.17
130.42
129.96
129.32
128.62
127.97
127.94
127.33
126.48
126.45
126.42
124.82
124.77
124.69
123.89
123.49
122.81
122.28
110.50
77.58
77.16
76.74
60.48
53.60
53.27
52.95
33.26
33.16
13.66
f1 (ppm)

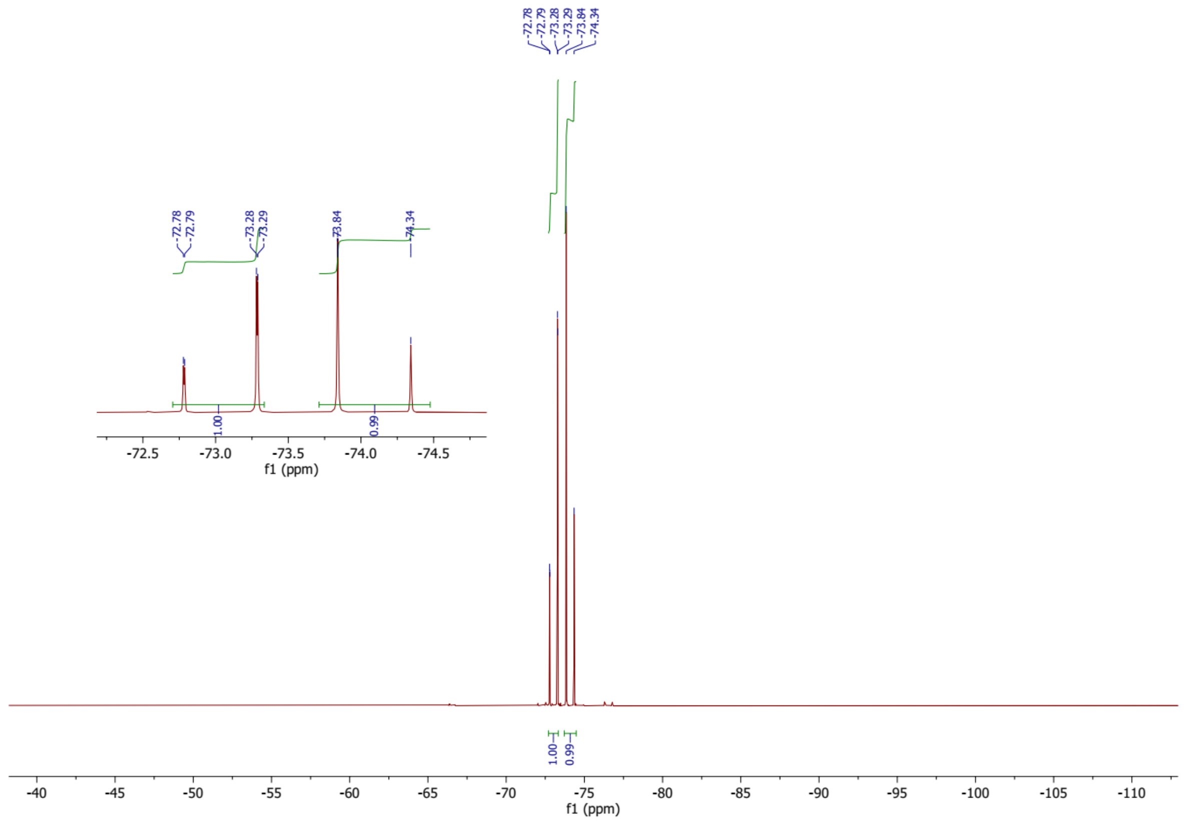
-72.78
-72.79
-73.28
-73.29
-73.84
-74.34
1.00
0.99
f1 (ppm)

6h

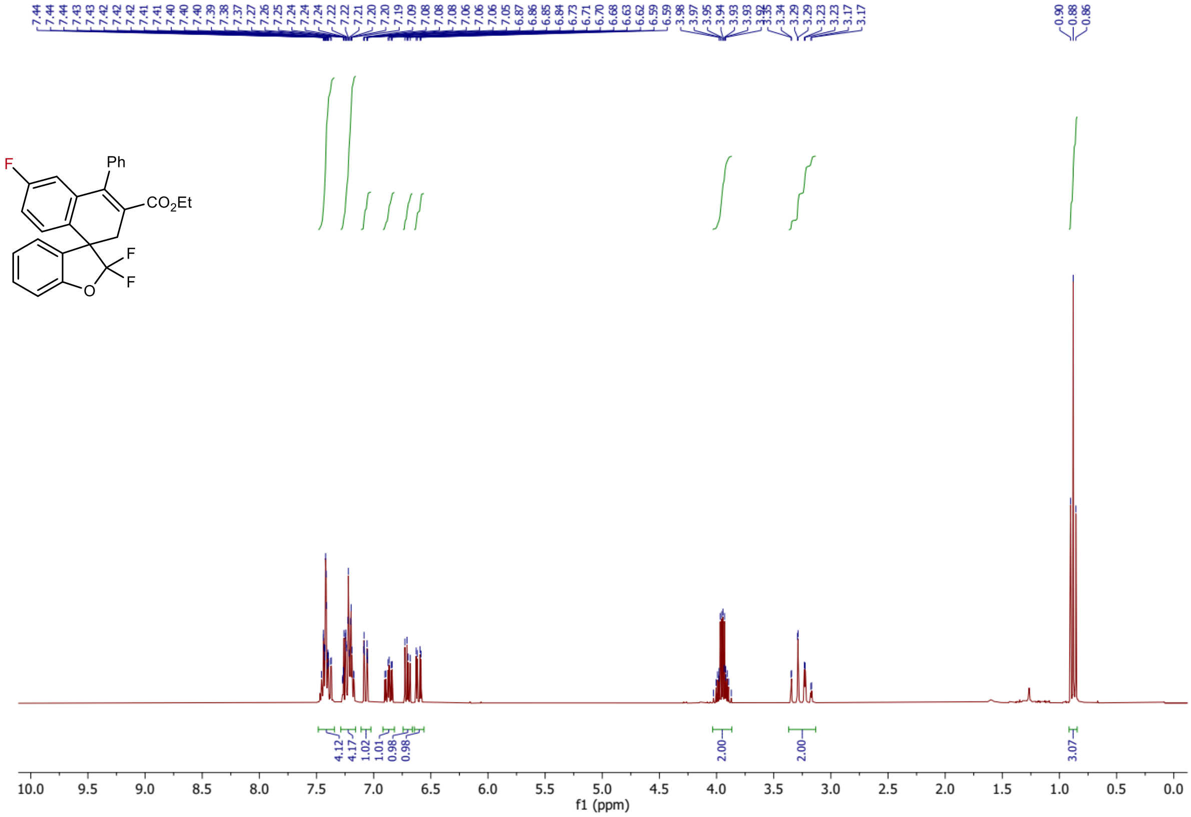
F
Ph
CO2Et
F
F
O
7.44
7.44
7.44
7.43
7.43
7.42
7.42
7.42
7.41
7.41
7.40
7.40
7.39
7.38
7.37
7.27
7.26
7.25
7.24
7.24
7.24
7.22
7.22
7.21
7.20
7.20
7.19
7.09
7.08
7.08
7.08
7.06
7.06
7.06
7.05
6.87
6.86
6.85
6.84
6.73
6.71
6.70
6.68
6.63
6.62
6.59
6.59
3.98
3.97
3.95
3.94
3.93
3.93
3.92
3.35
3.34
3.29
3.29
3.23
3.23
3.17
0.90
0.88
0.86
4.12
4.17
1.02
1.01
0.98
0.98
2.00
2.00
3.07
10.0 9.5 9.0 8.5 8.0 7.5 7.0 6.5 6.0 5.5 5.0 4.5 4.0 3.5 3.0 2.5 2.0 1.5 1.0 0.5 0.0
f1 (ppm)

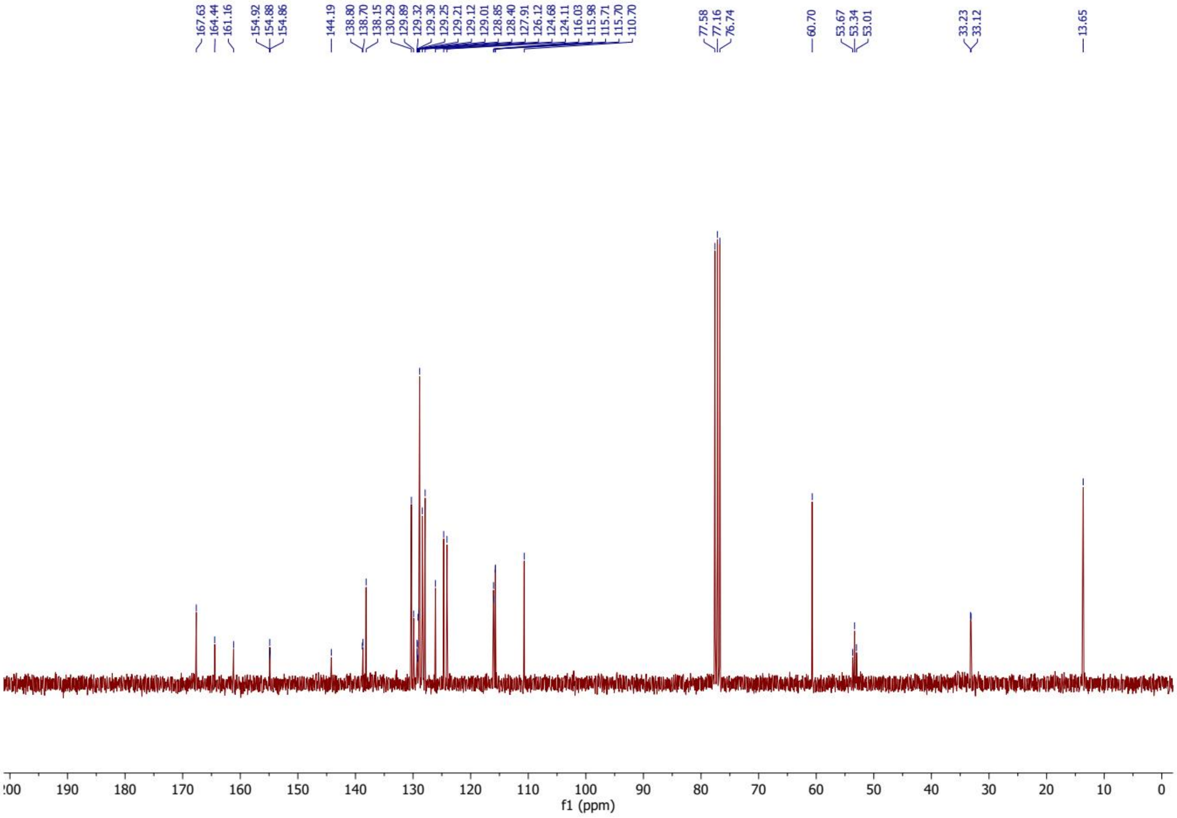
167.63
164.44
161.16
154.92
154.88
154.86
144.19
138.80
138.70
138.15
130.29
129.89
129.32
129.30
129.25
129.21
129.12
129.01
128.85
128.40
127.91
126.12
124.68
124.11
116.03
115.98
115.71
115.70
110.70
77.58
77.16
76.74
60.70
53.67
53.34
53.01
33.23
33.12
13.65
200 190 180 170 160 150 140 130 120 110 100 90 80 70 60 50 40 30 20 10 0
f1 (ppm)

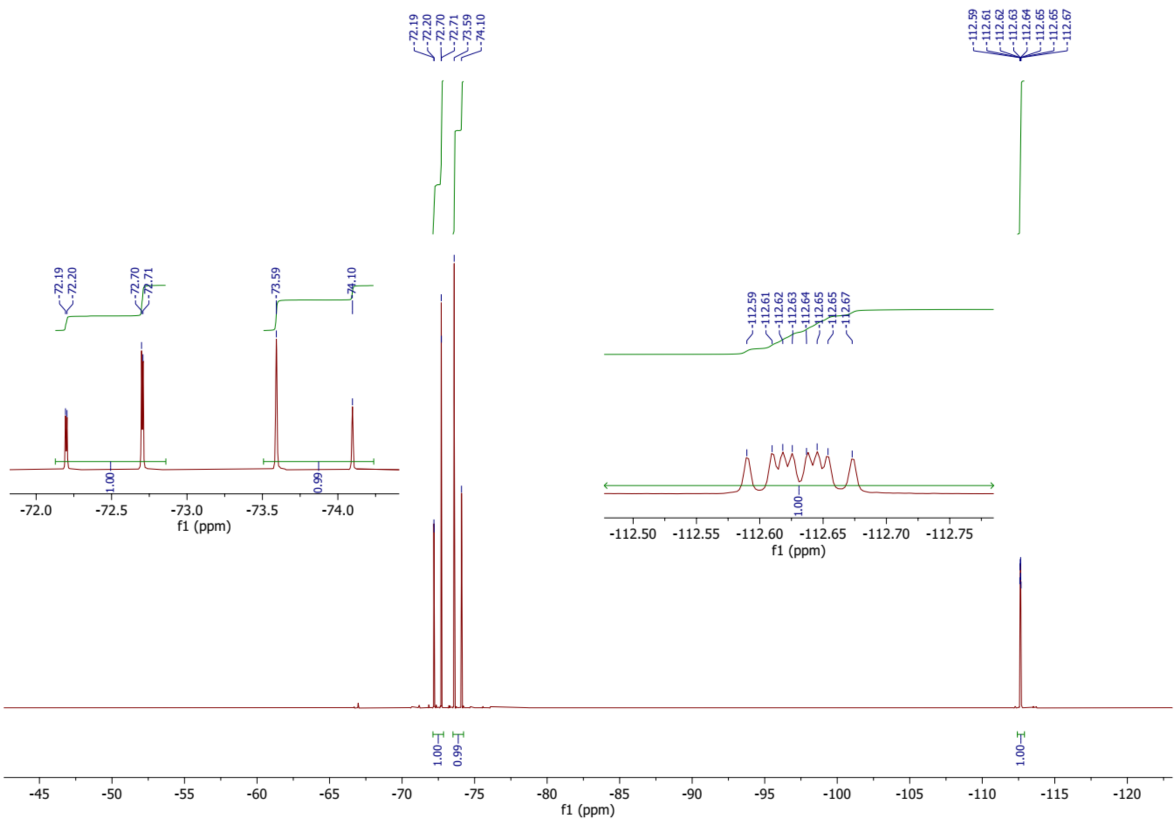

6i

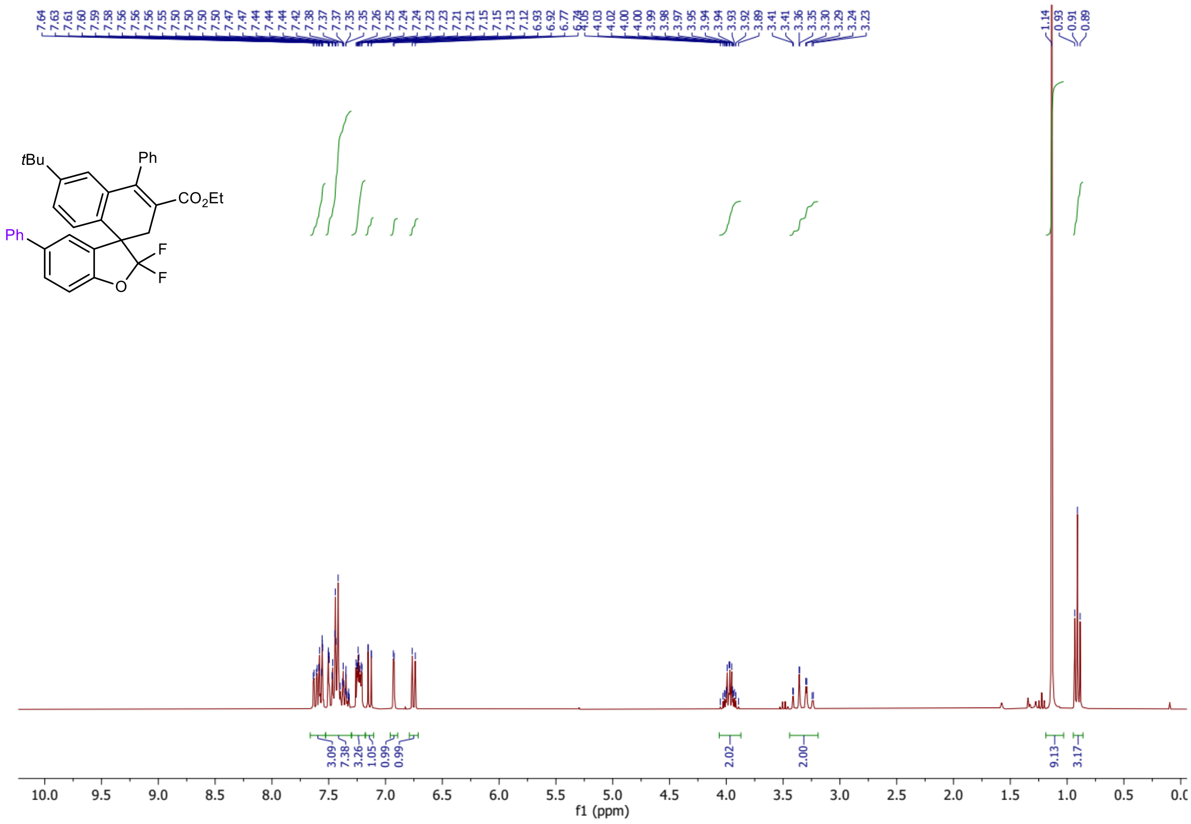

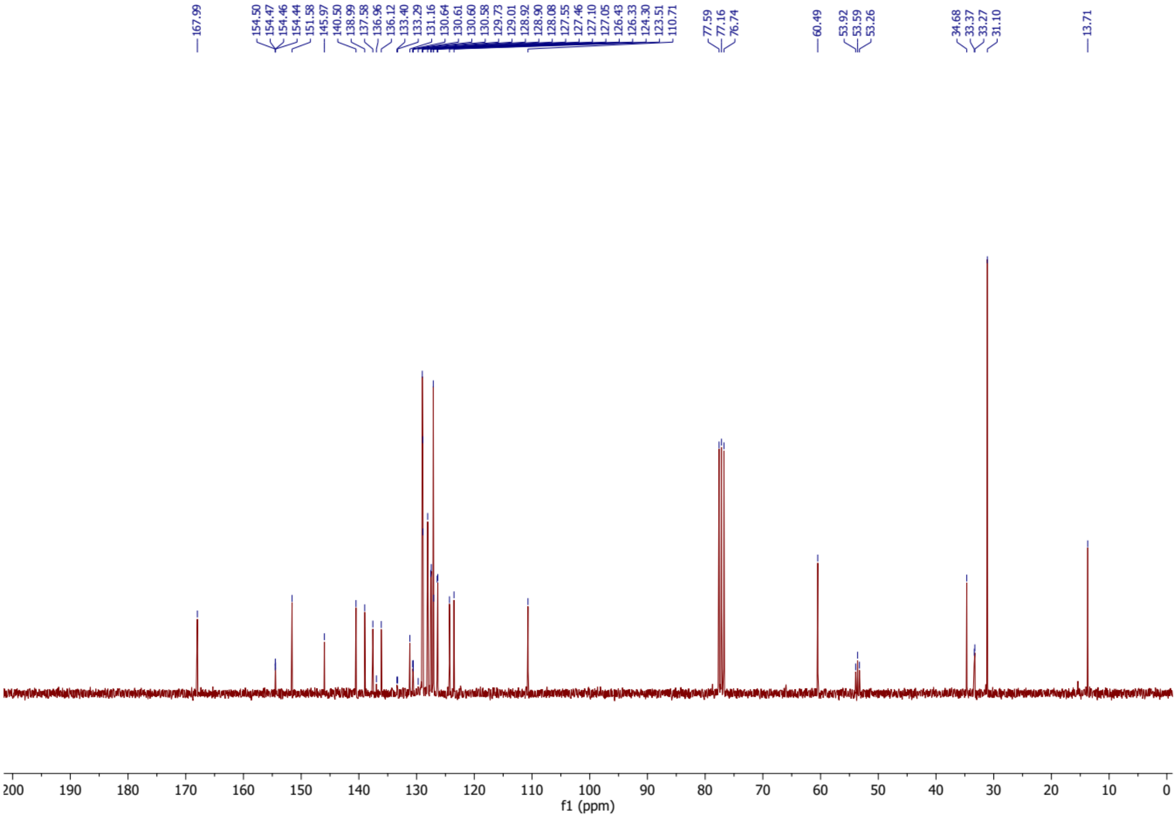
167.99
154.50
154.47
154.46
154.44
151.58
145.97
140.50
138.99
137.58
136.96
136.12
133.40
133.29
131.16
130.64
130.61
130.60
130.58
129.73
129.01
128.92
128.90
128.08
127.55
127.46
127.10
127.05
126.43
126.33
124.30
123.51
110.71
77.59
77.16
76.74
60.49
53.92
53.59
53.26
34.68
33.37
33.27
31.10
13.71
f1 (ppm)

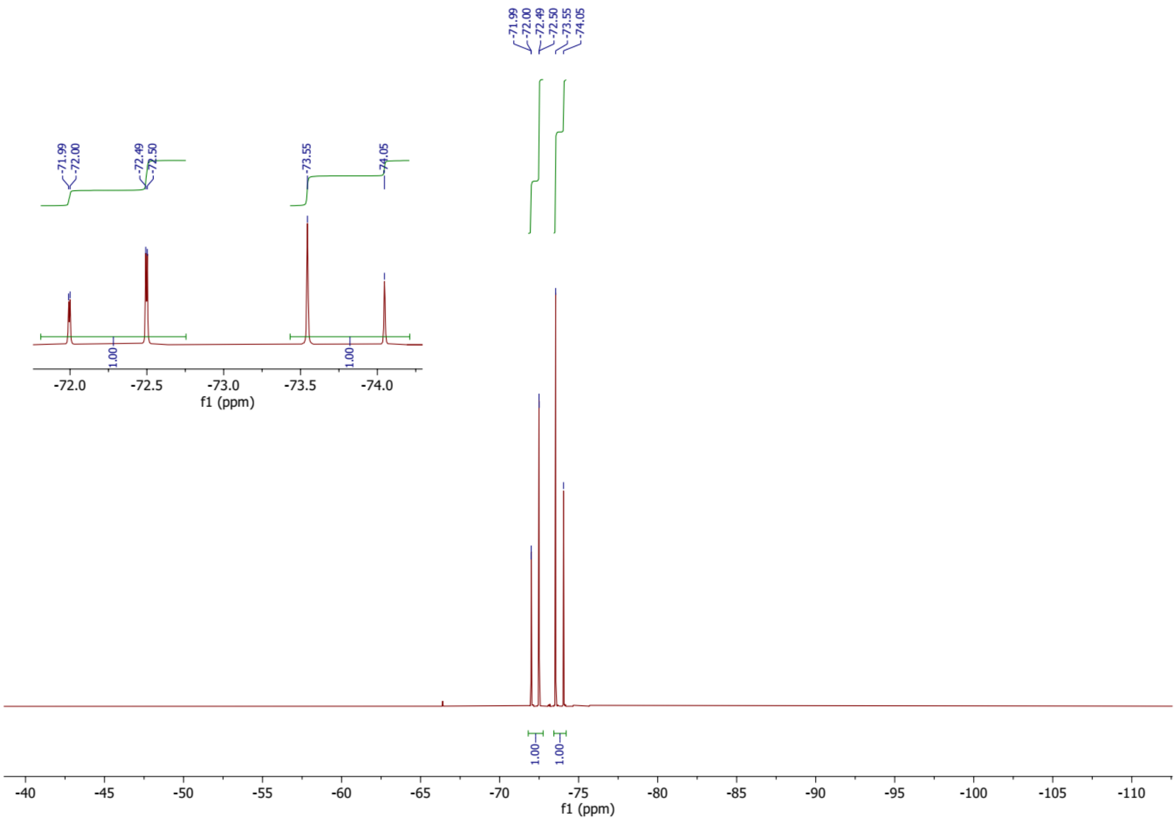
-71.99
-72.00
-72.49
-72.50
-73.55
-74.05
1.00
1.00
f1 (ppm)

6j

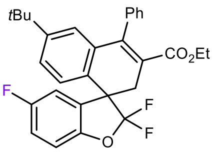
tBu
Ph
CO2Et
F
F
F
O

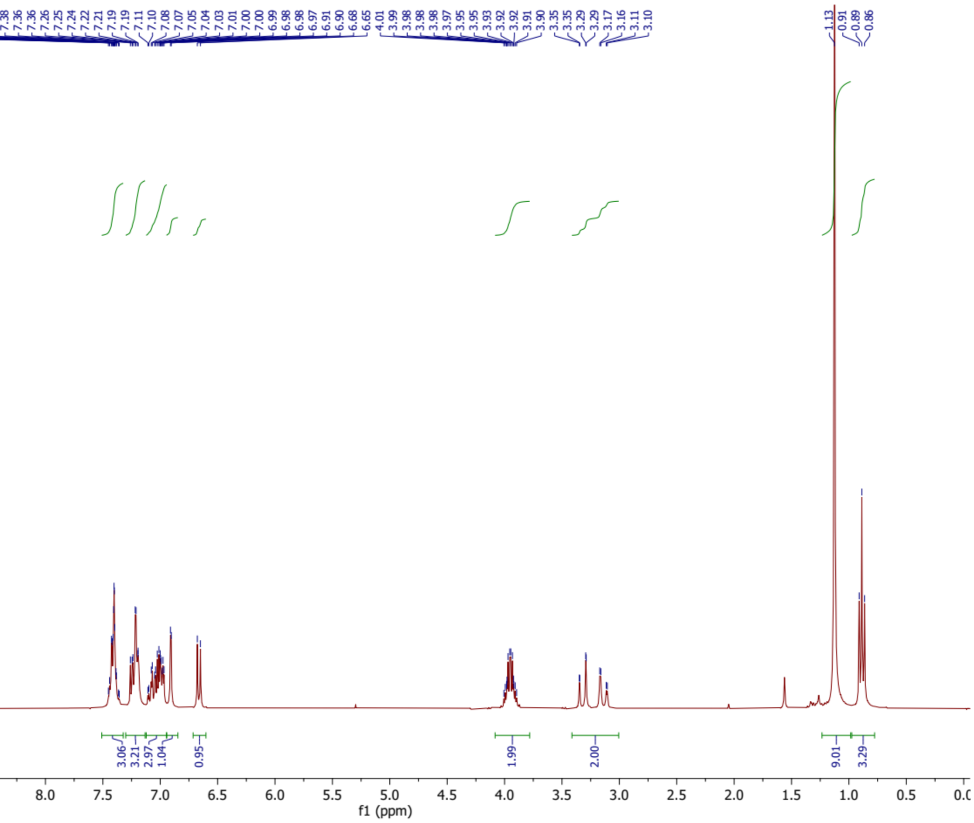
7.45 7.44 7.42 7.42 7.41 7.41 7.40 7.40 7.40 7.39 7.38 7.36 7.36 7.26 7.25 7.24 7.22 7.21 7.19 7.19 7.11 7.10 7.08 7.07 7.05 7.04 7.03 7.01 7.00 6.99 6.98 6.98 6.97 6.91 6.90 6.68 6.65 4.01 3.99 3.98 3.98 3.98 3.97 3.95 3.95 3.93 3.92 3.92 3.91 3.90 3.35 3.35 3.29 3.29 3.17 3.16 3.11 3.10
1.13 0.91 0.89 0.86
3.06 3.21 2.97 1.04 0.95 1.99 2.00 9.01 3.29
10.0 9.5 9.0 8.5 8.0 7.5 7.0 6.5 6.0 5.5 5.0 4.5 4.0 3.5 3.0 2.5 2.0 1.5 1.0 0.5 0.0
f1 (ppm)

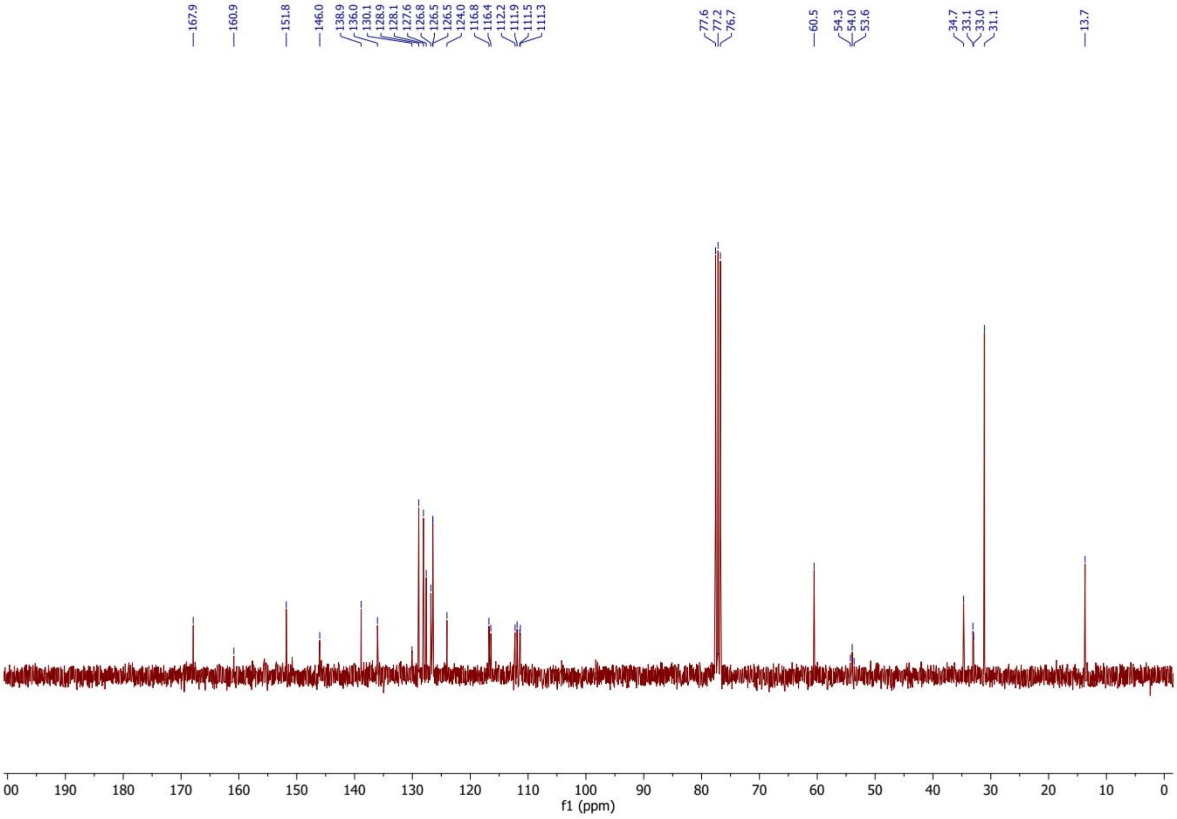
167.9
160.9
151.8
146.0
138.9 136.0 130.1 128.9 128.1 127.6 126.8 126.5 124.0 116.8 116.4 112.2 111.9 111.5 111.3
77.6 77.2 76.7
60.5
54.3 54.0 53.6
34.7 33.1 33.0 31.1
13.7
00 190 180 170 160 150 140 130 120 110 100 90 80 70 60 50 40 30 20 10 0
f1 (ppm)

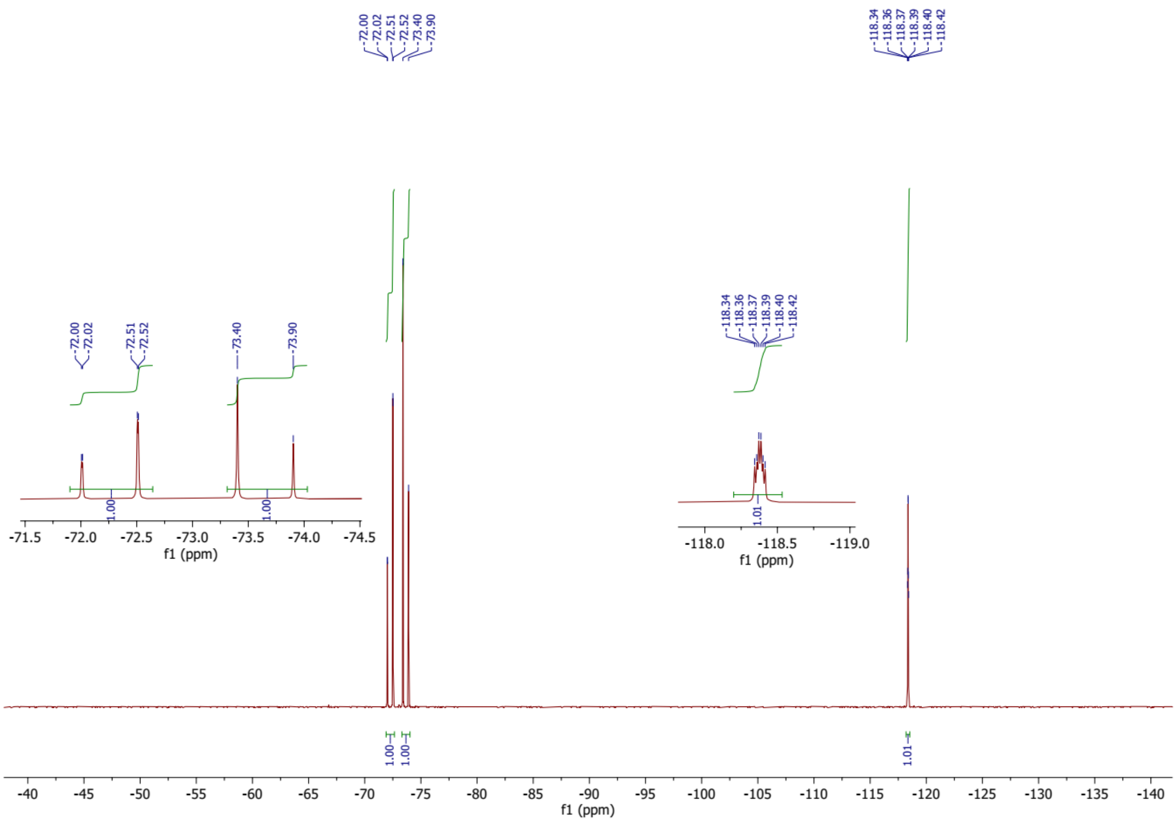
-72.00
-72.02
-72.51
-72.52
-73.40
-73.90
-118.34
-118.36
-118.37
-118.39
-118.40
-118.42
1.00
1.00
1.01
f1 (ppm)

6k

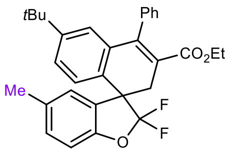
tBu
Ph
CO_2Et
Me
F
F
O

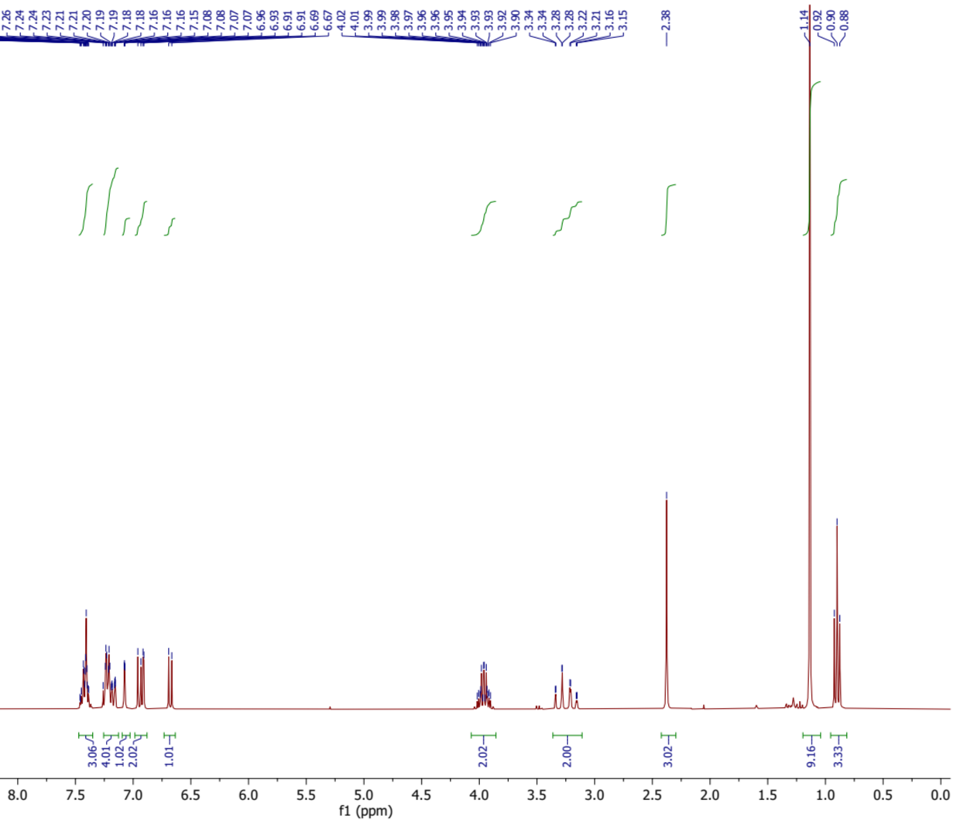
2.38
1.14
0.92
0.90
0.88
3.06
4.01
1.02
2.02
1.01
2.02
2.00
3.02
9.16
3.33
f1 (ppm)

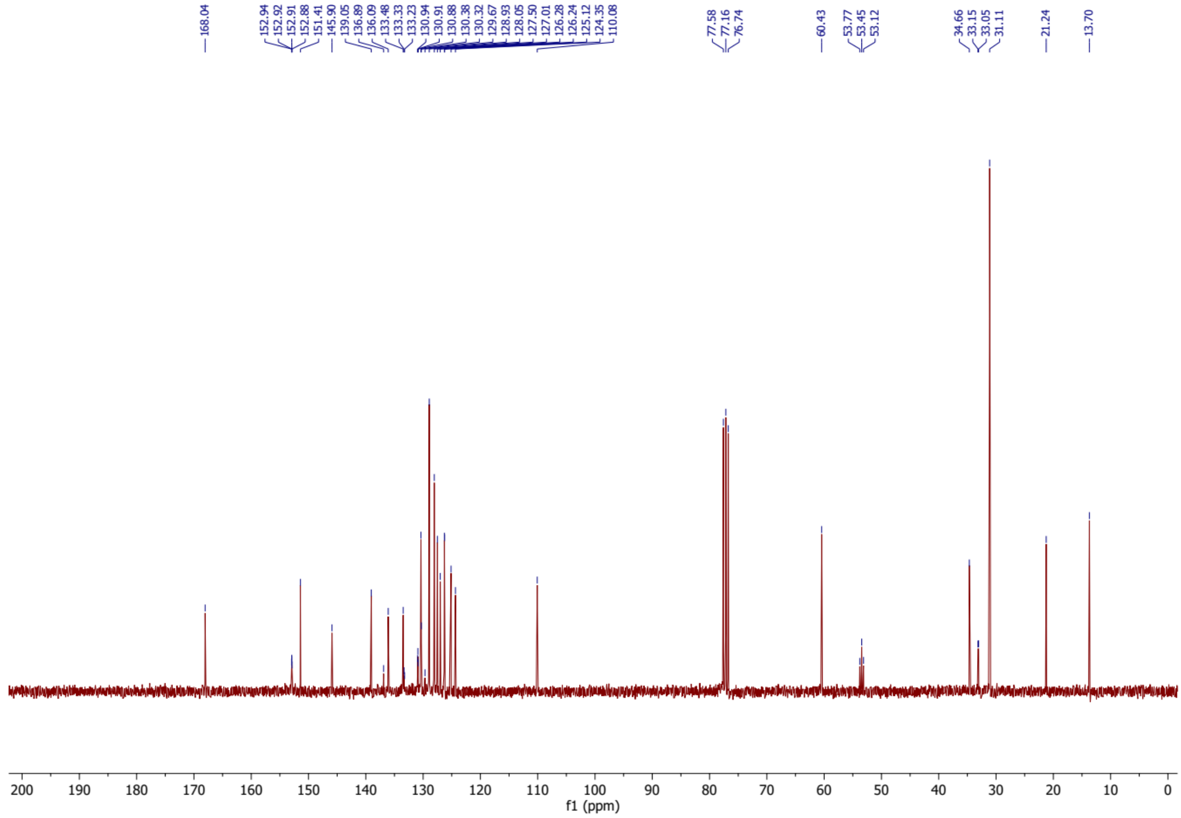
168.04
152.94
152.92
152.91
152.88
151.41
145.90
139.05
136.89
136.09
133.48
133.33
133.23
130.94
130.91
130.88
130.38
130.32
129.67
128.93
128.05
127.50
127.01
126.28
126.24
125.12
124.35
110.08
77.58
77.16
76.74
60.43
53.77
53.45
53.12
34.66
33.15
33.05
31.11
21.24
13.70
200 190 180 170 160 150 140 130 120 110 100 90 80 70 60 50 40 30 20 10 0
f1 (ppm)

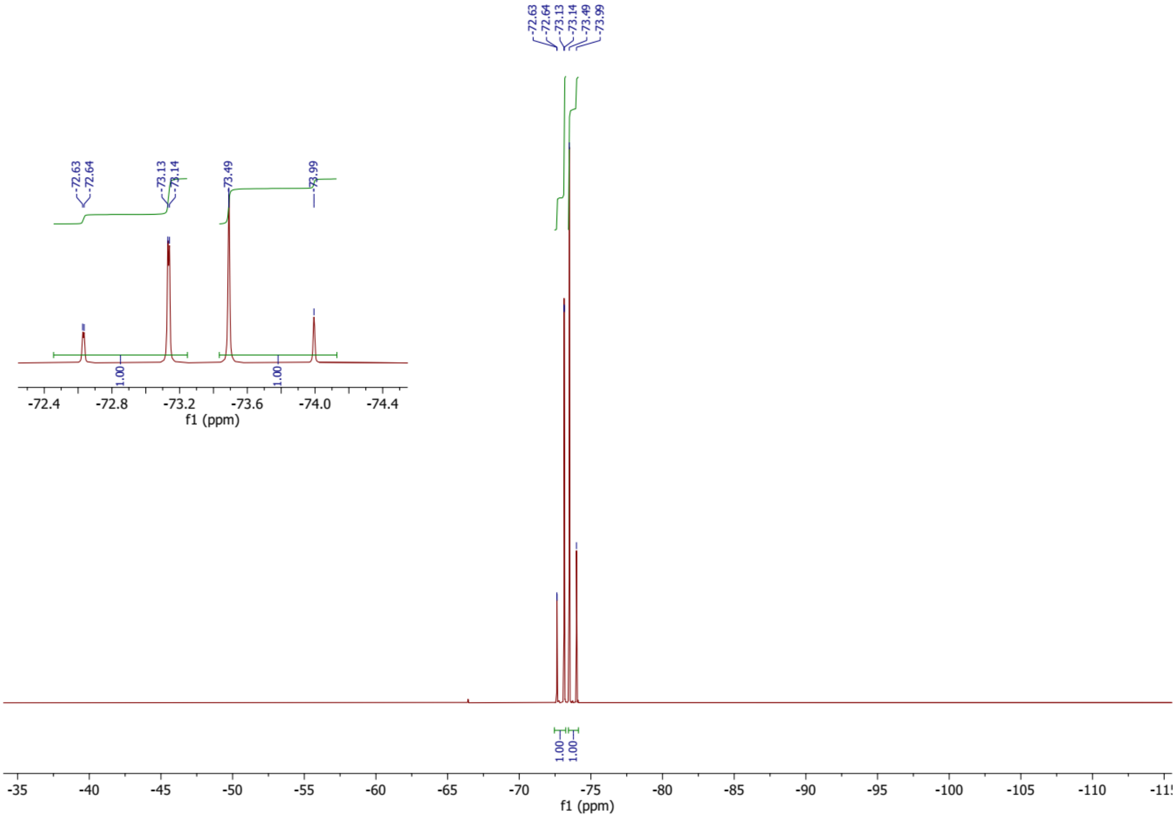
-72.63
-72.64
-73.13
-73.14
-73.49
-73.99
1.00
1.00
-72.4 -72.8 -73.2 -73.6 -74.0 -74.4
f1 (ppm)
-35 -40 -45 -50 -55 -60 -65 -70 -75 -80 -85 -90 -95 -100 -105 -110
f1 (ppm)

6l

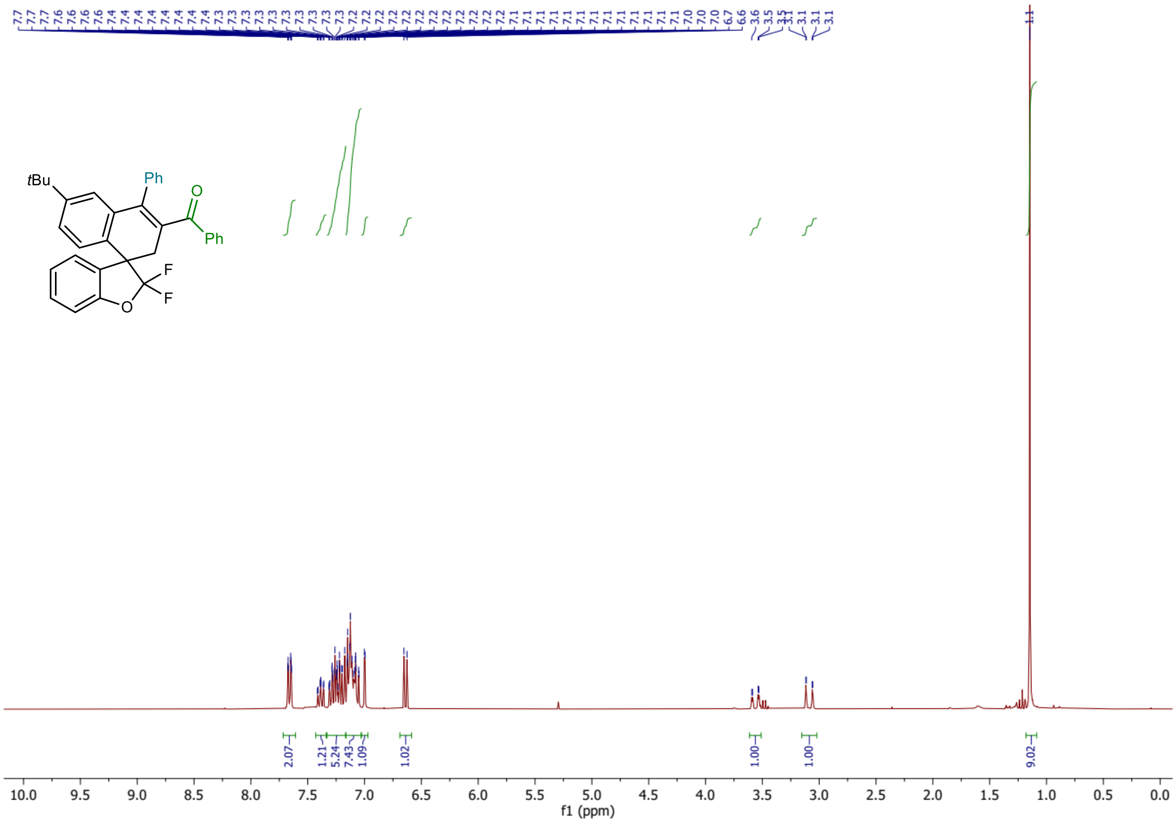

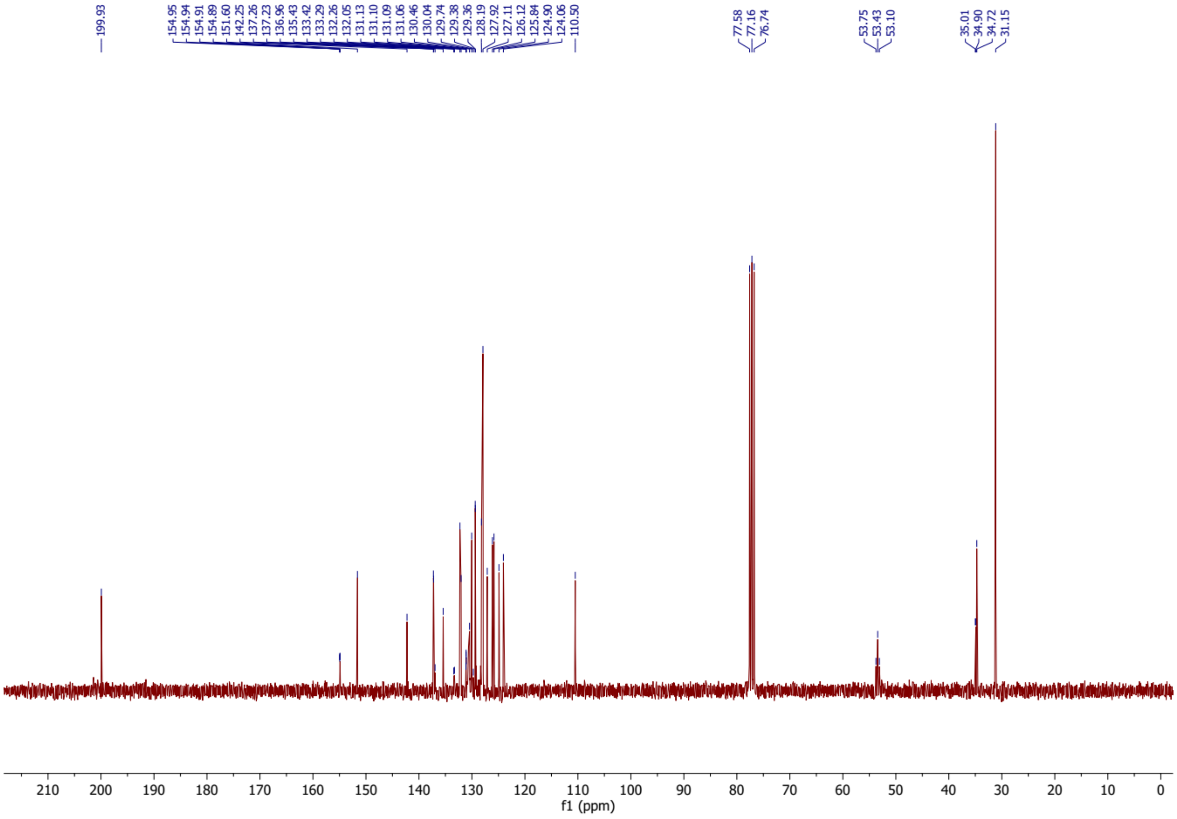

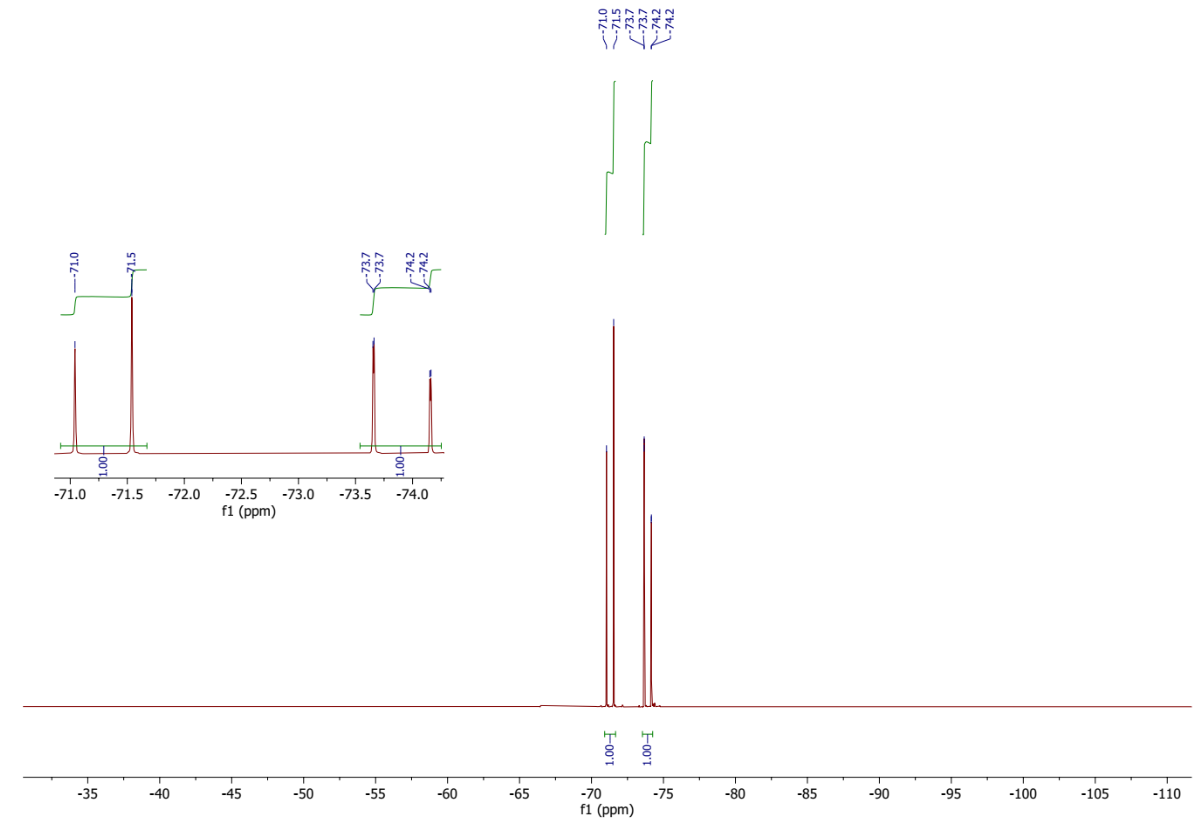
-71.0
-71.5
-73.7
-73.7
-74.2
-74.2
1.00
1.00
f1 (ppm)

6m

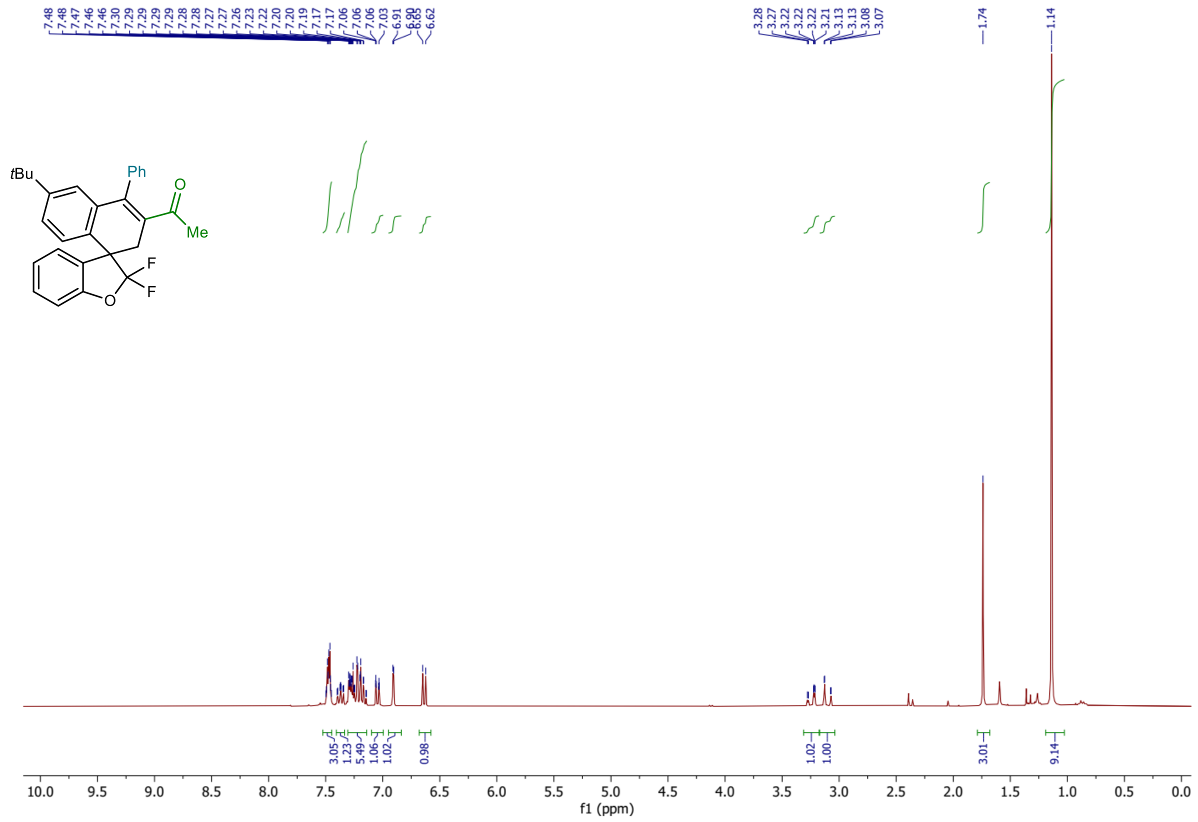
tBu
Ph
O
Me
F
F
O
7.48
7.48
7.47
7.46
7.46
7.30
7.29
7.29
7.29
7.28
7.28
7.27
7.27
7.26
7.23
7.22
7.22
7.20
7.20
7.19
7.17
7.17
7.06
7.06
7.03
6.91
6.65
6.62
3.28
3.27
3.22
3.22
3.22
3.21
3.13
3.13
3.08
3.07
1.74
1.14
3.05
1.23
5.49
1.06
1.02
0.98
1.02
1.00
3.01
9.14
f1 (ppm)

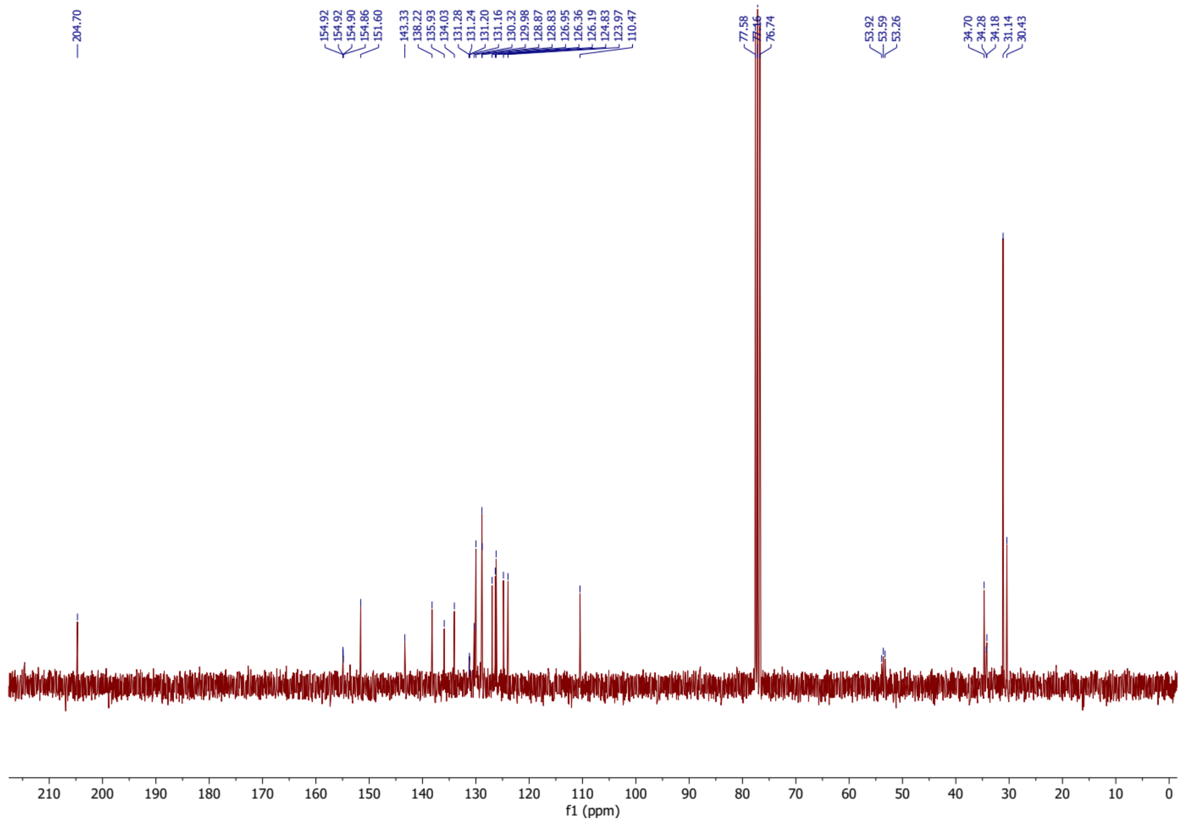
204.70
154.92
154.92
154.90
154.86
151.60
143.33
138.22
135.93
134.03
131.28
131.24
131.20
131.16
130.32
129.98
128.87
128.83
126.95
126.36
126.19
124.83
123.97
110.47
77.58
77.16
76.74
53.92
53.59
53.26
34.70
34.28
34.18
31.14
30.43
210 200 190 180 170 160 150 140 130 120 110 100 90 80 70 60 50 40 30 20 10 0
f1 (ppm)

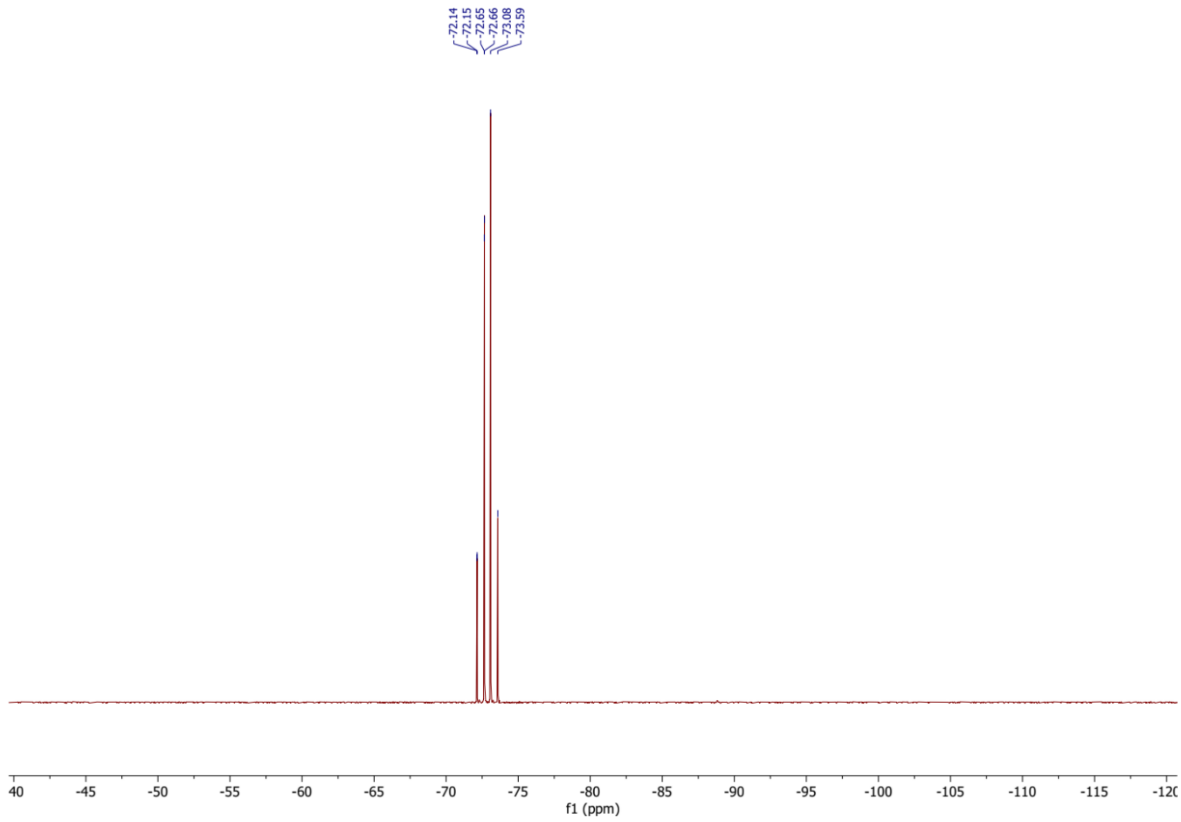
-72.14
-72.15
-72.65
-72.66
-73.08
-73.59
-40 -45 -50 -55 -60 -65 -70 -75 -80 -85 -90 -95 -100 -105 -110 -115 -120
f1 (ppm)

6n

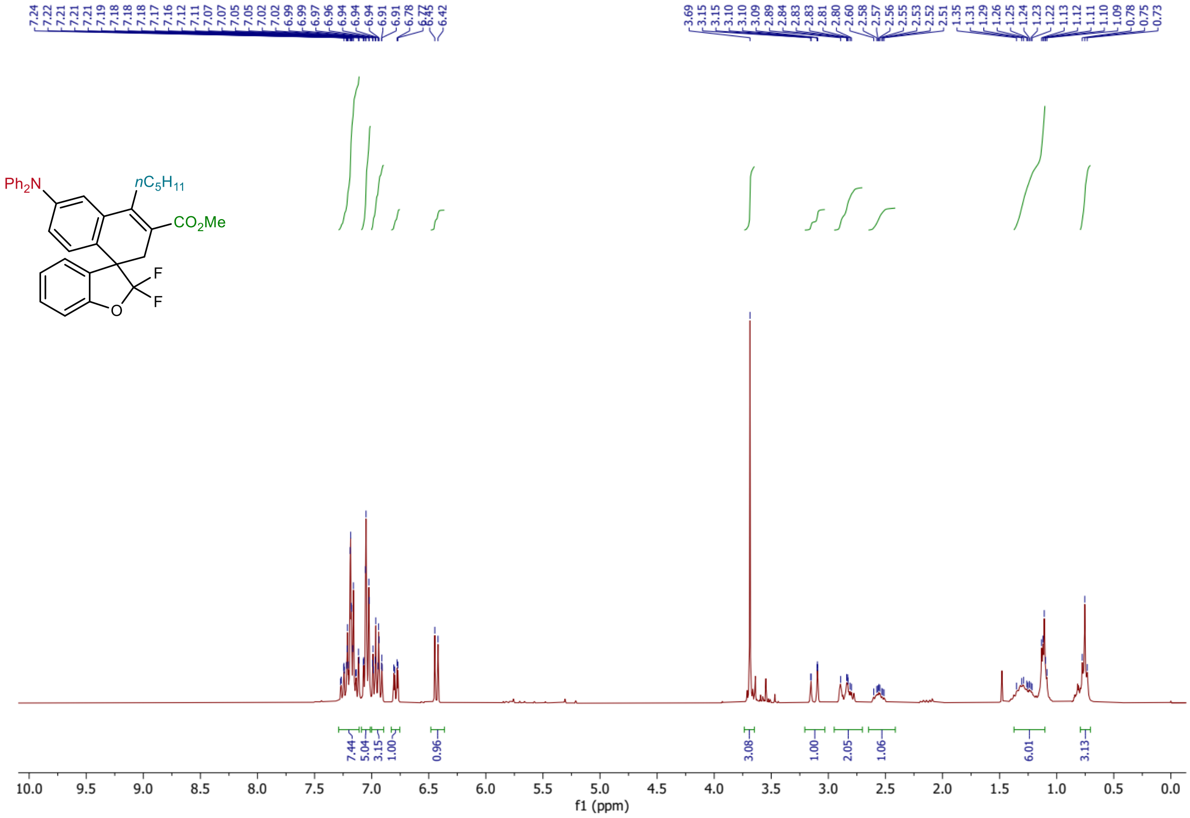

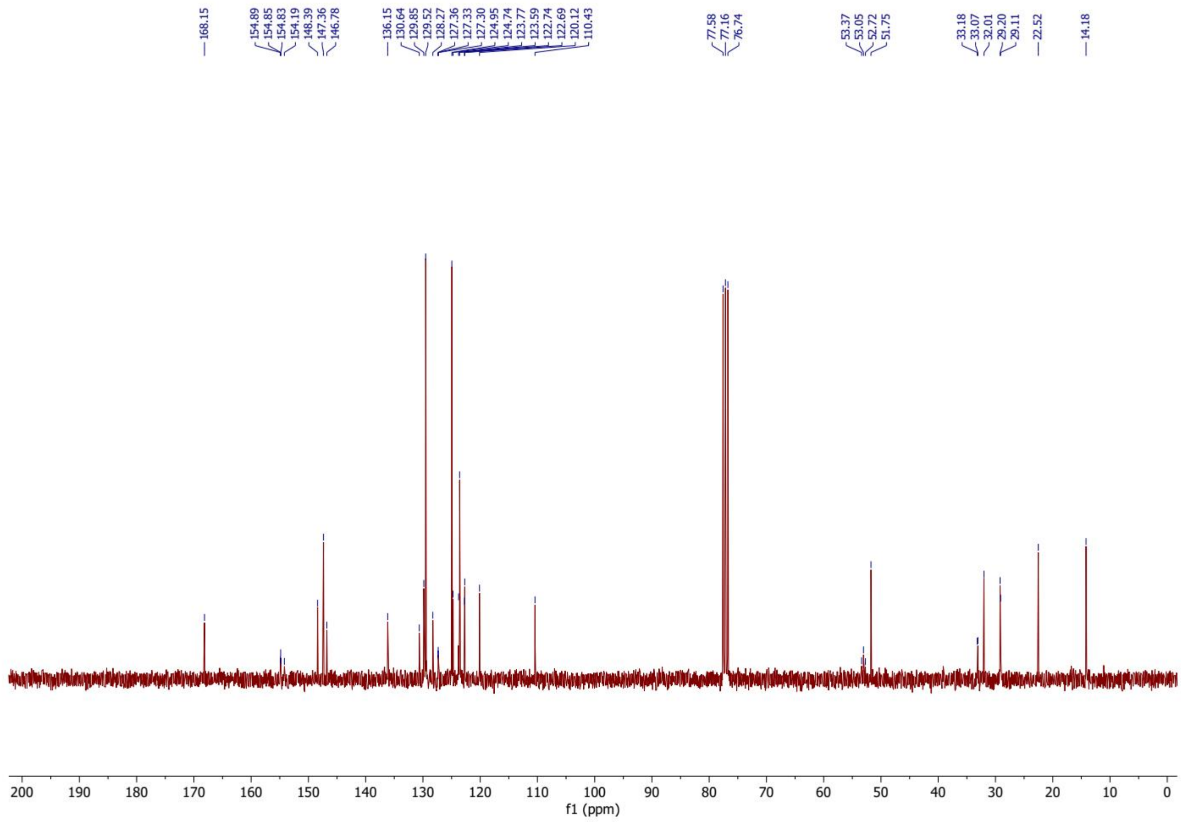

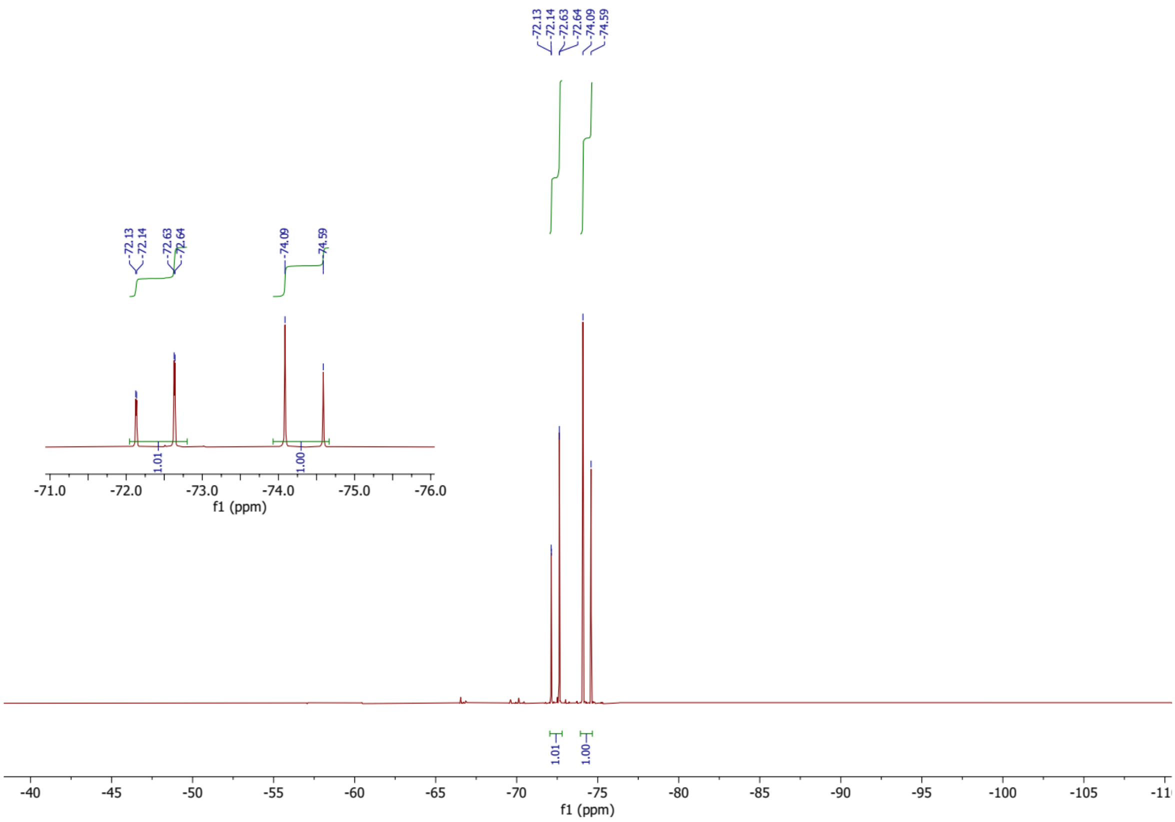

6o

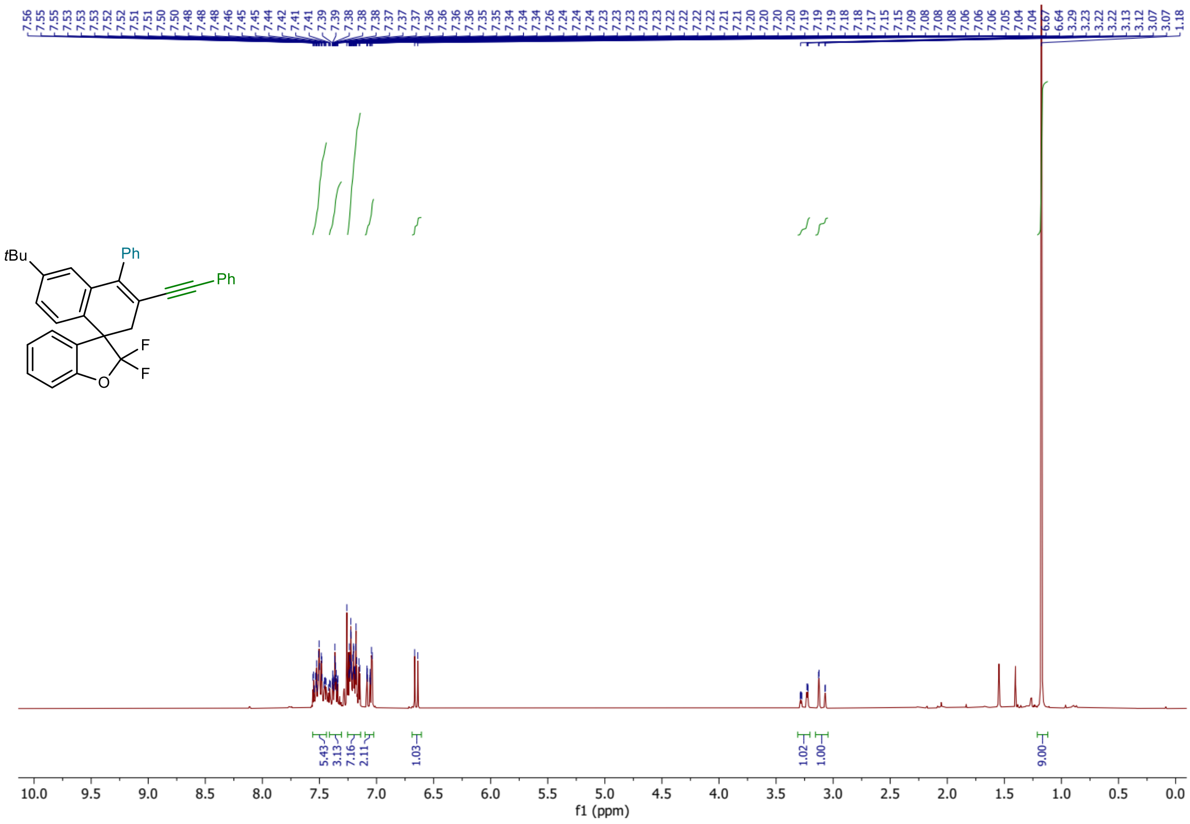

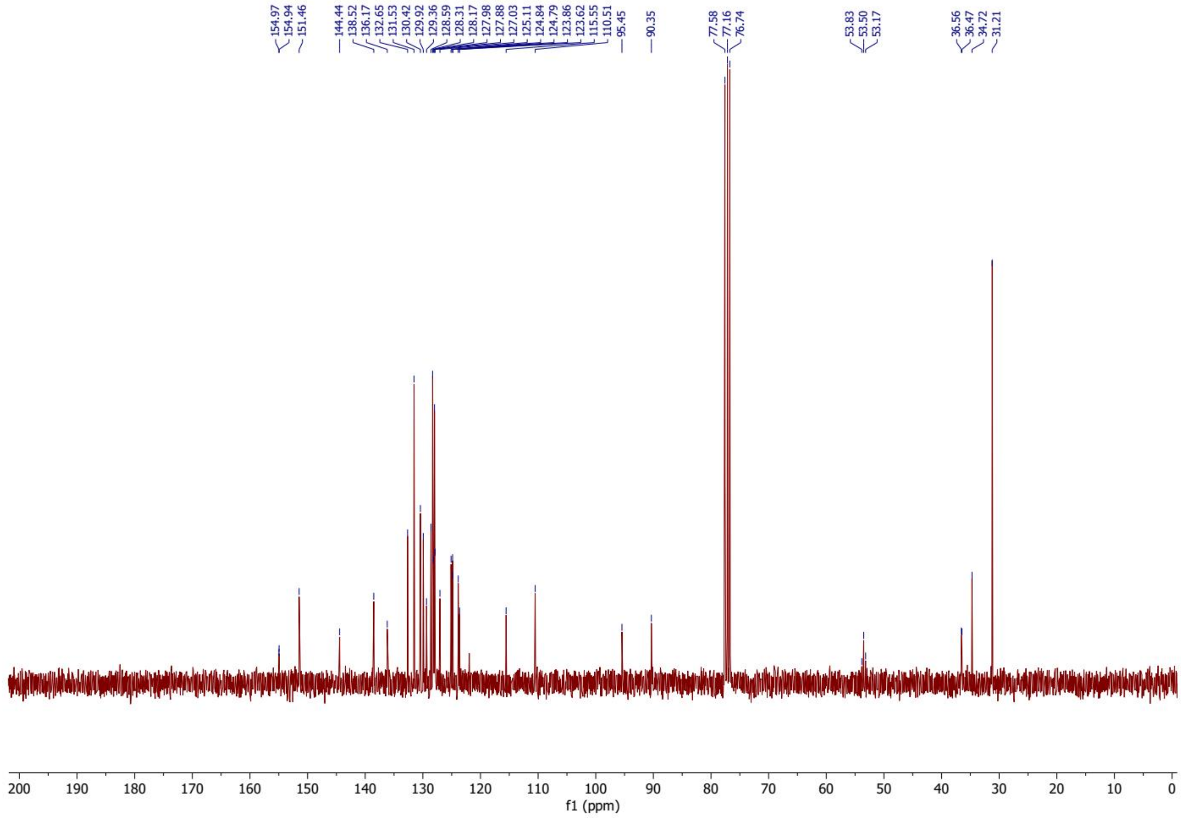
154.97
154.94
151.46
144.44
138.52
136.17
132.65
131.53
130.42
129.92
129.36
128.59
128.31
128.17
127.98
127.88
127.03
125.11
124.84
124.79
123.86
123.62
115.55
110.51
95.45
90.35
77.58
77.16
76.74
53.83
53.50
53.17
36.56
36.47
34.72
31.21
200
190
180
170
160
150
140
130
120
110
100
90
80
70
60
50
40
30
20
10
0
f1 (ppm)

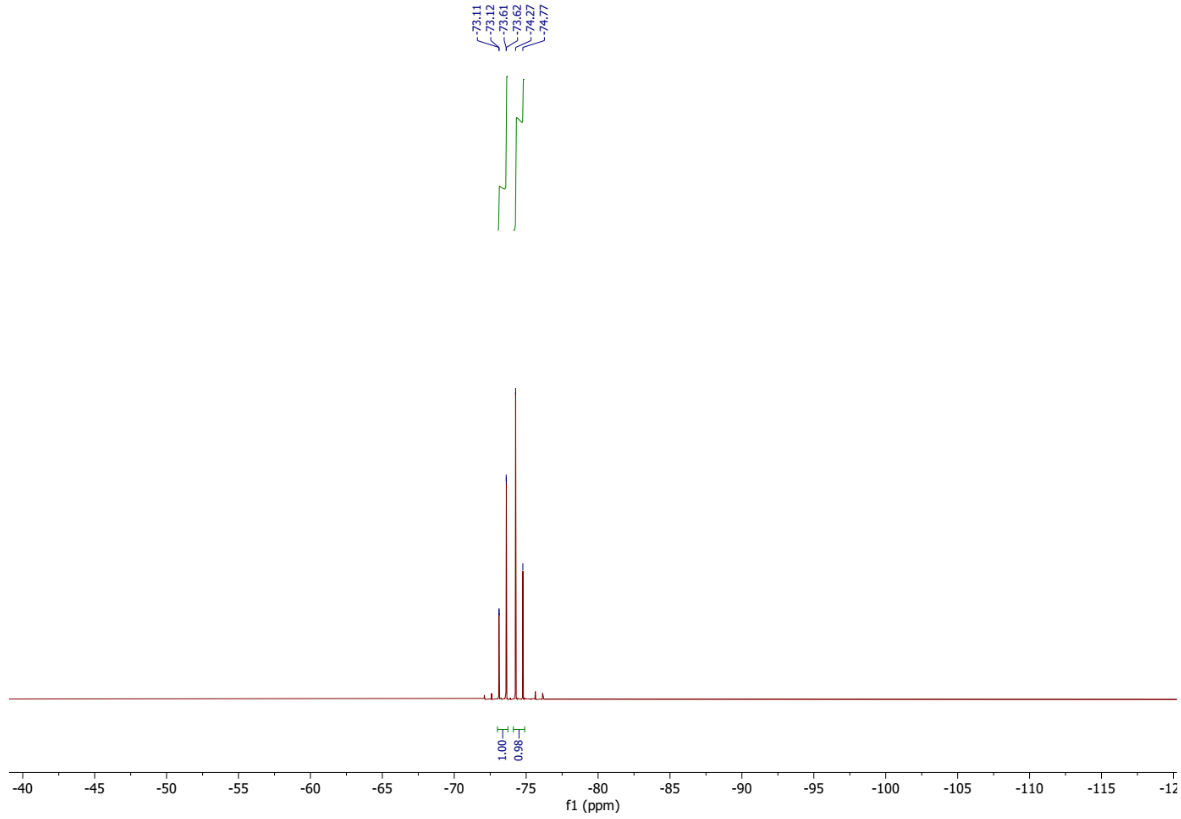
-73.11
-73.12
-73.61
-73.62
-74.27
-74.77
1.00
0.98
-40
-45
-50
-55
-60
-65
-70
-75
-80
-85
-90
-95
-100
-105
-110
-115
f1 (ppm)

6p

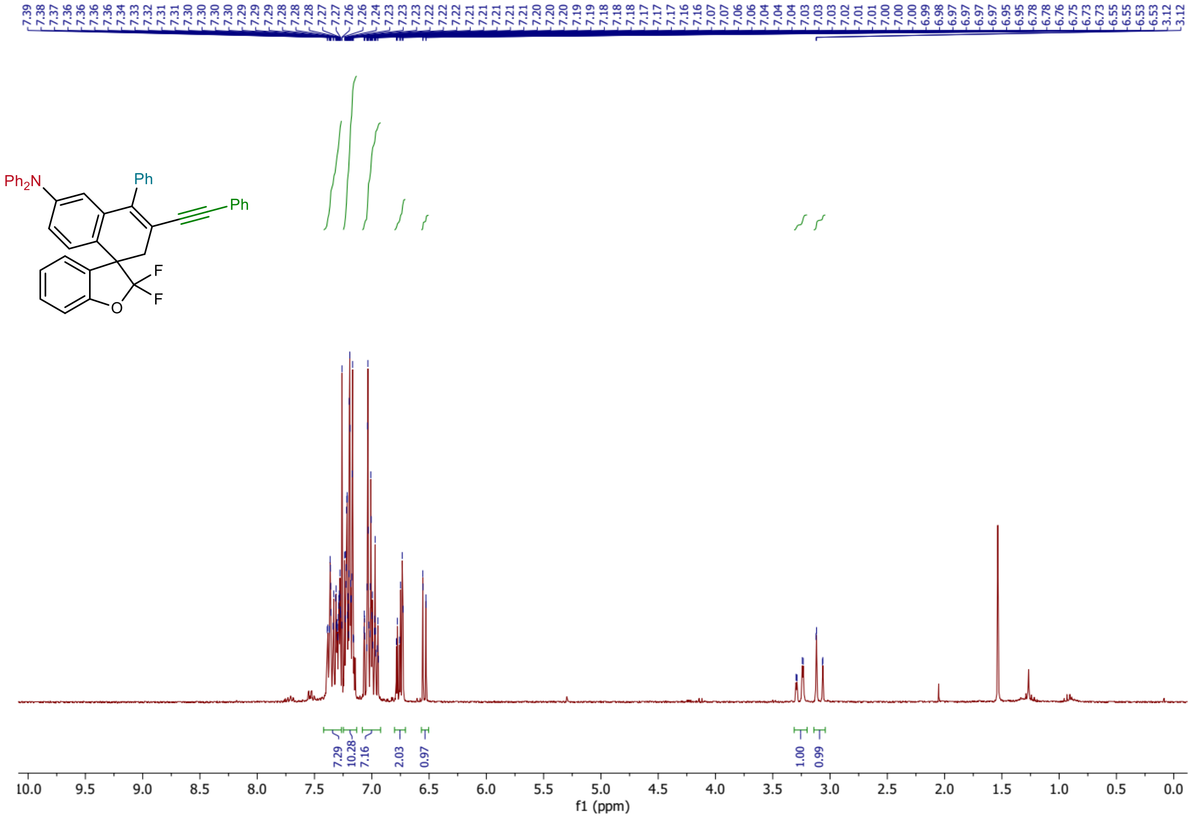
Ph2N
Ph
Ph
F
F
O
7.39 7.38 7.37 7.36 7.36 7.36 7.34 7.33 7.32 7.31 7.31 7.30 7.30 7.30 7.29 7.29 7.29 7.28 7.28 7.28 7.27 7.27 7.26 7.26 7.24 7.23 7.23 7.23 7.22 7.22 7.22 7.22 7.21 7.21 7.21 7.21 7.21 7.20 7.20 7.19 7.19 7.18 7.18 7.17 7.17 7.17 7.16 7.07 7.06 7.06 7.04 7.04 7.03 7.03 7.03 7.02 7.01 7.01 7.00 7.00 7.00 6.99 6.98 6.97 6.97 6.97 6.95 6.95 6.78 6.76 6.75 6.73 6.73 6.55 6.55 6.53 6.53 3.12 3.12
7.29
10.28
7.16
2.03
0.97
1.00
0.99
10.0 9.5 9.0 8.5 8.0 7.5 7.0 6.5 6.0 5.5 5.0 4.5 4.0 3.5 3.0 2.5 2.0 1.5 1.0 0.5 0.0
f1 (ppm)

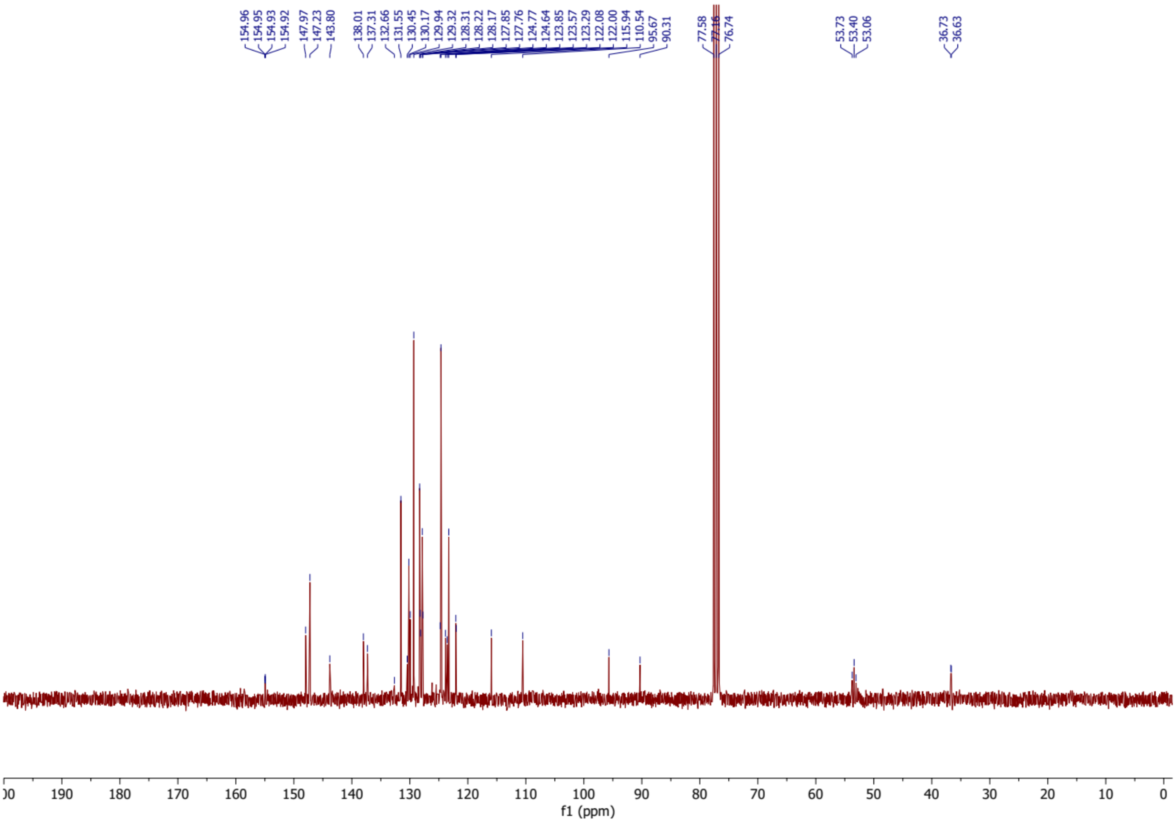
154.96 154.95 154.93 154.92 147.97 147.23 143.80 138.01 137.31 132.66 131.55 130.45 130.17 129.94 129.32 128.31 128.22 128.17 127.85 127.76 124.77 124.64 123.85 123.57 123.29 122.08 122.00 115.94 110.54 95.67 90.31 77.58 77.16 76.74 53.73 53.40 53.06 36.73 36.63
190 180 170 160 150 140 130 120 110 100 90 80 70 60 50 40 30 20 10 0
f1 (ppm)

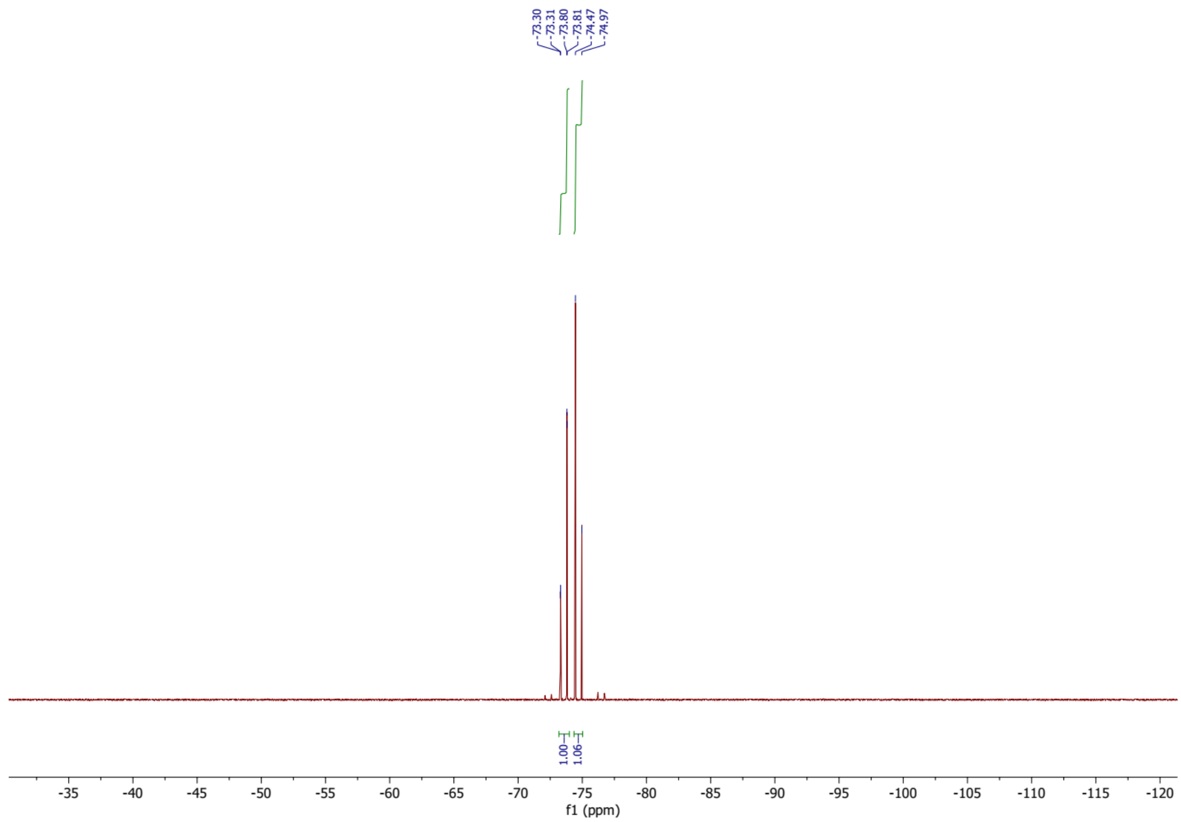

6q

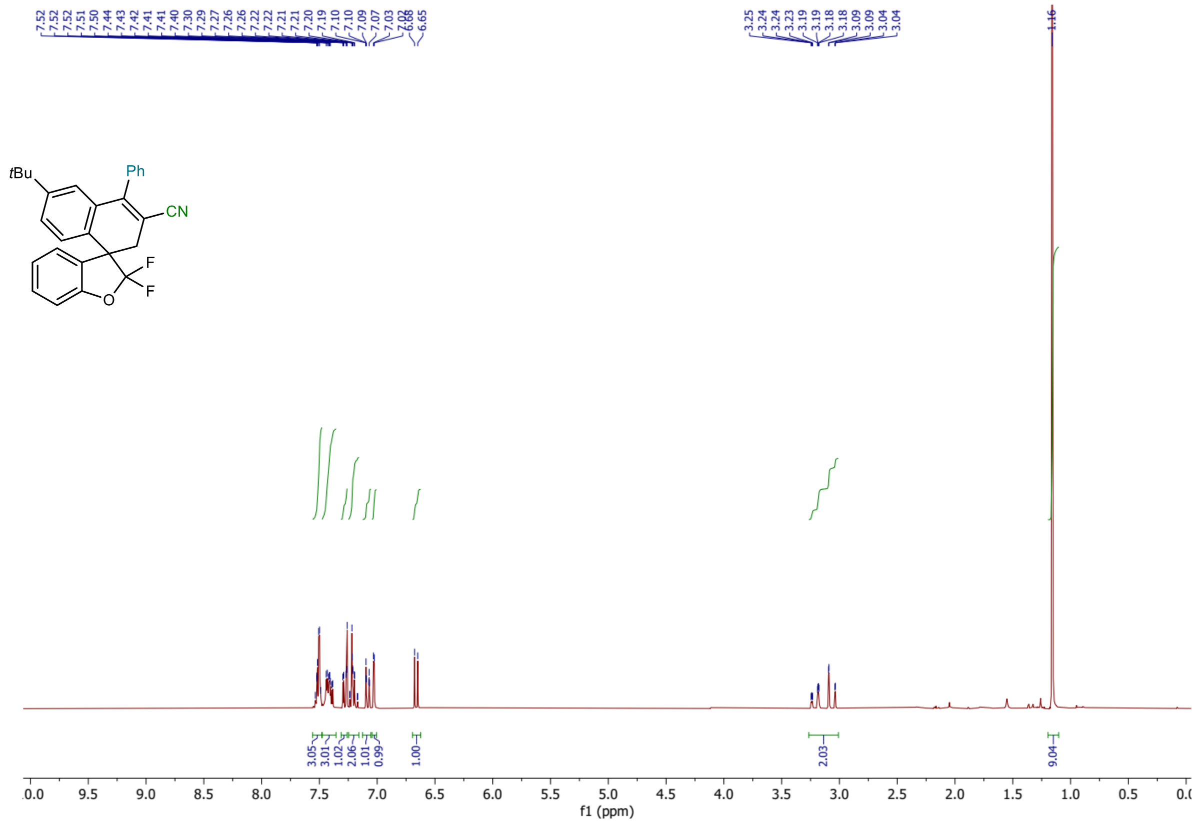

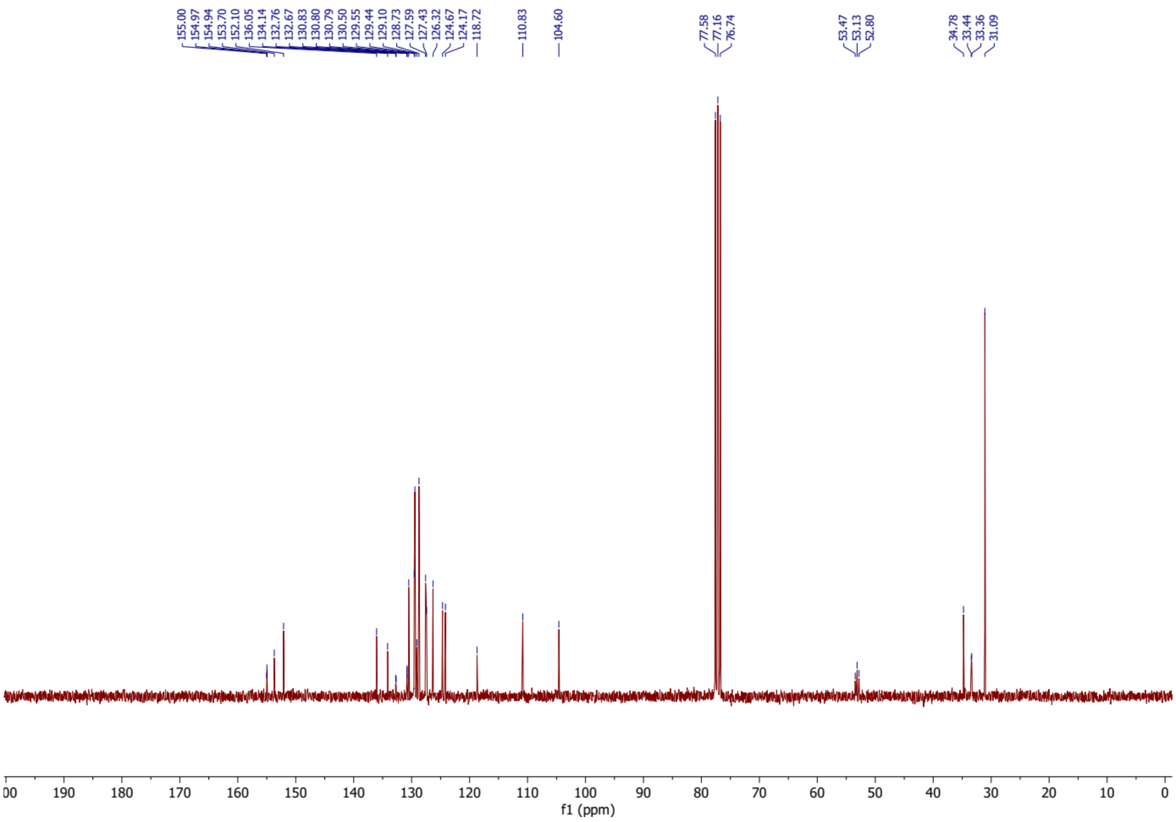
155.00
154.97
154.94
153.70
152.10
136.05
134.14
132.76
132.67
130.83
130.80
130.79
130.50
129.55
129.44
129.10
128.73
127.59
127.43
126.32
124.67
124.17
118.72
110.83
104.60
77.58
77.16
76.74
53.47
53.13
52.80
34.78
33.44
33.36
31.09
f1 (ppm)

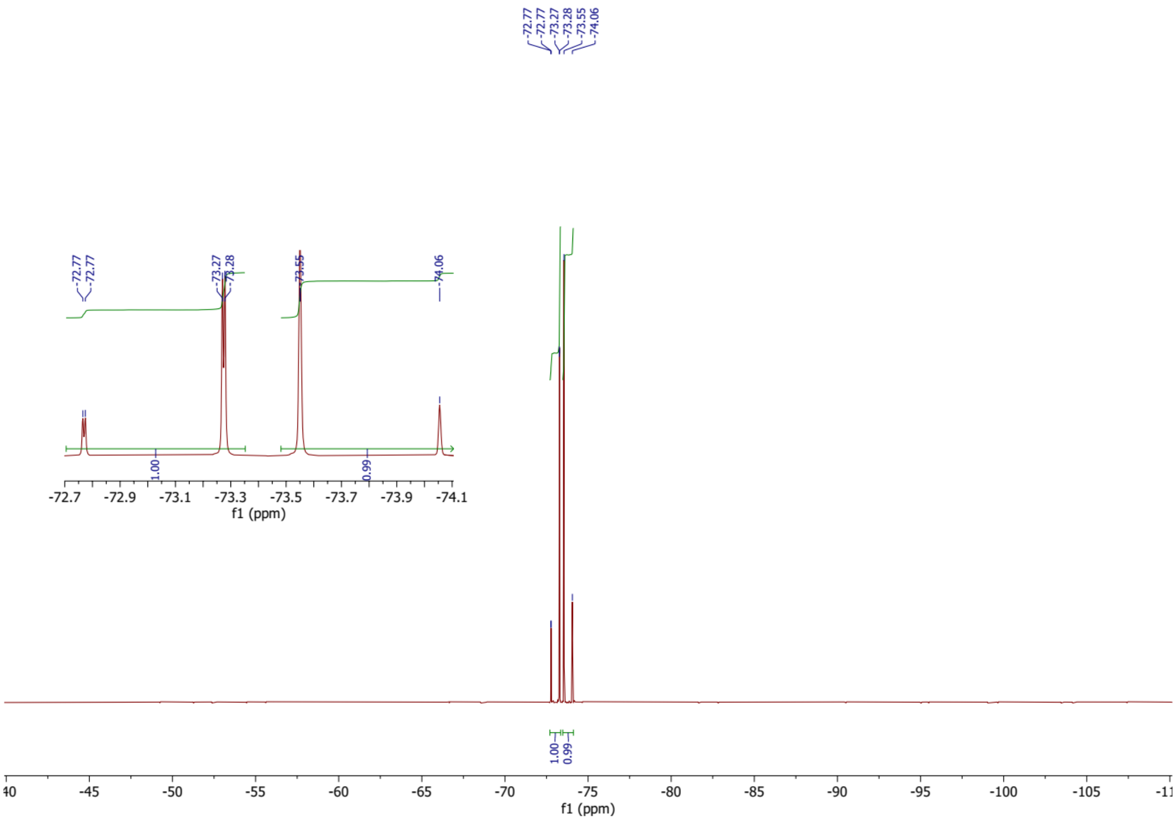
-72.77
-72.77
-73.27
-73.28
-73.55
-74.06
1.00
0.99
f1 (ppm)

6r

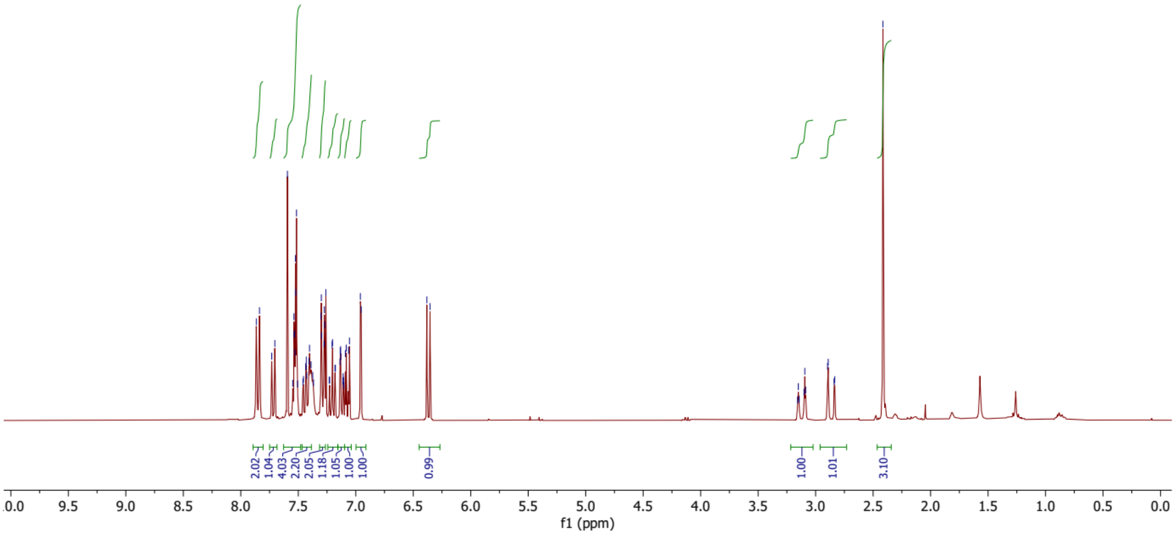
7.86 7.84 7.73 7.70 7.59 7.55 7.54 7.54 7.53 7.53 7.52 7.52 7.52 7.51 7.46 7.45 7.43 7.43 7.43 7.43 7.41 7.40 7.39 7.37 7.37 7.30 7.30 7.30 7.28 7.27 7.27 7.26 7.23 7.23 7.21 7.20 7.18 7.18 7.14 7.13 7.13 7.13 7.11 7.11 7.11 7.10 7.09 7.08 7.06 7.05 6.96 6.95 6.38 6.35
3.16 3.15 3.14 3.10 3.09 3.09 2.90 2.89 2.84 2.83
2.41
2.02 1.04 4.03 2.20 2.05 1.18 1.05 1.00 1.00
0.99
1.00
1.01
3.10
f1 (ppm)

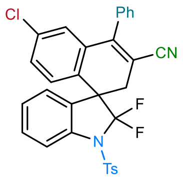
Cl
Ph
CN
F
F
N
Ts

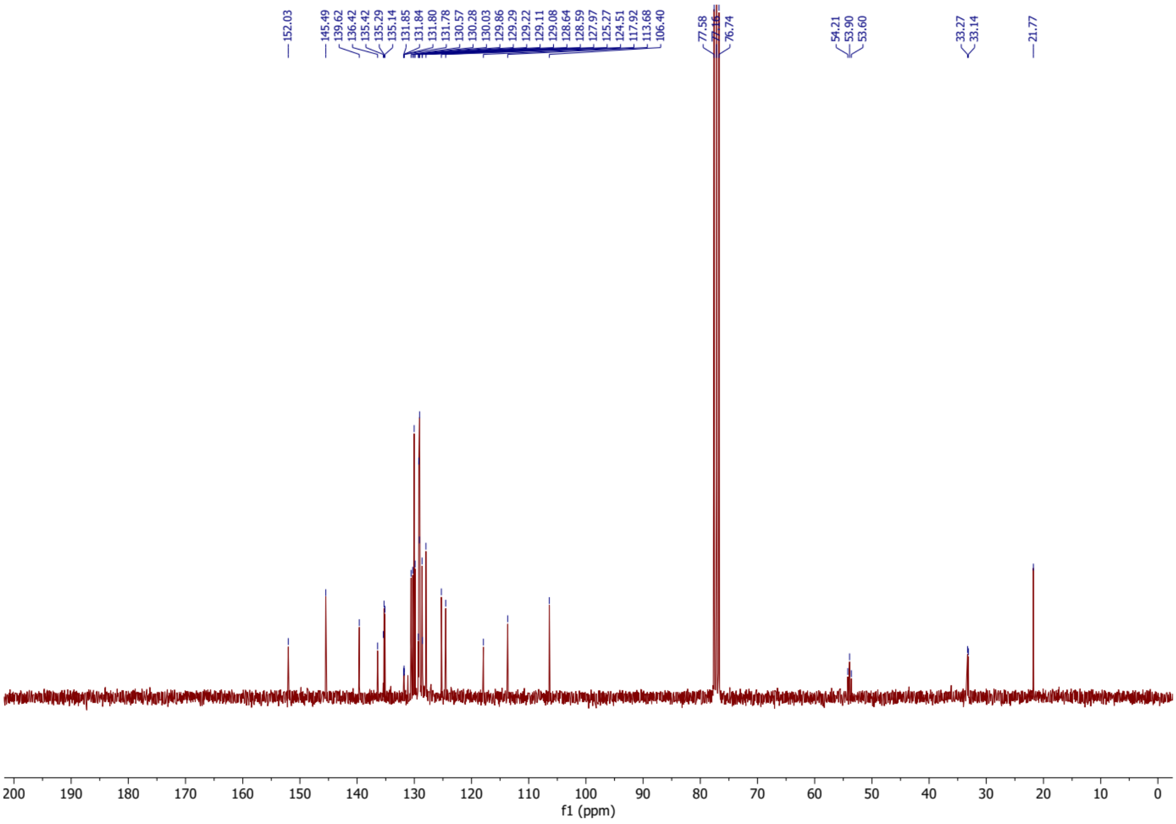
152.03
145.49 139.62 136.42 135.42 135.29 135.14 131.85 131.84 131.80 131.78 130.57 130.28 130.03 129.86 129.29 129.22 129.11 129.08 128.64 128.59 127.97 125.27 124.51 117.92 113.68 106.40
77.58 77.16 76.74
54.21 53.90 53.60
33.27 33.14
21.77
f1 (ppm)

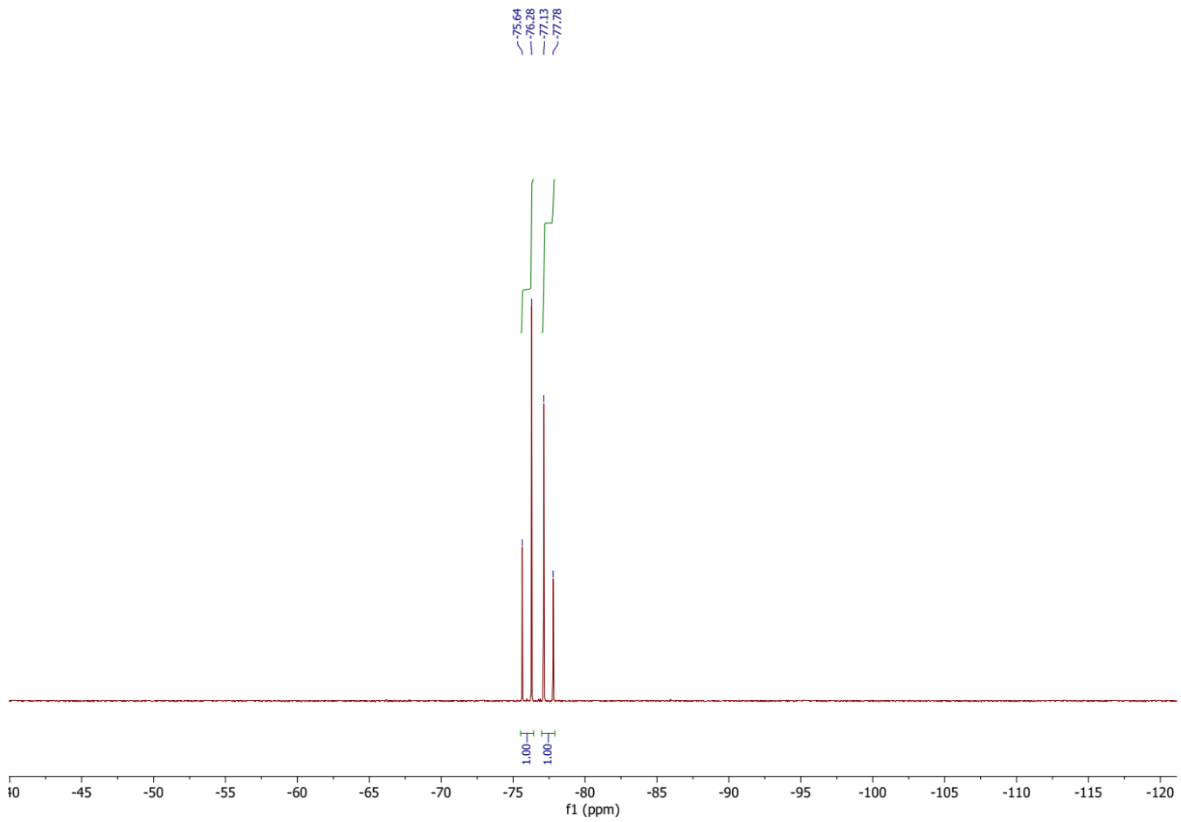
-75.64
-76.28
-77.13
-77.78
1.00
1.00
f1 (ppm)

6. Supplementary References

[1] Tang, L., Liu, Z. Y., She, W. & Feng, C. Selective single C-F bond arylation of trifluoromethylalkene derivatives. *Chem Sci*. **10,** 8701-8705 (2019).

[2] Newman, S. G. & Lautens, M. Palladium-catalyzed carboiodination of alkenes: carbon-carbon bond formation with retention of reactive functionality. *J Am Chem Soc*. **133,** 1778-1780 (2011).

[3] Ye, J.*, et al.* Remote C-H alkylation and C-C bond cleavage enabled by an in situ generated palladacycle. *Nat Chem*. **9,** 361-368 (2017).
